# Immunomic, genomic and transcriptomic characterization of CT26 colorectal carcinoma

**DOI:** 10.1186/1471-2164-15-190

**Published:** 2014-03-13

**Authors:** John C Castle, Martin Loewer, Sebastian Boegel, Jos de Graaf, Christian Bender, Arbel D Tadmor, Valesca Boisguerin, Thomas Bukur, Patrick Sorn, Claudia Paret, Mustafa Diken, Sebastian Kreiter, Özlem Türeci, Ugur Sahin

**Affiliations:** TRON gGmbH - Translational Oncology, Johannes Gutenberg-University Medical Center gGmbH, Langenbeckstr. 1, Building 708, 55131 Mainz, Germany; University Medical Center of the Johannes Gutenberg, University Mainz, 55131 Mainz, Germany; BioNTech AG, Kupferbergterrasse 17-19, 55131 Mainz, Germany

**Keywords:** Immunotherapy, Cancer models, Computational immunology, Colorectal cancer

## Abstract

**Background:**

Tumor models are critical for our understanding of cancer and the development of cancer therapeutics. Here, we present an integrated map of the genome, transcriptome and immunome of an epithelial mouse tumor, the CT26 colon carcinoma cell line.

**Results:**

We found that Kras is homozygously mutated at p.G12D, Apc and Tp53 are not mutated, and Cdkn2a is homozygously deleted. Proliferation and stem-cell markers, including Top2a, Birc5 (Survivin), Cldn6 and Mki67, are highly expressed while differentiation and top-crypt markers Muc2, Ms4a8a (MS4A8B) and Epcam are not. Myc, Trp53 (tp53), Mdm2, Hif1a, and Nras are highly expressed while Egfr and Flt1 are not. MHC class I but not MHC class II is expressed. Several known cancer-testis antigens are expressed, including Atad2, Cep55, and Pbk. The highest expressed gene is a mutated form of the mouse tumor antigen gp70. Of the 1,688 non-synonymous point variations, 154 are both in expressed genes and in peptides predicted to bind MHC and thus potential targets for immunotherapy development. Based on its molecular signature, we predicted that CT26 is refractory to anti-EGFR mAbs and sensitive to MEK and MET inhibitors, as have been previously reported.

**Conclusions:**

CT26 cells share molecular features with aggressive, undifferentiated, refractory human colorectal carcinoma cells. As CT26 is one of the most extensively used syngeneic mouse tumor models, our data provide a map for the rationale design of mode-of-action studies for pre-clinical evaluation of targeted- and immunotherapies.

**Electronic supplementary material:**

The online version of this article (doi:10.1186/1471-2164-15-190) contains supplementary material, which is available to authorized users.

## Background

Murine CT26 (Colon Tumor #26) cells were developed in 1975 by exposing BALB/c mice to N-nitroso-N-methylurethane (NMU), resulting in a rapid-growing grade IV carcinoma that is easily implanted and readily metastasizes [1]. Used in over 500 published studies, the CT26 colon carcinoma is one of the most commonly used cell lines in drug development. Numerous cytotoxic agents as well as therapeutics targeting specific signaling pathways have been studied with these cells [2–4]. Moreover, as the CT26 model in BALB/c mice provides a syngeneic in vivo test system, it is frequently used for developing and testing immunotherapeutic concepts.

In sharp contrast to its frequent use in drug development, there have been no comprehensive studies of the genome and transcriptome of CT26. Kras is mutated in CT26 [5] but other mutations are not known. Mutations in Cdkn2a, Mek, Braf and Pi3k in combination with Egfr and Vegf expression, for instance, may influence the results of pre-clinical investigations of treatment modalities. Moreover, while gp70, the product of the envelope gene of murine leukemia virus (MuLV)-related cell surface antigen, is a known model antigen for studying antigen-specific immune responses in the CT26 system, there is no comprehensive knowledge of potential tumor antigens in this cell line system.

Further, the lack of comprehensive data on the murine CT26 colon cancer data sharply contrasts to the extensive molecular characterization of human colorectal cancer (CRC). As a group, human CRC is highly heterogeneous with multiple evolutionary paths, with molecular signatures classifying subtypes and steps from adenoma to carcinoma. Many human CRC genomes are now known and multiple molecular signatures, classifications and biomarker concepts are published [6–9]. As comprehensive genomic and transcriptomic data of CT26 has not been available, it is unclear how CT26, a chemically-induced tumor, molecularly correlates to human CRC subtypes and to what extent it may be used as model.

To answer these questions, we utilized next-generation sequencing, bioinformatics and immuno-informatics to create an integrated mouse solid tumor mutanome, transcriptome and immunome, providing an overdue analysis of the CT26 cancer cell line.

## Results and discussion

**Figure 1:**
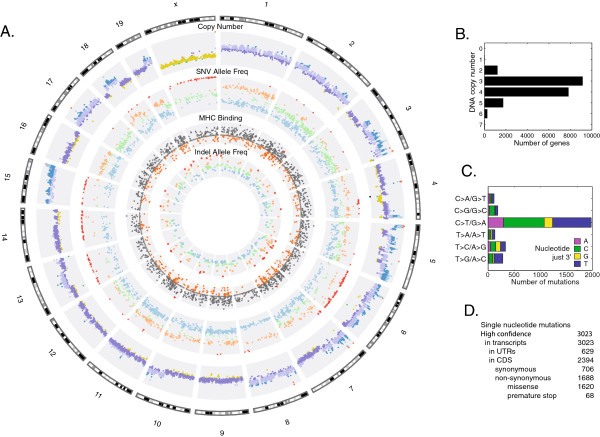
**The CT26 Genome. A)** Circos diagram showing (outer to inner): cytogenetic bands in black, gray and white. Track 1: DNA copy number. Black: deleted; green: haploid; yellow: diploid; dark purple: triploid; light purple: tetraploid; dark blue: pentaploid; bright blue: hexaploid; red: higher copy number. Track 2: High confidence point mutations, plotted based on DNA allele frequency. Inner is allele frequency 0, outer is allele frequency 100. Colors are blue (0–40), green (40–60), orange (60–90), and red (90–100). Track 3: predicted MHC binding IC50 scores for missense mutations. Mutations in peptides likely to bind MHC are colored orange (IC50 < 500 nM). Track 4: insertions and deletions plotted according to allele frequency. **B)** DNA gene copy number. **C)** Single nucleotide mutation changes and the nucleotide immediately 3′ of the mutation. **D)** Mutation classification.

*The CT26 tumor genome*: using the NGS reads, we assessed copy number and nucleotide variations by comparing CT26 to BALB/cJ DNA. We determined absolute DNA copy number using the ratio of exome-seq reads mapping to each gene from CT26 versus those from BALB/cJ, and integrating variant allele fraction (Figure 1A, outer ring). We found that the ploidy of CT26 is strikingly large with large regions of triploidy and tetraploidy, in agreement with previous karyotyping results [10]. The median and mean copy number in average across all genes is 3 and 3.5, respectively, with 8,686 genes in triploid regions (45% of the genes) and 7,448 (39%) in tetraploid regions (Figure 1B). No reads map to the Y chromosome (DNA or RNA), suggesting that CT26 cells originated from a female mouse. Only one homozygous deletion was found, which contains the tumor suppressor Cdkn2a (cyclin-dependent kinase inhibitor 2A; Ink4a) locus on mouse chromosome 4.

We identified 3,023 high-confidence single nucleotide variations (SNVs; Figure 1A, 2nd ring) and 362 short insertions and deletions (indels; Figure 1A, inner ring). Indels are dominated by A/T deletions (44%). We selected high confidence SNVs in exons (3,023; Figure 1D), the majority of which are localized in coding regions (2,394; 79%). Of the SNVs in coding regions, the majority (1,688; 71%) cause non-synonymous protein changes, including 1,620 missense and 68 nonsense variants. The CCDS database identifies 32 million protein-encoding nucleotides in the mouse genome. Relative to a 2011 BALB/cJ genome, the CT26 variation rate in coding regions is 53 non-synonymous and 22 silent mutations per Mb. This is significantly more than the average found in spontaneous human tumors (4 mutations per Mb) but still within the range observed for primary human CRC tumors, which ranges from less than 1 per Mb to over 100 mutations per Mb [11].

The identified SNVs represent variations between the CT26 genome, derived from a BALB/c mouse in 1975, and a BALB/cJ mouse in 2011. As such, the SNVs include both somatic mutations associated with the CT26 onco-transformation and genetic drift in the BALB/c genome. We found 40,000 mouse SNPs that distinguish the BALB/cJ and mm9 (C57BL/6) exomes. Of these, only 1.6% show a discrepancy between the CT26 and 2011 BALB/cJ genomes. Thus, while this does not eliminate genetic drift or conclusively identify the substrain that gave rise to CT26 cells, it demonstrates that the genome of the mouse that originally created the CT26 cells is similar to that of the current BALB/cJ mouse.

Spontaneous human CRC tumors contain primarily C > T/G > A SNVs [12]. Of the 3,023 SNVs in the CT26 genome, 2,313 (77%) are transitions, of which most (1,980, 66%) are C > T/G > A mutations (Figure 1C), similar to the human CRC mutation profile. Based on data from over 7,000 human tumors, G is the dominate nucleotide immediately 3′ of the mutated nucleotide in human CRC tumors (CG > TG mutations) [11]. Conversely, we found that CT26 SNVs are depleted in CG > TG and CA > TA mutations and enriched in CT > TT and CC > TC mutations. This pattern, a C > T mutation followed by a pyrimidine, is found in tumor samples from human patients pre-treated with temozolomide, an alkylating anticancer drug [11]. CT26 was originally induced by the alkylating agent NMU. That temozolomide and NMU are both are associated with tumors enriched in C > T mutations at positions followed by a pyrimidine suggest a similar mutagenic pattern for these two alkylating agents.

Of the 3,023 CT26 SNVs, 296 (10%) are homozygous or heterozygous (100% allele frequency, Figure 1A, 2nd ring), even in amplified regions with high copy number. Homozygous variants cluster across chromosomes 6, 13, 14, 15, and X. These regions could be the result of either a loss of heterozygosity (LOH) onco-transformation or genetic drift in a BALB/c mouse followed by inbreeding. If the result of an onco-transformation, that the regions experienced LOH, followed by mutations and copy number amplification suggests that resulting individual alleles were amplified 2-fold (chr X), 3-fold (chr 14), 4-fold (chr 6), and 5-fold (chr 15).

We further investigated chromosome X. Mutations occur on chromosome X with 100% and 50% DNA allele frequency, suggesting that chromosome X is diploid in CT26 cells. Female cells typically express XIST and inactivate one X allele. In CT26, the RNA-Seq data show that XIST is not expressed and, examining the allele expression of heterozygous mutations, that transcription occurs from both chromosome X alleles. These findings are concordant with a scenario where the chromosome X experienced both a loss of the inactivated allele and an amplification of the non-inactivated allele (occurring in either order).

In summary, the data imply that the CT26 has a complex genome of high ploidy which underwent several amplification events. Relative to a 2011 BALB/c genome, the number of mutations is higher than average, with many non-synonymous mutations. The mutation pattern reflects the treatment with the NMU alkylating agent, a similar but distinct pattern than found in spontaneous primary CRC.

*CT26 SNVs in onco-relevant genes*: we investigated whether mutations associated with CRC [12–14] are also prevalent in CT26. APC, KRAS and TP53 are frequent drivers of the linear and uniform evolution of spontaneous human CRC; of these, only Kras is mutated in CT26. The CT26 Kras genomic locus is triploid and all alleles contain V8M (located in a small molecule binding site [15]) and G12D (known to stimulate proliferation) mutations.

Several CRC subtypes are linked to syndromes based on inherited gene defects and mutations. Genes associated with familial CRC (e.g., HNPCC, Lynch Syndrome, FAP, Peutz-Jeghers) include mismatch repair genes Mlh1, Mlh2, Mlh6, Msh2, Myh, Pms1, Stk1, Mutyh and Ctnnb1. None are mutated in CT26. The lack of mutations in mismatch repair genes Mlh1 and Msh2, which are associated with CRC microsatellite instability (MSI), agrees with the lack of mutation in Braf, which is frequently associated with the MSI-high phenotype [16].

Further, the tumor suppressor Cdkn2a is homozygously deleted and the genomic Mapk1 (MEK) and Met loci are amplified in CT26. CRC-associated genes Fbxw7, Pik2ca, Pten, Smad2, Smad4, Tcf7l2 are not mutated. Non-synonymous point mutations occur in other CRC genes Brca2 (R2066K), Pdgfra (V103I), Nav3 (V154I, S334N), Atr (H792Q), Cdk8 (S87F), and Rel (A406T). Mutations in cancer-related genes include mTor (V971M), Birc2 (E395K), Casp4 (H84Y), Cenpe (A834V), Esr1 (P508S), Hdac2 (P228S), Ins1 (Y40C), Insr (A493V), Muc1 (L555F), Pik3c3 (S282A), Pik3cg (D120N), Fgfr1 (S107F), Ddr2 (A161V), Notch1 (R365S) and Rhoj (L137F). Frameshift-causing indels occur in oncogenes Ewsr1 (at amino acid 629) and Mpp3 (at amino acid 91).

**Figure 2:**
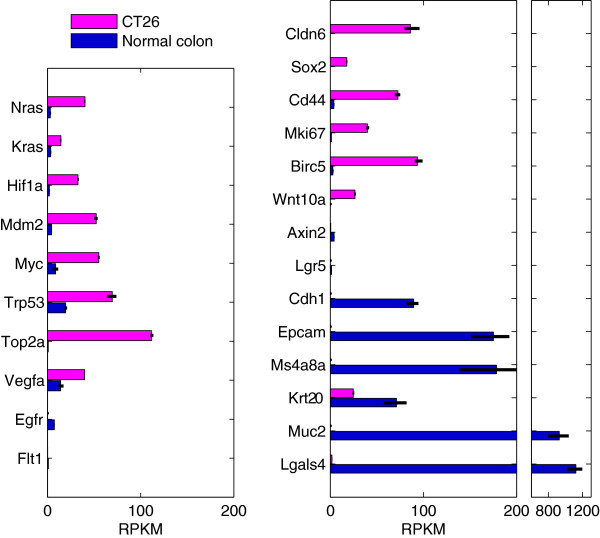
**Gene expression of onco-relevant genes, stem cell and proliferation markers, WNT signaling pathway genes, EMT and epithelial markers, and differentiation markers in CT26 and mouse colon.** Error bars represent the maximum and minimum values for the CT26 triplicates and the six normal colon samples (triplicates of one male and triplicates of one female mouse).

**Figure 3:**
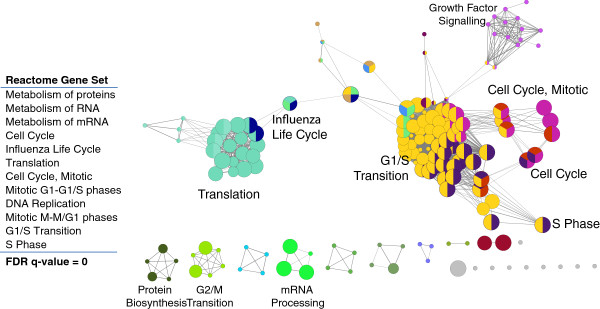
**Reactome pathways over-expressed in CT26 versus normal colon.** Enriched Reactome pathways are displayed using Cytoscape ClueGO. Ribosomal associated genes are over-expressed in CT26; while they are part of the influenza gene set, we posit their over-expression reflects increased translational activity rather than an influenza-related process. For each gene set, Cytoscape ClueGo calculates a false-discovery rate statistic (FDR q-value) reflecting the likelihood of the given enrichment by chance: the pathways listed in the table those with a zero q-value. Each dot in the image represents a separate Reactome gene set. Gene sets with common members are placed proximally, grouped into common themes, colored the same and labeled. Q-values for enriched pathways, Cytoscape settings and gene membership are in the supplementary information.

**Figure 4:**
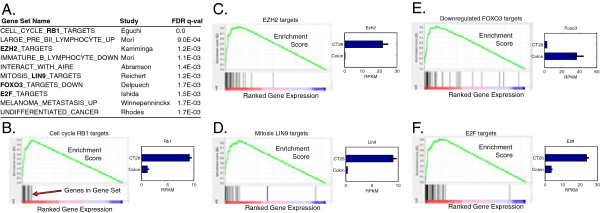
**Gene sets over-expressed in CT26 versus normal colon. A)**. The top 10 over-expressed gene sets. Gene sets included those curated from literature and overexpression was determined using GenePattern [47]. **B-F)**: gene set enrichment for the targets of specific genes, including the GenePattern enrichment plot showing the enrichment plot (green) and the location of the individual gene set members in the expression ranked-ordered list (black vertical lines). The plot on the right (blue bars) shows the expression of the targeting gene RB1 **(B)**, E2H2 **(C)**, LIN9 **(D)**, FOXO3 **(E)** and E2F1 **(F)**.

*CT26 gene expression*: we generated gene expression profiles from CT26 cells. Cancer-relevant genes such Nras, Vegfa, Trp53 (TP53), Myc, Mdm2, and Hif1a are expressed at high levels in CT26 (Figure 2, left). Egfr and Flt1 are not expressed. Gene expression in CT26 relative to normal colon was used for pathway enrichment analysis in order to identify broadly enriched pathways (Figure 3). Not surprisingly, the identified pathways relate to cell proliferation (cell cycle phases and transitions, DNA replication) and increased translation (protein and RNA metabolism). We examined individual gene sets enriched in CT26 (Figure 4). Most enriched is “CELL_CYCLE_RB1_TARGETS”, a gene set curated from a study examining RB1 target genes involved in cell cycle regulation [17], reflecting over-expression of all Rb1 target genes (Figure 4B). Rb1 mRNA is itself 8-fold up-regulated. Ezh2, downstream of the Egfr-ras-raf pathway, impacts DNA methylation, promotes EMT and is associated with poor prognosis in CRC [18, 19]. Together with its target genes, Ezh2 is over-expressed in CT26 cells. Mechanistically, that Rb1, Ezh2, Lin9, and E2f mRNAs and their target genes are over-expressed suggests that the Rb1, Ezh2, Lin9, and E2f mRNA levels, in addition to post-translational modifications, play a critical role controlling activation of each pathway.

The gene set associated with genes down-regulated after Foxo3 up-regulation was found to be up-regulated (Figure 4E). In agreement with this, Foxo3 is significantly down-regulated in CT26 cells. Foxo3 expression has been identified as a potential biomarker for CRC outcome [20], with low Foxo3 associated with 2-fold shorter survival. The low Foxo3 expression, the high Ezh2 expression and the enrichment of the “melanoma metastasis” gene set [21] are all in line with the aggressive and high metastatic activity of CT26 cells.

Differentiation markers further corroborate that CT26 cells are in a highly proliferative, undifferentiated state. The “undifferentiated cancer” gene set is highly up-regulated in the CT26 cells (Figure 4A). Stem cell markers Cldn6 and Sox2 are highly expressed while differentiation markers Muc2 and Ms4a8a (human MS4A8B) [22] markers are not expressed (Figure 2, right). Whereas Lgals1 (Galectin-1) is over 30-fold up-regulated in CT26 cells, the orthologous gene Lgals4 (Galectin-4), a differentiation marker, is over 500-fold down-regulated in CT26 cells. The proliferation markers Top2a (DNA topoisomerase 2-alpha), Mki67 and Birc5 (Survivin) are all highly expressed in CT26 cells.

Epcam marks epithelial cells and colon crypt tops [23] and is not expressed in CT26 cells. Cdh1 (e-cadherin) marks the epithelial-mesenchymal transition [24] and is highly expressed in normal colon but not expressed in CT26. CD44 marks the crypt bottoms and is 18-fold up-regulated. Silencing of WNT targets such as ASCL2, AXIN2 and LGR5 is often accomplished through CpG promoter methylation and associated with poor prognosis and increase metastatic spread [25]. In CT26, Wnt10a is highly up-regulated but WNT target genes, with the exception of Birc5, are not expressed. These markers classify CT26 as cells that originated in the lower-crypt and are in an undifferentiated state prone to metastasize [26, 27].

CRC cohort studies have identified markers for classifying patient CRC tumors (Additional file 1: Table S8). The three-group CRC classification platform using differentiation marker KRT20 and “top crypt” markers CA, MS4A12 and CD177 [6] classifies CT26 as a tumor with a less mature phenotype and worse progression. The classification platform using genes FRMD6, ZEB1, HTR2B and CDX2 [7] classifies CT26 as the “CCS3” sub-type, with poor prognosis, low therapy response and resistance to cetuximab. The 7 gene “CRCassigner-7” platform [8] classifies CT26 cells as either “stem like” or “CR-TA” (cetuximab-resistant transit-amplifying).

**Figure 5:**
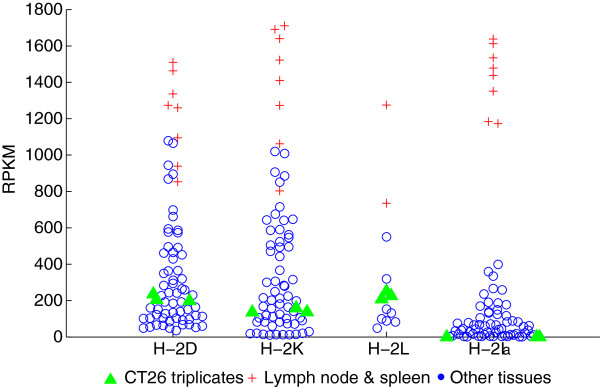
**MHC class I (H-2D, H2-K, H-2 L) and class II (H2-IA) expression in normal mouse tissues and CT26 cells.** The normal tissues are from both C57BL/6 J and BALB/c. The C57BL/6 J genome does not have the H-2 L locus.

*The CT26 cancer immunome*: immunotherapy concepts include targeting tumor-specific antigens presented on MHC molecules. We determined that CT26 cells have the same MHC types as the parental BALB/cJ mice: H-2D^d^, H-2K^d^ and H-2L^d^ (class I) and H-2la^d^ (class II). This is expected and a useful confirmation of the BALB/c-CT26 linage, given on-going reports of cell line mis-identifications. Class I loci H-2D^d^ and H-2K^d^ are expressed at levels comparable to normal tissues (Figure 5), lower than lymph node and spleen but higher than non-immune tissues (e.g., heart, kidney, brain). B2m, part of the MHC class I complex, is highly expressed. Both suggest that MHC class I is functional. Normal tissues show variable expression of MHC class II (e.g., lymph node and spleen are high, colon expresses at 150 RPKM and brain is low but non-zero). CT26 cells express neither MHC class II (0 RPKM) nor the MHC class II transactivator Ciita, suggesting that CT26 cells do not have functional MHC class II antigen presentation.

Genes with tumor-associated expression as well as genes with somatic mutations may act as tumor-associated antigens (TAAs) (Table 1). Gp70 (an endogenous envelope protein of from a MuLV-related retrovirus) is a classical model tumor antigen frequently exploited when using CT26 system to investigate CD8 T cell immunity [28]. Expression of gp70 in normal mouse tissues has been observed in mice over 8 months old [29, 30]; however, gp70 levels are strikingly high levels in murine tumor cell lines including CT26 [31]. Indeed, our data show that gp70 has the highest expression of all CT26 genes. While gp70 DNA was not captured by the NGS exome-capture, we were able to determine the gp70 sequence using the RNA-Seq reads, averaging over 5,000x coverage due to the high expression (Table 1, Additional file 1). Relative to the gp70 sequence in the mm9 genome, the CT26 gp70 sequence falls in a CT26 tetraploid region and has 9 non-synonymous mutations, including 3 homozygous and 6 heterozygous variants. Two variants are in dbSNP while three are found in Genbank mRNAs from other mouse tumor cell lines, suggesting that four could be unique to CT26 cells. Three variants introduce stop codons; however all are heterozygous such that a full length gp70 can likely be translated.

**Table 1:** **The CT26 gp70 SNVs relative to the mm9 reference genome (negative strand)**

Mm9 coordinate	Reference	Mutation	Zygosity	AA change	DbSNP 128	Observed in Genbank mRNAs?
chr8:125952138	T	A	Homo	S > T	rs30558843	Many, including CT26 [mRNA GU441834]
chr8:125951873	G	A	Hetero	W > *		No
chr8:125951822	A	G	Hetero	Y > C		CT26 [mRNA GU441834]
chr8:125951717	G	A	Hetero	W > *		No
chr8:125951634	G	A	Homo	E > K		CT26, B16 (melanoma), RCB0527-Jyg-MC(B) & RCB0526-Jyg-MC(A) (mammary)
chr8:125951556	G	T	Hetero	G > *		RCB0526-Jyg-MC(A) (mammary tumor)
chr8:125951208	G	A	Homo	G > S		CT26 [mRNA GU441834]
chr8:125950710	G	A	Hetero	E > L		RCB0526-Jyg-MC(A) (mammary tumor)
chr8:125950284	G	A	Hetero	G > R	rs30722372	No

AA, amino acid; *, stop codon.

The family of cancer testes (CT) antigens has high tumor cell selectivity. We found that CT antigens with the highest expression in CT26 cells are known colorectal CT antigens Casc5, Cep55 and Pbk (Table 2). These three, along with Atad2 and Ttk, have very low expression in the normal colon samples. Low expression of the human homologs of Casc5, Ctage5, Pbk and Spag9 has been observed in multiple tissues, such that these are cancer testes-selective antigens and they may be subject to tolerance [32]. Conversely, while expressed at 5-fold higher levels in CT26 cells, Rqcd1is also expressed at significant levels in normal colon and is thus not an ideal immunotherapy target.

**Table 2:** **Potential CT26 T-cell tumor expression antigens**

Gene	CT Antigen	CT26	Colon
Atad2	CT137	42.4	0.5
Casc5	CT29	10.2	0.1
Cep55	CT111	25.4	0.4
Ctage5	CT21	17.7	1.8
Dcaf12	CT102	11.4	7.8
Pbk	CT84	39.6	1.0
Rqcd1	CT129	30.4	5.4
Spag9	CT89	14.7	1.2
Ttk	CT96	13.1	0.3
Gp70	--	7225.4	0.0

Known CT antigens with expression above 10 RPKM in CT26 and below 10 RPKM in normal mouse colon are shown. CT26 and mouse colon expression values are in RPKM units.

In addition to tissue specific and over-expressed tumor antigens, somatic mutations provide tumor-specific immunotherapy T-cell targets [33] that may be used for truly individualized cancer therapeutics and vaccines [34]. A mutation for a cancer vaccine target must be expressed and presented on MHC molecules. Of the 3,023 CT26 point mutations, 1,172 are in expressed genes and, of these, 154 are in epitopes predicted to strongly bind to MHC molecules (highest 1% consensus percentile) (Figure 1, 3rd ring). 73 occur in highly expressed genes (at least 10 RPKM). Table 3 shows eight such point mutations that meet these criteria. For each SNV, Additional file 1: Table S2 lists the mutation-containing epitope and MHC allele predicted to have the strongest MHC binding by the IEDB algorithm [35]. Previous work by us and others [36] finds that roughly 30% of these mutations are antigenic and capable of generating a T cell response when used in immunizations. Thus, these mutations provide a broad portfolio of potentially exploitable TSAs for future studies.

**Table 3:** **Eight potential CT26 mutation antigens that are in genes expressed in CT26 and in epitopes predicted to bind MHC class I molecules based on IEDB consensus ranks**

Gene	Mutation	Epitope	MHC allele
Csnk1g3	N42K	VGP**K**FRVGKK	H-2D^d^
E2f8	I522T	**T**YLQPAQAQM	H-2K^d^
Fam111a	G213E	CVYGFK**E**ETI	H-2D^d^
Hdac2	P228S	KYYAVNFM	H-2K^d^
Nudt19	L335F	IYMT**F**PSENK	H-2K^d^
Phf3	G1814E	FPPQNMF**E**F	H-2D^d^
Smc3	D733A	KFKASR**A**SI	H-2K^d^

The bold letters signify the mutated amino acid.

^d^The official names of mouse MHC alleles.

## Conclusion

This is the first integrated genome, transcriptome and immunome map of a mouse epithelial tumor. We found that the patterns of mutations in onco-relevant genes, the gene expression signatures and the regulated pathways in CT26 cells are in agreement with their origin in colon epithelia and share features with human primary CRCs. The mutations and expression profiles are similar to those reported for sporadic, undifferentiated, therapy-refractory, metastasis-prone human CRC. Moreover, we identified non-synonymous SNVs with predicted MHC class I binding capability which, together with the robust MHC class I expression of CT26 cells, provide a valuable resource for use of the CT26 model system to develop immunotherapeutic approaches.

The integrated use of mutation allele fraction and DNA copy number allowed us to determine the absolute copy number and zygosity for each mutation. The CT26 cells have extensive triploidy and tetraploidy and a high mutation rate (53 non-synonymous mutations per Mb). While Trp53, Braf, and Pik3ca are not mutated, Kras is mutated at G12D. Similar to human CRC samples, there is a preference for C > T/G > A transitions. However, the CT26 mutation pattern shows a preference for C > T mutations at sites that are followed by a pyrimidine, a pattern that is more similar to that found in tumors from patients pre-treated with temozolomide than to that found in most human CRC tumors.

Clinically-approved patient selection biomarkers for anti-EGFR treatments cetuximab and panitumumab include assessment of EGFR levels and KRAS G12D mutation status. In CT26, we found the Kras G12D mutation and no expression of Egfr. Consistent with this, CT26 cells have been shown to be refractory to the rodent Egfr-targeting mAbs [2]. Similarly, KRAS G12D mutations and MAPK1 (MEK) and MET amplification are published biomarkers for colorectal tumor sensitivity to both MEK and MET inhibitors [3, 4]. The homozygous Kras G12D mutation and Mapk1 and Met amplifications in CT26 suggest sensitivity to MEK and MET inhibition. In concordance with this, CT26 cells have been shown to be sensitive to MEK and MET inhibitors [2, 37]. Further, the expression of markers such as Top2a and Cldn6 and lack of expression of Muc2, Epcam and Lgals4 show that CT26 cells are in an undifferentiated, proliferative state.

Our study provides an overdue genomic and transcriptomic analysis of one of the most frequently used cell lines for drug development. Further, the results form the basis for the rationale design of pre-clinical studies using this model for drug development based on detailed molecular knowledge.

## Methods

*Samples:* BALB/cJ mice (Charles River) were kept in accordance with legal policies on animal research at the University of Mainz. In 2011, Germline BALB/cJ DNA was extracted from mouse tail. CT26.WT colon carcinoma cells were purchased from the American Type Culture Collection (Product: ATCC CRL-2638, Lot Number: 58494154). 3rd and 4th passages of cells were used for tumor experiments.

*NGS sequencing and data processing*: exome capture from CT26 and BALB/cJ mice were sequenced in triplicate using the Agilent Sure-Select solution-based mouse protein coding exome capture assay. CT26 oligo(dT)-isolated RNA for gene expression profiling was prepared in triplicate. Libraries were sequenced on an Illumina HiSeq2000. Protocol details are found in the Additional file 1. DNA-derived sequence reads were aligned to the mm9 genome using bwa [38] (default options, version 0.5.8c). Ambiguous reads mapping to multiple locations of the genome were removed. RNA-derived sequence reads were aligned using bowtie [39] to the mm9 genome and RefSeq exon-exon junctions. Default and “-v2 –best” parameters were used for transcriptome and genome alignments, respectively.

For the exome reads, there was an average of 103 million read pairs per sample. As each sample was sequenced in triplicate, this resulted in over 300 million 50 nt paired-end reads for the CT26 and BALB/cJ exomes. 83% of the reads mapped to the mm9 reference genome, with 51% of the nucleotides on target, resulting in a mean coverage of 170x. The CT26 transcriptome was sequenced in triplicated with an average of 27 million reads and total of 81 million reads, of which 94% could be aligned. NGS read statistics are in Additional file 1.

*DNA copy number:* absolute allele copy number, and mutation allele fraction were simultaneously determined using a novel algorithm that assumes a) that mutation allele fraction can take only discrete values in tumor cells based on allele copy number and b) that the relative tumor to germline number of exome-seq reads mapping to a gene locus is proportional to locus copy number [40]. Copy number estimations are in Additional file 2.

*Mutation identification:* single nucleotide mutations (SNVs) that were identified by all algorithms samtools [18], Mutect [41], and SomaticSniper [42] and in the replicates were further filtered using binomial filters that eliminate erroneous tumor observations and decrease the likelihood that a mutation is classified as somatic due to lack of coverage in the germline sample. Insertions and deletions (indels) were identified using samtools and Varscan2 with at least 10 DNA reads support and further filtered by removing indels with germline support after realigning the reads to an integrated wild-type and mutated reference genome. SNVs and indels are in Additional files 3 and 4.

*SNP detection:* SNPs were detected by running the samtools mpileup command (version 0.1.19) on sites defined by dbSNP (version 128 for mm9), using the BALB/c and CT26 exome alignments as input and binning the results by the phred scaled SNP quality as returned by samtools/bcftools.

*Gene expression*: expression values were determined by counting reads overlapping transcript exons and junctions, and normalizing to RPKM expression units (Reads which map Per Kilobase of transcript length per Million mapped reads). 10 RPKM is roughly the 80th percentile (80% of the gene expression values fall below 10 RPKM). Gene expression values are in Additional file 5.

*Pathway enrichment:* the ENCODE Consortium profiled two normal mouse colons in triplicate using RNA-Seq [43]; raw data were downloaded and processed through the computational workflow used for the CT26 RNA-Seq reads. Gene expression profiles from the triplicate CT26 and six normal mouse colon RNA-Seq runs were statistically compared using a t-test. Enriched Reactome [44] gene sets were identified using GSEA [45] and Cytoscape ClueGO [46] and over-expressed genes (t-test > 20). Enriched Reactome pathways are in Additional file 6. Gene set enrichment was performed using GenePattern [47], the Molecular Signatures Database [48], and the expression ranked gene list. Enriched GenePattern gene sets are listed in Additional file 7 and gene membership is listed in Additional file 8. All identifiers were translated from mouse to human using Homologene [49]. The list of cancer testes (CT) antigens was from the CTdatabase [50].

*MHC typing and expression*: typing and expression were determined using RNA-Seq reads and the seq2HLA algorithm [51] using the parameter setting “—best” rather than “-a”. All mouse tissue samples were sequenced (RNA-Seq) by us except the normal colon dataset, which was retrieved from the ENCODE project. RNA-Seq fastq reads were mapped according to the parameters described in Boegel et al. [51]. Two distinct reference files were created for BALB/c, containing reference sequences for H-2D^d^, H-2K^d^, H-2L^d^ and H-2Ia, and for C57BL/6 containing reference sequences for H-2D^b^,H-2K^b^,H-2Ia^b^. Expression was determined by the total number of unique sequence reads mapping to class I or class II genes and normalized according to reads per kilobase of exon model per million mapped reads (RPKM) using the length of the allele transcripts contained in the reference dataset: H-2D^b^ =1567 nt, H-2K^b^ = 1564 nt, H-2Ia^b^ = 932 nt, H-2D^d^ = 1586 nt, H-2K^d^ = 1540 nt, H-2L^d^ = 1102 nt, H-2Ia^d^ = 978 nt.

*MHC binding*: MHC binding predictions were performed using the IEDB algorithm v2.5 [35], “consensus” setting, the CT26 cell-line specific MHC type and the identified somatic point mutations. The best neo-epitope for a mutation was calculated as follows: all possible 8-, 9-, 10-, 11-mer peptides containing the mutated amino acids were input to the IEDB algorithm, which predicts the binding affinity (IC50 in nM and the consensus percentile rank) of the peptide to the cell line HLA alleles. The best neo-epitope-MHC pair was defined as the peptide which has the strongest predicted binding affinity to the respective MHC allele. Epitopes with a consensus percentile rank of less than or equal to 1% are reported as likely immunogenic.

### Availability of supplementary information

CT26 and BALB/cJ NGS fastq reads are available from ENA as PRJEB5320 (RNA-Seq) and PRJEB5321 (Exome).

## Electronic supplementary material

Additional file 1: **Contains supplementary methods, supplementary tables, NGS read statistics, and the gp70 mutations and protein sequence.** (DOCX 49 KB)

Additional file 2: **Absolute copy number for each gene determined using the number of exome-seq reads mapping to each gene from CT26 and balb/c samples and using the allele fraction to determine ploidy.** Columns include gene, copy number, normalized ratio, chromosome and gene start coordinate in the mm9 assembly. (XLSX 888 KB)

Additional file 3: **The 3,023 high confidence point mutations found in CT26 transcripts.** Columns include chromosomal position of the mutation, reference and observed nt, classification non/synonymous, classification UTR/CDS, amino acid substitution, gene symbol, transcript ID and affected exon, mean gene expression (combined of gene expression and exon expression), possible repeat region, dbSNP ID and dbSNP validation source, MHC prediction for MHC class I and class II alleles for the mutated neo-epitope and corresponding wild type peptide, allele to which it is binding, percentile rank, neo-epitope sequence, IC 50 [nM]).The mutation-containing epitope and associated MHC allele were selected using the IEDB algorithm v2.5 [3], with consensus setting, with the listed epitope and MHC being the pair with the predicted strongest binding. (XLSX 561 KB)

Additional file 4: **The 363 insertion and deletion mutations.** Columns include chromosomal position of the mutation, reference and observed nt(s), location of indel, frameshift mutation, classification non/synonymous, in UTR, gene symbol, UCSC transcript ID (if the mutation can occur in more than one transcript of this gene they are separated by a space), transcript ID and affected exon, mean expression (combined of gene expression and exon expression), possible repeat region, dbSNP ID and dbSNP validation source, allele frequency for CT26 and Balb/c replicates, number of reads per sample supporting this indel. (XLSX 97 KB)

Additional file 5: **Gene expression values for CT26 and ENCODE normal mouse colon samples in RPKM values and raw read counts.** (XLSX 13 MB)

Additional file 6: **Results of the GSEA gene set Reactome pathway enrichment, including Reactome pathway name, gene membership and FDR q-values.** (XLSX 38 KB)

Additional file 7: **Results of the GenePattern enrichment using ranked ordered expression.** Gene sets included those curated from literature and overexpression was determined using GenePattern [4]. Gene membership and enrichment values are in the file. (XLSX 568 KB)

Additional file 8: **Contains the Gene Pattern gene set membership and enrichment values in an html format.** The file index.html is the entry point. (ZIP 13 MB)
