# Supplementary material for: Immunomic, genomic and transcriptomic characterization of CT26 colorectal carcinoma
Source: BMC Genomics. 2014 Mar 13;15(1):190. doi: 10.1186/1471-2164-15-190 (PMC4007559; doi:10.1186/1471-2164-15-190)
Supplement: Supplementary file 1 — Additional file 1: Contains supplementary methods, supplementary tables, NGS read statistics, and the gp70 mutations and protein sequence. (DOCX 49 KB) [file 12864_2013_7028_MOESM1_ESM.docx]

***Supplementary text for***

**Characterization of the CT26 colorectal carcinoma genome, transcriptome and immunome**

John C. Castle^1^, Martin Loewer^1^, Sebastian Boegel^1,2^, Jos de Graaf^1^, Christian Bender^1^, Arbel Tadmor^1^, Valesca Boisguerin^1,4^, Thomas Bukur^1^, Patrick Sorn^1^, Claudia Paret^1^, Mustafa Diken^1^, Sebastian Kreiter^1^, Özlem Türeci^3^, Ugur Sahin^1,2,4^

*^1^ TRON gGmbH - Translational Oncology at Johannes Gutenberg-University Medical Center gGmbH, Langenbeckstr. 1, Building 708, 55131 Mainz, Germany*

*^2^ University Medical Center of the Johannes Gutenberg-University Mainz, III. Medical Department, 55131, Mainz, Germany*

*^3^ Ganymed Pharmaceuticals AG, 55131 Mainz, Germany*

*^4^ BioNTech AG, Kupferbergterrasse 17-19, 55131 Mainz, Germany*

Ugur Sahin ([sahin@uni-mainz.de](mailto:sahin@uni-mainz.de)), John Castle ([john.castle@tron-mainz.de](mailto:john.castle@tron-mainz.de))

TRON - Translational Oncology at the Johannes Gutenberg University of Mainz Medicine

Langenbeckstr. 1, Building 708, 55131 Mainz, Germany

Table of Contents

[Supplementary Methods 2](#_Toc380481068)

[Supplementary Tables 3](#_Toc380481069)

[Therapies for CT26 4](#_Toc380481070)

[NGS read statistics 5](#_Toc380481071)

[The gp70 variants and protein sequence 6](#_Toc380481072)

[Supplementary References 8](#_Toc380481073)

Supplementary files are available at <http://tron-mainz.de/tron-facilities/computational-medicine/ct26/>.

# Supplementary Methods

*Nucleic acid extraction:* DNA and RNA from bulk CT26.WT cells and DNA from balb/c tail tissue were extracted in triplicate using Qiagen DNeasy Blood and Tissue kit and a combination of Trizol (Invitrogen) and Qiagen RNeasy Micro kit, respectively.

*DNA exome sequencing*: Exome capture for DNA resequencing was performed in triplicate using the Agilent Sure-Select mouse exome solution-based capture assay, designed to capture all mouse protein coding regions. 3 µg purified genomic DNA (gDNA) was fragmented to 150-200 bp using a Covaris S2 ultrasound device. Fragments were end repaired and 5’ phosphorylated and 3’ adenylated according to the manufacturer’s instructions (New England Biolabs) Illumina specific paired end adapters were ligated to the gDNA fragments (10:1 molar ratio of adapter to gDNA). Following pre-capture enrichment PCR flow cell specific sequences were added using Illumina PE PCR primers 1.0 and 2.0 for 4 PCR cycles (Agilent, Herculase II). 500 ng of adapter ligated, PCR enriched gDNA fragments were hybridized to Agilent’s SureSelect biotinylated mouse whole exome RNA library baits for 24 hrs at 65 °C. Hybridized gDNA/RNA bait complexes where removed using streptavidin coated magnetic beads, washed and the RNA baits cleaved off during elution in SureSelect elution buffer. These eluted gDNA fragments were PCR amplified post capture for 10 cycles (Agilent, Herculase II). Exome enriched gDNA libraries were clustered on the cBot using Truseq SR cluster kit v2.5 with 7 pM template and 2 X 101 bps were sequenced on the Illumina HiSeq2000 using Truseq SBS kit-HS.

*RNA gene expression profiling (RNA-Seq):* Barcoded mRNA-seq cDNA libraries were prepared in triplicate (modified Illumina mRNA-seq protocol). mRNA was isolated from 5 µg total RNA using Seramag Oligo(dT) magnetic beads (Thermo Scientific) and fragmented using divalent cations and heat. Fragments (160-220 bp) were converted to cDNA using random primers and SuperScriptII (Invitrogen) followed by second strand synthesis using DNA polymerase I and RNaseH. cDNA was end repaired, 5’ phosphorylated and 3’ adenylated according to the manufacturer’s instructions. 3’ single T-overhang Illumina multiplex specific adapters were ligated with T4 DNA ligase (10:1 molar ratio of adapter to cDNA insert). cDNA libraries were purified and size selected at 200-220 bp (E-Gel 2% SizeSelect gel, Invitrogen). Enrichment, adding of Illumina six base index and flow cell specific sequences was done by PCR using Phusion DNA polymerase (Finnzymes). All clean-ups up to this step were done with 1.8x volume of Agencourt AMPure XP magnetic beads. All quality controls were done using Invitrogen’s Qubit HS assay and fragment size was determined using Agilent’s 2100 Bioanalyzer HS DNA assay. Barcoded RNA-Seq libraries were clustered on the cBot using Truseq PE cluster kit v2.5 with 7 pM template and 1 X 50 nt reads were sequenced on the Illumina HiSeq2000 using Truseq SBS kit-HS 50 generating an average of 27.2 million reads per replicate (4.08 GB in total).

*NGS data processing*: DNA-derived sequence reads were aligned to the mm9 genome using bwa [[1](#_ENREF_1)] (default options, version 0.5.8c). Ambiguous reads mapping to multiple locations of the genome were removed. RNA-derived sequence were aligned using bowtie [[2](#_ENREF_2)] to the mm9 genome and RefSeq exon-exon junctions. Default and “-v2 –best” parameters were used for transcriptome and genome alignments, respectively.

# Supplementary Tables

Supplementary files are available at <http://tron-mainz.de/tron-facilities/computational-medicine/ct26/>.

**Table S1** (separate excel file). Absolute copy number for each gene determined using the number of exome-seq reads mapping to each gene from CT26 and balb/c samples and using the allele fraction to determine ploidy. Columns include gene, copy number, normalized ratio, chromosome and gene start coordinate in the mm9 assembly.

**Table S2** (separate excel file). The 3,023 high confidence point mutations found in CT26 transcripts Columns include chromosomal position of the mutation, reference and observed nt, classification non/synonymous, classification UTR/CDS, amino acid substitution, gene symbol, transcript ID and affected exon, mean gene expression (combined of gene expression and exon expression), possible repeat region, dbSNP ID and dbSNP validation source, MHC prediction for MHC class I and class II alleles for the mutated neo-epitope and corresponding wild type peptide, allele to which it is binding, percentile rank, neo-epitope sequence, IC 50 [nM]). The mutation-containing epitope and associated MHC allele were selected using the IEDB algorithm v2.5 [[3](#_ENREF_3)], with consensus setting, with the listed epitope and MHC being the pair with the predicted strongest binding.

**Table S3** (separate excel file). The 363 insertion and deletion mutations. Columns include chromosomal position of the mutation, reference and observed nt(s), location of indel, frameshift mutation, classification non/synonymous, in UTR, gene symbol, UCSC transcript ID (if the mutation can occur in more than one transcript of this gene they are separated by a space), transcript ID and affected exon, mean expression (combined of gene expression and exon expression), possible repeat region, dbSNP ID and dbSNP validation source, allele frequency for CT26 and Balb/c replicates, number of reads per sample supporting this indel.

**Table S4** (separate excel file). Gene expression values for CT26 and ENCODE normal mouse colon samples in RPKM values and raw read counts. Note: the first 50 genes are provided as an example; all values (25Mb) will be provided on our website.

**Table S5** (separate excel file). Results of the GSEA gene set Reactome pathway enrichment, including Reactome pathway name, gene membership and FDR q-values.

**Table S6** (separate excel file). Results of the GenePattern enrichment using ranked ordered expression . Gene sets included those curated from literature and overexpression was determined using GenePattern [[4](#_ENREF_4)]. Gene membership and enrichment values are in the file Files.CT26_GseaReport.zip.

**Files.CT26_GseaReport.zip** (zip file) contains the Gene Pattern gene set membership and enrichment values in an html format. The file index.html is the entry point.

# Therapies for CT26

**Table S7.** Therapeutic decision making for CT26 associated with molecular profiling

| Therapy | Molecular prediction | Confidence | Biomarker status in tumor | Outcome |
| --- | --- | --- | --- | --- |
| EGFR mAbs | Refractory | Regulatory | Egfr not expression | Refractory |
|  |  | Regulatory | Kras G12D mutated |  |
| MET inhibitors | Responsive | Non-regulatory | Kras G12D mutated | Responsive |
|  |  | Non-regulatory | Mek over-expressed |  |
| MEK inhibitors | Responsive | Non-regulatory | Kras G12D mutated | Responsive |
|  |  | Non-regulatory | Mapk1 over-expressed |  |

**Table S8.** CRC multi-gene molecular prognostic and stratification biomarker assays projected into CT26, including assays KRT20/top-crypt [[5](#_ENREF_5)], CRCassigner-7 [[6](#_ENREF_6)], and CCS test [[7](#_ENREF_7)]. Expression values (RPKM units) are shown for the CT26 and normal mouse colon samples. “Not in mouse” indicates that the human gene does not have a clear mouse homolog. The column “predicted prognosis” would be the output of the cited assay for a patient with a CT26 colon tumor.

| Assay | Marker | Type | CT26 | Colon | Predicted Prognosis |
| --- | --- | --- | --- | --- | --- |
| KRT20/top-crypt | KRT20 | Differentiation | 25 | 60 | poor progression; immature phenotype |
|  | CA1 | Top-crypt | 0 | 600 |  |
|  | CD177 | Top-crypt | 0 | 50 |  |
|  | SLC26A3 | Top-crypt | 0 | 60 |  |
|  | MS4A12 | Top-crypt | not in mouse | |  |
| CRCassigner-7 | SFRP2 | Stem-like | 0 | 4 | cetuximab-resistant; suggest FOLFIRI chemotherapy treatment or cMET inhibitors |
|  | ZEB1 | Stem-like | 6 | 1 |  |
|  | RARRES3 | Inflammatory | not in mouse | |  |
|  | CFTR | CR/CS | 0 | 2 |  |
|  | FLNA+ | CR-TA | 135 | 133 |  |
|  | FLNA- | CS-TA | 135 | 133 |  |
|  | MUC2 | Goblet/enterocyte | 0 | 928 |  |
|  | TFF3+ | Goblet | 0 | 678 |  |
|  | TFF3- | Enterocyte | 0 | 678 |  |
| CCS test | FRMD6 | CCS3 | 15 | 2 | poor prognosis; resistant to cetuximab |
|  | ZEB1 | CCS3 | 6 | 1 |  |
|  | HTR2B | CCS1 | 0 | 0 |  |
|  | CDX2 | CCS1 | 0 | 97 |  |

# NGS read statistics

**Table S9.** Statistics for the NGS exome reads.

| **library** | **read pairs** | **Reads** | **alignments** | **% aligned** | **MEAN TARGET COVERAGE** | **BASES ALIGNED** | **ON TARGET BASES** | **% ON TARGET BASES** |
| --- | --- | --- | --- | --- | --- | --- | --- | --- |
| **CT26_1** | 106,887,642 | 213,775,284 | 177,305,001 | 83 | 180 | 17,662,037,540 | 9,306,350,647 | 53 |
| **CT26_2** | 106,165,194 | 212,330,388 | 176,033,578 | 83 | 172 | 17,527,476,788 | 8,905,644,714 | 51 |
| **CT26_3** | 102,258,682 | 204,517,364 | 169,100,287 | 83 | 169 | 16,837,655,377 | 8,687,559,176 | 52 |
| **Balb/c_1** | 102,738,279 | 205,476,558 | 170,977,320 | 83 | 166 | 17,018,105,174 | 8,558,169,633 | 50 |
| **Balb/c_2** | 99,229,991 | 198,459,982 | 165,663,210 | 83 | 162 | 16,468,919,879 | 8,319,403,361 | 51 |
| **Balb/c_3** | 103,456,359 | 206,912,718 | 172,220,910 | 83 | 171 | 17,125,576,168 | 8,770,291,629 | 51 |

**Table S10.** Statistics for the NGS CT26 RNA-Seq reads.

| **library** | **reads** | **aligned** | **%aligned** |
| --- | --- | --- | --- |
| **CT26_1** | 21,299,236 | 20,020,856 | 94 |
| **CT26_2** | 34,668,157 | 33,031,790 | 95 |
| **CT26_3** | 25,820,372 | 24,020,744 | 93 |

# The gp70 variants and protein sequence

The nine SNVs in gp70 relative to the mm9 reference genome, negative strand.

| mm9 coordinate | Reference  (- strand) | Mutation  (- strand) | Zygosity | AA change | dbSNP 128 | Observed in Genbank mRNAs? |
| --- | --- | --- | --- | --- | --- | --- |
| chr8:125952138 | T | A | Homo | S>T | rs30558843 | Many, including CT26 [mRNA GU441834] |
| chr8:125951873 | G | A | Hetero | W>* |  | No |
| chr8:125951822 | A | G | Hetero | Y>C |  | CT26 [mRNA GU441834] |
| chr8:125951717 | G | A | Hetero | W>* |  | No |
| chr8:125951634 | G | A | Homo | E>K |  | CT26, B16 (melanoma), RCB0527-Jyg-MC(B) & RCB0526-Jyg-MC(A) (mammary) |
| chr8:125951556 | G | T | Hetero | G>* |  | RCB0526-Jyg-MC(A) (mammary tumor) |
| chr8:125951208 | G | A | Homo | G>S |  | CT26 [mRNA GU441834] |
| chr8:125950710 | G | A | Hetero | E>L |  | RCB0526-Jyg-MC(A) (mammary tumor) |
| chr8:125950284 | G | A | Hetero | G>R | rs30722372 | No |

The gp70 nucleotide sequence with homozygous variants found in CT26 cells

>gp70 [mm9; chr8:125,950,261-125,952,324; negative strand]

atggatacacgccgcccacgtcaaggcagcgaccacacccccgataaaaccatcatggagagtacaacgctctcaaaaccctttaaaaatcaggttaacccgtggggccccctaattgtccttctgattctcggaggggtcaaccccgttgcgttgggaaacagcccccaccaggtttttaacctcacctgggaagtgactaatggagaccgagaaacggtgtgggcaataaccggcaatcaccctctgtggacttggtggcctgacctcacaccagatctctgtatgttggccctccacgggccgtcctattggggcctagaatatcgggctcctttttctcctcccccggggcccccctgctgttcaggaagcagcgactccacgccaggctgttccagagattgtgaggagcccctgacttcatatactccccggtgcaatacggcctggaacagacttaagttatctaaagtgacacatgcccacaatgaaggattctatgtctgccccgggccacatcgcccccggtgggcccggtcgtgtggtggtccagaatccttctattgtgcctcttggggctgcgaaaccacaggccgagcatcctggaaaccatcctcgtcctgggactacatcacagtaagcaacaatctaacctcagaccaggcaaccccagtatgcaaaggtaataagtggtgcaactccttaactatccggttcacgagctttggaaaacaggccacctcctgggtcacaggccattggtggggattgcgcctatacgtctctggacatgacccagggctcatctttgggatccgacttaaaattacagactcggggccccgggtcccaatagggccaaaccccgtcttgtcagaccgacgaccaccttcccggcctagacccaccagatctcccccgccttcaaactccaccccaaccgagacacccctcaccctccccgaacccccgccagcgggagtcgaaaaccgattgttaaatctagtaaaaggagcctaccaagccctcaacctcaccagtcctgataaaacccaagagtgctggttatgcctagtatcgggacccccatactacgagggggttgccgtcctaagtacctactccaaccatacttctgccccagctaactgctctgtggcctctcaacacaaattgaccttgtccgaagtgaccggacagggactctgcataggagcggtccctaaaacccatcaagtcttgtgtaataccacccaaaagacaagcgatgggtcctactatttggccgctcccacaggaactacctgggcttgtagtactggactcactccctgtatctcaaccaccatacttgacctcaccaccgattactgtgtcctggtcgagctttggccaagggtgacctaccattcccctagttatgtttaccaccaatttgaaagacgagccaaatataaaagagaacccgtctcactaactctggccctactattaggaggactcactatgggcggaattgccgctggagtgggaacagggactaccgccctagtggccactcagcagttccaacaactccaggctgccatgcacgatgaccttaaagaagttgaaaagtccatcactaatctagaaaaatctttgacctccttgtccgaagtagtgttacagaatcgtagaggcctagatctactattcctaaaagagggaggtttgtgtgctgccttaaaagaagaatgctgtttctatgccgaccacacaggattggtacgggatagcatggccaaacttagagaaagattgagtcagagacaaaagctctttgaatcccaacaagggtggtttgaagggctgtttaataagtccccttggttcaccaccctgatatccaccatcatgggtcccctgataatcctcttgttaattttactctttgggccttgtattctcaatcgcctggtccagtttatcaaagacaggatttcggtagtgcaggccctggttctgactcaacaatatcatcaacttaagacaataggagattgtaaatcacgtgaataa

The gp70 protein sequence as found a) in CT26 cells, including homozygous (red) and heterozygous (blue) mutations, b) in CT26 cells, including only homozygous mutations (red) and c) in the mm9 mouse genome. The gp70 locus is tetraploid; the mutations have not been phased according to allele.

>gp70_with_all_CT26_variations

MDTRRPRQGSDHTPDKTIMESTTLSKPFKNQVNPWGPLIVLLILGGVNPVALGNSPHQVFNL**T**WEVTNGDRETVWAITGNHPLWTWWPDLTPDLCMLALHGPSYWGLEYRAPFSPPPGPPCCSGSSDSTPGCSRDCEEPLTSYTPRCNTA*****NRLKLSKVTHAHNEGF**C**VCPGPHRPRWARSCGGPESFYCASWGCETTGRAS*****KPSSSWDYITVSNNLTSDQATPVCKGN**K**WCNSLTIRFTSFGKQATSWVTGHWW*****LRLYVSGHDPGLIFGIRLKITDSGPRVPIGPNPVLSDRRPPSRPRPTRSPPPSNSTPTETPLTLPEPPPAGVENRLLNLVKGAYQALNLTSPDKTQECWLCLVSGPPYYEGVAVL**S**TYSNHTSAPANCSVASQHKLTLSEVTGQGLCIGAVPKTHQVLCNTTQKTSDGSYYLAAPTGTTWACSTGLTPCISTTILDLTTDYCVLVELWPRVTYHSPSYVYHQFERRAKYKREPVSLTLALLLGGLTMGGIAAGVGTGTTALVATQQFQQLQAAMHDDLKEV**L**KSITNLEKSLTSLSEVVLQNRRGLDLLFLKEGGLCAALKEECCFYADHTGLVRDSMAKLRERLSQRQKLFESQQGWFEGLFNKSPWFTTLISTIMGPLIILLLILLFGPCILNRLVQFIKDRISVVQALVLTQQYHQLKTI**R**DCKSRE*

>gp70_CT26_with_ homozygous_variations

MDTRRPRQGSDHTPDKTIMESTTLSKPFKNQVNPWGPLIVLLILGGVNPVALGNSPHQVFNL**T**WEVTNGDRETVWAITGNHPLWTWWPDLTPDLCMLALHGPSYWGLEYRAPFSPPPGPPCCSGSSDSTPGCSRDCEEPLTSYTPRCNTAWNRLKLSKVTHAHNEGFYVCPGPHRPRWARSCGGPESFYCASWGCETTGRASWKPSSSWDYITVSNNLTSDQATPVCKGN**K**WCNSLTIRFTSFGKQATSWVTGHWWGLRLYVSGHDPGLIFGIRLKITDSGPRVPIGPNPVLSDRRPPSRPRPTRSPPPSNSTPTETPLTLPEPPPAGVENRLLNLVKGAYQALNLTSPDKTQECWLCLVSGPPYYEGVAVL**S**TYSNHTSAPANCSVASQHKLTLSEVTGQGLCIGAVPKTHQVLCNTTQKTSDGSYYLAAPTGTTWACSTGLTPCISTTILDLTTDYCVLVELWPRVTYHSPSYVYHQFERRAKYKREPVSLTLALLLGGLTMGGIAAGVGTGTTALVATQQFQQLQAAMHDDLKEVEKSITNLEKSLTSLSEVVLQNRRGLDLLFLKEGGLCAALKEECCFYADHTGLVRDSMAKLRERLSQRQKLFESQQGWFEGLFNKSPWFTTLISTIMGPLIILLLILLFGPCILNRLVQFIKDRISVVQALVLTQQYHQLKTIGDCKSRE*

>gp70_mm9_genome

MDTRRPRQGSDHTPDKTIMESTTLSKPFKNQVNPWGPLIVLLILGGVNPVALGNSPHQVFNLSWEVTNGDRETVWAITGNHPLWTWWPDLTPDLCMLALHGPSYWGLEYRAPFSPPPGPPCCSGSSDSTPGCSRDCEEPLTSYTPRCNTAWNRLKLSKVTHAHNEGFYVCPGPHRPRWARSCGGPESFYCASWGCETTGRASWKPSSSWDYITVSNNLTSDQATPVCKGNEWCNSLTIRFTSFGKQATSWVTGHWWGLRLYVSGHDPGLIFGIRLKITDSGPRVPIGPNPVLSDRRPPSRPRPTRSPPPSNSTPTETPLTLPEPPPAGVENRLLNLVKGAYQALNLTSPDKTQECWLCLVSGPPYYEGVAVLGTYSNHTSAPANCSVASQHKLTLSEVTGQGLCIGAVPKTHQVLCNTTQKTSDGSYYLAAPTGTTWACSTGLTPCISTTILDLTTDYCVLVELWPRVTYHSPSYVYHQFERRAKYKREPVSLTLALLLGGLTMGGIAAGVGTGTTALVATQQFQQLQAAMHDDLKEVEKSITNLEKSLTSLSEVVLQNRRGLDLLFLKEGGLCAALKEECCFYADHTGLVRDSMAKLRERLSQRQKLFESQQGWFEGLFNKSPWFTTLISTIMGPLIILLLILLFGPCILNRLVQFIKDRISVVQALVLTQQYHQLKTIGDCKSRE*

# Supplementary References

1. Li H, Durbin R: **Fast and accurate short read alignment with Burrows-Wheeler transform.** *Bioinformatics* 2009, **25:**1754-1760.

2. Langmead B, Trapnell C, Pop M, Salzberg SL: **Ultrafast and memory-efficient alignment of short DNA sequences to the human genome.** *Genome Biol* 2009, **10:**R25.

3. Kim Y, Sette A, Peters B: **Applications for T-cell epitope queries and tools in the Immune Epitope Database and Analysis Resource.** *J Immunol Methods* 2011, **374:**62-69.

4. Reich M, Liefeld T, Gould J, Lerner J, Tamayo P, Mesirov JP: **GenePattern 2.0.** *Nat Genet* 2006, **38:**500-501.

5. Dalerba P, Kalisky T, Sahoo D, Rajendran PS, Rothenberg ME, Leyrat AA, Sim S, Okamoto J, Johnston DM, Qian D, et al: **Single-cell dissection of transcriptional heterogeneity in human colon tumors.** *Nat Biotechnol* 2011, **29:**1120-1127.

6. Sadanandam A, Lyssiotis CA, Homicsko K, Collisson EA, Gibb WJ, Wullschleger S, Ostos LC, Lannon WA, Grotzinger C, Del Rio M, et al: **A colorectal cancer classification system that associates cellular phenotype and responses to therapy.** *Nat Med* 2013, **19:**619-625.

7. De Sousa EMF, Wang X, Jansen M, Fessler E, Trinh A, de Rooij LP, de Jong JH, de Boer OJ, van Leersum R, Bijlsma MF, et al: **Poor-prognosis colon cancer is defined by a molecularly distinct subtype and develops from serrated precursor lesions.** *Nat Med* 2013, **19:**614-618.
