# Supplementary material for: Immunomic, genomic and transcriptomic characterization of CT26 colorectal carcinoma
Source: BMC Genomics. 2014 Mar 13;15(1):190. doi: 10.1186/1471-2164-15-190 (PMC4007559; doi:10.1186/1471-2164-15-190)
Supplement: Supplementary file 8 — Additional file 8: Contains the Gene Pattern gene set membership and enrichment values in an html format. The file index.html is the entry point. (ZIP 13 MB) [file 12864_2013_7028_MOESM8_ESM.zip › ABRAMSON_INTERACT_WITH_AIRE.html]

Details for gene set ABRAMSON\_INTERACT\_WITH\_AIRE[GSEA]

|  || Dataset | CT26\_gene\_expression |
| Phenotype | NoPhenotypeAvailable |
| Upregulated in class | na\_pos |
| GeneSet | ABRAMSON\_INTERACT\_WITH\_AIRE |
| Enrichment Score (ES) | 0.82206637 |
| Normalized Enrichment Score (NES) | 1.7266861 |
| Nominal p-value | 0.0 |
| FDR q-value | 0.0014402692 |
| FWER p-Value | 0.0080 |
Table: GSEA Results Summary

  

Fig 1: Enrichment plot: ABRAMSON\_INTERACT\_WITH\_AIRE      
 Profile of the Running ES Score & Positions of GeneSet Members on the Rank Ordered List

  

| PROBE | GENE SYMBOL | GENE\_TITLE | RANK IN GENE LIST | RANK METRIC SCORE | RUNNING ES | CORE ENRICHMENT || 1 | TOP2A |  |  | 8 | 56.000 | 0.0893 | Yes |
| 2 | RAD21 |  |  | 86 | 31.400 | 0.1348 | Yes |
| 3 | KPNB1 |  |  | 101 | 30.100 | 0.1822 | Yes |
| 4 | XPO1 |  |  | 148 | 27.000 | 0.2226 | Yes |
| 5 | NASP |  |  | 161 | 26.700 | 0.2647 | Yes |
| 6 | MCM6 |  |  | 163 | 26.600 | 0.3073 | Yes |
| 7 | SMC3 |  |  | 265 | 22.600 | 0.3372 | Yes |
| 8 | SUPT16H |  |  | 276 | 22.400 | 0.3725 | Yes |
| 9 | MSH6 |  |  | 338 | 21.000 | 0.4023 | Yes |
| 10 | MYBBP1A |  |  | 355 | 20.700 | 0.4345 | Yes |
| 11 | XPOT |  |  | 365 | 20.500 | 0.4668 | Yes |
| 12 | PCNA |  |  | 386 | 20.200 | 0.4980 | Yes |
| 13 | IPO7 |  |  | 423 | 19.500 | 0.5270 | Yes |
| 14 | PARP1 |  |  | 444 | 19.200 | 0.5565 | Yes |
| 15 | RANBP2 |  |  | 605 | 17.300 | 0.5741 | Yes |
| 16 | PABPC1 |  |  | 677 | 16.700 | 0.5963 | Yes |
| 17 | POLR2B |  |  | 699 | 16.400 | 0.6213 | Yes |
| 18 | NUP93 |  |  | 951 | 14.500 | 0.6286 | Yes |
| 19 | DDX5 |  |  | 1021 | 14.100 | 0.6468 | Yes |
| 20 | CAND1 |  |  | 1039 | 13.900 | 0.6681 | Yes |
| 21 | SMC1A |  |  | 1064 | 13.800 | 0.6887 | Yes |
| 22 | RUVBL2 |  |  | 1208 | 13.000 | 0.7004 | Yes |
| 23 | MCM2 |  |  | 1281 | 12.700 | 0.7162 | Yes |
| 24 | RANBP9 |  |  | 1481 | 11.700 | 0.7223 | Yes |
| 25 | MCM5 |  |  | 1526 | 11.500 | 0.7380 | Yes |
| 26 | MSH2 |  |  | 1546 | 11.500 | 0.7552 | Yes |
| 27 | EFTUD2 |  |  | 1596 | 11.300 | 0.7703 | Yes |
| 28 | TRIM28 |  |  | 1872 | 10.200 | 0.7691 | Yes |
| 29 | LMNB1 |  |  | 1916 | 10.000 | 0.7824 | Yes |
| 30 | GCN1L1 |  |  | 1945 | 9.900 | 0.7965 | Yes |
| 31 | SNRPB |  |  | 2049 | 9.500 | 0.8052 | Yes |
| 32 | POLR2A |  |  | 2058 | 9.500 | 0.8200 | Yes |
| 33 | SNRPD3 |  |  | 2255 | 8.900 | 0.8218 | Yes |
| 34 | C1QBP |  |  | 2623 | 7.900 | 0.8111 | Yes |
| 35 | GEMIN5 |  |  | 2648 | 7.800 | 0.8221 | Yes |
| 36 | CHD6 |  |  | 4788 | 3.400 | 0.6914 | No |
| 37 | DDX17 |  |  | 5551 | 2.200 | 0.6464 | No |
| 38 | PRKDC |  |  | 5996 | 1.600 | 0.6207 | No |
| 39 | HIST1H2AC |  |  | 7569 | 0.000 | 0.5207 | No |
Table: GSEA details [plain text format]

  

Fig 2: ABRAMSON\_INTERACT\_WITH\_AIRE: Random ES distribution      
 Gene set null distribution of ES for **ABRAMSON\_INTERACT\_WITH\_AIRE**

  
