# Supplementary material for: Immunomic, genomic and transcriptomic characterization of CT26 colorectal carcinoma
Source: BMC Genomics. 2014 Mar 13;15(1):190. doi: 10.1186/1471-2164-15-190 (PMC4007559; doi:10.1186/1471-2164-15-190)
Supplement: Supplementary file 8 — Additional file 8: Contains the Gene Pattern gene set membership and enrichment values in an html format. The file index.html is the entry point. (ZIP 13 MB) [file 12864_2013_7028_MOESM8_ESM.zip › BILANGES_SERUM_AND_RAPAMYCIN_SENSITIVE_GENES.html]

Details for gene set BILANGES\_SERUM\_AND\_RAPAMYCIN\_SENSITIVE\_GENES[GSEA]

|  || Dataset | CT26\_gene\_expression |
| Phenotype | NoPhenotypeAvailable |
| Upregulated in class | na\_pos |
| GeneSet | BILANGES\_SERUM\_AND\_RAPAMYCIN\_SENSITIVE\_GENES |
| Enrichment Score (ES) | 0.782523 |
| Normalized Enrichment Score (NES) | 1.6851931 |
| Nominal p-value | 0.0 |
| FDR q-value | 0.001657332 |
| FWER p-Value | 0.034 |
Table: GSEA Results Summary

  

Fig 1: Enrichment plot: BILANGES\_SERUM\_AND\_RAPAMYCIN\_SENSITIVE\_GENES      
 Profile of the Running ES Score & Positions of GeneSet Members on the Rank Ordered List

  

| PROBE | GENE SYMBOL | GENE\_TITLE | RANK IN GENE LIST | RANK METRIC SCORE | RUNNING ES | CORE ENRICHMENT || 1 | RPL5 |  |  | 15 | 45.800 | 0.0595 | Yes |
| 2 | EEF1G |  |  | 17 | 44.800 | 0.1186 | Yes |
| 3 | RPL27A |  |  | 32 | 38.700 | 0.1688 | Yes |
| 4 | RPL6 |  |  | 44 | 36.600 | 0.2164 | Yes |
| 5 | RPL26 |  |  | 67 | 33.300 | 0.2590 | Yes |
| 6 | RPS6 |  |  | 170 | 26.300 | 0.2872 | Yes |
| 7 | RPS8 |  |  | 172 | 26.100 | 0.3216 | Yes |
| 8 | RPL24 |  |  | 176 | 25.700 | 0.3553 | Yes |
| 9 | RPL14 |  |  | 236 | 23.500 | 0.3826 | Yes |
| 10 | RPS9 |  |  | 249 | 23.000 | 0.4122 | Yes |
| 11 | RPL30 |  |  | 319 | 21.300 | 0.4359 | Yes |
| 12 | EEF1B2 |  |  | 392 | 20.100 | 0.4578 | Yes |
| 13 | EEF1A1 |  |  | 419 | 19.600 | 0.4821 | Yes |
| 14 | COX7A2L |  |  | 534 | 18.100 | 0.4987 | Yes |
| 15 | RPS27A |  |  | 668 | 16.700 | 0.5123 | Yes |
| 16 | RPL7A |  |  | 755 | 15.900 | 0.5278 | Yes |
| 17 | RPS25 |  |  | 971 | 14.400 | 0.5331 | Yes |
| 18 | RPS7 |  |  | 1045 | 13.900 | 0.5468 | Yes |
| 19 | RPL4 |  |  | 1061 | 13.800 | 0.5640 | Yes |
| 20 | RPL15 |  |  | 1074 | 13.800 | 0.5815 | Yes |
| 21 | RPS3 |  |  | 1091 | 13.700 | 0.5986 | Yes |
| 22 | RPL23 |  |  | 1104 | 13.600 | 0.6157 | Yes |
| 23 | RPL28 |  |  | 1206 | 13.000 | 0.6265 | Yes |
| 24 | RPS3A |  |  | 1238 | 12.900 | 0.6415 | Yes |
| 25 | RPS4X |  |  | 1292 | 12.600 | 0.6548 | Yes |
| 26 | EIF4B |  |  | 1301 | 12.600 | 0.6709 | Yes |
| 27 | RPS24 |  |  | 1329 | 12.400 | 0.6856 | Yes |
| 28 | RPL8 |  |  | 1335 | 12.400 | 0.7016 | Yes |
| 29 | RPS17 |  |  | 1440 | 11.900 | 0.7107 | Yes |
| 30 | RPS10 |  |  | 1444 | 11.900 | 0.7262 | Yes |
| 31 | EEF2 |  |  | 1656 | 11.000 | 0.7273 | Yes |
| 32 | RBBP6 |  |  | 1681 | 10.900 | 0.7401 | Yes |
| 33 | RPL10 |  |  | 1749 | 10.600 | 0.7499 | Yes |
| 34 | RPL18 |  |  | 1991 | 9.700 | 0.7473 | Yes |
| 35 | RPS19 |  |  | 2039 | 9.500 | 0.7569 | Yes |
| 36 | RPL32 |  |  | 2062 | 9.500 | 0.7680 | Yes |
| 37 | FAU |  |  | 2161 | 9.100 | 0.7738 | Yes |
| 38 | RPL13A |  |  | 2211 | 9.000 | 0.7825 | Yes |
| 39 | RPS13 |  |  | 2425 | 8.400 | 0.7800 | No |
| 40 | RPL13 |  |  | 2604 | 7.900 | 0.7791 | No |
| 41 | RPS16 |  |  | 2958 | 7.000 | 0.7659 | No |
| 42 | RPS5 |  |  | 3104 | 6.600 | 0.7653 | No |
| 43 | GLTSCR2 |  |  | 3366 | 6.100 | 0.7568 | No |
| 44 | TPT1 |  |  | 4410 | 4.000 | 0.6956 | No |
| 45 | LTA4H |  |  | 4714 | 3.500 | 0.6809 | No |
| 46 | RPL18A |  |  | 4799 | 3.400 | 0.6800 | No |
| 47 | QARS |  |  | 5387 | 2.400 | 0.6458 | No |
| 48 | UBA52 |  |  | 5629 | 2.100 | 0.6332 | No |
| 49 | NBEA |  |  | 5791 | 1.900 | 0.6255 | No |
| 50 | RPS14 |  |  | 5940 | 1.700 | 0.6183 | No |
| 51 | SPRY2 |  |  | 6236 | 1.300 | 0.6012 | No |
| 52 | GNB2L1 |  |  | 6384 | 1.100 | 0.5933 | No |
| 53 | CECR6 |  |  | 10030 | 0.000 | 0.3610 | No |
| 54 | KCNK13 |  |  | 10900 | -0.200 | 0.3059 | No |
| 55 | PTMS |  |  | 11122 | -0.200 | 0.2921 | No |
| 56 | C8G |  |  | 11815 | -0.500 | 0.2486 | No |
| 57 | SIN3B |  |  | 13083 | -1.500 | 0.1699 | No |
Table: GSEA details [plain text format]

  

Fig 2: BILANGES\_SERUM\_AND\_RAPAMYCIN\_SENSITIVE\_GENES: Random ES distribution      
 Gene set null distribution of ES for **BILANGES\_SERUM\_AND\_RAPAMYCIN\_SENSITIVE\_GENES**

  
