# Supplementary material for: Immunomic, genomic and transcriptomic characterization of CT26 colorectal carcinoma
Source: BMC Genomics. 2014 Mar 13;15(1):190. doi: 10.1186/1471-2164-15-190 (PMC4007559; doi:10.1186/1471-2164-15-190)
Supplement: Supplementary file 8 — Additional file 8: Contains the Gene Pattern gene set membership and enrichment values in an html format. The file index.html is the entry point. (ZIP 13 MB) [file 12864_2013_7028_MOESM8_ESM.zip › BURTON_ADIPOGENESIS_3.html]

Details for gene set BURTON\_ADIPOGENESIS\_3[GSEA]

|  || Dataset | CT26\_gene\_expression |
| Phenotype | NoPhenotypeAvailable |
| Upregulated in class | na\_pos |
| GeneSet | BURTON\_ADIPOGENESIS\_3 |
| Enrichment Score (ES) | 0.7735372 |
| Normalized Enrichment Score (NES) | 1.6850427 |
| Nominal p-value | 0.0 |
| FDR q-value | 0.0015744654 |
| FWER p-Value | 0.034 |
Table: GSEA Results Summary

  

Fig 1: Enrichment plot: BURTON\_ADIPOGENESIS\_3      
 Profile of the Running ES Score & Positions of GeneSet Members on the Rank Ordered List

  

| PROBE | GENE SYMBOL | GENE\_TITLE | RANK IN GENE LIST | RANK METRIC SCORE | RUNNING ES | CORE ENRICHMENT || 1 | TOP2A |  |  | 8 | 56.000 | 0.0423 | Yes |
| 2 | CKS1B |  |  | 48 | 35.600 | 0.0671 | Yes |
| 3 | KIF20A |  |  | 77 | 31.900 | 0.0897 | Yes |
| 4 | MCM4 |  |  | 81 | 31.500 | 0.1136 | Yes |
| 5 | PRIM1 |  |  | 116 | 29.000 | 0.1336 | Yes |
| 6 | RRM1 |  |  | 138 | 27.500 | 0.1533 | Yes |
| 7 | NASP |  |  | 161 | 26.700 | 0.1724 | Yes |
| 8 | PRC1 |  |  | 181 | 25.500 | 0.1907 | Yes |
| 9 | KIAA0101 |  |  | 186 | 25.100 | 0.2096 | Yes |
| 10 | KIF11 |  |  | 197 | 24.700 | 0.2279 | Yes |
| 11 | TFDP1 |  |  | 199 | 24.700 | 0.2467 | Yes |
| 12 | INCENP |  |  | 260 | 22.700 | 0.2602 | Yes |
| 13 | CDC20 |  |  | 284 | 22.200 | 0.2758 | Yes |
| 14 | EZH2 |  |  | 286 | 22.000 | 0.2925 | Yes |
| 15 | MCM7 |  |  | 290 | 21.900 | 0.3091 | Yes |
| 16 | MCM3 |  |  | 315 | 21.400 | 0.3239 | Yes |
| 17 | KPNA2 |  |  | 354 | 20.700 | 0.3374 | Yes |
| 18 | CDCA7 |  |  | 356 | 20.700 | 0.3531 | Yes |
| 19 | BUB1 |  |  | 360 | 20.600 | 0.3687 | Yes |
| 20 | SMC2 |  |  | 379 | 20.300 | 0.3831 | Yes |
| 21 | PCNA |  |  | 386 | 20.200 | 0.3982 | Yes |
| 22 | ILF2 |  |  | 388 | 20.100 | 0.4135 | Yes |
| 23 | TOPBP1 |  |  | 401 | 19.900 | 0.4279 | Yes |
| 24 | MKI67 |  |  | 414 | 19.700 | 0.4422 | Yes |
| 25 | RAD51 |  |  | 427 | 19.400 | 0.4563 | Yes |
| 26 | NCAPH |  |  | 459 | 19.000 | 0.4689 | Yes |
| 27 | KIF22 |  |  | 461 | 18.900 | 0.4833 | Yes |
| 28 | BZW1 |  |  | 462 | 18.900 | 0.4977 | Yes |
| 29 | BIRC5 |  |  | 507 | 18.400 | 0.5090 | Yes |
| 30 | DNMT1 |  |  | 555 | 17.900 | 0.5197 | Yes |
| 31 | MAD2L1 |  |  | 559 | 17.900 | 0.5332 | Yes |
| 32 | KIF4A |  |  | 648 | 16.900 | 0.5405 | Yes |
| 33 | RACGAP1 |  |  | 682 | 16.600 | 0.5511 | Yes |
| 34 | HELLS |  |  | 705 | 16.400 | 0.5622 | Yes |
| 35 | NUSAP1 |  |  | 716 | 16.200 | 0.5740 | Yes |
| 36 | CSTF2 |  |  | 722 | 16.200 | 0.5861 | Yes |
| 37 | CCNE2 |  |  | 743 | 16.000 | 0.5970 | Yes |
| 38 | AURKA |  |  | 799 | 15.600 | 0.6055 | Yes |
| 39 | IMPDH2 |  |  | 826 | 15.400 | 0.6156 | Yes |
| 40 | TACC3 |  |  | 910 | 14.800 | 0.6216 | Yes |
| 41 | DTL |  |  | 936 | 14.700 | 0.6313 | Yes |
| 42 | CDCA8 |  |  | 1017 | 14.100 | 0.6369 | Yes |
| 43 | KIF2C |  |  | 1036 | 14.000 | 0.6465 | Yes |
| 44 | FEN1 |  |  | 1052 | 13.900 | 0.6562 | Yes |
| 45 | RRM2 |  |  | 1084 | 13.700 | 0.6647 | Yes |
| 46 | CCNF |  |  | 1108 | 13.500 | 0.6735 | Yes |
| 47 | CCNB2 |  |  | 1155 | 13.200 | 0.6807 | Yes |
| 48 | TCF19 |  |  | 1167 | 13.200 | 0.6901 | Yes |
| 49 | TRIP13 |  |  | 1182 | 13.100 | 0.6992 | Yes |
| 50 | POLA1 |  |  | 1250 | 12.800 | 0.7048 | Yes |
| 51 | MCM10 |  |  | 1264 | 12.700 | 0.7136 | Yes |
| 52 | HMGB2 |  |  | 1279 | 12.700 | 0.7225 | Yes |
| 53 | MCM2 |  |  | 1281 | 12.700 | 0.7321 | Yes |
| 54 | ASF1B |  |  | 1372 | 12.200 | 0.7357 | Yes |
| 55 | TTK |  |  | 1430 | 11.900 | 0.7412 | Yes |
| 56 | MCM5 |  |  | 1526 | 11.500 | 0.7439 | Yes |
| 57 | LMO4 |  |  | 1537 | 11.500 | 0.7521 | Yes |
| 58 | DCK |  |  | 1603 | 11.200 | 0.7565 | Yes |
| 59 | USP1 |  |  | 1669 | 10.900 | 0.7607 | Yes |
| 60 | RFC5 |  |  | 1676 | 10.900 | 0.7686 | Yes |
| 61 | MAN2A1 |  |  | 1899 | 10.100 | 0.7622 | Yes |
| 62 | SLBP |  |  | 1951 | 9.900 | 0.7665 | Yes |
| 63 | CKS2 |  |  | 1959 | 9.800 | 0.7735 | Yes |
| 64 | CCNB1 |  |  | 2266 | 8.900 | 0.7608 | No |
| 65 | CDC7 |  |  | 2273 | 8.900 | 0.7672 | No |
| 66 | RAD51AP1 |  |  | 2634 | 7.800 | 0.7502 | No |
| 67 | TK1 |  |  | 2762 | 7.500 | 0.7478 | No |
| 68 | BRCA1 |  |  | 2844 | 7.300 | 0.7482 | No |
| 69 | FIGNL1 |  |  | 2852 | 7.300 | 0.7534 | No |
| 70 | ABCA1 |  |  | 2911 | 7.100 | 0.7551 | No |
| 71 | TYMS |  |  | 3182 | 6.500 | 0.7428 | No |
| 72 | CDC6 |  |  | 3705 | 5.300 | 0.7135 | No |
| 73 | CENPK |  |  | 4002 | 4.800 | 0.6983 | No |
| 74 | TUBB6 |  |  | 5588 | 2.200 | 0.5987 | No |
| 75 | PLK1 |  |  | 6602 | 0.800 | 0.5346 | No |
| 76 | HGF |  |  | 10039 | 0.000 | 0.3152 | No |
| 77 | PTGFR |  |  | 10339 | -0.100 | 0.2961 | No |
| 78 | MTM1 |  |  | 10623 | -0.100 | 0.2781 | No |
| 79 | OSMR |  |  | 10917 | -0.200 | 0.2596 | No |
| 80 | VCAN |  |  | 11334 | -0.300 | 0.2332 | No |
| 81 | ADAM8 |  |  | 11623 | -0.400 | 0.2151 | No |
| 82 | RNASEH2C |  |  | 12461 | -1.000 | 0.1624 | No |
| 83 | HIST1H2AB |  |  | 12489 | -1.000 | 0.1615 | No |
| 84 | NQO1 |  |  | 14021 | -2.800 | 0.0658 | No |
| 85 | LRP5 |  |  | 14102 | -2.900 | 0.0629 | No |
| 86 | WNT4 |  |  | 14418 | -3.700 | 0.0457 | No |
| 87 | IL13RA1 |  |  | 14451 | -3.700 | 0.0464 | No |
| 88 | NID1 |  |  | 14522 | -3.900 | 0.0450 | No |
| 89 | SLCO3A1 |  |  | 14571 | -4.000 | 0.0449 | No |
| 90 | KLF5 |  |  | 14651 | -4.200 | 0.0431 | No |
| 91 | AQP1 |  |  | 14897 | -4.800 | 0.0311 | No |
| 92 | CX3CL1 |  |  | 15197 | -6.200 | 0.0168 | No |
| 93 | GAS6 |  |  | 15277 | -6.600 | 0.0168 | No |
| 94 | CSRP2 |  |  | 15473 | -8.300 | 0.0107 | No |
| 95 | TNXB |  |  | 15533 | -9.000 | 0.0138 | No |
Table: GSEA details [plain text format]

  

Fig 2: BURTON\_ADIPOGENESIS\_3: Random ES distribution      
 Gene set null distribution of ES for **BURTON\_ADIPOGENESIS\_3**

  
