# Supplementary material for: Immunomic, genomic and transcriptomic characterization of CT26 colorectal carcinoma
Source: BMC Genomics. 2014 Mar 13;15(1):190. doi: 10.1186/1471-2164-15-190 (PMC4007559; doi:10.1186/1471-2164-15-190)
Supplement: Supplementary file 8 — Additional file 8: Contains the Gene Pattern gene set membership and enrichment values in an html format. The file index.html is the entry point. (ZIP 13 MB) [file 12864_2013_7028_MOESM8_ESM.zip › CHANG_CYCLING_GENES.html]

Details for gene set CHANG\_CYCLING\_GENES[GSEA]

|  || Dataset | CT26\_gene\_expression |
| Phenotype | NoPhenotypeAvailable |
| Upregulated in class | na\_pos |
| GeneSet | CHANG\_CYCLING\_GENES |
| Enrichment Score (ES) | 0.7649451 |
| Normalized Enrichment Score (NES) | 1.6919044 |
| Nominal p-value | 0.0 |
| FDR q-value | 0.0019279879 |
| FWER p-Value | 0.03 |
Table: GSEA Results Summary

  

Fig 1: Enrichment plot: CHANG\_CYCLING\_GENES      
 Profile of the Running ES Score & Positions of GeneSet Members on the Rank Ordered List

  

| PROBE | GENE SYMBOL | GENE\_TITLE | RANK IN GENE LIST | RANK METRIC SCORE | RUNNING ES | CORE ENRICHMENT || 1 | SMC4 |  |  | 1 | 76.300 | 0.0418 | Yes |
| 2 | TOP2A |  |  | 8 | 56.000 | 0.0721 | Yes |
| 3 | CKAP2 |  |  | 16 | 45.400 | 0.0965 | Yes |
| 4 | TPX2 |  |  | 39 | 37.700 | 0.1158 | Yes |
| 5 | NCAPD2 |  |  | 55 | 35.000 | 0.1340 | Yes |
| 6 | CASP3 |  |  | 74 | 32.100 | 0.1505 | Yes |
| 7 | MCM4 |  |  | 81 | 31.500 | 0.1674 | Yes |
| 8 | PRIM1 |  |  | 116 | 29.000 | 0.1811 | Yes |
| 9 | ANP32E |  |  | 135 | 27.700 | 0.1951 | Yes |
| 10 | RRM1 |  |  | 138 | 27.500 | 0.2101 | Yes |
| 11 | TUBB |  |  | 153 | 26.800 | 0.2239 | Yes |
| 12 | UHRF1 |  |  | 162 | 26.600 | 0.2380 | Yes |
| 13 | MCM6 |  |  | 163 | 26.600 | 0.2525 | Yes |
| 14 | LYAR |  |  | 171 | 26.200 | 0.2665 | Yes |
| 15 | UBE2S |  |  | 177 | 25.600 | 0.2802 | Yes |
| 16 | CCNA2 |  |  | 178 | 25.600 | 0.2942 | Yes |
| 17 | KIAA0101 |  |  | 186 | 25.100 | 0.3075 | Yes |
| 18 | TSN |  |  | 191 | 24.800 | 0.3209 | Yes |
| 19 | CKAP2L |  |  | 217 | 24.100 | 0.3325 | Yes |
| 20 | RANGAP1 |  |  | 220 | 24.000 | 0.3455 | Yes |
| 21 | PTTG1 |  |  | 223 | 23.900 | 0.3585 | Yes |
| 22 | DIAPH3 |  |  | 235 | 23.500 | 0.3707 | Yes |
| 23 | HMMR |  |  | 246 | 23.200 | 0.3828 | Yes |
| 24 | EZH2 |  |  | 286 | 22.000 | 0.3923 | Yes |
| 25 | FOXM1 |  |  | 322 | 21.200 | 0.4017 | Yes |
| 26 | KIF23 |  |  | 337 | 21.000 | 0.4123 | Yes |
| 27 | CDCA7 |  |  | 356 | 20.700 | 0.4225 | Yes |
| 28 | BUB1 |  |  | 360 | 20.600 | 0.4336 | Yes |
| 29 | PWP1 |  |  | 364 | 20.500 | 0.4447 | Yes |
| 30 | ANLN |  |  | 374 | 20.400 | 0.4553 | Yes |
| 31 | PCNA |  |  | 386 | 20.200 | 0.4657 | Yes |
| 32 | ILF2 |  |  | 388 | 20.100 | 0.4766 | Yes |
| 33 | TIMP1 |  |  | 430 | 19.400 | 0.4846 | Yes |
| 34 | CENPF |  |  | 431 | 19.400 | 0.4953 | Yes |
| 35 | DKC1 |  |  | 458 | 19.100 | 0.5041 | Yes |
| 36 | NCAPH |  |  | 459 | 19.000 | 0.5145 | Yes |
| 37 | KIF22 |  |  | 461 | 18.900 | 0.5248 | Yes |
| 38 | BIRC5 |  |  | 507 | 18.400 | 0.5320 | Yes |
| 39 | BUB3 |  |  | 533 | 18.100 | 0.5403 | Yes |
| 40 | MAD2L1 |  |  | 559 | 17.900 | 0.5486 | Yes |
| 41 | PRPS2 |  |  | 572 | 17.700 | 0.5575 | Yes |
| 42 | ATAD2 |  |  | 596 | 17.400 | 0.5656 | Yes |
| 43 | BUB1B |  |  | 609 | 17.300 | 0.5743 | Yes |
| 44 | CENPQ |  |  | 635 | 17.000 | 0.5820 | Yes |
| 45 | PRR11 |  |  | 636 | 17.000 | 0.5913 | Yes |
| 46 | HELLS |  |  | 705 | 16.400 | 0.5960 | Yes |
| 47 | RFC2 |  |  | 712 | 16.300 | 0.6045 | Yes |
| 48 | NUSAP1 |  |  | 716 | 16.200 | 0.6132 | Yes |
| 49 | AURKA |  |  | 799 | 15.600 | 0.6165 | Yes |
| 50 | FAM64A |  |  | 841 | 15.300 | 0.6223 | Yes |
| 51 | NUF2 |  |  | 866 | 15.100 | 0.6290 | Yes |
| 52 | KIFC1 |  |  | 870 | 15.100 | 0.6371 | Yes |
| 53 | PBK |  |  | 874 | 15.000 | 0.6451 | Yes |
| 54 | AMD1 |  |  | 876 | 15.000 | 0.6533 | Yes |
| 55 | TACC3 |  |  | 910 | 14.800 | 0.6593 | Yes |
| 56 | GMNN |  |  | 911 | 14.800 | 0.6674 | Yes |
| 57 | SPAG5 |  |  | 927 | 14.700 | 0.6745 | Yes |
| 58 | MET |  |  | 954 | 14.500 | 0.6808 | Yes |
| 59 | CDCA8 |  |  | 1017 | 14.100 | 0.6846 | Yes |
| 60 | FEN1 |  |  | 1052 | 13.900 | 0.6900 | Yes |
| 61 | CDC25C |  |  | 1075 | 13.700 | 0.6961 | Yes |
| 62 | MELK |  |  | 1077 | 13.700 | 0.7036 | Yes |
| 63 | RRM2 |  |  | 1084 | 13.700 | 0.7107 | Yes |
| 64 | TIPIN |  |  | 1097 | 13.600 | 0.7174 | Yes |
| 65 | CCNF |  |  | 1108 | 13.500 | 0.7241 | Yes |
| 66 | CCNB2 |  |  | 1155 | 13.200 | 0.7284 | Yes |
| 67 | E2F1 |  |  | 1172 | 13.100 | 0.7346 | Yes |
| 68 | TRIP13 |  |  | 1182 | 13.100 | 0.7412 | Yes |
| 69 | GTSE1 |  |  | 1272 | 12.700 | 0.7425 | Yes |
| 70 | ASF1B |  |  | 1372 | 12.200 | 0.7428 | Yes |
| 71 | WSB1 |  |  | 1419 | 12.000 | 0.7465 | Yes |
| 72 | MCM5 |  |  | 1526 | 11.500 | 0.7460 | Yes |
| 73 | UBE2T |  |  | 1606 | 11.200 | 0.7471 | Yes |
| 74 | USP1 |  |  | 1669 | 10.900 | 0.7491 | Yes |
| 75 | ESCO2 |  |  | 1671 | 10.900 | 0.7550 | Yes |
| 76 | CENPA |  |  | 1762 | 10.600 | 0.7550 | Yes |
| 77 | CDCA5 |  |  | 1813 | 10.400 | 0.7575 | Yes |
| 78 | PSRC1 |  |  | 1818 | 10.400 | 0.7630 | Yes |
| 79 | LMNB1 |  |  | 1916 | 10.000 | 0.7623 | Yes |
| 80 | CKS2 |  |  | 1959 | 9.800 | 0.7649 | Yes |
| 81 | RFC4 |  |  | 2243 | 8.900 | 0.7517 | No |
| 82 | GAS2L3 |  |  | 2291 | 8.800 | 0.7535 | No |
| 83 | DHFR |  |  | 2366 | 8.600 | 0.7535 | No |
| 84 | CENPM |  |  | 2477 | 8.300 | 0.7510 | No |
| 85 | CCDC99 |  |  | 2632 | 7.800 | 0.7454 | No |
| 86 | RAD51AP1 |  |  | 2634 | 7.800 | 0.7496 | No |
| 87 | SGCD |  |  | 2877 | 7.200 | 0.7381 | No |
| 88 | UBE2C |  |  | 2879 | 7.200 | 0.7420 | No |
| 89 | ADAMTS1 |  |  | 2882 | 7.200 | 0.7458 | No |
| 90 | CDKN3 |  |  | 2917 | 7.100 | 0.7475 | No |
| 91 | MLF1IP |  |  | 2974 | 7.000 | 0.7478 | No |
| 92 | GINS2 |  |  | 3094 | 6.700 | 0.7438 | No |
| 93 | GINS3 |  |  | 3482 | 5.800 | 0.7222 | No |
| 94 | MCM8 |  |  | 3515 | 5.700 | 0.7233 | No |
| 95 | CDC25A |  |  | 3621 | 5.500 | 0.7196 | No |
| 96 | DEPDC1B |  |  | 3633 | 5.500 | 0.7219 | No |
| 97 | RECQL4 |  |  | 3672 | 5.400 | 0.7225 | No |
| 98 | CDC6 |  |  | 3705 | 5.300 | 0.7233 | No |
| 99 | BARD1 |  |  | 3765 | 5.200 | 0.7224 | No |
| 100 | WDR76 |  |  | 4028 | 4.700 | 0.7082 | No |
| 101 | EXO1 |  |  | 4074 | 4.700 | 0.7079 | No |
| 102 | DONSON |  |  | 4580 | 3.800 | 0.6776 | No |
| 103 | PHTF2 |  |  | 4679 | 3.600 | 0.6733 | No |
| 104 | MND1 |  |  | 4800 | 3.400 | 0.6675 | No |
| 105 | DEPDC1 |  |  | 4915 | 3.200 | 0.6620 | No |
| 106 | CDKL5 |  |  | 5255 | 2.600 | 0.6417 | No |
| 107 | FAM83D |  |  | 5761 | 1.900 | 0.6104 | No |
| 108 | SLC30A7 |  |  | 5830 | 1.800 | 0.6071 | No |
| 109 | MNT |  |  | 6292 | 1.200 | 0.5782 | No |
| 110 | ARL4A |  |  | 6514 | 0.900 | 0.5646 | No |
| 111 | PLK1 |  |  | 6602 | 0.800 | 0.5594 | No |
| 112 | KIAA1370 |  |  | 7249 | 0.200 | 0.5182 | No |
| 113 | NLRP2 |  |  | 7434 | 0.100 | 0.5065 | No |
| 114 | HIST1H2AC |  |  | 7569 | 0.000 | 0.4979 | No |
| 115 | EFHC1 |  |  | 10846 | -0.200 | 0.2883 | No |
| 116 | IFIT1 |  |  | 11185 | -0.300 | 0.2668 | No |
| 117 | ANKRD10 |  |  | 12304 | -0.900 | 0.1957 | No |
| 118 | FBXL20 |  |  | 12618 | -1.100 | 0.1763 | No |
| 119 | MBOAT1 |  |  | 12796 | -1.200 | 0.1656 | No |
| 120 | FANCG |  |  | 12874 | -1.300 | 0.1614 | No |
| 121 | CIITA |  |  | 13301 | -1.800 | 0.1351 | No |
| 122 | ITGB3 |  |  | 13878 | -2.600 | 0.0997 | No |
| 123 | HN1 |  |  | 13980 | -2.700 | 0.0947 | No |
| 124 | H2AFX |  |  | 14159 | -3.000 | 0.0850 | No |
| 125 | MLLT6 |  |  | 14167 | -3.100 | 0.0862 | No |
| 126 | TUBA4A |  |  | 14611 | -4.100 | 0.0601 | No |
| 127 | SDC1 |  |  | 15474 | -8.300 | 0.0095 | No |
| 128 | MAPK13 |  |  | 15694 | -14.700 | 0.0035 | No |
Table: GSEA details [plain text format]

  

Fig 2: CHANG\_CYCLING\_GENES: Random ES distribution      
 Gene set null distribution of ES for **CHANG\_CYCLING\_GENES**

  
