# Supplementary material for: Immunomic, genomic and transcriptomic characterization of CT26 colorectal carcinoma
Source: BMC Genomics. 2014 Mar 13;15(1):190. doi: 10.1186/1471-2164-15-190 (PMC4007559; doi:10.1186/1471-2164-15-190)
Supplement: Supplementary file 8 — Additional file 8: Contains the Gene Pattern gene set membership and enrichment values in an html format. The file index.html is the entry point. (ZIP 13 MB) [file 12864_2013_7028_MOESM8_ESM.zip › CHARAFE_BREAST_CANCER_LUMINAL_VS_BASAL_UP.html]

Details for gene set CHARAFE\_BREAST\_CANCER\_LUMINAL\_VS\_BASAL\_UP[GSEA]

|  || Dataset | CT26\_gene\_expression |
| Phenotype | NoPhenotypeAvailable |
| Upregulated in class | na\_neg |
| GeneSet | CHARAFE\_BREAST\_CANCER\_LUMINAL\_VS\_BASAL\_UP |
| Enrichment Score (ES) | -0.29749802 |
| Normalized Enrichment Score (NES) | NaN |
| Nominal p-value | NaN |
| FDR q-value | 1.0 |
| FWER p-Value | 0.0 |
Table: GSEA Results Summary

  

Fig 1: Enrichment plot: CHARAFE\_BREAST\_CANCER\_LUMINAL\_VS\_BASAL\_UP      
 Profile of the Running ES Score & Positions of GeneSet Members on the Rank Ordered List

  

| PROBE | GENE SYMBOL | GENE\_TITLE | RANK IN GENE LIST | RANK METRIC SCORE | RUNNING ES | CORE ENRICHMENT || 1 | TOMM70A |  |  | 180 | 25.500 | 0.0067 | No |
| 2 | CCND1 |  |  | 274 | 22.500 | 0.0168 | No |
| 3 | MYEF2 |  |  | 466 | 18.800 | 0.0180 | No |
| 4 | TBL1X |  |  | 472 | 18.800 | 0.0312 | No |
| 5 | CACYBP |  |  | 563 | 17.800 | 0.0382 | No |
| 6 | CADM1 |  |  | 616 | 17.300 | 0.0473 | No |
| 7 | PCBP2 |  |  | 632 | 17.000 | 0.0585 | No |
| 8 | PLA2G12A |  |  | 703 | 16.400 | 0.0658 | No |
| 9 | GARS |  |  | 745 | 16.000 | 0.0746 | No |
| 10 | GART |  |  | 899 | 14.800 | 0.0754 | No |
| 11 | GSPT1 |  |  | 970 | 14.400 | 0.0812 | No |
| 12 | API5 |  |  | 999 | 14.200 | 0.0896 | No |
| 13 | DDX42 |  |  | 1016 | 14.100 | 0.0987 | No |
| 14 | USP7 |  |  | 1038 | 14.000 | 0.1074 | No |
| 15 | THUMPD1 |  |  | 1141 | 13.300 | 0.1104 | No |
| 16 | REEP5 |  |  | 1142 | 13.300 | 0.1199 | No |
| 17 | MARS |  |  | 1244 | 12.800 | 0.1226 | No |
| 18 | CRNKL1 |  |  | 1275 | 12.700 | 0.1298 | No |
| 19 | FUS |  |  | 1345 | 12.300 | 0.1342 | No |
| 20 | TAPT1 |  |  | 1411 | 12.000 | 0.1386 | No |
| 21 | ZNF24 |  |  | 1435 | 11.900 | 0.1457 | No |
| 22 | TTC3 |  |  | 1663 | 11.000 | 0.1389 | No |
| 23 | PCK2 |  |  | 1665 | 11.000 | 0.1467 | No |
| 24 | KIAA0232 |  |  | 1797 | 10.400 | 0.1457 | No |
| 25 | SLC1A4 |  |  | 1867 | 10.200 | 0.1486 | No |
| 26 | NACA |  |  | 1893 | 10.100 | 0.1542 | No |
| 27 | TNIP1 |  |  | 2051 | 9.500 | 0.1509 | No |
| 28 | SDCCAG3 |  |  | 2231 | 9.000 | 0.1458 | No |
| 29 | POLE |  |  | 2239 | 8.900 | 0.1517 | No |
| 30 | TMEM57 |  |  | 2258 | 8.900 | 0.1570 | No |
| 31 | EMP2 |  |  | 2401 | 8.500 | 0.1539 | No |
| 32 | INTS3 |  |  | 2403 | 8.500 | 0.1599 | No |
| 33 | FRS2 |  |  | 2537 | 8.100 | 0.1572 | No |
| 34 | ICA1 |  |  | 2539 | 8.100 | 0.1629 | No |
| 35 | ASH1L |  |  | 2772 | 7.500 | 0.1533 | No |
| 36 | LARGE |  |  | 2773 | 7.500 | 0.1587 | No |
| 37 | UAP1L1 |  |  | 2884 | 7.200 | 0.1568 | No |
| 38 | SNX27 |  |  | 2907 | 7.100 | 0.1604 | No |
| 39 | SMARCC2 |  |  | 2940 | 7.000 | 0.1634 | No |
| 40 | FRMD4A |  |  | 2961 | 7.000 | 0.1671 | No |
| 41 | EVL |  |  | 3073 | 6.700 | 0.1648 | No |
| 42 | ARF3 |  |  | 3083 | 6.700 | 0.1690 | No |
| 43 | GNA12 |  |  | 3084 | 6.700 | 0.1738 | No |
| 44 | PGGT1B |  |  | 3168 | 6.500 | 0.1731 | No |
| 45 | BPTF |  |  | 3210 | 6.400 | 0.1751 | No |
| 46 | NUCB2 |  |  | 3300 | 6.200 | 0.1738 | No |
| 47 | RND1 |  |  | 3335 | 6.100 | 0.1760 | No |
| 48 | MDM4 |  |  | 3390 | 6.000 | 0.1768 | No |
| 49 | STRBP |  |  | 3516 | 5.700 | 0.1728 | No |
| 50 | ARID2 |  |  | 3618 | 5.500 | 0.1702 | No |
| 51 | ABHD12 |  |  | 3640 | 5.500 | 0.1728 | No |
| 52 | PLXNA3 |  |  | 3716 | 5.300 | 0.1718 | No |
| 53 | CSNK1D |  |  | 3756 | 5.300 | 0.1730 | No |
| 54 | USP42 |  |  | 3829 | 5.100 | 0.1721 | No |
| 55 | PBX1 |  |  | 3887 | 5.000 | 0.1720 | No |
| 56 | FKBP4 |  |  | 3912 | 4.900 | 0.1739 | No |
| 57 | ESR1 |  |  | 3916 | 4.900 | 0.1773 | No |
| 58 | SFI1 |  |  | 3976 | 4.800 | 0.1769 | No |
| 59 | BLNK |  |  | 4021 | 4.700 | 0.1774 | No |
| 60 | MYO5B |  |  | 4092 | 4.600 | 0.1762 | No |
| 61 | ZNF12 |  |  | 4150 | 4.500 | 0.1757 | No |
| 62 | PATZ1 |  |  | 4154 | 4.500 | 0.1788 | No |
| 63 | IQCE |  |  | 4303 | 4.200 | 0.1722 | No |
| 64 | POGZ |  |  | 4349 | 4.100 | 0.1723 | No |
| 65 | TTC9 |  |  | 4452 | 4.000 | 0.1685 | No |
| 66 | SERF2 |  |  | 4500 | 3.900 | 0.1683 | No |
| 67 | GTF3C1 |  |  | 4531 | 3.800 | 0.1691 | No |
| 68 | KLHL22 |  |  | 4550 | 3.800 | 0.1707 | No |
| 69 | CCDC117 |  |  | 4556 | 3.800 | 0.1731 | No |
| 70 | ZFYVE16 |  |  | 4570 | 3.800 | 0.1750 | No |
| 71 | CDC42SE1 |  |  | 4620 | 3.700 | 0.1744 | No |
| 72 | HK2 |  |  | 4656 | 3.600 | 0.1748 | No |
| 73 | SCYL3 |  |  | 4717 | 3.500 | 0.1734 | No |
| 74 | MAPK9 |  |  | 4725 | 3.500 | 0.1755 | No |
| 75 | RBAK |  |  | 4845 | 3.300 | 0.1701 | No |
| 76 | AR |  |  | 4866 | 3.300 | 0.1712 | No |
| 77 | TRPS1 |  |  | 4902 | 3.200 | 0.1713 | No |
| 78 | RDH13 |  |  | 5012 | 3.000 | 0.1664 | No |
| 79 | UBN1 |  |  | 5082 | 2.900 | 0.1640 | No |
| 80 | MTERFD3 |  |  | 5107 | 2.900 | 0.1645 | No |
| 81 | GARNL3 |  |  | 5128 | 2.800 | 0.1652 | No |
| 82 | POMT1 |  |  | 5214 | 2.700 | 0.1617 | No |
| 83 | DHRS13 |  |  | 5258 | 2.600 | 0.1607 | No |
| 84 | CEP350 |  |  | 5297 | 2.600 | 0.1602 | No |
| 85 | BCOR |  |  | 5312 | 2.600 | 0.1611 | No |
| 86 | NUDT4 |  |  | 5341 | 2.500 | 0.1611 | No |
| 87 | DNAJC1 |  |  | 5344 | 2.500 | 0.1628 | No |
| 88 | MCCC2 |  |  | 5422 | 2.400 | 0.1595 | No |
| 89 | ASB8 |  |  | 5457 | 2.300 | 0.1590 | No |
| 90 | BAZ2A |  |  | 5463 | 2.300 | 0.1603 | No |
| 91 | ANXA9 |  |  | 5477 | 2.300 | 0.1611 | No |
| 92 | RAB11FIP3 |  |  | 5500 | 2.300 | 0.1613 | No |
| 93 | DLG3 |  |  | 5508 | 2.300 | 0.1625 | No |
| 94 | DIP2C |  |  | 5515 | 2.300 | 0.1638 | No |
| 95 | NLK |  |  | 5545 | 2.200 | 0.1635 | No |
| 96 | SRRM2 |  |  | 5605 | 2.200 | 0.1613 | No |
| 97 | ATP6AP1 |  |  | 5625 | 2.100 | 0.1616 | No |
| 98 | DAAM1 |  |  | 5663 | 2.100 | 0.1607 | No |
| 99 | NPDC1 |  |  | 5676 | 2.100 | 0.1614 | No |
| 100 | KIAA0226 |  |  | 5710 | 2.000 | 0.1607 | No |
| 101 | TNRC18 |  |  | 5734 | 2.000 | 0.1607 | No |
| 102 | THSD4 |  |  | 5785 | 1.900 | 0.1588 | No |
| 103 | KLRG2 |  |  | 5864 | 1.800 | 0.1550 | No |
| 104 | SPTLC2 |  |  | 5941 | 1.700 | 0.1513 | No |
| 105 | KIAA1467 |  |  | 5946 | 1.700 | 0.1523 | No |
| 106 | KIAA0913 |  |  | 6005 | 1.600 | 0.1497 | No |
| 107 | ABCA3 |  |  | 6029 | 1.600 | 0.1494 | No |
| 108 | GGA3 |  |  | 6076 | 1.500 | 0.1475 | No |
| 109 | DENND4B |  |  | 6146 | 1.400 | 0.1440 | No |
| 110 | SLC16A6 |  |  | 6365 | 1.100 | 0.1307 | No |
| 111 | TGFB3 |  |  | 6394 | 1.100 | 0.1297 | No |
| 112 | KIAA0556 |  |  | 6408 | 1.000 | 0.1295 | No |
| 113 | GAMT |  |  | 6530 | 0.900 | 0.1223 | No |
| 114 | SECISBP2 |  |  | 6721 | 0.700 | 0.1105 | No |
| 115 | IQSEC1 |  |  | 6743 | 0.700 | 0.1097 | No |
| 116 | MEGF9 |  |  | 6807 | 0.600 | 0.1060 | No |
| 117 | ERGIC1 |  |  | 6809 | 0.600 | 0.1064 | No |
| 118 | ULK1 |  |  | 6908 | 0.500 | 0.1004 | No |
| 119 | TRIM3 |  |  | 6920 | 0.500 | 0.1001 | No |
| 120 | SLC25A44 |  |  | 6948 | 0.400 | 0.0986 | No |
| 121 | SFMBT2 |  |  | 6975 | 0.400 | 0.0972 | No |
| 122 | DENND1A |  |  | 6987 | 0.400 | 0.0968 | No |
| 123 | ZNF703 |  |  | 7022 | 0.400 | 0.0949 | No |
| 124 | DNALI1 |  |  | 7067 | 0.300 | 0.0922 | No |
| 125 | RALGPS1 |  |  | 7130 | 0.300 | 0.0885 | No |
| 126 | HPX |  |  | 7254 | 0.200 | 0.0806 | No |
| 127 | SNED1 |  |  | 7327 | 0.100 | 0.0760 | No |
| 128 | ARFIP2 |  |  | 7545 | 0.000 | 0.0620 | No |
| 129 | TGIF2 |  |  | 7561 | 0.000 | 0.0610 | No |
| 130 | VPS72 |  |  | 7631 | 0.000 | 0.0566 | No |
| 131 | ONECUT2 |  |  | 7644 | 0.000 | 0.0558 | No |
| 132 | JHDM1D |  |  | 7829 | 0.000 | 0.0439 | No |
| 133 | SYCP2 |  |  | 7879 | 0.000 | 0.0407 | No |
| 134 | ELOVL2 |  |  | 9163 | 0.000 | -0.0424 | No |
| 135 | PGR |  |  | 9371 | 0.000 | -0.0558 | No |
| 136 | TFF1 |  |  | 9404 | 0.000 | -0.0578 | No |
| 137 | PRRT3 |  |  | 9734 | 0.000 | -0.0791 | No |
| 138 | ZNF704 |  |  | 9795 | 0.000 | -0.0830 | No |
| 139 | SLC35A1 |  |  | 10016 | 0.000 | -0.0973 | No |
| 140 | ZNF398 |  |  | 10202 | -0.100 | -0.1092 | No |
| 141 | CLSTN2 |  |  | 10211 | -0.100 | -0.1096 | No |
| 142 | PRLR |  |  | 10318 | -0.100 | -0.1164 | No |
| 143 | F7 |  |  | 10359 | -0.100 | -0.1189 | No |
| 144 | LONRF2 |  |  | 10502 | -0.100 | -0.1280 | No |
| 145 | LRRN1 |  |  | 10511 | -0.100 | -0.1285 | No |
| 146 | GATA3 |  |  | 10535 | -0.100 | -0.1299 | No |
| 147 | SLC4A8 |  |  | 10600 | -0.100 | -0.1340 | No |
| 148 | CACNA1D |  |  | 10636 | -0.100 | -0.1362 | No |
| 149 | CTNND2 |  |  | 10706 | -0.100 | -0.1406 | No |
| 150 | CACNA2D2 |  |  | 10792 | -0.100 | -0.1460 | No |
| 151 | FAM110B |  |  | 10827 | -0.200 | -0.1480 | No |
| 152 | ETNK2 |  |  | 10883 | -0.200 | -0.1515 | No |
| 153 | ASTN2 |  |  | 10926 | -0.200 | -0.1540 | No |
| 154 | SLC38A1 |  |  | 10929 | -0.200 | -0.1540 | No |
| 155 | RHOH |  |  | 10973 | -0.200 | -0.1567 | No |
| 156 | WFS1 |  |  | 11038 | -0.200 | -0.1607 | No |
| 157 | RHPN1 |  |  | 11044 | -0.200 | -0.1608 | No |
| 158 | KIAA1598 |  |  | 11315 | -0.300 | -0.1781 | No |
| 159 | INHBB |  |  | 11390 | -0.300 | -0.1827 | No |
| 160 | MAPT |  |  | 11394 | -0.300 | -0.1827 | No |
| 161 | TMEM80 |  |  | 11424 | -0.300 | -0.1843 | No |
| 162 | SCUBE2 |  |  | 11553 | -0.400 | -0.1923 | No |
| 163 | FLJ22184 |  |  | 11571 | -0.400 | -0.1931 | No |
| 164 | DACH1 |  |  | 11584 | -0.400 | -0.1936 | No |
| 165 | PYCR1 |  |  | 11772 | -0.500 | -0.2054 | No |
| 166 | CACNG4 |  |  | 11818 | -0.500 | -0.2079 | No |
| 167 | MLL2 |  |  | 11842 | -0.600 | -0.2090 | No |
| 168 | PCP4 |  |  | 11857 | -0.600 | -0.2095 | No |
| 169 | AFF3 |  |  | 11862 | -0.600 | -0.2093 | No |
| 170 | PPP2R2C |  |  | 12035 | -0.700 | -0.2199 | No |
| 171 | ABCG1 |  |  | 12119 | -0.700 | -0.2248 | No |
| 172 | SLC7A8 |  |  | 12136 | -0.700 | -0.2253 | No |
| 173 | LMCD1 |  |  | 12191 | -0.800 | -0.2282 | No |
| 174 | DUSP8 |  |  | 12265 | -0.800 | -0.2324 | No |
| 175 | LNX1 |  |  | 12302 | -0.900 | -0.2341 | No |
| 176 | RNF103 |  |  | 12333 | -0.900 | -0.2354 | No |
| 177 | ANKRD13D |  |  | 12348 | -0.900 | -0.2356 | No |
| 178 | GPRC5C |  |  | 12460 | -1.000 | -0.2421 | No |
| 179 | IVD |  |  | 12491 | -1.000 | -0.2433 | No |
| 180 | CHN2 |  |  | 12584 | -1.000 | -0.2486 | No |
| 181 | BAI2 |  |  | 12620 | -1.100 | -0.2500 | No |
| 182 | ATP6V0E2 |  |  | 12702 | -1.100 | -0.2545 | No |
| 183 | PREX1 |  |  | 12737 | -1.200 | -0.2558 | No |
| 184 | MIF4GD |  |  | 12751 | -1.200 | -0.2558 | No |
| 185 | EPS8L1 |  |  | 12778 | -1.200 | -0.2566 | No |
| 186 | SHANK2 |  |  | 12864 | -1.300 | -0.2612 | No |
| 187 | RSAD1 |  |  | 12895 | -1.300 | -0.2622 | No |
| 188 | KLF2 |  |  | 12923 | -1.400 | -0.2629 | No |
| 189 | MYCN |  |  | 12938 | -1.400 | -0.2628 | No |
| 190 | ZNF444 |  |  | 12968 | -1.400 | -0.2637 | No |
| 191 | HEXDC |  |  | 12969 | -1.400 | -0.2627 | No |
| 192 | DNAJA4 |  |  | 12970 | -1.400 | -0.2617 | No |
| 193 | PRR14 |  |  | 12990 | -1.400 | -0.2619 | No |
| 194 | CYHR1 |  |  | 12998 | -1.400 | -0.2614 | No |
| 195 | ZMIZ1 |  |  | 13010 | -1.500 | -0.2610 | No |
| 196 | SYNGR2 |  |  | 13026 | -1.500 | -0.2609 | No |
| 197 | ENPP1 |  |  | 13035 | -1.500 | -0.2603 | No |
| 198 | SOX12 |  |  | 13043 | -1.500 | -0.2597 | No |
| 199 | IRGQ |  |  | 13048 | -1.500 | -0.2589 | No |
| 200 | MYB |  |  | 13065 | -1.500 | -0.2588 | No |
| 201 | FZD4 |  |  | 13203 | -1.700 | -0.2665 | No |
| 202 | SLC26A11 |  |  | 13307 | -1.800 | -0.2719 | No |
| 203 | RAB17 |  |  | 13310 | -1.800 | -0.2707 | No |
| 204 | TOB1 |  |  | 13383 | -1.900 | -0.2740 | No |
| 205 | DOPEY2 |  |  | 13485 | -2.000 | -0.2791 | No |
| 206 | ARRB1 |  |  | 13572 | -2.100 | -0.2831 | No |
| 207 | XBP1 |  |  | 13685 | -2.300 | -0.2887 | No |
| 208 | KIAA1244 |  |  | 13722 | -2.400 | -0.2893 | No |
| 209 | KCTD15 |  |  | 13740 | -2.400 | -0.2887 | No |
| 210 | RHBDF1 |  |  | 13767 | -2.400 | -0.2887 | No |
| 211 | TBX3 |  |  | 13785 | -2.400 | -0.2880 | No |
| 212 | CA12 |  |  | 13912 | -2.700 | -0.2943 | No |
| 213 | ISG20 |  |  | 13963 | -2.700 | -0.2956 | Yes |
| 214 | RGL2 |  |  | 13985 | -2.800 | -0.2949 | Yes |
| 215 | CFD |  |  | 13989 | -2.800 | -0.2931 | Yes |
| 216 | USP3 |  |  | 13994 | -2.800 | -0.2913 | Yes |
| 217 | RABEP2 |  |  | 14003 | -2.800 | -0.2898 | Yes |
| 218 | GPD1L |  |  | 14023 | -2.800 | -0.2890 | Yes |
| 219 | FAM46C |  |  | 14047 | -2.900 | -0.2885 | Yes |
| 220 | PRRT2 |  |  | 14115 | -3.000 | -0.2906 | Yes |
| 221 | LZTR1 |  |  | 14185 | -3.100 | -0.2929 | Yes |
| 222 | MYO6 |  |  | 14195 | -3.100 | -0.2912 | Yes |
| 223 | CTXN1 |  |  | 14196 | -3.100 | -0.2890 | Yes |
| 224 | GPR160 |  |  | 14205 | -3.100 | -0.2873 | Yes |
| 225 | ZNF467 |  |  | 14227 | -3.200 | -0.2863 | Yes |
| 226 | VPS37C |  |  | 14240 | -3.200 | -0.2848 | Yes |
| 227 | TESK1 |  |  | 14242 | -3.200 | -0.2826 | Yes |
| 228 | GGA1 |  |  | 14305 | -3.300 | -0.2842 | Yes |
| 229 | RAB40C |  |  | 14306 | -3.300 | -0.2818 | Yes |
| 230 | CACNB3 |  |  | 14311 | -3.400 | -0.2797 | Yes |
| 231 | LRP3 |  |  | 14314 | -3.400 | -0.2773 | Yes |
| 232 | GOLT1A |  |  | 14315 | -3.400 | -0.2749 | Yes |
| 233 | SIDT2 |  |  | 14396 | -3.600 | -0.2775 | Yes |
| 234 | TBC1D16 |  |  | 14408 | -3.600 | -0.2756 | Yes |
| 235 | SBK1 |  |  | 14466 | -3.800 | -0.2766 | Yes |
| 236 | SPATA2L |  |  | 14514 | -3.900 | -0.2768 | Yes |
| 237 | CREB3L1 |  |  | 14535 | -3.900 | -0.2753 | Yes |
| 238 | RUSC1 |  |  | 14540 | -3.900 | -0.2727 | Yes |
| 239 | SLC24A3 |  |  | 14544 | -3.900 | -0.2701 | Yes |
| 240 | SLC25A29 |  |  | 14549 | -3.900 | -0.2676 | Yes |
| 241 | CYB561 |  |  | 14574 | -4.000 | -0.2663 | Yes |
| 242 | FGFR4 |  |  | 14593 | -4.100 | -0.2645 | Yes |
| 243 | TLE3 |  |  | 14599 | -4.100 | -0.2619 | Yes |
| 244 | HIP1R |  |  | 14605 | -4.100 | -0.2592 | Yes |
| 245 | PLCXD1 |  |  | 14668 | -4.200 | -0.2602 | Yes |
| 246 | CIRBP |  |  | 14671 | -4.300 | -0.2573 | Yes |
| 247 | ABHD11 |  |  | 14728 | -4.400 | -0.2577 | Yes |
| 248 | NDUFS8 |  |  | 14757 | -4.500 | -0.2563 | Yes |
| 249 | MXRA8 |  |  | 14759 | -4.500 | -0.2531 | Yes |
| 250 | FOXA1 |  |  | 14800 | -4.600 | -0.2524 | Yes |
| 251 | MAGED2 |  |  | 14812 | -4.600 | -0.2498 | Yes |
| 252 | ADCY6 |  |  | 14830 | -4.700 | -0.2475 | Yes |
| 253 | HMG20B |  |  | 14832 | -4.700 | -0.2442 | Yes |
| 254 | KIAA0182 |  |  | 14869 | -4.800 | -0.2431 | Yes |
| 255 | CXXC5 |  |  | 14879 | -4.800 | -0.2402 | Yes |
| 256 | SLC2A10 |  |  | 14899 | -4.900 | -0.2379 | Yes |
| 257 | AGR2 |  |  | 14906 | -4.900 | -0.2348 | Yes |
| 258 | SOX13 |  |  | 14934 | -5.000 | -0.2329 | Yes |
| 259 | ACVR1B |  |  | 14954 | -5.000 | -0.2306 | Yes |
| 260 | KIFC2 |  |  | 14999 | -5.200 | -0.2297 | Yes |
| 261 | SLC44A4 |  |  | 15093 | -5.600 | -0.2317 | Yes |
| 262 | NME3 |  |  | 15106 | -5.700 | -0.2284 | Yes |
| 263 | ZBTB42 |  |  | 15114 | -5.800 | -0.2246 | Yes |
| 264 | SLC9A3R1 |  |  | 15121 | -5.800 | -0.2209 | Yes |
| 265 | CANT1 |  |  | 15165 | -6.000 | -0.2193 | Yes |
| 266 | BCAS1 |  |  | 15179 | -6.000 | -0.2159 | Yes |
| 267 | PPP1R16A |  |  | 15183 | -6.100 | -0.2117 | Yes |
| 268 | RHOB |  |  | 15194 | -6.100 | -0.2079 | Yes |
| 269 | PVRL2 |  |  | 15207 | -6.200 | -0.2042 | Yes |
| 270 | ATP8B1 |  |  | 15219 | -6.300 | -0.2004 | Yes |
| 271 | LFNG |  |  | 15268 | -6.500 | -0.1989 | Yes |
| 272 | SIDT1 |  |  | 15270 | -6.500 | -0.1942 | Yes |
| 273 | HPN |  |  | 15289 | -6.700 | -0.1906 | Yes |
| 274 | CREB3L4 |  |  | 15290 | -6.700 | -0.1858 | Yes |
| 275 | KRT19 |  |  | 15311 | -6.800 | -0.1822 | Yes |
| 276 | MLPH |  |  | 15321 | -6.900 | -0.1778 | Yes |
| 277 | GALNT6 |  |  | 15331 | -7.000 | -0.1733 | Yes |
| 278 | EPN3 |  |  | 15354 | -7.100 | -0.1697 | Yes |
| 279 | SH3GLB2 |  |  | 15361 | -7.200 | -0.1649 | Yes |
| 280 | MGAT4A |  |  | 15362 | -7.200 | -0.1597 | Yes |
| 281 | LLGL2 |  |  | 15387 | -7.400 | -0.1559 | Yes |
| 282 | CISH |  |  | 15408 | -7.600 | -0.1518 | Yes |
| 283 | MGRN1 |  |  | 15414 | -7.600 | -0.1466 | Yes |
| 284 | TFF3 |  |  | 15423 | -7.700 | -0.1416 | Yes |
| 285 | TJP3 |  |  | 15458 | -8.100 | -0.1380 | Yes |
| 286 | SLC37A1 |  |  | 15460 | -8.100 | -0.1322 | Yes |
| 287 | DDAH2 |  |  | 15469 | -8.200 | -0.1268 | Yes |
| 288 | ERBB3 |  |  | 15482 | -8.300 | -0.1216 | Yes |
| 289 | CAPN9 |  |  | 15493 | -8.400 | -0.1162 | Yes |
| 290 | DEGS2 |  |  | 15521 | -8.800 | -0.1117 | Yes |
| 291 | HMGCS2 |  |  | 15535 | -9.100 | -0.1060 | Yes |
| 292 | VIPR1 |  |  | 15540 | -9.200 | -0.0996 | Yes |
| 293 | ANXA6 |  |  | 15549 | -9.300 | -0.0934 | Yes |
| 294 | PLEKHH1 |  |  | 15552 | -9.300 | -0.0869 | Yes |
| 295 | KIF12 |  |  | 15574 | -9.700 | -0.0812 | Yes |
| 296 | RAB3D |  |  | 15592 | -10.000 | -0.0752 | Yes |
| 297 | SPDEF |  |  | 15628 | -11.200 | -0.0694 | Yes |
| 298 | STARD10 |  |  | 15644 | -11.700 | -0.0619 | Yes |
| 299 | KIAA1324 |  |  | 15680 | -13.300 | -0.0546 | Yes |
| 300 | TSPAN15 |  |  | 15695 | -14.700 | -0.0450 | Yes |
| 301 | ATP2C2 |  |  | 15698 | -15.100 | -0.0342 | Yes |
| 302 | TSPAN13 |  |  | 15709 | -16.100 | -0.0233 | Yes |
| 303 | PTPRF |  |  | 15749 | -35.900 | 0.0000 | Yes |
Table: GSEA details [plain text format]

  

Fig 2: CHARAFE\_BREAST\_CANCER\_LUMINAL\_VS\_BASAL\_UP: Random ES distribution      
 Gene set null distribution of ES for **CHARAFE\_BREAST\_CANCER\_LUMINAL\_VS\_BASAL\_UP**

  
