# Supplementary material for: Immunomic, genomic and transcriptomic characterization of CT26 colorectal carcinoma
Source: BMC Genomics. 2014 Mar 13;15(1):190. doi: 10.1186/1471-2164-15-190 (PMC4007559; doi:10.1186/1471-2164-15-190)
Supplement: Supplementary file 8 — Additional file 8: Contains the Gene Pattern gene set membership and enrichment values in an html format. The file index.html is the entry point. (ZIP 13 MB) [file 12864_2013_7028_MOESM8_ESM.zip › CHARAFE_BREAST_CANCER_LUMINAL_VS_MESENCHYMAL_UP.html]

Details for gene set CHARAFE\_BREAST\_CANCER\_LUMINAL\_VS\_MESENCHYMAL\_UP[GSEA]

|  || Dataset | CT26\_gene\_expression |
| Phenotype | NoPhenotypeAvailable |
| Upregulated in class | na\_neg |
| GeneSet | CHARAFE\_BREAST\_CANCER\_LUMINAL\_VS\_MESENCHYMAL\_UP |
| Enrichment Score (ES) | -0.51490206 |
| Normalized Enrichment Score (NES) | NaN |
| Nominal p-value | NaN |
| FDR q-value | 1.0 |
| FWER p-Value | 0.0 |
Table: GSEA Results Summary

  

Fig 1: Enrichment plot: CHARAFE\_BREAST\_CANCER\_LUMINAL\_VS\_MESENCHYMAL\_UP      
 Profile of the Running ES Score & Positions of GeneSet Members on the Rank Ordered List

  

| PROBE | GENE SYMBOL | GENE\_TITLE | RANK IN GENE LIST | RANK METRIC SCORE | RUNNING ES | CORE ENRICHMENT || 1 | COMMD3 |  |  | 2 | 66.400 | 0.0387 | No |
| 2 | ENSA |  |  | 90 | 31.100 | 0.0512 | No |
| 3 | SUOX |  |  | 687 | 16.500 | 0.0221 | No |
| 4 | PLA2G12A |  |  | 703 | 16.400 | 0.0307 | No |
| 5 | TRIB3 |  |  | 759 | 15.900 | 0.0364 | No |
| 6 | MRFAP1L1 |  |  | 836 | 15.300 | 0.0404 | No |
| 7 | GSPT1 |  |  | 970 | 14.400 | 0.0401 | No |
| 8 | EPB41L4A |  |  | 1131 | 13.400 | 0.0376 | No |
| 9 | REEP5 |  |  | 1142 | 13.300 | 0.0447 | No |
| 10 | ZNF24 |  |  | 1435 | 11.900 | 0.0327 | No |
| 11 | PCK2 |  |  | 1665 | 11.000 | 0.0242 | No |
| 12 | ISG20L2 |  |  | 1687 | 10.900 | 0.0292 | No |
| 13 | SETD6 |  |  | 1841 | 10.300 | 0.0253 | No |
| 14 | FEM1B |  |  | 1848 | 10.300 | 0.0309 | No |
| 15 | PNN |  |  | 1853 | 10.200 | 0.0366 | No |
| 16 | TNKS1BP1 |  |  | 2324 | 8.700 | 0.0111 | No |
| 17 | FAM63A |  |  | 2373 | 8.600 | 0.0130 | No |
| 18 | EMP2 |  |  | 2401 | 8.500 | 0.0162 | No |
| 19 | IRX5 |  |  | 2407 | 8.500 | 0.0209 | No |
| 20 | ICA1 |  |  | 2539 | 8.100 | 0.0171 | No |
| 21 | TMEM183A |  |  | 2574 | 8.000 | 0.0196 | No |
| 22 | IDH2 |  |  | 3040 | 6.800 | -0.0067 | No |
| 23 | UBQLN4 |  |  | 3041 | 6.800 | -0.0027 | No |
| 24 | ARF3 |  |  | 3083 | 6.700 | -0.0015 | No |
| 25 | MED28 |  |  | 3114 | 6.600 | 0.0004 | No |
| 26 | CHMP2A |  |  | 3246 | 6.300 | -0.0044 | No |
| 27 | CYB5A |  |  | 3415 | 6.000 | -0.0118 | No |
| 28 | SYMPK |  |  | 3471 | 5.800 | -0.0120 | No |
| 29 | VPS45 |  |  | 3499 | 5.800 | -0.0104 | No |
| 30 | SEPHS2 |  |  | 3703 | 5.300 | -0.0205 | No |
| 31 | PLXNA3 |  |  | 3716 | 5.300 | -0.0181 | No |
| 32 | CHD2 |  |  | 3772 | 5.200 | -0.0187 | No |
| 33 | PEX11B |  |  | 3902 | 4.900 | -0.0242 | No |
| 34 | FKBP4 |  |  | 3912 | 4.900 | -0.0219 | No |
| 35 | MREG |  |  | 3914 | 4.900 | -0.0191 | No |
| 36 | ESR1 |  |  | 3916 | 4.900 | -0.0163 | No |
| 37 | SFI1 |  |  | 3976 | 4.800 | -0.0174 | No |
| 38 | BLNK |  |  | 4021 | 4.700 | -0.0175 | No |
| 39 | MYO5B |  |  | 4092 | 4.600 | -0.0193 | No |
| 40 | PATZ1 |  |  | 4154 | 4.500 | -0.0207 | No |
| 41 | DUSP16 |  |  | 4160 | 4.500 | -0.0184 | No |
| 42 | TSC22D3 |  |  | 4183 | 4.500 | -0.0172 | No |
| 43 | IQCE |  |  | 4303 | 4.200 | -0.0225 | No |
| 44 | USP18 |  |  | 4312 | 4.200 | -0.0205 | No |
| 45 | ZKSCAN1 |  |  | 4404 | 4.000 | -0.0241 | No |
| 46 | TTC9 |  |  | 4452 | 4.000 | -0.0248 | No |
| 47 | SERF2 |  |  | 4500 | 3.900 | -0.0256 | No |
| 48 | ZCCHC8 |  |  | 4554 | 3.800 | -0.0268 | No |
| 49 | SIGIRR |  |  | 4590 | 3.700 | -0.0269 | No |
| 50 | CDC42SE1 |  |  | 4620 | 3.700 | -0.0267 | No |
| 51 | DDI2 |  |  | 4626 | 3.700 | -0.0248 | No |
| 52 | MYLIP |  |  | 4669 | 3.600 | -0.0254 | No |
| 53 | SCYL3 |  |  | 4717 | 3.500 | -0.0265 | No |
| 54 | TRPS1 |  |  | 4902 | 3.200 | -0.0365 | No |
| 55 | ZXDC |  |  | 4905 | 3.200 | -0.0348 | No |
| 56 | PADI2 |  |  | 4965 | 3.100 | -0.0368 | No |
| 57 | VRK3 |  |  | 5035 | 3.000 | -0.0396 | No |
| 58 | MRPL41 |  |  | 5102 | 2.900 | -0.0422 | No |
| 59 | ERP29 |  |  | 5129 | 2.800 | -0.0422 | No |
| 60 | DHRS13 |  |  | 5258 | 2.600 | -0.0490 | No |
| 61 | PDCD4 |  |  | 5291 | 2.600 | -0.0496 | No |
| 62 | DNAJC1 |  |  | 5344 | 2.500 | -0.0515 | No |
| 63 | RPS6KA5 |  |  | 5391 | 2.400 | -0.0531 | No |
| 64 | MCCC2 |  |  | 5422 | 2.400 | -0.0536 | No |
| 65 | ASB8 |  |  | 5457 | 2.300 | -0.0545 | No |
| 66 | ANXA9 |  |  | 5477 | 2.300 | -0.0544 | No |
| 67 | DLG3 |  |  | 5508 | 2.300 | -0.0550 | No |
| 68 | ATP6AP1 |  |  | 5625 | 2.100 | -0.0613 | No |
| 69 | DAAM1 |  |  | 5663 | 2.100 | -0.0625 | No |
| 70 | NPDC1 |  |  | 5676 | 2.100 | -0.0620 | No |
| 71 | SSBP2 |  |  | 5677 | 2.100 | -0.0608 | No |
| 72 | SSR4 |  |  | 5686 | 2.000 | -0.0602 | No |
| 73 | ZHX2 |  |  | 5714 | 2.000 | -0.0608 | No |
| 74 | SLC29A2 |  |  | 5760 | 1.900 | -0.0626 | No |
| 75 | IFT20 |  |  | 5813 | 1.900 | -0.0648 | No |
| 76 | KLRG2 |  |  | 5864 | 1.800 | -0.0670 | No |
| 77 | RFWD2 |  |  | 5915 | 1.700 | -0.0693 | No |
| 78 | KLHL28 |  |  | 5922 | 1.700 | -0.0687 | No |
| 79 | SPTLC2 |  |  | 5941 | 1.700 | -0.0689 | No |
| 80 | PLEKHF2 |  |  | 5986 | 1.600 | -0.0708 | No |
| 81 | CRABP2 |  |  | 6074 | 1.500 | -0.0756 | No |
| 82 | BLOC1S1 |  |  | 6111 | 1.500 | -0.0770 | No |
| 83 | FAM110A |  |  | 6134 | 1.400 | -0.0776 | No |
| 84 | GSTO2 |  |  | 6162 | 1.400 | -0.0786 | No |
| 85 | RERG |  |  | 6189 | 1.400 | -0.0795 | No |
| 86 | NOL3 |  |  | 6364 | 1.100 | -0.0901 | No |
| 87 | EPB41L5 |  |  | 6387 | 1.100 | -0.0909 | No |
| 88 | PPCS |  |  | 6398 | 1.100 | -0.0909 | No |
| 89 | KIAA0556 |  |  | 6408 | 1.000 | -0.0909 | No |
| 90 | FANCF |  |  | 6555 | 0.900 | -0.0999 | No |
| 91 | TNK2 |  |  | 6576 | 0.800 | -0.1007 | No |
| 92 | BCR |  |  | 6757 | 0.600 | -0.1121 | No |
| 93 | RAB5B |  |  | 6771 | 0.600 | -0.1126 | No |
| 94 | MEGF9 |  |  | 6807 | 0.600 | -0.1145 | No |
| 95 | PCDH1 |  |  | 6840 | 0.600 | -0.1162 | No |
| 96 | PPP2R5A |  |  | 6841 | 0.600 | -0.1159 | No |
| 97 | DENND2D |  |  | 6843 | 0.600 | -0.1156 | No |
| 98 | CCDC6 |  |  | 6898 | 0.500 | -0.1188 | No |
| 99 | ULK1 |  |  | 6908 | 0.500 | -0.1191 | No |
| 100 | TRIM3 |  |  | 6920 | 0.500 | -0.1195 | No |
| 101 | BOLA1 |  |  | 6956 | 0.400 | -0.1215 | No |
| 102 | RAB27B |  |  | 7009 | 0.400 | -0.1247 | No |
| 103 | RALGPS1 |  |  | 7130 | 0.300 | -0.1323 | No |
| 104 | GRHL1 |  |  | 7134 | 0.300 | -0.1323 | No |
| 105 | PER2 |  |  | 7135 | 0.300 | -0.1322 | No |
| 106 | S100A8 |  |  | 7177 | 0.200 | -0.1347 | No |
| 107 | KIAA1370 |  |  | 7249 | 0.200 | -0.1392 | No |
| 108 | JTB |  |  | 7257 | 0.200 | -0.1395 | No |
| 109 | ULK3 |  |  | 7458 | 0.100 | -0.1525 | No |
| 110 | MB |  |  | 7516 | 0.100 | -0.1561 | No |
| 111 | ARFIP2 |  |  | 7545 | 0.000 | -0.1579 | No |
| 112 | JHDM1D |  |  | 7829 | 0.000 | -0.1763 | No |
| 113 | SYCP2 |  |  | 7879 | 0.000 | -0.1795 | No |
| 114 | MMEL1 |  |  | 7957 | 0.000 | -0.1845 | No |
| 115 | MSX2 |  |  | 8468 | 0.000 | -0.2177 | No |
| 116 | NEBL |  |  | 8477 | 0.000 | -0.2182 | No |
| 117 | ALDH6A1 |  |  | 9206 | 0.000 | -0.2655 | No |
| 118 | TFF1 |  |  | 9404 | 0.000 | -0.2783 | No |
| 119 | SLC16A14 |  |  | 9538 | 0.000 | -0.2870 | No |
| 120 | PROM2 |  |  | 9924 | 0.000 | -0.3120 | No |
| 121 | PPFIBP2 |  |  | 9972 | 0.000 | -0.3150 | No |
| 122 | SLC35A1 |  |  | 10016 | 0.000 | -0.3178 | No |
| 123 | ABCA12 |  |  | 10054 | 0.000 | -0.3202 | No |
| 124 | LYPD3 |  |  | 10056 | 0.000 | -0.3203 | No |
| 125 | PRRG4 |  |  | 10093 | 0.000 | -0.3226 | No |
| 126 | PRLR |  |  | 10318 | -0.100 | -0.3371 | No |
| 127 | GATA3 |  |  | 10535 | -0.100 | -0.3511 | No |
| 128 | ANK3 |  |  | 10538 | -0.100 | -0.3512 | No |
| 129 | CACNA1D |  |  | 10636 | -0.100 | -0.3574 | No |
| 130 | CTNND2 |  |  | 10706 | -0.100 | -0.3619 | No |
| 131 | FGD3 |  |  | 10949 | -0.200 | -0.3775 | No |
| 132 | RHOH |  |  | 10973 | -0.200 | -0.3788 | No |
| 133 | COG7 |  |  | 11011 | -0.200 | -0.3811 | No |
| 134 | CSAD |  |  | 11023 | -0.200 | -0.3817 | No |
| 135 | WFS1 |  |  | 11038 | -0.200 | -0.3825 | No |
| 136 | RHPN1 |  |  | 11044 | -0.200 | -0.3827 | No |
| 137 | KIAA0319L |  |  | 11048 | -0.200 | -0.3828 | No |
| 138 | CCDC64 |  |  | 11054 | -0.200 | -0.3830 | No |
| 139 | DBP |  |  | 11091 | -0.200 | -0.3852 | No |
| 140 | AZGP1 |  |  | 11151 | -0.200 | -0.3890 | No |
| 141 | MPP7 |  |  | 11182 | -0.300 | -0.3907 | No |
| 142 | INADL |  |  | 11324 | -0.300 | -0.3997 | No |
| 143 | FUT1 |  |  | 11332 | -0.300 | -0.4000 | No |
| 144 | INHBB |  |  | 11390 | -0.300 | -0.4035 | No |
| 145 | KRT23 |  |  | 11398 | -0.300 | -0.4038 | No |
| 146 | CHMP4C |  |  | 11402 | -0.300 | -0.4038 | No |
| 147 | ANKRD22 |  |  | 11403 | -0.300 | -0.4037 | No |
| 148 | VAV3 |  |  | 11471 | -0.400 | -0.4078 | No |
| 149 | SLC44A2 |  |  | 11504 | -0.400 | -0.4096 | No |
| 150 | BLVRB |  |  | 11639 | -0.400 | -0.4181 | No |
| 151 | CACNG4 |  |  | 11818 | -0.500 | -0.4294 | No |
| 152 | FAM59A |  |  | 11902 | -0.600 | -0.4344 | No |
| 153 | NR2F6 |  |  | 11920 | -0.600 | -0.4352 | No |
| 154 | CDH3 |  |  | 11996 | -0.600 | -0.4397 | No |
| 155 | GPR157 |  |  | 12013 | -0.700 | -0.4403 | No |
| 156 | FBP1 |  |  | 12043 | -0.700 | -0.4418 | No |
| 157 | FREM2 |  |  | 12070 | -0.700 | -0.4431 | No |
| 158 | ABCG1 |  |  | 12119 | -0.700 | -0.4458 | No |
| 159 | SLC7A8 |  |  | 12136 | -0.700 | -0.4464 | No |
| 160 | TMPRSS13 |  |  | 12159 | -0.700 | -0.4474 | No |
| 161 | PLCH1 |  |  | 12192 | -0.800 | -0.4491 | No |
| 162 | PVRL4 |  |  | 12257 | -0.800 | -0.4527 | No |
| 163 | EXPH5 |  |  | 12260 | -0.800 | -0.4524 | No |
| 164 | LMTK3 |  |  | 12269 | -0.800 | -0.4525 | No |
| 165 | LNX1 |  |  | 12302 | -0.900 | -0.4540 | No |
| 166 | RNF103 |  |  | 12333 | -0.900 | -0.4554 | No |
| 167 | SH3YL1 |  |  | 12342 | -0.900 | -0.4554 | No |
| 168 | EFHD1 |  |  | 12480 | -1.000 | -0.4638 | No |
| 169 | APH1A |  |  | 12495 | -1.000 | -0.4641 | No |
| 170 | CADPS2 |  |  | 12565 | -1.000 | -0.4680 | No |
| 171 | TMEM41A |  |  | 12578 | -1.000 | -0.4682 | No |
| 172 | CHN2 |  |  | 12584 | -1.000 | -0.4679 | No |
| 173 | PREX1 |  |  | 12737 | -1.200 | -0.4771 | No |
| 174 | EPS8L1 |  |  | 12778 | -1.200 | -0.4790 | No |
| 175 | TPD52L1 |  |  | 12800 | -1.200 | -0.4797 | No |
| 176 | SHANK2 |  |  | 12864 | -1.300 | -0.4830 | No |
| 177 | DNAJA4 |  |  | 12970 | -1.400 | -0.4890 | No |
| 178 | CYHR1 |  |  | 12998 | -1.400 | -0.4899 | No |
| 179 | FUK |  |  | 13002 | -1.500 | -0.4893 | No |
| 180 | MGMT |  |  | 13003 | -1.500 | -0.4884 | No |
| 181 | SYNGR2 |  |  | 13026 | -1.500 | -0.4889 | No |
| 182 | MYB |  |  | 13065 | -1.500 | -0.4905 | No |
| 183 | MCF2L |  |  | 13074 | -1.500 | -0.4902 | No |
| 184 | HOOK1 |  |  | 13159 | -1.600 | -0.4947 | No |
| 185 | PPP1R3D |  |  | 13180 | -1.700 | -0.4950 | No |
| 186 | RAB17 |  |  | 13310 | -1.800 | -0.5023 | No |
| 187 | SYNGR1 |  |  | 13320 | -1.800 | -0.5019 | No |
| 188 | DOK7 |  |  | 13377 | -1.900 | -0.5044 | No |
| 189 | RASEF |  |  | 13381 | -1.900 | -0.5035 | No |
| 190 | TOB1 |  |  | 13383 | -1.900 | -0.5024 | No |
| 191 | PLXNB1 |  |  | 13408 | -1.900 | -0.5029 | No |
| 192 | CASZ1 |  |  | 13441 | -1.900 | -0.5039 | No |
| 193 | PRODH |  |  | 13507 | -2.000 | -0.5069 | No |
| 194 | SMPDL3B |  |  | 13513 | -2.000 | -0.5061 | No |
| 195 | KIAA0247 |  |  | 13534 | -2.100 | -0.5061 | No |
| 196 | CYP4B1 |  |  | 13578 | -2.100 | -0.5077 | No |
| 197 | RAB11FIP4 |  |  | 13591 | -2.100 | -0.5073 | No |
| 198 | TMED3 |  |  | 13623 | -2.200 | -0.5080 | No |
| 199 | SYT7 |  |  | 13662 | -2.200 | -0.5092 | No |
| 200 | XBP1 |  |  | 13685 | -2.300 | -0.5093 | No |
| 201 | KIAA1244 |  |  | 13722 | -2.400 | -0.5102 | No |
| 202 | CLN3 |  |  | 13725 | -2.400 | -0.5089 | No |
| 203 | RHBDF1 |  |  | 13767 | -2.400 | -0.5102 | No |
| 204 | ABAT |  |  | 13779 | -2.400 | -0.5095 | No |
| 205 | EFNA4 |  |  | 13863 | -2.600 | -0.5134 | Yes |
| 206 | EPHA1 |  |  | 13887 | -2.600 | -0.5134 | Yes |
| 207 | BIK |  |  | 13901 | -2.600 | -0.5127 | Yes |
| 208 | TMEM134 |  |  | 13907 | -2.600 | -0.5115 | Yes |
| 209 | CA12 |  |  | 13912 | -2.700 | -0.5102 | Yes |
| 210 | ENPP5 |  |  | 13936 | -2.700 | -0.5101 | Yes |
| 211 | TOX3 |  |  | 13964 | -2.700 | -0.5103 | Yes |
| 212 | PRR15 |  |  | 13978 | -2.700 | -0.5095 | Yes |
| 213 | MYO5C |  |  | 13983 | -2.700 | -0.5082 | Yes |
| 214 | RGL2 |  |  | 13985 | -2.800 | -0.5067 | Yes |
| 215 | CCDC97 |  |  | 13998 | -2.800 | -0.5058 | Yes |
| 216 | RABEP2 |  |  | 14003 | -2.800 | -0.5044 | Yes |
| 217 | GPD1L |  |  | 14023 | -2.800 | -0.5040 | Yes |
| 218 | FAM46C |  |  | 14047 | -2.900 | -0.5038 | Yes |
| 219 | MUC1 |  |  | 14055 | -2.900 | -0.5026 | Yes |
| 220 | GRHL2 |  |  | 14075 | -2.900 | -0.5021 | Yes |
| 221 | TMBIM4 |  |  | 14139 | -3.000 | -0.5045 | Yes |
| 222 | CAMK2N1 |  |  | 14149 | -3.000 | -0.5033 | Yes |
| 223 | PDXDC1 |  |  | 14163 | -3.000 | -0.5024 | Yes |
| 224 | MYO6 |  |  | 14195 | -3.100 | -0.5026 | Yes |
| 225 | GPR160 |  |  | 14205 | -3.100 | -0.5014 | Yes |
| 226 | ZNF467 |  |  | 14227 | -3.200 | -0.5009 | Yes |
| 227 | MAL2 |  |  | 14235 | -3.200 | -0.4995 | Yes |
| 228 | PPM1H |  |  | 14278 | -3.300 | -0.5003 | Yes |
| 229 | RAB40C |  |  | 14306 | -3.300 | -0.5001 | Yes |
| 230 | PTPN6 |  |  | 14308 | -3.300 | -0.4982 | Yes |
| 231 | CACNB3 |  |  | 14311 | -3.400 | -0.4964 | Yes |
| 232 | GOLT1A |  |  | 14315 | -3.400 | -0.4946 | Yes |
| 233 | S100A14 |  |  | 14322 | -3.400 | -0.4930 | Yes |
| 234 | ARRDC4 |  |  | 14327 | -3.400 | -0.4913 | Yes |
| 235 | ARHGEF5 |  |  | 14353 | -3.400 | -0.4909 | Yes |
| 236 | TM7SF2 |  |  | 14430 | -3.700 | -0.4937 | Yes |
| 237 | SCAMP2 |  |  | 14473 | -3.800 | -0.4942 | Yes |
| 238 | TRAF4 |  |  | 14489 | -3.800 | -0.4929 | Yes |
| 239 | ALDH3B2 |  |  | 14512 | -3.900 | -0.4921 | Yes |
| 240 | SPATA2L |  |  | 14514 | -3.900 | -0.4899 | Yes |
| 241 | OVOL2 |  |  | 14530 | -3.900 | -0.4886 | Yes |
| 242 | RUSC1 |  |  | 14540 | -3.900 | -0.4869 | Yes |
| 243 | CEBPA |  |  | 14543 | -3.900 | -0.4847 | Yes |
| 244 | SLC25A29 |  |  | 14549 | -3.900 | -0.4828 | Yes |
| 245 | IRF6 |  |  | 14564 | -4.000 | -0.4814 | Yes |
| 246 | FA2H |  |  | 14570 | -4.000 | -0.4793 | Yes |
| 247 | CYB561 |  |  | 14574 | -4.000 | -0.4772 | Yes |
| 248 | SERINC2 |  |  | 14597 | -4.100 | -0.4762 | Yes |
| 249 | TLE3 |  |  | 14599 | -4.100 | -0.4739 | Yes |
| 250 | HIP1R |  |  | 14605 | -4.100 | -0.4718 | Yes |
| 251 | MARVELD2 |  |  | 14626 | -4.100 | -0.4707 | Yes |
| 252 | TMEM125 |  |  | 14632 | -4.200 | -0.4686 | Yes |
| 253 | TMEM30B |  |  | 14665 | -4.200 | -0.4682 | Yes |
| 254 | CIRBP |  |  | 14671 | -4.300 | -0.4661 | Yes |
| 255 | FLAD1 |  |  | 14713 | -4.400 | -0.4661 | Yes |
| 256 | ARRDC1 |  |  | 14716 | -4.400 | -0.4637 | Yes |
| 257 | HOOK2 |  |  | 14725 | -4.400 | -0.4617 | Yes |
| 258 | ABHD11 |  |  | 14728 | -4.400 | -0.4592 | Yes |
| 259 | DDR1 |  |  | 14778 | -4.500 | -0.4598 | Yes |
| 260 | RMND5B |  |  | 14795 | -4.600 | -0.4581 | Yes |
| 261 | PRKD2 |  |  | 14797 | -4.600 | -0.4555 | Yes |
| 262 | FOXA1 |  |  | 14800 | -4.600 | -0.4529 | Yes |
| 263 | MYO1D |  |  | 14801 | -4.600 | -0.4503 | Yes |
| 264 | KIAA1522 |  |  | 14819 | -4.600 | -0.4487 | Yes |
| 265 | EPHB3 |  |  | 14823 | -4.700 | -0.4461 | Yes |
| 266 | FRAT2 |  |  | 14829 | -4.700 | -0.4437 | Yes |
| 267 | ADCY6 |  |  | 14830 | -4.700 | -0.4410 | Yes |
| 268 | CGN |  |  | 14839 | -4.700 | -0.4387 | Yes |
| 269 | AGR2 |  |  | 14906 | -4.900 | -0.4402 | Yes |
| 270 | SOX13 |  |  | 14934 | -5.000 | -0.4390 | Yes |
| 271 | ACVR1B |  |  | 14954 | -5.000 | -0.4373 | Yes |
| 272 | TMC4 |  |  | 14959 | -5.100 | -0.4346 | Yes |
| 273 | FAAH |  |  | 14961 | -5.100 | -0.4317 | Yes |
| 274 | TMEM45B |  |  | 14978 | -5.100 | -0.4297 | Yes |
| 275 | KIFC2 |  |  | 14999 | -5.200 | -0.4280 | Yes |
| 276 | FAM102A |  |  | 15011 | -5.200 | -0.4257 | Yes |
| 277 | ST6GALNAC2 |  |  | 15090 | -5.600 | -0.4275 | Yes |
| 278 | ROGDI |  |  | 15096 | -5.600 | -0.4245 | Yes |
| 279 | TUFT1 |  |  | 15100 | -5.700 | -0.4214 | Yes |
| 280 | TMEM79 |  |  | 15103 | -5.700 | -0.4182 | Yes |
| 281 | NME3 |  |  | 15106 | -5.700 | -0.4150 | Yes |
| 282 | ZBTB42 |  |  | 15114 | -5.800 | -0.4121 | Yes |
| 283 | TRPM4 |  |  | 15118 | -5.800 | -0.4089 | Yes |
| 284 | SLC9A3R1 |  |  | 15121 | -5.800 | -0.4056 | Yes |
| 285 | FXYD3 |  |  | 15130 | -5.900 | -0.4027 | Yes |
| 286 | FAM83H |  |  | 15145 | -5.900 | -0.4002 | Yes |
| 287 | MYH14 |  |  | 15164 | -6.000 | -0.3978 | Yes |
| 288 | MAP7 |  |  | 15177 | -6.000 | -0.3951 | Yes |
| 289 | BCAS1 |  |  | 15179 | -6.000 | -0.3917 | Yes |
| 290 | PPP1R16A |  |  | 15183 | -6.100 | -0.3883 | Yes |
| 291 | OVOL1 |  |  | 15189 | -6.100 | -0.3851 | Yes |
| 292 | RHOB |  |  | 15194 | -6.100 | -0.3818 | Yes |
| 293 | H2AFJ |  |  | 15204 | -6.200 | -0.3787 | Yes |
| 294 | SEMA4A |  |  | 15231 | -6.400 | -0.3767 | Yes |
| 295 | LIMK2 |  |  | 15249 | -6.400 | -0.3740 | Yes |
| 296 | MARVELD3 |  |  | 15255 | -6.500 | -0.3706 | Yes |
| 297 | F11R |  |  | 15257 | -6.500 | -0.3668 | Yes |
| 298 | SIDT1 |  |  | 15270 | -6.500 | -0.3638 | Yes |
| 299 | TSPAN1 |  |  | 15280 | -6.600 | -0.3605 | Yes |
| 300 | SYTL1 |  |  | 15286 | -6.600 | -0.3570 | Yes |
| 301 | CREB3L4 |  |  | 15290 | -6.700 | -0.3533 | Yes |
| 302 | SEMA3F |  |  | 15305 | -6.800 | -0.3502 | Yes |
| 303 | KRT19 |  |  | 15311 | -6.800 | -0.3466 | Yes |
| 304 | CLDN4 |  |  | 15314 | -6.800 | -0.3428 | Yes |
| 305 | CRIP2 |  |  | 15318 | -6.900 | -0.3389 | Yes |
| 306 | MLPH |  |  | 15321 | -6.900 | -0.3350 | Yes |
| 307 | ALDH4A1 |  |  | 15325 | -6.900 | -0.3312 | Yes |
| 308 | CLDN3 |  |  | 15328 | -6.900 | -0.3273 | Yes |
| 309 | GALNT6 |  |  | 15331 | -7.000 | -0.3233 | Yes |
| 310 | EPN3 |  |  | 15354 | -7.100 | -0.3206 | Yes |
| 311 | ZBTB7B |  |  | 15356 | -7.100 | -0.3165 | Yes |
| 312 | MGAT4A |  |  | 15362 | -7.200 | -0.3126 | Yes |
| 313 | LLGL2 |  |  | 15387 | -7.400 | -0.3099 | Yes |
| 314 | MKNK2 |  |  | 15392 | -7.400 | -0.3058 | Yes |
| 315 | SULT2B1 |  |  | 15411 | -7.600 | -0.3026 | Yes |
| 316 | SEPP1 |  |  | 15421 | -7.700 | -0.2986 | Yes |
| 317 | TFF3 |  |  | 15423 | -7.700 | -0.2942 | Yes |
| 318 | PRSS8 |  |  | 15441 | -7.900 | -0.2907 | Yes |
| 319 | BSPRY |  |  | 15450 | -8.000 | -0.2865 | Yes |
| 320 | TJP3 |  |  | 15458 | -8.100 | -0.2823 | Yes |
| 321 | PKP3 |  |  | 15459 | -8.100 | -0.2775 | Yes |
| 322 | SLC37A1 |  |  | 15460 | -8.100 | -0.2728 | Yes |
| 323 | EHF |  |  | 15467 | -8.200 | -0.2684 | Yes |
| 324 | GRB7 |  |  | 15481 | -8.300 | -0.2644 | Yes |
| 325 | ERBB3 |  |  | 15482 | -8.300 | -0.2596 | Yes |
| 326 | GPR56 |  |  | 15489 | -8.400 | -0.2550 | Yes |
| 327 | ELMO3 |  |  | 15519 | -8.800 | -0.2518 | Yes |
| 328 | DEGS2 |  |  | 15521 | -8.800 | -0.2467 | Yes |
| 329 | CRIP1 |  |  | 15534 | -9.000 | -0.2422 | Yes |
| 330 | VIPR1 |  |  | 15540 | -9.200 | -0.2372 | Yes |
| 331 | TRAPPC6A |  |  | 15541 | -9.200 | -0.2318 | Yes |
| 332 | IGFBP2 |  |  | 15542 | -9.200 | -0.2264 | Yes |
| 333 | ITPK1 |  |  | 15545 | -9.300 | -0.2211 | Yes |
| 334 | PAQR4 |  |  | 15560 | -9.500 | -0.2165 | Yes |
| 335 | SSH3 |  |  | 15564 | -9.600 | -0.2111 | Yes |
| 336 | ENTPD2 |  |  | 15575 | -9.700 | -0.2061 | Yes |
| 337 | CDS1 |  |  | 15590 | -10.000 | -0.2011 | Yes |
| 338 | RAB3D |  |  | 15592 | -10.000 | -0.1954 | Yes |
| 339 | EPPK1 |  |  | 15597 | -10.100 | -0.1897 | Yes |
| 340 | SORL1 |  |  | 15613 | -10.500 | -0.1846 | Yes |
| 341 | SPINT1 |  |  | 15618 | -10.800 | -0.1785 | Yes |
| 342 | LSR |  |  | 15620 | -10.900 | -0.1722 | Yes |
| 343 | CBLC |  |  | 15624 | -11.000 | -0.1660 | Yes |
| 344 | TMPRSS2 |  |  | 15627 | -11.100 | -0.1596 | Yes |
| 345 | SPDEF |  |  | 15628 | -11.200 | -0.1531 | Yes |
| 346 | ST14 |  |  | 15630 | -11.300 | -0.1466 | Yes |
| 347 | ERBB2 |  |  | 15634 | -11.400 | -0.1401 | Yes |
| 348 | SPINT2 |  |  | 15640 | -11.600 | -0.1337 | Yes |
| 349 | STARD10 |  |  | 15644 | -11.700 | -0.1270 | Yes |
| 350 | CNNM4 |  |  | 15651 | -11.900 | -0.1205 | Yes |
| 351 | PLEKHA6 |  |  | 15666 | -12.500 | -0.1141 | Yes |
| 352 | JUP |  |  | 15672 | -12.700 | -0.1070 | Yes |
| 353 | LAD1 |  |  | 15673 | -12.900 | -0.0994 | Yes |
| 354 | KIAA1324 |  |  | 15680 | -13.300 | -0.0921 | Yes |
| 355 | ATP2C2 |  |  | 15698 | -15.100 | -0.0843 | Yes |
| 356 | CLDN7 |  |  | 15700 | -15.300 | -0.0755 | Yes |
| 357 | SELENBP1 |  |  | 15707 | -15.900 | -0.0666 | Yes |
| 358 | ELF3 |  |  | 15708 | -16.000 | -0.0572 | Yes |
| 359 | TSPAN13 |  |  | 15709 | -16.100 | -0.0478 | Yes |
| 360 | GGT6 |  |  | 15710 | -16.100 | -0.0384 | Yes |
| 361 | CDH1 |  |  | 15723 | -17.500 | -0.0290 | Yes |
| 362 | AP1M2 |  |  | 15724 | -17.800 | -0.0186 | Yes |
| 363 | RAB25 |  |  | 15748 | -34.500 | 0.0001 | Yes |
Table: GSEA details [plain text format]

  

Fig 2: CHARAFE\_BREAST\_CANCER\_LUMINAL\_VS\_MESENCHYMAL\_UP: Random ES distribution      
 Gene set null distribution of ES for **CHARAFE\_BREAST\_CANCER\_LUMINAL\_VS\_MESENCHYMAL\_UP**

  
