# Supplementary material for: Immunomic, genomic and transcriptomic characterization of CT26 colorectal carcinoma
Source: BMC Genomics. 2014 Mar 13;15(1):190. doi: 10.1186/1471-2164-15-190 (PMC4007559; doi:10.1186/1471-2164-15-190)
Supplement: Supplementary file 8 — Additional file 8: Contains the Gene Pattern gene set membership and enrichment values in an html format. The file index.html is the entry point. (ZIP 13 MB) [file 12864_2013_7028_MOESM8_ESM.zip › DELPUECH_FOXO3_TARGETS_DN.html]

Details for gene set DELPUECH\_FOXO3\_TARGETS\_DN[GSEA]

|  || Dataset | CT26\_gene\_expression |
| Phenotype | NoPhenotypeAvailable |
| Upregulated in class | na\_pos |
| GeneSet | DELPUECH\_FOXO3\_TARGETS\_DN |
| Enrichment Score (ES) | 0.8214733 |
| Normalized Enrichment Score (NES) | 1.7180318 |
| Nominal p-value | 0.0 |
| FDR q-value | 0.0016714783 |
| FWER p-Value | 0.013 |
Table: GSEA Results Summary

  

Fig 1: Enrichment plot: DELPUECH\_FOXO3\_TARGETS\_DN      
 Profile of the Running ES Score & Positions of GeneSet Members on the Rank Ordered List

  

| PROBE | GENE SYMBOL | GENE\_TITLE | RANK IN GENE LIST | RANK METRIC SCORE | RUNNING ES | CORE ENRICHMENT || 1 | SMC4 |  |  | 1 | 76.300 | 0.1317 | Yes |
| 2 | HAX1 |  |  | 70 | 33.100 | 0.1845 | Yes |
| 3 | EXOSC8 |  |  | 72 | 32.500 | 0.2405 | Yes |
| 4 | PRC1 |  |  | 181 | 25.500 | 0.2777 | Yes |
| 5 | LARS |  |  | 247 | 23.200 | 0.3136 | Yes |
| 6 | MTHFD2 |  |  | 255 | 22.900 | 0.3527 | Yes |
| 7 | FOXM1 |  |  | 322 | 21.200 | 0.3851 | Yes |
| 8 | NEK2 |  |  | 346 | 20.900 | 0.4197 | Yes |
| 9 | XPOT |  |  | 365 | 20.500 | 0.4540 | Yes |
| 10 | SHMT2 |  |  | 375 | 20.300 | 0.4884 | Yes |
| 11 | IARS |  |  | 384 | 20.200 | 0.5228 | Yes |
| 12 | MKI67 |  |  | 414 | 19.700 | 0.5550 | Yes |
| 13 | CENPF |  |  | 431 | 19.400 | 0.5875 | Yes |
| 14 | KIF22 |  |  | 461 | 18.900 | 0.6182 | Yes |
| 15 | CEBPG |  |  | 532 | 18.100 | 0.6450 | Yes |
| 16 | ZWINT |  |  | 574 | 17.700 | 0.6730 | Yes |
| 17 | NUSAP1 |  |  | 716 | 16.200 | 0.6920 | Yes |
| 18 | AURKA |  |  | 799 | 15.600 | 0.7137 | Yes |
| 19 | IMPDH2 |  |  | 826 | 15.400 | 0.7386 | Yes |
| 20 | AARS |  |  | 846 | 15.300 | 0.7638 | Yes |
| 21 | VEGFA |  |  | 946 | 14.600 | 0.7827 | Yes |
| 22 | SLC7A1 |  |  | 1173 | 13.100 | 0.7910 | Yes |
| 23 | APEX1 |  |  | 1563 | 11.400 | 0.7859 | Yes |
| 24 | PCK2 |  |  | 1665 | 11.000 | 0.7985 | Yes |
| 25 | PFN2 |  |  | 1781 | 10.500 | 0.8093 | Yes |
| 26 | SLC1A4 |  |  | 1867 | 10.200 | 0.8215 | Yes |
| 27 | TIAL1 |  |  | 2775 | 7.500 | 0.7767 | No |
| 28 | UBE2C |  |  | 2879 | 7.200 | 0.7826 | No |
| 29 | SNORD47 |  |  | 4001 | 4.800 | 0.7195 | No |
| 30 | H1F0 |  |  | 5042 | 3.000 | 0.6585 | No |
| 31 | EFNB2 |  |  | 5270 | 2.600 | 0.6485 | No |
| 32 | GADD45A |  |  | 5475 | 2.300 | 0.6395 | No |
| 33 | NUPR1 |  |  | 5649 | 2.100 | 0.6321 | No |
| 34 | MB |  |  | 7516 | 0.100 | 0.5136 | No |
| 35 | PITX2 |  |  | 8960 | 0.000 | 0.4217 | No |
| 36 | PYCR1 |  |  | 11772 | -0.500 | 0.2437 | No |
| 37 | AREG |  |  | 12421 | -0.900 | 0.2040 | No |
| 38 | CDC42EP3 |  |  | 14774 | -4.500 | 0.0621 | No |
Table: GSEA details [plain text format]

  

Fig 2: DELPUECH\_FOXO3\_TARGETS\_DN: Random ES distribution      
 Gene set null distribution of ES for **DELPUECH\_FOXO3\_TARGETS\_DN**

  
