# Supplementary material for: Immunomic, genomic and transcriptomic characterization of CT26 colorectal carcinoma
Source: BMC Genomics. 2014 Mar 13;15(1):190. doi: 10.1186/1471-2164-15-190 (PMC4007559; doi:10.1186/1471-2164-15-190)
Supplement: Supplementary file 8 — Additional file 8: Contains the Gene Pattern gene set membership and enrichment values in an html format. The file index.html is the entry point. (ZIP 13 MB) [file 12864_2013_7028_MOESM8_ESM.zip › EGUCHI_CELL_CYCLE_RB1_TARGETS.html]

Details for gene set EGUCHI\_CELL\_CYCLE\_RB1\_TARGETS[GSEA]

|  || Dataset | CT26\_gene\_expression |
| Phenotype | NoPhenotypeAvailable |
| Upregulated in class | na\_pos |
| GeneSet | EGUCHI\_CELL\_CYCLE\_RB1\_TARGETS |
| Enrichment Score (ES) | 0.9043682 |
| Normalized Enrichment Score (NES) | 1.8121259 |
| Nominal p-value | 0.0 |
| FDR q-value | 0.0 |
| FWER p-Value | 0.0 |
Table: GSEA Results Summary

  

Fig 1: Enrichment plot: EGUCHI\_CELL\_CYCLE\_RB1\_TARGETS      
 Profile of the Running ES Score & Positions of GeneSet Members on the Rank Ordered List

  

| PROBE | GENE SYMBOL | GENE\_TITLE | RANK IN GENE LIST | RANK METRIC SCORE | RUNNING ES | CORE ENRICHMENT || 1 | SMC4 |  |  | 1 | 76.300 | 0.1490 | Yes |
| 2 | KIF20A |  |  | 77 | 31.900 | 0.2065 | Yes |
| 3 | MCM4 |  |  | 81 | 31.500 | 0.2679 | Yes |
| 4 | ECT2 |  |  | 100 | 30.500 | 0.3263 | Yes |
| 5 | MCM6 |  |  | 163 | 26.600 | 0.3743 | Yes |
| 6 | CCNA2 |  |  | 178 | 25.600 | 0.4235 | Yes |
| 7 | KIF11 |  |  | 197 | 24.700 | 0.4706 | Yes |
| 8 | MCM7 |  |  | 290 | 21.900 | 0.5075 | Yes |
| 9 | MCM3 |  |  | 315 | 21.400 | 0.5478 | Yes |
| 10 | NEK2 |  |  | 346 | 20.900 | 0.5867 | Yes |
| 11 | BUB1 |  |  | 360 | 20.600 | 0.6261 | Yes |
| 12 | SMC2 |  |  | 379 | 20.300 | 0.6646 | Yes |
| 13 | CENPE |  |  | 391 | 20.100 | 0.7032 | Yes |
| 14 | NCAPG |  |  | 524 | 18.200 | 0.7304 | Yes |
| 15 | CCNE2 |  |  | 743 | 16.000 | 0.7477 | Yes |
| 16 | NDC80 |  |  | 878 | 15.000 | 0.7685 | Yes |
| 17 | CDCA8 |  |  | 1017 | 14.100 | 0.7873 | Yes |
| 18 | FEN1 |  |  | 1052 | 13.900 | 0.8123 | Yes |
| 19 | CDC25C |  |  | 1075 | 13.700 | 0.8377 | Yes |
| 20 | HMGB2 |  |  | 1279 | 12.700 | 0.8496 | Yes |
| 21 | MCM2 |  |  | 1281 | 12.700 | 0.8743 | Yes |
| 22 | KIF18A |  |  | 1462 | 11.800 | 0.8859 | Yes |
| 23 | MCM5 |  |  | 1526 | 11.500 | 0.9044 | Yes |
Table: GSEA details [plain text format]

  

Fig 2: EGUCHI\_CELL\_CYCLE\_RB1\_TARGETS: Random ES distribution      
 Gene set null distribution of ES for **EGUCHI\_CELL\_CYCLE\_RB1\_TARGETS**

  
