# Supplementary material for: Immunomic, genomic and transcriptomic characterization of CT26 colorectal carcinoma
Source: BMC Genomics. 2014 Mar 13;15(1):190. doi: 10.1186/1471-2164-15-190 (PMC4007559; doi:10.1186/1471-2164-15-190)
Supplement: Supplementary file 8 — Additional file 8: Contains the Gene Pattern gene set membership and enrichment values in an html format. The file index.html is the entry point. (ZIP 13 MB) [file 12864_2013_7028_MOESM8_ESM.zip › FARMER_BREAST_CANCER_CLUSTER_2.html]

Details for gene set FARMER\_BREAST\_CANCER\_CLUSTER\_2[GSEA]

|  || Dataset | CT26\_gene\_expression |
| Phenotype | NoPhenotypeAvailable |
| Upregulated in class | na\_pos |
| GeneSet | FARMER\_BREAST\_CANCER\_CLUSTER\_2 |
| Enrichment Score (ES) | 0.8244365 |
| Normalized Enrichment Score (NES) | 1.6931728 |
| Nominal p-value | 0.0 |
| FDR q-value | 0.0020072397 |
| FWER p-Value | 0.029 |
Table: GSEA Results Summary

  

Fig 1: Enrichment plot: FARMER\_BREAST\_CANCER\_CLUSTER\_2      
 Profile of the Running ES Score & Positions of GeneSet Members on the Rank Ordered List

  

| PROBE | GENE SYMBOL | GENE\_TITLE | RANK IN GENE LIST | RANK METRIC SCORE | RUNNING ES | CORE ENRICHMENT || 1 | TPX2 |  |  | 39 | 37.700 | 0.0681 | Yes |
| 2 | KIF20A |  |  | 77 | 31.900 | 0.1254 | Yes |
| 3 | MCM4 |  |  | 81 | 31.500 | 0.1841 | Yes |
| 4 | CCNA2 |  |  | 178 | 25.600 | 0.2259 | Yes |
| 5 | PRC1 |  |  | 181 | 25.500 | 0.2735 | Yes |
| 6 | KIF11 |  |  | 197 | 24.700 | 0.3187 | Yes |
| 7 | PTTG1 |  |  | 223 | 23.900 | 0.3619 | Yes |
| 8 | NEK2 |  |  | 346 | 20.900 | 0.3932 | Yes |
| 9 | BUB1 |  |  | 360 | 20.600 | 0.4309 | Yes |
| 10 | CENPE |  |  | 391 | 20.100 | 0.4666 | Yes |
| 11 | NCAPG |  |  | 524 | 18.200 | 0.4923 | Yes |
| 12 | MAD2L1 |  |  | 559 | 17.900 | 0.5236 | Yes |
| 13 | ATAD2 |  |  | 596 | 17.400 | 0.5539 | Yes |
| 14 | BUB1B |  |  | 609 | 17.300 | 0.5855 | Yes |
| 15 | KIF4A |  |  | 648 | 16.900 | 0.6147 | Yes |
| 16 | CEP55 |  |  | 662 | 16.800 | 0.6453 | Yes |
| 17 | NUSAP1 |  |  | 716 | 16.200 | 0.6722 | Yes |
| 18 | NDC80 |  |  | 878 | 15.000 | 0.6900 | Yes |
| 19 | MELK |  |  | 1077 | 13.700 | 0.7031 | Yes |
| 20 | CCNB2 |  |  | 1155 | 13.200 | 0.7229 | Yes |
| 21 | ARMC1 |  |  | 1191 | 13.100 | 0.7451 | Yes |
| 22 | MTFR1 |  |  | 1311 | 12.500 | 0.7610 | Yes |
| 23 | RRS1 |  |  | 1355 | 12.300 | 0.7812 | Yes |
| 24 | MTERFD1 |  |  | 1719 | 10.800 | 0.7783 | Yes |
| 25 | KIF15 |  |  | 1723 | 10.700 | 0.7982 | Yes |
| 26 | CENPA |  |  | 1762 | 10.600 | 0.8156 | Yes |
| 27 | ASPM |  |  | 1918 | 10.000 | 0.8244 | Yes |
| 28 | SLC25A32 |  |  | 2451 | 8.300 | 0.8061 | No |
| 29 | RB1CC1 |  |  | 2782 | 7.400 | 0.7990 | No |
| 30 | MRPL15 |  |  | 2818 | 7.400 | 0.8106 | No |
| 31 | RAD54B |  |  | 3981 | 4.800 | 0.7456 | No |
| 32 | PRKDC |  |  | 5996 | 1.600 | 0.6205 | No |
Table: GSEA details [plain text format]

  

Fig 2: FARMER\_BREAST\_CANCER\_CLUSTER\_2: Random ES distribution      
 Gene set null distribution of ES for **FARMER\_BREAST\_CANCER\_CLUSTER\_2**

  
