# Supplementary material for: Immunomic, genomic and transcriptomic characterization of CT26 colorectal carcinoma
Source: BMC Genomics. 2014 Mar 13;15(1):190. doi: 10.1186/1471-2164-15-190 (PMC4007559; doi:10.1186/1471-2164-15-190)
Supplement: Supplementary file 8 — Additional file 8: Contains the Gene Pattern gene set membership and enrichment values in an html format. The file index.html is the entry point. (ZIP 13 MB) [file 12864_2013_7028_MOESM8_ESM.zip › HUTTMANN_B_CLL_POOR_SURVIVAL_UP.html]

Details for gene set HUTTMANN\_B\_CLL\_POOR\_SURVIVAL\_UP[GSEA]

|  || Dataset | CT26\_gene\_expression |
| Phenotype | NoPhenotypeAvailable |
| Upregulated in class | na\_neg |
| GeneSet | HUTTMANN\_B\_CLL\_POOR\_SURVIVAL\_UP |
| Enrichment Score (ES) | -0.2551446 |
| Normalized Enrichment Score (NES) | NaN |
| Nominal p-value | NaN |
| FDR q-value | 1.0 |
| FWER p-Value | 0.0 |
Table: GSEA Results Summary

  

Fig 1: Enrichment plot: HUTTMANN\_B\_CLL\_POOR\_SURVIVAL\_UP      
 Profile of the Running ES Score & Positions of GeneSet Members on the Rank Ordered List

  

| PROBE | GENE SYMBOL | GENE\_TITLE | RANK IN GENE LIST | RANK METRIC SCORE | RUNNING ES | CORE ENRICHMENT || 1 | NCKAP1 |  |  | 109 | 29.500 | 0.0228 | No |
| 2 | CAPG |  |  | 113 | 29.100 | 0.0519 | No |
| 3 | ERCC1 |  |  | 114 | 29.000 | 0.0812 | No |
| 4 | FADS1 |  |  | 292 | 21.900 | 0.0919 | No |
| 5 | NEDD4 |  |  | 363 | 20.500 | 0.1081 | No |
| 6 | TIMP1 |  |  | 430 | 19.400 | 0.1235 | No |
| 7 | MAN1B1 |  |  | 446 | 19.200 | 0.1419 | No |
| 8 | RHOBTB3 |  |  | 517 | 18.300 | 0.1558 | No |
| 9 | ANXA1 |  |  | 697 | 16.400 | 0.1609 | No |
| 10 | SPG20 |  |  | 1007 | 14.200 | 0.1553 | No |
| 11 | EIF4EBP1 |  |  | 1218 | 13.000 | 0.1549 | No |
| 12 | CDC37 |  |  | 1343 | 12.300 | 0.1593 | No |
| 13 | OGT |  |  | 1367 | 12.200 | 0.1701 | No |
| 14 | PARVB |  |  | 1437 | 11.900 | 0.1777 | No |
| 15 | ATF5 |  |  | 1492 | 11.700 | 0.1860 | No |
| 16 | DIAPH1 |  |  | 1742 | 10.600 | 0.1807 | No |
| 17 | NUMA1 |  |  | 1784 | 10.500 | 0.1887 | No |
| 18 | PPP1R15A |  |  | 1809 | 10.400 | 0.1976 | No |
| 19 | YES1 |  |  | 1851 | 10.300 | 0.2054 | No |
| 20 | ARHGDIA |  |  | 1887 | 10.100 | 0.2133 | No |
| 21 | CDKN1A |  |  | 2165 | 9.100 | 0.2046 | No |
| 22 | OGFR |  |  | 2192 | 9.100 | 0.2122 | No |
| 23 | SFTPB |  |  | 2395 | 8.500 | 0.2077 | No |
| 24 | PEA15 |  |  | 2542 | 8.100 | 0.2065 | No |
| 25 | SF3A2 |  |  | 2787 | 7.400 | 0.1982 | No |
| 26 | TPST2 |  |  | 2812 | 7.400 | 0.2042 | No |
| 27 | RAPGEF1 |  |  | 2855 | 7.300 | 0.2088 | No |
| 28 | PON2 |  |  | 2864 | 7.200 | 0.2156 | No |
| 29 | IFI44 |  |  | 2871 | 7.200 | 0.2225 | No |
| 30 | LAMA5 |  |  | 2954 | 7.000 | 0.2242 | No |
| 31 | THBS1 |  |  | 2977 | 7.000 | 0.2299 | No |
| 32 | SERPINF1 |  |  | 2990 | 6.900 | 0.2361 | No |
| 33 | TBC1D1 |  |  | 3167 | 6.500 | 0.2313 | No |
| 34 | NRG1 |  |  | 3185 | 6.500 | 0.2368 | No |
| 35 | ATP11A |  |  | 3273 | 6.300 | 0.2375 | No |
| 36 | TGFBR3 |  |  | 3297 | 6.200 | 0.2423 | No |
| 37 | MAPKAPK2 |  |  | 3345 | 6.100 | 0.2454 | No |
| 38 | RNH1 |  |  | 3404 | 6.000 | 0.2477 | No |
| 39 | PRKCA |  |  | 3484 | 5.800 | 0.2485 | No |
| 40 | MAD1L1 |  |  | 3575 | 5.600 | 0.2484 | No |
| 41 | IGF1R |  |  | 3776 | 5.200 | 0.2407 | No |
| 42 | DUSP6 |  |  | 3922 | 4.900 | 0.2363 | No |
| 43 | S100A13 |  |  | 3956 | 4.900 | 0.2391 | No |
| 44 | GALNT2 |  |  | 4049 | 4.700 | 0.2380 | No |
| 45 | CD9 |  |  | 4086 | 4.600 | 0.2403 | No |
| 46 | MYO9B |  |  | 4127 | 4.600 | 0.2424 | No |
| 47 | PATZ1 |  |  | 4154 | 4.500 | 0.2452 | No |
| 48 | RRBP1 |  |  | 4243 | 4.400 | 0.2440 | No |
| 49 | ARHGEF1 |  |  | 4313 | 4.200 | 0.2438 | No |
| 50 | MAST4 |  |  | 4444 | 4.000 | 0.2394 | No |
| 51 | SLC16A3 |  |  | 4681 | 3.600 | 0.2279 | No |
| 52 | TLE1 |  |  | 4769 | 3.500 | 0.2258 | No |
| 53 | IL6R |  |  | 4909 | 3.200 | 0.2201 | No |
| 54 | TNFRSF1B |  |  | 5066 | 2.900 | 0.2129 | No |
| 55 | ARID5A |  |  | 5186 | 2.700 | 0.2080 | No |
| 56 | H1FX |  |  | 5195 | 2.700 | 0.2102 | No |
| 57 | ACVR2A |  |  | 5259 | 2.600 | 0.2088 | No |
| 58 | THNSL1 |  |  | 5560 | 2.200 | 0.1917 | No |
| 59 | B3GALNT1 |  |  | 5693 | 2.000 | 0.1852 | No |
| 60 | CLCN5 |  |  | 5833 | 1.800 | 0.1780 | No |
| 61 | GRINA |  |  | 5854 | 1.800 | 0.1786 | No |
| 62 | ANKRD55 |  |  | 5978 | 1.600 | 0.1722 | No |
| 63 | GGA3 |  |  | 6076 | 1.500 | 0.1675 | No |
| 64 | DNAJB2 |  |  | 6159 | 1.400 | 0.1636 | No |
| 65 | COMMD4 |  |  | 6426 | 1.000 | 0.1475 | No |
| 66 | TSPO |  |  | 6428 | 1.000 | 0.1484 | No |
| 67 | CBX4 |  |  | 6436 | 1.000 | 0.1490 | No |
| 68 | RNF41 |  |  | 6496 | 0.900 | 0.1461 | No |
| 69 | PAK1 |  |  | 6510 | 0.900 | 0.1462 | No |
| 70 | ADAMTS6 |  |  | 6544 | 0.900 | 0.1450 | No |
| 71 | PHEX |  |  | 6592 | 0.800 | 0.1427 | No |
| 72 | CABIN1 |  |  | 6593 | 0.800 | 0.1435 | No |
| 73 | KLF10 |  |  | 6616 | 0.800 | 0.1429 | No |
| 74 | SPAG4 |  |  | 6633 | 0.800 | 0.1427 | No |
| 75 | GRN |  |  | 6698 | 0.700 | 0.1393 | No |
| 76 | MX1 |  |  | 6780 | 0.600 | 0.1347 | No |
| 77 | KIAA0895 |  |  | 6803 | 0.600 | 0.1339 | No |
| 78 | DAK |  |  | 6916 | 0.500 | 0.1272 | No |
| 79 | CRY1 |  |  | 6963 | 0.400 | 0.1246 | No |
| 80 | ARMCX1 |  |  | 7033 | 0.400 | 0.1205 | No |
| 81 | SPRED2 |  |  | 7090 | 0.300 | 0.1172 | No |
| 82 | PDE3B |  |  | 7100 | 0.300 | 0.1170 | No |
| 83 | SERTAD3 |  |  | 7152 | 0.300 | 0.1140 | No |
| 84 | PLD1 |  |  | 7171 | 0.200 | 0.1130 | No |
| 85 | PRKCSH |  |  | 7236 | 0.200 | 0.1091 | No |
| 86 | MEST |  |  | 7253 | 0.200 | 0.1083 | No |
| 87 | ANGPT2 |  |  | 7409 | 0.100 | 0.0984 | No |
| 88 | HIST1H2AC |  |  | 7569 | 0.000 | 0.0881 | No |
| 89 | PSD3 |  |  | 7743 | 0.000 | 0.0770 | No |
| 90 | DUSP5 |  |  | 7861 | 0.000 | 0.0694 | No |
| 91 | SIX6 |  |  | 7970 | 0.000 | 0.0625 | No |
| 92 | EFHC2 |  |  | 7988 | 0.000 | 0.0614 | No |
| 93 | ERBB4 |  |  | 8246 | 0.000 | 0.0448 | No |
| 94 | L1TD1 |  |  | 8412 | 0.000 | 0.0342 | No |
| 95 | LDOC1 |  |  | 8425 | 0.000 | 0.0334 | No |
| 96 | OR2B2 |  |  | 8550 | 0.000 | 0.0254 | No |
| 97 | OR10H3 |  |  | 8677 | 0.000 | 0.0173 | No |
| 98 | AKR1D1 |  |  | 8954 | 0.000 | -0.0005 | No |
| 99 | SLC4A10 |  |  | 8972 | 0.000 | -0.0016 | No |
| 100 | ZDHHC11 |  |  | 9333 | 0.000 | -0.0248 | No |
| 101 | KLK2 |  |  | 9527 | 0.000 | -0.0372 | No |
| 102 | PNMA2 |  |  | 9858 | 0.000 | -0.0585 | No |
| 103 | ZSCAN18 |  |  | 9873 | 0.000 | -0.0594 | No |
| 104 | NCAN |  |  | 9993 | 0.000 | -0.0671 | No |
| 105 | NMB |  |  | 10045 | 0.000 | -0.0704 | No |
| 106 | RYR3 |  |  | 10047 | 0.000 | -0.0704 | No |
| 107 | CR2 |  |  | 10131 | -0.100 | -0.0757 | No |
| 108 | GABRR2 |  |  | 10290 | -0.100 | -0.0858 | No |
| 109 | PRLR |  |  | 10318 | -0.100 | -0.0874 | No |
| 110 | GATA3 |  |  | 10535 | -0.100 | -0.1012 | No |
| 111 | PIPOX |  |  | 10597 | -0.100 | -0.1050 | No |
| 112 | PIM2 |  |  | 10599 | -0.100 | -0.1050 | No |
| 113 | PRF1 |  |  | 10614 | -0.100 | -0.1058 | No |
| 114 | PPP2R2B |  |  | 10618 | -0.100 | -0.1059 | No |
| 115 | PLXNC1 |  |  | 10658 | -0.100 | -0.1083 | No |
| 116 | ZBTB48 |  |  | 10749 | -0.100 | -0.1140 | No |
| 117 | ADAMTS3 |  |  | 10865 | -0.200 | -0.1212 | No |
| 118 | CCR2 |  |  | 10919 | -0.200 | -0.1244 | No |
| 119 | XAB2 |  |  | 10960 | -0.200 | -0.1268 | No |
| 120 | TMEM45A |  |  | 11035 | -0.200 | -0.1314 | No |
| 121 | FAM89B |  |  | 11042 | -0.200 | -0.1316 | No |
| 122 | FRMD4B |  |  | 11103 | -0.200 | -0.1352 | No |
| 123 | DMD |  |  | 11128 | -0.200 | -0.1366 | No |
| 124 | IL16 |  |  | 11135 | -0.200 | -0.1368 | No |
| 125 | NPY2R |  |  | 11142 | -0.200 | -0.1369 | No |
| 126 | RBMS3 |  |  | 11232 | -0.300 | -0.1424 | No |
| 127 | NPTX1 |  |  | 11304 | -0.300 | -0.1467 | No |
| 128 | LILRB4 |  |  | 11307 | -0.300 | -0.1465 | No |
| 129 | CD6 |  |  | 11313 | -0.300 | -0.1465 | No |
| 130 | CD5 |  |  | 11386 | -0.300 | -0.1508 | No |
| 131 | CNR1 |  |  | 11468 | -0.400 | -0.1557 | No |
| 132 | ZAP70 |  |  | 11684 | -0.500 | -0.1690 | No |
| 133 | MAN2C1 |  |  | 11805 | -0.500 | -0.1762 | No |
| 134 | ITGA4 |  |  | 11836 | -0.600 | -0.1776 | No |
| 135 | MRPL4 |  |  | 11888 | -0.600 | -0.1802 | No |
| 136 | BLK |  |  | 11889 | -0.600 | -0.1796 | No |
| 137 | EHBP1L1 |  |  | 11925 | -0.600 | -0.1813 | No |
| 138 | IL17RB |  |  | 11963 | -0.600 | -0.1831 | No |
| 139 | LAG3 |  |  | 11985 | -0.600 | -0.1838 | No |
| 140 | SERPINE2 |  |  | 12212 | -0.800 | -0.1976 | No |
| 141 | VAMP2 |  |  | 12214 | -0.800 | -0.1968 | No |
| 142 | P2RX1 |  |  | 12305 | -0.900 | -0.2017 | No |
| 143 | PRSS3 |  |  | 12352 | -0.900 | -0.2038 | No |
| 144 | AKAP12 |  |  | 12445 | -1.000 | -0.2087 | No |
| 145 | PGLS |  |  | 12465 | -1.000 | -0.2089 | No |
| 146 | LTK |  |  | 12522 | -1.000 | -0.2115 | No |
| 147 | FGFR1 |  |  | 12555 | -1.000 | -0.2126 | No |
| 148 | HOMER2 |  |  | 12562 | -1.000 | -0.2119 | No |
| 149 | CXCR3 |  |  | 12696 | -1.100 | -0.2194 | No |
| 150 | CDK10 |  |  | 12699 | -1.100 | -0.2184 | No |
| 151 | MSRB2 |  |  | 12732 | -1.200 | -0.2193 | No |
| 152 | ITGAL |  |  | 12784 | -1.200 | -0.2214 | No |
| 153 | AEBP1 |  |  | 12850 | -1.300 | -0.2242 | No |
| 154 | MYB |  |  | 13065 | -1.500 | -0.2365 | No |
| 155 | SRPX |  |  | 13129 | -1.600 | -0.2390 | No |
| 156 | RFXANK |  |  | 13138 | -1.600 | -0.2379 | No |
| 157 | NCF2 |  |  | 13191 | -1.700 | -0.2395 | No |
| 158 | GYPC |  |  | 13224 | -1.700 | -0.2398 | No |
| 159 | RIN3 |  |  | 13325 | -1.800 | -0.2445 | No |
| 160 | ANKRD57 |  |  | 13332 | -1.800 | -0.2430 | No |
| 161 | IGHG1 |  |  | 13362 | -1.800 | -0.2431 | No |
| 162 | GATM |  |  | 13389 | -1.900 | -0.2428 | No |
| 163 | ATP5D |  |  | 13403 | -1.900 | -0.2418 | No |
| 164 | DPYSL2 |  |  | 13414 | -1.900 | -0.2405 | No |
| 165 | SLC25A1 |  |  | 13418 | -1.900 | -0.2388 | No |
| 166 | LPL |  |  | 13425 | -1.900 | -0.2372 | No |
| 167 | RXRA |  |  | 13495 | -2.000 | -0.2397 | No |
| 168 | ATP6V0C |  |  | 13612 | -2.200 | -0.2449 | No |
| 169 | ARL4D |  |  | 13642 | -2.200 | -0.2446 | No |
| 170 | XBP1 |  |  | 13685 | -2.300 | -0.2449 | No |
| 171 | TKT |  |  | 13763 | -2.400 | -0.2475 | No |
| 172 | ABR |  |  | 13812 | -2.500 | -0.2481 | No |
| 173 | WNT5A |  |  | 13923 | -2.700 | -0.2524 | Yes |
| 174 | JUND |  |  | 13940 | -2.700 | -0.2507 | Yes |
| 175 | ST6GALNAC4 |  |  | 13970 | -2.700 | -0.2499 | Yes |
| 176 | IGSF3 |  |  | 13971 | -2.700 | -0.2471 | Yes |
| 177 | RFNG |  |  | 13974 | -2.700 | -0.2445 | Yes |
| 178 | MYO5C |  |  | 13983 | -2.700 | -0.2423 | Yes |
| 179 | NAGLU |  |  | 14005 | -2.800 | -0.2409 | Yes |
| 180 | CLPTM1 |  |  | 14014 | -2.800 | -0.2385 | Yes |
| 181 | CEBPD |  |  | 14041 | -2.900 | -0.2373 | Yes |
| 182 | GAA |  |  | 14061 | -2.900 | -0.2356 | Yes |
| 183 | POLM |  |  | 14130 | -3.000 | -0.2369 | Yes |
| 184 | CHST12 |  |  | 14207 | -3.100 | -0.2387 | Yes |
| 185 | LDB1 |  |  | 14372 | -3.500 | -0.2458 | Yes |
| 186 | IRF7 |  |  | 14385 | -3.500 | -0.2430 | Yes |
| 187 | TYROBP |  |  | 14426 | -3.700 | -0.2418 | Yes |
| 188 | TM7SF2 |  |  | 14430 | -3.700 | -0.2383 | Yes |
| 189 | MEF2D |  |  | 14498 | -3.800 | -0.2388 | Yes |
| 190 | PXN |  |  | 14509 | -3.800 | -0.2356 | Yes |
| 191 | DNM2 |  |  | 14533 | -3.900 | -0.2331 | Yes |
| 192 | FA2H |  |  | 14570 | -4.000 | -0.2314 | Yes |
| 193 | NEU1 |  |  | 14592 | -4.100 | -0.2286 | Yes |
| 194 | CD38 |  |  | 14617 | -4.100 | -0.2260 | Yes |
| 195 | DAPK3 |  |  | 14618 | -4.100 | -0.2219 | Yes |
| 196 | CEACAM1 |  |  | 14627 | -4.100 | -0.2183 | Yes |
| 197 | HGS |  |  | 14659 | -4.200 | -0.2160 | Yes |
| 198 | TRIM2 |  |  | 14677 | -4.300 | -0.2128 | Yes |
| 199 | CPT1A |  |  | 14690 | -4.300 | -0.2092 | Yes |
| 200 | ADRBK1 |  |  | 14708 | -4.400 | -0.2059 | Yes |
| 201 | HS3ST1 |  |  | 14743 | -4.400 | -0.2036 | Yes |
| 202 | SH3TC1 |  |  | 14785 | -4.500 | -0.2017 | Yes |
| 203 | TIMP3 |  |  | 14808 | -4.600 | -0.1985 | Yes |
| 204 | PTPRCAP |  |  | 14849 | -4.700 | -0.1963 | Yes |
| 205 | PPAP2B |  |  | 14867 | -4.800 | -0.1926 | Yes |
| 206 | HLA-G |  |  | 14882 | -4.800 | -0.1886 | Yes |
| 207 | BTG2 |  |  | 14992 | -5.200 | -0.1904 | Yes |
| 208 | NUCB1 |  |  | 15024 | -5.300 | -0.1870 | Yes |
| 209 | CNKSR1 |  |  | 15069 | -5.500 | -0.1843 | Yes |
| 210 | GLUL |  |  | 15078 | -5.500 | -0.1793 | Yes |
| 211 | ROGDI |  |  | 15096 | -5.600 | -0.1747 | Yes |
| 212 | OGDH |  |  | 15098 | -5.700 | -0.1690 | Yes |
| 213 | MGAT4B |  |  | 15102 | -5.700 | -0.1635 | Yes |
| 214 | EGLN3 |  |  | 15149 | -5.900 | -0.1605 | Yes |
| 215 | FZR1 |  |  | 15155 | -6.000 | -0.1547 | Yes |
| 216 | RHOB |  |  | 15194 | -6.100 | -0.1510 | Yes |
| 217 | CTSF |  |  | 15223 | -6.300 | -0.1465 | Yes |
| 218 | FCER1G |  |  | 15228 | -6.300 | -0.1404 | Yes |
| 219 | MYO15B |  |  | 15276 | -6.600 | -0.1367 | Yes |
| 220 | PSD4 |  |  | 15283 | -6.600 | -0.1305 | Yes |
| 221 | HPCAL1 |  |  | 15307 | -6.800 | -0.1251 | Yes |
| 222 | GM2A |  |  | 15342 | -7.000 | -0.1202 | Yes |
| 223 | FZD1 |  |  | 15355 | -7.100 | -0.1138 | Yes |
| 224 | SH3GLB2 |  |  | 15361 | -7.200 | -0.1069 | Yes |
| 225 | COPE |  |  | 15375 | -7.300 | -0.1003 | Yes |
| 226 | MAN2B1 |  |  | 15440 | -7.900 | -0.0965 | Yes |
| 227 | ZYX |  |  | 15444 | -7.900 | -0.0887 | Yes |
| 228 | RHOT2 |  |  | 15513 | -8.700 | -0.0843 | Yes |
| 229 | PTPN18 |  |  | 15526 | -8.900 | -0.0761 | Yes |
| 230 | CRIP1 |  |  | 15534 | -9.000 | -0.0674 | Yes |
| 231 | RNASE4 |  |  | 15639 | -11.600 | -0.0624 | Yes |
| 232 | PBXIP1 |  |  | 15642 | -11.600 | -0.0508 | Yes |
| 233 | UQCRC1 |  |  | 15725 | -17.800 | -0.0382 | Yes |
| 234 | STXBP2 |  |  | 15727 | -18.300 | -0.0197 | Yes |
| 235 | ATP2A3 |  |  | 15735 | -20.900 | 0.0009 | Yes |
Table: GSEA details [plain text format]

  

Fig 2: HUTTMANN\_B\_CLL\_POOR\_SURVIVAL\_UP: Random ES distribution      
 Gene set null distribution of ES for **HUTTMANN\_B\_CLL\_POOR\_SURVIVAL\_UP**

  
