# Supplementary material for: Immunomic, genomic and transcriptomic characterization of CT26 colorectal carcinoma
Source: BMC Genomics. 2014 Mar 13;15(1):190. doi: 10.1186/1471-2164-15-190 (PMC4007559; doi:10.1186/1471-2164-15-190)
Supplement: Supplementary file 8 — Additional file 8: Contains the Gene Pattern gene set membership and enrichment values in an html format. The file index.html is the entry point. (ZIP 13 MB) [file 12864_2013_7028_MOESM8_ESM.zip › ISHIDA_E2F_TARGETS.html]

Details for gene set ISHIDA\_E2F\_TARGETS[GSEA]

|  || Dataset | CT26\_gene\_expression |
| Phenotype | NoPhenotypeAvailable |
| Upregulated in class | na\_pos |
| GeneSet | ISHIDA\_E2F\_TARGETS |
| Enrichment Score (ES) | 0.81235635 |
| Normalized Enrichment Score (NES) | 1.7161361 |
| Nominal p-value | 0.0 |
| FDR q-value | 0.0014625435 |
| FWER p-Value | 0.013 |
Table: GSEA Results Summary

  

Fig 1: Enrichment plot: ISHIDA\_E2F\_TARGETS      
 Profile of the Running ES Score & Positions of GeneSet Members on the Rank Ordered List

  

| PROBE | GENE SYMBOL | GENE\_TITLE | RANK IN GENE LIST | RANK METRIC SCORE | RUNNING ES | CORE ENRICHMENT || 1 | TOP2A |  |  | 8 | 56.000 | 0.0669 | Yes |
| 2 | LIG1 |  |  | 104 | 30.000 | 0.0970 | Yes |
| 3 | PRIM1 |  |  | 116 | 29.000 | 0.1312 | Yes |
| 4 | RPA1 |  |  | 120 | 28.700 | 0.1656 | Yes |
| 5 | RRM1 |  |  | 138 | 27.500 | 0.1976 | Yes |
| 6 | CCNA2 |  |  | 178 | 25.600 | 0.2259 | Yes |
| 7 | PRC1 |  |  | 181 | 25.500 | 0.2565 | Yes |
| 8 | PTTG1 |  |  | 223 | 23.900 | 0.2827 | Yes |
| 9 | CDC20 |  |  | 284 | 22.200 | 0.3056 | Yes |
| 10 | EZH2 |  |  | 286 | 22.000 | 0.3320 | Yes |
| 11 | MCM7 |  |  | 290 | 21.900 | 0.3582 | Yes |
| 12 | MCM3 |  |  | 315 | 21.400 | 0.3824 | Yes |
| 13 | KPNA2 |  |  | 354 | 20.700 | 0.4049 | Yes |
| 14 | CDCA7 |  |  | 356 | 20.700 | 0.4298 | Yes |
| 15 | BUB1 |  |  | 360 | 20.600 | 0.4544 | Yes |
| 16 | DBF4 |  |  | 369 | 20.400 | 0.4785 | Yes |
| 17 | ANLN |  |  | 374 | 20.400 | 0.5028 | Yes |
| 18 | SMC2 |  |  | 379 | 20.300 | 0.5270 | Yes |
| 19 | PCNA |  |  | 386 | 20.200 | 0.5509 | Yes |
| 20 | MKI67 |  |  | 414 | 19.700 | 0.5729 | Yes |
| 21 | RAD51 |  |  | 427 | 19.400 | 0.5955 | Yes |
| 22 | NCAPH |  |  | 459 | 19.000 | 0.6164 | Yes |
| 23 | DUT |  |  | 658 | 16.800 | 0.6240 | Yes |
| 24 | NUSAP1 |  |  | 716 | 16.200 | 0.6399 | Yes |
| 25 | SGOL1 |  |  | 857 | 15.100 | 0.6492 | Yes |
| 26 | NDC80 |  |  | 878 | 15.000 | 0.6660 | Yes |
| 27 | CCNE1 |  |  | 1031 | 14.000 | 0.6731 | Yes |
| 28 | FEN1 |  |  | 1052 | 13.900 | 0.6886 | Yes |
| 29 | RRM2 |  |  | 1084 | 13.700 | 0.7031 | Yes |
| 30 | CCNB2 |  |  | 1155 | 13.200 | 0.7146 | Yes |
| 31 | TRIP13 |  |  | 1182 | 13.100 | 0.7287 | Yes |
| 32 | E2F8 |  |  | 1210 | 13.000 | 0.7426 | Yes |
| 33 | HMGB2 |  |  | 1279 | 12.700 | 0.7536 | Yes |
| 34 | ASF1B |  |  | 1372 | 12.200 | 0.7624 | Yes |
| 35 | CDT1 |  |  | 1580 | 11.300 | 0.7628 | Yes |
| 36 | UBE2T |  |  | 1606 | 11.200 | 0.7747 | Yes |
| 37 | SLBP |  |  | 1951 | 9.900 | 0.7647 | Yes |
| 38 | CDKN2C |  |  | 1988 | 9.700 | 0.7741 | Yes |
| 39 | HMGB3 |  |  | 1996 | 9.700 | 0.7853 | Yes |
| 40 | STMN1 |  |  | 2088 | 9.400 | 0.7909 | Yes |
| 41 | RFC3 |  |  | 2115 | 9.300 | 0.8004 | Yes |
| 42 | LBR |  |  | 2246 | 8.900 | 0.8029 | Yes |
| 43 | CCNB1 |  |  | 2266 | 8.900 | 0.8124 | Yes |
| 44 | CDK2 |  |  | 2519 | 8.100 | 0.8061 | No |
| 45 | TK1 |  |  | 2762 | 7.500 | 0.7997 | No |
| 46 | AURKB |  |  | 2822 | 7.300 | 0.8047 | No |
| 47 | RB1 |  |  | 2925 | 7.100 | 0.8068 | No |
| 48 | TYMS |  |  | 3182 | 6.500 | 0.7983 | No |
| 49 | D4S234E |  |  | 13217 | -1.700 | 0.1613 | No |
Table: GSEA details [plain text format]

  

Fig 2: ISHIDA\_E2F\_TARGETS: Random ES distribution      
 Gene set null distribution of ES for **ISHIDA\_E2F\_TARGETS**

  
