# Supplementary material for: Immunomic, genomic and transcriptomic characterization of CT26 colorectal carcinoma
Source: BMC Genomics. 2014 Mar 13;15(1):190. doi: 10.1186/1471-2164-15-190 (PMC4007559; doi:10.1186/1471-2164-15-190)
Supplement: Supplementary file 8 — Additional file 8: Contains the Gene Pattern gene set membership and enrichment values in an html format. The file index.html is the entry point. (ZIP 13 MB) [file 12864_2013_7028_MOESM8_ESM.zip › JAEGER_METASTASIS_DN.html]

Details for gene set JAEGER\_METASTASIS\_DN[GSEA]

|  || Dataset | CT26\_gene\_expression |
| Phenotype | NoPhenotypeAvailable |
| Upregulated in class | na\_neg |
| GeneSet | JAEGER\_METASTASIS\_DN |
| Enrichment Score (ES) | -0.58051056 |
| Normalized Enrichment Score (NES) | NaN |
| Nominal p-value | NaN |
| FDR q-value | 1.0 |
| FWER p-Value | 0.0 |
Table: GSEA Results Summary

  

Fig 1: Enrichment plot: JAEGER\_METASTASIS\_DN      
 Profile of the Running ES Score & Positions of GeneSet Members on the Rank Ordered List

  

| PROBE | GENE SYMBOL | GENE\_TITLE | RANK IN GENE LIST | RANK METRIC SCORE | RUNNING ES | CORE ENRICHMENT || 1 | ASPA |  |  | 581 | 17.600 | -0.0159 | No |
| 2 | GJA1 |  |  | 670 | 16.700 | -0.0011 | No |
| 3 | AHNAK |  |  | 681 | 16.600 | 0.0185 | No |
| 4 | TM4SF1 |  |  | 1353 | 12.300 | -0.0097 | No |
| 5 | DST |  |  | 1611 | 11.200 | -0.0125 | No |
| 6 | PTGS1 |  |  | 1732 | 10.700 | -0.0071 | No |
| 7 | KLK10 |  |  | 1865 | 10.200 | -0.0032 | No |
| 8 | SLC1A4 |  |  | 1867 | 10.200 | 0.0092 | No |
| 9 | FST |  |  | 2073 | 9.400 | 0.0075 | No |
| 10 | PLP2 |  |  | 2217 | 9.000 | 0.0093 | No |
| 11 | SMARCA2 |  |  | 2614 | 7.900 | -0.0065 | No |
| 12 | BICD2 |  |  | 2826 | 7.300 | -0.0112 | No |
| 13 | PRDX2 |  |  | 3006 | 6.900 | -0.0143 | No |
| 14 | BTG1 |  |  | 3025 | 6.800 | -0.0071 | No |
| 15 | AIM1 |  |  | 3242 | 6.400 | -0.0132 | No |
| 16 | NFIB |  |  | 3497 | 5.800 | -0.0225 | No |
| 17 | CLCA2 |  |  | 3907 | 4.900 | -0.0428 | No |
| 18 | MAST4 |  |  | 4444 | 4.000 | -0.0725 | No |
| 19 | AKR1B10 |  |  | 4841 | 3.300 | -0.0939 | No |
| 20 | LAMC2 |  |  | 5100 | 2.900 | -0.1070 | No |
| 21 | CLTB |  |  | 5145 | 2.800 | -0.1064 | No |
| 22 | MID2 |  |  | 5216 | 2.700 | -0.1076 | No |
| 23 | PTCH1 |  |  | 5568 | 2.200 | -0.1276 | No |
| 24 | CTNND1 |  |  | 6009 | 1.600 | -0.1540 | No |
| 25 | PAK6 |  |  | 6070 | 1.500 | -0.1560 | No |
| 26 | NET1 |  |  | 6133 | 1.400 | -0.1583 | No |
| 27 | KRT10 |  |  | 6484 | 0.900 | -0.1797 | No |
| 28 | COL7A1 |  |  | 6939 | 0.500 | -0.2084 | No |
| 29 | NRCAM |  |  | 7112 | 0.300 | -0.2191 | No |
| 30 | CTNNBIP1 |  |  | 7127 | 0.300 | -0.2196 | No |
| 31 | PER2 |  |  | 7135 | 0.300 | -0.2197 | No |
| 32 | S100A8 |  |  | 7177 | 0.200 | -0.2221 | No |
| 33 | NAP1L2 |  |  | 7216 | 0.200 | -0.2243 | No |
| 34 | IMPA2 |  |  | 7264 | 0.200 | -0.2271 | No |
| 35 | DIO2 |  |  | 7370 | 0.100 | -0.2337 | No |
| 36 | HAL |  |  | 7406 | 0.100 | -0.2359 | No |
| 37 | S100A2 |  |  | 7596 | 0.000 | -0.2480 | No |
| 38 | TPSAB1 |  |  | 7658 | 0.000 | -0.2520 | No |
| 39 | HLF |  |  | 7689 | 0.000 | -0.2539 | No |
| 40 | KLK11 |  |  | 7878 | 0.000 | -0.2660 | No |
| 41 | FAT2 |  |  | 7924 | 0.000 | -0.2689 | No |
| 42 | TYRP1 |  |  | 7962 | 0.000 | -0.2713 | No |
| 43 | DSC1 |  |  | 7966 | 0.000 | -0.2715 | No |
| 44 | KRT6A |  |  | 8001 | 0.000 | -0.2737 | No |
| 45 | MCOLN3 |  |  | 8010 | 0.000 | -0.2742 | No |
| 46 | AKR1C2 |  |  | 8104 | 0.000 | -0.2802 | No |
| 47 | CALML5 |  |  | 8136 | 0.000 | -0.2822 | No |
| 48 | DEFB1 |  |  | 8210 | 0.000 | -0.2869 | No |
| 49 | DSG1 |  |  | 8230 | 0.000 | -0.2881 | No |
| 50 | FCER1A |  |  | 8255 | 0.000 | -0.2897 | No |
| 51 | FLG |  |  | 8261 | 0.000 | -0.2900 | No |
| 52 | GPR87 |  |  | 8311 | 0.000 | -0.2931 | No |
| 53 | IVL |  |  | 8359 | 0.000 | -0.2962 | No |
| 54 | KRT33A |  |  | 8375 | 0.000 | -0.2971 | No |
| 55 | NEBL |  |  | 8477 | 0.000 | -0.3036 | No |
| 56 | SERPINB4 |  |  | 8824 | 0.000 | -0.3259 | No |
| 57 | SERPINB3 |  |  | 8825 | 0.000 | -0.3259 | No |
| 58 | SPINK5 |  |  | 8855 | 0.000 | -0.3278 | No |
| 59 | SPRR1B |  |  | 8860 | 0.000 | -0.3281 | No |
| 60 | TFAP2C |  |  | 8896 | 0.000 | -0.3303 | No |
| 61 | TFAP2B |  |  | 9039 | 0.000 | -0.3395 | No |
| 62 | CPA3 |  |  | 9040 | 0.000 | -0.3395 | No |
| 63 | DSC3 |  |  | 9097 | 0.000 | -0.3431 | No |
| 64 | KLK7 |  |  | 9135 | 0.000 | -0.3455 | No |
| 65 | DSG3 |  |  | 9142 | 0.000 | -0.3459 | No |
| 66 | KRT31 |  |  | 9157 | 0.000 | -0.3468 | No |
| 67 | CALML3 |  |  | 9178 | 0.000 | -0.3480 | No |
| 68 | VSNL1 |  |  | 9386 | 0.000 | -0.3614 | No |
| 69 | CTSG |  |  | 9420 | 0.000 | -0.3635 | No |
| 70 | CSTA |  |  | 9457 | 0.000 | -0.3658 | No |
| 71 | SERPINB13 |  |  | 9604 | 0.000 | -0.3752 | No |
| 72 | DPP6 |  |  | 9634 | 0.000 | -0.3771 | No |
| 73 | PCSK2 |  |  | 9643 | 0.000 | -0.3776 | No |
| 74 | LTF |  |  | 9647 | 0.000 | -0.3778 | No |
| 75 | BBOX1 |  |  | 9679 | 0.000 | -0.3798 | No |
| 76 | LPPR4 |  |  | 9705 | 0.000 | -0.3814 | No |
| 77 | CFH |  |  | 9774 | 0.000 | -0.3858 | No |
| 78 | CD207 |  |  | 9831 | 0.000 | -0.3894 | No |
| 79 | TACSTD2 |  |  | 9909 | 0.000 | -0.3944 | No |
| 80 | IL1R2 |  |  | 9980 | 0.000 | -0.3989 | No |
| 81 | SOX15 |  |  | 10002 | 0.000 | -0.4002 | No |
| 82 | CST6 |  |  | 10005 | 0.000 | -0.4004 | No |
| 83 | FHOD3 |  |  | 10019 | 0.000 | -0.4012 | No |
| 84 | ABCA12 |  |  | 10054 | 0.000 | -0.4034 | No |
| 85 | LYPD3 |  |  | 10056 | 0.000 | -0.4035 | No |
| 86 | BCL11A |  |  | 10082 | 0.000 | -0.4051 | No |
| 87 | KRT1 |  |  | 10089 | 0.000 | -0.4055 | No |
| 88 | ARHGEF4 |  |  | 10187 | -0.100 | -0.4116 | No |
| 89 | S100A9 |  |  | 10244 | -0.100 | -0.4151 | No |
| 90 | KRT5 |  |  | 10251 | -0.100 | -0.4153 | No |
| 91 | COL17A1 |  |  | 10433 | -0.100 | -0.4269 | No |
| 92 | AQP3 |  |  | 10500 | -0.100 | -0.4310 | No |
| 93 | KRT16 |  |  | 10524 | -0.100 | -0.4324 | No |
| 94 | GATA3 |  |  | 10535 | -0.100 | -0.4329 | No |
| 95 | ANK3 |  |  | 10538 | -0.100 | -0.4329 | No |
| 96 | SCEL |  |  | 10579 | -0.100 | -0.4353 | No |
| 97 | KRT15 |  |  | 10616 | -0.100 | -0.4375 | No |
| 98 | PLXNC1 |  |  | 10658 | -0.100 | -0.4401 | No |
| 99 | LOR |  |  | 10699 | -0.100 | -0.4425 | No |
| 100 | GABRE |  |  | 10761 | -0.100 | -0.4463 | No |
| 101 | ZNF185 |  |  | 10928 | -0.200 | -0.4568 | No |
| 102 | MATN2 |  |  | 10934 | -0.200 | -0.4569 | No |
| 103 | TRIM29 |  |  | 10939 | -0.200 | -0.4569 | No |
| 104 | RORA |  |  | 10995 | -0.200 | -0.4602 | No |
| 105 | PALMD |  |  | 11012 | -0.200 | -0.4610 | No |
| 106 | TMEM45A |  |  | 11035 | -0.200 | -0.4621 | No |
| 107 | FZD10 |  |  | 11184 | -0.300 | -0.4713 | No |
| 108 | IRX4 |  |  | 11214 | -0.300 | -0.4728 | No |
| 109 | POU2F3 |  |  | 11219 | -0.300 | -0.4727 | No |
| 110 | DUOX1 |  |  | 11260 | -0.300 | -0.4749 | No |
| 111 | ITPR3 |  |  | 11263 | -0.300 | -0.4747 | No |
| 112 | COL4A6 |  |  | 11306 | -0.300 | -0.4770 | No |
| 113 | KRT23 |  |  | 11398 | -0.300 | -0.4825 | No |
| 114 | NTRK2 |  |  | 11470 | -0.400 | -0.4866 | No |
| 115 | CYP26B1 |  |  | 11524 | -0.400 | -0.4895 | No |
| 116 | KLK8 |  |  | 11528 | -0.400 | -0.4892 | No |
| 117 | LGR5 |  |  | 11535 | -0.400 | -0.4891 | No |
| 118 | SCUBE2 |  |  | 11553 | -0.400 | -0.4897 | No |
| 119 | KRT14 |  |  | 11559 | -0.400 | -0.4896 | No |
| 120 | ABHD2 |  |  | 11594 | -0.400 | -0.4913 | No |
| 121 | ADCY2 |  |  | 11597 | -0.400 | -0.4909 | No |
| 122 | PTPRZ1 |  |  | 11664 | -0.500 | -0.4945 | No |
| 123 | LTB4R |  |  | 11764 | -0.500 | -0.5003 | No |
| 124 | TPSB2 |  |  | 11850 | -0.600 | -0.5050 | No |
| 125 | CDH3 |  |  | 11996 | -0.600 | -0.5137 | No |
| 126 | PPL |  |  | 12065 | -0.700 | -0.5172 | No |
| 127 | GNA15 |  |  | 12242 | -0.800 | -0.5275 | No |
| 128 | EXPH5 |  |  | 12260 | -0.800 | -0.5277 | No |
| 129 | COBL |  |  | 12291 | -0.800 | -0.5286 | No |
| 130 | PRSS3 |  |  | 12352 | -0.900 | -0.5314 | No |
| 131 | LAMA3 |  |  | 12360 | -0.900 | -0.5307 | No |
| 132 | ENPP2 |  |  | 12393 | -0.900 | -0.5317 | No |
| 133 | AREG |  |  | 12421 | -0.900 | -0.5323 | No |
| 134 | ITM2A |  |  | 12432 | -0.900 | -0.5319 | No |
| 135 | KCND3 |  |  | 12532 | -1.000 | -0.5370 | No |
| 136 | CHN2 |  |  | 12584 | -1.000 | -0.5391 | No |
| 137 | DUSP7 |  |  | 12616 | -1.100 | -0.5397 | No |
| 138 | SERPINB5 |  |  | 12715 | -1.200 | -0.5446 | No |
| 139 | TPD52L1 |  |  | 12800 | -1.200 | -0.5485 | No |
| 140 | MAL |  |  | 12884 | -1.300 | -0.5523 | No |
| 141 | JAG1 |  |  | 12886 | -1.300 | -0.5508 | No |
| 142 | QPCT |  |  | 12946 | -1.400 | -0.5528 | No |
| 143 | SRD5A1 |  |  | 12971 | -1.400 | -0.5527 | No |
| 144 | ZNF750 |  |  | 13024 | -1.500 | -0.5542 | No |
| 145 | DNASE1L3 |  |  | 13041 | -1.500 | -0.5534 | No |
| 146 | NMU |  |  | 13073 | -1.500 | -0.5536 | No |
| 147 | ASS1 |  |  | 13085 | -1.500 | -0.5524 | No |
| 148 | ANKRD57 |  |  | 13332 | -1.800 | -0.5661 | No |
| 149 | CCL27 |  |  | 13347 | -1.800 | -0.5648 | No |
| 150 | CLIC3 |  |  | 13379 | -1.900 | -0.5644 | No |
| 151 | EPHB6 |  |  | 13427 | -1.900 | -0.5652 | No |
| 152 | SLPI |  |  | 13439 | -1.900 | -0.5635 | No |
| 153 | BCL11B |  |  | 13468 | -2.000 | -0.5629 | No |
| 154 | MAF |  |  | 13580 | -2.100 | -0.5675 | No |
| 155 | EGFR |  |  | 13693 | -2.300 | -0.5719 | No |
| 156 | FGFR2 |  |  | 13828 | -2.500 | -0.5775 | Yes |
| 157 | CCRL1 |  |  | 13834 | -2.500 | -0.5747 | Yes |
| 158 | MMP28 |  |  | 13875 | -2.600 | -0.5741 | Yes |
| 159 | CA12 |  |  | 13912 | -2.700 | -0.5731 | Yes |
| 160 | WNT5A |  |  | 13923 | -2.700 | -0.5705 | Yes |
| 161 | EFS |  |  | 14060 | -2.900 | -0.5757 | Yes |
| 162 | GRHL2 |  |  | 14075 | -2.900 | -0.5730 | Yes |
| 163 | PERP |  |  | 14087 | -2.900 | -0.5702 | Yes |
| 164 | ABLIM1 |  |  | 14134 | -3.000 | -0.5695 | Yes |
| 165 | ALDH2 |  |  | 14145 | -3.000 | -0.5665 | Yes |
| 166 | MYO6 |  |  | 14195 | -3.100 | -0.5658 | Yes |
| 167 | CXADR |  |  | 14214 | -3.100 | -0.5632 | Yes |
| 168 | CDA |  |  | 14224 | -3.200 | -0.5599 | Yes |
| 169 | KLF4 |  |  | 14285 | -3.300 | -0.5597 | Yes |
| 170 | S100A14 |  |  | 14322 | -3.400 | -0.5578 | Yes |
| 171 | DSC2 |  |  | 14341 | -3.400 | -0.5548 | Yes |
| 172 | NLRX1 |  |  | 14437 | -3.700 | -0.5564 | Yes |
| 173 | SLC2A1 |  |  | 14452 | -3.700 | -0.5528 | Yes |
| 174 | SFN |  |  | 14484 | -3.800 | -0.5502 | Yes |
| 175 | ALDH3B2 |  |  | 14512 | -3.900 | -0.5471 | Yes |
| 176 | KRT17 |  |  | 14517 | -3.900 | -0.5426 | Yes |
| 177 | IRF6 |  |  | 14564 | -4.000 | -0.5407 | Yes |
| 178 | TUBA4A |  |  | 14611 | -4.100 | -0.5386 | Yes |
| 179 | EVPL |  |  | 14649 | -4.200 | -0.5359 | Yes |
| 180 | KLF5 |  |  | 14651 | -4.200 | -0.5308 | Yes |
| 181 | SPRR1A |  |  | 14825 | -4.700 | -0.5362 | Yes |
| 182 | PKP1 |  |  | 14887 | -4.800 | -0.5342 | Yes |
| 183 | KIT |  |  | 14956 | -5.100 | -0.5324 | Yes |
| 184 | CA2 |  |  | 14983 | -5.200 | -0.5277 | Yes |
| 185 | PRELP |  |  | 14985 | -5.200 | -0.5214 | Yes |
| 186 | BMP7 |  |  | 14994 | -5.200 | -0.5156 | Yes |
| 187 | CXCL14 |  |  | 15080 | -5.600 | -0.5142 | Yes |
| 188 | TUFT1 |  |  | 15100 | -5.700 | -0.5084 | Yes |
| 189 | ETS2 |  |  | 15108 | -5.700 | -0.5019 | Yes |
| 190 | DSP |  |  | 15126 | -5.800 | -0.4959 | Yes |
| 191 | FXYD3 |  |  | 15130 | -5.900 | -0.4889 | Yes |
| 192 | MAP7 |  |  | 15177 | -6.000 | -0.4845 | Yes |
| 193 | ECHDC2 |  |  | 15181 | -6.100 | -0.4772 | Yes |
| 194 | ITGB4 |  |  | 15227 | -6.300 | -0.4724 | Yes |
| 195 | KRT19 |  |  | 15311 | -6.800 | -0.4694 | Yes |
| 196 | PYCARD |  |  | 15333 | -7.000 | -0.4622 | Yes |
| 197 | SCNN1A |  |  | 15352 | -7.100 | -0.4547 | Yes |
| 198 | FGFBP1 |  |  | 15389 | -7.400 | -0.4480 | Yes |
| 199 | PRSS8 |  |  | 15441 | -7.900 | -0.4416 | Yes |
| 200 | F2RL1 |  |  | 15456 | -8.000 | -0.4327 | Yes |
| 201 | PKP3 |  |  | 15459 | -8.100 | -0.4229 | Yes |
| 202 | FCGBP |  |  | 15463 | -8.100 | -0.4132 | Yes |
| 203 | EHF |  |  | 15467 | -8.200 | -0.4034 | Yes |
| 204 | SDC1 |  |  | 15474 | -8.300 | -0.3936 | Yes |
| 205 | RAPGEFL1 |  |  | 15475 | -8.300 | -0.3834 | Yes |
| 206 | GJB3 |  |  | 15483 | -8.300 | -0.3737 | Yes |
| 207 | LY6D |  |  | 15505 | -8.600 | -0.3646 | Yes |
| 208 | ELMO3 |  |  | 15519 | -8.800 | -0.3546 | Yes |
| 209 | AP1G2 |  |  | 15531 | -9.000 | -0.3443 | Yes |
| 210 | STAP2 |  |  | 15544 | -9.200 | -0.3338 | Yes |
| 211 | GPX2 |  |  | 15551 | -9.300 | -0.3228 | Yes |
| 212 | SSH3 |  |  | 15564 | -9.600 | -0.3119 | Yes |
| 213 | MST1R |  |  | 15576 | -9.700 | -0.3007 | Yes |
| 214 | ABCC3 |  |  | 15580 | -9.800 | -0.2889 | Yes |
| 215 | CDS1 |  |  | 15590 | -10.000 | -0.2773 | Yes |
| 216 | EPPK1 |  |  | 15597 | -10.100 | -0.2653 | Yes |
| 217 | TXNIP |  |  | 15601 | -10.200 | -0.2530 | Yes |
| 218 | LSR |  |  | 15620 | -10.900 | -0.2408 | Yes |
| 219 | ST14 |  |  | 15630 | -11.300 | -0.2276 | Yes |
| 220 | SPINT2 |  |  | 15640 | -11.600 | -0.2139 | Yes |
| 221 | FGFR3 |  |  | 15646 | -11.800 | -0.1998 | Yes |
| 222 | DGKA |  |  | 15656 | -12.000 | -0.1857 | Yes |
| 223 | THBD |  |  | 15664 | -12.400 | -0.1710 | Yes |
| 224 | LAMB3 |  |  | 15671 | -12.700 | -0.1559 | Yes |
| 225 | JUP |  |  | 15672 | -12.700 | -0.1403 | Yes |
| 226 | LAD1 |  |  | 15673 | -12.900 | -0.1245 | Yes |
| 227 | MAPK13 |  |  | 15694 | -14.700 | -0.1078 | Yes |
| 228 | GSTT1 |  |  | 15714 | -16.500 | -0.0889 | Yes |
| 229 | CDH1 |  |  | 15723 | -17.500 | -0.0680 | Yes |
| 230 | PDZK1IP1 |  |  | 15737 | -22.300 | -0.0415 | Yes |
| 231 | RAB25 |  |  | 15748 | -34.500 | 0.0001 | Yes |
Table: GSEA details [plain text format]

  

Fig 2: JAEGER\_METASTASIS\_DN: Random ES distribution      
 Gene set null distribution of ES for **JAEGER\_METASTASIS\_DN**

  
