# Supplementary material for: Immunomic, genomic and transcriptomic characterization of CT26 colorectal carcinoma
Source: BMC Genomics. 2014 Mar 13;15(1):190. doi: 10.1186/1471-2164-15-190 (PMC4007559; doi:10.1186/1471-2164-15-190)
Supplement: Supplementary file 8 — Additional file 8: Contains the Gene Pattern gene set membership and enrichment values in an html format. The file index.html is the entry point. (ZIP 13 MB) [file 12864_2013_7028_MOESM8_ESM.zip › KAMMINGA_EZH2_TARGETS.html]

Details for gene set KAMMINGA\_EZH2\_TARGETS[GSEA]

|  || Dataset | CT26\_gene\_expression |
| Phenotype | NoPhenotypeAvailable |
| Upregulated in class | na\_pos |
| GeneSet | KAMMINGA\_EZH2\_TARGETS |
| Enrichment Score (ES) | 0.83192563 |
| Normalized Enrichment Score (NES) | 1.7557416 |
| Nominal p-value | 0.0 |
| FDR q-value | 0.0012006868 |
| FWER p-Value | 0.0040 |
Table: GSEA Results Summary

  

Fig 1: Enrichment plot: KAMMINGA\_EZH2\_TARGETS      
 Profile of the Running ES Score & Positions of GeneSet Members on the Rank Ordered List

  

| PROBE | GENE SYMBOL | GENE\_TITLE | RANK IN GENE LIST | RANK METRIC SCORE | RUNNING ES | CORE ENRICHMENT || 1 | SMC4 |  |  | 1 | 76.300 | 0.0985 | Yes |
| 2 | TOP2A |  |  | 8 | 56.000 | 0.1705 | Yes |
| 3 | MCM4 |  |  | 81 | 31.500 | 0.2066 | Yes |
| 4 | ECT2 |  |  | 100 | 30.500 | 0.2448 | Yes |
| 5 | PRIM1 |  |  | 116 | 29.000 | 0.2813 | Yes |
| 6 | RRM1 |  |  | 138 | 27.500 | 0.3155 | Yes |
| 7 | XPO1 |  |  | 148 | 27.000 | 0.3498 | Yes |
| 8 | PRC1 |  |  | 181 | 25.500 | 0.3807 | Yes |
| 9 | TFDP1 |  |  | 199 | 24.700 | 0.4116 | Yes |
| 10 | MCM3 |  |  | 315 | 21.400 | 0.4319 | Yes |
| 11 | NAP1L1 |  |  | 345 | 20.900 | 0.4570 | Yes |
| 12 | NEK2 |  |  | 346 | 20.900 | 0.4840 | Yes |
| 13 | KPNA2 |  |  | 354 | 20.700 | 0.5103 | Yes |
| 14 | CDCA7 |  |  | 356 | 20.700 | 0.5370 | Yes |
| 15 | DBF4 |  |  | 369 | 20.400 | 0.5626 | Yes |
| 16 | SMC2 |  |  | 379 | 20.300 | 0.5882 | Yes |
| 17 | PCNA |  |  | 386 | 20.200 | 0.6140 | Yes |
| 18 | TOPBP1 |  |  | 401 | 19.900 | 0.6388 | Yes |
| 19 | MKI67 |  |  | 414 | 19.700 | 0.6635 | Yes |
| 20 | MAD2L1 |  |  | 559 | 17.900 | 0.6774 | Yes |
| 21 | NUSAP1 |  |  | 716 | 16.200 | 0.6884 | Yes |
| 22 | RFC1 |  |  | 750 | 16.000 | 0.7070 | Yes |
| 23 | AURKA |  |  | 799 | 15.600 | 0.7241 | Yes |
| 24 | HAT1 |  |  | 852 | 15.200 | 0.7404 | Yes |
| 25 | TACC3 |  |  | 910 | 14.800 | 0.7559 | Yes |
| 26 | GMNN |  |  | 911 | 14.800 | 0.7750 | Yes |
| 27 | H2AFZ |  |  | 982 | 14.300 | 0.7890 | Yes |
| 28 | CCNB2 |  |  | 1155 | 13.200 | 0.7951 | Yes |
| 29 | MCM2 |  |  | 1281 | 12.700 | 0.8036 | Yes |
| 30 | EED |  |  | 1315 | 12.500 | 0.8176 | Yes |
| 31 | RPA3 |  |  | 1621 | 11.200 | 0.8127 | Yes |
| 32 | CDCA5 |  |  | 1813 | 10.400 | 0.8140 | Yes |
| 33 | RBL1 |  |  | 1840 | 10.300 | 0.8256 | Yes |
| 34 | CUL2 |  |  | 2005 | 9.600 | 0.8276 | Yes |
| 35 | SETDB1 |  |  | 2208 | 9.000 | 0.8264 | Yes |
| 36 | POLE2 |  |  | 2300 | 8.800 | 0.8319 | Yes |
| 37 | AURKB |  |  | 2822 | 7.300 | 0.8082 | No |
| 38 | AHCY |  |  | 3109 | 6.600 | 0.7985 | No |
| 39 | PPP1CC |  |  | 4111 | 4.600 | 0.7407 | No |
| 40 | DNTT |  |  | 7537 | 0.000 | 0.5227 | No |
Table: GSEA details [plain text format]

  

Fig 2: KAMMINGA\_EZH2\_TARGETS: Random ES distribution      
 Gene set null distribution of ES for **KAMMINGA\_EZH2\_TARGETS**

  
