# Supplementary material for: Immunomic, genomic and transcriptomic characterization of CT26 colorectal carcinoma
Source: BMC Genomics. 2014 Mar 13;15(1):190. doi: 10.1186/1471-2164-15-190 (PMC4007559; doi:10.1186/1471-2164-15-190)
Supplement: Supplementary file 8 — Additional file 8: Contains the Gene Pattern gene set membership and enrichment values in an html format. The file index.html is the entry point. (ZIP 13 MB) [file 12864_2013_7028_MOESM8_ESM.zip › KEGG_CYTOKINE_CYTOKINE_RECEPTOR_INTERACTION.html]

Details for gene set KEGG\_CYTOKINE\_CYTOKINE\_RECEPTOR\_INTERACTION[GSEA]

|  || Dataset | CT26\_gene\_expression |
| Phenotype | NoPhenotypeAvailable |
| Upregulated in class | na\_neg |
| GeneSet | KEGG\_CYTOKINE\_CYTOKINE\_RECEPTOR\_INTERACTION |
| Enrichment Score (ES) | -0.27474365 |
| Normalized Enrichment Score (NES) | NaN |
| Nominal p-value | NaN |
| FDR q-value | 1.0 |
| FWER p-Value | 0.0 |
Table: GSEA Results Summary

  

Fig 1: Enrichment plot: KEGG\_CYTOKINE\_CYTOKINE\_RECEPTOR\_INTERACTION      
 Profile of the Running ES Score & Positions of GeneSet Members on the Rank Ordered List

  

| PROBE | GENE SYMBOL | GENE\_TITLE | RANK IN GENE LIST | RANK METRIC SCORE | RUNNING ES | CORE ENRICHMENT || 1 | IL18RAP |  |  | 812 | 15.500 | -0.0182 | No |
| 2 | INHBA |  |  | 828 | 15.400 | 0.0146 | No |
| 3 | CSF1 |  |  | 891 | 14.900 | 0.0434 | No |
| 4 | VEGFA |  |  | 946 | 14.600 | 0.0720 | No |
| 5 | MET |  |  | 954 | 14.500 | 0.1034 | No |
| 6 | GHR |  |  | 1223 | 13.000 | 0.1147 | No |
| 7 | KITLG |  |  | 1485 | 11.700 | 0.1236 | No |
| 8 | IL6ST |  |  | 1801 | 10.400 | 0.1262 | No |
| 9 | IL1R1 |  |  | 2778 | 7.400 | 0.0796 | No |
| 10 | TNFRSF12A |  |  | 2839 | 7.300 | 0.0917 | No |
| 11 | TNFRSF10A |  |  | 2867 | 7.200 | 0.1058 | No |
| 12 | TNFSF9 |  |  | 3046 | 6.800 | 0.1093 | No |
| 13 | BMPR1A |  |  | 3060 | 6.800 | 0.1234 | No |
| 14 | ACVR1 |  |  | 3245 | 6.400 | 0.1256 | No |
| 15 | BMPR2 |  |  | 3553 | 5.700 | 0.1184 | No |
| 16 | LIF |  |  | 3573 | 5.600 | 0.1294 | No |
| 17 | EDA2R |  |  | 3738 | 5.300 | 0.1305 | No |
| 18 | IL7 |  |  | 3913 | 4.900 | 0.1301 | No |
| 19 | TGFBR1 |  |  | 4435 | 4.000 | 0.1053 | No |
| 20 | IFNAR1 |  |  | 4470 | 3.900 | 0.1117 | No |
| 21 | PDGFRB |  |  | 4522 | 3.800 | 0.1168 | No |
| 22 | IL6R |  |  | 4909 | 3.200 | 0.0989 | No |
| 23 | FIGF |  |  | 4938 | 3.200 | 0.1042 | No |
| 24 | TNFRSF9 |  |  | 5061 | 3.000 | 0.1029 | No |
| 25 | TNFRSF1B |  |  | 5066 | 2.900 | 0.1090 | No |
| 26 | ACVR2A |  |  | 5259 | 2.600 | 0.1024 | No |
| 27 | PDGFC |  |  | 5655 | 2.100 | 0.0815 | No |
| 28 | IL1RAP |  |  | 5660 | 2.100 | 0.0859 | No |
| 29 | IL11 |  |  | 5768 | 1.900 | 0.0832 | No |
| 30 | CCL25 |  |  | 5788 | 1.900 | 0.0861 | No |
| 31 | IL12RB1 |  |  | 5812 | 1.900 | 0.0888 | No |
| 32 | IL2RG |  |  | 5893 | 1.800 | 0.0876 | No |
| 33 | CLCF1 |  |  | 6024 | 1.600 | 0.0828 | No |
| 34 | VEGFB |  |  | 6031 | 1.600 | 0.0859 | No |
| 35 | CNTF |  |  | 6377 | 1.100 | 0.0661 | No |
| 36 | CXCL2 |  |  | 6388 | 1.100 | 0.0679 | No |
| 37 | TGFB3 |  |  | 6394 | 1.100 | 0.0699 | No |
| 38 | CCR1 |  |  | 6492 | 0.900 | 0.0657 | No |
| 39 | TGFB1 |  |  | 6532 | 0.900 | 0.0651 | No |
| 40 | PRL |  |  | 6706 | 0.700 | 0.0555 | No |
| 41 | IL15 |  |  | 7069 | 0.300 | 0.0329 | No |
| 42 | CXCL10 |  |  | 7089 | 0.300 | 0.0323 | No |
| 43 | PPBP |  |  | 7319 | 0.100 | 0.0178 | No |
| 44 | TNFSF12 |  |  | 7384 | 0.100 | 0.0139 | No |
| 45 | IL1A |  |  | 7413 | 0.100 | 0.0123 | No |
| 46 | IL12RB2 |  |  | 7425 | 0.100 | 0.0118 | No |
| 47 | IL23A |  |  | 7435 | 0.100 | 0.0115 | No |
| 48 | FAS |  |  | 7445 | 0.100 | 0.0111 | No |
| 49 | TNFRSF17 |  |  | 7446 | 0.100 | 0.0113 | No |
| 50 | TNFRSF1A |  |  | 7454 | 0.100 | 0.0111 | No |
| 51 | IL24 |  |  | 7459 | 0.100 | 0.0110 | No |
| 52 | IL6 |  |  | 7487 | 0.100 | 0.0095 | No |
| 53 | CCL17 |  |  | 7491 | 0.100 | 0.0096 | No |
| 54 | IL2 |  |  | 7503 | 0.100 | 0.0091 | No |
| 55 | CSF2 |  |  | 7540 | 0.000 | 0.0067 | No |
| 56 | IFNA7 |  |  | 7576 | 0.000 | 0.0045 | No |
| 57 | EGF |  |  | 7646 | 0.000 | 0.0000 | No |
| 58 | PDGFB |  |  | 7657 | 0.000 | -0.0006 | No |
| 59 | IL19 |  |  | 7666 | 0.000 | -0.0011 | No |
| 60 | TNFSF18 |  |  | 7677 | 0.000 | -0.0018 | No |
| 61 | CSF3 |  |  | 7703 | 0.000 | -0.0034 | No |
| 62 | INHBE |  |  | 7999 | 0.000 | -0.0224 | No |
| 63 | IL18R1 |  |  | 8018 | 0.000 | -0.0235 | No |
| 64 | BMPR1B |  |  | 8027 | 0.000 | -0.0240 | No |
| 65 | CCL26 |  |  | 8144 | 0.000 | -0.0315 | No |
| 66 | CD70 |  |  | 8147 | 0.000 | -0.0316 | No |
| 67 | IFNA16 |  |  | 8339 | 0.000 | -0.0439 | No |
| 68 | IFNA4 |  |  | 8340 | 0.000 | -0.0439 | No |
| 69 | IFNA5 |  |  | 8341 | 0.000 | -0.0439 | No |
| 70 | IFNB1 |  |  | 8342 | 0.000 | -0.0439 | No |
| 71 | IFNG |  |  | 8343 | 0.000 | -0.0439 | No |
| 72 | IL17A |  |  | 8344 | 0.000 | -0.0439 | No |
| 73 | IL20 |  |  | 8349 | 0.000 | -0.0442 | No |
| 74 | IL21 |  |  | 8350 | 0.000 | -0.0442 | No |
| 75 | IL22 |  |  | 8351 | 0.000 | -0.0442 | No |
| 76 | IL28A |  |  | 8352 | 0.000 | -0.0442 | No |
| 77 | IL3 |  |  | 8353 | 0.000 | -0.0442 | No |
| 78 | IL4 |  |  | 8355 | 0.000 | -0.0443 | No |
| 79 | EPO |  |  | 8957 | 0.000 | -0.0830 | No |
| 80 | CXCL6 |  |  | 8999 | 0.000 | -0.0856 | No |
| 81 | IL13 |  |  | 9074 | 0.000 | -0.0904 | No |
| 82 | TNFSF4 |  |  | 9152 | 0.000 | -0.0953 | No |
| 83 | IL5RA |  |  | 9192 | 0.000 | -0.0979 | No |
| 84 | IL23R |  |  | 9228 | 0.000 | -0.1001 | No |
| 85 | IL5 |  |  | 9293 | 0.000 | -0.1042 | No |
| 86 | INHBC |  |  | 9327 | 0.000 | -0.1064 | No |
| 87 | TNFSF8 |  |  | 9396 | 0.000 | -0.1107 | No |
| 88 | CCL1 |  |  | 9434 | 0.000 | -0.1131 | No |
| 89 | IL9 |  |  | 9455 | 0.000 | -0.1144 | No |
| 90 | IL20RA |  |  | 9465 | 0.000 | -0.1150 | No |
| 91 | IL12B |  |  | 9528 | 0.000 | -0.1190 | No |
| 92 | IFNK |  |  | 9548 | 0.000 | -0.1202 | No |
| 93 | TPO |  |  | 9550 | 0.000 | -0.1203 | No |
| 94 | MPL |  |  | 9581 | 0.000 | -0.1222 | No |
| 95 | CCR3 |  |  | 9606 | 0.000 | -0.1237 | No |
| 96 | FASLG |  |  | 9611 | 0.000 | -0.1240 | No |
| 97 | IL2RA |  |  | 9614 | 0.000 | -0.1241 | No |
| 98 | IL28B |  |  | 9818 | 0.000 | -0.1372 | No |
| 99 | CXCL3 |  |  | 9838 | 0.000 | -0.1384 | No |
| 100 | TNFRSF8 |  |  | 9866 | 0.000 | -0.1402 | No |
| 101 | GDF5 |  |  | 9878 | 0.000 | -0.1409 | No |
| 102 | IL7R |  |  | 9900 | 0.000 | -0.1422 | No |
| 103 | IL1B |  |  | 9952 | 0.000 | -0.1455 | No |
| 104 | CXCL11 |  |  | 9974 | 0.000 | -0.1469 | No |
| 105 | IL1R2 |  |  | 9980 | 0.000 | -0.1472 | No |
| 106 | HGF |  |  | 10039 | 0.000 | -0.1509 | No |
| 107 | IL10 |  |  | 10071 | 0.000 | -0.1529 | No |
| 108 | TSLP |  |  | 10103 | 0.000 | -0.1549 | No |
| 109 | CD40LG |  |  | 10183 | -0.100 | -0.1598 | No |
| 110 | IL17B |  |  | 10246 | -0.100 | -0.1636 | No |
| 111 | XCR1 |  |  | 10268 | -0.100 | -0.1647 | No |
| 112 | PRLR |  |  | 10318 | -0.100 | -0.1676 | No |
| 113 | CCL4 |  |  | 10361 | -0.100 | -0.1701 | No |
| 114 | EDA |  |  | 10408 | -0.100 | -0.1729 | No |
| 115 | CCR8 |  |  | 10423 | -0.100 | -0.1735 | No |
| 116 | OSM |  |  | 10430 | -0.100 | -0.1737 | No |
| 117 | CCR9 |  |  | 10533 | -0.100 | -0.1801 | No |
| 118 | CXCL9 |  |  | 10552 | -0.100 | -0.1810 | No |
| 119 | TNFSF15 |  |  | 10559 | -0.100 | -0.1812 | No |
| 120 | IL12A |  |  | 10588 | -0.100 | -0.1827 | No |
| 121 | TNFSF14 |  |  | 10622 | -0.100 | -0.1847 | No |
| 122 | CCR4 |  |  | 10656 | -0.100 | -0.1866 | No |
| 123 | IFNAR2 |  |  | 10770 | -0.100 | -0.1936 | No |
| 124 | AMH |  |  | 10778 | -0.100 | -0.1938 | No |
| 125 | TNFSF11 |  |  | 10785 | -0.100 | -0.1940 | No |
| 126 | CCL24 |  |  | 10835 | -0.200 | -0.1967 | No |
| 127 | XCL1 |  |  | 10837 | -0.200 | -0.1964 | No |
| 128 | TNFRSF11B |  |  | 10866 | -0.200 | -0.1977 | No |
| 129 | FLT3 |  |  | 10873 | -0.200 | -0.1977 | No |
| 130 | OSMR |  |  | 10917 | -0.200 | -0.2000 | No |
| 131 | CCR2 |  |  | 10919 | -0.200 | -0.1996 | No |
| 132 | EDAR |  |  | 10936 | -0.200 | -0.2002 | No |
| 133 | IL17RA |  |  | 10980 | -0.200 | -0.2025 | No |
| 134 | CCL7 |  |  | 11021 | -0.200 | -0.2047 | No |
| 135 | IL25 |  |  | 11026 | -0.200 | -0.2045 | No |
| 136 | CXCR6 |  |  | 11100 | -0.200 | -0.2088 | No |
| 137 | CCL2 |  |  | 11114 | -0.200 | -0.2092 | No |
| 138 | IL22RA2 |  |  | 11202 | -0.300 | -0.2141 | No |
| 139 | LIFR |  |  | 11290 | -0.300 | -0.2190 | No |
| 140 | INHBB |  |  | 11390 | -0.300 | -0.2248 | No |
| 141 | LEPR |  |  | 11393 | -0.300 | -0.2242 | No |
| 142 | CCL19 |  |  | 11443 | -0.300 | -0.2267 | No |
| 143 | LEP |  |  | 11569 | -0.400 | -0.2339 | No |
| 144 | TNF |  |  | 11574 | -0.400 | -0.2333 | No |
| 145 | IL9R |  |  | 11614 | -0.400 | -0.2349 | No |
| 146 | CSF3R |  |  | 11640 | -0.400 | -0.2356 | No |
| 147 | CCR6 |  |  | 11662 | -0.500 | -0.2359 | No |
| 148 | TGFB2 |  |  | 11795 | -0.500 | -0.2433 | No |
| 149 | CD27 |  |  | 11797 | -0.500 | -0.2423 | No |
| 150 | AMHR2 |  |  | 11826 | -0.500 | -0.2430 | No |
| 151 | CCL8 |  |  | 11845 | -0.600 | -0.2428 | No |
| 152 | TNFRSF13C |  |  | 11886 | -0.600 | -0.2441 | No |
| 153 | CNTFR |  |  | 11945 | -0.600 | -0.2465 | No |
| 154 | IL17RB |  |  | 11963 | -0.600 | -0.2463 | No |
| 155 | CCL13 |  |  | 11967 | -0.600 | -0.2451 | No |
| 156 | TNFSF10 |  |  | 12042 | -0.700 | -0.2484 | No |
| 157 | LTA |  |  | 12091 | -0.700 | -0.2499 | No |
| 158 | FLT1 |  |  | 12099 | -0.700 | -0.2488 | No |
| 159 | EPOR |  |  | 12120 | -0.700 | -0.2486 | No |
| 160 | CCL20 |  |  | 12138 | -0.700 | -0.2481 | No |
| 161 | CSF2RB |  |  | 12160 | -0.700 | -0.2480 | No |
| 162 | ACVR2B |  |  | 12164 | -0.700 | -0.2466 | No |
| 163 | CCL21 |  |  | 12271 | -0.800 | -0.2517 | No |
| 164 | CCL22 |  |  | 12279 | -0.800 | -0.2504 | No |
| 165 | CD40 |  |  | 12293 | -0.800 | -0.2495 | No |
| 166 | CXCL13 |  |  | 12321 | -0.900 | -0.2492 | No |
| 167 | CRLF2 |  |  | 12323 | -0.900 | -0.2473 | No |
| 168 | TNFRSF19 |  |  | 12338 | -0.900 | -0.2462 | No |
| 169 | IL15RA |  |  | 12590 | -1.100 | -0.2600 | No |
| 170 | TNFSF13B |  |  | 12622 | -1.100 | -0.2596 | No |
| 171 | TNFRSF4 |  |  | 12676 | -1.100 | -0.2606 | No |
| 172 | CXCR3 |  |  | 12696 | -1.100 | -0.2594 | No |
| 173 | KDR |  |  | 12697 | -1.100 | -0.2569 | No |
| 174 | CCR7 |  |  | 12772 | -1.200 | -0.2591 | No |
| 175 | IL10RA |  |  | 12837 | -1.300 | -0.2603 | No |
| 176 | IL2RB |  |  | 12898 | -1.300 | -0.2613 | No |
| 177 | CCL28 |  |  | 13098 | -1.600 | -0.2706 | Yes |
| 178 | TNFRSF25 |  |  | 13143 | -1.600 | -0.2700 | Yes |
| 179 | IL21R |  |  | 13181 | -1.700 | -0.2686 | Yes |
| 180 | VEGFC |  |  | 13182 | -1.700 | -0.2649 | Yes |
| 181 | IL10RB |  |  | 13277 | -1.700 | -0.2672 | Yes |
| 182 | BMP2 |  |  | 13318 | -1.800 | -0.2658 | Yes |
| 183 | CCL27 |  |  | 13347 | -1.800 | -0.2637 | Yes |
| 184 | PDGFRA |  |  | 13520 | -2.100 | -0.2701 | Yes |
| 185 | TNFRSF13B |  |  | 13554 | -2.100 | -0.2676 | Yes |
| 186 | CTF1 |  |  | 13565 | -2.100 | -0.2637 | Yes |
| 187 | CX3CR1 |  |  | 13586 | -2.100 | -0.2603 | Yes |
| 188 | CCL11 |  |  | 13654 | -2.200 | -0.2598 | Yes |
| 189 | EGFR |  |  | 13693 | -2.300 | -0.2572 | Yes |
| 190 | CCL5 |  |  | 13706 | -2.300 | -0.2529 | Yes |
| 191 | CCR10 |  |  | 13708 | -2.300 | -0.2479 | Yes |
| 192 | IL3RA |  |  | 13841 | -2.500 | -0.2510 | Yes |
| 193 | IL28RA |  |  | 13902 | -2.600 | -0.2491 | Yes |
| 194 | NGFR |  |  | 13977 | -2.700 | -0.2479 | Yes |
| 195 | TGFBR2 |  |  | 14001 | -2.800 | -0.2433 | Yes |
| 196 | TNFRSF18 |  |  | 14017 | -2.800 | -0.2381 | Yes |
| 197 | FLT4 |  |  | 14141 | -3.000 | -0.2394 | Yes |
| 198 | CXCL12 |  |  | 14228 | -3.200 | -0.2379 | Yes |
| 199 | CSF1R |  |  | 14344 | -3.400 | -0.2379 | Yes |
| 200 | IL4R |  |  | 14354 | -3.500 | -0.2307 | Yes |
| 201 | CXCR4 |  |  | 14374 | -3.500 | -0.2243 | Yes |
| 202 | PF4 |  |  | 14433 | -3.700 | -0.2199 | Yes |
| 203 | IL13RA1 |  |  | 14451 | -3.700 | -0.2129 | Yes |
| 204 | FLT3LG |  |  | 14527 | -3.900 | -0.2091 | Yes |
| 205 | TNFSF13 |  |  | 14579 | -4.000 | -0.2036 | Yes |
| 206 | LTBR |  |  | 14581 | -4.000 | -0.1949 | Yes |
| 207 | IFNGR1 |  |  | 14640 | -4.200 | -0.1894 | Yes |
| 208 | ACVRL1 |  |  | 14643 | -4.200 | -0.1803 | Yes |
| 209 | LTB |  |  | 14656 | -4.200 | -0.1718 | Yes |
| 210 | CXCL16 |  |  | 14924 | -4.900 | -0.1783 | Yes |
| 211 | ACVR1B |  |  | 14954 | -5.000 | -0.1691 | Yes |
| 212 | KIT |  |  | 14956 | -5.100 | -0.1580 | Yes |
| 213 | IL22RA1 |  |  | 14963 | -5.100 | -0.1472 | Yes |
| 214 | BMP7 |  |  | 14994 | -5.200 | -0.1377 | Yes |
| 215 | CXCL14 |  |  | 15080 | -5.600 | -0.1309 | Yes |
| 216 | CX3CL1 |  |  | 15197 | -6.200 | -0.1247 | Yes |
| 217 | TNFRSF14 |  |  | 15267 | -6.500 | -0.1149 | Yes |
| 218 | TNFRSF21 |  |  | 15339 | -7.000 | -0.1041 | Yes |
| 219 | IL18 |  |  | 15349 | -7.100 | -0.0890 | Yes |
| 220 | PDGFA |  |  | 15406 | -7.500 | -0.0762 | Yes |
| 221 | IFNGR2 |  |  | 15487 | -8.400 | -0.0629 | Yes |
| 222 | IL11RA |  |  | 15582 | -9.800 | -0.0474 | Yes |
| 223 | CSF2RA |  |  | 15641 | -11.600 | -0.0256 | Yes |
| 224 | TNFRSF11A |  |  | 15696 | -14.800 | 0.0034 | Yes |
Table: GSEA details [plain text format]

  

Fig 2: KEGG\_CYTOKINE\_CYTOKINE\_RECEPTOR\_INTERACTION: Random ES distribution      
 Gene set null distribution of ES for **KEGG\_CYTOKINE\_CYTOKINE\_RECEPTOR\_INTERACTION**

  
