# Supplementary material for: Immunomic, genomic and transcriptomic characterization of CT26 colorectal carcinoma
Source: BMC Genomics. 2014 Mar 13;15(1):190. doi: 10.1186/1471-2164-15-190 (PMC4007559; doi:10.1186/1471-2164-15-190)
Supplement: Supplementary file 8 — Additional file 8: Contains the Gene Pattern gene set membership and enrichment values in an html format. The file index.html is the entry point. (ZIP 13 MB) [file 12864_2013_7028_MOESM8_ESM.zip › KEGG_LYSOSOME.html]

Details for gene set KEGG\_LYSOSOME[GSEA]

|  || Dataset | CT26\_gene\_expression |
| Phenotype | NoPhenotypeAvailable |
| Upregulated in class | na\_neg |
| GeneSet | KEGG\_LYSOSOME |
| Enrichment Score (ES) | -0.23221499 |
| Normalized Enrichment Score (NES) | NaN |
| Nominal p-value | NaN |
| FDR q-value | 1.0 |
| FWER p-Value | 0.0 |
Table: GSEA Results Summary

  

Fig 1: Enrichment plot: KEGG\_LYSOSOME      
 Profile of the Running ES Score & Positions of GeneSet Members on the Rank Ordered List

  

| PROBE | GENE SYMBOL | GENE\_TITLE | RANK IN GENE LIST | RANK METRIC SCORE | RUNNING ES | CORE ENRICHMENT || 1 | CTSL2 |  |  | 267 | 22.600 | 0.0267 | No |
| 2 | ASAH1 |  |  | 490 | 18.600 | 0.0485 | No |
| 3 | ATP6V1H |  |  | 522 | 18.200 | 0.0818 | No |
| 4 | GNS |  |  | 818 | 15.500 | 0.0929 | No |
| 5 | CLTC |  |  | 1324 | 12.400 | 0.0846 | No |
| 6 | LAMP2 |  |  | 1552 | 11.400 | 0.0922 | No |
| 7 | M6PR |  |  | 1569 | 11.400 | 0.1132 | No |
| 8 | AP1M1 |  |  | 1711 | 10.800 | 0.1251 | No |
| 9 | AP3B1 |  |  | 1755 | 10.600 | 0.1429 | No |
| 10 | ABCA2 |  |  | 1767 | 10.600 | 0.1627 | No |
| 11 | PPT1 |  |  | 1861 | 10.200 | 0.1765 | No |
| 12 | AP1S1 |  |  | 1944 | 9.900 | 0.1904 | No |
| 13 | AP3S1 |  |  | 2139 | 9.200 | 0.1959 | No |
| 14 | AGA |  |  | 2198 | 9.000 | 0.2096 | No |
| 15 | CD63 |  |  | 2216 | 9.000 | 0.2259 | No |
| 16 | HGSNAT |  |  | 2875 | 7.200 | 0.1978 | No |
| 17 | GNPTAB |  |  | 2939 | 7.000 | 0.2073 | No |
| 18 | LAPTM4A |  |  | 3110 | 6.600 | 0.2092 | No |
| 19 | AP4B1 |  |  | 3700 | 5.400 | 0.1820 | No |
| 20 | SCARB2 |  |  | 3746 | 5.300 | 0.1894 | No |
| 21 | AP3M1 |  |  | 3789 | 5.200 | 0.1967 | No |
| 22 | ARSB |  |  | 3813 | 5.100 | 0.2052 | No |
| 23 | AP4E1 |  |  | 3973 | 4.800 | 0.2043 | No |
| 24 | CD164 |  |  | 4009 | 4.800 | 0.2113 | No |
| 25 | SUMF1 |  |  | 4422 | 4.000 | 0.1927 | No |
| 26 | GUSB |  |  | 4686 | 3.600 | 0.1829 | No |
| 27 | AP1G1 |  |  | 4753 | 3.500 | 0.1854 | No |
| 28 | IDS |  |  | 4813 | 3.400 | 0.1882 | No |
| 29 | AP4S1 |  |  | 4868 | 3.300 | 0.1912 | No |
| 30 | ACP2 |  |  | 4872 | 3.300 | 0.1974 | No |
| 31 | GLA |  |  | 4878 | 3.300 | 0.2034 | No |
| 32 | TPP1 |  |  | 4991 | 3.100 | 0.2023 | No |
| 33 | NAPSA |  |  | 5138 | 2.800 | 0.1984 | No |
| 34 | CLTB |  |  | 5145 | 2.800 | 0.2034 | No |
| 35 | LAMP3 |  |  | 5152 | 2.800 | 0.2084 | No |
| 36 | AP4M1 |  |  | 5469 | 2.300 | 0.1927 | No |
| 37 | ATP6AP1 |  |  | 5625 | 2.100 | 0.1868 | No |
| 38 | CTSZ |  |  | 5670 | 2.100 | 0.1881 | No |
| 39 | MFSD8 |  |  | 5702 | 2.000 | 0.1900 | No |
| 40 | GGA2 |  |  | 5776 | 1.900 | 0.1890 | No |
| 41 | SLC11A2 |  |  | 5802 | 1.900 | 0.1911 | No |
| 42 | GALC |  |  | 5888 | 1.800 | 0.1891 | No |
| 43 | GGA3 |  |  | 6076 | 1.500 | 0.1801 | No |
| 44 | CTSO |  |  | 6085 | 1.500 | 0.1824 | No |
| 45 | AP1S3 |  |  | 6106 | 1.500 | 0.1841 | No |
| 46 | ATP6V0A1 |  |  | 6125 | 1.500 | 0.1858 | No |
| 47 | LAMP1 |  |  | 6166 | 1.400 | 0.1860 | No |
| 48 | NPC1 |  |  | 6256 | 1.300 | 0.1828 | No |
| 49 | AP3M2 |  |  | 6274 | 1.300 | 0.1842 | No |
| 50 | ARSA |  |  | 6346 | 1.100 | 0.1818 | No |
| 51 | CLTA |  |  | 6412 | 1.000 | 0.1796 | No |
| 52 | PPT2 |  |  | 6537 | 0.900 | 0.1734 | No |
| 53 | CTSB |  |  | 6624 | 0.800 | 0.1695 | No |
| 54 | DNASE2 |  |  | 6719 | 0.700 | 0.1648 | No |
| 55 | CTSE |  |  | 6917 | 0.500 | 0.1532 | No |
| 56 | AP3S2 |  |  | 6959 | 0.400 | 0.1513 | No |
| 57 | NPC2 |  |  | 7003 | 0.400 | 0.1493 | No |
| 58 | ATP6V0D1 |  |  | 7233 | 0.200 | 0.1351 | No |
| 59 | DNASE2B |  |  | 9067 | 0.000 | 0.0178 | No |
| 60 | CTSG |  |  | 9420 | 0.000 | -0.0047 | No |
| 61 | ATP6V0D2 |  |  | 9735 | 0.000 | -0.0248 | No |
| 62 | ATP6V0A4 |  |  | 9926 | 0.000 | -0.0369 | No |
| 63 | CLN5 |  |  | 10180 | -0.100 | -0.0529 | No |
| 64 | PSAPL1 |  |  | 10325 | -0.100 | -0.0619 | No |
| 65 | AP3B2 |  |  | 10484 | -0.100 | -0.0718 | No |
| 66 | CTSD |  |  | 11022 | -0.200 | -0.1058 | No |
| 67 | SMPD1 |  |  | 11839 | -0.600 | -0.1568 | No |
| 68 | LIPA |  |  | 12129 | -0.700 | -0.1740 | No |
| 69 | ARSG |  |  | 12143 | -0.700 | -0.1735 | No |
| 70 | CTSK |  |  | 12303 | -0.900 | -0.1819 | No |
| 71 | CTNS |  |  | 12552 | -1.000 | -0.1958 | No |
| 72 | MANBA |  |  | 12580 | -1.000 | -0.1956 | No |
| 73 | ABCB9 |  |  | 12637 | -1.100 | -0.1970 | No |
| 74 | HYAL1 |  |  | 12651 | -1.100 | -0.1957 | No |
| 75 | GLB1 |  |  | 12701 | -1.100 | -0.1968 | No |
| 76 | GBA |  |  | 12756 | -1.200 | -0.1979 | No |
| 77 | CTSW |  |  | 12811 | -1.300 | -0.1988 | No |
| 78 | SLC17A5 |  |  | 12920 | -1.400 | -0.2030 | No |
| 79 | MCOLN1 |  |  | 12933 | -1.400 | -0.2011 | No |
| 80 | GALNS |  |  | 13197 | -1.700 | -0.2146 | No |
| 81 | IGF2R |  |  | 13275 | -1.700 | -0.2162 | No |
| 82 | CD68 |  |  | 13469 | -2.000 | -0.2247 | No |
| 83 | ATP6V0B |  |  | 13543 | -2.100 | -0.2253 | No |
| 84 | LGMN |  |  | 13549 | -2.100 | -0.2216 | No |
| 85 | ATP6V0C |  |  | 13612 | -2.200 | -0.2213 | No |
| 86 | ATP6V0A2 |  |  | 13705 | -2.300 | -0.2227 | No |
| 87 | CLN3 |  |  | 13725 | -2.400 | -0.2193 | No |
| 88 | IDUA |  |  | 13802 | -2.500 | -0.2193 | No |
| 89 | NAGLU |  |  | 14005 | -2.800 | -0.2268 | Yes |
| 90 | GAA |  |  | 14061 | -2.900 | -0.2247 | Yes |
| 91 | TCIRG1 |  |  | 14117 | -3.000 | -0.2224 | Yes |
| 92 | LAPTM4B |  |  | 14118 | -3.000 | -0.2166 | Yes |
| 93 | ENTPD4 |  |  | 14246 | -3.200 | -0.2185 | Yes |
| 94 | GNPTG |  |  | 14290 | -3.300 | -0.2149 | Yes |
| 95 | GGA1 |  |  | 14305 | -3.300 | -0.2094 | Yes |
| 96 | NAGPA |  |  | 14348 | -3.400 | -0.2055 | Yes |
| 97 | SLC11A1 |  |  | 14376 | -3.500 | -0.2004 | Yes |
| 98 | HEXA |  |  | 14447 | -3.700 | -0.1978 | Yes |
| 99 | NEU1 |  |  | 14592 | -4.100 | -0.1990 | Yes |
| 100 | CTSC |  |  | 14758 | -4.500 | -0.2009 | Yes |
| 101 | AP3D1 |  |  | 14853 | -4.700 | -0.1978 | Yes |
| 102 | FUCA1 |  |  | 14944 | -5.000 | -0.1938 | Yes |
| 103 | LAPTM5 |  |  | 15045 | -5.400 | -0.1898 | Yes |
| 104 | SGSH |  |  | 15104 | -5.700 | -0.1825 | Yes |
| 105 | SORT1 |  |  | 15161 | -6.000 | -0.1744 | Yes |
| 106 | CTSH |  |  | 15175 | -6.000 | -0.1636 | Yes |
| 107 | CTSF |  |  | 15223 | -6.300 | -0.1544 | Yes |
| 108 | PSAP |  |  | 15338 | -7.000 | -0.1482 | Yes |
| 109 | GM2A |  |  | 15342 | -7.000 | -0.1348 | Yes |
| 110 | HEXB |  |  | 15432 | -7.800 | -0.1254 | Yes |
| 111 | MAN2B1 |  |  | 15440 | -7.900 | -0.1106 | Yes |
| 112 | CTSA |  |  | 15485 | -8.400 | -0.0971 | Yes |
| 113 | CTSS |  |  | 15520 | -8.800 | -0.0822 | Yes |
| 114 | AP1B1 |  |  | 15567 | -9.600 | -0.0666 | Yes |
| 115 | ACP5 |  |  | 15611 | -10.400 | -0.0492 | Yes |
| 116 | NAGA |  |  | 15657 | -12.100 | -0.0286 | Yes |
| 117 | AP1M2 |  |  | 15724 | -17.800 | 0.0016 | Yes |
Table: GSEA details [plain text format]

  

Fig 2: KEGG\_LYSOSOME: Random ES distribution      
 Gene set null distribution of ES for **KEGG\_LYSOSOME**

  
