# Supplementary material for: Immunomic, genomic and transcriptomic characterization of CT26 colorectal carcinoma
Source: BMC Genomics. 2014 Mar 13;15(1):190. doi: 10.1186/1471-2164-15-190 (PMC4007559; doi:10.1186/1471-2164-15-190)
Supplement: Supplementary file 8 — Additional file 8: Contains the Gene Pattern gene set membership and enrichment values in an html format. The file index.html is the entry point. (ZIP 13 MB) [file 12864_2013_7028_MOESM8_ESM.zip › KEGG_NEUROACTIVE_LIGAND_RECEPTOR_INTERACTION.html]

Details for gene set KEGG\_NEUROACTIVE\_LIGAND\_RECEPTOR\_INTERACTION[GSEA]

|  || Dataset | CT26\_gene\_expression |
| Phenotype | NoPhenotypeAvailable |
| Upregulated in class | na\_neg |
| GeneSet | KEGG\_NEUROACTIVE\_LIGAND\_RECEPTOR\_INTERACTION |
| Enrichment Score (ES) | -0.39335534 |
| Normalized Enrichment Score (NES) | NaN |
| Nominal p-value | NaN |
| FDR q-value | 1.0 |
| FWER p-Value | 0.0 |
Table: GSEA Results Summary

  

Fig 1: Enrichment plot: KEGG\_NEUROACTIVE\_LIGAND\_RECEPTOR\_INTERACTION      
 Profile of the Running ES Score & Positions of GeneSet Members on the Rank Ordered List

  

| PROBE | GENE SYMBOL | GENE\_TITLE | RANK IN GENE LIST | RANK METRIC SCORE | RUNNING ES | CORE ENRICHMENT || 1 | NR3C1 |  |  | 1168 | 13.200 | -0.0131 | No |
| 2 | GHR |  |  | 1223 | 13.000 | 0.0447 | No |
| 3 | F2R |  |  | 2440 | 8.400 | 0.0059 | No |
| 4 | CHRNB1 |  |  | 2575 | 8.000 | 0.0349 | No |
| 5 | P2RX3 |  |  | 4041 | 4.700 | -0.0374 | No |
| 6 | GABBR1 |  |  | 4585 | 3.700 | -0.0550 | No |
| 7 | PARD3 |  |  | 4640 | 3.700 | -0.0410 | No |
| 8 | THRB |  |  | 4999 | 3.000 | -0.0500 | No |
| 9 | TSPO |  |  | 6428 | 1.000 | -0.1374 | No |
| 10 | P2RX7 |  |  | 6554 | 0.900 | -0.1412 | No |
| 11 | PRL |  |  | 6706 | 0.700 | -0.1476 | No |
| 12 | CALCRL |  |  | 6853 | 0.500 | -0.1547 | No |
| 13 | GRIK5 |  |  | 6876 | 0.500 | -0.1537 | No |
| 14 | ADRA2B |  |  | 7044 | 0.400 | -0.1626 | No |
| 15 | P2RY14 |  |  | 7245 | 0.200 | -0.1746 | No |
| 16 | GABRD |  |  | 7255 | 0.200 | -0.1742 | No |
| 17 | NTSR2 |  |  | 7274 | 0.200 | -0.1744 | No |
| 18 | LHB |  |  | 7411 | 0.100 | -0.1827 | No |
| 19 | ADRA1D |  |  | 7481 | 0.100 | -0.1867 | No |
| 20 | MC3R |  |  | 7564 | 0.000 | -0.1920 | No |
| 21 | CHRNE |  |  | 7648 | 0.000 | -0.1974 | No |
| 22 | TRHR |  |  | 7758 | 0.000 | -0.2044 | No |
| 23 | CHRNA1 |  |  | 7788 | 0.000 | -0.2063 | No |
| 24 | GLRA2 |  |  | 7794 | 0.000 | -0.2066 | No |
| 25 | CHRNA5 |  |  | 7815 | 0.000 | -0.2079 | No |
| 26 | RXFP2 |  |  | 7821 | 0.000 | -0.2082 | No |
| 27 | HRH4 |  |  | 7848 | 0.000 | -0.2099 | No |
| 28 | PTGDR |  |  | 7881 | 0.000 | -0.2119 | No |
| 29 | GRM3 |  |  | 7901 | 0.000 | -0.2132 | No |
| 30 | GRM8 |  |  | 7911 | 0.000 | -0.2137 | No |
| 31 | GRIN2A |  |  | 7969 | 0.000 | -0.2174 | No |
| 32 | CHRNB3 |  |  | 7973 | 0.000 | -0.2176 | No |
| 33 | CHRNA9 |  |  | 7989 | 0.000 | -0.2186 | No |
| 34 | GABRG1 |  |  | 8000 | 0.000 | -0.2192 | No |
| 35 | MC1R |  |  | 8028 | 0.000 | -0.2210 | No |
| 36 | BRS3 |  |  | 8130 | 0.000 | -0.2275 | No |
| 37 | CALCR |  |  | 8135 | 0.000 | -0.2277 | No |
| 38 | CGA |  |  | 8158 | 0.000 | -0.2292 | No |
| 39 | CHRM5 |  |  | 8159 | 0.000 | -0.2292 | No |
| 40 | DRD5 |  |  | 8229 | 0.000 | -0.2336 | No |
| 41 | FSHB |  |  | 8272 | 0.000 | -0.2363 | No |
| 42 | FSHR |  |  | 8273 | 0.000 | -0.2363 | No |
| 43 | GABRA2 |  |  | 8276 | 0.000 | -0.2365 | No |
| 44 | GABRA4 |  |  | 8277 | 0.000 | -0.2365 | No |
| 45 | GABRA5 |  |  | 8278 | 0.000 | -0.2365 | No |
| 46 | GABRA6 |  |  | 8279 | 0.000 | -0.2365 | No |
| 47 | GABRB1 |  |  | 8280 | 0.000 | -0.2365 | No |
| 48 | GHSR |  |  | 8289 | 0.000 | -0.2370 | No |
| 49 | GLRA1 |  |  | 8295 | 0.000 | -0.2373 | No |
| 50 | GLRA3 |  |  | 8296 | 0.000 | -0.2373 | No |
| 51 | GNRHR |  |  | 8305 | 0.000 | -0.2378 | No |
| 52 | GPR50 |  |  | 8310 | 0.000 | -0.2381 | No |
| 53 | GRM5 |  |  | 8314 | 0.000 | -0.2383 | No |
| 54 | HTR1A |  |  | 8333 | 0.000 | -0.2394 | No |
| 55 | HTR2C |  |  | 8334 | 0.000 | -0.2394 | No |
| 56 | MTNR1B |  |  | 8469 | 0.000 | -0.2481 | No |
| 57 | TAAR1 |  |  | 8872 | 0.000 | -0.2740 | No |
| 58 | TAAR2 |  |  | 8873 | 0.000 | -0.2740 | No |
| 59 | TAAR5 |  |  | 8875 | 0.000 | -0.2741 | No |
| 60 | TAAR6 |  |  | 8876 | 0.000 | -0.2741 | No |
| 61 | TAAR8 |  |  | 8877 | 0.000 | -0.2741 | No |
| 62 | TAAR9 |  |  | 8878 | 0.000 | -0.2741 | No |
| 63 | GRID2 |  |  | 8979 | 0.000 | -0.2805 | No |
| 64 | HTR5A |  |  | 8989 | 0.000 | -0.2811 | No |
| 65 | GABRB2 |  |  | 8991 | 0.000 | -0.2812 | No |
| 66 | HRH1 |  |  | 8993 | 0.000 | -0.2812 | No |
| 67 | LHCGR |  |  | 9014 | 0.000 | -0.2825 | No |
| 68 | PLG |  |  | 9037 | 0.000 | -0.2839 | No |
| 69 | GABRG2 |  |  | 9048 | 0.000 | -0.2846 | No |
| 70 | GABRA1 |  |  | 9055 | 0.000 | -0.2850 | No |
| 71 | RXFP1 |  |  | 9086 | 0.000 | -0.2869 | No |
| 72 | CHRNG |  |  | 9101 | 0.000 | -0.2878 | No |
| 73 | NPFFR2 |  |  | 9150 | 0.000 | -0.2909 | No |
| 74 | GABRQ |  |  | 9159 | 0.000 | -0.2914 | No |
| 75 | CHRND |  |  | 9168 | 0.000 | -0.2919 | No |
| 76 | MTNR1A |  |  | 9273 | 0.000 | -0.2986 | No |
| 77 | GABBR2 |  |  | 9284 | 0.000 | -0.2993 | No |
| 78 | GRM1 |  |  | 9338 | 0.000 | -0.3027 | No |
| 79 | HTR2A |  |  | 9366 | 0.000 | -0.3044 | No |
| 80 | UTS2R |  |  | 9367 | 0.000 | -0.3044 | No |
| 81 | NPY5R |  |  | 9385 | 0.000 | -0.3055 | No |
| 82 | HCRTR2 |  |  | 9388 | 0.000 | -0.3057 | No |
| 83 | DRD1 |  |  | 9397 | 0.000 | -0.3062 | No |
| 84 | CTSG |  |  | 9420 | 0.000 | -0.3076 | No |
| 85 | GRIK1 |  |  | 9427 | 0.000 | -0.3080 | No |
| 86 | GRIN2B |  |  | 9447 | 0.000 | -0.3092 | No |
| 87 | TSHB |  |  | 9452 | 0.000 | -0.3095 | No |
| 88 | GRIA2 |  |  | 9468 | 0.000 | -0.3104 | No |
| 89 | GABRR1 |  |  | 9476 | 0.000 | -0.3109 | No |
| 90 | GRIN2C |  |  | 9484 | 0.000 | -0.3113 | No |
| 91 | NMUR2 |  |  | 9495 | 0.000 | -0.3120 | No |
| 92 | MC4R |  |  | 9554 | 0.000 | -0.3157 | No |
| 93 | GRIK2 |  |  | 9560 | 0.000 | -0.3160 | No |
| 94 | CRHR1 |  |  | 9570 | 0.000 | -0.3166 | No |
| 95 | CHRNA6 |  |  | 9576 | 0.000 | -0.3170 | No |
| 96 | GRM2 |  |  | 9597 | 0.000 | -0.3182 | No |
| 97 | OPRM1 |  |  | 9605 | 0.000 | -0.3187 | No |
| 98 | MC5R |  |  | 9630 | 0.000 | -0.3202 | No |
| 99 | HTR7 |  |  | 9637 | 0.000 | -0.3206 | No |
| 100 | AGTR2 |  |  | 9655 | 0.000 | -0.3217 | No |
| 101 | HTR1F |  |  | 9658 | 0.000 | -0.3219 | No |
| 102 | GPR156 |  |  | 9676 | 0.000 | -0.3230 | No |
| 103 | NMBR |  |  | 9686 | 0.000 | -0.3235 | No |
| 104 | GABRG3 |  |  | 9715 | 0.000 | -0.3253 | No |
| 105 | HRH3 |  |  | 9732 | 0.000 | -0.3264 | No |
| 106 | MC2R |  |  | 9747 | 0.000 | -0.3273 | No |
| 107 | CHRNA2 |  |  | 9763 | 0.000 | -0.3282 | No |
| 108 | HTR1D |  |  | 9834 | 0.000 | -0.3328 | No |
| 109 | GRM6 |  |  | 9840 | 0.000 | -0.3331 | No |
| 110 | GHRHR |  |  | 9841 | 0.000 | -0.3331 | No |
| 111 | MAS1 |  |  | 9910 | 0.000 | -0.3375 | No |
| 112 | CHRNA4 |  |  | 9912 | 0.000 | -0.3375 | No |
| 113 | GRIA1 |  |  | 9936 | 0.000 | -0.3390 | No |
| 114 | GABRB3 |  |  | 9971 | 0.000 | -0.3412 | No |
| 115 | ADORA3 |  |  | 10011 | 0.000 | -0.3437 | No |
| 116 | DRD2 |  |  | 10026 | 0.000 | -0.3446 | No |
| 117 | CCKBR |  |  | 10084 | 0.000 | -0.3483 | No |
| 118 | GLRB |  |  | 10106 | 0.000 | -0.3497 | No |
| 119 | FPR1 |  |  | 10156 | -0.100 | -0.3523 | No |
| 120 | NPBWR1 |  |  | 10190 | -0.100 | -0.3540 | No |
| 121 | GABRP |  |  | 10218 | -0.100 | -0.3553 | No |
| 122 | P2RX5 |  |  | 10220 | -0.100 | -0.3549 | No |
| 123 | GRIN1 |  |  | 10279 | -0.100 | -0.3581 | No |
| 124 | GABRR2 |  |  | 10290 | -0.100 | -0.3583 | No |
| 125 | PRLR |  |  | 10318 | -0.100 | -0.3596 | No |
| 126 | P2RY10 |  |  | 10322 | -0.100 | -0.3593 | No |
| 127 | ADRA1A |  |  | 10328 | -0.100 | -0.3592 | No |
| 128 | PTGFR |  |  | 10339 | -0.100 | -0.3593 | No |
| 129 | GZMA |  |  | 10340 | -0.100 | -0.3589 | No |
| 130 | GRIA4 |  |  | 10348 | -0.100 | -0.3588 | No |
| 131 | GABRA3 |  |  | 10349 | -0.100 | -0.3584 | No |
| 132 | OPRK1 |  |  | 10357 | -0.100 | -0.3583 | No |
| 133 | GRID1 |  |  | 10369 | -0.100 | -0.3586 | No |
| 134 | AVPR2 |  |  | 10377 | -0.100 | -0.3586 | No |
| 135 | GRIA3 |  |  | 10424 | -0.100 | -0.3611 | No |
| 136 | GRM7 |  |  | 10450 | -0.100 | -0.3622 | No |
| 137 | CRHR2 |  |  | 10452 | -0.100 | -0.3618 | No |
| 138 | TACR3 |  |  | 10461 | -0.100 | -0.3618 | No |
| 139 | HTR6 |  |  | 10490 | -0.100 | -0.3632 | No |
| 140 | CYSLTR1 |  |  | 10497 | -0.100 | -0.3631 | No |
| 141 | CYSLTR2 |  |  | 10507 | -0.100 | -0.3632 | No |
| 142 | HRH2 |  |  | 10527 | -0.100 | -0.3639 | No |
| 143 | GRM4 |  |  | 10561 | -0.100 | -0.3656 | No |
| 144 | PRLHR |  |  | 10601 | -0.100 | -0.3676 | No |
| 145 | SCTR |  |  | 10620 | -0.100 | -0.3683 | No |
| 146 | DRD3 |  |  | 10688 | -0.100 | -0.3722 | No |
| 147 | DRD4 |  |  | 10713 | -0.100 | -0.3733 | No |
| 148 | GLP1R |  |  | 10729 | -0.100 | -0.3738 | No |
| 149 | GRIN2D |  |  | 10741 | -0.100 | -0.3740 | No |
| 150 | GABRE |  |  | 10761 | -0.100 | -0.3748 | No |
| 151 | GCGR |  |  | 10768 | -0.100 | -0.3747 | No |
| 152 | TRPV1 |  |  | 10796 | -0.100 | -0.3759 | No |
| 153 | TSHR |  |  | 10809 | -0.200 | -0.3758 | No |
| 154 | GRIK4 |  |  | 10811 | -0.200 | -0.3749 | No |
| 155 | GALR3 |  |  | 10878 | -0.200 | -0.3782 | No |
| 156 | OXTR |  |  | 10880 | -0.200 | -0.3773 | No |
| 157 | OPRL1 |  |  | 10930 | -0.200 | -0.3795 | No |
| 158 | GALR1 |  |  | 10975 | -0.200 | -0.3814 | No |
| 159 | CHRNA7 |  |  | 11004 | -0.200 | -0.3823 | No |
| 160 | AVPR1B |  |  | 11056 | -0.200 | -0.3847 | No |
| 161 | ADRA1B |  |  | 11074 | -0.200 | -0.3848 | No |
| 162 | CHRNA10 |  |  | 11083 | -0.200 | -0.3844 | No |
| 163 | HTR2B |  |  | 11086 | -0.200 | -0.3836 | No |
| 164 | HTR1B |  |  | 11107 | -0.200 | -0.3839 | No |
| 165 | F2RL2 |  |  | 11120 | -0.200 | -0.3837 | No |
| 166 | GRPR |  |  | 11139 | -0.200 | -0.3840 | No |
| 167 | NPY2R |  |  | 11142 | -0.200 | -0.3831 | No |
| 168 | SSTR5 |  |  | 11181 | -0.300 | -0.3842 | No |
| 169 | SSTR3 |  |  | 11204 | -0.300 | -0.3842 | No |
| 170 | F2 |  |  | 11305 | -0.300 | -0.3892 | No |
| 171 | TACR1 |  |  | 11308 | -0.300 | -0.3879 | No |
| 172 | LEPR |  |  | 11393 | -0.300 | -0.3919 | Yes |
| 173 | CHRM4 |  |  | 11404 | -0.300 | -0.3912 | Yes |
| 174 | NPFFR1 |  |  | 11432 | -0.300 | -0.3915 | Yes |
| 175 | GRIK3 |  |  | 11436 | -0.300 | -0.3903 | Yes |
| 176 | CNR1 |  |  | 11468 | -0.400 | -0.3904 | Yes |
| 177 | ADORA2B |  |  | 11483 | -0.400 | -0.3894 | Yes |
| 178 | P2RY13 |  |  | 11489 | -0.400 | -0.3878 | Yes |
| 179 | GPR83 |  |  | 11552 | -0.400 | -0.3900 | Yes |
| 180 | LEP |  |  | 11569 | -0.400 | -0.3891 | Yes |
| 181 | BDKRB1 |  |  | 11619 | -0.400 | -0.3904 | Yes |
| 182 | OPRD1 |  |  | 11624 | -0.400 | -0.3888 | Yes |
| 183 | GRIN3A |  |  | 11635 | -0.400 | -0.3875 | Yes |
| 184 | NTSR1 |  |  | 11651 | -0.400 | -0.3866 | Yes |
| 185 | CNR2 |  |  | 11682 | -0.500 | -0.3862 | Yes |
| 186 | GIPR |  |  | 11698 | -0.500 | -0.3848 | Yes |
| 187 | SSTR4 |  |  | 11739 | -0.500 | -0.3850 | Yes |
| 188 | LTB4R |  |  | 11764 | -0.500 | -0.3842 | Yes |
| 189 | ADRB2 |  |  | 11843 | -0.600 | -0.3864 | Yes |
| 190 | CHRNB2 |  |  | 11866 | -0.600 | -0.3850 | Yes |
| 191 | LTB4R2 |  |  | 11943 | -0.600 | -0.3871 | Yes |
| 192 | GLP2R |  |  | 11954 | -0.600 | -0.3849 | Yes |
| 193 | GRIN3B |  |  | 11964 | -0.600 | -0.3826 | Yes |
| 194 | C3AR1 |  |  | 11965 | -0.600 | -0.3798 | Yes |
| 195 | NPY1R |  |  | 12054 | -0.700 | -0.3822 | Yes |
| 196 | MCHR1 |  |  | 12108 | -0.700 | -0.3823 | Yes |
| 197 | EDNRB |  |  | 12155 | -0.700 | -0.3820 | Yes |
| 198 | PTGER2 |  |  | 12243 | -0.800 | -0.3838 | Yes |
| 199 | ADRA2C |  |  | 12250 | -0.800 | -0.3804 | Yes |
| 200 | ADORA1 |  |  | 12252 | -0.800 | -0.3767 | Yes |
| 201 | CHRM3 |  |  | 12254 | -0.800 | -0.3730 | Yes |
| 202 | P2RX1 |  |  | 12305 | -0.900 | -0.3720 | Yes |
| 203 | VIPR2 |  |  | 12317 | -0.900 | -0.3684 | Yes |
| 204 | PRSS3 |  |  | 12352 | -0.900 | -0.3664 | Yes |
| 205 | PRSS1 |  |  | 12370 | -0.900 | -0.3633 | Yes |
| 206 | PRSS2 |  |  | 12387 | -0.900 | -0.3600 | Yes |
| 207 | PTGIR |  |  | 12396 | -0.900 | -0.3563 | Yes |
| 208 | AVPR1A |  |  | 12399 | -0.900 | -0.3522 | Yes |
| 209 | NMUR1 |  |  | 12483 | -1.000 | -0.3528 | Yes |
| 210 | P2RY2 |  |  | 12549 | -1.000 | -0.3523 | Yes |
| 211 | ADORA2A |  |  | 12713 | -1.200 | -0.3572 | Yes |
| 212 | CHRNA3 |  |  | 12753 | -1.200 | -0.3540 | Yes |
| 213 | CCKAR |  |  | 12831 | -1.300 | -0.3529 | Yes |
| 214 | HCRTR1 |  |  | 12888 | -1.300 | -0.3504 | Yes |
| 215 | CHRM2 |  |  | 12903 | -1.300 | -0.3451 | Yes |
| 216 | HTR4 |  |  | 12993 | -1.400 | -0.3443 | Yes |
| 217 | P2RY4 |  |  | 13016 | -1.500 | -0.3386 | Yes |
| 218 | AGTR1 |  |  | 13128 | -1.600 | -0.3382 | Yes |
| 219 | EDNRA |  |  | 13135 | -1.600 | -0.3311 | Yes |
| 220 | ADCYAP1R1 |  |  | 13187 | -1.700 | -0.3263 | Yes |
| 221 | KISS1R |  |  | 13212 | -1.700 | -0.3199 | Yes |
| 222 | GALR2 |  |  | 13227 | -1.700 | -0.3128 | Yes |
| 223 | THRA |  |  | 13286 | -1.800 | -0.3080 | Yes |
| 224 | CHRNB4 |  |  | 13371 | -1.900 | -0.3045 | Yes |
| 225 | P2RX2 |  |  | 13509 | -2.000 | -0.3039 | Yes |
| 226 | SSTR2 |  |  | 13633 | -2.200 | -0.3015 | Yes |
| 227 | CHRM1 |  |  | 13644 | -2.200 | -0.2917 | Yes |
| 228 | PTAFR |  |  | 13676 | -2.300 | -0.2829 | Yes |
| 229 | F2RL3 |  |  | 13786 | -2.400 | -0.2786 | Yes |
| 230 | ADRB1 |  |  | 13803 | -2.500 | -0.2678 | Yes |
| 231 | BDKRB2 |  |  | 13805 | -2.500 | -0.2561 | Yes |
| 232 | PTGER3 |  |  | 13814 | -2.500 | -0.2448 | Yes |
| 233 | PPYR1 |  |  | 13845 | -2.600 | -0.2345 | Yes |
| 234 | C5AR1 |  |  | 13884 | -2.600 | -0.2247 | Yes |
| 235 | P2RY1 |  |  | 14216 | -3.100 | -0.2315 | Yes |
| 236 | PTGER4 |  |  | 14532 | -3.900 | -0.2334 | Yes |
| 237 | ADRB3 |  |  | 14670 | -4.300 | -0.2219 | Yes |
| 238 | P2RY6 |  |  | 14706 | -4.300 | -0.2039 | Yes |
| 239 | ADRA2A |  |  | 14754 | -4.500 | -0.1857 | Yes |
| 240 | GPR35 |  |  | 14764 | -4.500 | -0.1651 | Yes |
| 241 | TBXA2R |  |  | 14827 | -4.700 | -0.1469 | Yes |
| 242 | TACR2 |  |  | 14856 | -4.700 | -0.1266 | Yes |
| 243 | SSTR1 |  |  | 15216 | -6.300 | -0.1200 | Yes |
| 244 | PTGER1 |  |  | 15335 | -7.000 | -0.0947 | Yes |
| 245 | F2RL1 |  |  | 15456 | -8.000 | -0.0647 | Yes |
| 246 | P2RX4 |  |  | 15498 | -8.500 | -0.0272 | Yes |
| 247 | VIPR1 |  |  | 15540 | -9.200 | 0.0135 | Yes |
Table: GSEA details [plain text format]

  

Fig 2: KEGG\_NEUROACTIVE\_LIGAND\_RECEPTOR\_INTERACTION: Random ES distribution      
 Gene set null distribution of ES for **KEGG\_NEUROACTIVE\_LIGAND\_RECEPTOR\_INTERACTION**

  
