# Supplementary material for: Immunomic, genomic and transcriptomic characterization of CT26 colorectal carcinoma
Source: BMC Genomics. 2014 Mar 13;15(1):190. doi: 10.1186/1471-2164-15-190 (PMC4007559; doi:10.1186/1471-2164-15-190)
Supplement: Supplementary file 8 — Additional file 8: Contains the Gene Pattern gene set membership and enrichment values in an html format. The file index.html is the entry point. (ZIP 13 MB) [file 12864_2013_7028_MOESM8_ESM.zip › KIM_RESPONSE_TO_TSA_AND_DECITABINE_UP.html]

Details for gene set KIM\_RESPONSE\_TO\_TSA\_AND\_DECITABINE\_UP[GSEA]

|  || Dataset | CT26\_gene\_expression |
| Phenotype | NoPhenotypeAvailable |
| Upregulated in class | na\_neg |
| GeneSet | KIM\_RESPONSE\_TO\_TSA\_AND\_DECITABINE\_UP |
| Enrichment Score (ES) | -0.4519609 |
| Normalized Enrichment Score (NES) | NaN |
| Nominal p-value | NaN |
| FDR q-value | 1.0 |
| FWER p-Value | 0.0 |
Table: GSEA Results Summary

  

Fig 1: Enrichment plot: KIM\_RESPONSE\_TO\_TSA\_AND\_DECITABINE\_UP      
 Profile of the Running ES Score & Positions of GeneSet Members on the Rank Ordered List

  

| PROBE | GENE SYMBOL | GENE\_TITLE | RANK IN GENE LIST | RANK METRIC SCORE | RUNNING ES | CORE ENRICHMENT || 1 | NEFM |  |  | 12 | 50.900 | 0.1067 | No |
| 2 | TRIP6 |  |  | 1123 | 13.400 | 0.0640 | No |
| 3 | SAT1 |  |  | 1523 | 11.500 | 0.0627 | No |
| 4 | NEFL |  |  | 1544 | 11.500 | 0.0857 | No |
| 5 | GCHFR |  |  | 1587 | 11.300 | 0.1069 | No |
| 6 | DNAJB9 |  |  | 1827 | 10.300 | 0.1133 | No |
| 7 | NXT2 |  |  | 1952 | 9.900 | 0.1263 | No |
| 8 | LXN |  |  | 2009 | 9.600 | 0.1430 | No |
| 9 | CDKN1A |  |  | 2165 | 9.100 | 0.1523 | No |
| 10 | IFI27 |  |  | 2622 | 7.900 | 0.1398 | No |
| 11 | HTATIP2 |  |  | 2685 | 7.700 | 0.1521 | No |
| 12 | SERPINF1 |  |  | 2990 | 6.900 | 0.1472 | No |
| 13 | LAMC2 |  |  | 5100 | 2.900 | 0.0184 | No |
| 14 | FGD6 |  |  | 5427 | 2.400 | 0.0026 | No |
| 15 | NDUFA2 |  |  | 5636 | 2.100 | -0.0062 | No |
| 16 | IL1RAP |  |  | 5660 | 2.100 | -0.0033 | No |
| 17 | S100A3 |  |  | 6047 | 1.600 | -0.0246 | No |
| 18 | RNASET2 |  |  | 6340 | 1.200 | -0.0407 | No |
| 19 | PARD6A |  |  | 6437 | 1.000 | -0.0448 | No |
| 20 | TSGA10 |  |  | 6882 | 0.500 | -0.0721 | No |
| 21 | DNALI1 |  |  | 7067 | 0.300 | -0.0832 | No |
| 22 | TES |  |  | 7158 | 0.300 | -0.0884 | No |
| 23 | FBXO2 |  |  | 7311 | 0.100 | -0.0979 | No |
| 24 | KRT86 |  |  | 7328 | 0.100 | -0.0987 | No |
| 25 | CABYR |  |  | 7455 | 0.100 | -0.1065 | No |
| 26 | IL24 |  |  | 7459 | 0.100 | -0.1065 | No |
| 27 | S100A2 |  |  | 7596 | 0.000 | -0.1152 | No |
| 28 | SERPINI1 |  |  | 7609 | 0.000 | -0.1160 | No |
| 29 | CYP24A1 |  |  | 8019 | 0.000 | -0.1421 | No |
| 30 | CD70 |  |  | 8147 | 0.000 | -0.1503 | No |
| 31 | MAGEA4 |  |  | 8442 | 0.000 | -0.1691 | No |
| 32 | MAGEB1 |  |  | 8444 | 0.000 | -0.1691 | No |
| 33 | MAGEB2 |  |  | 8447 | 0.000 | -0.1692 | No |
| 34 | FAM50B |  |  | 9194 | 0.000 | -0.2170 | No |
| 35 | SLC25A31 |  |  | 9222 | 0.000 | -0.2187 | No |
| 36 | CPA4 |  |  | 9401 | 0.000 | -0.2301 | No |
| 37 | TRIM58 |  |  | 9472 | 0.000 | -0.2345 | No |
| 38 | FADS3 |  |  | 9598 | 0.000 | -0.2425 | No |
| 39 | HSD17B6 |  |  | 9615 | 0.000 | -0.2436 | No |
| 40 | STAG3 |  |  | 9861 | 0.000 | -0.2592 | No |
| 41 | KCNV2 |  |  | 9891 | 0.000 | -0.2611 | No |
| 42 | TACSTD2 |  |  | 9909 | 0.000 | -0.2622 | No |
| 43 | IL1R2 |  |  | 9980 | 0.000 | -0.2667 | No |
| 44 | CST6 |  |  | 10005 | 0.000 | -0.2682 | No |
| 45 | NMB |  |  | 10045 | 0.000 | -0.2707 | No |
| 46 | COLEC11 |  |  | 10177 | -0.100 | -0.2788 | No |
| 47 | HSD17B1 |  |  | 10454 | -0.100 | -0.2963 | No |
| 48 | AQP3 |  |  | 10500 | -0.100 | -0.2990 | No |
| 49 | CSPG5 |  |  | 10701 | -0.100 | -0.3115 | No |
| 50 | NINJ2 |  |  | 10932 | -0.200 | -0.3258 | No |
| 51 | TSPYL5 |  |  | 10977 | -0.200 | -0.3282 | No |
| 52 | WISP2 |  |  | 11285 | -0.300 | -0.3472 | No |
| 53 | KRT23 |  |  | 11398 | -0.300 | -0.3537 | No |
| 54 | ARL14 |  |  | 11603 | -0.400 | -0.3659 | No |
| 55 | RND2 |  |  | 11955 | -0.600 | -0.3871 | No |
| 56 | PPL |  |  | 12065 | -0.700 | -0.3926 | No |
| 57 | RPP25 |  |  | 12086 | -0.700 | -0.3924 | No |
| 58 | PSCA |  |  | 12187 | -0.800 | -0.3971 | No |
| 59 | HYAL1 |  |  | 12651 | -1.100 | -0.4244 | No |
| 60 | TPD52L1 |  |  | 12800 | -1.200 | -0.4314 | No |
| 61 | HNF4G |  |  | 12926 | -1.400 | -0.4364 | No |
| 62 | VAMP8 |  |  | 12956 | -1.400 | -0.4353 | No |
| 63 | HIST1H2BC |  |  | 12985 | -1.400 | -0.4341 | No |
| 64 | SLC27A2 |  |  | 13127 | -1.600 | -0.4398 | No |
| 65 | MAP1LC3B |  |  | 13300 | -1.800 | -0.4470 | No |
| 66 | CLIC3 |  |  | 13379 | -1.900 | -0.4480 | Yes |
| 67 | TOB1 |  |  | 13383 | -1.900 | -0.4441 | Yes |
| 68 | ICAM2 |  |  | 13385 | -1.900 | -0.4402 | Yes |
| 69 | CYP3A5 |  |  | 13415 | -1.900 | -0.4380 | Yes |
| 70 | FKBP1B |  |  | 13440 | -1.900 | -0.4356 | Yes |
| 71 | HCLS1 |  |  | 13478 | -2.000 | -0.4337 | Yes |
| 72 | LGMN |  |  | 13549 | -2.100 | -0.4337 | Yes |
| 73 | HIST1H2BD |  |  | 13709 | -2.300 | -0.4391 | Yes |
| 74 | COL3A1 |  |  | 13760 | -2.400 | -0.4372 | Yes |
| 75 | ATF3 |  |  | 13829 | -2.500 | -0.4363 | Yes |
| 76 | BIK |  |  | 13901 | -2.600 | -0.4353 | Yes |
| 77 | SLC6A8 |  |  | 13906 | -2.600 | -0.4301 | Yes |
| 78 | TAC1 |  |  | 13930 | -2.700 | -0.4259 | Yes |
| 79 | ISG20 |  |  | 13963 | -2.700 | -0.4222 | Yes |
| 80 | CDO1 |  |  | 13968 | -2.700 | -0.4168 | Yes |
| 81 | HN1 |  |  | 13980 | -2.700 | -0.4118 | Yes |
| 82 | MYO5C |  |  | 13983 | -2.700 | -0.4062 | Yes |
| 83 | MUC1 |  |  | 14055 | -2.900 | -0.4046 | Yes |
| 84 | GDF15 |  |  | 14074 | -2.900 | -0.3996 | Yes |
| 85 | CXADR |  |  | 14214 | -3.100 | -0.4020 | Yes |
| 86 | CDA |  |  | 14224 | -3.200 | -0.3958 | Yes |
| 87 | IRF7 |  |  | 14385 | -3.500 | -0.3987 | Yes |
| 88 | MUC13 |  |  | 14428 | -3.700 | -0.3935 | Yes |
| 89 | TM7SF2 |  |  | 14430 | -3.700 | -0.3858 | Yes |
| 90 | SFN |  |  | 14484 | -3.800 | -0.3812 | Yes |
| 91 | KRT17 |  |  | 14517 | -3.900 | -0.3750 | Yes |
| 92 | HSPA2 |  |  | 14518 | -3.900 | -0.3667 | Yes |
| 93 | PECAM1 |  |  | 14557 | -3.900 | -0.3609 | Yes |
| 94 | IFI30 |  |  | 14652 | -4.200 | -0.3581 | Yes |
| 95 | CDCP1 |  |  | 14707 | -4.300 | -0.3525 | Yes |
| 96 | FUCA1 |  |  | 14944 | -5.000 | -0.3570 | Yes |
| 97 | RHOF |  |  | 14951 | -5.000 | -0.3468 | Yes |
| 98 | FAAH |  |  | 14961 | -5.100 | -0.3367 | Yes |
| 99 | TGM2 |  |  | 15062 | -5.500 | -0.3314 | Yes |
| 100 | MAP7 |  |  | 15177 | -6.000 | -0.3261 | Yes |
| 101 | CLU |  |  | 15225 | -6.300 | -0.3158 | Yes |
| 102 | TSPAN1 |  |  | 15280 | -6.600 | -0.3053 | Yes |
| 103 | KRT19 |  |  | 15311 | -6.800 | -0.2929 | Yes |
| 104 | IL18 |  |  | 15349 | -7.100 | -0.2802 | Yes |
| 105 | F2RL1 |  |  | 15456 | -8.000 | -0.2701 | Yes |
| 106 | PDE2A |  |  | 15480 | -8.300 | -0.2541 | Yes |
| 107 | SMPD3 |  |  | 15555 | -9.500 | -0.2388 | Yes |
| 108 | NR1I2 |  |  | 15578 | -9.700 | -0.2197 | Yes |
| 109 | SST |  |  | 15599 | -10.200 | -0.1995 | Yes |
| 110 | LAMB3 |  |  | 15671 | -12.700 | -0.1772 | Yes |
| 111 | CLDN7 |  |  | 15700 | -15.300 | -0.1467 | Yes |
| 112 | CNN1 |  |  | 15718 | -16.900 | -0.1121 | Yes |
| 113 | KRT7 |  |  | 15742 | -26.100 | -0.0585 | Yes |
| 114 | ISYNA1 |  |  | 15744 | -27.900 | 0.0003 | Yes |
Table: GSEA details [plain text format]

  

Fig 2: KIM\_RESPONSE\_TO\_TSA\_AND\_DECITABINE\_UP: Random ES distribution      
 Gene set null distribution of ES for **KIM\_RESPONSE\_TO\_TSA\_AND\_DECITABINE\_UP**

  
