# Supplementary material for: Immunomic, genomic and transcriptomic characterization of CT26 colorectal carcinoma
Source: BMC Genomics. 2014 Mar 13;15(1):190. doi: 10.1186/1471-2164-15-190 (PMC4007559; doi:10.1186/1471-2164-15-190)
Supplement: Supplementary file 8 — Additional file 8: Contains the Gene Pattern gene set membership and enrichment values in an html format. The file index.html is the entry point. (ZIP 13 MB) [file 12864_2013_7028_MOESM8_ESM.zip › MORI_IMMATURE_B_LYMPHOCYTE_DN.html]

Details for gene set MORI\_IMMATURE\_B\_LYMPHOCYTE\_DN[GSEA]

|  || Dataset | CT26\_gene\_expression |
| Phenotype | NoPhenotypeAvailable |
| Upregulated in class | na\_pos |
| GeneSet | MORI\_IMMATURE\_B\_LYMPHOCYTE\_DN |
| Enrichment Score (ES) | 0.79758394 |
| Normalized Enrichment Score (NES) | 1.7343172 |
| Nominal p-value | 0.0 |
| FDR q-value | 0.001125515 |
| FWER p-Value | 0.0050 |
Table: GSEA Results Summary

  

Fig 1: Enrichment plot: MORI\_IMMATURE\_B\_LYMPHOCYTE\_DN      
 Profile of the Running ES Score & Positions of GeneSet Members on the Rank Ordered List

  

| PROBE | GENE SYMBOL | GENE\_TITLE | RANK IN GENE LIST | RANK METRIC SCORE | RUNNING ES | CORE ENRICHMENT || 1 | SMC4 |  |  | 1 | 76.300 | 0.0573 | Yes |
| 2 | TOP2A |  |  | 8 | 56.000 | 0.0990 | Yes |
| 3 | CKS1B |  |  | 48 | 35.600 | 0.1232 | Yes |
| 4 | LIG1 |  |  | 104 | 30.000 | 0.1422 | Yes |
| 5 | PRIM1 |  |  | 116 | 29.000 | 0.1633 | Yes |
| 6 | CALM2 |  |  | 124 | 28.300 | 0.1841 | Yes |
| 7 | ANP32E |  |  | 135 | 27.700 | 0.2043 | Yes |
| 8 | RRM1 |  |  | 138 | 27.500 | 0.2248 | Yes |
| 9 | TUBB |  |  | 153 | 26.800 | 0.2441 | Yes |
| 10 | LGALS1 |  |  | 158 | 26.700 | 0.2639 | Yes |
| 11 | MCM6 |  |  | 163 | 26.600 | 0.2836 | Yes |
| 12 | CCNA2 |  |  | 178 | 25.600 | 0.3020 | Yes |
| 13 | PRC1 |  |  | 181 | 25.500 | 0.3210 | Yes |
| 14 | TMPO |  |  | 203 | 24.500 | 0.3381 | Yes |
| 15 | CKAP5 |  |  | 210 | 24.300 | 0.3559 | Yes |
| 16 | HMMR |  |  | 246 | 23.200 | 0.3711 | Yes |
| 17 | MTHFD2 |  |  | 255 | 22.900 | 0.3878 | Yes |
| 18 | CDC20 |  |  | 284 | 22.200 | 0.4027 | Yes |
| 19 | EZH2 |  |  | 286 | 22.000 | 0.4192 | Yes |
| 20 | MCM7 |  |  | 290 | 21.900 | 0.4354 | Yes |
| 21 | MCM3 |  |  | 315 | 21.400 | 0.4500 | Yes |
| 22 | HMGB1 |  |  | 323 | 21.200 | 0.4655 | Yes |
| 23 | NEK2 |  |  | 346 | 20.900 | 0.4798 | Yes |
| 24 | KPNA2 |  |  | 354 | 20.700 | 0.4949 | Yes |
| 25 | CDCA7 |  |  | 356 | 20.700 | 0.5104 | Yes |
| 26 | SMC2 |  |  | 379 | 20.300 | 0.5242 | Yes |
| 27 | PCNA |  |  | 386 | 20.200 | 0.5390 | Yes |
| 28 | MKI67 |  |  | 414 | 19.700 | 0.5521 | Yes |
| 29 | RAD51 |  |  | 427 | 19.400 | 0.5659 | Yes |
| 30 | NCAPH |  |  | 459 | 19.000 | 0.5782 | Yes |
| 31 | DNMT1 |  |  | 555 | 17.900 | 0.5856 | Yes |
| 32 | DUT |  |  | 658 | 16.800 | 0.5917 | Yes |
| 33 | NUSAP1 |  |  | 716 | 16.200 | 0.6002 | Yes |
| 34 | RAN |  |  | 752 | 15.900 | 0.6099 | Yes |
| 35 | HMGN2 |  |  | 822 | 15.400 | 0.6171 | Yes |
| 36 | XRCC6 |  |  | 827 | 15.400 | 0.6284 | Yes |
| 37 | DTL |  |  | 936 | 14.700 | 0.6326 | Yes |
| 38 | MELK |  |  | 1077 | 13.700 | 0.6339 | Yes |
| 39 | RRM2 |  |  | 1084 | 13.700 | 0.6438 | Yes |
| 40 | PREP |  |  | 1098 | 13.600 | 0.6532 | Yes |
| 41 | CCNB2 |  |  | 1155 | 13.200 | 0.6596 | Yes |
| 42 | UCK2 |  |  | 1161 | 13.200 | 0.6692 | Yes |
| 43 | RBBP4 |  |  | 1179 | 13.100 | 0.6779 | Yes |
| 44 | E2F8 |  |  | 1210 | 13.000 | 0.6858 | Yes |
| 45 | PRDX4 |  |  | 1246 | 12.800 | 0.6931 | Yes |
| 46 | MCM10 |  |  | 1264 | 12.700 | 0.7016 | Yes |
| 47 | HMGB2 |  |  | 1279 | 12.700 | 0.7103 | Yes |
| 48 | MCM2 |  |  | 1281 | 12.700 | 0.7197 | Yes |
| 49 | H2AFV |  |  | 1310 | 12.500 | 0.7273 | Yes |
| 50 | SMARCA5 |  |  | 1341 | 12.300 | 0.7347 | Yes |
| 51 | TTK |  |  | 1430 | 11.900 | 0.7380 | Yes |
| 52 | DCK |  |  | 1603 | 11.200 | 0.7354 | Yes |
| 53 | PCK2 |  |  | 1665 | 11.000 | 0.7398 | Yes |
| 54 | CENPA |  |  | 1762 | 10.600 | 0.7416 | Yes |
| 55 | TUBA1A |  |  | 1792 | 10.500 | 0.7477 | Yes |
| 56 | CDCA5 |  |  | 1813 | 10.400 | 0.7542 | Yes |
| 57 | LMNB1 |  |  | 1916 | 10.000 | 0.7552 | Yes |
| 58 | SLBP |  |  | 1951 | 9.900 | 0.7605 | Yes |
| 59 | CDKN2C |  |  | 1988 | 9.700 | 0.7655 | Yes |
| 60 | HMGB3 |  |  | 1996 | 9.700 | 0.7723 | Yes |
| 61 | U2AF1 |  |  | 2010 | 9.600 | 0.7787 | Yes |
| 62 | STMN1 |  |  | 2088 | 9.400 | 0.7808 | Yes |
| 63 | IQGAP3 |  |  | 2092 | 9.400 | 0.7877 | Yes |
| 64 | CDKN1A |  |  | 2165 | 9.100 | 0.7899 | Yes |
| 65 | CENPL |  |  | 2261 | 8.900 | 0.7906 | Yes |
| 66 | CCNB1 |  |  | 2266 | 8.900 | 0.7970 | Yes |
| 67 | CBX1 |  |  | 2359 | 8.600 | 0.7976 | Yes |
| 68 | CDCA3 |  |  | 2591 | 7.900 | 0.7888 | No |
| 69 | SMARCA4 |  |  | 2647 | 7.800 | 0.7911 | No |
| 70 | UBE2C |  |  | 2879 | 7.200 | 0.7818 | No |
| 71 | CDKN3 |  |  | 2917 | 7.100 | 0.7848 | No |
| 72 | RAD54L |  |  | 3132 | 6.600 | 0.7761 | No |
| 73 | CHEK1 |  |  | 3593 | 5.600 | 0.7509 | No |
| 74 | SLC29A1 |  |  | 3927 | 4.900 | 0.7333 | No |
| 75 | SLC16A1 |  |  | 4053 | 4.700 | 0.7289 | No |
| 76 | TXN |  |  | 5081 | 2.900 | 0.6655 | No |
| 77 | ACTN4 |  |  | 5359 | 2.500 | 0.6497 | No |
| 78 | RAG1 |  |  | 8983 | 0.000 | 0.4184 | No |
| 79 | IGLL1 |  |  | 9066 | 0.000 | 0.4132 | No |
| 80 | CCND3 |  |  | 11484 | -0.400 | 0.2592 | No |
| 81 | ENPEP |  |  | 12224 | -0.800 | 0.2126 | No |
| 82 | H2AFX |  |  | 14159 | -3.000 | 0.0914 | No |
| 83 | GSN |  |  | 15151 | -5.900 | 0.0326 | No |
| 84 | RAMP1 |  |  | 15374 | -7.300 | 0.0239 | No |
Table: GSEA details [plain text format]

  

Fig 2: MORI\_IMMATURE\_B\_LYMPHOCYTE\_DN: Random ES distribution      
 Gene set null distribution of ES for **MORI\_IMMATURE\_B\_LYMPHOCYTE\_DN**

  
