# Supplementary material for: Immunomic, genomic and transcriptomic characterization of CT26 colorectal carcinoma
Source: BMC Genomics. 2014 Mar 13;15(1):190. doi: 10.1186/1471-2164-15-190 (PMC4007559; doi:10.1186/1471-2164-15-190)
Supplement: Supplementary file 8 — Additional file 8: Contains the Gene Pattern gene set membership and enrichment values in an html format. The file index.html is the entry point. (ZIP 13 MB) [file 12864_2013_7028_MOESM8_ESM.zip › MORI_LARGE_PRE_BII_LYMPHOCYTE_UP.html]

Details for gene set MORI\_LARGE\_PRE\_BII\_LYMPHOCYTE\_UP[GSEA]

|  || Dataset | CT26\_gene\_expression |
| Phenotype | NoPhenotypeAvailable |
| Upregulated in class | na\_pos |
| GeneSet | MORI\_LARGE\_PRE\_BII\_LYMPHOCYTE\_UP |
| Enrichment Score (ES) | 0.8166424 |
| Normalized Enrichment Score (NES) | 1.7875162 |
| Nominal p-value | 0.0 |
| FDR q-value | 8.986061E-4 |
| FWER p-Value | 0.0020 |
Table: GSEA Results Summary

  

Fig 1: Enrichment plot: MORI\_LARGE\_PRE\_BII\_LYMPHOCYTE\_UP      
 Profile of the Running ES Score & Positions of GeneSet Members on the Rank Ordered List

  

| PROBE | GENE SYMBOL | GENE\_TITLE | RANK IN GENE LIST | RANK METRIC SCORE | RUNNING ES | CORE ENRICHMENT || 1 | SMC4 |  |  | 1 | 76.300 | 0.0569 | Yes |
| 2 | TOP2A |  |  | 8 | 56.000 | 0.0983 | Yes |
| 3 | ANAPC5 |  |  | 38 | 37.900 | 0.1247 | Yes |
| 4 | CKS1B |  |  | 48 | 35.600 | 0.1507 | Yes |
| 5 | RAD21 |  |  | 86 | 31.400 | 0.1718 | Yes |
| 6 | LIG1 |  |  | 104 | 30.000 | 0.1931 | Yes |
| 7 | CALM2 |  |  | 124 | 28.300 | 0.2130 | Yes |
| 8 | ANP32E |  |  | 135 | 27.700 | 0.2330 | Yes |
| 9 | RRM1 |  |  | 138 | 27.500 | 0.2534 | Yes |
| 10 | TUBB |  |  | 153 | 26.800 | 0.2725 | Yes |
| 11 | LGALS1 |  |  | 158 | 26.700 | 0.2922 | Yes |
| 12 | MCM6 |  |  | 163 | 26.600 | 0.3118 | Yes |
| 13 | UBE2S |  |  | 177 | 25.600 | 0.3301 | Yes |
| 14 | CCNA2 |  |  | 178 | 25.600 | 0.3492 | Yes |
| 15 | PRC1 |  |  | 181 | 25.500 | 0.3681 | Yes |
| 16 | TMPO |  |  | 203 | 24.500 | 0.3850 | Yes |
| 17 | RANGAP1 |  |  | 220 | 24.000 | 0.4019 | Yes |
| 18 | HMMR |  |  | 246 | 23.200 | 0.4177 | Yes |
| 19 | CDC20 |  |  | 284 | 22.200 | 0.4319 | Yes |
| 20 | MCM7 |  |  | 290 | 21.900 | 0.4479 | Yes |
| 21 | MCM3 |  |  | 315 | 21.400 | 0.4623 | Yes |
| 22 | HMGB1 |  |  | 323 | 21.200 | 0.4777 | Yes |
| 23 | KIF23 |  |  | 337 | 21.000 | 0.4925 | Yes |
| 24 | KPNA2 |  |  | 354 | 20.700 | 0.5070 | Yes |
| 25 | CDCA7 |  |  | 356 | 20.700 | 0.5224 | Yes |
| 26 | SMC2 |  |  | 379 | 20.300 | 0.5361 | Yes |
| 27 | CENPE |  |  | 391 | 20.100 | 0.5504 | Yes |
| 28 | MKI67 |  |  | 414 | 19.700 | 0.5637 | Yes |
| 29 | NCAPH |  |  | 459 | 19.000 | 0.5751 | Yes |
| 30 | KIF22 |  |  | 461 | 18.900 | 0.5891 | Yes |
| 31 | PSMC1 |  |  | 493 | 18.600 | 0.6010 | Yes |
| 32 | IDE |  |  | 521 | 18.300 | 0.6130 | Yes |
| 33 | BUB3 |  |  | 533 | 18.100 | 0.6258 | Yes |
| 34 | DUT |  |  | 658 | 16.800 | 0.6304 | Yes |
| 35 | RACGAP1 |  |  | 682 | 16.600 | 0.6413 | Yes |
| 36 | RAN |  |  | 752 | 15.900 | 0.6488 | Yes |
| 37 | AURKA |  |  | 799 | 15.600 | 0.6575 | Yes |
| 38 | HMGN2 |  |  | 822 | 15.400 | 0.6676 | Yes |
| 39 | XRCC6 |  |  | 827 | 15.400 | 0.6788 | Yes |
| 40 | DTL |  |  | 936 | 14.700 | 0.6829 | Yes |
| 41 | MELK |  |  | 1077 | 13.700 | 0.6842 | Yes |
| 42 | RRM2 |  |  | 1084 | 13.700 | 0.6940 | Yes |
| 43 | CCNB2 |  |  | 1155 | 13.200 | 0.6994 | Yes |
| 44 | RBBP4 |  |  | 1179 | 13.100 | 0.7077 | Yes |
| 45 | E2F8 |  |  | 1210 | 13.000 | 0.7155 | Yes |
| 46 | PRDX4 |  |  | 1246 | 12.800 | 0.7228 | Yes |
| 47 | MCM2 |  |  | 1281 | 12.700 | 0.7301 | Yes |
| 48 | H2AFV |  |  | 1310 | 12.500 | 0.7377 | Yes |
| 49 | PPP2R5C |  |  | 1333 | 12.400 | 0.7455 | Yes |
| 50 | ASF1B |  |  | 1372 | 12.200 | 0.7522 | Yes |
| 51 | TTK |  |  | 1430 | 11.900 | 0.7574 | Yes |
| 52 | MCM5 |  |  | 1526 | 11.500 | 0.7600 | Yes |
| 53 | CENPA |  |  | 1762 | 10.600 | 0.7529 | Yes |
| 54 | TUBA1A |  |  | 1792 | 10.500 | 0.7589 | Yes |
| 55 | NUCKS1 |  |  | 1798 | 10.400 | 0.7663 | Yes |
| 56 | CDCA5 |  |  | 1813 | 10.400 | 0.7732 | Yes |
| 57 | SMARCC1 |  |  | 1873 | 10.200 | 0.7770 | Yes |
| 58 | LMNB1 |  |  | 1916 | 10.000 | 0.7818 | Yes |
| 59 | SLBP |  |  | 1951 | 9.900 | 0.7870 | Yes |
| 60 | CDKN2C |  |  | 1988 | 9.700 | 0.7920 | Yes |
| 61 | HMGB3 |  |  | 1996 | 9.700 | 0.7988 | Yes |
| 62 | MYBL2 |  |  | 2007 | 9.600 | 0.8053 | Yes |
| 63 | STMN1 |  |  | 2088 | 9.400 | 0.8072 | Yes |
| 64 | CDKN1A |  |  | 2165 | 9.100 | 0.8091 | Yes |
| 65 | CENPL |  |  | 2261 | 8.900 | 0.8097 | Yes |
| 66 | CCNB1 |  |  | 2266 | 8.900 | 0.8161 | Yes |
| 67 | CBX1 |  |  | 2359 | 8.600 | 0.8166 | Yes |
| 68 | CDCA3 |  |  | 2591 | 7.900 | 0.8078 | No |
| 69 | SMARCA4 |  |  | 2647 | 7.800 | 0.8101 | No |
| 70 | CDKN3 |  |  | 2917 | 7.100 | 0.7982 | No |
| 71 | SLC29A1 |  |  | 3927 | 4.900 | 0.7375 | No |
| 72 | THOC4 |  |  | 4495 | 3.900 | 0.7042 | No |
| 73 | TXN |  |  | 5081 | 2.900 | 0.6691 | No |
| 74 | FANCC |  |  | 6296 | 1.200 | 0.5925 | No |
| 75 | LGALS9 |  |  | 8006 | 0.000 | 0.4834 | No |
| 76 | IGLL1 |  |  | 9066 | 0.000 | 0.4158 | No |
| 77 | SLC12A3 |  |  | 9217 | 0.000 | 0.4062 | No |
| 78 | ENPEP |  |  | 12224 | -0.800 | 0.2150 | No |
| 79 | ZNF358 |  |  | 14024 | -2.800 | 0.1023 | No |
| 80 | H2AFX |  |  | 14159 | -3.000 | 0.0960 | No |
| 81 | RAMP1 |  |  | 15374 | -7.300 | 0.0239 | No |
Table: GSEA details [plain text format]

  

Fig 2: MORI\_LARGE\_PRE\_BII\_LYMPHOCYTE\_UP: Random ES distribution      
 Gene set null distribution of ES for **MORI\_LARGE\_PRE\_BII\_LYMPHOCYTE\_UP**

  
