# Supplementary material for: Immunomic, genomic and transcriptomic characterization of CT26 colorectal carcinoma
Source: BMC Genomics. 2014 Mar 13;15(1):190. doi: 10.1186/1471-2164-15-190 (PMC4007559; doi:10.1186/1471-2164-15-190)
Supplement: Supplementary file 8 — Additional file 8: Contains the Gene Pattern gene set membership and enrichment values in an html format. The file index.html is the entry point. (ZIP 13 MB) [file 12864_2013_7028_MOESM8_ESM.zip › PID_AURORA_B_PATHWAY.html]

Details for gene set PID\_AURORA\_B\_PATHWAY[GSEA]

|  || Dataset | CT26\_gene\_expression |
| Phenotype | NoPhenotypeAvailable |
| Upregulated in class | na\_pos |
| GeneSet | PID\_AURORA\_B\_PATHWAY |
| Enrichment Score (ES) | 0.82327396 |
| Normalized Enrichment Score (NES) | 1.7106273 |
| Nominal p-value | 0.0 |
| FDR q-value | 0.0014400368 |
| FWER p-Value | 0.016 |
Table: GSEA Results Summary

  

Fig 1: Enrichment plot: PID\_AURORA\_B\_PATHWAY      
 Profile of the Running ES Score & Positions of GeneSet Members on the Rank Ordered List

  

| PROBE | GENE SYMBOL | GENE\_TITLE | RANK IN GENE LIST | RANK METRIC SCORE | RUNNING ES | CORE ENRICHMENT || 1 | SMC4 |  |  | 1 | 76.300 | 0.1115 | Yes |
| 2 | NPM1 |  |  | 36 | 38.000 | 0.1649 | Yes |
| 3 | NSUN2 |  |  | 42 | 37.100 | 0.2188 | Yes |
| 4 | NCAPD2 |  |  | 55 | 35.000 | 0.2692 | Yes |
| 5 | CUL3 |  |  | 63 | 33.800 | 0.3181 | Yes |
| 6 | KIF20A |  |  | 77 | 31.900 | 0.3639 | Yes |
| 7 | PSMA3 |  |  | 245 | 23.200 | 0.3872 | Yes |
| 8 | INCENP |  |  | 260 | 22.700 | 0.4195 | Yes |
| 9 | KIF23 |  |  | 337 | 21.000 | 0.4454 | Yes |
| 10 | BUB1 |  |  | 360 | 20.600 | 0.4741 | Yes |
| 11 | SMC2 |  |  | 379 | 20.300 | 0.5026 | Yes |
| 12 | NCAPH |  |  | 459 | 19.000 | 0.5254 | Yes |
| 13 | VIM |  |  | 503 | 18.500 | 0.5497 | Yes |
| 14 | BIRC5 |  |  | 507 | 18.400 | 0.5764 | Yes |
| 15 | NCL |  |  | 508 | 18.400 | 0.6033 | Yes |
| 16 | NCAPG |  |  | 524 | 18.200 | 0.6289 | Yes |
| 17 | PPP2R5D |  |  | 607 | 17.300 | 0.6490 | Yes |
| 18 | RACGAP1 |  |  | 682 | 16.600 | 0.6685 | Yes |
| 19 | RASA1 |  |  | 696 | 16.400 | 0.6917 | Yes |
| 20 | AURKA |  |  | 799 | 15.600 | 0.7080 | Yes |
| 21 | SGOL1 |  |  | 857 | 15.100 | 0.7264 | Yes |
| 22 | NDC80 |  |  | 878 | 15.000 | 0.7471 | Yes |
| 23 | RHOA |  |  | 956 | 14.500 | 0.7634 | Yes |
| 24 | CDCA8 |  |  | 1017 | 14.100 | 0.7802 | Yes |
| 25 | KIF2C |  |  | 1036 | 14.000 | 0.7995 | Yes |
| 26 | CBX5 |  |  | 1058 | 13.800 | 0.8183 | Yes |
| 27 | PEBP1 |  |  | 1458 | 11.800 | 0.8102 | Yes |
| 28 | EVI5 |  |  | 1525 | 11.500 | 0.8228 | Yes |
| 29 | CENPA |  |  | 1762 | 10.600 | 0.8233 | Yes |
| 30 | STMN1 |  |  | 2088 | 9.400 | 0.8163 | No |
| 31 | AURKB |  |  | 2822 | 7.300 | 0.7803 | No |
| 32 | PPP1CC |  |  | 4111 | 4.600 | 0.7051 | No |
| 33 | KLHL9 |  |  | 4189 | 4.400 | 0.7066 | No |
| 34 | TACC1 |  |  | 6521 | 0.900 | 0.5596 | No |
| 35 | AURKC |  |  | 7817 | 0.000 | 0.4772 | No |
| 36 | KLHL13 |  |  | 11392 | -0.300 | 0.2501 | No |
| 37 | MYLK |  |  | 14958 | -5.100 | 0.0307 | No |
| 38 | DES |  |  | 15681 | -13.400 | 0.0043 | No |
Table: GSEA details [plain text format]

  

Fig 2: PID\_AURORA\_B\_PATHWAY: Random ES distribution      
 Gene set null distribution of ES for **PID\_AURORA\_B\_PATHWAY**

  
