# Supplementary material for: Immunomic, genomic and transcriptomic characterization of CT26 colorectal carcinoma
Source: BMC Genomics. 2014 Mar 13;15(1):190. doi: 10.1186/1471-2164-15-190 (PMC4007559; doi:10.1186/1471-2164-15-190)
Supplement: Supplementary file 8 — Additional file 8: Contains the Gene Pattern gene set membership and enrichment values in an html format. The file index.html is the entry point. (ZIP 13 MB) [file 12864_2013_7028_MOESM8_ESM.zip › REACTOME_BIOLOGICAL_OXIDATIONS.html]

Details for gene set REACTOME\_BIOLOGICAL\_OXIDATIONS[GSEA]

|  || Dataset | CT26\_gene\_expression |
| Phenotype | NoPhenotypeAvailable |
| Upregulated in class | na\_neg |
| GeneSet | REACTOME\_BIOLOGICAL\_OXIDATIONS |
| Enrichment Score (ES) | -0.28208348 |
| Normalized Enrichment Score (NES) | NaN |
| Nominal p-value | NaN |
| FDR q-value | 1.0 |
| FWER p-Value | 0.0 |
Table: GSEA Results Summary

  

Fig 1: Enrichment plot: REACTOME\_BIOLOGICAL\_OXIDATIONS      
 Profile of the Running ES Score & Positions of GeneSet Members on the Rank Ordered List

  

| PROBE | GENE SYMBOL | GENE\_TITLE | RANK IN GENE LIST | RANK METRIC SCORE | RUNNING ES | CORE ENRICHMENT || 1 | UGDH |  |  | 76 | 32.000 | 0.0740 | No |
| 2 | CYP51A1 |  |  | 390 | 20.100 | 0.1035 | No |
| 3 | PTGS2 |  |  | 421 | 19.600 | 0.1499 | No |
| 4 | ADH7 |  |  | 698 | 16.400 | 0.1727 | No |
| 5 | MAT2A |  |  | 1336 | 12.400 | 0.1625 | No |
| 6 | PAPSS1 |  |  | 1619 | 11.200 | 0.1721 | No |
| 7 | PTGS1 |  |  | 1732 | 10.700 | 0.1913 | No |
| 8 | UGP2 |  |  | 1799 | 10.400 | 0.2127 | No |
| 9 | MAT2B |  |  | 1891 | 10.100 | 0.2318 | No |
| 10 | GSTO1 |  |  | 1995 | 9.700 | 0.2491 | No |
| 11 | GCLC |  |  | 2200 | 9.000 | 0.2582 | No |
| 12 | GSTA4 |  |  | 2779 | 7.400 | 0.2395 | No |
| 13 | AHCY |  |  | 3109 | 6.600 | 0.2347 | No |
| 14 | NNMT |  |  | 3216 | 6.400 | 0.2437 | No |
| 15 | MGST2 |  |  | 3855 | 5.100 | 0.2155 | No |
| 16 | MTR |  |  | 3857 | 5.000 | 0.2277 | No |
| 17 | GSTP1 |  |  | 4141 | 4.500 | 0.2207 | No |
| 18 | OPLAH |  |  | 4314 | 4.200 | 0.2201 | No |
| 19 | CYP39A1 |  |  | 4491 | 3.900 | 0.2184 | No |
| 20 | MAOA |  |  | 4971 | 3.100 | 0.1954 | No |
| 21 | CNDP2 |  |  | 5188 | 2.700 | 0.1883 | No |
| 22 | SMOX |  |  | 5654 | 2.100 | 0.1637 | No |
| 23 | NAT1 |  |  | 5674 | 2.100 | 0.1677 | No |
| 24 | TPMT |  |  | 6110 | 1.500 | 0.1435 | No |
| 25 | GSTO2 |  |  | 6162 | 1.400 | 0.1437 | No |
| 26 | BPNT1 |  |  | 6343 | 1.100 | 0.1349 | No |
| 27 | GCLM |  |  | 6379 | 1.100 | 0.1354 | No |
| 28 | CYP27B1 |  |  | 6800 | 0.600 | 0.1100 | No |
| 29 | SLC35D1 |  |  | 7302 | 0.100 | 0.0782 | No |
| 30 | CYP46A1 |  |  | 7339 | 0.100 | 0.0762 | No |
| 31 | CYP2U1 |  |  | 7372 | 0.100 | 0.0744 | No |
| 32 | GSTA5 |  |  | 7539 | 0.000 | 0.0637 | No |
| 33 | NAT2 |  |  | 7722 | 0.000 | 0.0521 | No |
| 34 | CYP26A1 |  |  | 7774 | 0.000 | 0.0488 | No |
| 35 | CYP3A7 |  |  | 7820 | 0.000 | 0.0459 | No |
| 36 | UGT2B17 |  |  | 7831 | 0.000 | 0.0453 | No |
| 37 | CYP7A1 |  |  | 7872 | 0.000 | 0.0428 | No |
| 38 | ADH4 |  |  | 7898 | 0.000 | 0.0412 | No |
| 39 | CYP1A1 |  |  | 7946 | 0.000 | 0.0381 | No |
| 40 | CYP26C1 |  |  | 7975 | 0.000 | 0.0364 | No |
| 41 | CYP24A1 |  |  | 8019 | 0.000 | 0.0336 | No |
| 42 | CYP3A4 |  |  | 8023 | 0.000 | 0.0334 | No |
| 43 | CYP17A1 |  |  | 8034 | 0.000 | 0.0328 | No |
| 44 | CYP11B2 |  |  | 8197 | 0.000 | 0.0224 | No |
| 45 | CYP11B1 |  |  | 8198 | 0.000 | 0.0224 | No |
| 46 | CYP19A1 |  |  | 8199 | 0.000 | 0.0224 | No |
| 47 | CYP2B6 |  |  | 8200 | 0.000 | 0.0224 | No |
| 48 | CYP2C19 |  |  | 8201 | 0.000 | 0.0224 | No |
| 49 | GLYAT |  |  | 8298 | 0.000 | 0.0163 | No |
| 50 | SULT1E1 |  |  | 8869 | 0.000 | -0.0202 | No |
| 51 | SULT2A1 |  |  | 8870 | 0.000 | -0.0202 | No |
| 52 | CYP2A13 |  |  | 9027 | 0.000 | -0.0302 | No |
| 53 | CYP1A2 |  |  | 9065 | 0.000 | -0.0325 | No |
| 54 | FMO3 |  |  | 9073 | 0.000 | -0.0330 | No |
| 55 | CYP4F8 |  |  | 9078 | 0.000 | -0.0332 | No |
| 56 | ACSM1 |  |  | 9094 | 0.000 | -0.0342 | No |
| 57 | UGT2A1 |  |  | 9203 | 0.000 | -0.0411 | No |
| 58 | CYP4A11 |  |  | 9242 | 0.000 | -0.0435 | No |
| 59 | CYP2C9 |  |  | 9267 | 0.000 | -0.0451 | No |
| 60 | UGT2B28 |  |  | 9329 | 0.000 | -0.0490 | No |
| 61 | CYP11A1 |  |  | 9339 | 0.000 | -0.0496 | No |
| 62 | CYP21A2 |  |  | 9416 | 0.000 | -0.0544 | No |
| 63 | CYP3A43 |  |  | 9678 | 0.000 | -0.0711 | No |
| 64 | CYP8B1 |  |  | 10168 | -0.100 | -0.1021 | No |
| 65 | MAT1A |  |  | 10178 | -0.100 | -0.1025 | No |
| 66 | TBXAS1 |  |  | 10196 | -0.100 | -0.1033 | No |
| 67 | UGT2B7 |  |  | 10236 | -0.100 | -0.1056 | No |
| 68 | CYP1B1 |  |  | 10464 | -0.100 | -0.1198 | No |
| 69 | POMC |  |  | 10894 | -0.200 | -0.1468 | No |
| 70 | GGT1 |  |  | 11138 | -0.200 | -0.1618 | No |
| 71 | CYP7B1 |  |  | 11388 | -0.300 | -0.1770 | No |
| 72 | FMO2 |  |  | 11411 | -0.300 | -0.1777 | No |
| 73 | CYP2R1 |  |  | 11493 | -0.400 | -0.1819 | No |
| 74 | CYP26B1 |  |  | 11524 | -0.400 | -0.1828 | No |
| 75 | CYP2E1 |  |  | 11701 | -0.500 | -0.1928 | No |
| 76 | PAOX |  |  | 11719 | -0.500 | -0.1927 | No |
| 77 | FMO1 |  |  | 11729 | -0.500 | -0.1920 | No |
| 78 | GSTA3 |  |  | 12030 | -0.700 | -0.2095 | No |
| 79 | MAOB |  |  | 12706 | -1.100 | -0.2500 | No |
| 80 | SULT1B1 |  |  | 12899 | -1.300 | -0.2591 | No |
| 81 | COMT |  |  | 12975 | -1.400 | -0.2604 | No |
| 82 | ACSS2 |  |  | 13050 | -1.500 | -0.2614 | No |
| 83 | SULT4A1 |  |  | 13114 | -1.600 | -0.2615 | No |
| 84 | CYP2J2 |  |  | 13397 | -1.900 | -0.2749 | No |
| 85 | CYP3A5 |  |  | 13415 | -1.900 | -0.2713 | No |
| 86 | CYP4F3 |  |  | 13496 | -2.000 | -0.2715 | No |
| 87 | CYP4B1 |  |  | 13578 | -2.100 | -0.2715 | No |
| 88 | CYP2C8 |  |  | 13595 | -2.100 | -0.2673 | No |
| 89 | SULT1C2 |  |  | 13650 | -2.200 | -0.2654 | No |
| 90 | UGT1A6 |  |  | 13764 | -2.400 | -0.2667 | No |
| 91 | UGT1A1 |  |  | 14006 | -2.800 | -0.2752 | Yes |
| 92 | CYP2C18 |  |  | 14105 | -3.000 | -0.2741 | Yes |
| 93 | UGT1A3 |  |  | 14131 | -3.000 | -0.2683 | Yes |
| 94 | UGT1A7 |  |  | 14137 | -3.000 | -0.2612 | Yes |
| 95 | UGT1A4 |  |  | 14140 | -3.000 | -0.2539 | Yes |
| 96 | ALDH2 |  |  | 14145 | -3.000 | -0.2468 | Yes |
| 97 | GSTM1 |  |  | 14300 | -3.300 | -0.2485 | Yes |
| 98 | CYP2W1 |  |  | 14493 | -3.800 | -0.2514 | Yes |
| 99 | GSTM4 |  |  | 14539 | -3.900 | -0.2447 | Yes |
| 100 | GSTM5 |  |  | 14612 | -4.100 | -0.2392 | Yes |
| 101 | GSS |  |  | 14700 | -4.300 | -0.2341 | Yes |
| 102 | MGST1 |  |  | 14718 | -4.400 | -0.2244 | Yes |
| 103 | PTGIS |  |  | 14729 | -4.400 | -0.2142 | Yes |
| 104 | CYP2F1 |  |  | 14772 | -4.500 | -0.2058 | Yes |
| 105 | ALDH1A1 |  |  | 14938 | -5.000 | -0.2040 | Yes |
| 106 | CYP4F12 |  |  | 14991 | -5.200 | -0.1945 | Yes |
| 107 | CYP27A1 |  |  | 15066 | -5.500 | -0.1857 | Yes |
| 108 | ACSS1 |  |  | 15247 | -6.400 | -0.1814 | Yes |
| 109 | CYP2S1 |  |  | 15383 | -7.300 | -0.1721 | Yes |
| 110 | UGT2B10 |  |  | 15398 | -7.500 | -0.1545 | Yes |
| 111 | ADH1C |  |  | 15410 | -7.600 | -0.1365 | Yes |
| 112 | SULT2B1 |  |  | 15411 | -7.600 | -0.1177 | Yes |
| 113 | CYP2D6 |  |  | 15438 | -7.900 | -0.0999 | Yes |
| 114 | PAPSS2 |  |  | 15445 | -7.900 | -0.0808 | Yes |
| 115 | CYP4F2 |  |  | 15546 | -9.300 | -0.0643 | Yes |
| 116 | SULT1A1 |  |  | 15697 | -14.900 | -0.0372 | Yes |
| 117 | MGST3 |  |  | 15712 | -16.400 | 0.0024 | Yes |
Table: GSEA details [plain text format]

  

Fig 2: REACTOME\_BIOLOGICAL\_OXIDATIONS: Random ES distribution      
 Gene set null distribution of ES for **REACTOME\_BIOLOGICAL\_OXIDATIONS**

  
