# Supplementary material for: Immunomic, genomic and transcriptomic characterization of CT26 colorectal carcinoma
Source: BMC Genomics. 2014 Mar 13;15(1):190. doi: 10.1186/1471-2164-15-190 (PMC4007559; doi:10.1186/1471-2164-15-190)
Supplement: Supplementary file 8 — Additional file 8: Contains the Gene Pattern gene set membership and enrichment values in an html format. The file index.html is the entry point. (ZIP 13 MB) [file 12864_2013_7028_MOESM8_ESM.zip › REACTOME_CLASS_A1_RHODOPSIN_LIKE_RECEPTORS.html]

Details for gene set REACTOME\_CLASS\_A1\_RHODOPSIN\_LIKE\_RECEPTORS[GSEA]

|  || Dataset | CT26\_gene\_expression |
| Phenotype | NoPhenotypeAvailable |
| Upregulated in class | na\_neg |
| GeneSet | REACTOME\_CLASS\_A1\_RHODOPSIN\_LIKE\_RECEPTORS |
| Enrichment Score (ES) | -0.54768115 |
| Normalized Enrichment Score (NES) | NaN |
| Nominal p-value | NaN |
| FDR q-value | 1.0 |
| FWER p-Value | 0.0 |
Table: GSEA Results Summary

  

Fig 1: Enrichment plot: REACTOME\_CLASS\_A1\_RHODOPSIN\_LIKE\_RECEPTORS      
 Profile of the Running ES Score & Positions of GeneSet Members on the Rank Ordered List

  

| PROBE | GENE SYMBOL | GENE\_TITLE | RANK IN GENE LIST | RANK METRIC SCORE | RUNNING ES | CORE ENRICHMENT || 1 | ANXA1 |  |  | 697 | 16.400 | 0.0092 | No |
| 2 | HEBP1 |  |  | 1978 | 9.700 | -0.0413 | No |
| 3 | F2R |  |  | 2440 | 8.400 | -0.0433 | No |
| 4 | PMCH |  |  | 3480 | 5.800 | -0.0912 | No |
| 5 | CCL25 |  |  | 5788 | 1.900 | -0.2338 | No |
| 6 | GNRH1 |  |  | 6126 | 1.500 | -0.2506 | No |
| 7 | PROKR1 |  |  | 6193 | 1.400 | -0.2503 | No |
| 8 | CXCL2 |  |  | 6388 | 1.100 | -0.2591 | No |
| 9 | CCR1 |  |  | 6492 | 0.900 | -0.2628 | No |
| 10 | GPR44 |  |  | 6826 | 0.600 | -0.2823 | No |
| 11 | ADRA2B |  |  | 7044 | 0.400 | -0.2950 | No |
| 12 | CXCL10 |  |  | 7089 | 0.300 | -0.2969 | No |
| 13 | NPFF |  |  | 7140 | 0.300 | -0.2991 | No |
| 14 | P2RY14 |  |  | 7245 | 0.200 | -0.3051 | No |
| 15 | NTSR2 |  |  | 7274 | 0.200 | -0.3063 | No |
| 16 | PPBP |  |  | 7319 | 0.100 | -0.3088 | No |
| 17 | LHB |  |  | 7411 | 0.100 | -0.3143 | No |
| 18 | OXT |  |  | 7433 | 0.100 | -0.3154 | No |
| 19 | ADRA1D |  |  | 7481 | 0.100 | -0.3181 | No |
| 20 | CCL17 |  |  | 7491 | 0.100 | -0.3183 | No |
| 21 | UTS2 |  |  | 7534 | 0.000 | -0.3210 | No |
| 22 | GHRL |  |  | 7558 | 0.000 | -0.3225 | No |
| 23 | MC3R |  |  | 7564 | 0.000 | -0.3228 | No |
| 24 | PROK1 |  |  | 7579 | 0.000 | -0.3237 | No |
| 25 | OPN1SW |  |  | 7624 | 0.000 | -0.3266 | No |
| 26 | TRHR |  |  | 7758 | 0.000 | -0.3352 | No |
| 27 | RXFP2 |  |  | 7821 | 0.000 | -0.3392 | No |
| 28 | NPS |  |  | 7825 | 0.000 | -0.3394 | No |
| 29 | HRH4 |  |  | 7848 | 0.000 | -0.3408 | No |
| 30 | PTGDR |  |  | 7881 | 0.000 | -0.3428 | No |
| 31 | MC1R |  |  | 8028 | 0.000 | -0.3523 | No |
| 32 | BRS3 |  |  | 8130 | 0.000 | -0.3588 | No |
| 33 | CGA |  |  | 8158 | 0.000 | -0.3605 | No |
| 34 | CHRM5 |  |  | 8159 | 0.000 | -0.3605 | No |
| 35 | DRD5 |  |  | 8229 | 0.000 | -0.3650 | No |
| 36 | FSHB |  |  | 8272 | 0.000 | -0.3677 | No |
| 37 | FSHR |  |  | 8273 | 0.000 | -0.3677 | No |
| 38 | GAST |  |  | 8284 | 0.000 | -0.3683 | No |
| 39 | GHSR |  |  | 8289 | 0.000 | -0.3686 | No |
| 40 | GNRHR |  |  | 8305 | 0.000 | -0.3696 | No |
| 41 | HTR1A |  |  | 8333 | 0.000 | -0.3713 | No |
| 42 | HTR2C |  |  | 8334 | 0.000 | -0.3713 | No |
| 43 | MTNR1B |  |  | 8469 | 0.000 | -0.3800 | No |
| 44 | NMS |  |  | 8485 | 0.000 | -0.3809 | No |
| 45 | NPSR1 |  |  | 8488 | 0.000 | -0.3811 | No |
| 46 | OPN1LW |  |  | 8753 | 0.000 | -0.3981 | No |
| 47 | OPN4 |  |  | 8754 | 0.000 | -0.3981 | No |
| 48 | RLN2 |  |  | 8804 | 0.000 | -0.4013 | No |
| 49 | TAAR1 |  |  | 8872 | 0.000 | -0.4056 | No |
| 50 | TAAR2 |  |  | 8873 | 0.000 | -0.4056 | No |
| 51 | TAAR5 |  |  | 8875 | 0.000 | -0.4056 | No |
| 52 | TAAR6 |  |  | 8876 | 0.000 | -0.4056 | No |
| 53 | TAAR8 |  |  | 8877 | 0.000 | -0.4056 | No |
| 54 | UTS2D |  |  | 8934 | 0.000 | -0.4093 | No |
| 55 | KNG1 |  |  | 8986 | 0.000 | -0.4126 | No |
| 56 | HTR5A |  |  | 8989 | 0.000 | -0.4127 | No |
| 57 | HRH1 |  |  | 8993 | 0.000 | -0.4129 | No |
| 58 | CXCL6 |  |  | 8999 | 0.000 | -0.4132 | No |
| 59 | LHCGR |  |  | 9014 | 0.000 | -0.4141 | No |
| 60 | OPN5 |  |  | 9015 | 0.000 | -0.4141 | No |
| 61 | RXFP1 |  |  | 9086 | 0.000 | -0.4186 | No |
| 62 | PROKR2 |  |  | 9145 | 0.000 | -0.4224 | No |
| 63 | NPFFR2 |  |  | 9150 | 0.000 | -0.4226 | No |
| 64 | TRH |  |  | 9182 | 0.000 | -0.4246 | No |
| 65 | MTNR1A |  |  | 9273 | 0.000 | -0.4304 | No |
| 66 | TAC3 |  |  | 9300 | 0.000 | -0.4321 | No |
| 67 | RGR |  |  | 9347 | 0.000 | -0.4351 | No |
| 68 | HTR2A |  |  | 9366 | 0.000 | -0.4362 | No |
| 69 | UTS2R |  |  | 9367 | 0.000 | -0.4362 | No |
| 70 | NPY5R |  |  | 9385 | 0.000 | -0.4373 | No |
| 71 | HCRTR2 |  |  | 9388 | 0.000 | -0.4375 | No |
| 72 | DRD1 |  |  | 9397 | 0.000 | -0.4380 | No |
| 73 | TSHB |  |  | 9452 | 0.000 | -0.4415 | No |
| 74 | NMUR2 |  |  | 9495 | 0.000 | -0.4442 | No |
| 75 | MC4R |  |  | 9554 | 0.000 | -0.4479 | No |
| 76 | RHO |  |  | 9573 | 0.000 | -0.4491 | No |
| 77 | OPRM1 |  |  | 9605 | 0.000 | -0.4511 | No |
| 78 | CCR3 |  |  | 9606 | 0.000 | -0.4511 | No |
| 79 | MC5R |  |  | 9630 | 0.000 | -0.4526 | No |
| 80 | HTR7 |  |  | 9637 | 0.000 | -0.4530 | No |
| 81 | AGTR2 |  |  | 9655 | 0.000 | -0.4541 | No |
| 82 | HTR1F |  |  | 9658 | 0.000 | -0.4542 | No |
| 83 | NMBR |  |  | 9686 | 0.000 | -0.4559 | No |
| 84 | RXFP3 |  |  | 9707 | 0.000 | -0.4572 | No |
| 85 | C5 |  |  | 9718 | 0.000 | -0.4579 | No |
| 86 | HRH3 |  |  | 9732 | 0.000 | -0.4587 | No |
| 87 | MC2R |  |  | 9747 | 0.000 | -0.4596 | No |
| 88 | RRH |  |  | 9788 | 0.000 | -0.4622 | No |
| 89 | HTR1D |  |  | 9834 | 0.000 | -0.4651 | No |
| 90 | CXCL3 |  |  | 9838 | 0.000 | -0.4653 | No |
| 91 | AVP |  |  | 9946 | 0.000 | -0.4722 | No |
| 92 | CXCL11 |  |  | 9974 | 0.000 | -0.4739 | No |
| 93 | ADORA3 |  |  | 10011 | 0.000 | -0.4763 | No |
| 94 | DRD2 |  |  | 10026 | 0.000 | -0.4772 | No |
| 95 | NMB |  |  | 10045 | 0.000 | -0.4783 | No |
| 96 | CCKBR |  |  | 10084 | 0.000 | -0.4808 | No |
| 97 | RLN3 |  |  | 10099 | 0.000 | -0.4817 | No |
| 98 | FPR1 |  |  | 10156 | -0.100 | -0.4850 | No |
| 99 | NPBWR1 |  |  | 10190 | -0.100 | -0.4868 | No |
| 100 | XCR1 |  |  | 10268 | -0.100 | -0.4914 | No |
| 101 | P2RY10 |  |  | 10322 | -0.100 | -0.4945 | No |
| 102 | ADRA1A |  |  | 10328 | -0.100 | -0.4945 | No |
| 103 | PTGFR |  |  | 10339 | -0.100 | -0.4948 | No |
| 104 | OPRK1 |  |  | 10357 | -0.100 | -0.4956 | No |
| 105 | CCL4 |  |  | 10361 | -0.100 | -0.4954 | No |
| 106 | AVPR2 |  |  | 10377 | -0.100 | -0.4961 | No |
| 107 | PNOC |  |  | 10385 | -0.100 | -0.4962 | No |
| 108 | NPW |  |  | 10389 | -0.100 | -0.4961 | No |
| 109 | CCR8 |  |  | 10423 | -0.100 | -0.4979 | No |
| 110 | GPR77 |  |  | 10438 | -0.100 | -0.4984 | No |
| 111 | RXFP4 |  |  | 10457 | -0.100 | -0.4993 | No |
| 112 | TACR3 |  |  | 10461 | -0.100 | -0.4991 | No |
| 113 | HTR6 |  |  | 10490 | -0.100 | -0.5006 | No |
| 114 | CYSLTR1 |  |  | 10497 | -0.100 | -0.5007 | No |
| 115 | CYSLTR2 |  |  | 10507 | -0.100 | -0.5009 | No |
| 116 | HRH2 |  |  | 10527 | -0.100 | -0.5018 | No |
| 117 | CCR9 |  |  | 10533 | -0.100 | -0.5018 | No |
| 118 | CXCL9 |  |  | 10552 | -0.100 | -0.5026 | No |
| 119 | PRLHR |  |  | 10601 | -0.100 | -0.5054 | No |
| 120 | PDYN |  |  | 10628 | -0.100 | -0.5067 | No |
| 121 | CCR4 |  |  | 10656 | -0.100 | -0.5081 | No |
| 122 | LOC728830 |  |  | 10672 | -0.100 | -0.5088 | No |
| 123 | HCRT |  |  | 10681 | -0.100 | -0.5090 | No |
| 124 | DRD3 |  |  | 10688 | -0.100 | -0.5090 | No |
| 125 | TSHR |  |  | 10809 | -0.200 | -0.5161 | No |
| 126 | GPR55 |  |  | 10815 | -0.200 | -0.5158 | No |
| 127 | XCL1 |  |  | 10837 | -0.200 | -0.5165 | No |
| 128 | GALR3 |  |  | 10878 | -0.200 | -0.5184 | No |
| 129 | OXTR |  |  | 10880 | -0.200 | -0.5178 | No |
| 130 | POMC |  |  | 10894 | -0.200 | -0.5180 | No |
| 131 | CCR2 |  |  | 10919 | -0.200 | -0.5189 | No |
| 132 | OPRL1 |  |  | 10930 | -0.200 | -0.5188 | No |
| 133 | GALR1 |  |  | 10975 | -0.200 | -0.5210 | No |
| 134 | CCL7 |  |  | 11021 | -0.200 | -0.5233 | No |
| 135 | AVPR1B |  |  | 11056 | -0.200 | -0.5248 | No |
| 136 | ADRA1B |  |  | 11074 | -0.200 | -0.5252 | No |
| 137 | GPR65 |  |  | 11081 | -0.200 | -0.5250 | No |
| 138 | HTR2B |  |  | 11086 | -0.200 | -0.5246 | No |
| 139 | CXCR6 |  |  | 11100 | -0.200 | -0.5247 | No |
| 140 | HTR1B |  |  | 11107 | -0.200 | -0.5245 | No |
| 141 | CCL2 |  |  | 11114 | -0.200 | -0.5242 | No |
| 142 | F2RL2 |  |  | 11120 | -0.200 | -0.5238 | No |
| 143 | GRPR |  |  | 11139 | -0.200 | -0.5243 | No |
| 144 | NPY2R |  |  | 11142 | -0.200 | -0.5238 | No |
| 145 | P2RY12 |  |  | 11167 | -0.200 | -0.5247 | No |
| 146 | SSTR5 |  |  | 11181 | -0.300 | -0.5246 | No |
| 147 | SSTR3 |  |  | 11204 | -0.300 | -0.5250 | No |
| 148 | F2 |  |  | 11305 | -0.300 | -0.5304 | No |
| 149 | TACR1 |  |  | 11308 | -0.300 | -0.5296 | No |
| 150 | NPFFR1 |  |  | 11432 | -0.300 | -0.5365 | No |
| 151 | CCL19 |  |  | 11443 | -0.300 | -0.5362 | No |
| 152 | CNR1 |  |  | 11468 | -0.400 | -0.5364 | No |
| 153 | ADORA2B |  |  | 11483 | -0.400 | -0.5360 | No |
| 154 | P2RY13 |  |  | 11489 | -0.400 | -0.5350 | No |
| 155 | NTS |  |  | 11525 | -0.400 | -0.5359 | No |
| 156 | BDKRB1 |  |  | 11619 | -0.400 | -0.5406 | No |
| 157 | OPRD1 |  |  | 11624 | -0.400 | -0.5395 | No |
| 158 | NTSR1 |  |  | 11651 | -0.400 | -0.5399 | No |
| 159 | FFAR1 |  |  | 11661 | -0.500 | -0.5388 | No |
| 160 | CCR6 |  |  | 11662 | -0.500 | -0.5372 | No |
| 161 | CNR2 |  |  | 11682 | -0.500 | -0.5367 | No |
| 162 | SSTR4 |  |  | 11739 | -0.500 | -0.5387 | No |
| 163 | LTB4R |  |  | 11764 | -0.500 | -0.5386 | No |
| 164 | EDN1 |  |  | 11777 | -0.500 | -0.5377 | No |
| 165 | PRLH |  |  | 11811 | -0.500 | -0.5382 | No |
| 166 | ADRB2 |  |  | 11843 | -0.600 | -0.5382 | No |
| 167 | LTB4R2 |  |  | 11943 | -0.600 | -0.5426 | No |
| 168 | C3AR1 |  |  | 11965 | -0.600 | -0.5420 | No |
| 169 | NPY1R |  |  | 12054 | -0.700 | -0.5454 | Yes |
| 170 | CCBP2 |  |  | 12069 | -0.700 | -0.5440 | Yes |
| 171 | MCHR1 |  |  | 12108 | -0.700 | -0.5441 | Yes |
| 172 | CCL20 |  |  | 12138 | -0.700 | -0.5437 | Yes |
| 173 | EDNRB |  |  | 12155 | -0.700 | -0.5424 | Yes |
| 174 | KISS1 |  |  | 12176 | -0.800 | -0.5410 | Yes |
| 175 | EDN3 |  |  | 12211 | -0.800 | -0.5406 | Yes |
| 176 | FFAR3 |  |  | 12216 | -0.800 | -0.5382 | Yes |
| 177 | PTGER2 |  |  | 12243 | -0.800 | -0.5372 | Yes |
| 178 | ADORA1 |  |  | 12252 | -0.800 | -0.5351 | Yes |
| 179 | CHRM3 |  |  | 12254 | -0.800 | -0.5325 | Yes |
| 180 | CCL21 |  |  | 12271 | -0.800 | -0.5309 | Yes |
| 181 | CCL22 |  |  | 12279 | -0.800 | -0.5287 | Yes |
| 182 | CXCL13 |  |  | 12321 | -0.900 | -0.5284 | Yes |
| 183 | PTGIR |  |  | 12396 | -0.900 | -0.5302 | Yes |
| 184 | AVPR1A |  |  | 12399 | -0.900 | -0.5273 | Yes |
| 185 | NMUR1 |  |  | 12483 | -1.000 | -0.5294 | Yes |
| 186 | GPR18 |  |  | 12538 | -1.000 | -0.5296 | Yes |
| 187 | P2RY2 |  |  | 12549 | -1.000 | -0.5269 | Yes |
| 188 | GPR68 |  |  | 12582 | -1.000 | -0.5257 | Yes |
| 189 | CXCR3 |  |  | 12696 | -1.100 | -0.5293 | Yes |
| 190 | ADORA2A |  |  | 12713 | -1.200 | -0.5264 | Yes |
| 191 | CCR7 |  |  | 12772 | -1.200 | -0.5262 | Yes |
| 192 | CCKAR |  |  | 12831 | -1.300 | -0.5256 | Yes |
| 193 | GPR17 |  |  | 12880 | -1.300 | -0.5244 | Yes |
| 194 | HCRTR1 |  |  | 12888 | -1.300 | -0.5206 | Yes |
| 195 | CHRM2 |  |  | 12903 | -1.300 | -0.5172 | Yes |
| 196 | GPR39 |  |  | 12912 | -1.400 | -0.5131 | Yes |
| 197 | GRP |  |  | 12978 | -1.400 | -0.5126 | Yes |
| 198 | GPR132 |  |  | 12986 | -1.400 | -0.5084 | Yes |
| 199 | HTR4 |  |  | 12993 | -1.400 | -0.5042 | Yes |
| 200 | GPR4 |  |  | 13015 | -1.500 | -0.5006 | Yes |
| 201 | P2RY4 |  |  | 13016 | -1.500 | -0.4956 | Yes |
| 202 | OPN3 |  |  | 13031 | -1.500 | -0.4916 | Yes |
| 203 | APP |  |  | 13046 | -1.500 | -0.4875 | Yes |
| 204 | NMU |  |  | 13073 | -1.500 | -0.4843 | Yes |
| 205 | CCL28 |  |  | 13098 | -1.600 | -0.4805 | Yes |
| 206 | AGTR1 |  |  | 13128 | -1.600 | -0.4771 | Yes |
| 207 | EDNRA |  |  | 13135 | -1.600 | -0.4722 | Yes |
| 208 | PENK |  |  | 13156 | -1.600 | -0.4682 | Yes |
| 209 | KISS1R |  |  | 13212 | -1.700 | -0.4661 | Yes |
| 210 | GALR2 |  |  | 13227 | -1.700 | -0.4614 | Yes |
| 211 | CCL27 |  |  | 13347 | -1.800 | -0.4631 | Yes |
| 212 | GPBAR1 |  |  | 13500 | -2.000 | -0.4663 | Yes |
| 213 | NPY |  |  | 13555 | -2.100 | -0.4629 | Yes |
| 214 | CX3CR1 |  |  | 13586 | -2.100 | -0.4579 | Yes |
| 215 | SSTR2 |  |  | 13633 | -2.200 | -0.4536 | Yes |
| 216 | CHRM1 |  |  | 13644 | -2.200 | -0.4469 | Yes |
| 217 | CCL11 |  |  | 13654 | -2.200 | -0.4403 | Yes |
| 218 | CCK |  |  | 13672 | -2.300 | -0.4337 | Yes |
| 219 | PTAFR |  |  | 13676 | -2.300 | -0.4263 | Yes |
| 220 | CCL5 |  |  | 13706 | -2.300 | -0.4206 | Yes |
| 221 | CCR10 |  |  | 13708 | -2.300 | -0.4131 | Yes |
| 222 | F2RL3 |  |  | 13786 | -2.400 | -0.4101 | Yes |
| 223 | ADRB1 |  |  | 13803 | -2.500 | -0.4029 | Yes |
| 224 | BDKRB2 |  |  | 13805 | -2.500 | -0.3947 | Yes |
| 225 | PTGER3 |  |  | 13814 | -2.500 | -0.3869 | Yes |
| 226 | CCRL1 |  |  | 13834 | -2.500 | -0.3799 | Yes |
| 227 | PPYR1 |  |  | 13845 | -2.600 | -0.3719 | Yes |
| 228 | C5AR1 |  |  | 13884 | -2.600 | -0.3658 | Yes |
| 229 | TAC1 |  |  | 13930 | -2.700 | -0.3598 | Yes |
| 230 | INSL3 |  |  | 13939 | -2.700 | -0.3514 | Yes |
| 231 | DARC |  |  | 14153 | -3.000 | -0.3552 | Yes |
| 232 | PPY |  |  | 14193 | -3.100 | -0.3475 | Yes |
| 233 | P2RY1 |  |  | 14216 | -3.100 | -0.3386 | Yes |
| 234 | CXCL12 |  |  | 14228 | -3.200 | -0.3288 | Yes |
| 235 | CXCR7 |  |  | 14254 | -3.200 | -0.3198 | Yes |
| 236 | CXCR4 |  |  | 14374 | -3.500 | -0.3159 | Yes |
| 237 | PF4 |  |  | 14433 | -3.700 | -0.3074 | Yes |
| 238 | PTGER4 |  |  | 14532 | -3.900 | -0.3009 | Yes |
| 239 | ADRB3 |  |  | 14670 | -4.300 | -0.2955 | Yes |
| 240 | P2RY6 |  |  | 14706 | -4.300 | -0.2835 | Yes |
| 241 | ADRA2A |  |  | 14754 | -4.500 | -0.2717 | Yes |
| 242 | SAA1 |  |  | 14809 | -4.600 | -0.2600 | Yes |
| 243 | TBXA2R |  |  | 14827 | -4.700 | -0.2455 | Yes |
| 244 | FFAR2 |  |  | 14833 | -4.700 | -0.2303 | Yes |
| 245 | AGT |  |  | 14844 | -4.700 | -0.2154 | Yes |
| 246 | TACR2 |  |  | 14856 | -4.700 | -0.2006 | Yes |
| 247 | CXCL16 |  |  | 14924 | -4.900 | -0.1887 | Yes |
| 248 | INSL5 |  |  | 15099 | -5.700 | -0.1811 | Yes |
| 249 | GAL |  |  | 15193 | -6.100 | -0.1669 | Yes |
| 250 | CX3CL1 |  |  | 15197 | -6.200 | -0.1466 | Yes |
| 251 | SSTR1 |  |  | 15216 | -6.300 | -0.1270 | Yes |
| 252 | EDN2 |  |  | 15252 | -6.400 | -0.1081 | Yes |
| 253 | PYY |  |  | 15309 | -6.800 | -0.0892 | Yes |
| 254 | PTGER1 |  |  | 15335 | -7.000 | -0.0677 | Yes |
| 255 | F2RL1 |  |  | 15456 | -8.000 | -0.0490 | Yes |
| 256 | SST |  |  | 15599 | -10.200 | -0.0244 | Yes |
| 257 | C3 |  |  | 15605 | -10.300 | 0.0093 | Yes |
Table: GSEA details [plain text format]

  

Fig 2: REACTOME\_CLASS\_A1\_RHODOPSIN\_LIKE\_RECEPTORS: Random ES distribution      
 Gene set null distribution of ES for **REACTOME\_CLASS\_A1\_RHODOPSIN\_LIKE\_RECEPTORS**

  
