# Supplementary material for: Immunomic, genomic and transcriptomic characterization of CT26 colorectal carcinoma
Source: BMC Genomics. 2014 Mar 13;15(1):190. doi: 10.1186/1471-2164-15-190 (PMC4007559; doi:10.1186/1471-2164-15-190)
Supplement: Supplementary file 8 — Additional file 8: Contains the Gene Pattern gene set membership and enrichment values in an html format. The file index.html is the entry point. (ZIP 13 MB) [file 12864_2013_7028_MOESM8_ESM.zip › REACTOME_DNA_REPLICATION.html]

Details for gene set REACTOME\_DNA\_REPLICATION[GSEA]

|  || Dataset | CT26\_gene\_expression |
| Phenotype | NoPhenotypeAvailable |
| Upregulated in class | na\_pos |
| GeneSet | REACTOME\_DNA\_REPLICATION |
| Enrichment Score (ES) | 0.75823283 |
| Normalized Enrichment Score (NES) | 1.6873641 |
| Nominal p-value | 0.0 |
| FDR q-value | 0.0017992357 |
| FWER p-Value | 0.032 |
Table: GSEA Results Summary

  

Fig 1: Enrichment plot: REACTOME\_DNA\_REPLICATION      
 Profile of the Running ES Score & Positions of GeneSet Members on the Rank Ordered List

  

| PROBE | GENE SYMBOL | GENE\_TITLE | RANK IN GENE LIST | RANK METRIC SCORE | RUNNING ES | CORE ENRICHMENT || 1 | PSMD12 |  |  | 27 | 39.500 | 0.0164 | Yes |
| 2 | PSMD1 |  |  | 41 | 37.400 | 0.0328 | Yes |
| 3 | KIF20A |  |  | 77 | 31.900 | 0.0452 | Yes |
| 4 | MCM4 |  |  | 81 | 31.500 | 0.0595 | Yes |
| 5 | RAD21 |  |  | 86 | 31.400 | 0.0737 | Yes |
| 6 | RCC2 |  |  | 99 | 30.500 | 0.0870 | Yes |
| 7 | LIG1 |  |  | 104 | 30.000 | 0.1005 | Yes |
| 8 | PRIM1 |  |  | 116 | 29.000 | 0.1132 | Yes |
| 9 | RPA1 |  |  | 120 | 28.700 | 0.1262 | Yes |
| 10 | XPO1 |  |  | 148 | 27.000 | 0.1369 | Yes |
| 11 | MCM6 |  |  | 163 | 26.600 | 0.1482 | Yes |
| 12 | PSMC2 |  |  | 173 | 26.000 | 0.1596 | Yes |
| 13 | CCNA2 |  |  | 178 | 25.600 | 0.1711 | Yes |
| 14 | AHCTF1 |  |  | 189 | 24.800 | 0.1819 | Yes |
| 15 | CKAP5 |  |  | 210 | 24.300 | 0.1918 | Yes |
| 16 | RANGAP1 |  |  | 220 | 24.000 | 0.2023 | Yes |
| 17 | PSMD2 |  |  | 227 | 23.700 | 0.2128 | Yes |
| 18 | PSMA3 |  |  | 245 | 23.200 | 0.2224 | Yes |
| 19 | PSMC4 |  |  | 253 | 22.900 | 0.2324 | Yes |
| 20 | INCENP |  |  | 260 | 22.700 | 0.2425 | Yes |
| 21 | SMC3 |  |  | 265 | 22.600 | 0.2526 | Yes |
| 22 | CDC20 |  |  | 284 | 22.200 | 0.2617 | Yes |
| 23 | MCM7 |  |  | 290 | 21.900 | 0.2715 | Yes |
| 24 | MCM3 |  |  | 315 | 21.400 | 0.2798 | Yes |
| 25 | KIF23 |  |  | 337 | 21.000 | 0.2881 | Yes |
| 26 | BUB1 |  |  | 360 | 20.600 | 0.2961 | Yes |
| 27 | DBF4 |  |  | 369 | 20.400 | 0.3050 | Yes |
| 28 | PSMA5 |  |  | 381 | 20.200 | 0.3136 | Yes |
| 29 | PCNA |  |  | 386 | 20.200 | 0.3226 | Yes |
| 30 | NUP107 |  |  | 399 | 19.900 | 0.3310 | Yes |
| 31 | CENPN |  |  | 403 | 19.900 | 0.3400 | Yes |
| 32 | STAG2 |  |  | 456 | 19.100 | 0.3454 | Yes |
| 33 | PSMA4 |  |  | 484 | 18.600 | 0.3523 | Yes |
| 34 | PSMC1 |  |  | 493 | 18.600 | 0.3603 | Yes |
| 35 | BIRC5 |  |  | 507 | 18.400 | 0.3679 | Yes |
| 36 | BUB3 |  |  | 533 | 18.100 | 0.3747 | Yes |
| 37 | MAD2L1 |  |  | 559 | 17.900 | 0.3813 | Yes |
| 38 | ZWINT |  |  | 574 | 17.700 | 0.3885 | Yes |
| 39 | PSMC6 |  |  | 579 | 17.600 | 0.3964 | Yes |
| 40 | RANBP2 |  |  | 605 | 17.300 | 0.4027 | Yes |
| 41 | PPP2R5D |  |  | 607 | 17.300 | 0.4106 | Yes |
| 42 | BUB1B |  |  | 609 | 17.300 | 0.4185 | Yes |
| 43 | PAFAH1B1 |  |  | 614 | 17.300 | 0.4262 | Yes |
| 44 | ZWILCH |  |  | 620 | 17.200 | 0.4338 | Yes |
| 45 | CENPQ |  |  | 635 | 17.000 | 0.4407 | Yes |
| 46 | RPS27A |  |  | 668 | 16.700 | 0.4464 | Yes |
| 47 | FBXO5 |  |  | 688 | 16.500 | 0.4527 | Yes |
| 48 | RFC2 |  |  | 712 | 16.300 | 0.4588 | Yes |
| 49 | KIF2A |  |  | 719 | 16.200 | 0.4658 | Yes |
| 50 | PSMA1 |  |  | 724 | 16.200 | 0.4730 | Yes |
| 51 | SGOL1 |  |  | 857 | 15.100 | 0.4715 | Yes |
| 52 | PSMD14 |  |  | 859 | 15.100 | 0.4784 | Yes |
| 53 | NUF2 |  |  | 866 | 15.100 | 0.4849 | Yes |
| 54 | NDC80 |  |  | 878 | 15.000 | 0.4911 | Yes |
| 55 | PSMB3 |  |  | 881 | 15.000 | 0.4979 | Yes |
| 56 | PSME1 |  |  | 893 | 14.900 | 0.5041 | Yes |
| 57 | GMNN |  |  | 911 | 14.800 | 0.5098 | Yes |
| 58 | SGOL2 |  |  | 1015 | 14.100 | 0.5096 | Yes |
| 59 | CDCA8 |  |  | 1017 | 14.100 | 0.5161 | Yes |
| 60 | KIF2C |  |  | 1036 | 14.000 | 0.5214 | Yes |
| 61 | FEN1 |  |  | 1052 | 13.900 | 0.5268 | Yes |
| 62 | SMC1A |  |  | 1064 | 13.800 | 0.5324 | Yes |
| 63 | NUP85 |  |  | 1073 | 13.800 | 0.5383 | Yes |
| 64 | MIS12 |  |  | 1095 | 13.600 | 0.5432 | Yes |
| 65 | PSMC5 |  |  | 1103 | 13.600 | 0.5490 | Yes |
| 66 | E2F1 |  |  | 1172 | 13.100 | 0.5506 | Yes |
| 67 | PSMD11 |  |  | 1192 | 13.100 | 0.5555 | Yes |
| 68 | RPA2 |  |  | 1205 | 13.000 | 0.5607 | Yes |
| 69 | POLA1 |  |  | 1250 | 12.800 | 0.5637 | Yes |
| 70 | CENPH |  |  | 1257 | 12.800 | 0.5692 | Yes |
| 71 | NUP37 |  |  | 1258 | 12.800 | 0.5751 | Yes |
| 72 | CENPI |  |  | 1262 | 12.700 | 0.5808 | Yes |
| 73 | MCM10 |  |  | 1264 | 12.700 | 0.5865 | Yes |
| 74 | MCM2 |  |  | 1281 | 12.700 | 0.5914 | Yes |
| 75 | PSMB7 |  |  | 1288 | 12.700 | 0.5968 | Yes |
| 76 | SPC25 |  |  | 1296 | 12.600 | 0.6022 | Yes |
| 77 | PSMA7 |  |  | 1308 | 12.500 | 0.6072 | Yes |
| 78 | PPP2R5C |  |  | 1333 | 12.400 | 0.6114 | Yes |
| 79 | PSMD7 |  |  | 1346 | 12.300 | 0.6163 | Yes |
| 80 | PSME4 |  |  | 1361 | 12.300 | 0.6210 | Yes |
| 81 | PSMD6 |  |  | 1380 | 12.200 | 0.6255 | Yes |
| 82 | MAPRE1 |  |  | 1391 | 12.100 | 0.6304 | Yes |
| 83 | PSMD10 |  |  | 1417 | 12.000 | 0.6343 | Yes |
| 84 | KIF18A |  |  | 1462 | 11.800 | 0.6369 | Yes |
| 85 | MCM5 |  |  | 1526 | 11.500 | 0.6382 | Yes |
| 86 | PSMB1 |  |  | 1532 | 11.500 | 0.6431 | Yes |
| 87 | CDT1 |  |  | 1580 | 11.300 | 0.6453 | Yes |
| 88 | RPA3 |  |  | 1621 | 11.200 | 0.6479 | Yes |
| 89 | SPC24 |  |  | 1627 | 11.200 | 0.6527 | Yes |
| 90 | NUP133 |  |  | 1634 | 11.100 | 0.6575 | Yes |
| 91 | SEH1L |  |  | 1666 | 10.900 | 0.6605 | Yes |
| 92 | RFC5 |  |  | 1676 | 10.900 | 0.6649 | Yes |
| 93 | CENPA |  |  | 1762 | 10.600 | 0.6644 | Yes |
| 94 | PSMD5 |  |  | 1765 | 10.600 | 0.6691 | Yes |
| 95 | PSME2 |  |  | 1904 | 10.000 | 0.6648 | Yes |
| 96 | CASC5 |  |  | 1912 | 10.000 | 0.6690 | Yes |
| 97 | CENPC1 |  |  | 1914 | 10.000 | 0.6735 | Yes |
| 98 | PSMB2 |  |  | 1917 | 10.000 | 0.6780 | Yes |
| 99 | DSN1 |  |  | 1919 | 10.000 | 0.6825 | Yes |
| 100 | PSMA2 |  |  | 1980 | 9.700 | 0.6831 | Yes |
| 101 | PPP2R1B |  |  | 2048 | 9.500 | 0.6832 | Yes |
| 102 | STAG1 |  |  | 2077 | 9.400 | 0.6857 | Yes |
| 103 | RFC3 |  |  | 2115 | 9.300 | 0.6876 | Yes |
| 104 | POLA2 |  |  | 2142 | 9.200 | 0.6902 | Yes |
| 105 | CDKN1A |  |  | 2165 | 9.100 | 0.6930 | Yes |
| 106 | PPP2CB |  |  | 2170 | 9.100 | 0.6969 | Yes |
| 107 | CLIP1 |  |  | 2185 | 9.100 | 0.7002 | Yes |
| 108 | PSMA6 |  |  | 2205 | 9.000 | 0.7031 | Yes |
| 109 | CENPT |  |  | 2232 | 9.000 | 0.7056 | Yes |
| 110 | PSMC3 |  |  | 2237 | 9.000 | 0.7095 | Yes |
| 111 | POLE |  |  | 2239 | 8.900 | 0.7135 | Yes |
| 112 | RFC4 |  |  | 2243 | 8.900 | 0.7174 | Yes |
| 113 | KNTC1 |  |  | 2256 | 8.900 | 0.7207 | Yes |
| 114 | CENPL |  |  | 2261 | 8.900 | 0.7246 | Yes |
| 115 | CDC7 |  |  | 2273 | 8.900 | 0.7280 | Yes |
| 116 | POLE2 |  |  | 2300 | 8.800 | 0.7303 | Yes |
| 117 | POLD1 |  |  | 2302 | 8.800 | 0.7343 | Yes |
| 118 | APITD1 |  |  | 2304 | 8.800 | 0.7383 | Yes |
| 119 | PMF1 |  |  | 2386 | 8.500 | 0.7370 | Yes |
| 120 | GINS4 |  |  | 2439 | 8.400 | 0.7375 | Yes |
| 121 | CENPM |  |  | 2477 | 8.300 | 0.7390 | Yes |
| 122 | RPS27 |  |  | 2503 | 8.200 | 0.7412 | Yes |
| 123 | PSMD8 |  |  | 2504 | 8.200 | 0.7449 | Yes |
| 124 | PSMD9 |  |  | 2508 | 8.200 | 0.7485 | Yes |
| 125 | TAOK1 |  |  | 2518 | 8.100 | 0.7517 | Yes |
| 126 | CDK2 |  |  | 2519 | 8.100 | 0.7554 | Yes |
| 127 | PSMD4 |  |  | 2609 | 7.900 | 0.7533 | Yes |
| 128 | CCDC99 |  |  | 2632 | 7.800 | 0.7555 | Yes |
| 129 | AURKB |  |  | 2822 | 7.300 | 0.7467 | Yes |
| 130 | NSL1 |  |  | 2916 | 7.100 | 0.7440 | Yes |
| 131 | RB1 |  |  | 2925 | 7.100 | 0.7468 | Yes |
| 132 | MLF1IP |  |  | 2974 | 7.000 | 0.7469 | Yes |
| 133 | PSMD13 |  |  | 2986 | 6.900 | 0.7494 | Yes |
| 134 | GINS1 |  |  | 2999 | 6.900 | 0.7518 | Yes |
| 135 | ZW10 |  |  | 3019 | 6.900 | 0.7537 | Yes |
| 136 | CENPP |  |  | 3065 | 6.800 | 0.7540 | Yes |
| 137 | GINS2 |  |  | 3094 | 6.700 | 0.7553 | Yes |
| 138 | NUDC |  |  | 3111 | 6.600 | 0.7573 | Yes |
| 139 | E2F3 |  |  | 3189 | 6.500 | 0.7553 | Yes |
| 140 | PSMB4 |  |  | 3191 | 6.500 | 0.7582 | Yes |
| 141 | NUP43 |  |  | 3343 | 6.100 | 0.7513 | No |
| 142 | NDEL1 |  |  | 3503 | 5.800 | 0.7438 | No |
| 143 | MCM8 |  |  | 3515 | 5.700 | 0.7457 | No |
| 144 | POLD3 |  |  | 3544 | 5.700 | 0.7465 | No |
| 145 | MAD1L1 |  |  | 3575 | 5.600 | 0.7472 | No |
| 146 | CLASP1 |  |  | 3634 | 5.500 | 0.7460 | No |
| 147 | CDC6 |  |  | 3705 | 5.300 | 0.7439 | No |
| 148 | PPP2R5E |  |  | 3781 | 5.200 | 0.7415 | No |
| 149 | CENPK |  |  | 4002 | 4.800 | 0.7296 | No |
| 150 | PPP1CC |  |  | 4111 | 4.600 | 0.7248 | No |
| 151 | PPP2R1A |  |  | 4124 | 4.600 | 0.7261 | No |
| 152 | PSMB5 |  |  | 4221 | 4.400 | 0.7220 | No |
| 153 | SEC13 |  |  | 4498 | 3.900 | 0.7061 | No |
| 154 | PSMF1 |  |  | 5043 | 3.000 | 0.6725 | No |
| 155 | POLD2 |  |  | 5234 | 2.700 | 0.6616 | No |
| 156 | CDKN1B |  |  | 5505 | 2.300 | 0.6453 | No |
| 157 | UBA52 |  |  | 5629 | 2.100 | 0.6384 | No |
| 158 | PSMD3 |  |  | 5741 | 1.900 | 0.6321 | No |
| 159 | CENPO |  |  | 5968 | 1.600 | 0.6183 | No |
| 160 | ITGB3BP |  |  | 6480 | 0.900 | 0.5859 | No |
| 161 | PLK1 |  |  | 6602 | 0.800 | 0.5785 | No |
| 162 | PPP2CA |  |  | 6791 | 0.600 | 0.5668 | No |
| 163 | PPP2R5A |  |  | 6841 | 0.600 | 0.5639 | No |
| 164 | E2F2 |  |  | 7036 | 0.400 | 0.5516 | No |
| 165 | KIF2B |  |  | 8366 | 0.000 | 0.4663 | No |
| 166 | PSMA8 |  |  | 9516 | 0.000 | 0.3925 | No |
| 167 | PSMB6 |  |  | 9945 | 0.000 | 0.3650 | No |
| 168 | CCNA1 |  |  | 10419 | -0.100 | 0.3347 | No |
| 169 | PSMB8 |  |  | 11241 | -0.300 | 0.2822 | No |
| 170 | POLD4 |  |  | 12916 | -1.400 | 0.1753 | No |
| 171 | PSMB10 |  |  | 13961 | -2.700 | 0.1095 | No |
| 172 | PPP2R5B |  |  | 14239 | -3.200 | 0.0932 | No |
| 173 | PSMB9 |  |  | 14266 | -3.300 | 0.0931 | No |
| 174 | GORASP1 |  |  | 14777 | -4.500 | 0.0624 | No |
Table: GSEA details [plain text format]

  

Fig 2: REACTOME\_DNA\_REPLICATION: Random ES distribution      
 Gene set null distribution of ES for **REACTOME\_DNA\_REPLICATION**

  
