# Supplementary material for: Immunomic, genomic and transcriptomic characterization of CT26 colorectal carcinoma
Source: BMC Genomics. 2014 Mar 13;15(1):190. doi: 10.1186/1471-2164-15-190 (PMC4007559; doi:10.1186/1471-2164-15-190)
Supplement: Supplementary file 8 — Additional file 8: Contains the Gene Pattern gene set membership and enrichment values in an html format. The file index.html is the entry point. (ZIP 13 MB) [file 12864_2013_7028_MOESM8_ESM.zip › REACTOME_GPCR_LIGAND_BINDING.html]

Details for gene set REACTOME\_GPCR\_LIGAND\_BINDING[GSEA]

|  || Dataset | CT26\_gene\_expression |
| Phenotype | NoPhenotypeAvailable |
| Upregulated in class | na\_neg |
| GeneSet | REACTOME\_GPCR\_LIGAND\_BINDING |
| Enrichment Score (ES) | -0.46124336 |
| Normalized Enrichment Score (NES) | NaN |
| Nominal p-value | NaN |
| FDR q-value | 1.0 |
| FWER p-Value | 0.0 |
Table: GSEA Results Summary

  

Fig 1: Enrichment plot: REACTOME\_GPCR\_LIGAND\_BINDING      
 Profile of the Running ES Score & Positions of GeneSet Members on the Rank Ordered List

  

| PROBE | GENE SYMBOL | GENE\_TITLE | RANK IN GENE LIST | RANK METRIC SCORE | RUNNING ES | CORE ENRICHMENT || 1 | WNT10A |  |  | 539 | 18.100 | 0.0024 | No |
| 2 | ANXA1 |  |  | 697 | 16.400 | 0.0261 | No |
| 3 | HEBP1 |  |  | 1978 | 9.700 | -0.0370 | No |
| 4 | SMO |  |  | 2387 | 8.500 | -0.0459 | No |
| 5 | GNG10 |  |  | 2412 | 8.500 | -0.0299 | No |
| 6 | F2R |  |  | 2440 | 8.400 | -0.0143 | No |
| 7 | GNB1 |  |  | 3457 | 5.800 | -0.0683 | No |
| 8 | PMCH |  |  | 3480 | 5.800 | -0.0578 | No |
| 9 | GNG5 |  |  | 3909 | 4.900 | -0.0754 | No |
| 10 | FZD6 |  |  | 4142 | 4.500 | -0.0812 | No |
| 11 | GNG12 |  |  | 4271 | 4.300 | -0.0806 | No |
| 12 | GABBR1 |  |  | 4585 | 3.700 | -0.0933 | No |
| 13 | GNB2 |  |  | 5311 | 2.600 | -0.1350 | No |
| 14 | GNG13 |  |  | 5348 | 2.500 | -0.1322 | No |
| 15 | PTCH1 |  |  | 5568 | 2.200 | -0.1419 | No |
| 16 | GNAS |  |  | 5595 | 2.200 | -0.1390 | No |
| 17 | WNT10B |  |  | 5648 | 2.100 | -0.1381 | No |
| 18 | GNG8 |  |  | 5784 | 1.900 | -0.1429 | No |
| 19 | CCL25 |  |  | 5788 | 1.900 | -0.1392 | No |
| 20 | GNRH1 |  |  | 6126 | 1.500 | -0.1580 | No |
| 21 | PROKR1 |  |  | 6193 | 1.400 | -0.1593 | No |
| 22 | FZD3 |  |  | 6218 | 1.300 | -0.1582 | No |
| 23 | CXCL2 |  |  | 6388 | 1.100 | -0.1669 | No |
| 24 | CCR1 |  |  | 6492 | 0.900 | -0.1717 | No |
| 25 | ADM2 |  |  | 6640 | 0.800 | -0.1796 | No |
| 26 | PTHLH |  |  | 6699 | 0.700 | -0.1820 | No |
| 27 | TAS1R2 |  |  | 6777 | 0.600 | -0.1857 | No |
| 28 | GPR44 |  |  | 6826 | 0.600 | -0.1876 | No |
| 29 | CALCRL |  |  | 6853 | 0.500 | -0.1883 | No |
| 30 | ADRA2B |  |  | 7044 | 0.400 | -0.1998 | No |
| 31 | CXCL10 |  |  | 7089 | 0.300 | -0.2020 | No |
| 32 | NPFF |  |  | 7140 | 0.300 | -0.2046 | No |
| 33 | P2RY14 |  |  | 7245 | 0.200 | -0.2110 | No |
| 34 | NTSR2 |  |  | 7274 | 0.200 | -0.2124 | No |
| 35 | PPBP |  |  | 7319 | 0.100 | -0.2150 | No |
| 36 | LHB |  |  | 7411 | 0.100 | -0.2207 | No |
| 37 | OXT |  |  | 7433 | 0.100 | -0.2219 | No |
| 38 | ADRA1D |  |  | 7481 | 0.100 | -0.2247 | No |
| 39 | CCL17 |  |  | 7491 | 0.100 | -0.2251 | No |
| 40 | UTS2 |  |  | 7534 | 0.000 | -0.2278 | No |
| 41 | GHRL |  |  | 7558 | 0.000 | -0.2293 | No |
| 42 | MC3R |  |  | 7564 | 0.000 | -0.2297 | No |
| 43 | PROK1 |  |  | 7579 | 0.000 | -0.2306 | No |
| 44 | OPN1SW |  |  | 7624 | 0.000 | -0.2334 | No |
| 45 | TAS1R1 |  |  | 7729 | 0.000 | -0.2402 | No |
| 46 | TRHR |  |  | 7758 | 0.000 | -0.2420 | No |
| 47 | WNT1 |  |  | 7759 | 0.000 | -0.2420 | No |
| 48 | WNT7A |  |  | 7781 | 0.000 | -0.2434 | No |
| 49 | RXFP2 |  |  | 7821 | 0.000 | -0.2459 | No |
| 50 | NPS |  |  | 7825 | 0.000 | -0.2461 | No |
| 51 | HRH4 |  |  | 7848 | 0.000 | -0.2475 | No |
| 52 | PTGDR |  |  | 7881 | 0.000 | -0.2496 | No |
| 53 | GRM3 |  |  | 7901 | 0.000 | -0.2508 | No |
| 54 | GRM8 |  |  | 7911 | 0.000 | -0.2514 | No |
| 55 | MC1R |  |  | 8028 | 0.000 | -0.2590 | No |
| 56 | BRS3 |  |  | 8130 | 0.000 | -0.2655 | No |
| 57 | CGA |  |  | 8158 | 0.000 | -0.2673 | No |
| 58 | CHRM5 |  |  | 8159 | 0.000 | -0.2673 | No |
| 59 | CRH |  |  | 8178 | 0.000 | -0.2684 | No |
| 60 | DRD5 |  |  | 8229 | 0.000 | -0.2717 | No |
| 61 | FSHB |  |  | 8272 | 0.000 | -0.2744 | No |
| 62 | FSHR |  |  | 8273 | 0.000 | -0.2744 | No |
| 63 | GAST |  |  | 8284 | 0.000 | -0.2751 | No |
| 64 | GHSR |  |  | 8289 | 0.000 | -0.2753 | No |
| 65 | GNGT1 |  |  | 8304 | 0.000 | -0.2762 | No |
| 66 | GNRHR |  |  | 8305 | 0.000 | -0.2762 | No |
| 67 | HTR1A |  |  | 8333 | 0.000 | -0.2780 | No |
| 68 | HTR2C |  |  | 8334 | 0.000 | -0.2780 | No |
| 69 | MTNR1B |  |  | 8469 | 0.000 | -0.2867 | No |
| 70 | NMS |  |  | 8485 | 0.000 | -0.2877 | No |
| 71 | NPSR1 |  |  | 8488 | 0.000 | -0.2878 | No |
| 72 | OPN1LW |  |  | 8753 | 0.000 | -0.3049 | No |
| 73 | OPN4 |  |  | 8754 | 0.000 | -0.3049 | No |
| 74 | PTH |  |  | 8795 | 0.000 | -0.3075 | No |
| 75 | RLN2 |  |  | 8804 | 0.000 | -0.3081 | No |
| 76 | TAAR1 |  |  | 8872 | 0.000 | -0.3124 | No |
| 77 | TAAR2 |  |  | 8873 | 0.000 | -0.3124 | No |
| 78 | TAAR5 |  |  | 8875 | 0.000 | -0.3125 | No |
| 79 | TAAR6 |  |  | 8876 | 0.000 | -0.3125 | No |
| 80 | TAAR8 |  |  | 8877 | 0.000 | -0.3125 | No |
| 81 | UTS2D |  |  | 8934 | 0.000 | -0.3161 | No |
| 82 | WNT3A |  |  | 8942 | 0.000 | -0.3166 | No |
| 83 | KNG1 |  |  | 8986 | 0.000 | -0.3194 | No |
| 84 | HTR5A |  |  | 8989 | 0.000 | -0.3195 | No |
| 85 | HRH1 |  |  | 8993 | 0.000 | -0.3197 | No |
| 86 | CXCL6 |  |  | 8999 | 0.000 | -0.3200 | No |
| 87 | LHCGR |  |  | 9014 | 0.000 | -0.3209 | No |
| 88 | OPN5 |  |  | 9015 | 0.000 | -0.3209 | No |
| 89 | GPRC6A |  |  | 9019 | 0.000 | -0.3211 | No |
| 90 | RXFP1 |  |  | 9086 | 0.000 | -0.3254 | No |
| 91 | WNT3 |  |  | 9098 | 0.000 | -0.3261 | No |
| 92 | PROKR2 |  |  | 9145 | 0.000 | -0.3291 | No |
| 93 | NPFFR2 |  |  | 9150 | 0.000 | -0.3294 | No |
| 94 | TRH |  |  | 9182 | 0.000 | -0.3314 | No |
| 95 | GIP |  |  | 9204 | 0.000 | -0.3327 | No |
| 96 | MTNR1A |  |  | 9273 | 0.000 | -0.3372 | No |
| 97 | GABBR2 |  |  | 9284 | 0.000 | -0.3378 | No |
| 98 | TAC3 |  |  | 9300 | 0.000 | -0.3388 | No |
| 99 | WNT9B |  |  | 9337 | 0.000 | -0.3411 | No |
| 100 | GRM1 |  |  | 9338 | 0.000 | -0.3411 | No |
| 101 | RGR |  |  | 9347 | 0.000 | -0.3416 | No |
| 102 | HTR2A |  |  | 9366 | 0.000 | -0.3428 | No |
| 103 | UTS2R |  |  | 9367 | 0.000 | -0.3428 | No |
| 104 | NPY5R |  |  | 9385 | 0.000 | -0.3439 | No |
| 105 | HCRTR2 |  |  | 9388 | 0.000 | -0.3440 | No |
| 106 | DRD1 |  |  | 9397 | 0.000 | -0.3446 | No |
| 107 | TSHB |  |  | 9452 | 0.000 | -0.3481 | No |
| 108 | CALCA |  |  | 9487 | 0.000 | -0.3503 | No |
| 109 | NMUR2 |  |  | 9495 | 0.000 | -0.3507 | No |
| 110 | MC4R |  |  | 9554 | 0.000 | -0.3545 | No |
| 111 | RHO |  |  | 9573 | 0.000 | -0.3557 | No |
| 112 | GRM2 |  |  | 9597 | 0.000 | -0.3572 | No |
| 113 | OPRM1 |  |  | 9605 | 0.000 | -0.3576 | No |
| 114 | CCR3 |  |  | 9606 | 0.000 | -0.3576 | No |
| 115 | MC5R |  |  | 9630 | 0.000 | -0.3591 | No |
| 116 | HTR7 |  |  | 9637 | 0.000 | -0.3595 | No |
| 117 | AGTR2 |  |  | 9655 | 0.000 | -0.3606 | No |
| 118 | HTR1F |  |  | 9658 | 0.000 | -0.3607 | No |
| 119 | NMBR |  |  | 9686 | 0.000 | -0.3625 | No |
| 120 | RXFP3 |  |  | 9707 | 0.000 | -0.3638 | No |
| 121 | C5 |  |  | 9718 | 0.000 | -0.3644 | No |
| 122 | HRH3 |  |  | 9732 | 0.000 | -0.3653 | No |
| 123 | MC2R |  |  | 9747 | 0.000 | -0.3662 | No |
| 124 | RRH |  |  | 9788 | 0.000 | -0.3688 | No |
| 125 | GNB3 |  |  | 9821 | 0.000 | -0.3709 | No |
| 126 | HTR1D |  |  | 9834 | 0.000 | -0.3716 | No |
| 127 | CXCL3 |  |  | 9838 | 0.000 | -0.3718 | No |
| 128 | GRM6 |  |  | 9840 | 0.000 | -0.3719 | No |
| 129 | GHRHR |  |  | 9841 | 0.000 | -0.3719 | No |
| 130 | CASR |  |  | 9851 | 0.000 | -0.3725 | No |
| 131 | WNT7B |  |  | 9876 | 0.000 | -0.3740 | No |
| 132 | AVP |  |  | 9946 | 0.000 | -0.3785 | No |
| 133 | CXCL11 |  |  | 9974 | 0.000 | -0.3803 | No |
| 134 | ADORA3 |  |  | 10011 | 0.000 | -0.3826 | No |
| 135 | WNT16 |  |  | 10015 | 0.000 | -0.3828 | No |
| 136 | DRD2 |  |  | 10026 | 0.000 | -0.3835 | No |
| 137 | GHRH |  |  | 10029 | 0.000 | -0.3836 | No |
| 138 | NMB |  |  | 10045 | 0.000 | -0.3846 | No |
| 139 | CCKBR |  |  | 10084 | 0.000 | -0.3870 | No |
| 140 | RLN3 |  |  | 10099 | 0.000 | -0.3879 | No |
| 141 | FPR1 |  |  | 10156 | -0.100 | -0.3914 | No |
| 142 | NPBWR1 |  |  | 10190 | -0.100 | -0.3933 | No |
| 143 | XCR1 |  |  | 10268 | -0.100 | -0.3981 | No |
| 144 | P2RY10 |  |  | 10322 | -0.100 | -0.4013 | No |
| 145 | ADRA1A |  |  | 10328 | -0.100 | -0.4015 | No |
| 146 | PTGFR |  |  | 10339 | -0.100 | -0.4019 | No |
| 147 | OPRK1 |  |  | 10357 | -0.100 | -0.4028 | No |
| 148 | CCL4 |  |  | 10361 | -0.100 | -0.4028 | No |
| 149 | AVPR2 |  |  | 10377 | -0.100 | -0.4036 | No |
| 150 | PNOC |  |  | 10385 | -0.100 | -0.4038 | No |
| 151 | NPW |  |  | 10389 | -0.100 | -0.4038 | No |
| 152 | CCR8 |  |  | 10423 | -0.100 | -0.4057 | No |
| 153 | ADCYAP1 |  |  | 10435 | -0.100 | -0.4062 | No |
| 154 | GPR77 |  |  | 10438 | -0.100 | -0.4062 | No |
| 155 | GRM7 |  |  | 10450 | -0.100 | -0.4067 | No |
| 156 | CRHR2 |  |  | 10452 | -0.100 | -0.4065 | No |
| 157 | RXFP4 |  |  | 10457 | -0.100 | -0.4066 | No |
| 158 | TACR3 |  |  | 10461 | -0.100 | -0.4066 | No |
| 159 | HTR6 |  |  | 10490 | -0.100 | -0.4082 | No |
| 160 | CYSLTR1 |  |  | 10497 | -0.100 | -0.4084 | No |
| 161 | CYSLTR2 |  |  | 10507 | -0.100 | -0.4087 | No |
| 162 | HRH2 |  |  | 10527 | -0.100 | -0.4098 | No |
| 163 | CCR9 |  |  | 10533 | -0.100 | -0.4099 | No |
| 164 | CXCL9 |  |  | 10552 | -0.100 | -0.4108 | No |
| 165 | GRM4 |  |  | 10561 | -0.100 | -0.4112 | No |
| 166 | GNG3 |  |  | 10566 | -0.100 | -0.4112 | No |
| 167 | PRLHR |  |  | 10601 | -0.100 | -0.4132 | No |
| 168 | SCTR |  |  | 10620 | -0.100 | -0.4142 | No |
| 169 | PDYN |  |  | 10628 | -0.100 | -0.4144 | No |
| 170 | GNG7 |  |  | 10645 | -0.100 | -0.4153 | No |
| 171 | CCR4 |  |  | 10656 | -0.100 | -0.4157 | No |
| 172 | LOC728830 |  |  | 10672 | -0.100 | -0.4165 | No |
| 173 | HCRT |  |  | 10681 | -0.100 | -0.4168 | No |
| 174 | DRD3 |  |  | 10688 | -0.100 | -0.4170 | No |
| 175 | GLP1R |  |  | 10729 | -0.100 | -0.4194 | No |
| 176 | GCGR |  |  | 10768 | -0.100 | -0.4216 | No |
| 177 | TSHR |  |  | 10809 | -0.200 | -0.4238 | No |
| 178 | GPR55 |  |  | 10815 | -0.200 | -0.4237 | No |
| 179 | XCL1 |  |  | 10837 | -0.200 | -0.4247 | No |
| 180 | WNT8B |  |  | 10854 | -0.200 | -0.4253 | No |
| 181 | GALR3 |  |  | 10878 | -0.200 | -0.4264 | No |
| 182 | OXTR |  |  | 10880 | -0.200 | -0.4260 | No |
| 183 | POMC |  |  | 10894 | -0.200 | -0.4264 | No |
| 184 | CCR2 |  |  | 10919 | -0.200 | -0.4276 | No |
| 185 | OPRL1 |  |  | 10930 | -0.200 | -0.4278 | No |
| 186 | SHH |  |  | 10965 | -0.200 | -0.4296 | No |
| 187 | GALR1 |  |  | 10975 | -0.200 | -0.4298 | No |
| 188 | CCL7 |  |  | 11021 | -0.200 | -0.4323 | No |
| 189 | WNT9A |  |  | 11031 | -0.200 | -0.4325 | No |
| 190 | AVPR1B |  |  | 11056 | -0.200 | -0.4336 | No |
| 191 | ADRA1B |  |  | 11074 | -0.200 | -0.4343 | No |
| 192 | GPR65 |  |  | 11081 | -0.200 | -0.4343 | No |
| 193 | HTR2B |  |  | 11086 | -0.200 | -0.4341 | No |
| 194 | CXCR6 |  |  | 11100 | -0.200 | -0.4346 | No |
| 195 | HTR1B |  |  | 11107 | -0.200 | -0.4345 | No |
| 196 | WNT2 |  |  | 11109 | -0.200 | -0.4342 | No |
| 197 | CCL2 |  |  | 11114 | -0.200 | -0.4340 | No |
| 198 | F2RL2 |  |  | 11120 | -0.200 | -0.4340 | No |
| 199 | GRPR |  |  | 11139 | -0.200 | -0.4347 | No |
| 200 | FZD7 |  |  | 11141 | -0.200 | -0.4344 | No |
| 201 | NPY2R |  |  | 11142 | -0.200 | -0.4339 | No |
| 202 | P2RY12 |  |  | 11167 | -0.200 | -0.4351 | No |
| 203 | SSTR5 |  |  | 11181 | -0.300 | -0.4353 | No |
| 204 | FZD10 |  |  | 11184 | -0.300 | -0.4348 | No |
| 205 | SSTR3 |  |  | 11204 | -0.300 | -0.4354 | No |
| 206 | GNGT2 |  |  | 11299 | -0.300 | -0.4409 | No |
| 207 | F2 |  |  | 11305 | -0.300 | -0.4406 | No |
| 208 | TACR1 |  |  | 11308 | -0.300 | -0.4401 | No |
| 209 | NPFFR1 |  |  | 11432 | -0.300 | -0.4475 | No |
| 210 | CCL19 |  |  | 11443 | -0.300 | -0.4475 | No |
| 211 | CNR1 |  |  | 11468 | -0.400 | -0.4483 | No |
| 212 | ADORA2B |  |  | 11483 | -0.400 | -0.4484 | No |
| 213 | P2RY13 |  |  | 11489 | -0.400 | -0.4479 | No |
| 214 | NTS |  |  | 11525 | -0.400 | -0.4493 | No |
| 215 | GNB4 |  |  | 11555 | -0.400 | -0.4504 | No |
| 216 | BDKRB1 |  |  | 11619 | -0.400 | -0.4536 | No |
| 217 | OPRD1 |  |  | 11624 | -0.400 | -0.4531 | No |
| 218 | CD55 |  |  | 11649 | -0.400 | -0.4538 | No |
| 219 | NTSR1 |  |  | 11651 | -0.400 | -0.4530 | No |
| 220 | FFAR1 |  |  | 11661 | -0.500 | -0.4526 | No |
| 221 | CCR6 |  |  | 11662 | -0.500 | -0.4515 | No |
| 222 | CNR2 |  |  | 11682 | -0.500 | -0.4517 | No |
| 223 | GIPR |  |  | 11698 | -0.500 | -0.4517 | No |
| 224 | DHH |  |  | 11704 | -0.500 | -0.4510 | No |
| 225 | SSTR4 |  |  | 11739 | -0.500 | -0.4522 | No |
| 226 | IAPP |  |  | 11750 | -0.500 | -0.4518 | No |
| 227 | LTB4R |  |  | 11764 | -0.500 | -0.4516 | No |
| 228 | EDN1 |  |  | 11777 | -0.500 | -0.4513 | No |
| 229 | PRLH |  |  | 11811 | -0.500 | -0.4524 | No |
| 230 | ADRB2 |  |  | 11843 | -0.600 | -0.4532 | No |
| 231 | CALCB |  |  | 11861 | -0.600 | -0.4531 | No |
| 232 | LTB4R2 |  |  | 11943 | -0.600 | -0.4571 | No |
| 233 | GLP2R |  |  | 11954 | -0.600 | -0.4565 | No |
| 234 | C3AR1 |  |  | 11965 | -0.600 | -0.4559 | No |
| 235 | GNG4 |  |  | 11993 | -0.600 | -0.4564 | No |
| 236 | NPY1R |  |  | 12054 | -0.700 | -0.4589 | No |
| 237 | CCBP2 |  |  | 12069 | -0.700 | -0.4583 | No |
| 238 | MCHR1 |  |  | 12108 | -0.700 | -0.4594 | No |
| 239 | CCL20 |  |  | 12138 | -0.700 | -0.4598 | Yes |
| 240 | WNT11 |  |  | 12139 | -0.700 | -0.4584 | Yes |
| 241 | EDNRB |  |  | 12155 | -0.700 | -0.4579 | Yes |
| 242 | GNG2 |  |  | 12174 | -0.800 | -0.4574 | Yes |
| 243 | KISS1 |  |  | 12176 | -0.800 | -0.4558 | Yes |
| 244 | EDN3 |  |  | 12211 | -0.800 | -0.4564 | Yes |
| 245 | FFAR3 |  |  | 12216 | -0.800 | -0.4550 | Yes |
| 246 | PTGER2 |  |  | 12243 | -0.800 | -0.4550 | Yes |
| 247 | ADORA1 |  |  | 12252 | -0.800 | -0.4539 | Yes |
| 248 | CHRM3 |  |  | 12254 | -0.800 | -0.4523 | Yes |
| 249 | CCL21 |  |  | 12271 | -0.800 | -0.4517 | Yes |
| 250 | CCL22 |  |  | 12279 | -0.800 | -0.4505 | Yes |
| 251 | TAS1R3 |  |  | 12296 | -0.800 | -0.4499 | Yes |
| 252 | VIPR2 |  |  | 12317 | -0.900 | -0.4493 | Yes |
| 253 | CXCL13 |  |  | 12321 | -0.900 | -0.4476 | Yes |
| 254 | PTGIR |  |  | 12396 | -0.900 | -0.4506 | Yes |
| 255 | AVPR1A |  |  | 12399 | -0.900 | -0.4488 | Yes |
| 256 | NMUR1 |  |  | 12483 | -1.000 | -0.4522 | Yes |
| 257 | GPR18 |  |  | 12538 | -1.000 | -0.4536 | Yes |
| 258 | P2RY2 |  |  | 12549 | -1.000 | -0.4522 | Yes |
| 259 | WNT6 |  |  | 12563 | -1.000 | -0.4510 | Yes |
| 260 | GPR68 |  |  | 12582 | -1.000 | -0.4501 | Yes |
| 261 | CXCR3 |  |  | 12696 | -1.100 | -0.4551 | Yes |
| 262 | RAMP3 |  |  | 12698 | -1.100 | -0.4529 | Yes |
| 263 | ADORA2A |  |  | 12713 | -1.200 | -0.4514 | Yes |
| 264 | CCR7 |  |  | 12772 | -1.200 | -0.4526 | Yes |
| 265 | GNB5 |  |  | 12793 | -1.200 | -0.4515 | Yes |
| 266 | CCKAR |  |  | 12831 | -1.300 | -0.4512 | Yes |
| 267 | GPR17 |  |  | 12880 | -1.300 | -0.4516 | Yes |
| 268 | HCRTR1 |  |  | 12888 | -1.300 | -0.4494 | Yes |
| 269 | CHRM2 |  |  | 12903 | -1.300 | -0.4476 | Yes |
| 270 | GPR39 |  |  | 12912 | -1.400 | -0.4452 | Yes |
| 271 | EMR1 |  |  | 12951 | -1.400 | -0.4448 | Yes |
| 272 | GRP |  |  | 12978 | -1.400 | -0.4436 | Yes |
| 273 | GPR132 |  |  | 12986 | -1.400 | -0.4412 | Yes |
| 274 | HTR4 |  |  | 12993 | -1.400 | -0.4387 | Yes |
| 275 | GPR4 |  |  | 13015 | -1.500 | -0.4369 | Yes |
| 276 | P2RY4 |  |  | 13016 | -1.500 | -0.4338 | Yes |
| 277 | OPN3 |  |  | 13031 | -1.500 | -0.4316 | Yes |
| 278 | APP |  |  | 13046 | -1.500 | -0.4294 | Yes |
| 279 | NMU |  |  | 13073 | -1.500 | -0.4280 | Yes |
| 280 | CCL28 |  |  | 13098 | -1.600 | -0.4263 | Yes |
| 281 | AGTR1 |  |  | 13128 | -1.600 | -0.4249 | Yes |
| 282 | EDNRA |  |  | 13135 | -1.600 | -0.4219 | Yes |
| 283 | PENK |  |  | 13156 | -1.600 | -0.4199 | Yes |
| 284 | ADCYAP1R1 |  |  | 13187 | -1.700 | -0.4184 | Yes |
| 285 | FZD4 |  |  | 13203 | -1.700 | -0.4158 | Yes |
| 286 | KISS1R |  |  | 13212 | -1.700 | -0.4128 | Yes |
| 287 | GALR2 |  |  | 13227 | -1.700 | -0.4102 | Yes |
| 288 | PTCH2 |  |  | 13245 | -1.700 | -0.4078 | Yes |
| 289 | CCL27 |  |  | 13347 | -1.800 | -0.4107 | Yes |
| 290 | GPBAR1 |  |  | 13500 | -2.000 | -0.4164 | Yes |
| 291 | NPY |  |  | 13555 | -2.100 | -0.4156 | Yes |
| 292 | CX3CR1 |  |  | 13586 | -2.100 | -0.4132 | Yes |
| 293 | SSTR2 |  |  | 13633 | -2.200 | -0.4116 | Yes |
| 294 | CHRM1 |  |  | 13644 | -2.200 | -0.4077 | Yes |
| 295 | CCL11 |  |  | 13654 | -2.200 | -0.4038 | Yes |
| 296 | CCK |  |  | 13672 | -2.300 | -0.4001 | Yes |
| 297 | PTAFR |  |  | 13676 | -2.300 | -0.3956 | Yes |
| 298 | CCL5 |  |  | 13706 | -2.300 | -0.3927 | Yes |
| 299 | CCR10 |  |  | 13708 | -2.300 | -0.3880 | Yes |
| 300 | F2RL3 |  |  | 13786 | -2.400 | -0.3880 | Yes |
| 301 | ADRB1 |  |  | 13803 | -2.500 | -0.3839 | Yes |
| 302 | BDKRB2 |  |  | 13805 | -2.500 | -0.3788 | Yes |
| 303 | PTGER3 |  |  | 13814 | -2.500 | -0.3742 | Yes |
| 304 | CCRL1 |  |  | 13834 | -2.500 | -0.3702 | Yes |
| 305 | FZD9 |  |  | 13836 | -2.500 | -0.3651 | Yes |
| 306 | PPYR1 |  |  | 13845 | -2.600 | -0.3603 | Yes |
| 307 | C5AR1 |  |  | 13884 | -2.600 | -0.3574 | Yes |
| 308 | WNT5A |  |  | 13923 | -2.700 | -0.3543 | Yes |
| 309 | TAC1 |  |  | 13930 | -2.700 | -0.3491 | Yes |
| 310 | INSL3 |  |  | 13939 | -2.700 | -0.3440 | Yes |
| 311 | GNG11 |  |  | 14019 | -2.800 | -0.3434 | Yes |
| 312 | FZD2 |  |  | 14070 | -2.900 | -0.3406 | Yes |
| 313 | DARC |  |  | 14153 | -3.000 | -0.3397 | Yes |
| 314 | ADM |  |  | 14162 | -3.000 | -0.3341 | Yes |
| 315 | PPY |  |  | 14193 | -3.100 | -0.3296 | Yes |
| 316 | P2RY1 |  |  | 14216 | -3.100 | -0.3246 | Yes |
| 317 | CXCL12 |  |  | 14228 | -3.200 | -0.3187 | Yes |
| 318 | CXCR7 |  |  | 14254 | -3.200 | -0.3137 | Yes |
| 319 | IHH |  |  | 14312 | -3.400 | -0.3104 | Yes |
| 320 | SCT |  |  | 14335 | -3.400 | -0.3048 | Yes |
| 321 | CXCR4 |  |  | 14374 | -3.500 | -0.3000 | Yes |
| 322 | RAMP2 |  |  | 14395 | -3.600 | -0.2939 | Yes |
| 323 | WNT4 |  |  | 14418 | -3.700 | -0.2877 | Yes |
| 324 | VIP |  |  | 14431 | -3.700 | -0.2808 | Yes |
| 325 | PF4 |  |  | 14433 | -3.700 | -0.2732 | Yes |
| 326 | PTGER4 |  |  | 14532 | -3.900 | -0.2715 | Yes |
| 327 | WNT2B |  |  | 14615 | -4.100 | -0.2684 | Yes |
| 328 | FZD8 |  |  | 14636 | -4.200 | -0.2610 | Yes |
| 329 | ADRB3 |  |  | 14670 | -4.300 | -0.2543 | Yes |
| 330 | P2RY6 |  |  | 14706 | -4.300 | -0.2477 | Yes |
| 331 | ADRA2A |  |  | 14754 | -4.500 | -0.2414 | Yes |
| 332 | SAA1 |  |  | 14809 | -4.600 | -0.2354 | Yes |
| 333 | TBXA2R |  |  | 14827 | -4.700 | -0.2268 | Yes |
| 334 | FFAR2 |  |  | 14833 | -4.700 | -0.2174 | Yes |
| 335 | AGT |  |  | 14844 | -4.700 | -0.2084 | Yes |
| 336 | TACR2 |  |  | 14856 | -4.700 | -0.1994 | Yes |
| 337 | CXCL16 |  |  | 14924 | -4.900 | -0.1936 | Yes |
| 338 | FZD5 |  |  | 15076 | -5.500 | -0.1920 | Yes |
| 339 | INSL5 |  |  | 15099 | -5.700 | -0.1817 | Yes |
| 340 | GAL |  |  | 15193 | -6.100 | -0.1751 | Yes |
| 341 | CX3CL1 |  |  | 15197 | -6.200 | -0.1625 | Yes |
| 342 | SSTR1 |  |  | 15216 | -6.300 | -0.1506 | Yes |
| 343 | EDN2 |  |  | 15252 | -6.400 | -0.1397 | Yes |
| 344 | PYY |  |  | 15309 | -6.800 | -0.1293 | Yes |
| 345 | PTGER1 |  |  | 15335 | -7.000 | -0.1164 | Yes |
| 346 | GCG |  |  | 15351 | -7.100 | -0.1027 | Yes |
| 347 | FZD1 |  |  | 15355 | -7.100 | -0.0883 | Yes |
| 348 | RAMP1 |  |  | 15374 | -7.300 | -0.0743 | Yes |
| 349 | F2RL1 |  |  | 15456 | -8.000 | -0.0631 | Yes |
| 350 | VIPR1 |  |  | 15540 | -9.200 | -0.0494 | Yes |
| 351 | CD97 |  |  | 15585 | -9.900 | -0.0318 | Yes |
| 352 | SST |  |  | 15599 | -10.200 | -0.0116 | Yes |
| 353 | C3 |  |  | 15605 | -10.300 | 0.0094 | Yes |
Table: GSEA details [plain text format]

  

Fig 2: REACTOME\_GPCR\_LIGAND\_BINDING: Random ES distribution      
 Gene set null distribution of ES for **REACTOME\_GPCR\_LIGAND\_BINDING**

  
