# Supplementary material for: Immunomic, genomic and transcriptomic characterization of CT26 colorectal carcinoma
Source: BMC Genomics. 2014 Mar 13;15(1):190. doi: 10.1186/1471-2164-15-190 (PMC4007559; doi:10.1186/1471-2164-15-190)
Supplement: Supplementary file 8 — Additional file 8: Contains the Gene Pattern gene set membership and enrichment values in an html format. The file index.html is the entry point. (ZIP 13 MB) [file 12864_2013_7028_MOESM8_ESM.zip › REACTOME_G_ALPHA_I_SIGNALLING_EVENTS.html]

Details for gene set REACTOME\_G\_ALPHA\_I\_SIGNALLING\_EVENTS[GSEA]

|  || Dataset | CT26\_gene\_expression |
| Phenotype | NoPhenotypeAvailable |
| Upregulated in class | na\_neg |
| GeneSet | REACTOME\_G\_ALPHA\_I\_SIGNALLING\_EVENTS |
| Enrichment Score (ES) | -0.33706623 |
| Normalized Enrichment Score (NES) | NaN |
| Nominal p-value | NaN |
| FDR q-value | 1.0 |
| FWER p-Value | 0.0 |
Table: GSEA Results Summary

  

Fig 1: Enrichment plot: REACTOME\_G\_ALPHA\_I\_SIGNALLING\_EVENTS      
 Profile of the Running ES Score & Positions of GeneSet Members on the Rank Ordered List

  

| PROBE | GENE SYMBOL | GENE\_TITLE | RANK IN GENE LIST | RANK METRIC SCORE | RUNNING ES | CORE ENRICHMENT || 1 | ANXA1 |  |  | 697 | 16.400 | 0.0162 | No |
| 2 | GNAI3 |  |  | 884 | 14.900 | 0.0597 | No |
| 3 | HEBP1 |  |  | 1978 | 9.700 | 0.0256 | No |
| 4 | RGS19 |  |  | 2034 | 9.500 | 0.0574 | No |
| 5 | GNG10 |  |  | 2412 | 8.500 | 0.0648 | No |
| 6 | GNB1 |  |  | 3457 | 5.800 | 0.0194 | No |
| 7 | PMCH |  |  | 3480 | 5.800 | 0.0396 | No |
| 8 | GNG5 |  |  | 3909 | 4.900 | 0.0303 | No |
| 9 | GNG12 |  |  | 4271 | 4.300 | 0.0232 | No |
| 10 | RGS20 |  |  | 4387 | 4.100 | 0.0310 | No |
| 11 | GABBR1 |  |  | 4585 | 3.700 | 0.0321 | No |
| 12 | GNB2 |  |  | 5311 | 2.600 | -0.0047 | No |
| 13 | GNG13 |  |  | 5348 | 2.500 | 0.0023 | No |
| 14 | RGS12 |  |  | 5535 | 2.200 | -0.0015 | No |
| 15 | GNAS |  |  | 5595 | 2.200 | 0.0029 | No |
| 16 | ADCY7 |  |  | 5626 | 2.100 | 0.0088 | No |
| 17 | GNG8 |  |  | 5784 | 1.900 | 0.0058 | No |
| 18 | CCL25 |  |  | 5788 | 1.900 | 0.0127 | No |
| 19 | CXCL2 |  |  | 6388 | 1.100 | -0.0217 | No |
| 20 | CCR1 |  |  | 6492 | 0.900 | -0.0249 | No |
| 21 | GPR44 |  |  | 6826 | 0.600 | -0.0441 | No |
| 22 | GNAT2 |  |  | 6950 | 0.400 | -0.0505 | No |
| 23 | ADRA2B |  |  | 7044 | 0.400 | -0.0550 | No |
| 24 | CXCL10 |  |  | 7089 | 0.300 | -0.0567 | No |
| 25 | P2RY14 |  |  | 7245 | 0.200 | -0.0659 | No |
| 26 | PPBP |  |  | 7319 | 0.100 | -0.0702 | No |
| 27 | OPN1SW |  |  | 7624 | 0.000 | -0.0897 | No |
| 28 | HRH4 |  |  | 7848 | 0.000 | -0.1040 | No |
| 29 | GNGT1 |  |  | 8304 | 0.000 | -0.1332 | No |
| 30 | HTR1A |  |  | 8333 | 0.000 | -0.1350 | No |
| 31 | MTNR1B |  |  | 8469 | 0.000 | -0.1436 | No |
| 32 | NMS |  |  | 8485 | 0.000 | -0.1446 | No |
| 33 | OPN1LW |  |  | 8753 | 0.000 | -0.1617 | No |
| 34 | KNG1 |  |  | 8986 | 0.000 | -0.1766 | No |
| 35 | HTR5A |  |  | 8989 | 0.000 | -0.1767 | No |
| 36 | CXCL6 |  |  | 8999 | 0.000 | -0.1773 | No |
| 37 | OPN5 |  |  | 9015 | 0.000 | -0.1783 | No |
| 38 | MTNR1A |  |  | 9273 | 0.000 | -0.1948 | No |
| 39 | GABBR2 |  |  | 9284 | 0.000 | -0.1954 | No |
| 40 | RGR |  |  | 9347 | 0.000 | -0.1994 | No |
| 41 | NPY5R |  |  | 9385 | 0.000 | -0.2018 | No |
| 42 | NMUR2 |  |  | 9495 | 0.000 | -0.2088 | No |
| 43 | RHO |  |  | 9573 | 0.000 | -0.2137 | No |
| 44 | OPRM1 |  |  | 9605 | 0.000 | -0.2157 | No |
| 45 | CCR3 |  |  | 9606 | 0.000 | -0.2157 | No |
| 46 | ADCY8 |  |  | 9623 | 0.000 | -0.2167 | No |
| 47 | RGS18 |  |  | 9653 | 0.000 | -0.2186 | No |
| 48 | AGTR2 |  |  | 9655 | 0.000 | -0.2186 | No |
| 49 | HTR1F |  |  | 9658 | 0.000 | -0.2188 | No |
| 50 | RXFP3 |  |  | 9707 | 0.000 | -0.2218 | No |
| 51 | C5 |  |  | 9718 | 0.000 | -0.2225 | No |
| 52 | HRH3 |  |  | 9732 | 0.000 | -0.2233 | No |
| 53 | RRH |  |  | 9788 | 0.000 | -0.2269 | No |
| 54 | GNB3 |  |  | 9821 | 0.000 | -0.2289 | No |
| 55 | HTR1D |  |  | 9834 | 0.000 | -0.2297 | No |
| 56 | CXCL3 |  |  | 9838 | 0.000 | -0.2299 | No |
| 57 | CASR |  |  | 9851 | 0.000 | -0.2306 | No |
| 58 | RGS7 |  |  | 9872 | 0.000 | -0.2319 | No |
| 59 | CXCL11 |  |  | 9974 | 0.000 | -0.2384 | No |
| 60 | ADORA3 |  |  | 10011 | 0.000 | -0.2407 | No |
| 61 | DRD2 |  |  | 10026 | 0.000 | -0.2416 | No |
| 62 | RLN3 |  |  | 10099 | 0.000 | -0.2462 | No |
| 63 | FPR1 |  |  | 10156 | -0.100 | -0.2494 | No |
| 64 | NPBWR1 |  |  | 10190 | -0.100 | -0.2512 | No |
| 65 | OPRK1 |  |  | 10357 | -0.100 | -0.2615 | No |
| 66 | PNOC |  |  | 10385 | -0.100 | -0.2628 | No |
| 67 | NPW |  |  | 10389 | -0.100 | -0.2627 | No |
| 68 | CCR8 |  |  | 10423 | -0.100 | -0.2644 | No |
| 69 | RXFP4 |  |  | 10457 | -0.100 | -0.2661 | No |
| 70 | CCR9 |  |  | 10533 | -0.100 | -0.2706 | No |
| 71 | CXCL9 |  |  | 10552 | -0.100 | -0.2714 | No |
| 72 | GNG3 |  |  | 10566 | -0.100 | -0.2718 | No |
| 73 | GNAT1 |  |  | 10570 | -0.100 | -0.2717 | No |
| 74 | RGS9 |  |  | 10575 | -0.100 | -0.2715 | No |
| 75 | PDYN |  |  | 10628 | -0.100 | -0.2745 | No |
| 76 | GNG7 |  |  | 10645 | -0.100 | -0.2752 | No |
| 77 | CCR4 |  |  | 10656 | -0.100 | -0.2754 | No |
| 78 | DRD3 |  |  | 10688 | -0.100 | -0.2770 | No |
| 79 | RGS8 |  |  | 10735 | -0.100 | -0.2796 | No |
| 80 | GPR55 |  |  | 10815 | -0.200 | -0.2839 | No |
| 81 | RGS6 |  |  | 10822 | -0.200 | -0.2836 | No |
| 82 | ADCY1 |  |  | 10858 | -0.200 | -0.2851 | No |
| 83 | GALR3 |  |  | 10878 | -0.200 | -0.2856 | No |
| 84 | POMC |  |  | 10894 | -0.200 | -0.2858 | No |
| 85 | CCR2 |  |  | 10919 | -0.200 | -0.2866 | No |
| 86 | OPRL1 |  |  | 10930 | -0.200 | -0.2865 | No |
| 87 | GALR1 |  |  | 10975 | -0.200 | -0.2886 | No |
| 88 | CXCR6 |  |  | 11100 | -0.200 | -0.2958 | No |
| 89 | HTR1B |  |  | 11107 | -0.200 | -0.2954 | No |
| 90 | NPY2R |  |  | 11142 | -0.200 | -0.2968 | No |
| 91 | P2RY12 |  |  | 11167 | -0.200 | -0.2976 | No |
| 92 | SSTR5 |  |  | 11181 | -0.300 | -0.2974 | No |
| 93 | SSTR3 |  |  | 11204 | -0.300 | -0.2977 | No |
| 94 | GNGT2 |  |  | 11299 | -0.300 | -0.3026 | No |
| 95 | CCL19 |  |  | 11443 | -0.300 | -0.3106 | No |
| 96 | CNR1 |  |  | 11468 | -0.400 | -0.3107 | No |
| 97 | P2RY13 |  |  | 11489 | -0.400 | -0.3105 | No |
| 98 | GNB4 |  |  | 11555 | -0.400 | -0.3132 | No |
| 99 | ADCY2 |  |  | 11597 | -0.400 | -0.3143 | No |
| 100 | RGS1 |  |  | 11607 | -0.400 | -0.3134 | No |
| 101 | BDKRB1 |  |  | 11619 | -0.400 | -0.3126 | No |
| 102 | OPRD1 |  |  | 11624 | -0.400 | -0.3114 | No |
| 103 | CCR6 |  |  | 11662 | -0.500 | -0.3119 | No |
| 104 | CNR2 |  |  | 11682 | -0.500 | -0.3113 | No |
| 105 | RGS4 |  |  | 11703 | -0.500 | -0.3107 | No |
| 106 | SSTR4 |  |  | 11739 | -0.500 | -0.3111 | No |
| 107 | C3AR1 |  |  | 11965 | -0.600 | -0.3233 | No |
| 108 | GNG4 |  |  | 11993 | -0.600 | -0.3228 | No |
| 109 | NPY1R |  |  | 12054 | -0.700 | -0.3240 | No |
| 110 | MCHR1 |  |  | 12108 | -0.700 | -0.3248 | No |
| 111 | CCL20 |  |  | 12138 | -0.700 | -0.3241 | No |
| 112 | GNAI2 |  |  | 12163 | -0.700 | -0.3230 | No |
| 113 | GNG2 |  |  | 12174 | -0.800 | -0.3207 | No |
| 114 | ADORA1 |  |  | 12252 | -0.800 | -0.3227 | No |
| 115 | CCL21 |  |  | 12271 | -0.800 | -0.3208 | No |
| 116 | CXCL13 |  |  | 12321 | -0.900 | -0.3206 | No |
| 117 | NMUR1 |  |  | 12483 | -1.000 | -0.3272 | No |
| 118 | GPR18 |  |  | 12538 | -1.000 | -0.3270 | No |
| 119 | CXCR3 |  |  | 12696 | -1.100 | -0.3330 | Yes |
| 120 | RGS11 |  |  | 12733 | -1.200 | -0.3308 | Yes |
| 121 | CCR7 |  |  | 12772 | -1.200 | -0.3288 | Yes |
| 122 | GNB5 |  |  | 12793 | -1.200 | -0.3256 | Yes |
| 123 | ADCY4 |  |  | 12802 | -1.200 | -0.3217 | Yes |
| 124 | GNAZ |  |  | 12843 | -1.300 | -0.3194 | Yes |
| 125 | GPR17 |  |  | 12880 | -1.300 | -0.3169 | Yes |
| 126 | CHRM2 |  |  | 12903 | -1.300 | -0.3135 | Yes |
| 127 | ADCY3 |  |  | 12965 | -1.400 | -0.3122 | Yes |
| 128 | P2RY4 |  |  | 13016 | -1.500 | -0.3098 | Yes |
| 129 | OPN3 |  |  | 13031 | -1.500 | -0.3051 | Yes |
| 130 | APP |  |  | 13046 | -1.500 | -0.3005 | Yes |
| 131 | NMU |  |  | 13073 | -1.500 | -0.2965 | Yes |
| 132 | CCL28 |  |  | 13098 | -1.600 | -0.2921 | Yes |
| 133 | PENK |  |  | 13156 | -1.600 | -0.2898 | Yes |
| 134 | GALR2 |  |  | 13227 | -1.700 | -0.2880 | Yes |
| 135 | CCL27 |  |  | 13347 | -1.800 | -0.2890 | Yes |
| 136 | NPY |  |  | 13555 | -2.100 | -0.2944 | Yes |
| 137 | GNAI1 |  |  | 13615 | -2.200 | -0.2900 | Yes |
| 138 | SSTR2 |  |  | 13633 | -2.200 | -0.2830 | Yes |
| 139 | ADCY9 |  |  | 13683 | -2.300 | -0.2775 | Yes |
| 140 | CCL5 |  |  | 13706 | -2.300 | -0.2704 | Yes |
| 141 | CCR10 |  |  | 13708 | -2.300 | -0.2619 | Yes |
| 142 | BDKRB2 |  |  | 13805 | -2.500 | -0.2588 | Yes |
| 143 | PTGER3 |  |  | 13814 | -2.500 | -0.2500 | Yes |
| 144 | PPYR1 |  |  | 13845 | -2.600 | -0.2423 | Yes |
| 145 | C5AR1 |  |  | 13884 | -2.600 | -0.2350 | Yes |
| 146 | GNG11 |  |  | 14019 | -2.800 | -0.2332 | Yes |
| 147 | PPY |  |  | 14193 | -3.100 | -0.2328 | Yes |
| 148 | CXCL12 |  |  | 14228 | -3.200 | -0.2231 | Yes |
| 149 | CXCR7 |  |  | 14254 | -3.200 | -0.2128 | Yes |
| 150 | CXCR4 |  |  | 14374 | -3.500 | -0.2074 | Yes |
| 151 | PF4 |  |  | 14433 | -3.700 | -0.1974 | Yes |
| 152 | ADRA2A |  |  | 14754 | -4.500 | -0.2012 | Yes |
| 153 | SAA1 |  |  | 14809 | -4.600 | -0.1875 | Yes |
| 154 | ADCY6 |  |  | 14830 | -4.700 | -0.1714 | Yes |
| 155 | AGT |  |  | 14844 | -4.700 | -0.1547 | Yes |
| 156 | CXCL16 |  |  | 14924 | -4.900 | -0.1416 | Yes |
| 157 | INSL5 |  |  | 15099 | -5.700 | -0.1315 | Yes |
| 158 | GAL |  |  | 15193 | -6.100 | -0.1148 | Yes |
| 159 | SSTR1 |  |  | 15216 | -6.300 | -0.0928 | Yes |
| 160 | PYY |  |  | 15309 | -6.800 | -0.0735 | Yes |
| 161 | ADCY5 |  |  | 15313 | -6.800 | -0.0484 | Yes |
| 162 | SST |  |  | 15599 | -10.200 | -0.0287 | Yes |
| 163 | C3 |  |  | 15605 | -10.300 | 0.0092 | Yes |
Table: GSEA details [plain text format]

  

Fig 2: REACTOME\_G\_ALPHA\_I\_SIGNALLING\_EVENTS: Random ES distribution      
 Gene set null distribution of ES for **REACTOME\_G\_ALPHA\_I\_SIGNALLING\_EVENTS**

  
