# Supplementary material for: Immunomic, genomic and transcriptomic characterization of CT26 colorectal carcinoma
Source: BMC Genomics. 2014 Mar 13;15(1):190. doi: 10.1186/1471-2164-15-190 (PMC4007559; doi:10.1186/1471-2164-15-190)
Supplement: Supplementary file 8 — Additional file 8: Contains the Gene Pattern gene set membership and enrichment values in an html format. The file index.html is the entry point. (ZIP 13 MB) [file 12864_2013_7028_MOESM8_ESM.zip › REACTOME_G_ALPHA_Q_SIGNALLING_EVENTS.html]

Details for gene set REACTOME\_G\_ALPHA\_Q\_SIGNALLING\_EVENTS[GSEA]

|  || Dataset | CT26\_gene\_expression |
| Phenotype | NoPhenotypeAvailable |
| Upregulated in class | na\_neg |
| GeneSet | REACTOME\_G\_ALPHA\_Q\_SIGNALLING\_EVENTS |
| Enrichment Score (ES) | -0.31744346 |
| Normalized Enrichment Score (NES) | NaN |
| Nominal p-value | NaN |
| FDR q-value | 1.0 |
| FWER p-Value | 0.0 |
Table: GSEA Results Summary

  

Fig 1: Enrichment plot: REACTOME\_G\_ALPHA\_Q\_SIGNALLING\_EVENTS      
 Profile of the Running ES Score & Positions of GeneSet Members on the Rank Ordered List

  

| PROBE | GENE SYMBOL | GENE\_TITLE | RANK IN GENE LIST | RANK METRIC SCORE | RUNNING ES | CORE ENRICHMENT || 1 | ANXA1 |  |  | 697 | 16.400 | 0.0123 | No |
| 2 | RGS19 |  |  | 2034 | 9.500 | -0.0404 | No |
| 3 | GNG10 |  |  | 2412 | 8.500 | -0.0350 | No |
| 4 | F2R |  |  | 2440 | 8.400 | -0.0075 | No |
| 5 | PIK3CA |  |  | 3066 | 6.800 | -0.0240 | No |
| 6 | GNB1 |  |  | 3457 | 5.800 | -0.0289 | No |
| 7 | PMCH |  |  | 3480 | 5.800 | -0.0101 | No |
| 8 | DGKD |  |  | 3804 | 5.100 | -0.0131 | No |
| 9 | GNG5 |  |  | 3909 | 4.900 | -0.0027 | No |
| 10 | PLCB1 |  |  | 3939 | 4.900 | 0.0125 | No |
| 11 | PLCB4 |  |  | 4208 | 4.400 | 0.0106 | No |
| 12 | GRK5 |  |  | 4213 | 4.400 | 0.0256 | No |
| 13 | GNG12 |  |  | 4271 | 4.300 | 0.0369 | No |
| 14 | GNAQ |  |  | 4482 | 3.900 | 0.0370 | No |
| 15 | ITPR2 |  |  | 4594 | 3.700 | 0.0427 | No |
| 16 | TRIO |  |  | 4740 | 3.500 | 0.0456 | No |
| 17 | PIK3R1 |  |  | 5018 | 3.000 | 0.0383 | No |
| 18 | GNB2 |  |  | 5311 | 2.600 | 0.0286 | No |
| 19 | GNG13 |  |  | 5348 | 2.500 | 0.0349 | No |
| 20 | PRKCE |  |  | 5608 | 2.100 | 0.0256 | No |
| 21 | PIK3R2 |  |  | 5623 | 2.100 | 0.0320 | No |
| 22 | GNG8 |  |  | 5784 | 1.900 | 0.0284 | No |
| 23 | PIK3R3 |  |  | 6055 | 1.500 | 0.0163 | No |
| 24 | GNRH1 |  |  | 6126 | 1.500 | 0.0170 | No |
| 25 | PROKR1 |  |  | 6193 | 1.400 | 0.0176 | No |
| 26 | DGKE |  |  | 6491 | 0.900 | 0.0017 | No |
| 27 | KALRN |  |  | 6766 | 0.600 | -0.0138 | No |
| 28 | NPFF |  |  | 7140 | 0.300 | -0.0367 | No |
| 29 | NTSR2 |  |  | 7274 | 0.200 | -0.0445 | No |
| 30 | OXT |  |  | 7433 | 0.100 | -0.0543 | No |
| 31 | ADRA1D |  |  | 7481 | 0.100 | -0.0570 | No |
| 32 | UTS2 |  |  | 7534 | 0.000 | -0.0603 | No |
| 33 | GHRL |  |  | 7558 | 0.000 | -0.0618 | No |
| 34 | PROK1 |  |  | 7579 | 0.000 | -0.0631 | No |
| 35 | TRHR |  |  | 7758 | 0.000 | -0.0745 | No |
| 36 | NPS |  |  | 7825 | 0.000 | -0.0787 | No |
| 37 | BRS3 |  |  | 8130 | 0.000 | -0.0982 | No |
| 38 | CHRM5 |  |  | 8159 | 0.000 | -0.1000 | No |
| 39 | DGKK |  |  | 8220 | 0.000 | -0.1039 | No |
| 40 | GAST |  |  | 8284 | 0.000 | -0.1079 | No |
| 41 | GHSR |  |  | 8289 | 0.000 | -0.1082 | No |
| 42 | GNGT1 |  |  | 8304 | 0.000 | -0.1091 | No |
| 43 | GNRHR |  |  | 8305 | 0.000 | -0.1091 | No |
| 44 | HTR2C |  |  | 8334 | 0.000 | -0.1109 | No |
| 45 | NMS |  |  | 8485 | 0.000 | -0.1205 | No |
| 46 | NPSR1 |  |  | 8488 | 0.000 | -0.1206 | No |
| 47 | OPN4 |  |  | 8754 | 0.000 | -0.1376 | No |
| 48 | UTS2D |  |  | 8934 | 0.000 | -0.1491 | No |
| 49 | KNG1 |  |  | 8986 | 0.000 | -0.1524 | No |
| 50 | HRH1 |  |  | 8993 | 0.000 | -0.1528 | No |
| 51 | GPRC6A |  |  | 9019 | 0.000 | -0.1544 | No |
| 52 | PROKR2 |  |  | 9145 | 0.000 | -0.1624 | No |
| 53 | NPFFR2 |  |  | 9150 | 0.000 | -0.1627 | No |
| 54 | TRH |  |  | 9182 | 0.000 | -0.1646 | No |
| 55 | TAC3 |  |  | 9300 | 0.000 | -0.1722 | No |
| 56 | HTR2A |  |  | 9366 | 0.000 | -0.1763 | No |
| 57 | UTS2R |  |  | 9367 | 0.000 | -0.1763 | No |
| 58 | HCRTR2 |  |  | 9388 | 0.000 | -0.1776 | No |
| 59 | TRPC6 |  |  | 9389 | 0.000 | -0.1776 | No |
| 60 | NMUR2 |  |  | 9495 | 0.000 | -0.1843 | No |
| 61 | RGS18 |  |  | 9653 | 0.000 | -0.1944 | No |
| 62 | NMBR |  |  | 9686 | 0.000 | -0.1965 | No |
| 63 | GNB3 |  |  | 9821 | 0.000 | -0.2051 | No |
| 64 | CASR |  |  | 9851 | 0.000 | -0.2069 | No |
| 65 | TRPC7 |  |  | 9927 | 0.000 | -0.2117 | No |
| 66 | AVP |  |  | 9946 | 0.000 | -0.2129 | No |
| 67 | DGKI |  |  | 9977 | 0.000 | -0.2148 | No |
| 68 | DGKB |  |  | 10006 | 0.000 | -0.2166 | No |
| 69 | NMB |  |  | 10045 | 0.000 | -0.2191 | No |
| 70 | CCKBR |  |  | 10084 | 0.000 | -0.2215 | No |
| 71 | RASGRP1 |  |  | 10213 | -0.100 | -0.2294 | No |
| 72 | XCR1 |  |  | 10268 | -0.100 | -0.2325 | No |
| 73 | P2RY10 |  |  | 10322 | -0.100 | -0.2355 | No |
| 74 | ADRA1A |  |  | 10328 | -0.100 | -0.2355 | No |
| 75 | PTGFR |  |  | 10339 | -0.100 | -0.2358 | No |
| 76 | TACR3 |  |  | 10461 | -0.100 | -0.2432 | No |
| 77 | CYSLTR1 |  |  | 10497 | -0.100 | -0.2451 | No |
| 78 | CYSLTR2 |  |  | 10507 | -0.100 | -0.2453 | No |
| 79 | GNG3 |  |  | 10566 | -0.100 | -0.2487 | No |
| 80 | GNG7 |  |  | 10645 | -0.100 | -0.2534 | No |
| 81 | HCRT |  |  | 10681 | -0.100 | -0.2553 | No |
| 82 | TRPC3 |  |  | 10759 | -0.100 | -0.2599 | No |
| 83 | GCGR |  |  | 10768 | -0.100 | -0.2600 | No |
| 84 | XCL1 |  |  | 10837 | -0.200 | -0.2637 | No |
| 85 | OXTR |  |  | 10880 | -0.200 | -0.2657 | No |
| 86 | PRKCQ |  |  | 11027 | -0.200 | -0.2744 | No |
| 87 | AVPR1B |  |  | 11056 | -0.200 | -0.2755 | No |
| 88 | ADRA1B |  |  | 11074 | -0.200 | -0.2759 | No |
| 89 | GPR65 |  |  | 11081 | -0.200 | -0.2755 | No |
| 90 | HTR2B |  |  | 11086 | -0.200 | -0.2751 | No |
| 91 | F2RL2 |  |  | 11120 | -0.200 | -0.2765 | No |
| 92 | GRPR |  |  | 11139 | -0.200 | -0.2770 | No |
| 93 | ITPR3 |  |  | 11263 | -0.300 | -0.2838 | No |
| 94 | DGKG |  |  | 11297 | -0.300 | -0.2849 | No |
| 95 | GNGT2 |  |  | 11299 | -0.300 | -0.2839 | No |
| 96 | F2 |  |  | 11305 | -0.300 | -0.2832 | No |
| 97 | TACR1 |  |  | 11308 | -0.300 | -0.2823 | No |
| 98 | NPFFR1 |  |  | 11432 | -0.300 | -0.2891 | No |
| 99 | NTS |  |  | 11525 | -0.400 | -0.2937 | No |
| 100 | GNB4 |  |  | 11555 | -0.400 | -0.2941 | No |
| 101 | BDKRB1 |  |  | 11619 | -0.400 | -0.2968 | No |
| 102 | NTSR1 |  |  | 11651 | -0.400 | -0.2974 | No |
| 103 | FFAR1 |  |  | 11661 | -0.500 | -0.2962 | No |
| 104 | LTB4R |  |  | 11764 | -0.500 | -0.3010 | No |
| 105 | EDN1 |  |  | 11777 | -0.500 | -0.3001 | No |
| 106 | LTB4R2 |  |  | 11943 | -0.600 | -0.3086 | No |
| 107 | GNG4 |  |  | 11993 | -0.600 | -0.3096 | No |
| 108 | MCHR1 |  |  | 12108 | -0.700 | -0.3145 | No |
| 109 | EDNRB |  |  | 12155 | -0.700 | -0.3150 | Yes |
| 110 | RGS2 |  |  | 12156 | -0.700 | -0.3126 | Yes |
| 111 | GNG2 |  |  | 12174 | -0.800 | -0.3109 | Yes |
| 112 | KISS1 |  |  | 12176 | -0.800 | -0.3082 | Yes |
| 113 | PLCB2 |  |  | 12180 | -0.800 | -0.3056 | Yes |
| 114 | EDN3 |  |  | 12211 | -0.800 | -0.3047 | Yes |
| 115 | FFAR3 |  |  | 12216 | -0.800 | -0.3022 | Yes |
| 116 | GNA15 |  |  | 12242 | -0.800 | -0.3010 | Yes |
| 117 | CHRM3 |  |  | 12254 | -0.800 | -0.2989 | Yes |
| 118 | AVPR1A |  |  | 12399 | -0.900 | -0.3051 | Yes |
| 119 | NMUR1 |  |  | 12483 | -1.000 | -0.3069 | Yes |
| 120 | PRKCD |  |  | 12508 | -1.000 | -0.3050 | Yes |
| 121 | P2RY2 |  |  | 12549 | -1.000 | -0.3041 | Yes |
| 122 | GPR68 |  |  | 12582 | -1.000 | -0.3026 | Yes |
| 123 | DGKH |  |  | 12791 | -1.200 | -0.3118 | Yes |
| 124 | GNB5 |  |  | 12793 | -1.200 | -0.3077 | Yes |
| 125 | CCKAR |  |  | 12831 | -1.300 | -0.3055 | Yes |
| 126 | GPR17 |  |  | 12880 | -1.300 | -0.3041 | Yes |
| 127 | HCRTR1 |  |  | 12888 | -1.300 | -0.3000 | Yes |
| 128 | GRP |  |  | 12978 | -1.400 | -0.3009 | Yes |
| 129 | GPR132 |  |  | 12986 | -1.400 | -0.2965 | Yes |
| 130 | GPR4 |  |  | 13015 | -1.500 | -0.2930 | Yes |
| 131 | PRKCH |  |  | 13040 | -1.500 | -0.2894 | Yes |
| 132 | APP |  |  | 13046 | -1.500 | -0.2845 | Yes |
| 133 | NMU |  |  | 13073 | -1.500 | -0.2809 | Yes |
| 134 | AGTR1 |  |  | 13128 | -1.600 | -0.2788 | Yes |
| 135 | EDNRA |  |  | 13135 | -1.600 | -0.2736 | Yes |
| 136 | KISS1R |  |  | 13212 | -1.700 | -0.2726 | Yes |
| 137 | CHRM1 |  |  | 13644 | -2.200 | -0.2926 | Yes |
| 138 | DAGLA |  |  | 13668 | -2.300 | -0.2861 | Yes |
| 139 | CCK |  |  | 13672 | -2.300 | -0.2783 | Yes |
| 140 | PTAFR |  |  | 13676 | -2.300 | -0.2705 | Yes |
| 141 | F2RL3 |  |  | 13786 | -2.400 | -0.2691 | Yes |
| 142 | BDKRB2 |  |  | 13805 | -2.500 | -0.2616 | Yes |
| 143 | RASGRP2 |  |  | 13817 | -2.500 | -0.2536 | Yes |
| 144 | DAGLB |  |  | 13895 | -2.600 | -0.2495 | Yes |
| 145 | TAC1 |  |  | 13930 | -2.700 | -0.2423 | Yes |
| 146 | GNG11 |  |  | 14019 | -2.800 | -0.2382 | Yes |
| 147 | GNA14 |  |  | 14050 | -2.900 | -0.2301 | Yes |
| 148 | P2RY1 |  |  | 14216 | -3.100 | -0.2299 | Yes |
| 149 | DGKQ |  |  | 14628 | -4.200 | -0.2416 | Yes |
| 150 | P2RY6 |  |  | 14706 | -4.300 | -0.2316 | Yes |
| 151 | ADRBK1 |  |  | 14708 | -4.400 | -0.2164 | Yes |
| 152 | MGLL |  |  | 14742 | -4.400 | -0.2032 | Yes |
| 153 | SAA1 |  |  | 14809 | -4.600 | -0.1914 | Yes |
| 154 | TBXA2R |  |  | 14827 | -4.700 | -0.1762 | Yes |
| 155 | FFAR2 |  |  | 14833 | -4.700 | -0.1602 | Yes |
| 156 | AGT |  |  | 14844 | -4.700 | -0.1445 | Yes |
| 157 | TACR2 |  |  | 14856 | -4.700 | -0.1288 | Yes |
| 158 | GNA11 |  |  | 15004 | -5.200 | -0.1202 | Yes |
| 159 | EDN2 |  |  | 15252 | -6.400 | -0.1138 | Yes |
| 160 | PTGER1 |  |  | 15335 | -7.000 | -0.0947 | Yes |
| 161 | GCG |  |  | 15351 | -7.100 | -0.0710 | Yes |
| 162 | PLCB3 |  |  | 15425 | -7.700 | -0.0489 | Yes |
| 163 | F2RL1 |  |  | 15456 | -8.000 | -0.0230 | Yes |
| 164 | DGKA |  |  | 15656 | -12.000 | 0.0060 | Yes |
Table: GSEA details [plain text format]

  

Fig 2: REACTOME\_G\_ALPHA\_Q\_SIGNALLING\_EVENTS: Random ES distribution      
 Gene set null distribution of ES for **REACTOME\_G\_ALPHA\_Q\_SIGNALLING\_EVENTS**

  
