# Supplementary material for: Immunomic, genomic and transcriptomic characterization of CT26 colorectal carcinoma
Source: BMC Genomics. 2014 Mar 13;15(1):190. doi: 10.1186/1471-2164-15-190 (PMC4007559; doi:10.1186/1471-2164-15-190)
Supplement: Supplementary file 8 — Additional file 8: Contains the Gene Pattern gene set membership and enrichment values in an html format. The file index.html is the entry point. (ZIP 13 MB) [file 12864_2013_7028_MOESM8_ESM.zip › REACTOME_G_ALPHA_S_SIGNALLING_EVENTS.html]

Details for gene set REACTOME\_G\_ALPHA\_S\_SIGNALLING\_EVENTS[GSEA]

|  || Dataset | CT26\_gene\_expression |
| Phenotype | NoPhenotypeAvailable |
| Upregulated in class | na\_neg |
| GeneSet | REACTOME\_G\_ALPHA\_S\_SIGNALLING\_EVENTS |
| Enrichment Score (ES) | -0.4090771 |
| Normalized Enrichment Score (NES) | NaN |
| Nominal p-value | NaN |
| FDR q-value | 1.0 |
| FWER p-Value | 0.0 |
Table: GSEA Results Summary

  

Fig 1: Enrichment plot: REACTOME\_G\_ALPHA\_S\_SIGNALLING\_EVENTS      
 Profile of the Running ES Score & Positions of GeneSet Members on the Rank Ordered List

  

| PROBE | GENE SYMBOL | GENE\_TITLE | RANK IN GENE LIST | RANK METRIC SCORE | RUNNING ES | CORE ENRICHMENT || 1 | GNAI3 |  |  | 884 | 14.900 | 0.0319 | No |
| 2 | GNG10 |  |  | 2412 | 8.500 | -0.0153 | No |
| 3 | GNB1 |  |  | 3457 | 5.800 | -0.0477 | No |
| 4 | GNG5 |  |  | 3909 | 4.900 | -0.0475 | No |
| 5 | GNG12 |  |  | 4271 | 4.300 | -0.0450 | No |
| 6 | GNB2 |  |  | 5311 | 2.600 | -0.0961 | No |
| 7 | GNG13 |  |  | 5348 | 2.500 | -0.0835 | No |
| 8 | GNAS |  |  | 5595 | 2.200 | -0.0862 | No |
| 9 | ADCY7 |  |  | 5626 | 2.100 | -0.0757 | No |
| 10 | GNG8 |  |  | 5784 | 1.900 | -0.0744 | No |
| 11 | PDE7A |  |  | 5796 | 1.900 | -0.0639 | No |
| 12 | ADM2 |  |  | 6640 | 0.800 | -0.1130 | No |
| 13 | PTHLH |  |  | 6699 | 0.700 | -0.1126 | No |
| 14 | CALCRL |  |  | 6853 | 0.500 | -0.1194 | No |
| 15 | PDE3B |  |  | 7100 | 0.300 | -0.1334 | No |
| 16 | LHB |  |  | 7411 | 0.100 | -0.1526 | No |
| 17 | MC3R |  |  | 7564 | 0.000 | -0.1623 | No |
| 18 | RXFP2 |  |  | 7821 | 0.000 | -0.1787 | No |
| 19 | NPS |  |  | 7825 | 0.000 | -0.1789 | No |
| 20 | PTGDR |  |  | 7881 | 0.000 | -0.1824 | No |
| 21 | MC1R |  |  | 8028 | 0.000 | -0.1917 | No |
| 22 | CGA |  |  | 8158 | 0.000 | -0.2000 | No |
| 23 | CRH |  |  | 8178 | 0.000 | -0.2012 | No |
| 24 | DRD5 |  |  | 8229 | 0.000 | -0.2044 | No |
| 25 | FSHB |  |  | 8272 | 0.000 | -0.2071 | No |
| 26 | FSHR |  |  | 8273 | 0.000 | -0.2071 | No |
| 27 | GNGT1 |  |  | 8304 | 0.000 | -0.2090 | No |
| 28 | NPSR1 |  |  | 8488 | 0.000 | -0.2207 | No |
| 29 | PTH |  |  | 8795 | 0.000 | -0.2403 | No |
| 30 | RLN2 |  |  | 8804 | 0.000 | -0.2408 | No |
| 31 | TAAR1 |  |  | 8872 | 0.000 | -0.2451 | No |
| 32 | TAAR2 |  |  | 8873 | 0.000 | -0.2451 | No |
| 33 | TAAR5 |  |  | 8875 | 0.000 | -0.2452 | No |
| 34 | TAAR6 |  |  | 8876 | 0.000 | -0.2452 | No |
| 35 | TAAR8 |  |  | 8877 | 0.000 | -0.2452 | No |
| 36 | PDE4B |  |  | 8900 | 0.000 | -0.2466 | No |
| 37 | LHCGR |  |  | 9014 | 0.000 | -0.2538 | No |
| 38 | RXFP1 |  |  | 9086 | 0.000 | -0.2583 | No |
| 39 | GIP |  |  | 9204 | 0.000 | -0.2658 | No |
| 40 | DRD1 |  |  | 9397 | 0.000 | -0.2781 | No |
| 41 | TSHB |  |  | 9452 | 0.000 | -0.2815 | No |
| 42 | CALCA |  |  | 9487 | 0.000 | -0.2837 | No |
| 43 | MC4R |  |  | 9554 | 0.000 | -0.2879 | No |
| 44 | ADCY8 |  |  | 9623 | 0.000 | -0.2923 | No |
| 45 | MC5R |  |  | 9630 | 0.000 | -0.2927 | No |
| 46 | HTR7 |  |  | 9637 | 0.000 | -0.2931 | No |
| 47 | MC2R |  |  | 9747 | 0.000 | -0.3000 | No |
| 48 | GNB3 |  |  | 9821 | 0.000 | -0.3047 | No |
| 49 | GHRHR |  |  | 9841 | 0.000 | -0.3059 | No |
| 50 | AVP |  |  | 9946 | 0.000 | -0.3126 | No |
| 51 | GHRH |  |  | 10029 | 0.000 | -0.3178 | No |
| 52 | RLN3 |  |  | 10099 | 0.000 | -0.3222 | No |
| 53 | PDE11A |  |  | 10245 | -0.100 | -0.3309 | No |
| 54 | AVPR2 |  |  | 10377 | -0.100 | -0.3387 | No |
| 55 | ADCYAP1 |  |  | 10435 | -0.100 | -0.3417 | No |
| 56 | CRHR2 |  |  | 10452 | -0.100 | -0.3422 | No |
| 57 | HTR6 |  |  | 10490 | -0.100 | -0.3439 | No |
| 58 | HRH2 |  |  | 10527 | -0.100 | -0.3457 | No |
| 59 | GNG3 |  |  | 10566 | -0.100 | -0.3475 | No |
| 60 | SCTR |  |  | 10620 | -0.100 | -0.3503 | No |
| 61 | GNG7 |  |  | 10645 | -0.100 | -0.3512 | No |
| 62 | GLP1R |  |  | 10729 | -0.100 | -0.3559 | No |
| 63 | GCGR |  |  | 10768 | -0.100 | -0.3578 | No |
| 64 | TSHR |  |  | 10809 | -0.200 | -0.3592 | No |
| 65 | ADCY1 |  |  | 10858 | -0.200 | -0.3610 | No |
| 66 | POMC |  |  | 10894 | -0.200 | -0.3621 | No |
| 67 | PDE10A |  |  | 10979 | -0.200 | -0.3663 | No |
| 68 | PDE1A |  |  | 11191 | -0.300 | -0.3780 | No |
| 69 | GNGT2 |  |  | 11299 | -0.300 | -0.3831 | No |
| 70 | ADORA2B |  |  | 11483 | -0.400 | -0.3924 | No |
| 71 | PDE8B |  |  | 11537 | -0.400 | -0.3934 | No |
| 72 | PDE7B |  |  | 11544 | -0.400 | -0.3914 | No |
| 73 | GNB4 |  |  | 11555 | -0.400 | -0.3897 | No |
| 74 | ADCY2 |  |  | 11597 | -0.400 | -0.3899 | No |
| 75 | GIPR |  |  | 11698 | -0.500 | -0.3934 | No |
| 76 | IAPP |  |  | 11750 | -0.500 | -0.3937 | No |
| 77 | ADRB2 |  |  | 11843 | -0.600 | -0.3960 | No |
| 78 | CALCB |  |  | 11861 | -0.600 | -0.3935 | No |
| 79 | GLP2R |  |  | 11954 | -0.600 | -0.3958 | No |
| 80 | GNG4 |  |  | 11993 | -0.600 | -0.3947 | No |
| 81 | GNAI2 |  |  | 12163 | -0.700 | -0.4014 | No |
| 82 | GNG2 |  |  | 12174 | -0.800 | -0.3972 | No |
| 83 | PTGER2 |  |  | 12243 | -0.800 | -0.3968 | No |
| 84 | VIPR2 |  |  | 12317 | -0.900 | -0.3962 | No |
| 85 | PTGIR |  |  | 12396 | -0.900 | -0.3958 | No |
| 86 | PDE4D |  |  | 12503 | -1.000 | -0.3967 | No |
| 87 | RAMP3 |  |  | 12698 | -1.100 | -0.4025 | Yes |
| 88 | ADORA2A |  |  | 12713 | -1.200 | -0.3963 | Yes |
| 89 | GNB5 |  |  | 12793 | -1.200 | -0.3943 | Yes |
| 90 | ADCY4 |  |  | 12802 | -1.200 | -0.3876 | Yes |
| 91 | GNAZ |  |  | 12843 | -1.300 | -0.3825 | Yes |
| 92 | PDE4C |  |  | 12852 | -1.300 | -0.3753 | Yes |
| 93 | ADCY3 |  |  | 12965 | -1.400 | -0.3741 | Yes |
| 94 | HTR4 |  |  | 12993 | -1.400 | -0.3676 | Yes |
| 95 | ADCYAP1R1 |  |  | 13187 | -1.700 | -0.3698 | Yes |
| 96 | PDE8A |  |  | 13299 | -1.800 | -0.3662 | Yes |
| 97 | GPBAR1 |  |  | 13500 | -2.000 | -0.3672 | Yes |
| 98 | GNAI1 |  |  | 13615 | -2.200 | -0.3614 | Yes |
| 99 | ADCY9 |  |  | 13683 | -2.300 | -0.3520 | Yes |
| 100 | ADRB1 |  |  | 13803 | -2.500 | -0.3448 | Yes |
| 101 | INSL3 |  |  | 13939 | -2.700 | -0.3374 | Yes |
| 102 | GNG11 |  |  | 14019 | -2.800 | -0.3258 | Yes |
| 103 | ADM |  |  | 14162 | -3.000 | -0.3171 | Yes |
| 104 | SCT |  |  | 14335 | -3.400 | -0.3079 | Yes |
| 105 | RAMP2 |  |  | 14395 | -3.600 | -0.2904 | Yes |
| 106 | VIP |  |  | 14431 | -3.700 | -0.2706 | Yes |
| 107 | PTGER4 |  |  | 14532 | -3.900 | -0.2539 | Yes |
| 108 | PDE1B |  |  | 14541 | -3.900 | -0.2313 | Yes |
| 109 | PDE3A |  |  | 14637 | -4.200 | -0.2124 | Yes |
| 110 | ADRB3 |  |  | 14670 | -4.300 | -0.1889 | Yes |
| 111 | ADCY6 |  |  | 14830 | -4.700 | -0.1712 | Yes |
| 112 | ADCY5 |  |  | 15313 | -6.800 | -0.1617 | Yes |
| 113 | GCG |  |  | 15351 | -7.100 | -0.1219 | Yes |
| 114 | RAMP1 |  |  | 15374 | -7.300 | -0.0800 | Yes |
| 115 | PDE2A |  |  | 15480 | -8.300 | -0.0375 | Yes |
| 116 | VIPR1 |  |  | 15540 | -9.200 | 0.0134 | Yes |
Table: GSEA details [plain text format]

  

Fig 2: REACTOME\_G\_ALPHA\_S\_SIGNALLING\_EVENTS: Random ES distribution      
 Gene set null distribution of ES for **REACTOME\_G\_ALPHA\_S\_SIGNALLING\_EVENTS**

  
