# Supplementary material for: Immunomic, genomic and transcriptomic characterization of CT26 colorectal carcinoma
Source: BMC Genomics. 2014 Mar 13;15(1):190. doi: 10.1186/1471-2164-15-190 (PMC4007559; doi:10.1186/1471-2164-15-190)
Supplement: Supplementary file 8 — Additional file 8: Contains the Gene Pattern gene set membership and enrichment values in an html format. The file index.html is the entry point. (ZIP 13 MB) [file 12864_2013_7028_MOESM8_ESM.zip › REACTOME_MITOTIC_M_M_G1_PHASES.html]

Details for gene set REACTOME\_MITOTIC\_M\_M\_G1\_PHASES[GSEA]

|  || Dataset | CT26\_gene\_expression |
| Phenotype | NoPhenotypeAvailable |
| Upregulated in class | na\_pos |
| GeneSet | REACTOME\_MITOTIC\_M\_M\_G1\_PHASES |
| Enrichment Score (ES) | 0.7633832 |
| Normalized Enrichment Score (NES) | 1.6896632 |
| Nominal p-value | 0.0 |
| FDR q-value | 0.0019191848 |
| FWER p-Value | 0.032 |
Table: GSEA Results Summary

  

Fig 1: Enrichment plot: REACTOME\_MITOTIC\_M\_M\_G1\_PHASES      
 Profile of the Running ES Score & Positions of GeneSet Members on the Rank Ordered List

  

| PROBE | GENE SYMBOL | GENE\_TITLE | RANK IN GENE LIST | RANK METRIC SCORE | RUNNING ES | CORE ENRICHMENT || 1 | PSMD12 |  |  | 27 | 39.500 | 0.0182 | Yes |
| 2 | PSMD1 |  |  | 41 | 37.400 | 0.0363 | Yes |
| 3 | KIF20A |  |  | 77 | 31.900 | 0.0502 | Yes |
| 4 | MCM4 |  |  | 81 | 31.500 | 0.0659 | Yes |
| 5 | RAD21 |  |  | 86 | 31.400 | 0.0815 | Yes |
| 6 | RCC2 |  |  | 99 | 30.500 | 0.0961 | Yes |
| 7 | PRIM1 |  |  | 116 | 29.000 | 0.1098 | Yes |
| 8 | RPA1 |  |  | 120 | 28.700 | 0.1241 | Yes |
| 9 | XPO1 |  |  | 148 | 27.000 | 0.1360 | Yes |
| 10 | MCM6 |  |  | 163 | 26.600 | 0.1485 | Yes |
| 11 | PSMC2 |  |  | 173 | 26.000 | 0.1611 | Yes |
| 12 | AHCTF1 |  |  | 189 | 24.800 | 0.1727 | Yes |
| 13 | CKAP5 |  |  | 210 | 24.300 | 0.1836 | Yes |
| 14 | RANGAP1 |  |  | 220 | 24.000 | 0.1952 | Yes |
| 15 | PSMD2 |  |  | 227 | 23.700 | 0.2068 | Yes |
| 16 | PSMA3 |  |  | 245 | 23.200 | 0.2174 | Yes |
| 17 | PSMC4 |  |  | 253 | 22.900 | 0.2285 | Yes |
| 18 | INCENP |  |  | 260 | 22.700 | 0.2396 | Yes |
| 19 | SMC3 |  |  | 265 | 22.600 | 0.2508 | Yes |
| 20 | CDC20 |  |  | 284 | 22.200 | 0.2608 | Yes |
| 21 | MCM7 |  |  | 290 | 21.900 | 0.2716 | Yes |
| 22 | MCM3 |  |  | 315 | 21.400 | 0.2809 | Yes |
| 23 | KIF23 |  |  | 337 | 21.000 | 0.2901 | Yes |
| 24 | BUB1 |  |  | 360 | 20.600 | 0.2991 | Yes |
| 25 | DBF4 |  |  | 369 | 20.400 | 0.3089 | Yes |
| 26 | PSMA5 |  |  | 381 | 20.200 | 0.3184 | Yes |
| 27 | NUP107 |  |  | 399 | 19.900 | 0.3274 | Yes |
| 28 | CENPN |  |  | 403 | 19.900 | 0.3372 | Yes |
| 29 | STAG2 |  |  | 456 | 19.100 | 0.3436 | Yes |
| 30 | PSMA4 |  |  | 484 | 18.600 | 0.3512 | Yes |
| 31 | PSMC1 |  |  | 493 | 18.600 | 0.3601 | Yes |
| 32 | BIRC5 |  |  | 507 | 18.400 | 0.3686 | Yes |
| 33 | BUB3 |  |  | 533 | 18.100 | 0.3761 | Yes |
| 34 | MAD2L1 |  |  | 559 | 17.900 | 0.3836 | Yes |
| 35 | ZWINT |  |  | 574 | 17.700 | 0.3916 | Yes |
| 36 | PSMC6 |  |  | 579 | 17.600 | 0.4002 | Yes |
| 37 | RANBP2 |  |  | 605 | 17.300 | 0.4074 | Yes |
| 38 | PPP2R5D |  |  | 607 | 17.300 | 0.4161 | Yes |
| 39 | BUB1B |  |  | 609 | 17.300 | 0.4247 | Yes |
| 40 | PAFAH1B1 |  |  | 614 | 17.300 | 0.4332 | Yes |
| 41 | ZWILCH |  |  | 620 | 17.200 | 0.4416 | Yes |
| 42 | CENPQ |  |  | 635 | 17.000 | 0.4493 | Yes |
| 43 | RPS27A |  |  | 668 | 16.700 | 0.4557 | Yes |
| 44 | FBXO5 |  |  | 688 | 16.500 | 0.4628 | Yes |
| 45 | KIF2A |  |  | 719 | 16.200 | 0.4690 | Yes |
| 46 | PSMA1 |  |  | 724 | 16.200 | 0.4770 | Yes |
| 47 | SGOL1 |  |  | 857 | 15.100 | 0.4761 | Yes |
| 48 | PSMD14 |  |  | 859 | 15.100 | 0.4837 | Yes |
| 49 | NUF2 |  |  | 866 | 15.100 | 0.4909 | Yes |
| 50 | NDC80 |  |  | 878 | 15.000 | 0.4978 | Yes |
| 51 | PSMB3 |  |  | 881 | 15.000 | 0.5053 | Yes |
| 52 | PSME1 |  |  | 893 | 14.900 | 0.5121 | Yes |
| 53 | GMNN |  |  | 911 | 14.800 | 0.5185 | Yes |
| 54 | SGOL2 |  |  | 1015 | 14.100 | 0.5190 | Yes |
| 55 | CDCA8 |  |  | 1017 | 14.100 | 0.5261 | Yes |
| 56 | KIF2C |  |  | 1036 | 14.000 | 0.5320 | Yes |
| 57 | SMC1A |  |  | 1064 | 13.800 | 0.5372 | Yes |
| 58 | NUP85 |  |  | 1073 | 13.800 | 0.5437 | Yes |
| 59 | MIS12 |  |  | 1095 | 13.600 | 0.5492 | Yes |
| 60 | PSMC5 |  |  | 1103 | 13.600 | 0.5556 | Yes |
| 61 | E2F1 |  |  | 1172 | 13.100 | 0.5579 | Yes |
| 62 | PSMD11 |  |  | 1192 | 13.100 | 0.5633 | Yes |
| 63 | RPA2 |  |  | 1205 | 13.000 | 0.5691 | Yes |
| 64 | POLA1 |  |  | 1250 | 12.800 | 0.5727 | Yes |
| 65 | CENPH |  |  | 1257 | 12.800 | 0.5788 | Yes |
| 66 | NUP37 |  |  | 1258 | 12.800 | 0.5853 | Yes |
| 67 | CENPI |  |  | 1262 | 12.700 | 0.5915 | Yes |
| 68 | MCM10 |  |  | 1264 | 12.700 | 0.5979 | Yes |
| 69 | MCM2 |  |  | 1281 | 12.700 | 0.6033 | Yes |
| 70 | PSMB7 |  |  | 1288 | 12.700 | 0.6093 | Yes |
| 71 | SPC25 |  |  | 1296 | 12.600 | 0.6152 | Yes |
| 72 | PSMA7 |  |  | 1308 | 12.500 | 0.6208 | Yes |
| 73 | PPP2R5C |  |  | 1333 | 12.400 | 0.6255 | Yes |
| 74 | PSMD7 |  |  | 1346 | 12.300 | 0.6310 | Yes |
| 75 | PSME4 |  |  | 1361 | 12.300 | 0.6363 | Yes |
| 76 | PSMD6 |  |  | 1380 | 12.200 | 0.6413 | Yes |
| 77 | MAPRE1 |  |  | 1391 | 12.100 | 0.6468 | Yes |
| 78 | PSMD10 |  |  | 1417 | 12.000 | 0.6512 | Yes |
| 79 | KIF18A |  |  | 1462 | 11.800 | 0.6544 | Yes |
| 80 | MCM5 |  |  | 1526 | 11.500 | 0.6562 | Yes |
| 81 | PSMB1 |  |  | 1532 | 11.500 | 0.6616 | Yes |
| 82 | CDT1 |  |  | 1580 | 11.300 | 0.6643 | Yes |
| 83 | RPA3 |  |  | 1621 | 11.200 | 0.6674 | Yes |
| 84 | SPC24 |  |  | 1627 | 11.200 | 0.6728 | Yes |
| 85 | NUP133 |  |  | 1634 | 11.100 | 0.6780 | Yes |
| 86 | SEH1L |  |  | 1666 | 10.900 | 0.6815 | Yes |
| 87 | CENPA |  |  | 1762 | 10.600 | 0.6808 | Yes |
| 88 | PSMD5 |  |  | 1765 | 10.600 | 0.6860 | Yes |
| 89 | PSME2 |  |  | 1904 | 10.000 | 0.6822 | Yes |
| 90 | CASC5 |  |  | 1912 | 10.000 | 0.6868 | Yes |
| 91 | CENPC1 |  |  | 1914 | 10.000 | 0.6918 | Yes |
| 92 | PSMB2 |  |  | 1917 | 10.000 | 0.6967 | Yes |
| 93 | DSN1 |  |  | 1919 | 10.000 | 0.7017 | Yes |
| 94 | PSMA2 |  |  | 1980 | 9.700 | 0.7028 | Yes |
| 95 | PPP2R1B |  |  | 2048 | 9.500 | 0.7033 | Yes |
| 96 | STAG1 |  |  | 2077 | 9.400 | 0.7062 | Yes |
| 97 | POLA2 |  |  | 2142 | 9.200 | 0.7068 | Yes |
| 98 | PPP2CB |  |  | 2170 | 9.100 | 0.7096 | Yes |
| 99 | CLIP1 |  |  | 2185 | 9.100 | 0.7133 | Yes |
| 100 | PSMA6 |  |  | 2205 | 9.000 | 0.7167 | Yes |
| 101 | CENPT |  |  | 2232 | 9.000 | 0.7195 | Yes |
| 102 | PSMC3 |  |  | 2237 | 9.000 | 0.7238 | Yes |
| 103 | POLE |  |  | 2239 | 8.900 | 0.7283 | Yes |
| 104 | KNTC1 |  |  | 2256 | 8.900 | 0.7317 | Yes |
| 105 | CENPL |  |  | 2261 | 8.900 | 0.7360 | Yes |
| 106 | CDC7 |  |  | 2273 | 8.900 | 0.7398 | Yes |
| 107 | POLE2 |  |  | 2300 | 8.800 | 0.7425 | Yes |
| 108 | APITD1 |  |  | 2304 | 8.800 | 0.7468 | Yes |
| 109 | PMF1 |  |  | 2386 | 8.500 | 0.7459 | Yes |
| 110 | CENPM |  |  | 2477 | 8.300 | 0.7443 | Yes |
| 111 | RPS27 |  |  | 2503 | 8.200 | 0.7469 | Yes |
| 112 | PSMD8 |  |  | 2504 | 8.200 | 0.7510 | Yes |
| 113 | PSMD9 |  |  | 2508 | 8.200 | 0.7550 | Yes |
| 114 | TAOK1 |  |  | 2518 | 8.100 | 0.7585 | Yes |
| 115 | CDK2 |  |  | 2519 | 8.100 | 0.7626 | Yes |
| 116 | PSMD4 |  |  | 2609 | 7.900 | 0.7609 | Yes |
| 117 | CCDC99 |  |  | 2632 | 7.800 | 0.7634 | Yes |
| 118 | AURKB |  |  | 2822 | 7.300 | 0.7550 | No |
| 119 | NSL1 |  |  | 2916 | 7.100 | 0.7526 | No |
| 120 | MLF1IP |  |  | 2974 | 7.000 | 0.7525 | No |
| 121 | PSMD13 |  |  | 2986 | 6.900 | 0.7552 | No |
| 122 | ZW10 |  |  | 3019 | 6.900 | 0.7567 | No |
| 123 | CENPP |  |  | 3065 | 6.800 | 0.7572 | No |
| 124 | NUDC |  |  | 3111 | 6.600 | 0.7577 | No |
| 125 | E2F3 |  |  | 3189 | 6.500 | 0.7560 | No |
| 126 | PSMB4 |  |  | 3191 | 6.500 | 0.7592 | No |
| 127 | NUP43 |  |  | 3343 | 6.100 | 0.7526 | No |
| 128 | NDEL1 |  |  | 3503 | 5.800 | 0.7454 | No |
| 129 | MCM8 |  |  | 3515 | 5.700 | 0.7475 | No |
| 130 | MAD1L1 |  |  | 3575 | 5.600 | 0.7466 | No |
| 131 | CLASP1 |  |  | 3634 | 5.500 | 0.7457 | No |
| 132 | CDC6 |  |  | 3705 | 5.300 | 0.7438 | No |
| 133 | PPP2R5E |  |  | 3781 | 5.200 | 0.7417 | No |
| 134 | CENPK |  |  | 4002 | 4.800 | 0.7300 | No |
| 135 | PPP1CC |  |  | 4111 | 4.600 | 0.7254 | No |
| 136 | PPP2R1A |  |  | 4124 | 4.600 | 0.7269 | No |
| 137 | PSMB5 |  |  | 4221 | 4.400 | 0.7230 | No |
| 138 | SEC13 |  |  | 4498 | 3.900 | 0.7073 | No |
| 139 | PSMF1 |  |  | 5043 | 3.000 | 0.6739 | No |
| 140 | UBA52 |  |  | 5629 | 2.100 | 0.6375 | No |
| 141 | PSMD3 |  |  | 5741 | 1.900 | 0.6313 | No |
| 142 | CENPO |  |  | 5968 | 1.600 | 0.6176 | No |
| 143 | ITGB3BP |  |  | 6480 | 0.900 | 0.5853 | No |
| 144 | PLK1 |  |  | 6602 | 0.800 | 0.5779 | No |
| 145 | PPP2CA |  |  | 6791 | 0.600 | 0.5662 | No |
| 146 | PPP2R5A |  |  | 6841 | 0.600 | 0.5634 | No |
| 147 | E2F2 |  |  | 7036 | 0.400 | 0.5511 | No |
| 148 | KIF2B |  |  | 8366 | 0.000 | 0.4659 | No |
| 149 | PSMA8 |  |  | 9516 | 0.000 | 0.3922 | No |
| 150 | PSMB6 |  |  | 9945 | 0.000 | 0.3648 | No |
| 151 | PSMB8 |  |  | 11241 | -0.300 | 0.2819 | No |
| 152 | PSMB10 |  |  | 13961 | -2.700 | 0.1089 | No |
| 153 | PPP2R5B |  |  | 14239 | -3.200 | 0.0928 | No |
| 154 | PSMB9 |  |  | 14266 | -3.300 | 0.0928 | No |
| 155 | GORASP1 |  |  | 14777 | -4.500 | 0.0623 | No |
Table: GSEA details [plain text format]

  

Fig 2: REACTOME\_MITOTIC\_M\_M\_G1\_PHASES: Random ES distribution      
 Gene set null distribution of ES for **REACTOME\_MITOTIC\_M\_M\_G1\_PHASES**

  
