# Supplementary material for: Immunomic, genomic and transcriptomic characterization of CT26 colorectal carcinoma
Source: BMC Genomics. 2014 Mar 13;15(1):190. doi: 10.1186/1471-2164-15-190 (PMC4007559; doi:10.1186/1471-2164-15-190)
Supplement: Supplementary file 8 — Additional file 8: Contains the Gene Pattern gene set membership and enrichment values in an html format. The file index.html is the entry point. (ZIP 13 MB) [file 12864_2013_7028_MOESM8_ESM.zip › REACTOME_M_G1_TRANSITION.html]

Details for gene set REACTOME\_M\_G1\_TRANSITION[GSEA]

|  || Dataset | CT26\_gene\_expression |
| Phenotype | NoPhenotypeAvailable |
| Upregulated in class | na\_pos |
| GeneSet | REACTOME\_M\_G1\_TRANSITION |
| Enrichment Score (ES) | 0.78291017 |
| Normalized Enrichment Score (NES) | 1.7107478 |
| Nominal p-value | 0.0 |
| FDR q-value | 0.0016000408 |
| FWER p-Value | 0.016 |
Table: GSEA Results Summary

  

Fig 1: Enrichment plot: REACTOME\_M\_G1\_TRANSITION      
 Profile of the Running ES Score & Positions of GeneSet Members on the Rank Ordered List

  

| PROBE | GENE SYMBOL | GENE\_TITLE | RANK IN GENE LIST | RANK METRIC SCORE | RUNNING ES | CORE ENRICHMENT || 1 | PSMD12 |  |  | 27 | 39.500 | 0.0419 | Yes |
| 2 | PSMD1 |  |  | 41 | 37.400 | 0.0823 | Yes |
| 3 | MCM4 |  |  | 81 | 31.500 | 0.1146 | Yes |
| 4 | PRIM1 |  |  | 116 | 29.000 | 0.1445 | Yes |
| 5 | RPA1 |  |  | 120 | 28.700 | 0.1760 | Yes |
| 6 | MCM6 |  |  | 163 | 26.600 | 0.2026 | Yes |
| 7 | PSMC2 |  |  | 173 | 26.000 | 0.2308 | Yes |
| 8 | PSMD2 |  |  | 227 | 23.700 | 0.2535 | Yes |
| 9 | PSMA3 |  |  | 245 | 23.200 | 0.2781 | Yes |
| 10 | PSMC4 |  |  | 253 | 22.900 | 0.3029 | Yes |
| 11 | MCM7 |  |  | 290 | 21.900 | 0.3248 | Yes |
| 12 | MCM3 |  |  | 315 | 21.400 | 0.3469 | Yes |
| 13 | DBF4 |  |  | 369 | 20.400 | 0.3660 | Yes |
| 14 | PSMA5 |  |  | 381 | 20.200 | 0.3876 | Yes |
| 15 | PSMA4 |  |  | 484 | 18.600 | 0.4016 | Yes |
| 16 | PSMC1 |  |  | 493 | 18.600 | 0.4217 | Yes |
| 17 | PSMC6 |  |  | 579 | 17.600 | 0.4357 | Yes |
| 18 | RPS27A |  |  | 668 | 16.700 | 0.4485 | Yes |
| 19 | PSMA1 |  |  | 724 | 16.200 | 0.4629 | Yes |
| 20 | PSMD14 |  |  | 859 | 15.100 | 0.4710 | Yes |
| 21 | PSMB3 |  |  | 881 | 15.000 | 0.4862 | Yes |
| 22 | PSME1 |  |  | 893 | 14.900 | 0.5020 | Yes |
| 23 | GMNN |  |  | 911 | 14.800 | 0.5172 | Yes |
| 24 | PSMC5 |  |  | 1103 | 13.600 | 0.5200 | Yes |
| 25 | E2F1 |  |  | 1172 | 13.100 | 0.5302 | Yes |
| 26 | PSMD11 |  |  | 1192 | 13.100 | 0.5434 | Yes |
| 27 | RPA2 |  |  | 1205 | 13.000 | 0.5570 | Yes |
| 28 | POLA1 |  |  | 1250 | 12.800 | 0.5683 | Yes |
| 29 | MCM10 |  |  | 1264 | 12.700 | 0.5815 | Yes |
| 30 | MCM2 |  |  | 1281 | 12.700 | 0.5945 | Yes |
| 31 | PSMB7 |  |  | 1288 | 12.700 | 0.6082 | Yes |
| 32 | PSMA7 |  |  | 1308 | 12.500 | 0.6207 | Yes |
| 33 | PSMD7 |  |  | 1346 | 12.300 | 0.6320 | Yes |
| 34 | PSME4 |  |  | 1361 | 12.300 | 0.6446 | Yes |
| 35 | PSMD6 |  |  | 1380 | 12.200 | 0.6570 | Yes |
| 36 | PSMD10 |  |  | 1417 | 12.000 | 0.6679 | Yes |
| 37 | MCM5 |  |  | 1526 | 11.500 | 0.6737 | Yes |
| 38 | PSMB1 |  |  | 1532 | 11.500 | 0.6861 | Yes |
| 39 | CDT1 |  |  | 1580 | 11.300 | 0.6956 | Yes |
| 40 | RPA3 |  |  | 1621 | 11.200 | 0.7054 | Yes |
| 41 | PSMD5 |  |  | 1765 | 10.600 | 0.7080 | Yes |
| 42 | PSME2 |  |  | 1904 | 10.000 | 0.7102 | Yes |
| 43 | PSMB2 |  |  | 1917 | 10.000 | 0.7205 | Yes |
| 44 | PSMA2 |  |  | 1980 | 9.700 | 0.7272 | Yes |
| 45 | POLA2 |  |  | 2142 | 9.200 | 0.7271 | Yes |
| 46 | PSMA6 |  |  | 2205 | 9.000 | 0.7331 | Yes |
| 47 | PSMC3 |  |  | 2237 | 9.000 | 0.7411 | Yes |
| 48 | POLE |  |  | 2239 | 8.900 | 0.7508 | Yes |
| 49 | CDC7 |  |  | 2273 | 8.900 | 0.7585 | Yes |
| 50 | POLE2 |  |  | 2300 | 8.800 | 0.7666 | Yes |
| 51 | PSMD8 |  |  | 2504 | 8.200 | 0.7627 | Yes |
| 52 | PSMD9 |  |  | 2508 | 8.200 | 0.7716 | Yes |
| 53 | CDK2 |  |  | 2519 | 8.100 | 0.7799 | Yes |
| 54 | PSMD4 |  |  | 2609 | 7.900 | 0.7829 | Yes |
| 55 | PSMD13 |  |  | 2986 | 6.900 | 0.7665 | No |
| 56 | E2F3 |  |  | 3189 | 6.500 | 0.7608 | No |
| 57 | PSMB4 |  |  | 3191 | 6.500 | 0.7680 | No |
| 58 | MCM8 |  |  | 3515 | 5.700 | 0.7536 | No |
| 59 | CDC6 |  |  | 3705 | 5.300 | 0.7474 | No |
| 60 | PSMB5 |  |  | 4221 | 4.400 | 0.7195 | No |
| 61 | PSMF1 |  |  | 5043 | 3.000 | 0.6704 | No |
| 62 | UBA52 |  |  | 5629 | 2.100 | 0.6354 | No |
| 63 | PSMD3 |  |  | 5741 | 1.900 | 0.6304 | No |
| 64 | E2F2 |  |  | 7036 | 0.400 | 0.5484 | No |
| 65 | PSMA8 |  |  | 9516 | 0.000 | 0.3903 | No |
| 66 | PSMB6 |  |  | 9945 | 0.000 | 0.3630 | No |
| 67 | PSMB8 |  |  | 11241 | -0.300 | 0.2807 | No |
| 68 | PSMB10 |  |  | 13961 | -2.700 | 0.1103 | No |
| 69 | PSMB9 |  |  | 14266 | -3.300 | 0.0946 | No |
Table: GSEA details [plain text format]

  

Fig 2: REACTOME\_M\_G1\_TRANSITION: Random ES distribution      
 Gene set null distribution of ES for **REACTOME\_M\_G1\_TRANSITION**

  
