# Supplementary material for: Immunomic, genomic and transcriptomic characterization of CT26 colorectal carcinoma
Source: BMC Genomics. 2014 Mar 13;15(1):190. doi: 10.1186/1471-2164-15-190 (PMC4007559; doi:10.1186/1471-2164-15-190)
Supplement: Supplementary file 8 — Additional file 8: Contains the Gene Pattern gene set membership and enrichment values in an html format. The file index.html is the entry point. (ZIP 13 MB) [file 12864_2013_7028_MOESM8_ESM.zip › REACTOME_PEPTIDE_LIGAND_BINDING_RECEPTORS.html]

Details for gene set REACTOME\_PEPTIDE\_LIGAND\_BINDING\_RECEPTORS[GSEA]

|  || Dataset | CT26\_gene\_expression |
| Phenotype | NoPhenotypeAvailable |
| Upregulated in class | na\_neg |
| GeneSet | REACTOME\_PEPTIDE\_LIGAND\_BINDING\_RECEPTORS |
| Enrichment Score (ES) | -0.5125848 |
| Normalized Enrichment Score (NES) | NaN |
| Nominal p-value | NaN |
| FDR q-value | 1.0 |
| FWER p-Value | 0.0 |
Table: GSEA Results Summary

  

Fig 1: Enrichment plot: REACTOME\_PEPTIDE\_LIGAND\_BINDING\_RECEPTORS      
 Profile of the Running ES Score & Positions of GeneSet Members on the Rank Ordered List

  

| PROBE | GENE SYMBOL | GENE\_TITLE | RANK IN GENE LIST | RANK METRIC SCORE | RUNNING ES | CORE ENRICHMENT || 1 | ANXA1 |  |  | 697 | 16.400 | 0.0279 | No |
| 2 | HEBP1 |  |  | 1978 | 9.700 | -0.0113 | No |
| 3 | F2R |  |  | 2440 | 8.400 | -0.0037 | No |
| 4 | PMCH |  |  | 3480 | 5.800 | -0.0447 | No |
| 5 | CCL25 |  |  | 5788 | 1.900 | -0.1842 | No |
| 6 | PROKR1 |  |  | 6193 | 1.400 | -0.2040 | No |
| 7 | CXCL2 |  |  | 6388 | 1.100 | -0.2115 | No |
| 8 | CCR1 |  |  | 6492 | 0.900 | -0.2142 | No |
| 9 | CXCL10 |  |  | 7089 | 0.300 | -0.2511 | No |
| 10 | NPFF |  |  | 7140 | 0.300 | -0.2530 | No |
| 11 | NTSR2 |  |  | 7274 | 0.200 | -0.2606 | No |
| 12 | PPBP |  |  | 7319 | 0.100 | -0.2630 | No |
| 13 | OXT |  |  | 7433 | 0.100 | -0.2698 | No |
| 14 | CCL17 |  |  | 7491 | 0.100 | -0.2730 | No |
| 15 | UTS2 |  |  | 7534 | 0.000 | -0.2757 | No |
| 16 | GHRL |  |  | 7558 | 0.000 | -0.2772 | No |
| 17 | MC3R |  |  | 7564 | 0.000 | -0.2775 | No |
| 18 | PROK1 |  |  | 7579 | 0.000 | -0.2784 | No |
| 19 | TRHR |  |  | 7758 | 0.000 | -0.2898 | No |
| 20 | RXFP2 |  |  | 7821 | 0.000 | -0.2938 | No |
| 21 | NPS |  |  | 7825 | 0.000 | -0.2940 | No |
| 22 | MC1R |  |  | 8028 | 0.000 | -0.3069 | No |
| 23 | BRS3 |  |  | 8130 | 0.000 | -0.3134 | No |
| 24 | GAST |  |  | 8284 | 0.000 | -0.3232 | No |
| 25 | GHSR |  |  | 8289 | 0.000 | -0.3235 | No |
| 26 | NMS |  |  | 8485 | 0.000 | -0.3360 | No |
| 27 | NPSR1 |  |  | 8488 | 0.000 | -0.3361 | No |
| 28 | RLN2 |  |  | 8804 | 0.000 | -0.3563 | No |
| 29 | UTS2D |  |  | 8934 | 0.000 | -0.3646 | No |
| 30 | KNG1 |  |  | 8986 | 0.000 | -0.3679 | No |
| 31 | CXCL6 |  |  | 8999 | 0.000 | -0.3686 | No |
| 32 | RXFP1 |  |  | 9086 | 0.000 | -0.3742 | No |
| 33 | PROKR2 |  |  | 9145 | 0.000 | -0.3779 | No |
| 34 | NPFFR2 |  |  | 9150 | 0.000 | -0.3781 | No |
| 35 | TRH |  |  | 9182 | 0.000 | -0.3801 | No |
| 36 | TAC3 |  |  | 9300 | 0.000 | -0.3876 | No |
| 37 | UTS2R |  |  | 9367 | 0.000 | -0.3919 | No |
| 38 | NPY5R |  |  | 9385 | 0.000 | -0.3930 | No |
| 39 | HCRTR2 |  |  | 9388 | 0.000 | -0.3931 | No |
| 40 | NMUR2 |  |  | 9495 | 0.000 | -0.3999 | No |
| 41 | MC4R |  |  | 9554 | 0.000 | -0.4036 | No |
| 42 | OPRM1 |  |  | 9605 | 0.000 | -0.4068 | No |
| 43 | CCR3 |  |  | 9606 | 0.000 | -0.4068 | No |
| 44 | MC5R |  |  | 9630 | 0.000 | -0.4083 | No |
| 45 | AGTR2 |  |  | 9655 | 0.000 | -0.4098 | No |
| 46 | NMBR |  |  | 9686 | 0.000 | -0.4118 | No |
| 47 | RXFP3 |  |  | 9707 | 0.000 | -0.4130 | No |
| 48 | C5 |  |  | 9718 | 0.000 | -0.4137 | No |
| 49 | MC2R |  |  | 9747 | 0.000 | -0.4155 | No |
| 50 | CXCL3 |  |  | 9838 | 0.000 | -0.4213 | No |
| 51 | AVP |  |  | 9946 | 0.000 | -0.4281 | No |
| 52 | CXCL11 |  |  | 9974 | 0.000 | -0.4299 | No |
| 53 | NMB |  |  | 10045 | 0.000 | -0.4343 | No |
| 54 | CCKBR |  |  | 10084 | 0.000 | -0.4368 | No |
| 55 | RLN3 |  |  | 10099 | 0.000 | -0.4377 | No |
| 56 | FPR1 |  |  | 10156 | -0.100 | -0.4408 | No |
| 57 | NPBWR1 |  |  | 10190 | -0.100 | -0.4425 | No |
| 58 | XCR1 |  |  | 10268 | -0.100 | -0.4470 | No |
| 59 | OPRK1 |  |  | 10357 | -0.100 | -0.4522 | No |
| 60 | CCL4 |  |  | 10361 | -0.100 | -0.4520 | No |
| 61 | AVPR2 |  |  | 10377 | -0.100 | -0.4525 | No |
| 62 | PNOC |  |  | 10385 | -0.100 | -0.4525 | No |
| 63 | NPW |  |  | 10389 | -0.100 | -0.4522 | No |
| 64 | CCR8 |  |  | 10423 | -0.100 | -0.4539 | No |
| 65 | GPR77 |  |  | 10438 | -0.100 | -0.4544 | No |
| 66 | RXFP4 |  |  | 10457 | -0.100 | -0.4551 | No |
| 67 | TACR3 |  |  | 10461 | -0.100 | -0.4548 | No |
| 68 | CCR9 |  |  | 10533 | -0.100 | -0.4589 | No |
| 69 | CXCL9 |  |  | 10552 | -0.100 | -0.4596 | No |
| 70 | PRLHR |  |  | 10601 | -0.100 | -0.4623 | No |
| 71 | PDYN |  |  | 10628 | -0.100 | -0.4635 | No |
| 72 | CCR4 |  |  | 10656 | -0.100 | -0.4648 | No |
| 73 | LOC728830 |  |  | 10672 | -0.100 | -0.4653 | No |
| 74 | HCRT |  |  | 10681 | -0.100 | -0.4654 | No |
| 75 | XCL1 |  |  | 10837 | -0.200 | -0.4744 | No |
| 76 | GALR3 |  |  | 10878 | -0.200 | -0.4761 | No |
| 77 | OXTR |  |  | 10880 | -0.200 | -0.4753 | No |
| 78 | POMC |  |  | 10894 | -0.200 | -0.4753 | No |
| 79 | CCR2 |  |  | 10919 | -0.200 | -0.4759 | No |
| 80 | OPRL1 |  |  | 10930 | -0.200 | -0.4757 | No |
| 81 | GALR1 |  |  | 10975 | -0.200 | -0.4776 | No |
| 82 | CCL7 |  |  | 11021 | -0.200 | -0.4796 | No |
| 83 | AVPR1B |  |  | 11056 | -0.200 | -0.4809 | No |
| 84 | CXCR6 |  |  | 11100 | -0.200 | -0.4828 | No |
| 85 | CCL2 |  |  | 11114 | -0.200 | -0.4827 | No |
| 86 | F2RL2 |  |  | 11120 | -0.200 | -0.4822 | No |
| 87 | GRPR |  |  | 11139 | -0.200 | -0.4824 | No |
| 88 | NPY2R |  |  | 11142 | -0.200 | -0.4817 | No |
| 89 | SSTR5 |  |  | 11181 | -0.300 | -0.4828 | No |
| 90 | SSTR3 |  |  | 11204 | -0.300 | -0.4829 | No |
| 91 | F2 |  |  | 11305 | -0.300 | -0.4879 | No |
| 92 | TACR1 |  |  | 11308 | -0.300 | -0.4867 | No |
| 93 | NPFFR1 |  |  | 11432 | -0.300 | -0.4933 | No |
| 94 | CCL19 |  |  | 11443 | -0.300 | -0.4926 | No |
| 95 | NTS |  |  | 11525 | -0.400 | -0.4960 | No |
| 96 | BDKRB1 |  |  | 11619 | -0.400 | -0.5002 | No |
| 97 | OPRD1 |  |  | 11624 | -0.400 | -0.4987 | No |
| 98 | NTSR1 |  |  | 11651 | -0.400 | -0.4986 | No |
| 99 | CCR6 |  |  | 11662 | -0.500 | -0.4971 | No |
| 100 | SSTR4 |  |  | 11739 | -0.500 | -0.4997 | No |
| 101 | EDN1 |  |  | 11777 | -0.500 | -0.4999 | No |
| 102 | PRLH |  |  | 11811 | -0.500 | -0.4998 | No |
| 103 | C3AR1 |  |  | 11965 | -0.600 | -0.5069 | No |
| 104 | NPY1R |  |  | 12054 | -0.700 | -0.5095 | Yes |
| 105 | CCBP2 |  |  | 12069 | -0.700 | -0.5073 | Yes |
| 106 | MCHR1 |  |  | 12108 | -0.700 | -0.5066 | Yes |
| 107 | CCL20 |  |  | 12138 | -0.700 | -0.5054 | Yes |
| 108 | EDNRB |  |  | 12155 | -0.700 | -0.5033 | Yes |
| 109 | KISS1 |  |  | 12176 | -0.800 | -0.5011 | Yes |
| 110 | EDN3 |  |  | 12211 | -0.800 | -0.4997 | Yes |
| 111 | CCL21 |  |  | 12271 | -0.800 | -0.4999 | Yes |
| 112 | CCL22 |  |  | 12279 | -0.800 | -0.4968 | Yes |
| 113 | CXCL13 |  |  | 12321 | -0.900 | -0.4955 | Yes |
| 114 | AVPR1A |  |  | 12399 | -0.900 | -0.4964 | Yes |
| 115 | NMUR1 |  |  | 12483 | -1.000 | -0.4973 | Yes |
| 116 | CXCR3 |  |  | 12696 | -1.100 | -0.5061 | Yes |
| 117 | CCR7 |  |  | 12772 | -1.200 | -0.5056 | Yes |
| 118 | CCKAR |  |  | 12831 | -1.300 | -0.5035 | Yes |
| 119 | HCRTR1 |  |  | 12888 | -1.300 | -0.5014 | Yes |
| 120 | GRP |  |  | 12978 | -1.400 | -0.5009 | Yes |
| 121 | APP |  |  | 13046 | -1.500 | -0.4986 | Yes |
| 122 | NMU |  |  | 13073 | -1.500 | -0.4936 | Yes |
| 123 | CCL28 |  |  | 13098 | -1.600 | -0.4880 | Yes |
| 124 | AGTR1 |  |  | 13128 | -1.600 | -0.4828 | Yes |
| 125 | EDNRA |  |  | 13135 | -1.600 | -0.4761 | Yes |
| 126 | PENK |  |  | 13156 | -1.600 | -0.4703 | Yes |
| 127 | KISS1R |  |  | 13212 | -1.700 | -0.4663 | Yes |
| 128 | GALR2 |  |  | 13227 | -1.700 | -0.4597 | Yes |
| 129 | CCL27 |  |  | 13347 | -1.800 | -0.4594 | Yes |
| 130 | NPY |  |  | 13555 | -2.100 | -0.4633 | Yes |
| 131 | CX3CR1 |  |  | 13586 | -2.100 | -0.4560 | Yes |
| 132 | SSTR2 |  |  | 13633 | -2.200 | -0.4492 | Yes |
| 133 | CCL11 |  |  | 13654 | -2.200 | -0.4407 | Yes |
| 134 | CCK |  |  | 13672 | -2.300 | -0.4316 | Yes |
| 135 | CCL5 |  |  | 13706 | -2.300 | -0.4236 | Yes |
| 136 | CCR10 |  |  | 13708 | -2.300 | -0.4135 | Yes |
| 137 | F2RL3 |  |  | 13786 | -2.400 | -0.4078 | Yes |
| 138 | BDKRB2 |  |  | 13805 | -2.500 | -0.3979 | Yes |
| 139 | CCRL1 |  |  | 13834 | -2.500 | -0.3886 | Yes |
| 140 | PPYR1 |  |  | 13845 | -2.600 | -0.3777 | Yes |
| 141 | C5AR1 |  |  | 13884 | -2.600 | -0.3686 | Yes |
| 142 | TAC1 |  |  | 13930 | -2.700 | -0.3596 | Yes |
| 143 | INSL3 |  |  | 13939 | -2.700 | -0.3481 | Yes |
| 144 | DARC |  |  | 14153 | -3.000 | -0.3485 | Yes |
| 145 | PPY |  |  | 14193 | -3.100 | -0.3373 | Yes |
| 146 | CXCL12 |  |  | 14228 | -3.200 | -0.3253 | Yes |
| 147 | CXCR7 |  |  | 14254 | -3.200 | -0.3128 | Yes |
| 148 | CXCR4 |  |  | 14374 | -3.500 | -0.3049 | Yes |
| 149 | PF4 |  |  | 14433 | -3.700 | -0.2922 | Yes |
| 150 | SAA1 |  |  | 14809 | -4.600 | -0.2959 | Yes |
| 151 | AGT |  |  | 14844 | -4.700 | -0.2773 | Yes |
| 152 | TACR2 |  |  | 14856 | -4.700 | -0.2572 | Yes |
| 153 | CXCL16 |  |  | 14924 | -4.900 | -0.2398 | Yes |
| 154 | INSL5 |  |  | 15099 | -5.700 | -0.2257 | Yes |
| 155 | GAL |  |  | 15193 | -6.100 | -0.2047 | Yes |
| 156 | CX3CL1 |  |  | 15197 | -6.200 | -0.1775 | Yes |
| 157 | SSTR1 |  |  | 15216 | -6.300 | -0.1507 | Yes |
| 158 | EDN2 |  |  | 15252 | -6.400 | -0.1246 | Yes |
| 159 | PYY |  |  | 15309 | -6.800 | -0.0981 | Yes |
| 160 | F2RL1 |  |  | 15456 | -8.000 | -0.0721 | Yes |
| 161 | SST |  |  | 15599 | -10.200 | -0.0360 | Yes |
| 162 | C3 |  |  | 15605 | -10.300 | 0.0092 | Yes |
Table: GSEA details [plain text format]

  

Fig 2: REACTOME\_PEPTIDE\_LIGAND\_BINDING\_RECEPTORS: Random ES distribution      
 Gene set null distribution of ES for **REACTOME\_PEPTIDE\_LIGAND\_BINDING\_RECEPTORS**

  
