# Supplementary material for: Immunomic, genomic and transcriptomic characterization of CT26 colorectal carcinoma
Source: BMC Genomics. 2014 Mar 13;15(1):190. doi: 10.1186/1471-2164-15-190 (PMC4007559; doi:10.1186/1471-2164-15-190)
Supplement: Supplementary file 8 — Additional file 8: Contains the Gene Pattern gene set membership and enrichment values in an html format. The file index.html is the entry point. (ZIP 13 MB) [file 12864_2013_7028_MOESM8_ESM.zip › REACTOME_PHOSPHOLIPID_METABOLISM.html]

Details for gene set REACTOME\_PHOSPHOLIPID\_METABOLISM[GSEA]

|  || Dataset | CT26\_gene\_expression |
| Phenotype | NoPhenotypeAvailable |
| Upregulated in class | na\_neg |
| GeneSet | REACTOME\_PHOSPHOLIPID\_METABOLISM |
| Enrichment Score (ES) | -0.23851122 |
| Normalized Enrichment Score (NES) | NaN |
| Nominal p-value | NaN |
| FDR q-value | 1.0 |
| FWER p-Value | 0.0 |
Table: GSEA Results Summary

  

Fig 1: Enrichment plot: REACTOME\_PHOSPHOLIPID\_METABOLISM      
 Profile of the Running ES Score & Positions of GeneSet Members on the Rank Ordered List

  

| PROBE | GENE SYMBOL | GENE\_TITLE | RANK IN GENE LIST | RANK METRIC SCORE | RUNNING ES | CORE ENRICHMENT || 1 | PLA2G4A |  |  | 168 | 26.500 | 0.0269 | No |
| 2 | DEGS1 |  |  | 426 | 19.500 | 0.0381 | No |
| 3 | ASAH1 |  |  | 490 | 18.600 | 0.0605 | No |
| 4 | SACM1L |  |  | 552 | 18.000 | 0.0822 | No |
| 5 | PLA2G12A |  |  | 703 | 16.400 | 0.0959 | No |
| 6 | PITPNB |  |  | 725 | 16.200 | 0.1176 | No |
| 7 | LPGAT1 |  |  | 1035 | 14.000 | 0.1177 | No |
| 8 | AGPAT6 |  |  | 1120 | 13.400 | 0.1314 | No |
| 9 | CRLS1 |  |  | 1189 | 13.100 | 0.1456 | No |
| 10 | CEPT1 |  |  | 1232 | 12.900 | 0.1613 | No |
| 11 | MTMR2 |  |  | 1504 | 11.600 | 0.1604 | No |
| 12 | SYNJ2 |  |  | 1947 | 9.900 | 0.1461 | No |
| 13 | VAPB |  |  | 1994 | 9.700 | 0.1569 | No |
| 14 | PIK3CB |  |  | 2292 | 8.800 | 0.1504 | No |
| 15 | OCRL |  |  | 2658 | 7.800 | 0.1381 | No |
| 16 | PTEN |  |  | 2795 | 7.400 | 0.1399 | No |
| 17 | SGMS1 |  |  | 2989 | 6.900 | 0.1373 | No |
| 18 | OSBP |  |  | 3014 | 6.900 | 0.1456 | No |
| 19 | PIK3C2A |  |  | 3023 | 6.800 | 0.1547 | No |
| 20 | PIK3CA |  |  | 3066 | 6.800 | 0.1617 | No |
| 21 | ARF3 |  |  | 3083 | 6.700 | 0.1702 | No |
| 22 | SGMS2 |  |  | 3302 | 6.200 | 0.1650 | No |
| 23 | PNPLA8 |  |  | 3333 | 6.200 | 0.1719 | No |
| 24 | PIK3C3 |  |  | 3430 | 5.900 | 0.1742 | No |
| 25 | COL4A3BP |  |  | 3693 | 5.400 | 0.1650 | No |
| 26 | SGPL1 |  |  | 3701 | 5.300 | 0.1721 | No |
| 27 | MTMR6 |  |  | 3728 | 5.300 | 0.1780 | No |
| 28 | ARSB |  |  | 3813 | 5.100 | 0.1799 | No |
| 29 | CHPT1 |  |  | 3892 | 5.000 | 0.1820 | No |
| 30 | HADHA |  |  | 3998 | 4.800 | 0.1821 | No |
| 31 | ARSJ |  |  | 4000 | 4.800 | 0.1888 | No |
| 32 | PGS1 |  |  | 4030 | 4.700 | 0.1936 | No |
| 33 | GNPAT |  |  | 4045 | 4.700 | 0.1994 | No |
| 34 | PPM1L |  |  | 4116 | 4.600 | 0.2015 | No |
| 35 | ETNK1 |  |  | 4248 | 4.300 | 0.1992 | No |
| 36 | MTMR1 |  |  | 4283 | 4.300 | 0.2031 | No |
| 37 | HADHB |  |  | 4298 | 4.200 | 0.2082 | No |
| 38 | VAC14 |  |  | 4385 | 4.100 | 0.2085 | No |
| 39 | INPP5E |  |  | 4409 | 4.000 | 0.2127 | No |
| 40 | SUMF1 |  |  | 4422 | 4.000 | 0.2176 | No |
| 41 | LPIN1 |  |  | 4431 | 4.000 | 0.2228 | No |
| 42 | TAZ |  |  | 4548 | 3.800 | 0.2208 | No |
| 43 | SGPP1 |  |  | 4583 | 3.700 | 0.2238 | No |
| 44 | PCYT1A |  |  | 4792 | 3.400 | 0.2153 | No |
| 45 | GLA |  |  | 4878 | 3.300 | 0.2146 | No |
| 46 | PIK3R1 |  |  | 5018 | 3.000 | 0.2099 | No |
| 47 | CHKA |  |  | 5050 | 3.000 | 0.2122 | No |
| 48 | SMPD4 |  |  | 5106 | 2.900 | 0.2128 | No |
| 49 | PTDSS1 |  |  | 5148 | 2.800 | 0.2142 | No |
| 50 | SYNJ1 |  |  | 5181 | 2.800 | 0.2161 | No |
| 51 | CDS2 |  |  | 5281 | 2.600 | 0.2134 | No |
| 52 | SPTLC1 |  |  | 5323 | 2.500 | 0.2144 | No |
| 53 | PLD2 |  |  | 5362 | 2.500 | 0.2155 | No |
| 54 | MTMR4 |  |  | 5594 | 2.200 | 0.2038 | No |
| 55 | PIK3R2 |  |  | 5623 | 2.100 | 0.2050 | No |
| 56 | PI4K2B |  |  | 5645 | 2.100 | 0.2066 | No |
| 57 | VAPA |  |  | 5723 | 2.000 | 0.2045 | No |
| 58 | LPIN2 |  |  | 5778 | 1.900 | 0.2038 | No |
| 59 | GALC |  |  | 5888 | 1.800 | 0.1993 | No |
| 60 | MTMR14 |  |  | 5891 | 1.800 | 0.2018 | No |
| 61 | MTMR3 |  |  | 5906 | 1.700 | 0.2033 | No |
| 62 | SPTLC2 |  |  | 5941 | 1.700 | 0.2035 | No |
| 63 | PCYT1B |  |  | 6050 | 1.600 | 0.1989 | No |
| 64 | PIK3R3 |  |  | 6055 | 1.500 | 0.2007 | No |
| 65 | ARSK |  |  | 6095 | 1.500 | 0.2004 | No |
| 66 | PLA2G6 |  |  | 6332 | 1.200 | 0.1869 | No |
| 67 | ARSA |  |  | 6346 | 1.100 | 0.1877 | No |
| 68 | UGCG |  |  | 6397 | 1.100 | 0.1860 | No |
| 69 | PRKD1 |  |  | 6497 | 0.900 | 0.1810 | No |
| 70 | PLA2G4C |  |  | 6509 | 0.900 | 0.1815 | No |
| 71 | PIK3R4 |  |  | 6527 | 0.900 | 0.1817 | No |
| 72 | ASAH2 |  |  | 6675 | 0.700 | 0.1733 | No |
| 73 | GPAM |  |  | 6697 | 0.700 | 0.1729 | No |
| 74 | PLA2G2E |  |  | 6748 | 0.700 | 0.1707 | No |
| 75 | MTMR7 |  |  | 6880 | 0.500 | 0.1630 | No |
| 76 | AGPAT5 |  |  | 6914 | 0.500 | 0.1616 | No |
| 77 | AGPAT3 |  |  | 6988 | 0.400 | 0.1575 | No |
| 78 | INPP4B |  |  | 7088 | 0.300 | 0.1516 | No |
| 79 | PLD1 |  |  | 7171 | 0.200 | 0.1466 | No |
| 80 | TPTE2 |  |  | 8911 | 0.000 | 0.0351 | No |
| 81 | NEU4 |  |  | 9312 | 0.000 | 0.0094 | No |
| 82 | PLA2G4D |  |  | 9323 | 0.000 | 0.0088 | No |
| 83 | ENPP7 |  |  | 9328 | 0.000 | 0.0085 | No |
| 84 | PIK3C2G |  |  | 9537 | 0.000 | -0.0048 | No |
| 85 | SLC44A5 |  |  | 9666 | 0.000 | -0.0130 | No |
| 86 | CHAT |  |  | 10420 | -0.100 | -0.0611 | No |
| 87 | MTM1 |  |  | 10623 | -0.100 | -0.0740 | No |
| 88 | PIK3CD |  |  | 10848 | -0.200 | -0.0880 | No |
| 89 | ETNK2 |  |  | 10883 | -0.200 | -0.0899 | No |
| 90 | PEMT |  |  | 11375 | -0.300 | -0.1210 | No |
| 91 | NEU3 |  |  | 11487 | -0.400 | -0.1275 | No |
| 92 | SLC44A2 |  |  | 11504 | -0.400 | -0.1280 | No |
| 93 | PNPLA3 |  |  | 11575 | -0.400 | -0.1319 | No |
| 94 | PLA2G2D |  |  | 11611 | -0.400 | -0.1336 | No |
| 95 | NEU2 |  |  | 11727 | -0.500 | -0.1403 | No |
| 96 | SMPD1 |  |  | 11839 | -0.600 | -0.1465 | No |
| 97 | MBOAT2 |  |  | 11982 | -0.600 | -0.1548 | No |
| 98 | ARSG |  |  | 12143 | -0.700 | -0.1641 | No |
| 99 | PPAP2A |  |  | 12152 | -0.700 | -0.1636 | No |
| 100 | PLA2G1B |  |  | 12340 | -0.900 | -0.1743 | No |
| 101 | PIK3CG |  |  | 12345 | -0.900 | -0.1733 | No |
| 102 | PLA2G2A |  |  | 12402 | -0.900 | -0.1756 | No |
| 103 | GLB1 |  |  | 12701 | -1.100 | -0.1931 | No |
| 104 | GBA |  |  | 12756 | -1.200 | -0.1949 | No |
| 105 | MBOAT1 |  |  | 12796 | -1.200 | -0.1957 | No |
| 106 | PIK3R5 |  |  | 13133 | -1.600 | -0.2149 | No |
| 107 | CHKB |  |  | 13216 | -1.700 | -0.2178 | No |
| 108 | SLC44A1 |  |  | 13270 | -1.700 | -0.2188 | No |
| 109 | INPPL1 |  |  | 13280 | -1.800 | -0.2168 | No |
| 110 | SUMF2 |  |  | 13283 | -1.800 | -0.2144 | No |
| 111 | CDIPT |  |  | 13346 | -1.800 | -0.2158 | No |
| 112 | PIP5K1C |  |  | 13480 | -2.000 | -0.2215 | No |
| 113 | SPHK1 |  |  | 13514 | -2.000 | -0.2207 | No |
| 114 | PTDSS2 |  |  | 13684 | -2.300 | -0.2283 | No |
| 115 | CERK |  |  | 13711 | -2.300 | -0.2267 | No |
| 116 | PLA2G3 |  |  | 13736 | -2.400 | -0.2248 | No |
| 117 | ARSI |  |  | 13748 | -2.400 | -0.2221 | No |
| 118 | PPAP2C |  |  | 13754 | -2.400 | -0.2190 | No |
| 119 | GBA2 |  |  | 13847 | -2.600 | -0.2212 | No |
| 120 | GPD1L |  |  | 14023 | -2.800 | -0.2285 | No |
| 121 | PHOSPHO1 |  |  | 14147 | -3.000 | -0.2321 | No |
| 122 | PLA2G4F |  |  | 14248 | -3.200 | -0.2340 | Yes |
| 123 | ACHE |  |  | 14259 | -3.200 | -0.2301 | Yes |
| 124 | AGPAT1 |  |  | 14350 | -3.400 | -0.2310 | Yes |
| 125 | HEXA |  |  | 14447 | -3.700 | -0.2319 | Yes |
| 126 | PLA2G5 |  |  | 14516 | -3.900 | -0.2307 | Yes |
| 127 | SPHK2 |  |  | 14572 | -4.000 | -0.2285 | Yes |
| 128 | NEU1 |  |  | 14592 | -4.100 | -0.2239 | Yes |
| 129 | PNPLA2 |  |  | 14647 | -4.200 | -0.2214 | Yes |
| 130 | MGLL |  |  | 14742 | -4.400 | -0.2212 | Yes |
| 131 | AGPAT2 |  |  | 14831 | -4.700 | -0.2202 | Yes |
| 132 | PPAP2B |  |  | 14867 | -4.800 | -0.2156 | Yes |
| 133 | ARF1 |  |  | 14888 | -4.800 | -0.2100 | Yes |
| 134 | SLC44A3 |  |  | 15001 | -5.200 | -0.2098 | Yes |
| 135 | GPD1 |  |  | 15017 | -5.300 | -0.2033 | Yes |
| 136 | PLD4 |  |  | 15025 | -5.300 | -0.1962 | Yes |
| 137 | PIP5K1B |  |  | 15043 | -5.400 | -0.1896 | Yes |
| 138 | PIK3C2B |  |  | 15054 | -5.400 | -0.1826 | Yes |
| 139 | SLC44A4 |  |  | 15093 | -5.600 | -0.1770 | Yes |
| 140 | PCYT2 |  |  | 15144 | -5.900 | -0.1718 | Yes |
| 141 | CSNK1G2 |  |  | 15184 | -6.100 | -0.1657 | Yes |
| 142 | GAL3ST1 |  |  | 15185 | -6.100 | -0.1570 | Yes |
| 143 | LPIN3 |  |  | 15186 | -6.100 | -0.1483 | Yes |
| 144 | AGPAT4 |  |  | 15198 | -6.200 | -0.1402 | Yes |
| 145 | PSAP |  |  | 15338 | -7.000 | -0.1392 | Yes |
| 146 | GM2A |  |  | 15342 | -7.000 | -0.1294 | Yes |
| 147 | SGPP2 |  |  | 15370 | -7.300 | -0.1208 | Yes |
| 148 | HEXB |  |  | 15432 | -7.800 | -0.1136 | Yes |
| 149 | CTSA |  |  | 15485 | -8.400 | -0.1050 | Yes |
| 150 | SMPD2 |  |  | 15494 | -8.500 | -0.0934 | Yes |
| 151 | DEGS2 |  |  | 15521 | -8.800 | -0.0826 | Yes |
| 152 | SMPD3 |  |  | 15555 | -9.500 | -0.0712 | Yes |
| 153 | PLA2G2F |  |  | 15563 | -9.500 | -0.0581 | Yes |
| 154 | CDS1 |  |  | 15590 | -10.000 | -0.0456 | Yes |
| 155 | PLD3 |  |  | 15629 | -11.200 | -0.0321 | Yes |
| 156 | DGAT2 |  |  | 15632 | -11.400 | -0.0160 | Yes |
| 157 | PLA2G10 |  |  | 15715 | -16.500 | 0.0022 | Yes |
Table: GSEA details [plain text format]

  

Fig 2: REACTOME\_PHOSPHOLIPID\_METABOLISM: Random ES distribution      
 Gene set null distribution of ES for **REACTOME\_PHOSPHOLIPID\_METABOLISM**

  
