# Supplementary material for: Immunomic, genomic and transcriptomic characterization of CT26 colorectal carcinoma
Source: BMC Genomics. 2014 Mar 13;15(1):190. doi: 10.1186/1471-2164-15-190 (PMC4007559; doi:10.1186/1471-2164-15-190)
Supplement: Supplementary file 8 — Additional file 8: Contains the Gene Pattern gene set membership and enrichment values in an html format. The file index.html is the entry point. (ZIP 13 MB) [file 12864_2013_7028_MOESM8_ESM.zip › REICHERT_MITOSIS_LIN9_TARGETS.html]

Details for gene set REICHERT\_MITOSIS\_LIN9\_TARGETS[GSEA]

|  || Dataset | CT26\_gene\_expression |
| Phenotype | NoPhenotypeAvailable |
| Upregulated in class | na\_pos |
| GeneSet | REICHERT\_MITOSIS\_LIN9\_TARGETS |
| Enrichment Score (ES) | 0.8496788 |
| Normalized Enrichment Score (NES) | 1.7263824 |
| Nominal p-value | 0.0 |
| FDR q-value | 0.0012002243 |
| FWER p-Value | 0.0080 |
Table: GSEA Results Summary

  

Fig 1: Enrichment plot: REICHERT\_MITOSIS\_LIN9\_TARGETS      
 Profile of the Running ES Score & Positions of GeneSet Members on the Rank Ordered List

  

| PROBE | GENE SYMBOL | GENE\_TITLE | RANK IN GENE LIST | RANK METRIC SCORE | RUNNING ES | CORE ENRICHMENT || 1 | CALD1 |  |  | 0 | 95.000 | 0.1860 | Yes |
| 2 | TOP2A |  |  | 8 | 56.000 | 0.2952 | Yes |
| 3 | NCAPD2 |  |  | 55 | 35.000 | 0.3608 | Yes |
| 4 | KIF20A |  |  | 77 | 31.900 | 0.4219 | Yes |
| 5 | HMMR |  |  | 246 | 23.200 | 0.4566 | Yes |
| 6 | KIF23 |  |  | 337 | 21.000 | 0.4920 | Yes |
| 7 | CENPE |  |  | 391 | 20.100 | 0.5280 | Yes |
| 8 | MKI67 |  |  | 414 | 19.700 | 0.5651 | Yes |
| 9 | CENPF |  |  | 431 | 19.400 | 0.6021 | Yes |
| 10 | ANAPC1 |  |  | 453 | 19.100 | 0.6382 | Yes |
| 11 | CEP55 |  |  | 662 | 16.800 | 0.6578 | Yes |
| 12 | FBXO5 |  |  | 688 | 16.500 | 0.6885 | Yes |
| 13 | NUSAP1 |  |  | 716 | 16.200 | 0.7185 | Yes |
| 14 | AURKA |  |  | 799 | 15.600 | 0.7439 | Yes |
| 15 | KIF2C |  |  | 1036 | 14.000 | 0.7563 | Yes |
| 16 | CCNF |  |  | 1108 | 13.500 | 0.7782 | Yes |
| 17 | HMGB2 |  |  | 1279 | 12.700 | 0.7922 | Yes |
| 18 | CDCA2 |  |  | 1643 | 11.100 | 0.7909 | Yes |
| 19 | CENPA |  |  | 1762 | 10.600 | 0.8041 | Yes |
| 20 | CASC5 |  |  | 1912 | 10.000 | 0.8142 | Yes |
| 21 | ASPM |  |  | 1918 | 10.000 | 0.8335 | Yes |
| 22 | VCPIP1 |  |  | 1966 | 9.800 | 0.8497 | Yes |
| 23 | CCNB1 |  |  | 2266 | 8.900 | 0.8481 | No |
| 24 | PLK1 |  |  | 6602 | 0.800 | 0.5740 | No |
| 25 | LMLN |  |  | 6652 | 0.800 | 0.5724 | No |
| 26 | MYO6 |  |  | 14195 | -3.100 | 0.0988 | No |
Table: GSEA details [plain text format]

  

Fig 2: REICHERT\_MITOSIS\_LIN9\_TARGETS: Random ES distribution      
 Gene set null distribution of ES for **REICHERT\_MITOSIS\_LIN9\_TARGETS**

  
