# Supplementary material for: Immunomic, genomic and transcriptomic characterization of CT26 colorectal carcinoma
Source: BMC Genomics. 2014 Mar 13;15(1):190. doi: 10.1186/1471-2164-15-190 (PMC4007559; doi:10.1186/1471-2164-15-190)
Supplement: Supplementary file 8 — Additional file 8: Contains the Gene Pattern gene set membership and enrichment values in an html format. The file index.html is the entry point. (ZIP 13 MB) [file 12864_2013_7028_MOESM8_ESM.zip › RHODES_UNDIFFERENTIATED_CANCER.html]

Details for gene set RHODES\_UNDIFFERENTIATED\_CANCER[GSEA]

|  || Dataset | CT26\_gene\_expression |
| Phenotype | NoPhenotypeAvailable |
| Upregulated in class | na\_pos |
| GeneSet | RHODES\_UNDIFFERENTIATED\_CANCER |
| Enrichment Score (ES) | 0.78445584 |
| Normalized Enrichment Score (NES) | 1.6995542 |
| Nominal p-value | 0.0 |
| FDR q-value | 0.0016507958 |
| FWER p-Value | 0.022 |
Table: GSEA Results Summary

  

Fig 1: Enrichment plot: RHODES\_UNDIFFERENTIATED\_CANCER      
 Profile of the Running ES Score & Positions of GeneSet Members on the Rank Ordered List

  

| PROBE | GENE SYMBOL | GENE\_TITLE | RANK IN GENE LIST | RANK METRIC SCORE | RUNNING ES | CORE ENRICHMENT || 1 | TOP2A |  |  | 8 | 56.000 | 0.0539 | Yes |
| 2 | CKS1B |  |  | 48 | 35.600 | 0.0860 | Yes |
| 3 | EIF2S2 |  |  | 50 | 35.400 | 0.1203 | Yes |
| 4 | NCAPD2 |  |  | 55 | 35.000 | 0.1541 | Yes |
| 5 | RAD21 |  |  | 86 | 31.400 | 0.1827 | Yes |
| 6 | TUBB |  |  | 153 | 26.800 | 0.2045 | Yes |
| 7 | MCM6 |  |  | 163 | 26.600 | 0.2298 | Yes |
| 8 | UBE2S |  |  | 177 | 25.600 | 0.2538 | Yes |
| 9 | CCNA2 |  |  | 178 | 25.600 | 0.2787 | Yes |
| 10 | KIAA0101 |  |  | 186 | 25.100 | 0.3026 | Yes |
| 11 | YBX1 |  |  | 219 | 24.100 | 0.3240 | Yes |
| 12 | PSMD2 |  |  | 227 | 23.700 | 0.3466 | Yes |
| 13 | MTHFD2 |  |  | 255 | 22.900 | 0.3671 | Yes |
| 14 | CCT6A |  |  | 273 | 22.500 | 0.3879 | Yes |
| 15 | CDC20 |  |  | 284 | 22.200 | 0.4088 | Yes |
| 16 | EZH2 |  |  | 286 | 22.000 | 0.4302 | Yes |
| 17 | MCM3 |  |  | 315 | 21.400 | 0.4492 | Yes |
| 18 | FOXM1 |  |  | 322 | 21.200 | 0.4694 | Yes |
| 19 | KIF23 |  |  | 337 | 21.000 | 0.4889 | Yes |
| 20 | KPNA2 |  |  | 354 | 20.700 | 0.5080 | Yes |
| 21 | PCNA |  |  | 386 | 20.200 | 0.5256 | Yes |
| 22 | ILF2 |  |  | 388 | 20.100 | 0.5451 | Yes |
| 23 | NCAPH |  |  | 459 | 19.000 | 0.5591 | Yes |
| 24 | TMSB10 |  |  | 481 | 18.600 | 0.5758 | Yes |
| 25 | BIRC5 |  |  | 507 | 18.400 | 0.5921 | Yes |
| 26 | CEBPG |  |  | 532 | 18.100 | 0.6082 | Yes |
| 27 | NME1 |  |  | 540 | 18.100 | 0.6253 | Yes |
| 28 | SLC7A5 |  |  | 542 | 18.100 | 0.6428 | Yes |
| 29 | MAD2L1 |  |  | 559 | 17.900 | 0.6592 | Yes |
| 30 | GARS |  |  | 745 | 16.000 | 0.6630 | Yes |
| 31 | PSMD14 |  |  | 859 | 15.100 | 0.6704 | Yes |
| 32 | COL1A2 |  |  | 916 | 14.700 | 0.6811 | Yes |
| 33 | H2AFZ |  |  | 982 | 14.300 | 0.6909 | Yes |
| 34 | KIF2C |  |  | 1036 | 14.000 | 0.7011 | Yes |
| 35 | MELK |  |  | 1077 | 13.700 | 0.7119 | Yes |
| 36 | TRIP13 |  |  | 1182 | 13.100 | 0.7180 | Yes |
| 37 | PRDX4 |  |  | 1246 | 12.800 | 0.7264 | Yes |
| 38 | HMGB2 |  |  | 1279 | 12.700 | 0.7367 | Yes |
| 39 | MCM2 |  |  | 1281 | 12.700 | 0.7490 | Yes |
| 40 | PSMB7 |  |  | 1288 | 12.700 | 0.7609 | Yes |
| 41 | RPA3 |  |  | 1621 | 11.200 | 0.7506 | Yes |
| 42 | CENPA |  |  | 1762 | 10.600 | 0.7520 | Yes |
| 43 | SSBP1 |  |  | 1831 | 10.300 | 0.7577 | Yes |
| 44 | CKS2 |  |  | 1959 | 9.800 | 0.7591 | Yes |
| 45 | MYBL2 |  |  | 2007 | 9.600 | 0.7654 | Yes |
| 46 | ADRM1 |  |  | 2035 | 9.500 | 0.7730 | Yes |
| 47 | GPSM2 |  |  | 2213 | 9.000 | 0.7704 | Yes |
| 48 | RFC4 |  |  | 2243 | 8.900 | 0.7772 | Yes |
| 49 | CCNB1 |  |  | 2266 | 8.900 | 0.7845 | Yes |
| 50 | DPM1 |  |  | 2854 | 7.300 | 0.7541 | No |
| 51 | UBE2C |  |  | 2879 | 7.200 | 0.7596 | No |
| 52 | CDKN3 |  |  | 2917 | 7.100 | 0.7641 | No |
| 53 | NUDT1 |  |  | 3655 | 5.400 | 0.7224 | No |
| 54 | CDC6 |  |  | 3705 | 5.300 | 0.7244 | No |
| 55 | POLR2K |  |  | 3717 | 5.300 | 0.7289 | No |
| 56 | SLC16A1 |  |  | 4053 | 4.700 | 0.7121 | No |
| 57 | KIF14 |  |  | 4209 | 4.400 | 0.7065 | No |
| 58 | TAP1 |  |  | 6230 | 1.300 | 0.5789 | No |
| 59 | GCLM |  |  | 6379 | 1.100 | 0.5706 | No |
| 60 | CXCL9 |  |  | 10552 | -0.100 | 0.3047 | No |
| 61 | H2AFX |  |  | 14159 | -3.000 | 0.0777 | No |
| 62 | IFI30 |  |  | 14652 | -4.200 | 0.0504 | No |
| 63 | SEC61B |  |  | 15261 | -6.500 | 0.0180 | No |
| 64 | GAS6 |  |  | 15277 | -6.600 | 0.0234 | No |
| 65 | GGH |  |  | 15304 | -6.800 | 0.0284 | No |
Table: GSEA details [plain text format]

  

Fig 2: RHODES\_UNDIFFERENTIATED\_CANCER: Random ES distribution      
 Gene set null distribution of ES for **RHODES\_UNDIFFERENTIATED\_CANCER**

  
