# Supplementary material for: Immunomic, genomic and transcriptomic characterization of CT26 colorectal carcinoma
Source: BMC Genomics. 2014 Mar 13;15(1):190. doi: 10.1186/1471-2164-15-190 (PMC4007559; doi:10.1186/1471-2164-15-190)
Supplement: Supplementary file 8 — Additional file 8: Contains the Gene Pattern gene set membership and enrichment values in an html format. The file index.html is the entry point. (ZIP 13 MB) [file 12864_2013_7028_MOESM8_ESM.zip › RODRIGUES_NTN1_TARGETS_DN.html]

Details for gene set RODRIGUES\_NTN1\_TARGETS\_DN[GSEA]

|  || Dataset | CT26\_gene\_expression |
| Phenotype | NoPhenotypeAvailable |
| Upregulated in class | na\_neg |
| GeneSet | RODRIGUES\_NTN1\_TARGETS\_DN |
| Enrichment Score (ES) | -0.2989498 |
| Normalized Enrichment Score (NES) | NaN |
| Nominal p-value | NaN |
| FDR q-value | 1.0 |
| FWER p-Value | 0.0 |
Table: GSEA Results Summary

  

Fig 1: Enrichment plot: RODRIGUES\_NTN1\_TARGETS\_DN      
 Profile of the Running ES Score & Positions of GeneSet Members on the Rank Ordered List

  

| PROBE | GENE SYMBOL | GENE\_TITLE | RANK IN GENE LIST | RANK METRIC SCORE | RUNNING ES | CORE ENRICHMENT || 1 | TAF15 |  |  | 30 | 39.000 | 0.0441 | No |
| 2 | NASP |  |  | 161 | 26.700 | 0.0673 | No |
| 3 | EREG |  |  | 604 | 17.300 | 0.0594 | No |
| 4 | MKLN1 |  |  | 610 | 17.300 | 0.0795 | No |
| 5 | AHNAK |  |  | 681 | 16.600 | 0.0946 | No |
| 6 | EPB41L2 |  |  | 715 | 16.200 | 0.1116 | No |
| 7 | HECTD1 |  |  | 756 | 15.900 | 0.1278 | No |
| 8 | VEGFA |  |  | 946 | 14.600 | 0.1329 | No |
| 9 | MET |  |  | 954 | 14.500 | 0.1496 | No |
| 10 | NFAT5 |  |  | 1118 | 13.400 | 0.1549 | No |
| 11 | MSLN |  |  | 1188 | 13.100 | 0.1660 | No |
| 12 | ARL4C |  |  | 1201 | 13.000 | 0.1806 | No |
| 13 | MED6 |  |  | 1230 | 12.900 | 0.1940 | No |
| 14 | FSCN1 |  |  | 1330 | 12.400 | 0.2023 | No |
| 15 | PACSIN2 |  |  | 1409 | 12.000 | 0.2114 | No |
| 16 | SPAG9 |  |  | 1489 | 11.700 | 0.2202 | No |
| 17 | CLOCK |  |  | 1533 | 11.500 | 0.2310 | No |
| 18 | PIP5K1A |  |  | 1706 | 10.800 | 0.2327 | No |
| 19 | NUMA1 |  |  | 1784 | 10.500 | 0.2402 | No |
| 20 | WNK1 |  |  | 1926 | 9.900 | 0.2428 | No |
| 21 | OGFR |  |  | 2192 | 9.100 | 0.2366 | No |
| 22 | BIN1 |  |  | 2194 | 9.100 | 0.2473 | No |
| 23 | CUL4A |  |  | 2323 | 8.700 | 0.2493 | No |
| 24 | FN1 |  |  | 2577 | 8.000 | 0.2426 | No |
| 25 | RBM5 |  |  | 2601 | 7.900 | 0.2504 | No |
| 26 | SFXN3 |  |  | 2606 | 7.900 | 0.2595 | No |
| 27 | HTATIP2 |  |  | 2685 | 7.700 | 0.2636 | No |
| 28 | PIK3CA |  |  | 3066 | 6.800 | 0.2473 | No |
| 29 | CNOT4 |  |  | 3116 | 6.600 | 0.2519 | No |
| 30 | MDM4 |  |  | 3390 | 6.000 | 0.2415 | No |
| 31 | FLNB |  |  | 3519 | 5.700 | 0.2400 | No |
| 32 | IER3 |  |  | 3561 | 5.600 | 0.2440 | No |
| 33 | STK17B |  |  | 3574 | 5.600 | 0.2499 | No |
| 34 | UBE2G2 |  |  | 3710 | 5.300 | 0.2475 | No |
| 35 | DGKD |  |  | 3804 | 5.100 | 0.2475 | No |
| 36 | DUSP6 |  |  | 3922 | 4.900 | 0.2458 | No |
| 37 | ENC1 |  |  | 4056 | 4.700 | 0.2428 | No |
| 38 | LIMK1 |  |  | 4065 | 4.700 | 0.2479 | No |
| 39 | RNF213 |  |  | 4073 | 4.700 | 0.2530 | No |
| 40 | ZNF175 |  |  | 4140 | 4.500 | 0.2541 | No |
| 41 | RRBP1 |  |  | 4243 | 4.400 | 0.2527 | No |
| 42 | ARID5B |  |  | 4378 | 4.100 | 0.2490 | No |
| 43 | RAPH1 |  |  | 4535 | 3.800 | 0.2435 | No |
| 44 | KLF13 |  |  | 4588 | 3.700 | 0.2445 | No |
| 45 | SIGIRR |  |  | 4590 | 3.700 | 0.2488 | No |
| 46 | DAPP1 |  |  | 4795 | 3.400 | 0.2397 | No |
| 47 | ARRB2 |  |  | 4803 | 3.400 | 0.2433 | No |
| 48 | SPEN |  |  | 4948 | 3.200 | 0.2379 | No |
| 49 | TNFRSF1B |  |  | 5066 | 2.900 | 0.2338 | No |
| 50 | CBL |  |  | 5703 | 2.000 | 0.1954 | No |
| 51 | ITGB5 |  |  | 5786 | 1.900 | 0.1924 | No |
| 52 | NR4A1 |  |  | 5818 | 1.900 | 0.1927 | No |
| 53 | TNNC1 |  |  | 5874 | 1.800 | 0.1913 | No |
| 54 | RBMS2 |  |  | 6071 | 1.500 | 0.1805 | No |
| 55 | ARHGAP26 |  |  | 6209 | 1.300 | 0.1732 | No |
| 56 | SHB |  |  | 6282 | 1.200 | 0.1700 | No |
| 57 | RIT1 |  |  | 6507 | 0.900 | 0.1567 | No |
| 58 | MAGI1 |  |  | 6755 | 0.600 | 0.1416 | No |
| 59 | PCDH1 |  |  | 6840 | 0.600 | 0.1370 | No |
| 60 | CTSE |  |  | 6917 | 0.500 | 0.1327 | No |
| 61 | BOLA1 |  |  | 6956 | 0.400 | 0.1307 | No |
| 62 | RAB27B |  |  | 7009 | 0.400 | 0.1279 | No |
| 63 | FBXO2 |  |  | 7311 | 0.100 | 0.1087 | No |
| 64 | KLK6 |  |  | 7890 | 0.000 | 0.0717 | No |
| 65 | PRLR |  |  | 10318 | -0.100 | -0.0837 | No |
| 66 | KRT13 |  |  | 10384 | -0.100 | -0.0877 | No |
| 67 | CYSLTR1 |  |  | 10497 | -0.100 | -0.0948 | No |
| 68 | TNFSF15 |  |  | 10559 | -0.100 | -0.0986 | No |
| 69 | POU3F1 |  |  | 10651 | -0.100 | -0.1043 | No |
| 70 | CACNA1I |  |  | 10776 | -0.100 | -0.1121 | No |
| 71 | FOLR1 |  |  | 10947 | -0.200 | -0.1228 | No |
| 72 | PGF |  |  | 11160 | -0.200 | -0.1361 | No |
| 73 | EVI2A |  |  | 11455 | -0.300 | -0.1546 | No |
| 74 | ADAMTS5 |  |  | 11465 | -0.400 | -0.1547 | No |
| 75 | IRF8 |  |  | 11497 | -0.400 | -0.1562 | No |
| 76 | PPL |  |  | 12065 | -0.700 | -0.1917 | No |
| 77 | EPOR |  |  | 12120 | -0.700 | -0.1943 | No |
| 78 | ADORA1 |  |  | 12252 | -0.800 | -0.2018 | No |
| 79 | PRSS3 |  |  | 12352 | -0.900 | -0.2071 | No |
| 80 | AREG |  |  | 12421 | -0.900 | -0.2104 | No |
| 81 | HSPG2 |  |  | 12492 | -1.000 | -0.2137 | No |
| 82 | SEZ6L2 |  |  | 12543 | -1.000 | -0.2157 | No |
| 83 | DDC |  |  | 12763 | -1.200 | -0.2283 | No |
| 84 | KLF2 |  |  | 12923 | -1.400 | -0.2368 | No |
| 85 | ZFPM1 |  |  | 13090 | -1.500 | -0.2457 | No |
| 86 | TNFRSF25 |  |  | 13143 | -1.600 | -0.2471 | No |
| 87 | HOXB5 |  |  | 13162 | -1.600 | -0.2464 | No |
| 88 | HOXB6 |  |  | 13168 | -1.600 | -0.2448 | No |
| 89 | GDA |  |  | 13171 | -1.600 | -0.2431 | No |
| 90 | MLXIP |  |  | 13376 | -1.900 | -0.2539 | No |
| 91 | CHRM1 |  |  | 13644 | -2.200 | -0.2684 | No |
| 92 | TGFBR2 |  |  | 14001 | -2.800 | -0.2879 | No |
| 93 | NAGLU |  |  | 14005 | -2.800 | -0.2848 | No |
| 94 | KLK1 |  |  | 14044 | -2.900 | -0.2838 | No |
| 95 | GAA |  |  | 14061 | -2.900 | -0.2814 | No |
| 96 | SPG7 |  |  | 14135 | -3.000 | -0.2825 | No |
| 97 | MINK1 |  |  | 14269 | -3.300 | -0.2872 | No |
| 98 | TSPAN8 |  |  | 14454 | -3.700 | -0.2946 | Yes |
| 99 | SOX9 |  |  | 14468 | -3.800 | -0.2909 | Yes |
| 100 | CDX2 |  |  | 14485 | -3.800 | -0.2875 | Yes |
| 101 | PXN |  |  | 14509 | -3.800 | -0.2845 | Yes |
| 102 | CEBPA |  |  | 14543 | -3.900 | -0.2820 | Yes |
| 103 | IFNGR1 |  |  | 14640 | -4.200 | -0.2832 | Yes |
| 104 | ASCL2 |  |  | 14755 | -4.500 | -0.2852 | Yes |
| 105 | TIMP3 |  |  | 14808 | -4.600 | -0.2831 | Yes |
| 106 | KIAA0182 |  |  | 14869 | -4.800 | -0.2812 | Yes |
| 107 | CLDN2 |  |  | 14905 | -4.900 | -0.2777 | Yes |
| 108 | SOX13 |  |  | 14934 | -5.000 | -0.2736 | Yes |
| 109 | RHOF |  |  | 14951 | -5.000 | -0.2687 | Yes |
| 110 | MYLK |  |  | 14958 | -5.100 | -0.2631 | Yes |
| 111 | KIFC2 |  |  | 14999 | -5.200 | -0.2595 | Yes |
| 112 | PIP5K1B |  |  | 15043 | -5.400 | -0.2559 | Yes |
| 113 | DMPK |  |  | 15082 | -5.600 | -0.2517 | Yes |
| 114 | SEMA4G |  |  | 15111 | -5.800 | -0.2467 | Yes |
| 115 | MYH14 |  |  | 15164 | -6.000 | -0.2429 | Yes |
| 116 | CANT1 |  |  | 15165 | -6.000 | -0.2358 | Yes |
| 117 | KCNQ1 |  |  | 15178 | -6.000 | -0.2295 | Yes |
| 118 | VIL1 |  |  | 15205 | -6.200 | -0.2239 | Yes |
| 119 | PVRL2 |  |  | 15207 | -6.200 | -0.2166 | Yes |
| 120 | MMP15 |  |  | 15214 | -6.300 | -0.2096 | Yes |
| 121 | ITGB4 |  |  | 15227 | -6.300 | -0.2029 | Yes |
| 122 | ITGA3 |  |  | 15237 | -6.400 | -0.1959 | Yes |
| 123 | VASP |  |  | 15281 | -6.600 | -0.1909 | Yes |
| 124 | ID1 |  |  | 15303 | -6.800 | -0.1842 | Yes |
| 125 | CLDN4 |  |  | 15314 | -6.800 | -0.1768 | Yes |
| 126 | TNS4 |  |  | 15326 | -6.900 | -0.1694 | Yes |
| 127 | CLDN3 |  |  | 15328 | -6.900 | -0.1613 | Yes |
| 128 | TNFRSF21 |  |  | 15339 | -7.000 | -0.1537 | Yes |
| 129 | USH1C |  |  | 15367 | -7.200 | -0.1469 | Yes |
| 130 | VDR |  |  | 15397 | -7.500 | -0.1399 | Yes |
| 131 | PRSS8 |  |  | 15441 | -7.900 | -0.1334 | Yes |
| 132 | PAPSS2 |  |  | 15445 | -7.900 | -0.1242 | Yes |
| 133 | BCAM |  |  | 15506 | -8.700 | -0.1178 | Yes |
| 134 | MYO1A |  |  | 15511 | -8.700 | -0.1078 | Yes |
| 135 | KRT18 |  |  | 15678 | -13.100 | -0.1030 | Yes |
| 136 | SULT1A1 |  |  | 15697 | -14.900 | -0.0865 | Yes |
| 137 | LGALS4 |  |  | 15713 | -16.500 | -0.0680 | Yes |
| 138 | HLA-DRB1 |  |  | 15738 | -25.000 | -0.0401 | Yes |
| 139 | RAB25 |  |  | 15748 | -34.500 | 0.0001 | Yes |
Table: GSEA details [plain text format]

  

Fig 2: RODRIGUES\_NTN1\_TARGETS\_DN: Random ES distribution      
 Gene set null distribution of ES for **RODRIGUES\_NTN1\_TARGETS\_DN**

  
