# Supplementary material for: Immunomic, genomic and transcriptomic characterization of CT26 colorectal carcinoma
Source: BMC Genomics. 2014 Mar 13;15(1):190. doi: 10.1186/1471-2164-15-190 (PMC4007559; doi:10.1186/1471-2164-15-190)
Supplement: Supplementary file 8 — Additional file 8: Contains the Gene Pattern gene set membership and enrichment values in an html format. The file index.html is the entry point. (ZIP 13 MB) [file 12864_2013_7028_MOESM8_ESM.zip › SENGUPTA_NASOPHARYNGEAL_CARCINOMA_DN.html]

Details for gene set SENGUPTA\_NASOPHARYNGEAL\_CARCINOMA\_DN[GSEA]

|  || Dataset | CT26\_gene\_expression |
| Phenotype | NoPhenotypeAvailable |
| Upregulated in class | na\_neg |
| GeneSet | SENGUPTA\_NASOPHARYNGEAL\_CARCINOMA\_DN |
| Enrichment Score (ES) | -0.46197295 |
| Normalized Enrichment Score (NES) | NaN |
| Nominal p-value | NaN |
| FDR q-value | 1.0 |
| FWER p-Value | 0.0 |
Table: GSEA Results Summary

  

Fig 1: Enrichment plot: SENGUPTA\_NASOPHARYNGEAL\_CARCINOMA\_DN      
 Profile of the Running ES Score & Positions of GeneSet Members on the Rank Ordered List

  

| PROBE | GENE SYMBOL | GENE\_TITLE | RANK IN GENE LIST | RANK METRIC SCORE | RUNNING ES | CORE ENRICHMENT || 1 | CDH26 |  |  | 20 | 42.800 | 0.0534 | No |
| 2 | LOC643037 |  |  | 102 | 30.000 | 0.0866 | No |
| 3 | CETN2 |  |  | 468 | 18.800 | 0.0871 | No |
| 4 | PRDX1 |  |  | 548 | 18.000 | 0.1050 | No |
| 5 | POR |  |  | 1019 | 14.100 | 0.0927 | No |
| 6 | SERPINB6 |  |  | 1101 | 13.600 | 0.1049 | No |
| 7 | CSTB |  |  | 1684 | 10.900 | 0.0813 | No |
| 8 | TUBA1A |  |  | 1792 | 10.500 | 0.0878 | No |
| 9 | AKR1C3 |  |  | 2269 | 8.900 | 0.0685 | No |
| 10 | LRP11 |  |  | 2474 | 8.300 | 0.0659 | No |
| 11 | TRAF3IP1 |  |  | 2516 | 8.100 | 0.0737 | No |
| 12 | IFT57 |  |  | 3271 | 6.300 | 0.0331 | No |
| 13 | TACC2 |  |  | 3465 | 5.800 | 0.0281 | No |
| 14 | DNAH9 |  |  | 3564 | 5.600 | 0.0289 | No |
| 15 | AHNAK2 |  |  | 3900 | 4.900 | 0.0136 | No |
| 16 | PRDX5 |  |  | 4096 | 4.600 | 0.0069 | No |
| 17 | SLC22A4 |  |  | 4337 | 4.200 | -0.0032 | No |
| 18 | TTC9 |  |  | 4452 | 4.000 | -0.0055 | No |
| 19 | LZTFL1 |  |  | 4539 | 3.800 | -0.0061 | No |
| 20 | PSENEN |  |  | 4625 | 3.700 | -0.0069 | No |
| 21 | SPAG1 |  |  | 4913 | 3.200 | -0.0213 | No |
| 22 | TRIM7 |  |  | 5030 | 3.000 | -0.0249 | No |
| 23 | ADSSL1 |  |  | 5105 | 2.900 | -0.0260 | No |
| 24 | MORN2 |  |  | 5390 | 2.400 | -0.0413 | No |
| 25 | PIAS3 |  |  | 5552 | 2.200 | -0.0488 | No |
| 26 | NBEA |  |  | 5791 | 1.900 | -0.0617 | No |
| 27 | IFT172 |  |  | 5889 | 1.800 | -0.0657 | No |
| 28 | WDR54 |  |  | 5930 | 1.700 | -0.0661 | No |
| 29 | TEX9 |  |  | 5960 | 1.700 | -0.0658 | No |
| 30 | IQCG |  |  | 5975 | 1.600 | -0.0646 | No |
| 31 | SPA17 |  |  | 6012 | 1.600 | -0.0649 | No |
| 32 | WDR66 |  |  | 6124 | 1.500 | -0.0702 | No |
| 33 | EFCAB2 |  |  | 6145 | 1.400 | -0.0697 | No |
| 34 | DNAL1 |  |  | 6184 | 1.400 | -0.0703 | No |
| 35 | GCLM |  |  | 6379 | 1.100 | -0.0814 | No |
| 36 | CCDC114 |  |  | 6435 | 1.000 | -0.0837 | No |
| 37 | ROPN1L |  |  | 6451 | 1.000 | -0.0834 | No |
| 38 | LRRC48 |  |  | 6741 | 0.700 | -0.1011 | No |
| 39 | PIH1D2 |  |  | 6784 | 0.600 | -0.1031 | No |
| 40 | TSGA10 |  |  | 6882 | 0.500 | -0.1087 | No |
| 41 | RIBC1 |  |  | 6883 | 0.500 | -0.1080 | No |
| 42 | SPAG17 |  |  | 6924 | 0.500 | -0.1100 | No |
| 43 | SERPINB7 |  |  | 6960 | 0.400 | -0.1117 | No |
| 44 | BANK1 |  |  | 7019 | 0.400 | -0.1149 | No |
| 45 | DNALI1 |  |  | 7067 | 0.300 | -0.1176 | No |
| 46 | DNAI2 |  |  | 7139 | 0.300 | -0.1218 | No |
| 47 | CCDC96 |  |  | 7151 | 0.300 | -0.1221 | No |
| 48 | CCDC65 |  |  | 7155 | 0.300 | -0.1219 | No |
| 49 | VTCN1 |  |  | 7190 | 0.200 | -0.1239 | No |
| 50 | IMPA2 |  |  | 7264 | 0.200 | -0.1283 | No |
| 51 | ZMYND10 |  |  | 7298 | 0.100 | -0.1303 | No |
| 52 | WDR78 |  |  | 7334 | 0.100 | -0.1324 | No |
| 53 | TEKT2 |  |  | 7394 | 0.100 | -0.1361 | No |
| 54 | PACRG |  |  | 7423 | 0.100 | -0.1378 | No |
| 55 | TMEM146 |  |  | 7489 | 0.100 | -0.1419 | No |
| 56 | LRRC34 |  |  | 7505 | 0.100 | -0.1427 | No |
| 57 | SPATA17 |  |  | 7543 | 0.000 | -0.1451 | No |
| 58 | AKAP14 |  |  | 7618 | 0.000 | -0.1499 | No |
| 59 | CCDC11 |  |  | 7649 | 0.000 | -0.1518 | No |
| 60 | YSK4 |  |  | 7753 | 0.000 | -0.1584 | No |
| 61 | HYDIN |  |  | 7882 | 0.000 | -0.1667 | No |
| 62 | TPPP3 |  |  | 7928 | 0.000 | -0.1696 | No |
| 63 | ABCA13 |  |  | 7934 | 0.000 | -0.1699 | No |
| 64 | CCDC81 |  |  | 8026 | 0.000 | -0.1758 | No |
| 65 | CHST9 |  |  | 8160 | 0.000 | -0.1843 | No |
| 66 | FBXO15 |  |  | 8254 | 0.000 | -0.1903 | No |
| 67 | MDH1B |  |  | 8454 | 0.000 | -0.2032 | No |
| 68 | MSMB |  |  | 8467 | 0.000 | -0.2039 | No |
| 69 | SERPINB3 |  |  | 8825 | 0.000 | -0.2270 | No |
| 70 | SLC22A16 |  |  | 8831 | 0.000 | -0.2273 | No |
| 71 | SPATA18 |  |  | 8850 | 0.000 | -0.2284 | No |
| 72 | TCL1A |  |  | 8899 | 0.000 | -0.2315 | No |
| 73 | TEKT1 |  |  | 8901 | 0.000 | -0.2316 | No |
| 74 | TTC29 |  |  | 8924 | 0.000 | -0.2330 | No |
| 75 | MORN3 |  |  | 8987 | 0.000 | -0.2370 | No |
| 76 | WDR63 |  |  | 9064 | 0.000 | -0.2419 | No |
| 77 | ARMC4 |  |  | 9083 | 0.000 | -0.2431 | No |
| 78 | KRT4 |  |  | 9219 | 0.000 | -0.2518 | No |
| 79 | EFCAB1 |  |  | 9225 | 0.000 | -0.2521 | No |
| 80 | NEK11 |  |  | 9226 | 0.000 | -0.2521 | No |
| 81 | RIBC2 |  |  | 9230 | 0.000 | -0.2523 | No |
| 82 | KCNE1 |  |  | 9294 | 0.000 | -0.2564 | No |
| 83 | SPINLW1 |  |  | 9336 | 0.000 | -0.2590 | No |
| 84 | DYX1C1 |  |  | 9425 | 0.000 | -0.2647 | No |
| 85 | AK7 |  |  | 9441 | 0.000 | -0.2657 | No |
| 86 | TTC21A |  |  | 9450 | 0.000 | -0.2662 | No |
| 87 | IL20RA |  |  | 9465 | 0.000 | -0.2671 | No |
| 88 | KCNRG |  |  | 9511 | 0.000 | -0.2700 | No |
| 89 | EFHB |  |  | 9567 | 0.000 | -0.2735 | No |
| 90 | LTF |  |  | 9647 | 0.000 | -0.2786 | No |
| 91 | CASC1 |  |  | 9673 | 0.000 | -0.2802 | No |
| 92 | WDR69 |  |  | 9684 | 0.000 | -0.2809 | No |
| 93 | CLDN10 |  |  | 9719 | 0.000 | -0.2831 | No |
| 94 | DYNLRB2 |  |  | 9720 | 0.000 | -0.2831 | No |
| 95 | CCDC113 |  |  | 9737 | 0.000 | -0.2841 | No |
| 96 | ARMC2 |  |  | 9742 | 0.000 | -0.2843 | No |
| 97 | TCTEX1D1 |  |  | 9850 | 0.000 | -0.2912 | No |
| 98 | STOML3 |  |  | 10009 | 0.000 | -0.3014 | No |
| 99 | CR2 |  |  | 10131 | -0.100 | -0.3091 | No |
| 100 | ARMC3 |  |  | 10149 | -0.100 | -0.3101 | No |
| 101 | SPAG6 |  |  | 10175 | -0.100 | -0.3116 | No |
| 102 | DNAH5 |  |  | 10193 | -0.100 | -0.3125 | No |
| 103 | MUC16 |  |  | 10199 | -0.100 | -0.3127 | No |
| 104 | MS4A1 |  |  | 10206 | -0.100 | -0.3130 | No |
| 105 | S100A9 |  |  | 10244 | -0.100 | -0.3152 | No |
| 106 | FANK1 |  |  | 10276 | -0.100 | -0.3171 | No |
| 107 | DNAH2 |  |  | 10278 | -0.100 | -0.3170 | No |
| 108 | TTC25 |  |  | 10295 | -0.100 | -0.3180 | No |
| 109 | TTC12 |  |  | 10379 | -0.100 | -0.3232 | No |
| 110 | AQP3 |  |  | 10500 | -0.100 | -0.3308 | No |
| 111 | ALDH3B1 |  |  | 10515 | -0.100 | -0.3316 | No |
| 112 | KLF8 |  |  | 10521 | -0.100 | -0.3318 | No |
| 113 | MUC20 |  |  | 10606 | -0.100 | -0.3370 | No |
| 114 | GPR110 |  |  | 10625 | -0.100 | -0.3381 | No |
| 115 | TNFSF11 |  |  | 10785 | -0.100 | -0.3482 | No |
| 116 | EFHC1 |  |  | 10846 | -0.200 | -0.3518 | No |
| 117 | TMEM154 |  |  | 10972 | -0.200 | -0.3596 | No |
| 118 | FOXJ1 |  |  | 11065 | -0.200 | -0.3653 | No |
| 119 | SCGB1A1 |  |  | 11105 | -0.200 | -0.3675 | No |
| 120 | ANKRD35 |  |  | 11209 | -0.300 | -0.3738 | No |
| 121 | DUOX1 |  |  | 11260 | -0.300 | -0.3766 | No |
| 122 | SYTL4 |  |  | 11333 | -0.300 | -0.3809 | No |
| 123 | CAPSL |  |  | 11343 | -0.300 | -0.3811 | No |
| 124 | AGBL2 |  |  | 11380 | -0.300 | -0.3830 | No |
| 125 | ZMYND12 |  |  | 11439 | -0.300 | -0.3864 | No |
| 126 | KRT14 |  |  | 11559 | -0.400 | -0.3936 | No |
| 127 | ATP10B |  |  | 11566 | -0.400 | -0.3934 | No |
| 128 | ALOX15 |  |  | 11576 | -0.400 | -0.3935 | No |
| 129 | WDR16 |  |  | 11612 | -0.400 | -0.3952 | No |
| 130 | FCRLA |  |  | 11615 | -0.400 | -0.3949 | No |
| 131 | CD55 |  |  | 11649 | -0.400 | -0.3965 | No |
| 132 | DMKN |  |  | 11665 | -0.500 | -0.3968 | No |
| 133 | TMEM40 |  |  | 11696 | -0.500 | -0.3981 | No |
| 134 | IQCD |  |  | 11711 | -0.500 | -0.3984 | No |
| 135 | PPIL6 |  |  | 11804 | -0.500 | -0.4037 | No |
| 136 | UPK1B |  |  | 11851 | -0.600 | -0.4059 | No |
| 137 | CHST6 |  |  | 11968 | -0.600 | -0.4126 | No |
| 138 | GSTA3 |  |  | 12030 | -0.700 | -0.4156 | No |
| 139 | TSPAN6 |  |  | 12064 | -0.700 | -0.4168 | No |
| 140 | RHOV |  |  | 12087 | -0.700 | -0.4174 | No |
| 141 | PAX5 |  |  | 12277 | -0.800 | -0.4285 | No |
| 142 | CCDC19 |  |  | 12289 | -0.800 | -0.4282 | No |
| 143 | COBL |  |  | 12291 | -0.800 | -0.4273 | No |
| 144 | LCN2 |  |  | 12320 | -0.900 | -0.4279 | No |
| 145 | ST6GALNAC1 |  |  | 12375 | -0.900 | -0.4302 | No |
| 146 | FMO5 |  |  | 12434 | -0.900 | -0.4328 | No |
| 147 | FAM131A |  |  | 12450 | -1.000 | -0.4325 | No |
| 148 | ANKRD37 |  |  | 12473 | -1.000 | -0.4327 | No |
| 149 | SIX2 |  |  | 12479 | -1.000 | -0.4317 | No |
| 150 | RBM24 |  |  | 12488 | -1.000 | -0.4309 | No |
| 151 | DNAI1 |  |  | 12504 | -1.000 | -0.4306 | No |
| 152 | BNIPL |  |  | 12614 | -1.100 | -0.4362 | No |
| 153 | KATNB1 |  |  | 12658 | -1.100 | -0.4376 | No |
| 154 | CD19 |  |  | 12861 | -1.300 | -0.4490 | No |
| 155 | DNAJA4 |  |  | 12970 | -1.400 | -0.4542 | No |
| 156 | DNER |  |  | 13021 | -1.500 | -0.4555 | No |
| 157 | PLCE1 |  |  | 13069 | -1.500 | -0.4566 | No |
| 158 | NMU |  |  | 13073 | -1.500 | -0.4548 | No |
| 159 | ASS1 |  |  | 13085 | -1.500 | -0.4536 | No |
| 160 | SLC27A2 |  |  | 13127 | -1.600 | -0.4542 | No |
| 161 | MT1E |  |  | 13193 | -1.700 | -0.4563 | No |
| 162 | VPREB3 |  |  | 13208 | -1.700 | -0.4550 | No |
| 163 | CCDC17 |  |  | 13261 | -1.700 | -0.4562 | No |
| 164 | SYNGR1 |  |  | 13320 | -1.800 | -0.4576 | No |
| 165 | VILL |  |  | 13365 | -1.800 | -0.4581 | No |
| 166 | CLIC3 |  |  | 13379 | -1.900 | -0.4565 | No |
| 167 | PCP4L1 |  |  | 13391 | -1.900 | -0.4548 | No |
| 168 | BASP1 |  |  | 13424 | -1.900 | -0.4545 | No |
| 169 | SLPI |  |  | 13439 | -1.900 | -0.4529 | No |
| 170 | FKBP1B |  |  | 13440 | -1.900 | -0.4505 | No |
| 171 | RRAD |  |  | 13458 | -2.000 | -0.4490 | No |
| 172 | TRAK1 |  |  | 13490 | -2.000 | -0.4485 | No |
| 173 | PTPRN2 |  |  | 13502 | -2.000 | -0.4466 | No |
| 174 | CYP4B1 |  |  | 13578 | -2.100 | -0.4488 | No |
| 175 | WDR13 |  |  | 13605 | -2.200 | -0.4477 | No |
| 176 | VPS37B |  |  | 13613 | -2.200 | -0.4453 | No |
| 177 | SORBS2 |  |  | 13622 | -2.200 | -0.4430 | No |
| 178 | ABHD14B |  |  | 13707 | -2.300 | -0.4455 | No |
| 179 | TOX3 |  |  | 13964 | -2.700 | -0.4585 | Yes |
| 180 | PRR15 |  |  | 13978 | -2.700 | -0.4559 | Yes |
| 181 | NQO1 |  |  | 14021 | -2.800 | -0.4550 | Yes |
| 182 | MUC1 |  |  | 14055 | -2.900 | -0.4535 | Yes |
| 183 | ABLIM1 |  |  | 14134 | -3.000 | -0.4546 | Yes |
| 184 | CLIC6 |  |  | 14154 | -3.000 | -0.4520 | Yes |
| 185 | POU2AF1 |  |  | 14181 | -3.100 | -0.4498 | Yes |
| 186 | TUBB3 |  |  | 14360 | -3.500 | -0.4568 | Yes |
| 187 | MUC13 |  |  | 14428 | -3.700 | -0.4563 | Yes |
| 188 | TCEA3 |  |  | 14429 | -3.700 | -0.4516 | Yes |
| 189 | LRG1 |  |  | 14477 | -3.800 | -0.4498 | Yes |
| 190 | ALDH3B2 |  |  | 14512 | -3.900 | -0.4470 | Yes |
| 191 | FUT2 |  |  | 14563 | -4.000 | -0.4451 | Yes |
| 192 | ZFYVE21 |  |  | 14634 | -4.200 | -0.4442 | Yes |
| 193 | MUC4 |  |  | 14642 | -4.200 | -0.4393 | Yes |
| 194 | SLC16A5 |  |  | 14661 | -4.200 | -0.4351 | Yes |
| 195 | MGLL |  |  | 14742 | -4.400 | -0.4347 | Yes |
| 196 | MYO1D |  |  | 14801 | -4.600 | -0.4325 | Yes |
| 197 | TUBB2A |  |  | 14824 | -4.700 | -0.4279 | Yes |
| 198 | CGN |  |  | 14839 | -4.700 | -0.4228 | Yes |
| 199 | MFSD4 |  |  | 14851 | -4.700 | -0.4175 | Yes |
| 200 | CXXC5 |  |  | 14879 | -4.800 | -0.4131 | Yes |
| 201 | SLC2A10 |  |  | 14899 | -4.900 | -0.4081 | Yes |
| 202 | AGR2 |  |  | 14906 | -4.900 | -0.4022 | Yes |
| 203 | DHCR24 |  |  | 14915 | -4.900 | -0.3965 | Yes |
| 204 | TMC5 |  |  | 14928 | -5.000 | -0.3908 | Yes |
| 205 | TMC4 |  |  | 14959 | -5.100 | -0.3862 | Yes |
| 206 | MS4A8B |  |  | 14975 | -5.100 | -0.3807 | Yes |
| 207 | TMEM45B |  |  | 14978 | -5.100 | -0.3743 | Yes |
| 208 | FAM3B |  |  | 15002 | -5.200 | -0.3691 | Yes |
| 209 | ANXA11 |  |  | 15026 | -5.300 | -0.3638 | Yes |
| 210 | CRYL1 |  |  | 15059 | -5.500 | -0.3589 | Yes |
| 211 | SLC44A4 |  |  | 15093 | -5.600 | -0.3538 | Yes |
| 212 | PROM1 |  |  | 15131 | -5.900 | -0.3487 | Yes |
| 213 | CKB |  |  | 15137 | -5.900 | -0.3415 | Yes |
| 214 | CFB |  |  | 15148 | -5.900 | -0.3346 | Yes |
| 215 | TMPRSS4 |  |  | 15222 | -6.300 | -0.3312 | Yes |
| 216 | CLDN23 |  |  | 15239 | -6.400 | -0.3241 | Yes |
| 217 | PRSS23 |  |  | 15264 | -6.500 | -0.3173 | Yes |
| 218 | HIST2H2BE |  |  | 15275 | -6.600 | -0.3095 | Yes |
| 219 | TSPAN1 |  |  | 15280 | -6.600 | -0.3013 | Yes |
| 220 | TNFRSF21 |  |  | 15339 | -7.000 | -0.2961 | Yes |
| 221 | CAPN5 |  |  | 15369 | -7.200 | -0.2888 | Yes |
| 222 | B3GNT7 |  |  | 15376 | -7.300 | -0.2798 | Yes |
| 223 | ADH1C |  |  | 15410 | -7.600 | -0.2723 | Yes |
| 224 | TFF3 |  |  | 15423 | -7.700 | -0.2632 | Yes |
| 225 | TJP3 |  |  | 15458 | -8.100 | -0.2550 | Yes |
| 226 | ATP12A |  |  | 15479 | -8.300 | -0.2457 | Yes |
| 227 | CAPN9 |  |  | 15493 | -8.400 | -0.2358 | Yes |
| 228 | BACE2 |  |  | 15495 | -8.500 | -0.2250 | Yes |
| 229 | TPPP |  |  | 15587 | -9.900 | -0.2182 | Yes |
| 230 | EPPK1 |  |  | 15597 | -10.100 | -0.2059 | Yes |
| 231 | CIB1 |  |  | 15635 | -11.400 | -0.1937 | Yes |
| 232 | FAM3D |  |  | 15690 | -14.200 | -0.1790 | Yes |
| 233 | CLDN7 |  |  | 15700 | -15.300 | -0.1600 | Yes |
| 234 | WFDC2 |  |  | 15702 | -15.400 | -0.1404 | Yes |
| 235 | PIGR |  |  | 15703 | -15.500 | -0.1206 | Yes |
| 236 | SELENBP1 |  |  | 15707 | -15.900 | -0.1005 | Yes |
| 237 | ELF3 |  |  | 15708 | -16.000 | -0.0800 | Yes |
| 238 | ALDH1L1 |  |  | 15711 | -16.100 | -0.0596 | Yes |
| 239 | PDZK1IP1 |  |  | 15737 | -22.300 | -0.0327 | Yes |
| 240 | KRT7 |  |  | 15742 | -26.100 | 0.0005 | Yes |
Table: GSEA details [plain text format]

  

Fig 2: SENGUPTA\_NASOPHARYNGEAL\_CARCINOMA\_DN: Random ES distribution      
 Gene set null distribution of ES for **SENGUPTA\_NASOPHARYNGEAL\_CARCINOMA\_DN**

  
