# Supplementary material for: Immunomic, genomic and transcriptomic characterization of CT26 colorectal carcinoma
Source: BMC Genomics. 2014 Mar 13;15(1):190. doi: 10.1186/1471-2164-15-190 (PMC4007559; doi:10.1186/1471-2164-15-190)
Supplement: Supplementary file 8 — Additional file 8: Contains the Gene Pattern gene set membership and enrichment values in an html format. The file index.html is the entry point. (ZIP 13 MB) [file 12864_2013_7028_MOESM8_ESM.zip › SOTIRIOU_BREAST_CANCER_GRADE_1_VS_3_UP.html]

Details for gene set SOTIRIOU\_BREAST\_CANCER\_GRADE\_1\_VS\_3\_UP[GSEA]

|  || Dataset | CT26\_gene\_expression |
| Phenotype | NoPhenotypeAvailable |
| Upregulated in class | na\_pos |
| GeneSet | SOTIRIOU\_BREAST\_CANCER\_GRADE\_1\_VS\_3\_UP |
| Enrichment Score (ES) | 0.7641118 |
| Normalized Enrichment Score (NES) | 1.6861377 |
| Nominal p-value | 0.0 |
| FDR q-value | 0.0017464295 |
| FWER p-Value | 0.033 |
Table: GSEA Results Summary

  

Fig 1: Enrichment plot: SOTIRIOU\_BREAST\_CANCER\_GRADE\_1\_VS\_3\_UP      
 Profile of the Running ES Score & Positions of GeneSet Members on the Rank Ordered List

  

| PROBE | GENE SYMBOL | GENE\_TITLE | RANK IN GENE LIST | RANK METRIC SCORE | RUNNING ES | CORE ENRICHMENT || 1 | TOP2A |  |  | 8 | 56.000 | 0.0287 | Yes |
| 2 | PSMD12 |  |  | 27 | 39.500 | 0.0481 | Yes |
| 3 | TCEB1 |  |  | 29 | 39.300 | 0.0686 | Yes |
| 4 | TPX2 |  |  | 39 | 37.700 | 0.0877 | Yes |
| 5 | KIF20A |  |  | 77 | 31.900 | 0.1019 | Yes |
| 6 | MCM4 |  |  | 81 | 31.500 | 0.1182 | Yes |
| 7 | ECT2 |  |  | 100 | 30.500 | 0.1329 | Yes |
| 8 | MCM6 |  |  | 163 | 26.600 | 0.1428 | Yes |
| 9 | UBE2S |  |  | 177 | 25.600 | 0.1553 | Yes |
| 10 | CCNA2 |  |  | 178 | 25.600 | 0.1687 | Yes |
| 11 | TOMM70A |  |  | 180 | 25.500 | 0.1819 | Yes |
| 12 | PRC1 |  |  | 181 | 25.500 | 0.1952 | Yes |
| 13 | KIF11 |  |  | 197 | 24.700 | 0.2071 | Yes |
| 14 | TMPO |  |  | 203 | 24.500 | 0.2196 | Yes |
| 15 | PTTG1 |  |  | 223 | 23.900 | 0.2309 | Yes |
| 16 | HMMR |  |  | 246 | 23.200 | 0.2415 | Yes |
| 17 | STIP1 |  |  | 278 | 22.300 | 0.2512 | Yes |
| 18 | CDC20 |  |  | 284 | 22.200 | 0.2624 | Yes |
| 19 | EZH2 |  |  | 286 | 22.000 | 0.2739 | Yes |
| 20 | MCM3 |  |  | 315 | 21.400 | 0.2832 | Yes |
| 21 | CCT5 |  |  | 321 | 21.200 | 0.2940 | Yes |
| 22 | FOXM1 |  |  | 322 | 21.200 | 0.3050 | Yes |
| 23 | NEK2 |  |  | 346 | 20.900 | 0.3144 | Yes |
| 24 | KPNA2 |  |  | 354 | 20.700 | 0.3248 | Yes |
| 25 | BUB1 |  |  | 360 | 20.600 | 0.3352 | Yes |
| 26 | XPOT |  |  | 365 | 20.500 | 0.3456 | Yes |
| 27 | SHMT2 |  |  | 375 | 20.300 | 0.3557 | Yes |
| 28 | CENPE |  |  | 391 | 20.100 | 0.3652 | Yes |
| 29 | CENPN |  |  | 403 | 19.900 | 0.3749 | Yes |
| 30 | MKI67 |  |  | 414 | 19.700 | 0.3845 | Yes |
| 31 | RAD51 |  |  | 427 | 19.400 | 0.3938 | Yes |
| 32 | CENPF |  |  | 431 | 19.400 | 0.4038 | Yes |
| 33 | NCAPH |  |  | 459 | 19.000 | 0.4119 | Yes |
| 34 | BIRC5 |  |  | 507 | 18.400 | 0.4185 | Yes |
| 35 | NCAPG |  |  | 524 | 18.200 | 0.4270 | Yes |
| 36 | NME1 |  |  | 540 | 18.100 | 0.4355 | Yes |
| 37 | SLC7A5 |  |  | 542 | 18.100 | 0.4449 | Yes |
| 38 | MAD2L1 |  |  | 559 | 17.900 | 0.4532 | Yes |
| 39 | ZWINT |  |  | 574 | 17.700 | 0.4615 | Yes |
| 40 | BUB1B |  |  | 609 | 17.300 | 0.4683 | Yes |
| 41 | KIF4A |  |  | 648 | 16.900 | 0.4747 | Yes |
| 42 | CEP55 |  |  | 662 | 16.800 | 0.4827 | Yes |
| 43 | GTPBP4 |  |  | 672 | 16.700 | 0.4908 | Yes |
| 44 | RACGAP1 |  |  | 682 | 16.600 | 0.4989 | Yes |
| 45 | FBXO5 |  |  | 688 | 16.500 | 0.5072 | Yes |
| 46 | NUSAP1 |  |  | 716 | 16.200 | 0.5139 | Yes |
| 47 | CCNE2 |  |  | 743 | 16.000 | 0.5206 | Yes |
| 48 | DNAJC9 |  |  | 763 | 15.900 | 0.5276 | Yes |
| 49 | PFDN6 |  |  | 764 | 15.800 | 0.5359 | Yes |
| 50 | NCAPG2 |  |  | 774 | 15.800 | 0.5435 | Yes |
| 51 | AURKA |  |  | 799 | 15.600 | 0.5501 | Yes |
| 52 | FAM64A |  |  | 841 | 15.300 | 0.5555 | Yes |
| 53 | KIFC1 |  |  | 870 | 15.100 | 0.5616 | Yes |
| 54 | NDC80 |  |  | 878 | 15.000 | 0.5689 | Yes |
| 55 | TACC3 |  |  | 910 | 14.800 | 0.5747 | Yes |
| 56 | SPAG5 |  |  | 927 | 14.700 | 0.5813 | Yes |
| 57 | UBE2N |  |  | 944 | 14.600 | 0.5879 | Yes |
| 58 | NUP93 |  |  | 951 | 14.500 | 0.5951 | Yes |
| 59 | H2AFZ |  |  | 982 | 14.300 | 0.6006 | Yes |
| 60 | GMPS |  |  | 994 | 14.200 | 0.6073 | Yes |
| 61 | CDCA8 |  |  | 1017 | 14.100 | 0.6133 | Yes |
| 62 | TROAP |  |  | 1028 | 14.000 | 0.6199 | Yes |
| 63 | KIF2C |  |  | 1036 | 14.000 | 0.6268 | Yes |
| 64 | FEN1 |  |  | 1052 | 13.900 | 0.6331 | Yes |
| 65 | MELK |  |  | 1077 | 13.700 | 0.6387 | Yes |
| 66 | RRM2 |  |  | 1084 | 13.700 | 0.6454 | Yes |
| 67 | CCNB2 |  |  | 1155 | 13.200 | 0.6478 | Yes |
| 68 | E2F1 |  |  | 1172 | 13.100 | 0.6536 | Yes |
| 69 | TRIP13 |  |  | 1182 | 13.100 | 0.6599 | Yes |
| 70 | E2F8 |  |  | 1210 | 13.000 | 0.6650 | Yes |
| 71 | MARS |  |  | 1244 | 12.800 | 0.6695 | Yes |
| 72 | CENPI |  |  | 1262 | 12.700 | 0.6751 | Yes |
| 73 | MCM10 |  |  | 1264 | 12.700 | 0.6816 | Yes |
| 74 | GTSE1 |  |  | 1272 | 12.700 | 0.6878 | Yes |
| 75 | MCM2 |  |  | 1281 | 12.700 | 0.6939 | Yes |
| 76 | NUTF2 |  |  | 1291 | 12.600 | 0.6999 | Yes |
| 77 | PSMA7 |  |  | 1308 | 12.500 | 0.7054 | Yes |
| 78 | H2AFV |  |  | 1310 | 12.500 | 0.7118 | Yes |
| 79 | SNRPC |  |  | 1339 | 12.300 | 0.7165 | Yes |
| 80 | TUBA1C |  |  | 1366 | 12.200 | 0.7212 | Yes |
| 81 | JMJD6 |  |  | 1400 | 12.100 | 0.7254 | Yes |
| 82 | TTK |  |  | 1430 | 11.900 | 0.7297 | Yes |
| 83 | ESPL1 |  |  | 1496 | 11.700 | 0.7316 | Yes |
| 84 | TXNRD1 |  |  | 1607 | 11.200 | 0.7304 | Yes |
| 85 | KIF15 |  |  | 1723 | 10.700 | 0.7287 | Yes |
| 86 | CENPA |  |  | 1762 | 10.600 | 0.7318 | Yes |
| 87 | LMNB1 |  |  | 1916 | 10.000 | 0.7272 | Yes |
| 88 | ASPM |  |  | 1918 | 10.000 | 0.7323 | Yes |
| 89 | DSN1 |  |  | 1919 | 10.000 | 0.7375 | Yes |
| 90 | CKS2 |  |  | 1959 | 9.800 | 0.7402 | Yes |
| 91 | TUBA1B |  |  | 1975 | 9.700 | 0.7442 | Yes |
| 92 | HMGB3 |  |  | 1996 | 9.700 | 0.7480 | Yes |
| 93 | MYBL2 |  |  | 2007 | 9.600 | 0.7524 | Yes |
| 94 | SNRPF |  |  | 2056 | 9.500 | 0.7543 | Yes |
| 95 | STMN1 |  |  | 2088 | 9.400 | 0.7572 | Yes |
| 96 | RFC4 |  |  | 2243 | 8.900 | 0.7520 | Yes |
| 97 | CCNB1 |  |  | 2266 | 8.900 | 0.7552 | Yes |
| 98 | RNASEH2A |  |  | 2408 | 8.500 | 0.7506 | Yes |
| 99 | HCCS |  |  | 2480 | 8.200 | 0.7503 | Yes |
| 100 | CDK2 |  |  | 2519 | 8.100 | 0.7521 | Yes |
| 101 | BYSL |  |  | 2559 | 8.000 | 0.7538 | Yes |
| 102 | CDCA3 |  |  | 2591 | 7.900 | 0.7559 | Yes |
| 103 | SNRPG |  |  | 2615 | 7.900 | 0.7586 | Yes |
| 104 | MRPL15 |  |  | 2818 | 7.400 | 0.7495 | Yes |
| 105 | AURKB |  |  | 2822 | 7.300 | 0.7531 | Yes |
| 106 | UBE2C |  |  | 2879 | 7.200 | 0.7533 | Yes |
| 107 | CDKN3 |  |  | 2917 | 7.100 | 0.7546 | Yes |
| 108 | COX7B |  |  | 2921 | 7.100 | 0.7581 | Yes |
| 109 | MLF1IP |  |  | 2974 | 7.000 | 0.7585 | Yes |
| 110 | GINS1 |  |  | 2999 | 6.900 | 0.7605 | Yes |
| 111 | TIMELESS |  |  | 3000 | 6.900 | 0.7641 | Yes |
| 112 | BLM |  |  | 3202 | 6.400 | 0.7546 | No |
| 113 | ORMDL2 |  |  | 3374 | 6.100 | 0.7468 | No |
| 114 | ITCH |  |  | 3397 | 6.000 | 0.7485 | No |
| 115 | PDSS1 |  |  | 3423 | 5.900 | 0.7500 | No |
| 116 | TIMM10 |  |  | 3578 | 5.600 | 0.7431 | No |
| 117 | CHEK1 |  |  | 3593 | 5.600 | 0.7451 | No |
| 118 | CDC25A |  |  | 3621 | 5.500 | 0.7462 | No |
| 119 | NUDT1 |  |  | 3655 | 5.400 | 0.7469 | No |
| 120 | VRK1 |  |  | 3707 | 5.300 | 0.7464 | No |
| 121 | POLR2K |  |  | 3717 | 5.300 | 0.7486 | No |
| 122 | RAD54B |  |  | 3981 | 4.800 | 0.7343 | No |
| 123 | EXO1 |  |  | 4074 | 4.700 | 0.7308 | No |
| 124 | KIF14 |  |  | 4209 | 4.400 | 0.7245 | No |
| 125 | LAGE3 |  |  | 4384 | 4.100 | 0.7155 | No |
| 126 | DONSON |  |  | 4580 | 3.800 | 0.7050 | No |
| 127 | OIP5 |  |  | 4605 | 3.700 | 0.7054 | No |
| 128 | STIL |  |  | 4898 | 3.200 | 0.6884 | No |
| 129 | TXN |  |  | 5081 | 2.900 | 0.6782 | No |
| 130 | POLQ |  |  | 5337 | 2.500 | 0.6632 | No |
| 131 | GPR172A |  |  | 6100 | 1.500 | 0.6152 | No |
| 132 | PLK1 |  |  | 6602 | 0.800 | 0.5835 | No |
| 133 | QPRT |  |  | 11064 | -0.200 | 0.2979 | No |
| 134 | PKMYT1 |  |  | 11212 | -0.300 | 0.2887 | No |
| 135 | MRPL12 |  |  | 12019 | -0.700 | 0.2374 | No |
| 136 | HN1 |  |  | 13980 | -2.700 | 0.1133 | No |
Table: GSEA details [plain text format]

  

Fig 2: SOTIRIOU\_BREAST\_CANCER\_GRADE\_1\_VS\_3\_UP: Random ES distribution      
 Gene set null distribution of ES for **SOTIRIOU\_BREAST\_CANCER\_GRADE\_1\_VS\_3\_UP**

  
