# Supplementary material for: Immunomic, genomic and transcriptomic characterization of CT26 colorectal carcinoma
Source: BMC Genomics. 2014 Mar 13;15(1):190. doi: 10.1186/1471-2164-15-190 (PMC4007559; doi:10.1186/1471-2164-15-190)
Supplement: Supplementary file 8 — Additional file 8: Contains the Gene Pattern gene set membership and enrichment values in an html format. The file index.html is the entry point. (ZIP 13 MB) [file 12864_2013_7028_MOESM8_ESM.zip › TANG_SENESCENCE_TP53_TARGETS_DN.html]

Details for gene set TANG\_SENESCENCE\_TP53\_TARGETS\_DN[GSEA]

|  || Dataset | CT26\_gene\_expression |
| Phenotype | NoPhenotypeAvailable |
| Upregulated in class | na\_pos |
| GeneSet | TANG\_SENESCENCE\_TP53\_TARGETS\_DN |
| Enrichment Score (ES) | 0.7899835 |
| Normalized Enrichment Score (NES) | 1.6858315 |
| Nominal p-value | 0.0 |
| FDR q-value | 0.0016994763 |
| FWER p-Value | 0.034 |
Table: GSEA Results Summary

  

Fig 1: Enrichment plot: TANG\_SENESCENCE\_TP53\_TARGETS\_DN      
 Profile of the Running ES Score & Positions of GeneSet Members on the Rank Ordered List

  

| PROBE | GENE SYMBOL | GENE\_TITLE | RANK IN GENE LIST | RANK METRIC SCORE | RUNNING ES | CORE ENRICHMENT || 1 | SMC4 |  |  | 1 | 76.300 | 0.1018 | Yes |
| 2 | TOP2A |  |  | 8 | 56.000 | 0.1763 | Yes |
| 3 | TPX2 |  |  | 39 | 37.700 | 0.2247 | Yes |
| 4 | KIF20A |  |  | 77 | 31.900 | 0.2650 | Yes |
| 5 | CCNA2 |  |  | 178 | 25.600 | 0.2928 | Yes |
| 6 | PRC1 |  |  | 181 | 25.500 | 0.3267 | Yes |
| 7 | KIF11 |  |  | 197 | 24.700 | 0.3587 | Yes |
| 8 | PTTG1 |  |  | 223 | 23.900 | 0.3891 | Yes |
| 9 | CDC20 |  |  | 284 | 22.200 | 0.4149 | Yes |
| 10 | EZH2 |  |  | 286 | 22.000 | 0.4442 | Yes |
| 11 | KIF23 |  |  | 337 | 21.000 | 0.4691 | Yes |
| 12 | NEK2 |  |  | 346 | 20.900 | 0.4965 | Yes |
| 13 | BUB1 |  |  | 360 | 20.600 | 0.5232 | Yes |
| 14 | DBF4 |  |  | 369 | 20.400 | 0.5499 | Yes |
| 15 | CENPE |  |  | 391 | 20.100 | 0.5754 | Yes |
| 16 | BIRC5 |  |  | 507 | 18.400 | 0.5927 | Yes |
| 17 | NCAPG |  |  | 524 | 18.200 | 0.6160 | Yes |
| 18 | MRE11A |  |  | 633 | 17.000 | 0.6318 | Yes |
| 19 | KIF4A |  |  | 648 | 16.900 | 0.6535 | Yes |
| 20 | RACGAP1 |  |  | 682 | 16.600 | 0.6735 | Yes |
| 21 | FBXO5 |  |  | 688 | 16.500 | 0.6953 | Yes |
| 22 | NUSAP1 |  |  | 716 | 16.200 | 0.7152 | Yes |
| 23 | PBK |  |  | 874 | 15.000 | 0.7252 | Yes |
| 24 | EXOSC9 |  |  | 888 | 14.900 | 0.7443 | Yes |
| 25 | SPAG5 |  |  | 927 | 14.700 | 0.7615 | Yes |
| 26 | CCNB2 |  |  | 1155 | 13.200 | 0.7647 | Yes |
| 27 | TTK |  |  | 1430 | 11.900 | 0.7631 | Yes |
| 28 | TCF4 |  |  | 1555 | 11.400 | 0.7704 | Yes |
| 29 | PLK4 |  |  | 1642 | 11.100 | 0.7798 | Yes |
| 30 | CENPA |  |  | 1762 | 10.600 | 0.7864 | Yes |
| 31 | LMNB1 |  |  | 1916 | 10.000 | 0.7900 | Yes |
| 32 | MAP1B |  |  | 2436 | 8.400 | 0.7681 | No |
| 33 | TFAM |  |  | 2472 | 8.300 | 0.7770 | No |
| 34 | TK1 |  |  | 2762 | 7.500 | 0.7686 | No |
| 35 | AURKB |  |  | 2822 | 7.300 | 0.7746 | No |
| 36 | UBE2C |  |  | 2879 | 7.200 | 0.7807 | No |
| 37 | CDC25A |  |  | 3621 | 5.500 | 0.7408 | No |
| 38 | BARD1 |  |  | 3765 | 5.200 | 0.7386 | No |
| 39 | NUP62 |  |  | 6237 | 1.300 | 0.5830 | No |
| 40 | IL12RB2 |  |  | 7425 | 0.100 | 0.5075 | No |
| 41 | ADARB1 |  |  | 7605 | 0.000 | 0.4961 | No |
| 42 | OR1E1 |  |  | 9534 | 0.000 | 0.3733 | No |
| 43 | TREM1 |  |  | 9547 | 0.000 | 0.3725 | No |
| 44 | PPL |  |  | 12065 | -0.700 | 0.2132 | No |
| 45 | NRGN |  |  | 12739 | -1.200 | 0.1719 | No |
| 46 | QPCT |  |  | 12946 | -1.400 | 0.1606 | No |
| 47 | FOSL2 |  |  | 13357 | -1.800 | 0.1369 | No |
| 48 | MAF |  |  | 13580 | -2.100 | 0.1256 | No |
| 49 | IGFBP5 |  |  | 14402 | -3.600 | 0.0781 | No |
| 50 | CTH |  |  | 15107 | -5.700 | 0.0409 | No |
Table: GSEA details [plain text format]

  

Fig 2: TANG\_SENESCENCE\_TP53\_TARGETS\_DN: Random ES distribution      
 Gene set null distribution of ES for **TANG\_SENESCENCE\_TP53\_TARGETS\_DN**

  
