# Supplementary material for: Immunomic, genomic and transcriptomic characterization of CT26 colorectal carcinoma
Source: BMC Genomics. 2014 Mar 13;15(1):190. doi: 10.1186/1471-2164-15-190 (PMC4007559; doi:10.1186/1471-2164-15-190)
Supplement: Supplementary file 8 — Additional file 8: Contains the Gene Pattern gene set membership and enrichment values in an html format. The file index.html is the entry point. (ZIP 13 MB) [file 12864_2013_7028_MOESM8_ESM.zip › TURASHVILI_BREAST_DUCTAL_CARCINOMA_VS_DUCTAL_NORMAL_DN.html]

Details for gene set TURASHVILI\_BREAST\_DUCTAL\_CARCINOMA\_VS\_DUCTAL\_NORMAL\_DN[GSEA]

|  || Dataset | CT26\_gene\_expression |
| Phenotype | NoPhenotypeAvailable |
| Upregulated in class | na\_neg |
| GeneSet | TURASHVILI\_BREAST\_DUCTAL\_CARCINOMA\_VS\_DUCTAL\_NORMAL\_DN |
| Enrichment Score (ES) | -0.4140414 |
| Normalized Enrichment Score (NES) | NaN |
| Nominal p-value | NaN |
| FDR q-value | 1.0 |
| FWER p-Value | 0.0 |
Table: GSEA Results Summary

  

Fig 1: Enrichment plot: TURASHVILI\_BREAST\_DUCTAL\_CARCINOMA\_VS\_DUCTAL\_NORMAL\_DN      
 Profile of the Running ES Score & Positions of GeneSet Members on the Rank Ordered List

  

| PROBE | GENE SYMBOL | GENE\_TITLE | RANK IN GENE LIST | RANK METRIC SCORE | RUNNING ES | CORE ENRICHMENT || 1 | CTSL2 |  |  | 267 | 22.600 | 0.0127 | No |
| 2 | ANXA1 |  |  | 697 | 16.400 | 0.0068 | No |
| 3 | PLEKHH2 |  |  | 776 | 15.800 | 0.0226 | No |
| 4 | CAV2 |  |  | 871 | 15.100 | 0.0365 | No |
| 5 | MET |  |  | 954 | 14.500 | 0.0504 | No |
| 6 | AK5 |  |  | 960 | 14.500 | 0.0692 | No |
| 7 | EGR1 |  |  | 1050 | 13.900 | 0.0818 | No |
| 8 | TRIP6 |  |  | 1123 | 13.400 | 0.0948 | No |
| 9 | RARRES1 |  |  | 1222 | 13.000 | 0.1057 | No |
| 10 | TM4SF1 |  |  | 1353 | 12.300 | 0.1136 | No |
| 11 | PSME4 |  |  | 1361 | 12.300 | 0.1294 | No |
| 12 | SPRED1 |  |  | 1466 | 11.800 | 0.1382 | No |
| 13 | DST |  |  | 1611 | 11.200 | 0.1438 | No |
| 14 | SOCS5 |  |  | 1680 | 10.900 | 0.1538 | No |
| 15 | SLC25A37 |  |  | 2080 | 9.400 | 0.1406 | No |
| 16 | MID1 |  |  | 2183 | 9.100 | 0.1461 | No |
| 17 | SF1 |  |  | 2224 | 9.000 | 0.1554 | No |
| 18 | INTS10 |  |  | 2509 | 8.200 | 0.1480 | No |
| 19 | CSNK1A1 |  |  | 2521 | 8.100 | 0.1579 | No |
| 20 | PTPN21 |  |  | 2690 | 7.700 | 0.1573 | No |
| 21 | RND3 |  |  | 2796 | 7.400 | 0.1603 | No |
| 22 | CP |  |  | 3184 | 6.500 | 0.1441 | No |
| 23 | SEC14L1 |  |  | 3319 | 6.200 | 0.1437 | No |
| 24 | NFIB |  |  | 3497 | 5.800 | 0.1400 | No |
| 25 | DLG5 |  |  | 3534 | 5.700 | 0.1452 | No |
| 26 | USP31 |  |  | 3586 | 5.600 | 0.1493 | No |
| 27 | CSDA |  |  | 4022 | 4.700 | 0.1276 | No |
| 28 | CHIC2 |  |  | 4976 | 3.100 | 0.0705 | No |
| 29 | ZNF462 |  |  | 5008 | 3.000 | 0.0725 | No |
| 30 | LAMC2 |  |  | 5100 | 2.900 | 0.0705 | No |
| 31 | NCOA7 |  |  | 5114 | 2.900 | 0.0735 | No |
| 32 | RAPGEF2 |  |  | 5223 | 2.700 | 0.0701 | No |
| 33 | CDC14B |  |  | 5228 | 2.700 | 0.0734 | No |
| 34 | BCOR |  |  | 5312 | 2.600 | 0.0715 | No |
| 35 | CD59 |  |  | 5419 | 2.400 | 0.0679 | No |
| 36 | SLC25A45 |  |  | 6064 | 1.500 | 0.0286 | No |
| 37 | MAFF |  |  | 6082 | 1.500 | 0.0295 | No |
| 38 | PDZK1 |  |  | 6158 | 1.400 | 0.0265 | No |
| 39 | CRYAB |  |  | 6197 | 1.400 | 0.0259 | No |
| 40 | ST3GAL6 |  |  | 6216 | 1.300 | 0.0265 | No |
| 41 | FOSB |  |  | 6375 | 1.100 | 0.0178 | No |
| 42 | ULK4 |  |  | 6649 | 0.800 | 0.0013 | No |
| 43 | APOD |  |  | 7013 | 0.400 | -0.0214 | No |
| 44 | VTCN1 |  |  | 7190 | 0.200 | -0.0325 | No |
| 45 | RBPMS |  |  | 7326 | 0.100 | -0.0410 | No |
| 46 | ZNF667 |  |  | 7349 | 0.100 | -0.0423 | No |
| 47 | ITGB8 |  |  | 7715 | 0.000 | -0.0657 | No |
| 48 | CDRT4 |  |  | 7723 | 0.000 | -0.0661 | No |
| 49 | CFI |  |  | 7765 | 0.000 | -0.0687 | No |
| 50 | TTC18 |  |  | 8022 | 0.000 | -0.0852 | No |
| 51 | CYB5R2 |  |  | 8194 | 0.000 | -0.0961 | No |
| 52 | PROL1 |  |  | 8789 | 0.000 | -0.1342 | No |
| 53 | MYBPC1 |  |  | 9007 | 0.000 | -0.1482 | No |
| 54 | TFAP2B |  |  | 9039 | 0.000 | -0.1501 | No |
| 55 | DSC3 |  |  | 9097 | 0.000 | -0.1538 | No |
| 56 | KLK7 |  |  | 9135 | 0.000 | -0.1562 | No |
| 57 | DSG3 |  |  | 9142 | 0.000 | -0.1566 | No |
| 58 | MAP2 |  |  | 9517 | 0.000 | -0.1805 | No |
| 59 | ELF5 |  |  | 9571 | 0.000 | -0.1839 | No |
| 60 | PTN |  |  | 9619 | 0.000 | -0.1870 | No |
| 61 | LTF |  |  | 9647 | 0.000 | -0.1887 | No |
| 62 | BBOX1 |  |  | 9679 | 0.000 | -0.1907 | No |
| 63 | PI15 |  |  | 9807 | 0.000 | -0.1988 | No |
| 64 | SCN4B |  |  | 9886 | 0.000 | -0.2038 | No |
| 65 | CMYA5 |  |  | 9984 | 0.000 | -0.2100 | No |
| 66 | FHOD3 |  |  | 10019 | 0.000 | -0.2122 | No |
| 67 | CHL1 |  |  | 10097 | 0.000 | -0.2172 | No |
| 68 | GABRP |  |  | 10218 | -0.100 | -0.2247 | No |
| 69 | SERPINA1 |  |  | 10232 | -0.100 | -0.2254 | No |
| 70 | KRT5 |  |  | 10251 | -0.100 | -0.2265 | No |
| 71 | CYP4X1 |  |  | 10364 | -0.100 | -0.2335 | No |
| 72 | ARMCX4 |  |  | 10414 | -0.100 | -0.2365 | No |
| 73 | COL17A1 |  |  | 10433 | -0.100 | -0.2375 | No |
| 74 | KRT16 |  |  | 10524 | -0.100 | -0.2432 | No |
| 75 | CHI3L1 |  |  | 10568 | -0.100 | -0.2458 | No |
| 76 | ZDHHC3 |  |  | 10589 | -0.100 | -0.2470 | No |
| 77 | KRT15 |  |  | 10616 | -0.100 | -0.2485 | No |
| 78 | PTX3 |  |  | 10726 | -0.100 | -0.2554 | No |
| 79 | ATP10D |  |  | 10728 | -0.100 | -0.2553 | No |
| 80 | GABRE |  |  | 10761 | -0.100 | -0.2572 | No |
| 81 | GPM6B |  |  | 10772 | -0.100 | -0.2577 | No |
| 82 | KIAA1217 |  |  | 10832 | -0.200 | -0.2612 | No |
| 83 | OXTR |  |  | 10880 | -0.200 | -0.2640 | No |
| 84 | MMP7 |  |  | 10891 | -0.200 | -0.2644 | No |
| 85 | TRIM29 |  |  | 10939 | -0.200 | -0.2671 | No |
| 86 | CLDN11 |  |  | 10988 | -0.200 | -0.2699 | No |
| 87 | PALMD |  |  | 11012 | -0.200 | -0.2711 | No |
| 88 | FZD7 |  |  | 11141 | -0.200 | -0.2791 | No |
| 89 | KCNC4 |  |  | 11336 | -0.300 | -0.2911 | No |
| 90 | KRT23 |  |  | 11398 | -0.300 | -0.2947 | No |
| 91 | TFPI2 |  |  | 11426 | -0.300 | -0.2960 | No |
| 92 | ADAMTS5 |  |  | 11465 | -0.400 | -0.2979 | No |
| 93 | KRT14 |  |  | 11559 | -0.400 | -0.3033 | No |
| 94 | SAMD5 |  |  | 11966 | -0.600 | -0.3286 | No |
| 95 | CDH3 |  |  | 11996 | -0.600 | -0.3297 | No |
| 96 | EDN3 |  |  | 12211 | -0.800 | -0.3423 | No |
| 97 | ALDH1A3 |  |  | 12233 | -0.800 | -0.3426 | No |
| 98 | LAMA3 |  |  | 12360 | -0.900 | -0.3495 | No |
| 99 | MAOB |  |  | 12706 | -1.100 | -0.3702 | No |
| 100 | PER1 |  |  | 12710 | -1.200 | -0.3688 | No |
| 101 | LIN37 |  |  | 12847 | -1.300 | -0.3758 | No |
| 102 | MEGF6 |  |  | 12947 | -1.400 | -0.3803 | No |
| 103 | PAPLN |  |  | 13025 | -1.500 | -0.3833 | No |
| 104 | TGFA |  |  | 13058 | -1.500 | -0.3833 | No |
| 105 | CCL28 |  |  | 13098 | -1.600 | -0.3837 | No |
| 106 | MAMDC2 |  |  | 13221 | -1.700 | -0.3893 | No |
| 107 | SOD2 |  |  | 13242 | -1.700 | -0.3884 | No |
| 108 | GATM |  |  | 13389 | -1.900 | -0.3952 | No |
| 109 | RRAD |  |  | 13458 | -2.000 | -0.3969 | No |
| 110 | ANPEP |  |  | 13506 | -2.000 | -0.3973 | No |
| 111 | GPC3 |  |  | 13526 | -2.100 | -0.3958 | No |
| 112 | SORBS2 |  |  | 13622 | -2.200 | -0.3990 | No |
| 113 | EGFR |  |  | 13693 | -2.300 | -0.4004 | No |
| 114 | ATF3 |  |  | 13829 | -2.500 | -0.4058 | No |
| 115 | GRAMD3 |  |  | 13900 | -2.600 | -0.4068 | No |
| 116 | TAC1 |  |  | 13930 | -2.700 | -0.4051 | No |
| 117 | CFD |  |  | 13989 | -2.800 | -0.4052 | No |
| 118 | RAB27A |  |  | 13990 | -2.800 | -0.4015 | No |
| 119 | ATP1A2 |  |  | 13996 | -2.800 | -0.3981 | No |
| 120 | FAM110C |  |  | 14034 | -2.800 | -0.3968 | No |
| 121 | TSHZ2 |  |  | 14304 | -3.300 | -0.4097 | Yes |
| 122 | ID4 |  |  | 14361 | -3.500 | -0.4087 | Yes |
| 123 | TF |  |  | 14444 | -3.700 | -0.4090 | Yes |
| 124 | SOX9 |  |  | 14468 | -3.800 | -0.4055 | Yes |
| 125 | HOXA3 |  |  | 14480 | -3.800 | -0.4012 | Yes |
| 126 | KRT17 |  |  | 14517 | -3.900 | -0.3984 | Yes |
| 127 | TUBB2B |  |  | 14644 | -4.200 | -0.4009 | Yes |
| 128 | TRIM2 |  |  | 14677 | -4.300 | -0.3973 | Yes |
| 129 | MGST1 |  |  | 14718 | -4.400 | -0.3941 | Yes |
| 130 | FOS |  |  | 14767 | -4.500 | -0.3912 | Yes |
| 131 | SFRP1 |  |  | 14932 | -5.000 | -0.3951 | Yes |
| 132 | KIT |  |  | 14956 | -5.100 | -0.3899 | Yes |
| 133 | MYLK |  |  | 14958 | -5.100 | -0.3832 | Yes |
| 134 | PDLIM3 |  |  | 15016 | -5.300 | -0.3799 | Yes |
| 135 | SYNPO2 |  |  | 15038 | -5.400 | -0.3741 | Yes |
| 136 | CMTM8 |  |  | 15195 | -6.100 | -0.3761 | Yes |
| 137 | CX3CL1 |  |  | 15197 | -6.200 | -0.3680 | Yes |
| 138 | MYH11 |  |  | 15218 | -6.300 | -0.3609 | Yes |
| 139 | ITGB4 |  |  | 15227 | -6.300 | -0.3531 | Yes |
| 140 | DUSP1 |  |  | 15260 | -6.500 | -0.3466 | Yes |
| 141 | STEAP3 |  |  | 15323 | -6.900 | -0.3415 | Yes |
| 142 | TNC |  |  | 15385 | -7.400 | -0.3356 | Yes |
| 143 | EHF |  |  | 15467 | -8.200 | -0.3300 | Yes |
| 144 | HOXA9 |  |  | 15470 | -8.200 | -0.3193 | Yes |
| 145 | FBXO32 |  |  | 15477 | -8.300 | -0.3088 | Yes |
| 146 | HMGCS2 |  |  | 15535 | -9.100 | -0.3004 | Yes |
| 147 | CLDN8 |  |  | 15568 | -9.600 | -0.2898 | Yes |
| 148 | IGJ |  |  | 15648 | -11.800 | -0.2793 | Yes |
| 149 | MGP |  |  | 15667 | -12.500 | -0.2640 | Yes |
| 150 | LAMB3 |  |  | 15671 | -12.700 | -0.2475 | Yes |
| 151 | TAGLN |  |  | 15684 | -13.700 | -0.2302 | Yes |
| 152 | PIGR |  |  | 15703 | -15.500 | -0.2109 | Yes |
| 153 | ELF3 |  |  | 15708 | -16.000 | -0.1900 | Yes |
| 154 | CNN1 |  |  | 15718 | -16.900 | -0.1683 | Yes |
| 155 | ACTA2 |  |  | 15733 | -20.600 | -0.1420 | Yes |
| 156 | PDZK1IP1 |  |  | 15737 | -22.300 | -0.1128 | Yes |
| 157 | NDRG2 |  |  | 15740 | -25.600 | -0.0792 | Yes |
| 158 | KRT7 |  |  | 15742 | -26.100 | -0.0448 | Yes |
| 159 | ACTG2 |  |  | 15747 | -34.300 | 0.0001 | Yes |
Table: GSEA details [plain text format]

  

Fig 2: TURASHVILI\_BREAST\_DUCTAL\_CARCINOMA\_VS\_DUCTAL\_NORMAL\_DN: Random ES distribution      
 Gene set null distribution of ES for **TURASHVILI\_BREAST\_DUCTAL\_CARCINOMA\_VS\_DUCTAL\_NORMAL\_DN**

  
