# Supplementary material for: Immunomic, genomic and transcriptomic characterization of CT26 colorectal carcinoma
Source: BMC Genomics. 2014 Mar 13;15(1):190. doi: 10.1186/1471-2164-15-190 (PMC4007559; doi:10.1186/1471-2164-15-190)
Supplement: Supplementary file 8 — Additional file 8: Contains the Gene Pattern gene set membership and enrichment values in an html format. The file index.html is the entry point. (ZIP 13 MB) [file 12864_2013_7028_MOESM8_ESM.zip › VECCHI_GASTRIC_CANCER_ADVANCED_VS_EARLY_DN.html]

Details for gene set VECCHI\_GASTRIC\_CANCER\_ADVANCED\_VS\_EARLY\_DN[GSEA]

|  || Dataset | CT26\_gene\_expression |
| Phenotype | NoPhenotypeAvailable |
| Upregulated in class | na\_neg |
| GeneSet | VECCHI\_GASTRIC\_CANCER\_ADVANCED\_VS\_EARLY\_DN |
| Enrichment Score (ES) | -0.5774733 |
| Normalized Enrichment Score (NES) | NaN |
| Nominal p-value | NaN |
| FDR q-value | 1.0 |
| FWER p-Value | 0.0 |
Table: GSEA Results Summary

  

Fig 1: Enrichment plot: VECCHI\_GASTRIC\_CANCER\_ADVANCED\_VS\_EARLY\_DN      
 Profile of the Running ES Score & Positions of GeneSet Members on the Rank Ordered List

  

| PROBE | GENE SYMBOL | GENE\_TITLE | RANK IN GENE LIST | RANK METRIC SCORE | RUNNING ES | CORE ENRICHMENT || 1 | HMMR |  |  | 246 | 23.200 | 0.0221 | No |
| 2 | GOT1 |  |  | 810 | 15.600 | 0.0116 | No |
| 3 | ALDH3A1 |  |  | 813 | 15.500 | 0.0367 | No |
| 4 | ESCO2 |  |  | 1671 | 10.900 | -0.0003 | No |
| 5 | AK1 |  |  | 1698 | 10.800 | 0.0157 | No |
| 6 | SLMO2 |  |  | 1969 | 9.800 | 0.0144 | No |
| 7 | RBM19 |  |  | 2140 | 9.200 | 0.0185 | No |
| 8 | AKR1C3 |  |  | 2269 | 8.900 | 0.0249 | No |
| 9 | RPS27L |  |  | 2638 | 7.800 | 0.0140 | No |
| 10 | XRCC4 |  |  | 2895 | 7.200 | 0.0094 | No |
| 11 | IFT57 |  |  | 3271 | 6.300 | -0.0043 | No |
| 12 | SSNA1 |  |  | 3413 | 6.000 | -0.0035 | No |
| 13 | ABHD12 |  |  | 3640 | 5.500 | -0.0090 | No |
| 14 | BLNK |  |  | 4021 | 4.700 | -0.0256 | No |
| 15 | ABHD13 |  |  | 4289 | 4.300 | -0.0357 | No |
| 16 | CHAC2 |  |  | 4908 | 3.200 | -0.0700 | No |
| 17 | RER1 |  |  | 5199 | 2.700 | -0.0841 | No |
| 18 | CA13 |  |  | 5217 | 2.700 | -0.0808 | No |
| 19 | GCNT1 |  |  | 5384 | 2.400 | -0.0875 | No |
| 20 | GATA4 |  |  | 5436 | 2.400 | -0.0868 | No |
| 21 | NAT1 |  |  | 5674 | 2.100 | -0.0986 | No |
| 22 | SENP8 |  |  | 6323 | 1.200 | -0.1380 | No |
| 23 | DOT1L |  |  | 6486 | 0.900 | -0.1469 | No |
| 24 | DERL1 |  |  | 6590 | 0.800 | -0.1522 | No |
| 25 | BTD |  |  | 6749 | 0.700 | -0.1612 | No |
| 26 | ODAM |  |  | 7623 | 0.000 | -0.2170 | No |
| 27 | CLDN18 |  |  | 7680 | 0.000 | -0.2206 | No |
| 28 | HGD |  |  | 7682 | 0.000 | -0.2206 | No |
| 29 | NAT2 |  |  | 7722 | 0.000 | -0.2231 | No |
| 30 | KCNK10 |  |  | 9664 | 0.000 | -0.3472 | No |
| 31 | CPS1 |  |  | 10293 | -0.100 | -0.3872 | No |
| 32 | CHCHD5 |  |  | 10596 | -0.100 | -0.4064 | No |
| 33 | ARX |  |  | 10961 | -0.200 | -0.4293 | No |
| 34 | ATP10B |  |  | 11566 | -0.400 | -0.4673 | No |
| 35 | BLVRB |  |  | 11639 | -0.400 | -0.4713 | No |
| 36 | OTC |  |  | 11725 | -0.500 | -0.4759 | No |
| 37 | TSIX |  |  | 11755 | -0.500 | -0.4769 | No |
| 38 | CFTR |  |  | 12341 | -0.900 | -0.5129 | No |
| 39 | PRSS3 |  |  | 12352 | -0.900 | -0.5120 | No |
| 40 | ENTPD3 |  |  | 12371 | -0.900 | -0.5117 | No |
| 41 | REG1A |  |  | 12386 | -0.900 | -0.5111 | No |
| 42 | PRSS2 |  |  | 12387 | -0.900 | -0.5097 | No |
| 43 | GP2 |  |  | 12412 | -0.900 | -0.5097 | No |
| 44 | TFF2 |  |  | 12436 | -1.000 | -0.5096 | No |
| 45 | POLD4 |  |  | 12916 | -1.400 | -0.5379 | No |
| 46 | ABCG2 |  |  | 12966 | -1.400 | -0.5388 | No |
| 47 | NMU |  |  | 13073 | -1.500 | -0.5431 | No |
| 48 | CYB561D1 |  |  | 13194 | -1.700 | -0.5480 | No |
| 49 | ABCG5 |  |  | 13273 | -1.700 | -0.5502 | No |
| 50 | REG3A |  |  | 13359 | -1.800 | -0.5527 | No |
| 51 | VILL |  |  | 13365 | -1.800 | -0.5501 | No |
| 52 | SLPI |  |  | 13439 | -1.900 | -0.5517 | No |
| 53 | NDUFA7 |  |  | 13489 | -2.000 | -0.5515 | No |
| 54 | PTPRN2 |  |  | 13502 | -2.000 | -0.5490 | No |
| 55 | SLC4A4 |  |  | 13592 | -2.100 | -0.5513 | No |
| 56 | RFFL |  |  | 13627 | -2.200 | -0.5499 | No |
| 57 | SULT1C2 |  |  | 13650 | -2.200 | -0.5477 | No |
| 58 | COX8A |  |  | 13681 | -2.300 | -0.5459 | No |
| 59 | KIAA1244 |  |  | 13722 | -2.400 | -0.5445 | No |
| 60 | AADAC |  |  | 13745 | -2.400 | -0.5420 | No |
| 61 | ISG20 |  |  | 13963 | -2.700 | -0.5515 | No |
| 62 | CALML4 |  |  | 14049 | -2.900 | -0.5522 | No |
| 63 | MUC1 |  |  | 14055 | -2.900 | -0.5478 | No |
| 64 | CLRN3 |  |  | 14476 | -3.800 | -0.5684 | No |
| 65 | VPS28 |  |  | 14558 | -3.900 | -0.5672 | No |
| 66 | VSIG2 |  |  | 14719 | -4.400 | -0.5703 | Yes |
| 67 | REEP6 |  |  | 14747 | -4.500 | -0.5647 | Yes |
| 68 | ADRA2A |  |  | 14754 | -4.500 | -0.5577 | Yes |
| 69 | WNK4 |  |  | 14770 | -4.500 | -0.5513 | Yes |
| 70 | PCBD1 |  |  | 14842 | -4.700 | -0.5482 | Yes |
| 71 | CAPN13 |  |  | 14865 | -4.800 | -0.5418 | Yes |
| 72 | TMC5 |  |  | 14928 | -5.000 | -0.5376 | Yes |
| 73 | MS4A8B |  |  | 14975 | -5.100 | -0.5322 | Yes |
| 74 | TMEM45B |  |  | 14978 | -5.100 | -0.5240 | Yes |
| 75 | CA2 |  |  | 14983 | -5.200 | -0.5158 | Yes |
| 76 | DDT |  |  | 14996 | -5.200 | -0.5081 | Yes |
| 77 | PIP5K1B |  |  | 15043 | -5.400 | -0.5022 | Yes |
| 78 | ALDOB |  |  | 15117 | -5.800 | -0.4974 | Yes |
| 79 | FXYD3 |  |  | 15130 | -5.900 | -0.4886 | Yes |
| 80 | SSTR1 |  |  | 15216 | -6.300 | -0.4837 | Yes |
| 81 | CLDN23 |  |  | 15239 | -6.400 | -0.4747 | Yes |
| 82 | SERPINB1 |  |  | 15245 | -6.400 | -0.4646 | Yes |
| 83 | CDC42EP5 |  |  | 15246 | -6.400 | -0.4541 | Yes |
| 84 | REG4 |  |  | 15297 | -6.700 | -0.4464 | Yes |
| 85 | MLPH |  |  | 15321 | -6.900 | -0.4366 | Yes |
| 86 | GPA33 |  |  | 15340 | -7.000 | -0.4263 | Yes |
| 87 | EPN3 |  |  | 15354 | -7.100 | -0.4156 | Yes |
| 88 | ZBTB7B |  |  | 15356 | -7.100 | -0.4041 | Yes |
| 89 | GCNT3 |  |  | 15372 | -7.300 | -0.3931 | Yes |
| 90 | CES2 |  |  | 15388 | -7.400 | -0.3820 | Yes |
| 91 | HSD17B2 |  |  | 15402 | -7.500 | -0.3706 | Yes |
| 92 | CYC1 |  |  | 15409 | -7.600 | -0.3586 | Yes |
| 93 | ADH1C |  |  | 15410 | -7.600 | -0.3462 | Yes |
| 94 | TFF3 |  |  | 15423 | -7.700 | -0.3344 | Yes |
| 95 | CAPN9 |  |  | 15493 | -8.400 | -0.3251 | Yes |
| 96 | CRIP1 |  |  | 15534 | -9.000 | -0.3130 | Yes |
| 97 | CLDN15 |  |  | 15595 | -10.100 | -0.3003 | Yes |
| 98 | SST |  |  | 15599 | -10.200 | -0.2839 | Yes |
| 99 | TM4SF20 |  |  | 15603 | -10.300 | -0.2673 | Yes |
| 100 | RHPN2 |  |  | 15609 | -10.400 | -0.2506 | Yes |
| 101 | TMPRSS2 |  |  | 15627 | -11.100 | -0.2336 | Yes |
| 102 | STARD10 |  |  | 15644 | -11.700 | -0.2156 | Yes |
| 103 | GMDS |  |  | 15655 | -12.000 | -0.1966 | Yes |
| 104 | KIAA1324 |  |  | 15680 | -13.300 | -0.1765 | Yes |
| 105 | FAM3D |  |  | 15690 | -14.200 | -0.1539 | Yes |
| 106 | DPP3 |  |  | 15693 | -14.400 | -0.1305 | Yes |
| 107 | SULT1A1 |  |  | 15697 | -14.900 | -0.1064 | Yes |
| 108 | KCNE3 |  |  | 15699 | -15.200 | -0.0817 | Yes |
| 109 | TPD52 |  |  | 15701 | -15.300 | -0.0568 | Yes |
| 110 | LGALS4 |  |  | 15713 | -16.500 | -0.0306 | Yes |
| 111 | ETHE1 |  |  | 15731 | -20.100 | 0.0012 | Yes |
Table: GSEA details [plain text format]

  

Fig 2: VECCHI\_GASTRIC\_CANCER\_ADVANCED\_VS\_EARLY\_DN: Random ES distribution      
 Gene set null distribution of ES for **VECCHI\_GASTRIC\_CANCER\_ADVANCED\_VS\_EARLY\_DN**

  
