# Supplementary material for: Immunomic, genomic and transcriptomic characterization of CT26 colorectal carcinoma
Source: BMC Genomics. 2014 Mar 13;15(1):190. doi: 10.1186/1471-2164-15-190 (PMC4007559; doi:10.1186/1471-2164-15-190)
Supplement: Supplementary file 8 — Additional file 8: Contains the Gene Pattern gene set membership and enrichment values in an html format. The file index.html is the entry point. (ZIP 13 MB) [file 12864_2013_7028_MOESM8_ESM.zip › WINNEPENNINCKX_MELANOMA_METASTASIS_UP.html]

Details for gene set WINNEPENNINCKX\_MELANOMA\_METASTASIS\_UP[GSEA]

|  || Dataset | CT26\_gene\_expression |
| Phenotype | NoPhenotypeAvailable |
| Upregulated in class | na\_pos |
| GeneSet | WINNEPENNINCKX\_MELANOMA\_METASTASIS\_UP |
| Enrichment Score (ES) | 0.7707017 |
| Normalized Enrichment Score (NES) | 1.7004929 |
| Nominal p-value | 0.0 |
| FDR q-value | 0.0017187949 |
| FWER p-Value | 0.021 |
Table: GSEA Results Summary

  

Fig 1: Enrichment plot: WINNEPENNINCKX\_MELANOMA\_METASTASIS\_UP      
 Profile of the Running ES Score & Positions of GeneSet Members on the Rank Ordered List

  

| PROBE | GENE SYMBOL | GENE\_TITLE | RANK IN GENE LIST | RANK METRIC SCORE | RUNNING ES | CORE ENRICHMENT || 1 | TOP2A |  |  | 8 | 56.000 | 0.0301 | Yes |
| 2 | CKS1B |  |  | 48 | 35.600 | 0.0471 | Yes |
| 3 | MCM4 |  |  | 81 | 31.500 | 0.0622 | Yes |
| 4 | ECT2 |  |  | 100 | 30.500 | 0.0778 | Yes |
| 5 | SRP19 |  |  | 147 | 27.000 | 0.0896 | Yes |
| 6 | XPO1 |  |  | 148 | 27.000 | 0.1043 | Yes |
| 7 | DDX18 |  |  | 160 | 26.700 | 0.1182 | Yes |
| 8 | NASP |  |  | 161 | 26.700 | 0.1328 | Yes |
| 9 | UHRF1 |  |  | 162 | 26.600 | 0.1474 | Yes |
| 10 | MCM6 |  |  | 163 | 26.600 | 0.1619 | Yes |
| 11 | PRC1 |  |  | 181 | 25.500 | 0.1748 | Yes |
| 12 | KIAA0101 |  |  | 186 | 25.100 | 0.1882 | Yes |
| 13 | KIF11 |  |  | 197 | 24.700 | 0.2011 | Yes |
| 14 | SSR3 |  |  | 221 | 24.000 | 0.2127 | Yes |
| 15 | PTTG1 |  |  | 223 | 23.900 | 0.2257 | Yes |
| 16 | TCOF1 |  |  | 275 | 22.400 | 0.2347 | Yes |
| 17 | SHCBP1 |  |  | 296 | 21.800 | 0.2454 | Yes |
| 18 | WHSC1 |  |  | 300 | 21.700 | 0.2570 | Yes |
| 19 | CCT5 |  |  | 321 | 21.200 | 0.2673 | Yes |
| 20 | MSH6 |  |  | 338 | 21.000 | 0.2778 | Yes |
| 21 | NEK2 |  |  | 346 | 20.900 | 0.2888 | Yes |
| 22 | KPNA2 |  |  | 354 | 20.700 | 0.2997 | Yes |
| 23 | HSPD1 |  |  | 357 | 20.600 | 0.3108 | Yes |
| 24 | BUB1 |  |  | 360 | 20.600 | 0.3219 | Yes |
| 25 | PWP1 |  |  | 364 | 20.500 | 0.3329 | Yes |
| 26 | ANLN |  |  | 374 | 20.400 | 0.3435 | Yes |
| 27 | SMC2 |  |  | 379 | 20.300 | 0.3544 | Yes |
| 28 | PCNA |  |  | 386 | 20.200 | 0.3650 | Yes |
| 29 | CENPN |  |  | 403 | 19.900 | 0.3749 | Yes |
| 30 | IPO7 |  |  | 423 | 19.500 | 0.3843 | Yes |
| 31 | CENPF |  |  | 431 | 19.400 | 0.3945 | Yes |
| 32 | TARS |  |  | 434 | 19.300 | 0.4049 | Yes |
| 33 | NCAPH |  |  | 459 | 19.000 | 0.4137 | Yes |
| 34 | CHORDC1 |  |  | 460 | 19.000 | 0.4241 | Yes |
| 35 | IMMT |  |  | 475 | 18.700 | 0.4335 | Yes |
| 36 | BIRC5 |  |  | 507 | 18.400 | 0.4415 | Yes |
| 37 | NCAPG |  |  | 524 | 18.200 | 0.4505 | Yes |
| 38 | ENY2 |  |  | 537 | 18.100 | 0.4596 | Yes |
| 39 | NME1 |  |  | 540 | 18.100 | 0.4694 | Yes |
| 40 | PAICS |  |  | 546 | 18.000 | 0.4789 | Yes |
| 41 | CACYBP |  |  | 563 | 17.800 | 0.4876 | Yes |
| 42 | ZWINT |  |  | 574 | 17.700 | 0.4966 | Yes |
| 43 | ATAD2 |  |  | 596 | 17.400 | 0.5048 | Yes |
| 44 | MAP4K4 |  |  | 597 | 17.400 | 0.5143 | Yes |
| 45 | CEP55 |  |  | 662 | 16.800 | 0.5194 | Yes |
| 46 | RANBP1 |  |  | 664 | 16.800 | 0.5285 | Yes |
| 47 | MRPS17 |  |  | 675 | 16.700 | 0.5370 | Yes |
| 48 | RACGAP1 |  |  | 682 | 16.600 | 0.5457 | Yes |
| 49 | NCBP1 |  |  | 721 | 16.200 | 0.5521 | Yes |
| 50 | NCAPG2 |  |  | 774 | 15.800 | 0.5574 | Yes |
| 51 | AURKA |  |  | 799 | 15.600 | 0.5644 | Yes |
| 52 | HSPA5 |  |  | 844 | 15.300 | 0.5700 | Yes |
| 53 | NUF2 |  |  | 866 | 15.100 | 0.5769 | Yes |
| 54 | CCT7 |  |  | 867 | 15.100 | 0.5851 | Yes |
| 55 | NDC80 |  |  | 878 | 15.000 | 0.5927 | Yes |
| 56 | GMNN |  |  | 911 | 14.800 | 0.5987 | Yes |
| 57 | NUDCD1 |  |  | 922 | 14.700 | 0.6061 | Yes |
| 58 | SPAG5 |  |  | 927 | 14.700 | 0.6139 | Yes |
| 59 | GMPS |  |  | 994 | 14.200 | 0.6174 | Yes |
| 60 | CNN3 |  |  | 1006 | 14.200 | 0.6245 | Yes |
| 61 | SGOL2 |  |  | 1015 | 14.100 | 0.6317 | Yes |
| 62 | CDCA8 |  |  | 1017 | 14.100 | 0.6393 | Yes |
| 63 | KIF2C |  |  | 1036 | 14.000 | 0.6458 | Yes |
| 64 | MELK |  |  | 1077 | 13.700 | 0.6508 | Yes |
| 65 | RRM2 |  |  | 1084 | 13.700 | 0.6579 | Yes |
| 66 | HSPA4 |  |  | 1112 | 13.500 | 0.6635 | Yes |
| 67 | ZNF367 |  |  | 1115 | 13.400 | 0.6707 | Yes |
| 68 | CCNB2 |  |  | 1155 | 13.200 | 0.6754 | Yes |
| 69 | FAM98A |  |  | 1177 | 13.100 | 0.6813 | Yes |
| 70 | KIRREL |  |  | 1219 | 13.000 | 0.6857 | Yes |
| 71 | SPC25 |  |  | 1296 | 12.600 | 0.6878 | Yes |
| 72 | H2AFV |  |  | 1310 | 12.500 | 0.6938 | Yes |
| 73 | SMARCA5 |  |  | 1341 | 12.300 | 0.6986 | Yes |
| 74 | CTNNAL1 |  |  | 1390 | 12.100 | 0.7021 | Yes |
| 75 | RBMX |  |  | 1453 | 11.800 | 0.7046 | Yes |
| 76 | CCT4 |  |  | 1578 | 11.300 | 0.7028 | Yes |
| 77 | UBE2T |  |  | 1606 | 11.200 | 0.7072 | Yes |
| 78 | RPA3 |  |  | 1621 | 11.200 | 0.7124 | Yes |
| 79 | RFC5 |  |  | 1676 | 10.900 | 0.7149 | Yes |
| 80 | MTERFD1 |  |  | 1719 | 10.800 | 0.7182 | Yes |
| 81 | MRPL32 |  |  | 1724 | 10.700 | 0.7238 | Yes |
| 82 | CENPA |  |  | 1762 | 10.600 | 0.7272 | Yes |
| 83 | HTRA2 |  |  | 1769 | 10.600 | 0.7326 | Yes |
| 84 | CDCA5 |  |  | 1813 | 10.400 | 0.7355 | Yes |
| 85 | GEMIN6 |  |  | 1855 | 10.200 | 0.7385 | Yes |
| 86 | ASPM |  |  | 1918 | 10.000 | 0.7400 | Yes |
| 87 | PRPF38B |  |  | 1942 | 9.900 | 0.7439 | Yes |
| 88 | CKS2 |  |  | 1959 | 9.800 | 0.7482 | Yes |
| 89 | MRPS5 |  |  | 1999 | 9.700 | 0.7511 | Yes |
| 90 | DNAJA1 |  |  | 2018 | 9.600 | 0.7551 | Yes |
| 91 | MRPS10 |  |  | 2119 | 9.300 | 0.7538 | Yes |
| 92 | RFC4 |  |  | 2243 | 8.900 | 0.7508 | Yes |
| 93 | KNTC1 |  |  | 2256 | 8.900 | 0.7549 | Yes |
| 94 | PHF14 |  |  | 2260 | 8.900 | 0.7596 | Yes |
| 95 | CCNB1 |  |  | 2266 | 8.900 | 0.7641 | Yes |
| 96 | RSRC1 |  |  | 2312 | 8.700 | 0.7660 | Yes |
| 97 | DHFR |  |  | 2366 | 8.600 | 0.7673 | Yes |
| 98 | NEIL3 |  |  | 2417 | 8.500 | 0.7688 | Yes |
| 99 | IWS1 |  |  | 2511 | 8.100 | 0.7672 | Yes |
| 100 | GJC1 |  |  | 2527 | 8.100 | 0.7707 | Yes |
| 101 | SNRPG |  |  | 2615 | 7.900 | 0.7694 | No |
| 102 | TK1 |  |  | 2762 | 7.500 | 0.7642 | No |
| 103 | PSMC3IP |  |  | 2785 | 7.400 | 0.7668 | No |
| 104 | CDKN3 |  |  | 2917 | 7.100 | 0.7623 | No |
| 105 | DCBLD2 |  |  | 2971 | 7.000 | 0.7628 | No |
| 106 | TIMELESS |  |  | 3000 | 6.900 | 0.7647 | No |
| 107 | GINS2 |  |  | 3094 | 6.700 | 0.7624 | No |
| 108 | RAD54L |  |  | 3132 | 6.600 | 0.7637 | No |
| 109 | TYMS |  |  | 3182 | 6.500 | 0.7641 | No |
| 110 | DPH3 |  |  | 3517 | 5.700 | 0.7458 | No |
| 111 | CHEK1 |  |  | 3593 | 5.600 | 0.7441 | No |
| 112 | CLASP1 |  |  | 3634 | 5.500 | 0.7445 | No |
| 113 | CDC6 |  |  | 3705 | 5.300 | 0.7429 | No |
| 114 | OGG1 |  |  | 3762 | 5.200 | 0.7422 | No |
| 115 | PLOD2 |  |  | 3786 | 5.200 | 0.7436 | No |
| 116 | TRUB2 |  |  | 3963 | 4.800 | 0.7349 | No |
| 117 | EXO1 |  |  | 4074 | 4.700 | 0.7305 | No |
| 118 | DONSON |  |  | 4580 | 3.800 | 0.7002 | No |
| 119 | GLRX2 |  |  | 5087 | 2.900 | 0.6694 | No |
| 120 | MRPS16 |  |  | 5134 | 2.800 | 0.6680 | No |
| 121 | NUDT4 |  |  | 5341 | 2.500 | 0.6561 | No |
| 122 | UBFD1 |  |  | 5417 | 2.400 | 0.6526 | No |
| 123 | RBM33 |  |  | 5483 | 2.300 | 0.6497 | No |
| 124 | SURF4 |  |  | 5631 | 2.100 | 0.6415 | No |
| 125 | TAF1A |  |  | 5949 | 1.700 | 0.6221 | No |
| 126 | SUPT7L |  |  | 6259 | 1.300 | 0.6030 | No |
| 127 | KDELR2 |  |  | 6538 | 0.900 | 0.5857 | No |
| 128 | CREM |  |  | 6654 | 0.700 | 0.5787 | No |
| 129 | PLEKHG2 |  |  | 7157 | 0.300 | 0.5467 | No |
| 130 | TMEM136 |  |  | 11148 | -0.200 | 0.2913 | No |
| 131 | PCDH17 |  |  | 11683 | -0.500 | 0.2574 | No |
| 132 | CDON |  |  | 11816 | -0.500 | 0.2492 | No |
| 133 | NANS |  |  | 12225 | -0.800 | 0.2235 | No |
| 134 | ARF4 |  |  | 12446 | -1.000 | 0.2100 | No |
| 135 | RHBDL2 |  |  | 13972 | -2.700 | 0.1138 | No |
Table: GSEA details [plain text format]

  

Fig 2: WINNEPENNINCKX\_MELANOMA\_METASTASIS\_UP: Random ES distribution      
 Gene set null distribution of ES for **WINNEPENNINCKX\_MELANOMA\_METASTASIS\_UP**

  
