# Supplementary material for: Immunomic, genomic and transcriptomic characterization of CT26 colorectal carcinoma
Source: BMC Genomics. 2014 Mar 13;15(1):190. doi: 10.1186/1471-2164-15-190 (PMC4007559; doi:10.1186/1471-2164-15-190)
Supplement: Supplementary file 8 — Additional file 8: Contains the Gene Pattern gene set membership and enrichment values in an html format. The file index.html is the entry point. (ZIP 13 MB) [file 12864_2013_7028_MOESM8_ESM.zip › gsea_report_for_na_neg_1374178656685.html]

Report for na\_neg 1374178656685 [GSEA]

| GS  follow link to MSigDB | GS DETAILS | SIZE | ES | NES | NOM p-val | FDR q-val | FWER p-val | RANK AT MAX | LEADING EDGE || 1 | KEGG\_CYTOKINE\_CYTOKINE\_RECEPTOR\_INTERACTION | Details ... | 224 | -0.27 |  |  | 1.000 | 0.000 | 2231 | tags=18%, list=14%, signal=21% |
| 2 | KEGG\_NEUROACTIVE\_LIGAND\_RECEPTOR\_INTERACTION | Details ... | 247 | -0.39 |  |  | 1.000 | 0.000 | 4358 | tags=31%, list=28%, signal=42% |
| 3 | KEGG\_LYSOSOME | Details ... | 117 | -0.23 |  |  | 1.000 | 0.000 | 1746 | tags=25%, list=11%, signal=28% |
| 4 | REACTOME\_PHOSPHOLIPID\_METABOLISM | Details ... | 157 | -0.24 |  |  | 1.000 | 0.000 | 1503 | tags=23%, list=10%, signal=25% |
| 5 | REACTOME\_BIOLOGICAL\_OXIDATIONS | Details ... | 117 | -0.28 |  |  | 1.000 | 0.000 | 1745 | tags=23%, list=11%, signal=26% |
| 6 | REACTOME\_PEPTIDE\_LIGAND\_BINDING\_RECEPTORS | Details ... | 162 | -0.51 |  |  | 1.000 | 0.000 | 3697 | tags=36%, list=23%, signal=47% |
| 7 | REACTOME\_CLASS\_A1\_RHODOPSIN\_LIKE\_RECEPTORS | Details ... | 257 | -0.55 |  |  | 1.000 | 0.000 | 3697 | tags=35%, list=23%, signal=45% |
| 8 | REACTOME\_G\_ALPHA\_Q\_SIGNALLING\_EVENTS | Details ... | 164 | -0.32 |  |  | 1.000 | 0.000 | 3596 | tags=34%, list=23%, signal=44% |
| 9 | REACTOME\_G\_ALPHA\_I\_SIGNALLING\_EVENTS | Details ... | 163 | -0.34 |  |  | 1.000 | 0.000 | 3055 | tags=28%, list=19%, signal=34% |
| 10 | REACTOME\_G\_ALPHA\_S\_SIGNALLING\_EVENTS | Details ... | 116 | -0.41 |  |  | 1.000 | 0.000 | 3053 | tags=26%, list=19%, signal=32% |
| 11 | REACTOME\_GPCR\_LIGAND\_BINDING | Details ... | 353 | -0.46 |  |  | 1.000 | 0.000 | 3613 | tags=33%, list=23%, signal=41% |
| 12 | KIM\_RESPONSE\_TO\_TSA\_AND\_DECITABINE\_UP | Details ... | 114 | -0.45 |  |  | 1.000 | 0.000 | 2372 | tags=43%, list=15%, signal=50% |
| 13 | SENGUPTA\_NASOPHARYNGEAL\_CARCINOMA\_DN | Details ... | 240 | -0.46 |  |  | 1.000 | 0.000 | 1787 | tags=26%, list=11%, signal=29% |
| 14 | TURASHVILI\_BREAST\_DUCTAL\_CARCINOMA\_VS\_DUCTAL\_NORMAL\_DN | Details ... | 159 | -0.41 |  |  | 1.000 | 0.000 | 1447 | tags=25%, list=9%, signal=27% |
| 15 | HUTTMANN\_B\_CLL\_POOR\_SURVIVAL\_UP | Details ... | 235 | -0.26 |  |  | 1.000 | 0.000 | 1828 | tags=27%, list=12%, signal=30% |
| 16 | CHARAFE\_BREAST\_CANCER\_LUMINAL\_VS\_BASAL\_UP | Details ... | 303 | -0.30 |  |  | 1.000 | 0.000 | 1788 | tags=30%, list=11%, signal=33% |
| 17 | CHARAFE\_BREAST\_CANCER\_LUMINAL\_VS\_MESENCHYMAL\_UP | Details ... | 363 | -0.51 |  |  | 1.000 | 0.000 | 1888 | tags=44%, list=12%, signal=49% |
| 18 | RODRIGUES\_NTN1\_TARGETS\_DN | Details ... | 139 | -0.30 |  |  | 1.000 | 0.000 | 1297 | tags=30%, list=8%, signal=33% |
| 19 | VECCHI\_GASTRIC\_CANCER\_ADVANCED\_VS\_EARLY\_DN | Details ... | 111 | -0.58 |  |  | 1.000 | 0.000 | 1032 | tags=41%, list=7%, signal=44% |
| 20 | JAEGER\_METASTASIS\_DN | Details ... | 231 | -0.58 |  |  | 1.000 | 0.000 | 1923 | tags=33%, list=12%, signal=37% |
| 21 | KINSEY\_TARGETS\_OF\_EWSR1\_FLII\_FUSION\_DN |  | 280 | -0.27 |  |  | 1.000 | 0.000 | 2263 | tags=34%, list=14%, signal=39% |
| 22 | SABATES\_COLORECTAL\_ADENOMA\_DN |  | 240 | -0.54 |  |  | 1.000 | 0.000 | 2572 | tags=40%, list=16%, signal=47% |
| 23 | JAATINEN\_HEMATOPOIETIC\_STEM\_CELL\_DN |  | 193 | -0.28 |  |  | 1.000 | 0.000 | 3005 | tags=33%, list=19%, signal=40% |
| 24 | WAMUNYOKOLI\_OVARIAN\_CANCER\_LMP\_UP |  | 218 | -0.34 |  |  | 1.000 | 0.000 | 1790 | tags=44%, list=11%, signal=49% |
| 25 | COLDREN\_GEFITINIB\_RESISTANCE\_DN |  | 189 | -0.53 |  |  | 1.000 | 0.000 | 1556 | tags=37%, list=10%, signal=41% |
| 26 | DELYS\_THYROID\_CANCER\_DN |  | 205 | -0.29 |  |  | 1.000 | 0.000 | 1732 | tags=22%, list=11%, signal=24% |
| 27 | CHIARADONNA\_NEOPLASTIC\_TRANSFORMATION\_KRAS\_DN |  | 138 | -0.33 |  |  | 1.000 | 0.000 | 1818 | tags=40%, list=12%, signal=45% |
| 28 | GAUSSMANN\_MLL\_AF4\_FUSION\_TARGETS\_F\_UP |  | 170 | -0.40 |  |  | 1.000 | 0.000 | 1632 | tags=29%, list=10%, signal=32% |
| 29 | GAUSSMANN\_MLL\_AF4\_FUSION\_TARGETS\_G\_UP |  | 195 | -0.30 |  |  | 1.000 | 0.000 | 1347 | tags=19%, list=9%, signal=21% |
| 30 | MCBRYAN\_PUBERTAL\_BREAST\_3\_4WK\_UP |  | 194 | -0.37 |  |  | 1.000 | 0.000 | 1423 | tags=33%, list=9%, signal=36% |
| 31 | MCBRYAN\_PUBERTAL\_BREAST\_4\_5WK\_UP |  | 246 | -0.35 |  |  | 1.000 | 0.000 | 1390 | tags=32%, list=9%, signal=35% |
| 32 | HAMAI\_APOPTOSIS\_VIA\_TRAIL\_DN |  | 158 | -0.42 |  |  | 1.000 | 0.000 | 1954 | tags=32%, list=12%, signal=36% |
| 33 | PEREZ\_TP63\_TARGETS |  | 302 | -0.32 |  |  | 1.000 | 0.000 | 2806 | tags=38%, list=18%, signal=46% |
| 34 | PEREZ\_TP53\_AND\_TP63\_TARGETS |  | 178 | -0.40 |  |  | 1.000 | 0.000 | 2539 | tags=40%, list=16%, signal=47% |
| 35 | SHETH\_LIVER\_CANCER\_VS\_TXNIP\_LOSS\_PAM4 |  | 233 | -0.28 |  |  | 1.000 | 0.000 | 861 | tags=15%, list=5%, signal=15% |
| 36 | RICKMAN\_METASTASIS\_DN |  | 227 | -0.27 |  |  | 1.000 | 0.000 | 2080 | tags=40%, list=13%, signal=46% |
| 37 | SCHAEFFER\_PROSTATE\_DEVELOPMENT\_48HR\_UP |  | 421 | -0.35 |  |  | 1.000 | 0.000 | 1831 | tags=29%, list=12%, signal=32% |
| 38 | WU\_CELL\_MIGRATION |  | 163 | -0.31 |  |  | 1.000 | 0.000 | 1434 | tags=33%, list=9%, signal=36% |
| 39 | SHEN\_SMARCA2\_TARGETS\_DN |  | 283 | -0.25 |  |  | 1.000 | 0.000 | 1307 | tags=11%, list=8%, signal=11% |
| 40 | NIKOLSKY\_BREAST\_CANCER\_11Q12\_Q14\_AMPLICON |  | 134 | -0.26 |  |  | 1.000 | 0.000 | 2193 | tags=31%, list=14%, signal=36% |
| 41 | ONDER\_CDH1\_TARGETS\_2\_DN |  | 413 | -0.43 |  |  | 1.000 | 0.000 | 1923 | tags=34%, list=12%, signal=38% |
| 42 | SANSOM\_APC\_TARGETS\_DN |  | 347 | -0.43 |  |  | 1.000 | 0.000 | 2399 | tags=41%, list=15%, signal=47% |
| 43 | HSIAO\_LIVER\_SPECIFIC\_GENES |  | 222 | -0.33 |  |  | 1.000 | 0.000 | 1158 | tags=19%, list=7%, signal=21% |
| 44 | AFFAR\_YY1\_TARGETS\_UP |  | 200 | -0.32 |  |  | 1.000 | 0.000 | 1835 | tags=28%, list=12%, signal=31% |
| 45 | MOREAUX\_MULTIPLE\_MYELOMA\_BY\_TACI\_UP |  | 345 | -0.22 |  |  | 1.000 | 0.000 | 1901 | tags=22%, list=12%, signal=25% |
| 46 | SATO\_SILENCED\_BY\_METHYLATION\_IN\_PANCREATIC\_CANCER\_1 |  | 360 | -0.27 |  |  | 1.000 | 0.000 | 1870 | tags=22%, list=12%, signal=24% |
| 47 | LEIN\_CHOROID\_PLEXUS\_MARKERS |  | 92 | -0.43 |  |  | 1.000 | 0.000 | 1760 | tags=29%, list=11%, signal=33% |
| 48 | RIGGI\_EWING\_SARCOMA\_PROGENITOR\_UP |  | 372 | -0.25 |  |  | 1.000 | 0.000 | 2042 | tags=22%, list=13%, signal=24% |
| 49 | SMID\_BREAST\_CANCER\_LUMINAL\_B\_UP |  | 150 | -0.32 |  |  | 1.000 | 0.000 | 2118 | tags=23%, list=13%, signal=27% |
| 50 | SMID\_BREAST\_CANCER\_LUMINAL\_A\_UP |  | 76 | -0.56 |  |  | 1.000 | 0.000 | 2698 | tags=50%, list=17%, signal=60% |
| 51 | SMID\_BREAST\_CANCER\_NORMAL\_LIKE\_UP |  | 409 | -0.35 |  |  | 1.000 | 0.000 | 1955 | tags=24%, list=12%, signal=27% |
| 52 | BOQUEST\_STEM\_CELL\_DN |  | 186 | -0.33 |  |  | 1.000 | 0.000 | 1194 | tags=19%, list=8%, signal=21% |
| 53 | LEE\_LIVER\_CANCER\_SURVIVAL\_UP |  | 150 | -0.50 |  |  | 1.000 | 0.000 | 1842 | tags=26%, list=12%, signal=29% |
| 54 | ZHANG\_TLX\_TARGETS\_60HR\_UP |  | 273 | -0.26 |  |  | 1.000 | 0.000 | 1811 | tags=30%, list=11%, signal=33% |
| 55 | MEISSNER\_NPC\_HCP\_WITH\_H3\_UNMETHYLATED |  | 450 | -0.53 |  |  | 1.000 | 0.000 | 1831 | tags=26%, list=12%, signal=28% |
| 56 | MEISSNER\_BRAIN\_HCP\_WITH\_H3K27ME3 |  | 243 | -0.53 |  |  | 1.000 | 0.000 | 2239 | tags=23%, list=14%, signal=26% |
| 57 | POOLA\_INVASIVE\_BREAST\_CANCER\_DN |  | 125 | -0.38 |  |  | 1.000 | 0.000 | 944 | tags=21%, list=6%, signal=22% |
| 58 | CHIANG\_LIVER\_CANCER\_SUBCLASS\_CTNNB1\_DN |  | 147 | -0.34 |  |  | 1.000 | 0.000 | 1570 | tags=24%, list=10%, signal=27% |
| 59 | CHIANG\_LIVER\_CANCER\_SUBCLASS\_PROLIFERATION\_DN |  | 158 | -0.45 |  |  | 1.000 | 0.000 | 2406 | tags=30%, list=15%, signal=35% |
| 60 | COULOUARN\_TEMPORAL\_TGFB1\_SIGNATURE\_DN |  | 127 | -0.34 |  |  | 1.000 | 0.000 | 1559 | tags=31%, list=10%, signal=35% |
| 61 | MEISSNER\_NPC\_HCP\_WITH\_H3K4ME2\_AND\_H3K27ME3 |  | 311 | -0.48 |  |  | 1.000 | 0.000 | 3188 | tags=28%, list=20%, signal=34% |
| 62 | MEISSNER\_NPC\_HCP\_WITH\_H3K4ME2 |  | 427 | -0.36 |  |  | 1.000 | 0.000 | 2943 | tags=37%, list=19%, signal=44% |
| 63 | MIKKELSEN\_MCV6\_HCP\_WITH\_H3K27ME3 |  | 387 | -0.41 |  |  | 1.000 | 0.000 | 3889 | tags=35%, list=25%, signal=46% |
| 64 | MIKKELSEN\_MCV6\_LCP\_WITH\_H3K4ME3 |  | 139 | -0.30 |  |  | 1.000 | 0.000 | 1172 | tags=22%, list=7%, signal=23% |
| 65 | MIKKELSEN\_MEF\_ICP\_WITH\_H3K27ME3 |  | 172 | -0.48 |  |  | 1.000 | 0.000 | 2617 | tags=18%, list=17%, signal=21% |
| 66 | MIKKELSEN\_IPS\_ICP\_WITH\_H3K4ME3\_AND\_H327ME3 |  | 109 | -0.35 |  |  | 1.000 | 0.000 | 2530 | tags=25%, list=16%, signal=29% |
| 67 | MIKKELSEN\_IPS\_LCP\_WITH\_H3K4ME3 |  | 140 | -0.34 |  |  | 1.000 | 0.000 | 2363 | tags=33%, list=15%, signal=38% |
| 68 | HOSHIDA\_LIVER\_CANCER\_SUBCLASS\_S3 |  | 249 | -0.32 |  |  | 1.000 | 0.000 | 908 | tags=18%, list=6%, signal=19% |
| 69 | CAIRO\_HEPATOBLASTOMA\_DN |  | 244 | -0.28 |  |  | 1.000 | 0.000 | 1072 | tags=16%, list=7%, signal=17% |
| 70 | CAIRO\_LIVER\_DEVELOPMENT\_DN |  | 216 | -0.43 |  |  | 1.000 | 0.000 | 1342 | tags=26%, list=9%, signal=28% |
| 71 | MIKKELSEN\_ES\_ICP\_WITH\_H3K4ME3\_AND\_H3K27ME3 |  | 118 | -0.30 |  |  | 1.000 | 0.000 | 1102 | tags=13%, list=7%, signal=14% |
| 72 | MIKKELSEN\_ES\_LCP\_WITH\_H3K4ME3 |  | 108 | -0.45 |  |  | 1.000 | 0.000 | 2363 | tags=37%, list=15%, signal=43% |
| 73 | MIKKELSEN\_NPC\_HCP\_WITH\_H3K27ME3 |  | 301 | -0.53 |  |  | 1.000 | 0.000 | 2259 | tags=22%, list=14%, signal=25% |
| 74 | MIKKELSEN\_NPC\_HCP\_WITH\_H3K4ME3\_AND\_H3K27ME3 |  | 189 | -0.28 |  |  | 1.000 | 0.000 | 2310 | tags=23%, list=15%, signal=27% |
| 75 | MIKKELSEN\_MEF\_HCP\_WITH\_H3\_UNMETHYLATED |  | 187 | -0.36 |  |  | 1.000 | 0.000 | 3357 | tags=20%, list=21%, signal=25% |
| 76 | SENGUPTA\_EBNA1\_ANTICORRELATED |  | 128 | -0.33 |  |  | 1.000 | 0.000 | 2235 | tags=33%, list=14%, signal=38% |
| 77 | CHYLA\_CBFA2T3\_TARGETS\_DN |  | 193 | -0.33 |  |  | 1.000 | 0.000 | 2175 | tags=28%, list=14%, signal=32% |
| 78 | ACOSTA\_PROLIFERATION\_INDEPENDENT\_MYC\_TARGETS\_DN |  | 102 | -0.31 |  |  | 1.000 | 0.000 | 2161 | tags=32%, list=14%, signal=37% |
| 79 | KATSANOU\_ELAVL1\_TARGETS\_UP |  | 149 | -0.37 |  |  | 1.000 | 0.000 | 1835 | tags=33%, list=12%, signal=37% |
| 80 | SERVITJA\_ISLET\_HNF1A\_TARGETS\_DN |  | 100 | -0.56 |  |  | 1.000 | 0.000 | 1837 | tags=37%, list=12%, signal=42% |
| 81 | SERVITJA\_LIVER\_HNF1A\_TARGETS\_DN |  | 148 | -0.29 |  |  | 1.000 | 0.000 | 1428 | tags=14%, list=9%, signal=15% |
| 82 | WANG\_MLL\_TARGETS |  | 261 | -0.27 |  |  | 1.000 | 0.000 | 1783 | tags=23%, list=11%, signal=25% |
| 83 | FOSTER\_KDM1A\_TARGETS\_UP |  | 225 | -0.28 |  |  | 1.000 | 0.000 | 1686 | tags=16%, list=11%, signal=18% |
| 84 | ACEVEDO\_FGFR1\_TARGETS\_IN\_PROSTATE\_CANCER\_MODEL\_DN |  | 274 | -0.35 |  |  | 1.000 | 0.000 | 1075 | tags=21%, list=7%, signal=22% |
| 85 | LIM\_MAMMARY\_STEM\_CELL\_DN |  | 383 | -0.46 |  |  | 1.000 | 0.000 | 1703 | tags=37%, list=11%, signal=40% |
| 86 | DURAND\_STROMA\_MAX\_UP |  | 274 | -0.36 |  |  | 1.000 | 0.000 | 2231 | tags=31%, list=14%, signal=36% |
| 87 | ZWANG\_EGF\_INTERVAL\_DN |  | 179 | -0.35 |  |  | 1.000 | 0.000 | 2312 | tags=33%, list=15%, signal=38% |
| 88 | AIGNER\_ZEB1\_TARGETS |  | 31 | -0.85 | -2.72 | 0.000 | 0.000 | 0.000 | 1696 | tags=65%, list=11%, signal=72% |
| 89 | WEBER\_METHYLATED\_LCP\_IN\_SPERM\_UP |  | 15 | -0.96 | -2.47 | 0.000 | 0.000 | 0.000 | 198 | tags=7%, list=1%, signal=7% |
| 90 | CHARAFE\_BREAST\_CANCER\_BASAL\_VS\_MESENCHYMAL\_UP |  | 99 | -0.63 | -2.41 | 0.000 | 0.000 | 0.000 | 1696 | tags=45%, list=11%, signal=51% |
| 91 | WANG\_CLASSIC\_ADIPOGENIC\_TARGETS\_OF\_PPARG |  | 23 | -0.79 | -2.37 | 0.000 | 0.000 | 0.000 | 2900 | tags=74%, list=18%, signal=90% |
| 92 | MIKKELSEN\_MEF\_LCP\_WITH\_H3K27ME3 |  | 58 | -0.65 | -2.35 | 0.000 | 0.000 | 0.000 | 1755 | tags=17%, list=11%, signal=19% |
| 93 | RICKMAN\_HEAD\_AND\_NECK\_CANCER\_D |  | 25 | -0.77 | -2.35 | 0.000 | 0.000 | 0.000 | 1307 | tags=44%, list=8%, signal=48% |
| 94 | KEGG\_AUTOIMMUNE\_THYROID\_DISEASE |  | 33 | -0.74 | -2.34 | 0.000 | 0.000 | 0.000 | 2307 | tags=27%, list=15%, signal=32% |
| 95 | LIU\_CDX2\_TARGETS\_UP |  | 34 | -0.70 | -2.29 | 0.000 | 0.000 | 0.000 | 2245 | tags=59%, list=14%, signal=68% |
| 96 | MCGARVEY\_SILENCED\_BY\_METHYLATION\_IN\_COLON\_CANCER |  | 41 | -0.68 | -2.28 | 0.000 | 0.000 | 0.002 | 2540 | tags=34%, list=16%, signal=41% |
| 97 | LIEN\_BREAST\_CARCINOMA\_METAPLASTIC\_VS\_DUCTAL\_DN |  | 84 | -0.61 | -2.28 | 0.000 | 0.000 | 0.002 | 2066 | tags=43%, list=13%, signal=49% |
| 98 | BIOCARTA\_TH1TH2\_PATHWAY |  | 18 | -0.85 | -2.25 | 0.000 | 0.000 | 0.005 | 1397 | tags=28%, list=9%, signal=30% |
| 99 | KEGG\_ASTHMA |  | 22 | -0.81 | -2.25 | 0.000 | 0.000 | 0.005 | 2307 | tags=32%, list=15%, signal=37% |
| 100 | KEGG\_GRAFT\_VERSUS\_HOST\_DISEASE |  | 26 | -0.74 | -2.24 | 0.000 | 0.000 | 0.005 | 2307 | tags=35%, list=15%, signal=40% |
| 101 | REACTOME\_VOLTAGE\_GATED\_POTASSIUM\_CHANNELS |  | 43 | -0.67 | -2.22 | 0.000 | 0.000 | 0.008 | 4440 | tags=49%, list=28%, signal=68% |
| 102 | ROY\_WOUND\_BLOOD\_VESSEL\_DN |  | 16 | -0.87 | -2.20 | 0.000 | 0.000 | 0.010 | 1289 | tags=63%, list=8%, signal=68% |
| 103 | KEGG\_ALLOGRAFT\_REJECTION |  | 27 | -0.73 | -2.20 | 0.000 | 0.000 | 0.010 | 2307 | tags=33%, list=15%, signal=39% |
| 104 | REACTOME\_AMINE\_LIGAND\_BINDING\_RECEPTORS |  | 36 | -0.67 | -2.17 | 0.000 | 0.000 | 0.013 | 3908 | tags=22%, list=25%, signal=29% |
| 105 | LOPEZ\_EPITHELIOID\_MESOTHELIOMA |  | 16 | -0.82 | -2.15 | 0.000 | 0.001 | 0.016 | 1921 | tags=44%, list=12%, signal=50% |
| 106 | BOSCO\_EPITHELIAL\_DIFFERENTIATION\_MODULE |  | 53 | -0.62 | -2.15 | 0.000 | 0.001 | 0.017 | 1289 | tags=25%, list=8%, signal=27% |
| 107 | BIOCARTA\_CYTOKINE\_PATHWAY |  | 19 | -0.75 | -2.14 | 0.000 | 0.001 | 0.017 | 402 | tags=5%, list=3%, signal=5% |
| 108 | WANG\_BARRETTS\_ESOPHAGUS\_UP |  | 46 | -0.62 | -2.13 | 0.000 | 0.001 | 0.021 | 1333 | tags=43%, list=8%, signal=47% |
| 109 | WU\_SILENCED\_BY\_METHYLATION\_IN\_BLADDER\_CANCER |  | 49 | -0.58 | -2.12 | 0.000 | 0.001 | 0.022 | 2511 | tags=53%, list=16%, signal=63% |
| 110 | NIELSEN\_LEIOMYOSARCOMA\_CNN1\_UP |  | 18 | -0.82 | -2.12 | 0.000 | 0.001 | 0.022 | 793 | tags=44%, list=5%, signal=47% |
| 111 | WANG\_BARRETTS\_ESOPHAGUS\_AND\_ESOPHAGUS\_CANCER\_UP |  | 24 | -0.72 | -2.11 | 0.000 | 0.001 | 0.024 | 1677 | tags=46%, list=11%, signal=51% |
| 112 | VILIMAS\_NOTCH1\_TARGETS\_DN |  | 20 | -0.72 | -2.07 | 0.000 | 0.002 | 0.052 | 2560 | tags=40%, list=16%, signal=48% |
| 113 | MEISSNER\_NPC\_HCP\_WITH\_H3K27ME3 |  | 69 | -0.54 | -2.06 | 0.000 | 0.002 | 0.052 | 2596 | tags=28%, list=16%, signal=33% |
| 114 | VANDESLUIS\_COMMD1\_TARGETS\_GROUP\_4\_UP |  | 19 | -0.75 | -2.06 | 0.000 | 0.002 | 0.053 | 488 | tags=37%, list=3%, signal=38% |
| 115 | MISHRA\_CARCINOMA\_ASSOCIATED\_FIBROBLAST\_DN |  | 23 | -0.69 | -2.05 | 0.000 | 0.002 | 0.057 | 440 | tags=48%, list=3%, signal=49% |
| 116 | NAKAYAMA\_SOFT\_TISSUE\_TUMORS\_PCA2\_DN |  | 69 | -0.53 | -2.03 | 0.000 | 0.002 | 0.067 | 2698 | tags=54%, list=17%, signal=64% |
| 117 | MCMURRAY\_TP53\_HRAS\_COOPERATION\_RESPONSE\_DN |  | 64 | -0.56 | -2.03 | 0.000 | 0.002 | 0.068 | 1983 | tags=31%, list=13%, signal=36% |
| 118 | WEST\_ADRENOCORTICAL\_TUMOR\_MARKERS\_DN |  | 19 | -0.73 | -2.02 | 0.000 | 0.002 | 0.088 | 2231 | tags=63%, list=14%, signal=73% |
| 119 | TUOMISTO\_TUMOR\_SUPPRESSION\_BY\_COL13A1\_DN |  | 15 | -0.80 | -2.01 | 0.000 | 0.003 | 0.090 | 488 | tags=20%, list=3%, signal=21% |
| 120 | DOANE\_BREAST\_CANCER\_CLASSES\_UP |  | 63 | -0.54 | -2.00 | 0.000 | 0.003 | 0.096 | 2597 | tags=44%, list=16%, signal=53% |
| 121 | LEE\_LIVER\_CANCER |  | 42 | -0.57 | -1.99 | 0.000 | 0.003 | 0.110 | 884 | tags=19%, list=6%, signal=20% |
| 122 | REACTOME\_CHEMOKINE\_RECEPTORS\_BIND\_CHEMOKINES |  | 44 | -0.64 | -1.98 | 0.000 | 0.003 | 0.119 | 3682 | tags=45%, list=23%, signal=59% |
| 123 | SMID\_BREAST\_CANCER\_RELAPSE\_IN\_LUNG\_DN |  | 33 | -0.63 | -1.97 | 0.000 | 0.004 | 0.133 | 2279 | tags=45%, list=14%, signal=53% |
| 124 | NIELSEN\_SCHWANNOMA\_UP |  | 15 | -0.78 | -1.97 | 0.009 | 0.004 | 0.134 | 1500 | tags=33%, list=10%, signal=37% |
| 125 | REACTOME\_DEFENSINS |  | 21 | -0.69 | -1.97 | 0.000 | 0.004 | 0.135 | 4089 | tags=19%, list=26%, signal=26% |
| 126 | NAKAYAMA\_SOFT\_TISSUE\_TUMORS\_PCA1\_DN |  | 72 | -0.59 | -1.96 | 0.000 | 0.004 | 0.144 | 2891 | tags=47%, list=18%, signal=58% |
| 127 | LIM\_MAMMARY\_LUMINAL\_MATURE\_UP |  | 106 | -0.50 | -1.94 | 0.000 | 0.005 | 0.165 | 2409 | tags=46%, list=15%, signal=54% |
| 128 | SEKI\_INFLAMMATORY\_RESPONSE\_LPS\_DN |  | 22 | -0.68 | -1.94 | 0.000 | 0.005 | 0.166 | 1825 | tags=45%, list=12%, signal=51% |
| 129 | REACTOME\_BETA\_DEFENSINS |  | 15 | -0.74 | -1.94 | 0.017 | 0.005 | 0.169 | 2276 | tags=13%, list=14%, signal=16% |
| 130 | MEISSNER\_BRAIN\_HCP\_WITH\_H3\_UNMETHYLATED |  | 30 | -0.62 | -1.94 | 0.000 | 0.005 | 0.170 | 1687 | tags=13%, list=11%, signal=15% |
| 131 | REACTOME\_AMINE\_DERIVED\_HORMONES |  | 15 | -0.70 | -1.90 | 0.008 | 0.006 | 0.214 | 3874 | tags=33%, list=25%, signal=44% |
| 132 | BARRIER\_COLON\_CANCER\_RECURRENCE\_DN |  | 17 | -0.69 | -1.86 | 0.010 | 0.009 | 0.289 | 1362 | tags=59%, list=9%, signal=64% |
| 133 | ABBUD\_LIF\_SIGNALING\_1\_UP |  | 44 | -0.53 | -1.86 | 0.000 | 0.009 | 0.303 | 1416 | tags=34%, list=9%, signal=37% |
| 134 | KANG\_AR\_TARGETS\_UP |  | 17 | -0.71 | -1.85 | 0.019 | 0.009 | 0.309 | 1978 | tags=41%, list=13%, signal=47% |
| 135 | REACTOME\_TERMINATION\_OF\_O\_GLYCAN\_BIOSYNTHESIS |  | 17 | -0.71 | -1.85 | 0.000 | 0.009 | 0.318 | 1781 | tags=47%, list=11%, signal=53% |
| 136 | KEGG\_MATURITY\_ONSET\_DIABETES\_OF\_THE\_YOUNG |  | 21 | -0.68 | -1.83 | 0.000 | 0.011 | 0.358 | 1896 | tags=29%, list=12%, signal=32% |
| 137 | KEGG\_INTESTINAL\_IMMUNE\_NETWORK\_FOR\_IGA\_PRODUCTION |  | 40 | -0.54 | -1.83 | 0.000 | 0.011 | 0.360 | 2404 | tags=35%, list=15%, signal=41% |
| 138 | OSADA\_ASCL1\_TARGETS\_UP |  | 45 | -0.54 | -1.83 | 0.000 | 0.011 | 0.365 | 2118 | tags=42%, list=13%, signal=49% |
| 139 | WINNEPENNINCKX\_MELANOMA\_METASTASIS\_DN |  | 39 | -0.57 | -1.83 | 0.000 | 0.011 | 0.371 | 2372 | tags=36%, list=15%, signal=42% |
| 140 | CROMER\_TUMORIGENESIS\_DN |  | 45 | -0.55 | -1.82 | 0.000 | 0.011 | 0.381 | 1255 | tags=33%, list=8%, signal=36% |
| 141 | VERNOCHET\_ADIPOGENESIS |  | 17 | -0.68 | -1.82 | 0.000 | 0.011 | 0.383 | 1208 | tags=59%, list=8%, signal=64% |
| 142 | REACTOME\_CYTOCHROME\_P450\_ARRANGED\_BY\_SUBSTRATE\_TYPE |  | 48 | -0.54 | -1.82 | 0.000 | 0.011 | 0.386 | 2354 | tags=29%, list=15%, signal=34% |
| 143 | BOQUEST\_STEM\_CELL\_CULTURED\_VS\_FRESH\_DN |  | 28 | -0.61 | -1.82 | 0.000 | 0.011 | 0.387 | 1219 | tags=25%, list=8%, signal=27% |
| 144 | SATO\_SILENCED\_EPIGENETICALLY\_IN\_PANCREATIC\_CANCER |  | 38 | -0.58 | -1.81 | 0.000 | 0.012 | 0.407 | 1850 | tags=32%, list=12%, signal=36% |
| 145 | BIOCARTA\_NKT\_PATHWAY |  | 27 | -0.61 | -1.79 | 0.000 | 0.013 | 0.440 | 1397 | tags=15%, list=9%, signal=16% |
| 146 | PID\_HNF3BPATHWAY |  | 42 | -0.52 | -1.79 | 0.000 | 0.013 | 0.455 | 1710 | tags=38%, list=11%, signal=43% |
| 147 | ROLEF\_GLIS3\_TARGETS |  | 35 | -0.55 | -1.77 | 0.000 | 0.015 | 0.491 | 1790 | tags=29%, list=11%, signal=32% |
| 148 | LUCAS\_HNF4A\_TARGETS\_UP |  | 51 | -0.49 | -1.75 | 0.000 | 0.018 | 0.555 | 1702 | tags=39%, list=11%, signal=44% |
| 149 | MADAN\_DPPA4\_TARGETS |  | 38 | -0.51 | -1.74 | 0.000 | 0.018 | 0.570 | 1793 | tags=16%, list=11%, signal=18% |
| 150 | MEISSNER\_BRAIN\_HCP\_WITH\_H3K4ME2 |  | 15 | -0.67 | -1.73 | 0.000 | 0.019 | 0.584 | 3165 | tags=47%, list=20%, signal=58% |
| 151 | SMID\_BREAST\_CANCER\_RELAPSE\_IN\_BRAIN\_DN |  | 70 | -0.45 | -1.72 | 0.000 | 0.020 | 0.614 | 951 | tags=24%, list=6%, signal=26% |
| 152 | GAURNIER\_PSMD4\_TARGETS |  | 57 | -0.51 | -1.71 | 0.000 | 0.022 | 0.650 | 2307 | tags=33%, list=15%, signal=39% |
| 153 | OHGUCHI\_LIVER\_HNF4A\_TARGETS\_DN |  | 133 | -0.42 | -1.69 | 0.000 | 0.024 | 0.684 | 1237 | tags=18%, list=8%, signal=19% |
| 154 | REACTOME\_LIGAND\_GATED\_ION\_CHANNEL\_TRANSPORT |  | 20 | -0.59 | -1.68 | 0.000 | 0.026 | 0.724 | 1935 | tags=5%, list=12%, signal=6% |
| 155 | REACTOME\_ION\_CHANNEL\_TRANSPORT |  | 53 | -0.47 | -1.67 | 0.000 | 0.027 | 0.733 | 958 | tags=13%, list=6%, signal=14% |
| 156 | REACTOME\_TIGHT\_JUNCTION\_INTERACTIONS |  | 29 | -0.53 | -1.67 | 0.000 | 0.028 | 0.751 | 846 | tags=31%, list=5%, signal=33% |
| 157 | DOANE\_BREAST\_CANCER\_ESR1\_UP |  | 99 | -0.41 | -1.67 | 0.000 | 0.028 | 0.753 | 2767 | tags=39%, list=18%, signal=47% |
| 158 | KIM\_GLIS2\_TARGETS\_UP |  | 82 | -0.50 | -1.67 | 0.000 | 0.028 | 0.755 | 2045 | tags=49%, list=13%, signal=56% |
| 159 | KEGG\_GLYCOSPHINGOLIPID\_BIOSYNTHESIS\_LACTO\_AND\_NEOLACTO\_SERIES |  | 23 | -0.59 | -1.66 | 0.025 | 0.028 | 0.768 | 2428 | tags=39%, list=15%, signal=46% |
| 160 | KOBAYASHI\_EGFR\_SIGNALING\_24HR\_UP |  | 81 | -0.44 | -1.66 | 0.000 | 0.028 | 0.772 | 2068 | tags=40%, list=13%, signal=45% |
| 161 | KEGG\_STEROID\_HORMONE\_BIOSYNTHESIS |  | 45 | -0.50 | -1.66 | 0.000 | 0.029 | 0.778 | 2900 | tags=33%, list=18%, signal=41% |
| 162 | WONG\_ENDMETRIUM\_CANCER\_UP |  | 20 | -0.58 | -1.65 | 0.013 | 0.031 | 0.796 | 1495 | tags=35%, list=9%, signal=39% |
| 163 | GOLUB\_ALL\_VS\_AML\_DN |  | 21 | -0.61 | -1.64 | 0.013 | 0.032 | 0.815 | 1762 | tags=48%, list=11%, signal=54% |
| 164 | ONDER\_CDH1\_SIGNALING\_VIA\_CTNNB1 |  | 72 | -0.43 | -1.64 | 0.000 | 0.032 | 0.825 | 1983 | tags=42%, list=13%, signal=47% |
| 165 | LI\_ADIPOGENESIS\_BY\_ACTIVATED\_PPARG |  | 16 | -0.61 | -1.63 | 0.020 | 0.034 | 0.839 | 1797 | tags=63%, list=11%, signal=70% |
| 166 | GU\_PDEF\_TARGETS\_DN |  | 31 | -0.53 | -1.63 | 0.000 | 0.034 | 0.841 | 1060 | tags=29%, list=7%, signal=31% |
| 167 | REACTOME\_REGULATION\_OF\_INSULIN\_LIKE\_GROWTH\_FACTOR\_IGF\_ACTIVITY\_BY\_INSULIN\_LIKE\_GROWTH\_FACTOR\_BINDING\_PROTEINS\_IGFBPS |  | 16 | -0.62 | -1.63 | 0.039 | 0.034 | 0.845 | 1349 | tags=31%, list=9%, signal=34% |
| 168 | MEISSNER\_NPC\_ICP\_WITH\_H3\_UNMETHYLATED |  | 17 | -0.58 | -1.62 | 0.040 | 0.035 | 0.857 | 1102 | tags=24%, list=7%, signal=25% |
| 169 | TORCHIA\_TARGETS\_OF\_EWSR1\_FLI1\_FUSION\_TOP20\_UP |  | 17 | -0.61 | -1.61 | 0.010 | 0.037 | 0.871 | 347 | tags=18%, list=2%, signal=18% |
| 170 | HESS\_TARGETS\_OF\_HOXA9\_AND\_MEIS1\_DN |  | 72 | -0.45 | -1.61 | 0.000 | 0.037 | 0.875 | 1609 | tags=33%, list=10%, signal=37% |
| 171 | MIKKELSEN\_ES\_ICP\_WITH\_H3K27ME3 |  | 37 | -0.48 | -1.61 | 0.026 | 0.038 | 0.881 | 921 | tags=8%, list=6%, signal=9% |
| 172 | KEGG\_OTHER\_GLYCAN\_DEGRADATION |  | 15 | -0.61 | -1.60 | 0.032 | 0.038 | 0.884 | 1832 | tags=47%, list=12%, signal=53% |
| 173 | BIOCARTA\_INFLAM\_PATHWAY |  | 26 | -0.53 | -1.60 | 0.000 | 0.039 | 0.888 | 345 | tags=8%, list=2%, signal=8% |
| 174 | ZEMBUTSU\_SENSITIVITY\_TO\_CISPLATIN |  | 19 | -0.57 | -1.59 | 0.027 | 0.041 | 0.901 | 1142 | tags=42%, list=7%, signal=45% |
| 175 | REACTOME\_COMPLEMENT\_CASCADE |  | 24 | -0.57 | -1.58 | 0.022 | 0.042 | 0.906 | 688 | tags=21%, list=4%, signal=22% |
| 176 | SCHUETZ\_BREAST\_CANCER\_DUCTAL\_INVASIVE\_DN |  | 79 | -0.41 | -1.57 | 0.000 | 0.045 | 0.925 | 620 | tags=18%, list=4%, signal=18% |
| 177 | SCHLESINGER\_H3K27ME3\_IN\_NORMAL\_AND\_METHYLATED\_IN\_CANCER |  | 26 | -0.52 | -1.56 | 0.000 | 0.049 | 0.940 | 851 | tags=12%, list=5%, signal=12% |
| 178 | POTTI\_ETOPOSIDE\_SENSITIVITY |  | 37 | -0.46 | -1.55 | 0.000 | 0.051 | 0.945 | 1457 | tags=51%, list=9%, signal=56% |
| 179 | SCHLESINGER\_METHYLATED\_DE\_NOVO\_IN\_CANCER |  | 80 | -0.40 | -1.55 | 0.000 | 0.051 | 0.945 | 2733 | tags=38%, list=17%, signal=45% |
| 180 | TONKS\_TARGETS\_OF\_RUNX1\_RUNX1T1\_FUSION\_GRANULOCYTE\_DN |  | 16 | -0.59 | -1.55 | 0.021 | 0.051 | 0.945 | 3328 | tags=44%, list=21%, signal=55% |
| 181 | VALK\_AML\_CLUSTER\_5 |  | 26 | -0.52 | -1.55 | 0.014 | 0.051 | 0.951 | 2075 | tags=38%, list=13%, signal=44% |
| 182 | REACTOME\_AMYLOIDS |  | 52 | -0.42 | -1.54 | 0.000 | 0.053 | 0.953 | 3021 | tags=58%, list=19%, signal=71% |
| 183 | MEISSNER\_BRAIN\_HCP\_WITH\_H3K4ME2\_AND\_H3K27ME3 |  | 54 | -0.46 | -1.54 | 0.000 | 0.054 | 0.956 | 2299 | tags=39%, list=15%, signal=45% |
| 184 | NIELSEN\_GIST\_AND\_SYNOVIAL\_SARCOMA\_UP |  | 20 | -0.56 | -1.53 | 0.032 | 0.055 | 0.960 | 3571 | tags=60%, list=23%, signal=77% |
| 185 | HALMOS\_CEBPA\_TARGETS\_DN |  | 42 | -0.46 | -1.53 | 0.000 | 0.057 | 0.967 | 1208 | tags=26%, list=8%, signal=28% |
| 186 | KEGG\_CELL\_ADHESION\_MOLECULES\_CAMS |  | 119 | -0.37 | -1.52 | 0.000 | 0.058 | 0.969 | 1433 | tags=23%, list=9%, signal=25% |
| 187 | ZEMBUTSU\_SENSITIVITY\_TO\_CYCLOPHOSPHAMIDE |  | 16 | -0.59 | -1.51 | 0.050 | 0.061 | 0.977 | 1538 | tags=31%, list=10%, signal=35% |
| 188 | PID\_WNT\_SIGNALING\_PATHWAY |  | 28 | -0.49 | -1.50 | 0.021 | 0.065 | 0.980 | 1915 | tags=36%, list=12%, signal=41% |
| 189 | NIELSEN\_GIST\_VS\_SYNOVIAL\_SARCOMA\_DN |  | 17 | -0.55 | -1.50 | 0.019 | 0.067 | 0.984 | 1583 | tags=29%, list=10%, signal=33% |
| 190 | BOYAULT\_LIVER\_CANCER\_SUBCLASS\_G3\_DN |  | 47 | -0.42 | -1.49 | 0.000 | 0.067 | 0.985 | 2169 | tags=38%, list=14%, signal=44% |
| 191 | REACTOME\_CLASS\_B\_2\_SECRETIN\_FAMILY\_RECEPTORS |  | 82 | -0.37 | -1.49 | 0.000 | 0.067 | 0.986 | 1915 | tags=22%, list=12%, signal=25% |
| 192 | REACTOME\_EFFECTS\_OF\_PIP2\_HYDROLYSIS |  | 23 | -0.51 | -1.49 | 0.060 | 0.067 | 0.986 | 3243 | tags=39%, list=21%, signal=49% |
| 193 | BERTUCCI\_INVASIVE\_CARCINOMA\_DUCTAL\_VS\_LOBULAR\_DN |  | 44 | -0.40 | -1.49 | 0.000 | 0.069 | 0.987 | 1889 | tags=43%, list=12%, signal=49% |
| 194 | MURATA\_VIRULENCE\_OF\_H\_PILORI |  | 22 | -0.50 | -1.49 | 0.015 | 0.069 | 0.988 | 529 | tags=27%, list=3%, signal=28% |
| 195 | MAHADEVAN\_IMATINIB\_RESISTANCE\_DN |  | 17 | -0.56 | -1.48 | 0.070 | 0.071 | 0.990 | 2093 | tags=59%, list=13%, signal=68% |
| 196 | VANDESLUIS\_COMMD1\_TARGETS\_GROUP\_3\_UP |  | 81 | -0.36 | -1.48 | 0.000 | 0.071 | 0.990 | 1309 | tags=28%, list=8%, signal=31% |
| 197 | REACTOME\_ION\_TRANSPORT\_BY\_P\_TYPE\_ATPASES |  | 33 | -0.48 | -1.48 | 0.081 | 0.071 | 0.990 | 958 | tags=21%, list=6%, signal=23% |
| 198 | MISHRA\_CARCINOMA\_ASSOCIATED\_FIBROBLAST\_UP |  | 20 | -0.52 | -1.47 | 0.069 | 0.073 | 0.992 | 2086 | tags=55%, list=13%, signal=63% |
| 199 | TURASHVILI\_BREAST\_LOBULAR\_CARCINOMA\_VS\_DUCTAL\_NORMAL\_DN |  | 79 | -0.39 | -1.47 | 0.000 | 0.073 | 0.992 | 819 | tags=16%, list=5%, signal=17% |
| 200 | BIOCARTA\_COMP\_PATHWAY |  | 17 | -0.56 | -1.47 | 0.046 | 0.075 | 0.995 | 688 | tags=29%, list=4%, signal=31% |
| 201 | KEGG\_GLYCEROLIPID\_METABOLISM |  | 44 | -0.43 | -1.46 | 0.059 | 0.075 | 0.995 | 1606 | tags=27%, list=10%, signal=30% |
| 202 | ENGELMANN\_CANCER\_PROGENITORS\_DN |  | 58 | -0.42 | -1.46 | 0.000 | 0.075 | 0.995 | 1756 | tags=34%, list=11%, signal=39% |
| 203 | REACTOME\_TRANSPORT\_OF\_GLUCOSE\_AND\_OTHER\_SUGARS\_BILE\_SALTS\_AND\_ORGANIC\_ACIDS\_METAL\_IONS\_AND\_AMINE\_COMPOUNDS |  | 86 | -0.36 | -1.46 | 0.000 | 0.075 | 0.995 | 1695 | tags=22%, list=11%, signal=25% |
| 204 | LEE\_LIVER\_CANCER\_MYC\_TGFA\_DN |  | 62 | -0.39 | -1.46 | 0.000 | 0.075 | 0.995 | 2127 | tags=31%, list=14%, signal=35% |
| 205 | TURASHVILI\_BREAST\_DUCTAL\_CARCINOMA\_VS\_LOBULAR\_NORMAL\_DN |  | 52 | -0.44 | -1.46 | 0.000 | 0.076 | 0.996 | 984 | tags=27%, list=6%, signal=29% |
| 206 | ZHENG\_IL22\_SIGNALING\_UP |  | 50 | -0.42 | -1.46 | 0.000 | 0.076 | 0.996 | 2437 | tags=38%, list=15%, signal=45% |
| 207 | WESTON\_VEGFA\_TARGETS |  | 103 | -0.32 | -1.46 | 0.000 | 0.076 | 0.997 | 1377 | tags=30%, list=9%, signal=33% |
| 208 | LEIN\_ASTROCYTE\_MARKERS |  | 38 | -0.44 | -1.46 | 0.000 | 0.076 | 0.997 | 1755 | tags=42%, list=11%, signal=47% |
| 209 | MUELLER\_METHYLATED\_IN\_GLIOBLASTOMA |  | 34 | -0.46 | -1.45 | 0.027 | 0.077 | 0.997 | 1527 | tags=29%, list=10%, signal=32% |
| 210 | MIKKELSEN\_MCV6\_ICP\_WITH\_H3K4ME3\_AND\_H3K27ME3 |  | 28 | -0.45 | -1.43 | 0.029 | 0.086 | 1.000 | 2486 | tags=21%, list=16%, signal=25% |
| 211 | WONG\_ENDMETRIUM\_CANCER\_DN |  | 76 | -0.37 | -1.43 | 0.000 | 0.086 | 1.000 | 2668 | tags=39%, list=17%, signal=47% |
| 212 | VILIMAS\_NOTCH1\_TARGETS\_UP |  | 50 | -0.42 | -1.43 | 0.000 | 0.086 | 1.000 | 3200 | tags=46%, list=20%, signal=58% |
| 213 | HAN\_JNK\_SINGALING\_DN |  | 37 | -0.42 | -1.43 | 0.034 | 0.086 | 1.000 | 979 | tags=24%, list=6%, signal=26% |
| 214 | ISSAEVA\_MLL2\_TARGETS |  | 58 | -0.42 | -1.43 | 0.000 | 0.087 | 1.000 | 1234 | tags=29%, list=8%, signal=32% |
| 215 | ZEMBUTSU\_SENSITIVITY\_TO\_VINCRISTINE |  | 18 | -0.53 | -1.43 | 0.070 | 0.087 | 1.000 | 1682 | tags=44%, list=11%, signal=50% |
| 216 | LIM\_MAMMARY\_LUMINAL\_PROGENITOR\_UP |  | 55 | -0.43 | -1.42 | 0.000 | 0.087 | 1.000 | 1377 | tags=22%, list=9%, signal=24% |
| 217 | KANNAN\_TP53\_TARGETS\_UP |  | 54 | -0.42 | -1.41 | 0.000 | 0.093 | 1.000 | 2217 | tags=46%, list=14%, signal=54% |
| 218 | YAO\_HOXA10\_TARGETS\_VIA\_PROGESTERONE\_UP |  | 74 | -0.38 | -1.40 | 0.000 | 0.097 | 1.000 | 1762 | tags=35%, list=11%, signal=39% |
| 219 | HATADA\_METHYLATED\_IN\_LUNG\_CANCER\_DN |  | 23 | -0.46 | -1.40 | 0.040 | 0.098 | 1.000 | 98 | tags=9%, list=1%, signal=9% |
| 220 | GOUYER\_TATI\_TARGETS\_DN |  | 15 | -0.56 | -1.40 | 0.109 | 0.098 | 1.000 | 938 | tags=53%, list=6%, signal=57% |
| 221 | HUMMERICH\_BENIGN\_SKIN\_TUMOR\_DN |  | 18 | -0.50 | -1.40 | 0.072 | 0.098 | 1.000 | 1762 | tags=22%, list=11%, signal=25% |
| 222 | VALK\_AML\_CLUSTER\_7 |  | 24 | -0.47 | -1.40 | 0.056 | 0.098 | 1.000 | 44 | tags=13%, list=0%, signal=13% |
| 223 | FERRANDO\_TAL1\_NEIGHBORS |  | 16 | -0.53 | -1.39 | 0.062 | 0.100 | 1.000 | 1527 | tags=25%, list=10%, signal=28% |
| 224 | OUELLET\_CULTURED\_OVARIAN\_CANCER\_INVASIVE\_VS\_LMP\_DN |  | 27 | -0.44 | -1.39 | 0.128 | 0.100 | 1.000 | 1462 | tags=22%, list=9%, signal=24% |
| 225 | REACTOME\_NCAM1\_INTERACTIONS |  | 39 | -0.44 | -1.39 | 0.000 | 0.100 | 1.000 | 2926 | tags=38%, list=19%, signal=47% |
| 226 | BAELDE\_DIABETIC\_NEPHROPATHY\_UP |  | 77 | -0.38 | -1.39 | 0.000 | 0.100 | 1.000 | 1665 | tags=38%, list=11%, signal=42% |
| 227 | LINDGREN\_BLADDER\_CANCER\_HIGH\_RECURRENCE |  | 44 | -0.40 | -1.38 | 0.000 | 0.108 | 1.000 | 1316 | tags=30%, list=8%, signal=32% |
| 228 | LIANG\_SILENCED\_BY\_METHYLATION\_UP |  | 30 | -0.45 | -1.37 | 0.054 | 0.109 | 1.000 | 1244 | tags=33%, list=8%, signal=36% |
| 229 | ZHENG\_GLIOBLASTOMA\_PLASTICITY\_DN |  | 55 | -0.39 | -1.37 | 0.000 | 0.109 | 1.000 | 595 | tags=22%, list=4%, signal=23% |
| 230 | SATO\_SILENCED\_BY\_DEACETYLATION\_IN\_PANCREATIC\_CANCER |  | 37 | -0.40 | -1.36 | 0.059 | 0.115 | 1.000 | 1922 | tags=41%, list=12%, signal=46% |
| 231 | REACTOME\_PLATELET\_CALCIUM\_HOMEOSTASIS |  | 15 | -0.54 | -1.36 | 0.109 | 0.114 | 1.000 | 1050 | tags=13%, list=7%, signal=14% |
| 232 | WANG\_THOC1\_TARGETS\_DN |  | 19 | -0.50 | -1.36 | 0.067 | 0.115 | 1.000 | 1283 | tags=21%, list=8%, signal=23% |
| 233 | STAMBOLSKY\_RESPONSE\_TO\_VITAMIN\_D3\_DN |  | 17 | -0.50 | -1.36 | 0.104 | 0.114 | 1.000 | 446 | tags=24%, list=3%, signal=24% |
| 234 | KEGG\_METABOLISM\_OF\_XENOBIOTICS\_BY\_CYTOCHROME\_P450 |  | 56 | -0.38 | -1.36 | 0.000 | 0.114 | 1.000 | 1745 | tags=36%, list=11%, signal=40% |
| 235 | RIGGINS\_TAMOXIFEN\_RESISTANCE\_UP |  | 66 | -0.38 | -1.35 | 0.000 | 0.119 | 1.000 | 2378 | tags=33%, list=15%, signal=39% |
| 236 | YU\_MYC\_TARGETS\_DN |  | 48 | -0.39 | -1.34 | 0.000 | 0.123 | 1.000 | 1804 | tags=40%, list=11%, signal=45% |
| 237 | REACTOME\_REGULATION\_OF\_GENE\_EXPRESSION\_IN\_BETA\_CELLS |  | 19 | -0.47 | -1.34 | 0.083 | 0.124 | 1.000 | 743 | tags=21%, list=5%, signal=22% |
| 238 | REACTOME\_GLYCOSPHINGOLIPID\_METABOLISM |  | 32 | -0.45 | -1.34 | 0.026 | 0.124 | 1.000 | 2040 | tags=38%, list=13%, signal=43% |
| 239 | NIELSEN\_SCHWANNOMA\_DN |  | 15 | -0.52 | -1.34 | 0.125 | 0.126 | 1.000 | 2058 | tags=40%, list=13%, signal=46% |
| 240 | KEGG\_DRUG\_METABOLISM\_CYTOCHROME\_P450 |  | 57 | -0.37 | -1.34 | 0.000 | 0.126 | 1.000 | 1745 | tags=32%, list=11%, signal=35% |
| 241 | LIU\_VAV3\_PROSTATE\_CARCINOGENESIS\_UP |  | 85 | -0.35 | -1.34 | 0.000 | 0.126 | 1.000 | 1821 | tags=32%, list=12%, signal=36% |
| 242 | LEE\_LIVER\_CANCER\_DENA\_DN |  | 71 | -0.39 | -1.34 | 0.000 | 0.125 | 1.000 | 1493 | tags=21%, list=9%, signal=23% |
| 243 | MIKKELSEN\_MCV6\_ICP\_WITH\_H3K27ME3 |  | 65 | -0.39 | -1.33 | 0.000 | 0.126 | 1.000 | 3914 | tags=31%, list=25%, signal=41% |
| 244 | REACTOME\_O\_LINKED\_GLYCOSYLATION\_OF\_MUCINS |  | 48 | -0.43 | -1.33 | 0.000 | 0.126 | 1.000 | 1323 | tags=29%, list=8%, signal=32% |
| 245 | WESTON\_VEGFA\_TARGETS\_6HR |  | 59 | -0.41 | -1.33 | 0.000 | 0.127 | 1.000 | 1497 | tags=37%, list=10%, signal=41% |
| 246 | POTTI\_ADRIAMYCIN\_SENSITIVITY |  | 67 | -0.38 | -1.33 | 0.000 | 0.127 | 1.000 | 1608 | tags=37%, list=10%, signal=41% |
| 247 | YAMASHITA\_LIVER\_CANCER\_STEM\_CELL\_UP |  | 46 | -0.41 | -1.33 | 0.105 | 0.127 | 1.000 | 973 | tags=28%, list=6%, signal=30% |
| 248 | CLASPER\_LYMPHATIC\_VESSELS\_DURING\_METASTASIS\_UP |  | 20 | -0.48 | -1.33 | 0.084 | 0.126 | 1.000 | 1589 | tags=40%, list=10%, signal=44% |
| 249 | NADLER\_OBESITY\_UP |  | 58 | -0.36 | -1.33 | 0.000 | 0.128 | 1.000 | 1099 | tags=29%, list=7%, signal=31% |
| 250 | BOYAULT\_LIVER\_CANCER\_SUBCLASS\_G6\_DN |  | 19 | -0.49 | -1.32 | 0.102 | 0.132 | 1.000 | 2297 | tags=53%, list=15%, signal=62% |
| 251 | REACTOME\_GENERATION\_OF\_SECOND\_MESSENGER\_MOLECULES |  | 22 | -0.49 | -1.32 | 0.125 | 0.132 | 1.000 | 470 | tags=14%, list=3%, signal=14% |
| 252 | REACTOME\_PHASE1\_FUNCTIONALIZATION\_OF\_COMPOUNDS |  | 64 | -0.35 | -1.31 | 0.000 | 0.136 | 1.000 | 2354 | tags=28%, list=15%, signal=33% |
| 253 | CHEBOTAEV\_GR\_TARGETS\_UP |  | 70 | -0.35 | -1.31 | 0.000 | 0.137 | 1.000 | 685 | tags=17%, list=4%, signal=18% |
| 254 | FINETTI\_BREAST\_CANCERS\_KINOME\_BLUE |  | 20 | -0.46 | -1.31 | 0.092 | 0.137 | 1.000 | 981 | tags=20%, list=6%, signal=21% |
| 255 | LIAN\_NEUTROPHIL\_GRANULE\_CONSTITUENTS |  | 22 | -0.47 | -1.31 | 0.077 | 0.138 | 1.000 | 3005 | tags=27%, list=19%, signal=34% |
| 256 | SMID\_BREAST\_CANCER\_RELAPSE\_IN\_BONE\_UP |  | 81 | -0.34 | -1.30 | 0.000 | 0.142 | 1.000 | 845 | tags=19%, list=5%, signal=19% |
| 257 | CHIARADONNA\_NEOPLASTIC\_TRANSFORMATION\_KRAS\_CDC25\_DN |  | 50 | -0.38 | -1.30 | 0.000 | 0.143 | 1.000 | 2312 | tags=42%, list=15%, signal=49% |
| 258 | PID\_CONE\_PATHWAY |  | 21 | -0.45 | -1.30 | 0.153 | 0.144 | 1.000 | 4452 | tags=29%, list=28%, signal=40% |
| 259 | SENGUPTA\_NASOPHARYNGEAL\_CARCINOMA\_WITH\_LMP1\_DN |  | 128 | -0.35 | -1.29 | 0.000 | 0.146 | 1.000 | 2733 | tags=24%, list=17%, signal=29% |
| 260 | KEGG\_COMPLEMENT\_AND\_COAGULATION\_CASCADES |  | 64 | -0.41 | -1.29 | 0.000 | 0.147 | 1.000 | 688 | tags=11%, list=4%, signal=11% |
| 261 | CHEN\_ETV5\_TARGETS\_SERTOLI |  | 20 | -0.46 | -1.29 | 0.059 | 0.149 | 1.000 | 400 | tags=15%, list=3%, signal=15% |
| 262 | PLASARI\_NFIC\_TARGETS\_BASAL\_UP |  | 26 | -0.45 | -1.29 | 0.130 | 0.150 | 1.000 | 1701 | tags=27%, list=11%, signal=30% |
| 263 | REACTOME\_LIPOPROTEIN\_METABOLISM |  | 26 | -0.42 | -1.28 | 0.093 | 0.156 | 1.000 | 455 | tags=15%, list=3%, signal=16% |
| 264 | REACTOME\_BILE\_ACID\_AND\_BILE\_SALT\_METABOLISM |  | 26 | -0.41 | -1.28 | 0.042 | 0.158 | 1.000 | 2928 | tags=31%, list=19%, signal=38% |
| 265 | KOHOUTEK\_CCNT1\_TARGETS |  | 44 | -0.39 | -1.28 | 0.100 | 0.157 | 1.000 | 2305 | tags=43%, list=15%, signal=50% |
| 266 | NIELSEN\_LIPOSARCOMA\_UP |  | 17 | -0.47 | -1.27 | 0.108 | 0.162 | 1.000 | 968 | tags=29%, list=6%, signal=31% |
| 267 | REACTOME\_NITRIC\_OXIDE\_STIMULATES\_GUANYLATE\_CYCLASE |  | 24 | -0.43 | -1.27 | 0.070 | 0.164 | 1.000 | 3162 | tags=46%, list=20%, signal=57% |
| 268 | HECKER\_IFNB1\_TARGETS |  | 72 | -0.32 | -1.26 | 0.000 | 0.166 | 1.000 | 1583 | tags=22%, list=10%, signal=25% |
| 269 | CORRE\_MULTIPLE\_MYELOMA\_DN |  | 55 | -0.34 | -1.26 | 0.111 | 0.166 | 1.000 | 1840 | tags=33%, list=12%, signal=37% |
| 270 | REN\_ALVEOLAR\_RHABDOMYOSARCOMA\_UP |  | 94 | -0.33 | -1.26 | 0.000 | 0.168 | 1.000 | 2084 | tags=21%, list=13%, signal=24% |
| 271 | REACTOME\_FORMATION\_OF\_FIBRIN\_CLOT\_CLOTTING\_CASCADE |  | 31 | -0.42 | -1.25 | 0.093 | 0.172 | 1.000 | 2869 | tags=19%, list=18%, signal=24% |
| 272 | BEGUM\_TARGETS\_OF\_PAX3\_FOXO1\_FUSION\_DN |  | 44 | -0.37 | -1.25 | 0.000 | 0.172 | 1.000 | 1983 | tags=45%, list=13%, signal=52% |
| 273 | WEBER\_METHYLATED\_ICP\_IN\_FIBROBLAST |  | 19 | -0.45 | -1.25 | 0.173 | 0.172 | 1.000 | 8733 | tags=100%, list=55%, signal=224% |
| 274 | ICHIBA\_GRAFT\_VERSUS\_HOST\_DISEASE\_D7\_DN |  | 38 | -0.38 | -1.25 | 0.091 | 0.171 | 1.000 | 822 | tags=24%, list=5%, signal=25% |
| 275 | HASLINGER\_B\_CLL\_WITH\_MUTATED\_VH\_GENES |  | 16 | -0.46 | -1.25 | 0.110 | 0.171 | 1.000 | 2914 | tags=44%, list=19%, signal=54% |
| 276 | REACTOME\_CELL\_CELL\_JUNCTION\_ORGANIZATION |  | 56 | -0.38 | -1.25 | 0.000 | 0.172 | 1.000 | 1010 | tags=23%, list=6%, signal=25% |
| 277 | BAUS\_TFF2\_TARGETS\_UP |  | 29 | -0.41 | -1.25 | 0.118 | 0.173 | 1.000 | 1485 | tags=34%, list=9%, signal=38% |
| 278 | CHIBA\_RESPONSE\_TO\_TSA\_UP |  | 51 | -0.38 | -1.24 | 0.000 | 0.178 | 1.000 | 1774 | tags=39%, list=11%, signal=44% |
| 279 | WOO\_LIVER\_CANCER\_RECURRENCE\_DN |  | 73 | -0.34 | -1.24 | 0.000 | 0.178 | 1.000 | 1028 | tags=23%, list=7%, signal=25% |
| 280 | REACTOME\_CGMP\_EFFECTS |  | 19 | -0.45 | -1.24 | 0.141 | 0.179 | 1.000 | 1210 | tags=26%, list=8%, signal=28% |
| 281 | HUANG\_FOXA2\_TARGETS\_DN |  | 35 | -0.38 | -1.24 | 0.057 | 0.179 | 1.000 | 1305 | tags=40%, list=8%, signal=44% |
| 282 | GARCIA\_TARGETS\_OF\_FLI1\_AND\_DAX1\_UP |  | 45 | -0.35 | -1.24 | 0.063 | 0.179 | 1.000 | 2568 | tags=47%, list=16%, signal=56% |
| 283 | KEGG\_ARACHIDONIC\_ACID\_METABOLISM |  | 53 | -0.35 | -1.23 | 0.050 | 0.182 | 1.000 | 1235 | tags=23%, list=8%, signal=24% |
| 284 | COWLING\_MYCN\_TARGETS |  | 37 | -0.38 | -1.23 | 0.071 | 0.184 | 1.000 | 1595 | tags=27%, list=10%, signal=30% |
| 285 | DELPUECH\_FOXO3\_TARGETS\_UP |  | 63 | -0.33 | -1.23 | 0.000 | 0.184 | 1.000 | 664 | tags=22%, list=4%, signal=23% |
| 286 | TSENG\_ADIPOGENIC\_POTENTIAL\_UP |  | 29 | -0.38 | -1.23 | 0.115 | 0.184 | 1.000 | 730 | tags=28%, list=5%, signal=29% |
| 287 | ZHAN\_V1\_LATE\_DIFFERENTIATION\_GENES\_UP |  | 31 | -0.40 | -1.23 | 0.114 | 0.185 | 1.000 | 1870 | tags=48%, list=12%, signal=55% |
| 288 | MCDOWELL\_ACUTE\_LUNG\_INJURY\_DN |  | 46 | -0.37 | -1.23 | 0.071 | 0.186 | 1.000 | 1108 | tags=26%, list=7%, signal=28% |
| 289 | REACTOME\_A\_TETRASACCHARIDE\_LINKER\_SEQUENCE\_IS\_REQUIRED\_FOR\_GAG\_SYNTHESIS |  | 23 | -0.42 | -1.23 | 0.114 | 0.186 | 1.000 | 769 | tags=26%, list=5%, signal=27% |
| 290 | BERNARD\_PPAPDC1B\_TARGETS\_DN |  | 47 | -0.37 | -1.22 | 0.091 | 0.187 | 1.000 | 1368 | tags=26%, list=9%, signal=28% |
| 291 | LUI\_THYROID\_CANCER\_CLUSTER\_2 |  | 36 | -0.38 | -1.22 | 0.061 | 0.188 | 1.000 | 944 | tags=28%, list=6%, signal=29% |
| 292 | VALK\_AML\_CLUSTER\_8 |  | 22 | -0.40 | -1.22 | 0.098 | 0.187 | 1.000 | 245 | tags=14%, list=2%, signal=14% |
| 293 | ZHAN\_MULTIPLE\_MYELOMA\_CD1\_DN |  | 36 | -0.38 | -1.22 | 0.188 | 0.191 | 1.000 | 1928 | tags=39%, list=12%, signal=44% |
| 294 | KAAB\_FAILED\_HEART\_ATRIUM\_UP |  | 32 | -0.38 | -1.22 | 0.088 | 0.191 | 1.000 | 2544 | tags=31%, list=16%, signal=37% |
| 295 | KIM\_BIPOLAR\_DISORDER\_OLIGODENDROCYTE\_DENSITY\_CORR\_DN |  | 78 | -0.31 | -1.22 | 0.250 | 0.190 | 1.000 | 1674 | tags=22%, list=11%, signal=24% |
| 296 | VART\_KSHV\_INFECTION\_ANGIOGENIC\_MARKERS\_DN |  | 129 | -0.30 | -1.22 | 0.000 | 0.191 | 1.000 | 2334 | tags=32%, list=15%, signal=37% |
| 297 | HUMMERICH\_MALIGNANT\_SKIN\_TUMOR\_DN |  | 17 | -0.46 | -1.22 | 0.136 | 0.191 | 1.000 | 2584 | tags=35%, list=16%, signal=42% |
| 298 | CUI\_TCF21\_TARGETS\_UP |  | 35 | -0.36 | -1.21 | 0.067 | 0.197 | 1.000 | 1137 | tags=29%, list=7%, signal=31% |
| 299 | REACTOME\_POTASSIUM\_CHANNELS |  | 97 | -0.30 | -1.21 | 0.000 | 0.200 | 1.000 | 4452 | tags=41%, list=28%, signal=57% |
| 300 | HUMMERICH\_BENIGN\_SKIN\_TUMOR\_UP |  | 15 | -0.47 | -1.21 | 0.211 | 0.200 | 1.000 | 6 | tags=7%, list=0%, signal=7% |
| 301 | LIU\_IL13\_MEMORY\_MODEL\_UP |  | 17 | -0.45 | -1.20 | 0.121 | 0.201 | 1.000 | 2404 | tags=47%, list=15%, signal=55% |
| 302 | YAO\_TEMPORAL\_RESPONSE\_TO\_PROGESTERONE\_CLUSTER\_1 |  | 65 | -0.36 | -1.20 | 0.167 | 0.205 | 1.000 | 1233 | tags=29%, list=8%, signal=32% |
| 303 | RICKMAN\_HEAD\_AND\_NECK\_CANCER\_F |  | 52 | -0.36 | -1.20 | 0.111 | 0.204 | 1.000 | 1195 | tags=17%, list=8%, signal=19% |
| 304 | PEDERSEN\_METASTASIS\_BY\_ERBB2\_ISOFORM\_6 |  | 27 | -0.41 | -1.20 | 0.148 | 0.206 | 1.000 | 1602 | tags=22%, list=10%, signal=25% |
| 305 | TOMLINS\_PROSTATE\_CANCER\_DN |  | 37 | -0.38 | -1.19 | 0.097 | 0.209 | 1.000 | 944 | tags=30%, list=6%, signal=32% |
| 306 | RUAN\_RESPONSE\_TO\_TNF\_TROGLITAZONE\_UP |  | 15 | -0.44 | -1.18 | 0.204 | 0.219 | 1.000 | 1797 | tags=60%, list=11%, signal=68% |
| 307 | BARRIER\_CANCER\_RELAPSE\_NORMAL\_SAMPLE\_DN |  | 27 | -0.42 | -1.18 | 0.188 | 0.218 | 1.000 | 1890 | tags=44%, list=12%, signal=50% |
| 308 | LEI\_HOXC8\_TARGETS\_DN |  | 17 | -0.44 | -1.18 | 0.206 | 0.218 | 1.000 | 613 | tags=29%, list=4%, signal=31% |
| 309 | TSENG\_ADIPOGENIC\_POTENTIAL\_DN |  | 44 | -0.37 | -1.18 | 0.115 | 0.220 | 1.000 | 889 | tags=23%, list=6%, signal=24% |
| 310 | GHANDHI\_DIRECT\_IRRADIATION\_DN |  | 24 | -0.40 | -1.18 | 0.190 | 0.222 | 1.000 | 3200 | tags=46%, list=20%, signal=57% |
| 311 | MIKKELSEN\_MCV6\_LCP\_WITH\_H3K27ME3 |  | 20 | -0.44 | -1.18 | 0.212 | 0.222 | 1.000 | 3913 | tags=30%, list=25%, signal=40% |
| 312 | EBAUER\_MYOGENIC\_TARGETS\_OF\_PAX3\_FOXO1\_FUSION |  | 49 | -0.33 | -1.18 | 0.143 | 0.222 | 1.000 | 889 | tags=14%, list=6%, signal=15% |
| 313 | URS\_ADIPOCYTE\_DIFFERENTIATION\_DN |  | 27 | -0.36 | -1.18 | 0.173 | 0.223 | 1.000 | 1991 | tags=52%, list=13%, signal=59% |
| 314 | LEE\_INTRATHYMIC\_T\_PROGENITOR |  | 18 | -0.45 | -1.17 | 0.191 | 0.223 | 1.000 | 643 | tags=22%, list=4%, signal=23% |
| 315 | MASRI\_RESISTANCE\_TO\_TAMOXIFEN\_AND\_AROMATASE\_INHIBITORS\_UP |  | 19 | -0.42 | -1.17 | 0.228 | 0.227 | 1.000 | 845 | tags=16%, list=5%, signal=17% |
| 316 | GAUSSMANN\_MLL\_AF4\_FUSION\_TARGETS\_E\_UP |  | 81 | -0.34 | -1.17 | 0.000 | 0.227 | 1.000 | 2959 | tags=42%, list=19%, signal=51% |
| 317 | AMBROSINI\_FLAVOPIRIDOL\_TREATMENT\_TP53 |  | 94 | -0.30 | -1.17 | 0.000 | 0.228 | 1.000 | 1286 | tags=22%, list=8%, signal=24% |
| 318 | FERREIRA\_EWINGS\_SARCOMA\_UNSTABLE\_VS\_STABLE\_DN |  | 85 | -0.32 | -1.16 | 0.000 | 0.240 | 1.000 | 1753 | tags=29%, list=11%, signal=33% |
| 319 | IZADPANAH\_STEM\_CELL\_ADIPOSE\_VS\_BONE\_DN |  | 95 | -0.32 | -1.16 | 0.000 | 0.241 | 1.000 | 2382 | tags=34%, list=15%, signal=39% |
| 320 | SHEDDEN\_LUNG\_CANCER\_GOOD\_SURVIVAL\_A5 |  | 55 | -0.34 | -1.15 | 0.143 | 0.243 | 1.000 | 1076 | tags=22%, list=7%, signal=23% |
| 321 | SCHRAETS\_MLL\_TARGETS\_DN |  | 31 | -0.38 | -1.15 | 0.186 | 0.243 | 1.000 | 1255 | tags=39%, list=8%, signal=42% |
| 322 | KEGG\_RETINOL\_METABOLISM |  | 48 | -0.32 | -1.15 | 0.200 | 0.243 | 1.000 | 2336 | tags=31%, list=15%, signal=37% |
| 323 | LIN\_SILENCED\_BY\_TUMOR\_MICROENVIRONMENT |  | 87 | -0.33 | -1.15 | 0.000 | 0.245 | 1.000 | 1602 | tags=28%, list=10%, signal=31% |
| 324 | KEGG\_NITROGEN\_METABOLISM |  | 22 | -0.40 | -1.15 | 0.192 | 0.248 | 1.000 | 768 | tags=27%, list=5%, signal=29% |
| 325 | NIKOLSKY\_BREAST\_CANCER\_16P13\_AMPLICON |  | 93 | -0.30 | -1.15 | 0.000 | 0.247 | 1.000 | 2087 | tags=28%, list=13%, signal=32% |
| 326 | MARKEY\_RB1\_CHRONIC\_LOF\_DN |  | 105 | -0.34 | -1.15 | 0.000 | 0.248 | 1.000 | 1407 | tags=27%, list=9%, signal=29% |
| 327 | MEISSNER\_NPC\_HCP\_WITH\_H3K4ME3\_AND\_H3K27ME3 |  | 124 | -0.28 | -1.14 | 0.000 | 0.249 | 1.000 | 1596 | tags=15%, list=10%, signal=17% |
| 328 | LANDIS\_BREAST\_CANCER\_PROGRESSION\_UP |  | 43 | -0.34 | -1.14 | 0.190 | 0.249 | 1.000 | 1187 | tags=35%, list=8%, signal=38% |
| 329 | NAKAMURA\_CANCER\_MICROENVIRONMENT\_UP |  | 18 | -0.41 | -1.14 | 0.246 | 0.255 | 1.000 | 3436 | tags=61%, list=22%, signal=78% |
| 330 | CHIANG\_LIVER\_CANCER\_SUBCLASS\_POLYSOMY7\_UP |  | 66 | -0.34 | -1.14 | 0.222 | 0.256 | 1.000 | 674 | tags=15%, list=4%, signal=16% |
| 331 | CHIARETTI\_ACUTE\_LYMPHOBLASTIC\_LEUKEMIA\_ZAP70 |  | 61 | -0.30 | -1.14 | 0.111 | 0.255 | 1.000 | 1299 | tags=23%, list=8%, signal=25% |
| 332 | HELLEBREKERS\_SILENCED\_DURING\_TUMOR\_ANGIOGENESIS |  | 72 | -0.32 | -1.14 | 0.111 | 0.255 | 1.000 | 1794 | tags=32%, list=11%, signal=36% |
| 333 | NIKOLSKY\_BREAST\_CANCER\_1Q21\_AMPLICON |  | 33 | -0.36 | -1.13 | 0.143 | 0.258 | 1.000 | 1888 | tags=39%, list=12%, signal=45% |
| 334 | APPEL\_IMATINIB\_RESPONSE |  | 32 | -0.36 | -1.13 | 0.225 | 0.260 | 1.000 | 1561 | tags=34%, list=10%, signal=38% |
| 335 | SU\_LIVER |  | 49 | -0.32 | -1.13 | 0.167 | 0.260 | 1.000 | 2319 | tags=22%, list=15%, signal=26% |
| 336 | THUM\_MIR21\_TARGETS\_HEART\_DISEASE\_UP |  | 16 | -0.44 | -1.13 | 0.220 | 0.262 | 1.000 | 2655 | tags=44%, list=17%, signal=53% |
| 337 | LEIN\_OLIGODENDROCYTE\_MARKERS |  | 62 | -0.31 | -1.13 | 0.000 | 0.263 | 1.000 | 1559 | tags=31%, list=10%, signal=34% |
| 338 | SUMI\_HNF4A\_TARGETS |  | 29 | -0.37 | -1.13 | 0.208 | 0.264 | 1.000 | 2255 | tags=31%, list=14%, signal=36% |
| 339 | CADWELL\_ATG16L1\_TARGETS\_UP |  | 84 | -0.28 | -1.13 | 0.000 | 0.264 | 1.000 | 2584 | tags=31%, list=16%, signal=37% |
| 340 | KEGG\_PROXIMAL\_TUBULE\_BICARBONATE\_RECLAMATION |  | 22 | -0.38 | -1.12 | 0.194 | 0.270 | 1.000 | 2159 | tags=50%, list=14%, signal=58% |
| 341 | BROWNE\_HCMV\_INFECTION\_30MIN\_UP |  | 47 | -0.32 | -1.12 | 0.174 | 0.270 | 1.000 | 1758 | tags=26%, list=11%, signal=29% |
| 342 | BIOCARTA\_TID\_PATHWAY |  | 19 | -0.40 | -1.12 | 0.222 | 0.273 | 1.000 | 1111 | tags=26%, list=7%, signal=28% |
| 343 | KEGG\_LEISHMANIA\_INFECTION |  | 57 | -0.31 | -1.12 | 0.188 | 0.275 | 1.000 | 1443 | tags=23%, list=9%, signal=25% |
| 344 | CROONQUIST\_STROMAL\_STIMULATION\_UP |  | 53 | -0.32 | -1.12 | 0.059 | 0.275 | 1.000 | 1460 | tags=30%, list=9%, signal=33% |
| 345 | DACOSTA\_ERCC3\_ALLELE\_XPCS\_VS\_TTD\_UP |  | 27 | -0.38 | -1.11 | 0.220 | 0.277 | 1.000 | 1788 | tags=30%, list=11%, signal=33% |
| 346 | REACTOME\_NUCLEAR\_RECEPTOR\_TRANSCRIPTION\_PATHWAY |  | 45 | -0.34 | -1.11 | 0.136 | 0.278 | 1.000 | 1662 | tags=24%, list=11%, signal=27% |
| 347 | PID\_IL23PATHWAY |  | 35 | -0.33 | -1.11 | 0.229 | 0.281 | 1.000 | 514 | tags=11%, list=3%, signal=12% |
| 348 | BANDRES\_RESPONSE\_TO\_CARMUSTIN\_WITHOUT\_MGMT\_48HR\_DN |  | 29 | -0.35 | -1.11 | 0.250 | 0.284 | 1.000 | 601 | tags=17%, list=4%, signal=18% |
| 349 | DAZARD\_UV\_RESPONSE\_CLUSTER\_G4 |  | 18 | -0.41 | -1.11 | 0.326 | 0.283 | 1.000 | 2066 | tags=44%, list=13%, signal=51% |
| 350 | PID\_SYNDECAN\_3\_PATHWAY |  | 16 | -0.42 | -1.11 | 0.300 | 0.284 | 1.000 | 3397 | tags=38%, list=22%, signal=48% |
| 351 | BRIDEAU\_IMPRINTED\_GENES |  | 61 | -0.31 | -1.10 | 0.333 | 0.284 | 1.000 | 1571 | tags=23%, list=10%, signal=25% |
| 352 | REACTOME\_CHONDROITIN\_SULFATE\_DERMATAN\_SULFATE\_METABOLISM |  | 40 | -0.33 | -1.10 | 0.200 | 0.287 | 1.000 | 2225 | tags=33%, list=14%, signal=38% |
| 353 | BORCZUK\_MALIGNANT\_MESOTHELIOMA\_DN |  | 82 | -0.33 | -1.10 | 0.500 | 0.287 | 1.000 | 2528 | tags=35%, list=16%, signal=42% |
| 354 | KEGG\_GLYCOSPHINGOLIPID\_BIOSYNTHESIS\_GANGLIO\_SERIES |  | 15 | -0.43 | -1.10 | 0.289 | 0.293 | 1.000 | 1781 | tags=47%, list=11%, signal=53% |
| 355 | CHEMELLO\_SOLEUS\_VS\_EDL\_MYOFIBERS\_UP |  | 32 | -0.33 | -1.09 | 0.222 | 0.302 | 1.000 | 944 | tags=13%, list=6%, signal=13% |
| 356 | NADERI\_BREAST\_CANCER\_PROGNOSIS\_DN |  | 18 | -0.40 | -1.09 | 0.284 | 0.303 | 1.000 | 157 | tags=22%, list=1%, signal=22% |
| 357 | YAO\_TEMPORAL\_RESPONSE\_TO\_PROGESTERONE\_CLUSTER\_5 |  | 28 | -0.34 | -1.09 | 0.263 | 0.304 | 1.000 | 2382 | tags=46%, list=15%, signal=55% |
| 358 | TONKS\_TARGETS\_OF\_RUNX1\_RUNX1T1\_FUSION\_SUSTAINED\_IN\_GRANULOCYTE\_UP |  | 15 | -0.43 | -1.09 | 0.315 | 0.305 | 1.000 | 813 | tags=33%, list=5%, signal=35% |
| 359 | REACTOME\_PACKAGING\_OF\_TELOMERE\_ENDS |  | 31 | -0.33 | -1.09 | 0.277 | 0.305 | 1.000 | 2976 | tags=65%, list=19%, signal=79% |
| 360 | JECHLINGER\_EPITHELIAL\_TO\_MESENCHYMAL\_TRANSITION\_DN |  | 64 | -0.31 | -1.08 | 0.444 | 0.309 | 1.000 | 1715 | tags=38%, list=11%, signal=42% |
| 361 | REACTOME\_SPHINGOLIPID\_METABOLISM |  | 53 | -0.32 | -1.08 | 0.375 | 0.310 | 1.000 | 1304 | tags=26%, list=8%, signal=29% |
| 362 | VANDESLUIS\_COMMD1\_TARGETS\_GROUP\_3\_DN |  | 34 | -0.34 | -1.08 | 0.270 | 0.316 | 1.000 | 927 | tags=21%, list=6%, signal=22% |
| 363 | REACTOME\_DEGRADATION\_OF\_THE\_EXTRACELLULAR\_MATRIX |  | 27 | -0.35 | -1.07 | 0.281 | 0.319 | 1.000 | 1367 | tags=19%, list=9%, signal=20% |
| 364 | OLSSON\_E2F3\_TARGETS\_UP |  | 26 | -0.35 | -1.07 | 0.241 | 0.321 | 1.000 | 2142 | tags=42%, list=14%, signal=49% |
| 365 | TSUNODA\_CISPLATIN\_RESISTANCE\_DN |  | 41 | -0.35 | -1.07 | 0.273 | 0.325 | 1.000 | 1313 | tags=29%, list=8%, signal=32% |
| 366 | BROWNE\_HCMV\_INFECTION\_2HR\_UP |  | 30 | -0.35 | -1.07 | 0.288 | 0.324 | 1.000 | 1922 | tags=40%, list=12%, signal=45% |
| 367 | ONDER\_CDH1\_TARGETS\_3\_DN |  | 46 | -0.32 | -1.07 | 0.222 | 0.326 | 1.000 | 1410 | tags=17%, list=9%, signal=19% |
| 368 | KEGG\_TYPE\_I\_DIABETES\_MELLITUS |  | 33 | -0.33 | -1.06 | 0.333 | 0.331 | 1.000 | 2307 | tags=30%, list=15%, signal=35% |
| 369 | LI\_INDUCED\_T\_TO\_NATURAL\_KILLER\_DN |  | 109 | -0.31 | -1.06 | 0.000 | 0.330 | 1.000 | 1820 | tags=33%, list=12%, signal=37% |
| 370 | YAO\_HOXA10\_TARGETS\_VIA\_PROGESTERONE\_DN |  | 18 | -0.39 | -1.06 | 0.291 | 0.331 | 1.000 | 1164 | tags=39%, list=7%, signal=42% |
| 371 | TERAMOTO\_OPN\_TARGETS\_CLUSTER\_6 |  | 26 | -0.34 | -1.06 | 0.286 | 0.331 | 1.000 | 866 | tags=15%, list=5%, signal=16% |
| 372 | CREIGHTON\_AKT1\_SIGNALING\_VIA\_MTOR\_UP |  | 32 | -0.34 | -1.06 | 0.400 | 0.337 | 1.000 | 1177 | tags=38%, list=7%, signal=40% |
| 373 | ROSS\_AML\_WITH\_AML1\_ETO\_FUSION |  | 65 | -0.29 | -1.06 | 0.250 | 0.339 | 1.000 | 1113 | tags=23%, list=7%, signal=25% |
| 374 | HOFFMANN\_LARGE\_TO\_SMALL\_PRE\_BII\_LYMPHOCYTE\_DN |  | 69 | -0.30 | -1.06 | 0.400 | 0.338 | 1.000 | 1949 | tags=38%, list=12%, signal=43% |
| 375 | REACTOME\_KERATAN\_SULFATE\_KERATIN\_METABOLISM |  | 29 | -0.33 | -1.05 | 0.353 | 0.342 | 1.000 | 1304 | tags=28%, list=8%, signal=30% |
| 376 | SCHOEN\_NFKB\_SIGNALING |  | 32 | -0.33 | -1.05 | 0.348 | 0.344 | 1.000 | 1466 | tags=31%, list=9%, signal=34% |
| 377 | REACTOME\_KERATAN\_SULFATE\_BIOSYNTHESIS |  | 25 | -0.35 | -1.05 | 0.286 | 0.343 | 1.000 | 1137 | tags=24%, list=7%, signal=26% |
| 378 | KORKOLA\_CORRELATED\_WITH\_POU5F1 |  | 25 | -0.36 | -1.05 | 0.365 | 0.347 | 1.000 | 1912 | tags=32%, list=12%, signal=36% |
| 379 | BOYAULT\_LIVER\_CANCER\_SUBCLASS\_G56\_DN |  | 15 | -0.41 | -1.05 | 0.386 | 0.347 | 1.000 | 1761 | tags=47%, list=11%, signal=52% |
| 380 | RUAN\_RESPONSE\_TO\_TNF\_DN |  | 80 | -0.31 | -1.04 | 0.000 | 0.351 | 1.000 | 1504 | tags=35%, list=10%, signal=38% |
| 381 | LEE\_LIVER\_CANCER\_ACOX1\_DN |  | 61 | -0.28 | -1.04 | 0.273 | 0.351 | 1.000 | 1015 | tags=13%, list=6%, signal=14% |
| 382 | CREIGHTON\_AKT1\_SIGNALING\_VIA\_MTOR\_DN |  | 22 | -0.37 | -1.04 | 0.370 | 0.358 | 1.000 | 471 | tags=27%, list=3%, signal=28% |
| 383 | KEGG\_FRUCTOSE\_AND\_MANNOSE\_METABOLISM |  | 34 | -0.33 | -1.04 | 0.375 | 0.357 | 1.000 | 1168 | tags=32%, list=7%, signal=35% |
| 384 | SMIRNOV\_CIRCULATING\_ENDOTHELIOCYTES\_IN\_CANCER\_UP |  | 144 | -0.27 | -1.04 | 0.000 | 0.359 | 1.000 | 1664 | tags=24%, list=11%, signal=27% |
| 385 | LANDIS\_ERBB2\_BREAST\_TUMORS\_65\_DN |  | 36 | -0.34 | -1.04 | 0.444 | 0.363 | 1.000 | 2622 | tags=56%, list=17%, signal=66% |
| 386 | CHANDRAN\_METASTASIS\_TOP50\_DN |  | 44 | -0.31 | -1.03 | 0.393 | 0.366 | 1.000 | 1277 | tags=30%, list=8%, signal=32% |
| 387 | GAUSSMANN\_MLL\_AF4\_FUSION\_TARGETS\_A\_DN |  | 76 | -0.30 | -1.03 | 0.600 | 0.368 | 1.000 | 1922 | tags=29%, list=12%, signal=33% |
| 388 | BIOCARTA\_DC\_PATHWAY |  | 21 | -0.36 | -1.03 | 0.317 | 0.376 | 1.000 | 4365 | tags=43%, list=28%, signal=59% |
| 389 | REACTOME\_CHYLOMICRON\_MEDIATED\_LIPID\_TRANSPORT |  | 16 | -0.38 | -1.02 | 0.424 | 0.390 | 1.000 | 455 | tags=19%, list=3%, signal=19% |
| 390 | COLIN\_PILOCYTIC\_ASTROCYTOMA\_VS\_GLIOBLASTOMA\_UP |  | 35 | -0.32 | -1.01 | 0.357 | 0.398 | 1.000 | 1755 | tags=31%, list=11%, signal=35% |
| 391 | BIOCARTA\_CTLA4\_PATHWAY |  | 18 | -0.38 | -1.01 | 0.404 | 0.403 | 1.000 | 13 | tags=6%, list=0%, signal=6% |
| 392 | REACTOME\_RNA\_POL\_I\_PROMOTER\_OPENING |  | 34 | -0.33 | -1.01 | 0.452 | 0.402 | 1.000 | 3021 | tags=74%, list=19%, signal=91% |
| 393 | HAHTOLA\_SEZARY\_SYNDROM\_DN |  | 34 | -0.31 | -1.01 | 0.515 | 0.408 | 1.000 | 487 | tags=9%, list=3%, signal=9% |
| 394 | DAUER\_STAT3\_TARGETS\_UP |  | 46 | -0.31 | -1.01 | 0.412 | 0.407 | 1.000 | 1922 | tags=30%, list=12%, signal=35% |
| 395 | HUANG\_DASATINIB\_RESISTANCE\_DN |  | 59 | -0.28 | -1.01 | 0.385 | 0.409 | 1.000 | 1285 | tags=22%, list=8%, signal=24% |
| 396 | REACTOME\_GLYCEROPHOSPHOLIPID\_BIOSYNTHESIS |  | 68 | -0.30 | -1.00 | 0.600 | 0.417 | 1.000 | 1728 | tags=31%, list=11%, signal=35% |
| 397 | PID\_HNF3APATHWAY |  | 41 | -0.29 | -1.00 | 0.368 | 0.420 | 1.000 | 2066 | tags=27%, list=13%, signal=31% |
| 398 | NOUSHMEHR\_GBM\_SILENCED\_BY\_METHYLATION |  | 43 | -0.29 | -1.00 | 0.393 | 0.422 | 1.000 | 1347 | tags=16%, list=9%, signal=18% |
| 399 | DUTERTRE\_ESTRADIOL\_RESPONSE\_6HR\_DN |  | 81 | -0.26 | -1.00 | 0.500 | 0.422 | 1.000 | 1337 | tags=20%, list=8%, signal=21% |
| 400 | YANG\_MUC2\_TARGETS\_DUODENUM\_3MO\_DN |  | 22 | -0.35 | -1.00 | 0.449 | 0.424 | 1.000 | 1730 | tags=32%, list=11%, signal=36% |
| 401 | REACTOME\_PRE\_NOTCH\_PROCESSING\_IN\_GOLGI |  | 16 | -0.38 | -0.99 | 0.417 | 0.428 | 1.000 | 483 | tags=25%, list=3%, signal=26% |
| 402 | KEGG\_GLYCEROPHOSPHOLIPID\_METABOLISM |  | 65 | -0.28 | -0.99 | 0.556 | 0.428 | 1.000 | 2067 | tags=31%, list=13%, signal=35% |
| 403 | HERNANDEZ\_ABERRANT\_MITOSIS\_BY\_DOCETACEL\_4NM\_UP |  | 19 | -0.35 | -0.99 | 0.418 | 0.429 | 1.000 | 943 | tags=21%, list=6%, signal=22% |
| 404 | PARK\_TRETINOIN\_RESPONSE\_AND\_RARA\_PLZF\_FUSION |  | 22 | -0.35 | -0.99 | 0.453 | 0.431 | 1.000 | 2698 | tags=45%, list=17%, signal=55% |
| 405 | VANTVEER\_BREAST\_CANCER\_METASTASIS\_UP |  | 44 | -0.29 | -0.98 | 0.538 | 0.445 | 1.000 | 1080 | tags=20%, list=7%, signal=22% |
| 406 | SCHWAB\_TARGETS\_OF\_BMYB\_POLYMORPHIC\_VARIANTS\_DN |  | 15 | -0.37 | -0.98 | 0.441 | 0.445 | 1.000 | 698 | tags=20%, list=4%, signal=21% |
| 407 | LIU\_LIVER\_CANCER |  | 27 | -0.33 | -0.98 | 0.500 | 0.450 | 1.000 | 917 | tags=19%, list=6%, signal=20% |
| 408 | REACTOME\_REGULATION\_OF\_BETA\_CELL\_DEVELOPMENT |  | 28 | -0.33 | -0.98 | 0.447 | 0.451 | 1.000 | 108 | tags=11%, list=1%, signal=11% |
| 409 | LEE\_LIVER\_CANCER\_CIPROFIBRATE\_DN |  | 61 | -0.28 | -0.97 | 0.444 | 0.456 | 1.000 | 2900 | tags=28%, list=18%, signal=34% |
| 410 | LIU\_SMARCA4\_TARGETS |  | 54 | -0.28 | -0.97 | 0.538 | 0.467 | 1.000 | 1677 | tags=26%, list=11%, signal=29% |
| 411 | YANG\_BREAST\_CANCER\_ESR1\_UP |  | 34 | -0.30 | -0.97 | 0.485 | 0.469 | 1.000 | 2066 | tags=29%, list=13%, signal=34% |
| 412 | WUNDER\_INFLAMMATORY\_RESPONSE\_AND\_CHOLESTEROL\_UP |  | 48 | -0.30 | -0.95 | 0.588 | 0.494 | 1.000 | 2045 | tags=35%, list=13%, signal=41% |
| 413 | RIZ\_ERYTHROID\_DIFFERENTIATION\_APOBEC2 |  | 27 | -0.31 | -0.95 | 0.490 | 0.496 | 1.000 | 2825 | tags=30%, list=18%, signal=36% |
| 414 | KEGG\_RENIN\_ANGIOTENSIN\_SYSTEM |  | 17 | -0.37 | -0.95 | 0.500 | 0.497 | 1.000 | 907 | tags=18%, list=6%, signal=19% |
| 415 | WANG\_TNF\_TARGETS |  | 23 | -0.35 | -0.95 | 0.548 | 0.497 | 1.000 | 2462 | tags=35%, list=16%, signal=41% |
| 416 | MOREAUX\_B\_LYMPHOCYTE\_MATURATION\_BY\_TACI\_UP |  | 74 | -0.25 | -0.95 | 1.000 | 0.505 | 1.000 | 1586 | tags=27%, list=10%, signal=30% |
| 417 | CASORELLI\_APL\_SECONDARY\_VS\_DE\_NOVO\_UP |  | 33 | -0.30 | -0.95 | 0.500 | 0.505 | 1.000 | 1710 | tags=33%, list=11%, signal=37% |
| 418 | KEGG\_ALDOSTERONE\_REGULATED\_SODIUM\_REABSORPTION |  | 39 | -0.27 | -0.94 | 0.622 | 0.506 | 1.000 | 2072 | tags=31%, list=13%, signal=35% |
| 419 | OHGUCHI\_LIVER\_HNF4A\_TARGETS\_UP |  | 41 | -0.29 | -0.94 | 0.500 | 0.512 | 1.000 | 1374 | tags=27%, list=9%, signal=29% |
| 420 | WANG\_NFKB\_TARGETS |  | 23 | -0.31 | -0.94 | 0.545 | 0.516 | 1.000 | 2870 | tags=30%, list=18%, signal=37% |
| 421 | GAVIN\_PDE3B\_TARGETS |  | 21 | -0.35 | -0.94 | 0.559 | 0.515 | 1.000 | 1059 | tags=19%, list=7%, signal=20% |
| 422 | ONO\_FOXP3\_TARGETS\_UP |  | 21 | -0.34 | -0.94 | 0.562 | 0.518 | 1.000 | 1890 | tags=24%, list=12%, signal=27% |
| 423 | LEE\_TARGETS\_OF\_PTCH1\_AND\_SUFU\_DN |  | 77 | -0.25 | -0.93 | 1.000 | 0.523 | 1.000 | 2665 | tags=30%, list=17%, signal=36% |
| 424 | REACTOME\_GLUTATHIONE\_CONJUGATION |  | 18 | -0.35 | -0.93 | 0.529 | 0.524 | 1.000 | 1451 | tags=33%, list=9%, signal=37% |
| 425 | RICKMAN\_HEAD\_AND\_NECK\_CANCER\_A |  | 84 | -0.24 | -0.93 | 1.000 | 0.531 | 1.000 | 1593 | tags=13%, list=10%, signal=14% |
| 426 | VANLOO\_SP3\_TARGETS\_DN |  | 82 | -0.30 | -0.93 | 1.000 | 0.530 | 1.000 | 1931 | tags=27%, list=12%, signal=30% |
| 427 | AMUNDSON\_GAMMA\_RADIATION\_RESISTANCE |  | 18 | -0.35 | -0.93 | 0.575 | 0.531 | 1.000 | 1596 | tags=22%, list=10%, signal=25% |
| 428 | RAMALHO\_STEMNESS\_DN |  | 67 | -0.26 | -0.93 | 0.750 | 0.531 | 1.000 | 2282 | tags=33%, list=14%, signal=38% |
| 429 | REACTOME\_SYNTHESIS\_OF\_BILE\_ACIDS\_AND\_BILE\_SALTS |  | 19 | -0.33 | -0.92 | 0.613 | 0.539 | 1.000 | 2624 | tags=32%, list=17%, signal=38% |
| 430 | KEGG\_SPHINGOLIPID\_METABOLISM |  | 35 | -0.30 | -0.92 | 0.579 | 0.538 | 1.000 | 1179 | tags=23%, list=7%, signal=25% |
| 431 | NAKAYAMA\_FGF2\_TARGETS |  | 25 | -0.31 | -0.91 | 0.648 | 0.550 | 1.000 | 1003 | tags=24%, list=6%, signal=26% |
| 432 | BRACHAT\_RESPONSE\_TO\_CAMPTOTHECIN\_UP |  | 25 | -0.30 | -0.91 | 0.667 | 0.554 | 1.000 | 955 | tags=20%, list=6%, signal=21% |
| 433 | STEGER\_ADIPOGENESIS\_UP |  | 20 | -0.34 | -0.91 | 0.619 | 0.563 | 1.000 | 1797 | tags=40%, list=11%, signal=45% |
| 434 | REACTOME\_SYNTHESIS\_OF\_BILE\_ACIDS\_AND\_BILE\_SALTS\_VIA\_7ALPHA\_HYDROXYCHOLESTEROL |  | 15 | -0.35 | -0.90 | 0.591 | 0.574 | 1.000 | 2624 | tags=33%, list=17%, signal=40% |
| 435 | VANASSE\_BCL2\_TARGETS\_UP |  | 33 | -0.29 | -0.90 | 0.634 | 0.573 | 1.000 | 415 | tags=12%, list=3%, signal=12% |
| 436 | REACTOME\_INTRINSIC\_PATHWAY |  | 17 | -0.34 | -0.89 | 0.617 | 0.587 | 1.000 | 2869 | tags=18%, list=18%, signal=22% |
| 437 | BUCKANOVICH\_T\_LYMPHOCYTE\_HOMING\_ON\_TUMOR\_DN |  | 23 | -0.31 | -0.89 | 0.707 | 0.598 | 1.000 | 897 | tags=13%, list=6%, signal=14% |
| 438 | ONDER\_CDH1\_TARGETS\_3\_UP |  | 17 | -0.32 | -0.88 | 0.638 | 0.597 | 1.000 | 939 | tags=18%, list=6%, signal=19% |
| 439 | REACTOME\_AMINE\_COMPOUND\_SLC\_TRANSPORTERS |  | 26 | -0.30 | -0.87 | 0.660 | 0.618 | 1.000 | 1481 | tags=15%, list=9%, signal=17% |
| 440 | REACTOME\_METAL\_ION\_SLC\_TRANSPORTERS |  | 22 | -0.31 | -0.87 | 0.722 | 0.629 | 1.000 | 2230 | tags=36%, list=14%, signal=42% |
| 441 | MEISSNER\_BRAIN\_ICP\_WITH\_H3K4ME3 |  | 24 | -0.29 | -0.86 | 0.720 | 0.634 | 1.000 | 436 | tags=13%, list=3%, signal=13% |
| 442 | KEGG\_GLYCOSAMINOGLYCAN\_BIOSYNTHESIS\_KERATAN\_SULFATE |  | 15 | -0.32 | -0.86 | 0.653 | 0.639 | 1.000 | 821 | tags=27%, list=5%, signal=28% |
| 443 | REACTOME\_GROWTH\_HORMONE\_RECEPTOR\_SIGNALING |  | 21 | -0.30 | -0.86 | 0.792 | 0.639 | 1.000 | 1562 | tags=29%, list=10%, signal=32% |
| 444 | REACTOME\_TRANSPORT\_OF\_VITAMINS\_NUCLEOSIDES\_AND\_RELATED\_MOLECULES |  | 30 | -0.28 | -0.85 | 0.771 | 0.644 | 1.000 | 1332 | tags=20%, list=8%, signal=22% |
| 445 | IGLESIAS\_E2F\_TARGETS\_DN |  | 15 | -0.33 | -0.85 | 0.700 | 0.651 | 1.000 | 453 | tags=13%, list=3%, signal=14% |
| 446 | REACTOME\_GLUCAGON\_TYPE\_LIGAND\_RECEPTORS |  | 33 | -0.28 | -0.85 | 0.750 | 0.656 | 1.000 | 1732 | tags=15%, list=11%, signal=17% |
| 447 | TAKEDA\_TARGETS\_OF\_NUP98\_HOXA9\_FUSION\_3D\_DN |  | 27 | -0.27 | -0.83 | 0.807 | 0.675 | 1.000 | 673 | tags=7%, list=4%, signal=8% |
| 448 | SCHLINGEMANN\_SKIN\_CARCINOGENESIS\_TPA\_DN |  | 27 | -0.29 | -0.83 | 0.727 | 0.673 | 1.000 | 2901 | tags=48%, list=18%, signal=59% |
| 449 | REACTOME\_ZINC\_TRANSPORTERS |  | 15 | -0.33 | -0.83 | 0.661 | 0.673 | 1.000 | 1423 | tags=27%, list=9%, signal=29% |
| 450 | NAKAJIMA\_EOSINOPHIL |  | 25 | -0.29 | -0.83 | 0.840 | 0.673 | 1.000 | 2214 | tags=20%, list=14%, signal=23% |
| 451 | POMEROY\_MEDULLOBLASTOMA\_PROGNOSIS\_UP |  | 42 | -0.25 | -0.83 | 0.895 | 0.677 | 1.000 | 2737 | tags=36%, list=17%, signal=43% |
| 452 | MEISSNER\_ES\_ICP\_WITH\_H3K4ME3 |  | 25 | -0.27 | -0.83 | 0.882 | 0.677 | 1.000 | 436 | tags=12%, list=3%, signal=12% |
| 453 | OHM\_METHYLATED\_IN\_ADULT\_CANCERS |  | 27 | -0.28 | -0.82 | 0.816 | 0.690 | 1.000 | 943 | tags=19%, list=6%, signal=20% |
| 454 | REACTOME\_SYNTHESIS\_OF\_PC |  | 18 | -0.29 | -0.82 | 0.790 | 0.690 | 1.000 | 1604 | tags=28%, list=10%, signal=31% |
| 455 | RUAN\_RESPONSE\_TO\_TROGLITAZONE\_DN |  | 19 | -0.30 | -0.81 | 0.860 | 0.704 | 1.000 | 1740 | tags=26%, list=11%, signal=30% |
| 456 | LEE\_DOUBLE\_POLAR\_THYMOCYTE |  | 23 | -0.27 | -0.80 | 0.902 | 0.713 | 1.000 | 1346 | tags=13%, list=9%, signal=14% |
| 457 | WEBER\_METHYLATED\_HCP\_IN\_FIBROBLAST\_DN |  | 34 | -0.23 | -0.79 | 0.977 | 0.723 | 1.000 | 12082 | tags=100%, list=77%, signal=428% |
| 458 | KLEIN\_TARGETS\_OF\_BCR\_ABL1\_FUSION |  | 41 | -0.25 | -0.78 | 0.947 | 0.731 | 1.000 | 2527 | tags=32%, list=16%, signal=38% |
| 459 | SARTIPY\_NORMAL\_AT\_INSULIN\_RESISTANCE\_DN |  | 16 | -0.30 | -0.78 | 0.762 | 0.731 | 1.000 | 1153 | tags=31%, list=7%, signal=34% |
| 460 | KUROZUMI\_RESPONSE\_TO\_ONCOCYTIC\_VIRUS\_AND\_CYCLIC\_RGD |  | 17 | -0.29 | -0.77 | 0.794 | 0.739 | 1.000 | 3660 | tags=29%, list=23%, signal=38% |
| 461 | KEGG\_GALACTOSE\_METABOLISM |  | 26 | -0.26 | -0.77 | 0.898 | 0.744 | 1.000 | 1690 | tags=23%, list=11%, signal=26% |
| 462 | REACTOME\_ACTIVATED\_NOTCH1\_TRANSMITS\_SIGNAL\_TO\_THE\_NUCLEUS |  | 25 | -0.26 | -0.77 | 0.919 | 0.745 | 1.000 | 2865 | tags=52%, list=18%, signal=63% |
| 463 | YIH\_RESPONSE\_TO\_ARSENITE\_C4 |  | 17 | -0.29 | -0.76 | 0.864 | 0.744 | 1.000 | 753 | tags=18%, list=5%, signal=19% |
| 464 | MIKKELSEN\_MEF\_ICP\_WITH\_H3K4ME3\_AND\_H3K27ME3 |  | 34 | -0.24 | -0.76 | 0.968 | 0.744 | 1.000 | 2088 | tags=12%, list=13%, signal=14% |
| 465 | REACTOME\_INHIBITION\_OF\_INSULIN\_SECRETION\_BY\_ADRENALINE\_NORADRENALINE |  | 25 | -0.24 | -0.76 | 0.938 | 0.746 | 1.000 | 2136 | tags=24%, list=14%, signal=28% |
| 466 | KANG\_FLUOROURACIL\_RESISTANCE\_DN |  | 15 | -0.29 | -0.73 | 0.848 | 0.769 | 1.000 | 1429 | tags=33%, list=9%, signal=37% |
| 467 | KEGG\_PRIMARY\_BILE\_ACID\_BIOSYNTHESIS |  | 16 | -0.27 | -0.73 | 0.907 | 0.772 | 1.000 | 2305 | tags=25%, list=15%, signal=29% |
| 468 | KEGG\_TASTE\_TRANSDUCTION |  | 42 | -0.21 | -0.72 | 0.929 | 0.774 | 1.000 | 3571 | tags=24%, list=23%, signal=31% |
| 469 | SU\_PLACENTA |  | 24 | -0.23 | -0.70 | 0.947 | 0.786 | 1.000 | 3694 | tags=42%, list=23%, signal=54% |
| 470 | LE\_NEURONAL\_DIFFERENTIATION\_UP |  | 18 | -0.25 | -0.69 | 0.965 | 0.793 | 1.000 | 201 | tags=6%, list=1%, signal=6% |
| 471 | WEBER\_METHYLATED\_HCP\_IN\_SPERM\_UP |  | 16 | -0.26 | -0.68 | 0.923 | 0.793 | 1.000 | 633 | tags=6%, list=4%, signal=7% |
| 472 | SU\_THYMUS |  | 16 | -0.25 | -0.68 | 0.954 | 0.792 | 1.000 | 2686 | tags=19%, list=17%, signal=23% |
| 473 | DING\_LUNG\_CANCER\_BY\_MUTATION\_RATE |  | 20 | -0.22 | -0.62 | 1.000 | 0.811 | 1.000 | 2790 | tags=35%, list=18%, signal=42% |
| 474 | BIOCARTA\_IL22BP\_PATHWAY |  | 16 | -0.23 | -0.57 | 0.981 | 0.814 | 1.000 | 788 | tags=13%, list=5%, signal=13% |
Table: Gene sets enriched in phenotype **na**[plain text format]****

  
