# Supplementary material for: Immunomic, genomic and transcriptomic characterization of CT26 colorectal carcinoma
Source: BMC Genomics. 2014 Mar 13;15(1):190. doi: 10.1186/1471-2164-15-190 (PMC4007559; doi:10.1186/1471-2164-15-190)
Supplement: Supplementary file 8 — Additional file 8: Contains the Gene Pattern gene set membership and enrichment values in an html format. The file index.html is the entry point. (ZIP 13 MB) [file 12864_2013_7028_MOESM8_ESM.zip › gsea_report_for_na_pos_1374178656685.html]

Report for na\_pos 1374178656685 [GSEA]

| GS  follow link to MSigDB | GS DETAILS | SIZE | ES | NES | NOM p-val | FDR q-val | FWER p-val | RANK AT MAX | LEADING EDGE || 1 | EGUCHI\_CELL\_CYCLE\_RB1\_TARGETS | Details ... | 23 | 0.90 | 1.81 | 0.000 | 0.000 | 0.000 | 1526 | tags=100%, list=10%, signal=111% |
| 2 | MORI\_LARGE\_PRE\_BII\_LYMPHOCYTE\_UP | Details ... | 81 | 0.82 | 1.79 | 0.000 | 0.001 | 0.002 | 2359 | tags=83%, list=15%, signal=97% |
| 3 | KAMMINGA\_EZH2\_TARGETS | Details ... | 40 | 0.83 | 1.76 | 0.000 | 0.001 | 0.004 | 2300 | tags=90%, list=15%, signal=105% |
| 4 | MORI\_IMMATURE\_B\_LYMPHOCYTE\_DN | Details ... | 84 | 0.80 | 1.73 | 0.000 | 0.001 | 0.005 | 2359 | tags=80%, list=15%, signal=93% |
| 5 | ABRAMSON\_INTERACT\_WITH\_AIRE | Details ... | 39 | 0.82 | 1.73 | 0.000 | 0.001 | 0.008 | 2648 | tags=90%, list=17%, signal=108% |
| 6 | REICHERT\_MITOSIS\_LIN9\_TARGETS | Details ... | 26 | 0.85 | 1.73 | 0.000 | 0.001 | 0.008 | 1966 | tags=85%, list=12%, signal=97% |
| 7 | DELPUECH\_FOXO3\_TARGETS\_DN | Details ... | 38 | 0.82 | 1.72 | 0.000 | 0.002 | 0.013 | 1867 | tags=68%, list=12%, signal=77% |
| 8 | ISHIDA\_E2F\_TARGETS | Details ... | 49 | 0.81 | 1.72 | 0.000 | 0.001 | 0.013 | 2266 | tags=88%, list=14%, signal=102% |
| 9 | REACTOME\_M\_G1\_TRANSITION | Details ... | 69 | 0.78 | 1.71 | 0.000 | 0.002 | 0.016 | 2609 | tags=78%, list=17%, signal=93% |
| 10 | PID\_AURORA\_B\_PATHWAY | Details ... | 38 | 0.82 | 1.71 | 0.000 | 0.001 | 0.016 | 1762 | tags=76%, list=11%, signal=86% |
| 11 | WINNEPENNINCKX\_MELANOMA\_METASTASIS\_UP | Details ... | 135 | 0.77 | 1.70 | 0.000 | 0.002 | 0.021 | 2527 | tags=74%, list=16%, signal=87% |
| 12 | RHODES\_UNDIFFERENTIATED\_CANCER | Details ... | 65 | 0.78 | 1.70 | 0.000 | 0.002 | 0.022 | 2266 | tags=75%, list=14%, signal=88% |
| 13 | FARMER\_BREAST\_CANCER\_CLUSTER\_2 | Details ... | 32 | 0.82 | 1.69 | 0.000 | 0.002 | 0.029 | 1918 | tags=84%, list=12%, signal=96% |
| 14 | CHANG\_CYCLING\_GENES | Details ... | 128 | 0.76 | 1.69 | 0.000 | 0.002 | 0.030 | 1959 | tags=63%, list=12%, signal=71% |
| 15 | REACTOME\_MITOTIC\_M\_M\_G1\_PHASES | Details ... | 155 | 0.76 | 1.69 | 0.000 | 0.002 | 0.032 | 2632 | tags=75%, list=17%, signal=90% |
| 16 | REACTOME\_DNA\_REPLICATION | Details ... | 174 | 0.76 | 1.69 | 0.000 | 0.002 | 0.032 | 3191 | tags=80%, list=20%, signal=100% |
| 17 | SOTIRIOU\_BREAST\_CANCER\_GRADE\_1\_VS\_3\_UP | Details ... | 136 | 0.76 | 1.69 | 0.000 | 0.002 | 0.033 | 3000 | tags=82%, list=19%, signal=100% |
| 18 | TANG\_SENESCENCE\_TP53\_TARGETS\_DN | Details ... | 50 | 0.79 | 1.69 | 0.000 | 0.002 | 0.034 | 1916 | tags=62%, list=12%, signal=70% |
| 19 | BILANGES\_SERUM\_AND\_RAPAMYCIN\_SENSITIVE\_GENES | Details ... | 57 | 0.78 | 1.69 | 0.000 | 0.002 | 0.034 | 2211 | tags=67%, list=14%, signal=77% |
| 20 | BURTON\_ADIPOGENESIS\_3 | Details ... | 95 | 0.77 | 1.69 | 0.000 | 0.002 | 0.034 | 1959 | tags=66%, list=12%, signal=75% |
| 21 | REACTOME\_SCFSKP2\_MEDIATED\_DEGRADATION\_OF\_P27\_P21 |  | 52 | 0.79 | 1.68 | 0.000 | 0.001 | 0.034 | 2609 | tags=77%, list=17%, signal=92% |
| 22 | PUJANA\_XPRSS\_INT\_NETWORK |  | 152 | 0.76 | 1.68 | 0.000 | 0.001 | 0.034 | 3000 | tags=78%, list=19%, signal=95% |
| 23 | MISSIAGLIA\_REGULATED\_BY\_METHYLATION\_DN |  | 105 | 0.77 | 1.68 | 0.000 | 0.001 | 0.035 | 2477 | tags=70%, list=16%, signal=83% |
| 24 | BURTON\_ADIPOGENESIS\_PEAK\_AT\_16HR |  | 40 | 0.80 | 1.68 | 0.000 | 0.001 | 0.037 | 1597 | tags=70%, list=10%, signal=78% |
| 25 | KEGG\_DNA\_REPLICATION |  | 33 | 0.81 | 1.68 | 0.000 | 0.001 | 0.038 | 2551 | tags=85%, list=16%, signal=101% |
| 26 | REACTOME\_TRANSLATION |  | 121 | 0.77 | 1.68 | 0.000 | 0.001 | 0.038 | 2624 | tags=74%, list=17%, signal=89% |
| 27 | KEGG\_RIBOSOME |  | 76 | 0.77 | 1.68 | 0.000 | 0.001 | 0.038 | 2746 | tags=78%, list=17%, signal=94% |
| 28 | HU\_ANGIOGENESIS\_DN |  | 32 | 0.81 | 1.68 | 0.000 | 0.001 | 0.041 | 1959 | tags=63%, list=12%, signal=71% |
| 29 | REACTOME\_G1\_S\_TRANSITION |  | 94 | 0.77 | 1.68 | 0.000 | 0.001 | 0.042 | 2609 | tags=74%, list=17%, signal=89% |
| 30 | LI\_WILMS\_TUMOR\_VS\_FETAL\_KIDNEY\_1\_DN |  | 151 | 0.76 | 1.67 | 0.000 | 0.001 | 0.044 | 2128 | tags=61%, list=14%, signal=70% |
| 31 | REACTOME\_S\_PHASE |  | 96 | 0.76 | 1.67 | 0.000 | 0.001 | 0.044 | 2609 | tags=74%, list=17%, signal=88% |
| 32 | LEE\_LIVER\_CANCER\_SURVIVAL\_DN |  | 154 | 0.75 | 1.67 | 0.000 | 0.001 | 0.044 | 2798 | tags=68%, list=18%, signal=81% |
| 33 | ZHANG\_TLX\_TARGETS\_36HR\_DN |  | 160 | 0.75 | 1.67 | 0.000 | 0.001 | 0.049 | 3324 | tags=83%, list=21%, signal=104% |
| 34 | MARKEY\_RB1\_ACUTE\_LOF\_DN |  | 204 | 0.75 | 1.67 | 0.000 | 0.001 | 0.050 | 2852 | tags=72%, list=18%, signal=86% |
| 35 | PUJANA\_BREAST\_CANCER\_WITH\_BRCA1\_MUTATED\_UP |  | 50 | 0.79 | 1.67 | 0.000 | 0.001 | 0.050 | 2893 | tags=88%, list=18%, signal=107% |
| 36 | REACTOME\_3\_UTR\_MEDIATED\_TRANSLATIONAL\_REGULATION |  | 83 | 0.77 | 1.67 | 0.000 | 0.001 | 0.053 | 2624 | tags=77%, list=17%, signal=92% |
| 37 | ZHANG\_TLX\_TARGETS\_60HR\_DN |  | 243 | 0.75 | 1.67 | 0.000 | 0.001 | 0.059 | 2852 | tags=71%, list=18%, signal=86% |
| 38 | KARLSSON\_TGFB1\_TARGETS\_UP |  | 108 | 0.76 | 1.67 | 0.000 | 0.001 | 0.059 | 2228 | tags=63%, list=14%, signal=73% |
| 39 | YU\_MYC\_TARGETS\_UP |  | 39 | 0.79 | 1.67 | 0.000 | 0.002 | 0.066 | 2965 | tags=90%, list=19%, signal=110% |
| 40 | REACTOME\_SCF\_BETA\_TRCP\_MEDIATED\_DEGRADATION\_OF\_EMI1 |  | 48 | 0.78 | 1.67 | 0.000 | 0.002 | 0.068 | 2609 | tags=75%, list=17%, signal=90% |
| 41 | WHITEFORD\_PEDIATRIC\_CANCER\_MARKERS |  | 108 | 0.76 | 1.66 | 0.000 | 0.002 | 0.069 | 2408 | tags=69%, list=15%, signal=80% |
| 42 | REACTOME\_VIF\_MEDIATED\_DEGRADATION\_OF\_APOBEC3G |  | 48 | 0.78 | 1.66 | 0.000 | 0.002 | 0.075 | 2609 | tags=75%, list=17%, signal=90% |
| 43 | REACTOME\_SYNTHESIS\_OF\_DNA |  | 81 | 0.76 | 1.66 | 0.000 | 0.002 | 0.076 | 2609 | tags=74%, list=17%, signal=88% |
| 44 | SEIDEN\_ONCOGENESIS\_BY\_MET |  | 71 | 0.77 | 1.66 | 0.000 | 0.002 | 0.078 | 1397 | tags=59%, list=9%, signal=65% |
| 45 | PUJANA\_BRCA\_CENTERED\_NETWORK |  | 108 | 0.76 | 1.66 | 0.000 | 0.002 | 0.080 | 3204 | tags=84%, list=20%, signal=105% |
| 46 | RHODES\_CANCER\_META\_SIGNATURE |  | 61 | 0.77 | 1.66 | 0.000 | 0.002 | 0.081 | 2056 | tags=66%, list=13%, signal=75% |
| 47 | REACTOME\_SRP\_DEPENDENT\_COTRANSLATIONAL\_PROTEIN\_TARGETING\_TO\_MEMBRANE |  | 95 | 0.76 | 1.66 | 0.000 | 0.002 | 0.081 | 2624 | tags=73%, list=17%, signal=87% |
| 48 | REACTOME\_METABOLISM\_OF\_RNA |  | 235 | 0.74 | 1.66 | 0.000 | 0.002 | 0.082 | 3204 | tags=75%, list=20%, signal=93% |
| 49 | REACTOME\_CROSS\_PRESENTATION\_OF\_SOLUBLE\_EXOGENOUS\_ANTIGENS\_ENDOSOMES |  | 46 | 0.78 | 1.66 | 0.000 | 0.002 | 0.083 | 2609 | tags=70%, list=17%, signal=83% |
| 50 | KOBAYASHI\_EGFR\_SIGNALING\_24HR\_DN |  | 229 | 0.74 | 1.66 | 0.000 | 0.002 | 0.088 | 3000 | tags=72%, list=19%, signal=88% |
| 51 | SCHLOSSER\_MYC\_TARGETS\_REPRESSED\_BY\_SERUM |  | 138 | 0.75 | 1.65 | 0.000 | 0.002 | 0.088 | 2179 | tags=64%, list=14%, signal=73% |
| 52 | REACTOME\_APC\_C\_CDC20\_MEDIATED\_DEGRADATION\_OF\_MITOTIC\_PROTEINS |  | 63 | 0.76 | 1.65 | 0.000 | 0.002 | 0.088 | 2609 | tags=73%, list=17%, signal=87% |
| 53 | HOFFMANN\_LARGE\_TO\_SMALL\_PRE\_BII\_LYMPHOCYTE\_UP |  | 149 | 0.75 | 1.65 | 0.000 | 0.002 | 0.088 | 2659 | tags=74%, list=17%, signal=88% |
| 54 | REACTOME\_NONSENSE\_MEDIATED\_DECAY\_ENHANCED\_BY\_THE\_EXON\_JUNCTION\_COMPLEX |  | 93 | 0.75 | 1.65 | 0.000 | 0.002 | 0.088 | 3193 | tags=81%, list=20%, signal=101% |
| 55 | REACTOME\_INFLUENZA\_LIFE\_CYCLE |  | 122 | 0.75 | 1.65 | 0.000 | 0.002 | 0.090 | 3152 | tags=80%, list=20%, signal=99% |
| 56 | REACTOME\_P53\_DEPENDENT\_G1\_DNA\_DAMAGE\_RESPONSE |  | 53 | 0.77 | 1.65 | 0.000 | 0.001 | 0.090 | 2609 | tags=74%, list=17%, signal=88% |
| 57 | GREENBAUM\_E2A\_TARGETS\_UP |  | 33 | 0.80 | 1.65 | 0.000 | 0.001 | 0.091 | 1988 | tags=73%, list=13%, signal=83% |
| 58 | REACTOME\_PEPTIDE\_CHAIN\_ELONGATION |  | 74 | 0.76 | 1.65 | 0.000 | 0.002 | 0.095 | 2624 | tags=76%, list=17%, signal=90% |
| 59 | LY\_AGING\_OLD\_DN |  | 53 | 0.78 | 1.65 | 0.000 | 0.002 | 0.096 | 2036 | tags=66%, list=13%, signal=76% |
| 60 | REACTOME\_ORC1\_REMOVAL\_FROM\_CHROMATIN |  | 59 | 0.77 | 1.65 | 0.000 | 0.002 | 0.101 | 2609 | tags=73%, list=17%, signal=87% |
| 61 | KONG\_E2F3\_TARGETS |  | 86 | 0.76 | 1.65 | 0.000 | 0.002 | 0.101 | 2879 | tags=76%, list=18%, signal=92% |
| 62 | BENPORATH\_PROLIFERATION |  | 128 | 0.75 | 1.65 | 0.000 | 0.002 | 0.102 | 2634 | tags=70%, list=17%, signal=83% |
| 63 | REACTOME\_ASSEMBLY\_OF\_THE\_PRE\_REPLICATIVE\_COMPLEX |  | 57 | 0.77 | 1.65 | 0.000 | 0.002 | 0.102 | 2609 | tags=74%, list=17%, signal=88% |
| 64 | ZHANG\_TLX\_TARGETS\_UP |  | 77 | 0.75 | 1.65 | 0.000 | 0.002 | 0.103 | 3202 | tags=81%, list=20%, signal=101% |
| 65 | REACTOME\_DESTABILIZATION\_OF\_MRNA\_BY\_AUF1\_HNRNP\_D0 |  | 48 | 0.78 | 1.65 | 0.000 | 0.002 | 0.115 | 2609 | tags=75%, list=17%, signal=90% |
| 66 | REACTOME\_MITOTIC\_G1\_G1\_S\_PHASES |  | 118 | 0.74 | 1.65 | 0.000 | 0.002 | 0.115 | 2609 | tags=68%, list=17%, signal=81% |
| 67 | REACTOME\_REGULATION\_OF\_MITOTIC\_CELL\_CYCLE |  | 74 | 0.76 | 1.64 | 0.000 | 0.002 | 0.120 | 2609 | tags=70%, list=17%, signal=84% |
| 68 | BERENJENO\_TRANSFORMED\_BY\_RHOA\_UP |  | 476 | 0.73 | 1.64 | 0.000 | 0.002 | 0.120 | 3036 | tags=68%, list=19%, signal=82% |
| 69 | KANG\_DOXORUBICIN\_RESISTANCE\_UP |  | 48 | 0.76 | 1.64 | 0.000 | 0.002 | 0.122 | 2999 | tags=81%, list=19%, signal=100% |
| 70 | ZHAN\_MULTIPLE\_MYELOMA\_PR\_UP |  | 38 | 0.79 | 1.64 | 0.000 | 0.002 | 0.126 | 2266 | tags=79%, list=14%, signal=92% |
| 71 | REACTOME\_METABOLISM\_OF\_MRNA |  | 193 | 0.74 | 1.64 | 0.000 | 0.002 | 0.130 | 3204 | tags=75%, list=20%, signal=93% |
| 72 | HU\_GENOTOXIC\_DAMAGE\_4HR |  | 34 | 0.79 | 1.64 | 0.000 | 0.002 | 0.131 | 1959 | tags=65%, list=12%, signal=74% |
| 73 | REACTOME\_INFLUENZA\_VIRAL\_RNA\_TRANSCRIPTION\_AND\_REPLICATION |  | 89 | 0.75 | 1.64 | 0.000 | 0.002 | 0.137 | 3152 | tags=81%, list=20%, signal=101% |
| 74 | KEGG\_SPLICEOSOME |  | 96 | 0.75 | 1.64 | 0.000 | 0.002 | 0.137 | 2926 | tags=78%, list=19%, signal=95% |
| 75 | WONG\_EMBRYONIC\_STEM\_CELL\_CORE |  | 304 | 0.74 | 1.64 | 0.000 | 0.002 | 0.143 | 2503 | tags=64%, list=16%, signal=75% |
| 76 | REACTOME\_MITOTIC\_PROMETAPHASE |  | 82 | 0.75 | 1.64 | 0.000 | 0.002 | 0.150 | 3111 | tags=80%, list=20%, signal=100% |
| 77 | MITSIADES\_RESPONSE\_TO\_APLIDIN\_DN |  | 217 | 0.73 | 1.64 | 0.000 | 0.002 | 0.150 | 2475 | tags=60%, list=16%, signal=70% |
| 78 | REACTOME\_CYCLIN\_E\_ASSOCIATED\_EVENTS\_DURING\_G1\_S\_TRANSITION\_ |  | 61 | 0.76 | 1.63 | 0.000 | 0.002 | 0.155 | 2609 | tags=70%, list=17%, signal=84% |
| 79 | REACTOME\_REGULATION\_OF\_APOPTOSIS |  | 56 | 0.76 | 1.63 | 0.000 | 0.002 | 0.155 | 3191 | tags=73%, list=20%, signal=91% |
| 80 | LE\_EGR2\_TARGETS\_UP |  | 102 | 0.75 | 1.63 | 0.000 | 0.002 | 0.165 | 2273 | tags=61%, list=14%, signal=71% |
| 81 | CROONQUIST\_IL6\_DEPRIVATION\_DN |  | 92 | 0.75 | 1.63 | 0.000 | 0.002 | 0.166 | 2366 | tags=67%, list=15%, signal=79% |
| 82 | REACTOME\_CYTOSOLIC\_TRNA\_AMINOACYLATION |  | 22 | 0.82 | 1.63 | 0.000 | 0.002 | 0.166 | 1617 | tags=77%, list=10%, signal=86% |
| 83 | REACTOME\_HOST\_INTERACTIONS\_OF\_HIV\_FACTORS |  | 115 | 0.74 | 1.63 | 0.000 | 0.002 | 0.166 | 2609 | tags=64%, list=17%, signal=77% |
| 84 | ROSTY\_CERVICAL\_CANCER\_PROLIFERATION\_CLUSTER |  | 129 | 0.74 | 1.63 | 0.000 | 0.002 | 0.172 | 2999 | tags=77%, list=19%, signal=94% |
| 85 | CROONQUIST\_NRAS\_SIGNALING\_DN |  | 70 | 0.75 | 1.63 | 0.000 | 0.002 | 0.175 | 2302 | tags=67%, list=15%, signal=78% |
| 86 | REACTOME\_CDK\_MEDIATED\_PHOSPHORYLATION\_AND\_REMOVAL\_OF\_CDC6 |  | 46 | 0.77 | 1.63 | 0.000 | 0.002 | 0.176 | 2609 | tags=74%, list=17%, signal=88% |
| 87 | REACTOME\_AUTODEGRADATION\_OF\_THE\_E3\_UBIQUITIN\_LIGASE\_COP1 |  | 47 | 0.77 | 1.63 | 0.000 | 0.002 | 0.181 | 2609 | tags=72%, list=17%, signal=86% |
| 88 | REACTOME\_EXTENSION\_OF\_TELOMERES |  | 23 | 0.82 | 1.63 | 0.000 | 0.002 | 0.181 | 2363 | tags=83%, list=15%, signal=97% |
| 89 | KEGG\_PROTEASOME |  | 43 | 0.79 | 1.63 | 0.000 | 0.002 | 0.185 | 2609 | tags=74%, list=17%, signal=89% |
| 90 | REACTOME\_ER\_PHAGOSOME\_PATHWAY |  | 56 | 0.76 | 1.63 | 0.000 | 0.002 | 0.187 | 2609 | tags=70%, list=17%, signal=83% |
| 91 | REACTOME\_CELL\_CYCLE\_MITOTIC |  | 284 | 0.73 | 1.63 | 0.000 | 0.002 | 0.188 | 2632 | tags=65%, list=17%, signal=77% |
| 92 | REACTOME\_PROCESSING\_OF\_CAPPED\_INTRON\_CONTAINING\_PRE\_MRNA |  | 107 | 0.74 | 1.63 | 0.000 | 0.002 | 0.191 | 3889 | tags=91%, list=25%, signal=120% |
| 93 | REACTOME\_CDT1\_ASSOCIATION\_WITH\_THE\_CDC6\_ORC\_ORIGIN\_COMPLEX |  | 48 | 0.76 | 1.62 | 0.000 | 0.002 | 0.191 | 2609 | tags=73%, list=17%, signal=87% |
| 94 | DAIRKEE\_CANCER\_PRONE\_RESPONSE\_BPA\_E2 |  | 107 | 0.74 | 1.62 | 0.000 | 0.002 | 0.191 | 2248 | tags=46%, list=14%, signal=53% |
| 95 | SCHLOSSER\_MYC\_TARGETS\_AND\_SERUM\_RESPONSE\_DN |  | 44 | 0.77 | 1.62 | 0.000 | 0.002 | 0.193 | 1953 | tags=64%, list=12%, signal=72% |
| 96 | FURUKAWA\_DUSP6\_TARGETS\_PCI35\_DN |  | 63 | 0.75 | 1.62 | 0.000 | 0.002 | 0.196 | 2266 | tags=56%, list=14%, signal=65% |
| 97 | BORCZUK\_MALIGNANT\_MESOTHELIOMA\_UP |  | 275 | 0.72 | 1.62 | 0.000 | 0.002 | 0.198 | 3061 | tags=67%, list=19%, signal=81% |
| 98 | MODY\_HIPPOCAMPUS\_PRENATAL |  | 40 | 0.78 | 1.62 | 0.000 | 0.002 | 0.198 | 2211 | tags=70%, list=14%, signal=81% |
| 99 | HOLLEMAN\_ASPARAGINASE\_RESISTANCE\_B\_ALL\_UP |  | 22 | 0.82 | 1.62 | 0.000 | 0.002 | 0.199 | 1091 | tags=50%, list=7%, signal=54% |
| 100 | BOYAULT\_LIVER\_CANCER\_SUBCLASS\_G3\_UP |  | 166 | 0.73 | 1.62 | 0.000 | 0.002 | 0.207 | 2548 | tags=66%, list=16%, signal=78% |
| 101 | TIEN\_INTESTINE\_PROBIOTICS\_2HR\_UP |  | 26 | 0.81 | 1.62 | 0.000 | 0.002 | 0.209 | 1581 | tags=58%, list=10%, signal=64% |
| 102 | REACTOME\_AUTODEGRADATION\_OF\_CDH1\_BY\_CDH1\_APC\_C |  | 55 | 0.75 | 1.62 | 0.000 | 0.002 | 0.229 | 2609 | tags=71%, list=17%, signal=85% |
| 103 | LI\_WILMS\_TUMOR\_VS\_FETAL\_KIDNEY\_2\_UP |  | 29 | 0.79 | 1.62 | 0.000 | 0.002 | 0.229 | 982 | tags=38%, list=6%, signal=40% |
| 104 | REACTOME\_REGULATION\_OF\_ORNITHINE\_DECARBOXYLASE\_ODC |  | 48 | 0.76 | 1.62 | 0.000 | 0.002 | 0.229 | 2986 | tags=73%, list=19%, signal=90% |
| 105 | MORI\_PRE\_BI\_LYMPHOCYTE\_UP |  | 74 | 0.75 | 1.62 | 0.000 | 0.002 | 0.229 | 2165 | tags=62%, list=14%, signal=72% |
| 106 | REACTOME\_MRNA\_SPLICING |  | 78 | 0.74 | 1.62 | 0.000 | 0.002 | 0.233 | 3889 | tags=92%, list=25%, signal=122% |
| 107 | KEGG\_NUCLEOTIDE\_EXCISION\_REPAIR |  | 42 | 0.77 | 1.62 | 0.000 | 0.002 | 0.237 | 2388 | tags=69%, list=15%, signal=81% |
| 108 | REACTOME\_CELL\_CYCLE\_CHECKPOINTS |  | 101 | 0.73 | 1.62 | 0.000 | 0.002 | 0.237 | 3621 | tags=82%, list=23%, signal=106% |
| 109 | CHIARADONNA\_NEOPLASTIC\_TRANSFORMATION\_KRAS\_UP |  | 116 | 0.74 | 1.61 | 0.000 | 0.002 | 0.253 | 1996 | tags=53%, list=13%, signal=60% |
| 110 | CHAUHAN\_RESPONSE\_TO\_METHOXYESTRADIOL\_UP |  | 46 | 0.77 | 1.61 | 0.000 | 0.003 | 0.263 | 2385 | tags=63%, list=15%, signal=74% |
| 111 | PENG\_LEUCINE\_DEPRIVATION\_DN |  | 172 | 0.73 | 1.61 | 0.000 | 0.003 | 0.265 | 2065 | tags=56%, list=13%, signal=64% |
| 112 | REACTOME\_TRANSPORT\_OF\_MATURE\_TRANSCRIPT\_TO\_CYTOPLASM |  | 44 | 0.76 | 1.61 | 0.000 | 0.003 | 0.265 | 2311 | tags=75%, list=15%, signal=88% |
| 113 | KANG\_AR\_TARGETS\_DN |  | 19 | 0.82 | 1.61 | 0.000 | 0.003 | 0.265 | 946 | tags=26%, list=6%, signal=28% |
| 114 | ZHOU\_CELL\_CYCLE\_GENES\_IN\_IR\_RESPONSE\_6HR |  | 72 | 0.74 | 1.61 | 0.000 | 0.003 | 0.273 | 3237 | tags=86%, list=21%, signal=108% |
| 115 | RAMALHO\_STEMNESS\_UP |  | 188 | 0.72 | 1.61 | 0.000 | 0.003 | 0.280 | 2799 | tags=65%, list=18%, signal=79% |
| 116 | REACTOME\_APC\_C\_CDH1\_MEDIATED\_DEGRADATION\_OF\_CDC20\_AND\_OTHER\_APC\_C\_CDH1\_TARGETED\_PROTEINS\_IN\_LATE\_MITOSIS\_EARLY\_G1 |  | 63 | 0.75 | 1.61 | 0.000 | 0.003 | 0.286 | 2986 | tags=75%, list=19%, signal=92% |
| 117 | TARTE\_PLASMA\_CELL\_VS\_PLASMABLAST\_DN |  | 285 | 0.72 | 1.61 | 0.000 | 0.003 | 0.290 | 2326 | tags=59%, list=15%, signal=68% |
| 118 | MILI\_PSEUDOPODIA\_HAPTOTAXIS\_UP |  | 425 | 0.71 | 1.61 | 0.000 | 0.003 | 0.299 | 3728 | tags=75%, list=24%, signal=96% |
| 119 | GRAHAM\_CML\_DIVIDING\_VS\_NORMAL\_QUIESCENT\_UP |  | 167 | 0.72 | 1.61 | 0.000 | 0.003 | 0.300 | 3206 | tags=65%, list=20%, signal=80% |
| 120 | WHITFIELD\_CELL\_CYCLE\_LITERATURE |  | 42 | 0.76 | 1.61 | 0.000 | 0.003 | 0.316 | 2366 | tags=74%, list=15%, signal=87% |
| 121 | REACTOME\_ACTIVATION\_OF\_THE\_PRE\_REPLICATIVE\_COMPLEX |  | 21 | 0.81 | 1.61 | 0.001 | 0.003 | 0.319 | 2519 | tags=90%, list=16%, signal=108% |
| 122 | GRAHAM\_NORMAL\_QUIESCENT\_VS\_NORMAL\_DIVIDING\_DN |  | 78 | 0.74 | 1.60 | 0.000 | 0.003 | 0.327 | 3182 | tags=73%, list=20%, signal=91% |
| 123 | AMUNDSON\_GAMMA\_RADIATION\_RESPONSE |  | 37 | 0.77 | 1.60 | 0.000 | 0.003 | 0.335 | 2266 | tags=73%, list=14%, signal=85% |
| 124 | REACTOME\_MRNA\_PROCESSING |  | 124 | 0.73 | 1.60 | 0.000 | 0.003 | 0.342 | 3889 | tags=84%, list=25%, signal=110% |
| 125 | DANG\_MYC\_TARGETS\_UP |  | 127 | 0.73 | 1.60 | 0.000 | 0.003 | 0.344 | 3189 | tags=73%, list=20%, signal=91% |
| 126 | IRITANI\_MAD1\_TARGETS\_DN |  | 40 | 0.76 | 1.60 | 0.000 | 0.003 | 0.347 | 2734 | tags=78%, list=17%, signal=94% |
| 127 | BILANGES\_SERUM\_RESPONSE\_TRANSLATION |  | 31 | 0.77 | 1.60 | 0.000 | 0.003 | 0.348 | 2039 | tags=45%, list=13%, signal=52% |
| 128 | HORIUCHI\_WTAP\_TARGETS\_DN |  | 271 | 0.72 | 1.60 | 0.000 | 0.003 | 0.354 | 2969 | tags=64%, list=19%, signal=77% |
| 129 | FEVR\_CTNNB1\_TARGETS\_DN |  | 469 | 0.71 | 1.60 | 0.000 | 0.003 | 0.359 | 2378 | tags=54%, list=15%, signal=62% |
| 130 | CHNG\_MULTIPLE\_MYELOMA\_HYPERPLOID\_UP |  | 42 | 0.76 | 1.60 | 0.000 | 0.003 | 0.359 | 2425 | tags=64%, list=15%, signal=76% |
| 131 | LY\_AGING\_PREMATURE\_DN |  | 28 | 0.79 | 1.60 | 0.001 | 0.003 | 0.360 | 2359 | tags=64%, list=15%, signal=75% |
| 132 | MANALO\_HYPOXIA\_DN |  | 253 | 0.71 | 1.60 | 0.000 | 0.003 | 0.361 | 2930 | tags=64%, list=19%, signal=77% |
| 133 | POMEROY\_MEDULLOBLASTOMA\_PROGNOSIS\_DN |  | 42 | 0.76 | 1.60 | 0.000 | 0.003 | 0.365 | 2039 | tags=62%, list=13%, signal=71% |
| 134 | SONG\_TARGETS\_OF\_IE86\_CMV\_PROTEIN |  | 57 | 0.75 | 1.60 | 0.000 | 0.003 | 0.367 | 2472 | tags=68%, list=16%, signal=81% |
| 135 | PUJANA\_BRCA2\_PCC\_NETWORK |  | 375 | 0.71 | 1.59 | 0.000 | 0.003 | 0.397 | 3204 | tags=62%, list=20%, signal=76% |
| 136 | REN\_BOUND\_BY\_E2F |  | 56 | 0.74 | 1.59 | 0.000 | 0.003 | 0.397 | 3765 | tags=88%, list=24%, signal=115% |
| 137 | PID\_MYC\_ACTIVPATHWAY |  | 76 | 0.74 | 1.59 | 0.000 | 0.003 | 0.398 | 2883 | tags=66%, list=18%, signal=80% |
| 138 | REACTOME\_SIGNALING\_BY\_WNT |  | 62 | 0.74 | 1.59 | 0.000 | 0.004 | 0.414 | 2609 | tags=66%, list=17%, signal=79% |
| 139 | WU\_APOPTOSIS\_BY\_CDKN1A\_VIA\_TP53 |  | 49 | 0.75 | 1.59 | 0.000 | 0.004 | 0.416 | 2879 | tags=78%, list=18%, signal=95% |
| 140 | PELLICCIOTTA\_HDAC\_IN\_ANTIGEN\_PRESENTATION\_DN |  | 45 | 0.75 | 1.59 | 0.000 | 0.004 | 0.424 | 2504 | tags=60%, list=16%, signal=71% |
| 141 | BIDUS\_METASTASIS\_UP |  | 177 | 0.71 | 1.59 | 0.000 | 0.004 | 0.442 | 2441 | tags=58%, list=15%, signal=67% |
| 142 | LEE\_EARLY\_T\_LYMPHOCYTE\_UP |  | 86 | 0.73 | 1.59 | 0.000 | 0.004 | 0.442 | 2999 | tags=65%, list=19%, signal=80% |
| 143 | REACTOME\_MRNA\_SPLICING\_MINOR\_PATHWAY |  | 36 | 0.76 | 1.59 | 0.000 | 0.004 | 0.449 | 2944 | tags=83%, list=19%, signal=102% |
| 144 | ZHOU\_CELL\_CYCLE\_GENES\_IN\_IR\_RESPONSE\_24HR |  | 109 | 0.72 | 1.59 | 0.000 | 0.004 | 0.456 | 2538 | tags=71%, list=16%, signal=84% |
| 145 | FOURNIER\_ACINAR\_DEVELOPMENT\_LATE\_2 |  | 249 | 0.71 | 1.59 | 0.000 | 0.004 | 0.464 | 3307 | tags=68%, list=21%, signal=85% |
| 146 | RHEIN\_ALL\_GLUCOCORTICOID\_THERAPY\_DN |  | 330 | 0.71 | 1.58 | 0.000 | 0.004 | 0.472 | 2366 | tags=53%, list=15%, signal=61% |
| 147 | FERREIRA\_EWINGS\_SARCOMA\_UNSTABLE\_VS\_STABLE\_UP |  | 145 | 0.71 | 1.58 | 0.000 | 0.004 | 0.474 | 2974 | tags=59%, list=19%, signal=72% |
| 148 | VANTVEER\_BREAST\_CANCER\_POOR\_PROGNOSIS |  | 44 | 0.75 | 1.58 | 0.000 | 0.004 | 0.479 | 1810 | tags=43%, list=11%, signal=49% |
| 149 | JI\_RESPONSE\_TO\_FSH\_DN |  | 55 | 0.74 | 1.58 | 0.000 | 0.004 | 0.483 | 2297 | tags=55%, list=15%, signal=64% |
| 150 | CAIRO\_PML\_TARGETS\_BOUND\_BY\_MYC\_UP |  | 23 | 0.79 | 1.58 | 0.000 | 0.004 | 0.487 | 2977 | tags=91%, list=19%, signal=112% |
| 151 | BOYAULT\_LIVER\_CANCER\_SUBCLASS\_G23\_UP |  | 48 | 0.74 | 1.58 | 0.000 | 0.004 | 0.488 | 1951 | tags=56%, list=12%, signal=64% |
| 152 | ZHANG\_BREAST\_CANCER\_PROGENITORS\_UP |  | 360 | 0.70 | 1.58 | 0.000 | 0.004 | 0.496 | 3223 | tags=64%, list=20%, signal=78% |
| 153 | PENG\_GLUTAMINE\_DEPRIVATION\_DN |  | 299 | 0.71 | 1.58 | 0.000 | 0.004 | 0.498 | 2356 | tags=55%, list=15%, signal=63% |
| 154 | CHICAS\_RB1\_TARGETS\_GROWING |  | 219 | 0.71 | 1.58 | 0.000 | 0.004 | 0.498 | 2325 | tags=46%, list=15%, signal=53% |
| 155 | REACTOME\_CELL\_CYCLE |  | 351 | 0.70 | 1.58 | 0.000 | 0.004 | 0.499 | 3215 | tags=63%, list=20%, signal=77% |
| 156 | MUELLER\_PLURINET |  | 261 | 0.71 | 1.58 | 0.000 | 0.004 | 0.509 | 2388 | tags=55%, list=15%, signal=64% |
| 157 | CHOI\_ATL\_STAGE\_PREDICTOR |  | 37 | 0.75 | 1.58 | 0.000 | 0.004 | 0.510 | 2263 | tags=57%, list=14%, signal=66% |
| 158 | REACTOME\_ACTIVATION\_OF\_NF\_KAPPAB\_IN\_B\_CELLS |  | 59 | 0.74 | 1.58 | 0.000 | 0.004 | 0.513 | 2609 | tags=63%, list=17%, signal=75% |
| 159 | REACTOME\_P53\_INDEPENDENT\_G1\_S\_DNA\_DAMAGE\_CHECKPOINT |  | 48 | 0.74 | 1.58 | 0.000 | 0.004 | 0.522 | 2609 | tags=69%, list=17%, signal=82% |
| 160 | BIOCARTA\_PROTEASOME\_PATHWAY |  | 27 | 0.78 | 1.58 | 0.000 | 0.004 | 0.523 | 2504 | tags=81%, list=16%, signal=97% |
| 161 | REACTOME\_ACTIVATION\_OF\_THE\_MRNA\_UPON\_BINDING\_OF\_THE\_CAP\_BINDING\_COMPLEX\_AND\_EIFS\_AND\_SUBSEQUENT\_BINDING\_TO\_43S |  | 41 | 0.75 | 1.58 | 0.000 | 0.004 | 0.525 | 2503 | tags=73%, list=16%, signal=87% |
| 162 | OUELLET\_OVARIAN\_CANCER\_INVASIVE\_VS\_LMP\_UP |  | 108 | 0.72 | 1.58 | 0.000 | 0.004 | 0.539 | 1980 | tags=56%, list=13%, signal=63% |
| 163 | SCHUHMACHER\_MYC\_TARGETS\_UP |  | 74 | 0.73 | 1.58 | 0.000 | 0.004 | 0.539 | 2235 | tags=59%, list=14%, signal=69% |
| 164 | KAUFFMANN\_DNA\_REPLICATION\_GENES |  | 124 | 0.71 | 1.58 | 0.000 | 0.005 | 0.550 | 2519 | tags=50%, list=16%, signal=59% |
| 165 | PENG\_RAPAMYCIN\_RESPONSE\_DN |  | 224 | 0.71 | 1.58 | 0.000 | 0.005 | 0.550 | 2623 | tags=58%, list=17%, signal=69% |
| 166 | VANTVEER\_BREAST\_CANCER\_METASTASIS\_DN |  | 105 | 0.72 | 1.57 | 0.000 | 0.005 | 0.555 | 2421 | tags=56%, list=15%, signal=66% |
| 167 | MENSSEN\_MYC\_TARGETS |  | 48 | 0.74 | 1.57 | 0.000 | 0.005 | 0.555 | 2039 | tags=63%, list=13%, signal=72% |
| 168 | SCIAN\_CELL\_CYCLE\_TARGETS\_OF\_TP53\_AND\_TP73\_DN |  | 21 | 0.80 | 1.57 | 0.001 | 0.005 | 0.561 | 2917 | tags=90%, list=19%, signal=111% |
| 169 | GARY\_CD5\_TARGETS\_DN |  | 373 | 0.70 | 1.57 | 0.000 | 0.005 | 0.568 | 3370 | tags=64%, list=21%, signal=80% |
| 170 | REACTOME\_INTERACTIONS\_OF\_VPR\_WITH\_HOST\_CELLULAR\_PROTEINS |  | 31 | 0.77 | 1.57 | 0.000 | 0.005 | 0.573 | 2311 | tags=74%, list=15%, signal=87% |
| 171 | REACTOME\_DNA\_STRAND\_ELONGATION |  | 27 | 0.77 | 1.57 | 0.001 | 0.005 | 0.592 | 2439 | tags=78%, list=15%, signal=92% |
| 172 | SIMBULAN\_PARP1\_TARGETS\_DN |  | 17 | 0.83 | 1.57 | 0.001 | 0.005 | 0.620 | 2266 | tags=76%, list=14%, signal=89% |
| 173 | PAL\_PRMT5\_TARGETS\_UP |  | 182 | 0.71 | 1.57 | 0.000 | 0.005 | 0.632 | 2355 | tags=56%, list=15%, signal=65% |
| 174 | WANG\_METASTASIS\_OF\_BREAST\_CANCER\_ESR1\_UP |  | 19 | 0.81 | 1.56 | 0.000 | 0.006 | 0.656 | 1272 | tags=53%, list=8%, signal=57% |
| 175 | REACTOME\_METABOLISM\_OF\_NON\_CODING\_RNA |  | 45 | 0.75 | 1.56 | 0.000 | 0.006 | 0.669 | 3354 | tags=82%, list=21%, signal=104% |
| 176 | BOHN\_PRIMARY\_IMMUNODEFICIENCY\_SYNDROM\_UP |  | 39 | 0.75 | 1.56 | 0.000 | 0.006 | 0.680 | 716 | tags=33%, list=5%, signal=35% |
| 177 | MALONEY\_RESPONSE\_TO\_17AAG\_DN |  | 71 | 0.72 | 1.56 | 0.000 | 0.006 | 0.707 | 2969 | tags=66%, list=19%, signal=81% |
| 178 | SARRIO\_EPITHELIAL\_MESENCHYMAL\_TRANSITION\_UP |  | 153 | 0.71 | 1.56 | 0.000 | 0.006 | 0.715 | 3209 | tags=62%, list=20%, signal=77% |
| 179 | BASSO\_B\_LYMPHOCYTE\_NETWORK |  | 124 | 0.71 | 1.56 | 0.000 | 0.006 | 0.726 | 2601 | tags=60%, list=17%, signal=71% |
| 180 | REACTOME\_ANTIGEN\_PROCESSING\_CROSS\_PRESENTATION |  | 69 | 0.72 | 1.56 | 0.000 | 0.006 | 0.727 | 2609 | tags=58%, list=17%, signal=69% |
| 181 | TIEN\_INTESTINE\_PROBIOTICS\_6HR\_UP |  | 49 | 0.74 | 1.56 | 0.000 | 0.006 | 0.727 | 1457 | tags=53%, list=9%, signal=58% |
| 182 | PID\_PLK1\_PATHWAY |  | 41 | 0.74 | 1.56 | 0.000 | 0.006 | 0.730 | 1954 | tags=59%, list=12%, signal=67% |
| 183 | BAKER\_HEMATOPOIESIS\_STAT3\_TARGETS |  | 16 | 0.81 | 1.56 | 0.003 | 0.006 | 0.732 | 1100 | tags=44%, list=7%, signal=47% |
| 184 | JIANG\_HYPOXIA\_VIA\_VHL |  | 28 | 0.76 | 1.56 | 0.001 | 0.006 | 0.732 | 2755 | tags=71%, list=17%, signal=86% |
| 185 | SMID\_BREAST\_CANCER\_LUMINAL\_A\_DN |  | 17 | 0.83 | 1.56 | 0.001 | 0.006 | 0.733 | 1031 | tags=65%, list=7%, signal=69% |
| 186 | YAO\_TEMPORAL\_RESPONSE\_TO\_PROGESTERONE\_CLUSTER\_11 |  | 90 | 0.71 | 1.56 | 0.000 | 0.006 | 0.734 | 1536 | tags=47%, list=10%, signal=51% |
| 187 | FLOTHO\_PEDIATRIC\_ALL\_THERAPY\_RESPONSE\_UP |  | 47 | 0.73 | 1.56 | 0.000 | 0.006 | 0.738 | 3247 | tags=68%, list=21%, signal=86% |
| 188 | SHEDDEN\_LUNG\_CANCER\_POOR\_SURVIVAL\_A6 |  | 401 | 0.69 | 1.56 | 0.000 | 0.006 | 0.743 | 3786 | tags=67%, list=24%, signal=85% |
| 189 | LY\_AGING\_MIDDLE\_DN |  | 16 | 0.81 | 1.56 | 0.001 | 0.006 | 0.746 | 2266 | tags=81%, list=14%, signal=95% |
| 190 | FERRANDO\_T\_ALL\_WITH\_MLL\_ENL\_FUSION\_DN |  | 80 | 0.71 | 1.55 | 0.000 | 0.006 | 0.747 | 2623 | tags=52%, list=17%, signal=63% |
| 191 | PARK\_HSC\_VS\_MULTIPOTENT\_PROGENITORS\_DN |  | 17 | 0.81 | 1.55 | 0.001 | 0.006 | 0.749 | 1783 | tags=65%, list=11%, signal=73% |
| 192 | GOBERT\_OLIGODENDROCYTE\_DIFFERENTIATION\_UP |  | 483 | 0.69 | 1.55 | 0.000 | 0.007 | 0.752 | 3712 | tags=66%, list=24%, signal=84% |
| 193 | KAUFFMANN\_MELANOMA\_RELAPSE\_UP |  | 54 | 0.73 | 1.55 | 0.000 | 0.007 | 0.757 | 3762 | tags=83%, list=24%, signal=109% |
| 194 | GRADE\_COLON\_AND\_RECTAL\_CANCER\_UP |  | 239 | 0.69 | 1.55 | 0.000 | 0.007 | 0.759 | 2266 | tags=54%, list=14%, signal=62% |
| 195 | REACTOME\_TRNA\_AMINOACYLATION |  | 40 | 0.74 | 1.55 | 0.000 | 0.007 | 0.770 | 2450 | tags=52%, list=16%, signal=62% |
| 196 | REACTOME\_HIV\_INFECTION |  | 185 | 0.70 | 1.55 | 0.000 | 0.007 | 0.773 | 3352 | tags=66%, list=21%, signal=83% |
| 197 | PEART\_HDAC\_PROLIFERATION\_CLUSTER\_DN |  | 71 | 0.72 | 1.55 | 0.000 | 0.007 | 0.773 | 3026 | tags=61%, list=19%, signal=75% |
| 198 | REACTOME\_CLEAVAGE\_OF\_GROWING\_TRANSCRIPT\_IN\_THE\_TERMINATION\_REGION\_ |  | 33 | 0.75 | 1.55 | 0.000 | 0.007 | 0.775 | 3602 | tags=85%, list=23%, signal=110% |
| 199 | ODONNELL\_TFRC\_TARGETS\_DN |  | 118 | 0.70 | 1.55 | 0.000 | 0.007 | 0.777 | 2347 | tags=47%, list=15%, signal=55% |
| 200 | REACTOME\_LAGGING\_STRAND\_SYNTHESIS |  | 17 | 0.81 | 1.55 | 0.002 | 0.007 | 0.784 | 2302 | tags=82%, list=15%, signal=96% |
| 201 | ZHANG\_RESPONSE\_TO\_CANTHARIDIN\_DN |  | 59 | 0.72 | 1.55 | 0.000 | 0.007 | 0.792 | 2106 | tags=49%, list=13%, signal=57% |
| 202 | ODONNELL\_TARGETS\_OF\_MYC\_AND\_TFRC\_DN |  | 40 | 0.74 | 1.55 | 0.000 | 0.007 | 0.795 | 2347 | tags=63%, list=15%, signal=73% |
| 203 | BHATTACHARYA\_EMBRYONIC\_STEM\_CELL |  | 77 | 0.71 | 1.55 | 0.000 | 0.007 | 0.797 | 1463 | tags=45%, list=9%, signal=50% |
| 204 | VERNELL\_RETINOBLASTOMA\_PATHWAY\_UP |  | 64 | 0.72 | 1.55 | 0.000 | 0.007 | 0.797 | 3765 | tags=80%, list=24%, signal=104% |
| 205 | NAKAMURA\_CANCER\_MICROENVIRONMENT\_DN |  | 43 | 0.74 | 1.55 | 0.000 | 0.007 | 0.804 | 2449 | tags=70%, list=16%, signal=82% |
| 206 | WANG\_TARGETS\_OF\_MLL\_CBP\_FUSION\_DN |  | 40 | 0.74 | 1.55 | 0.000 | 0.007 | 0.805 | 2792 | tags=63%, list=18%, signal=76% |
| 207 | REACTOME\_ACTIVATION\_OF\_ATR\_IN\_RESPONSE\_TO\_REPLICATION\_STRESS |  | 27 | 0.76 | 1.55 | 0.002 | 0.007 | 0.812 | 3705 | tags=96%, list=24%, signal=126% |
| 208 | REACTOME\_REGULATION\_OF\_MRNA\_STABILITY\_BY\_PROTEINS\_THAT\_BIND\_AU\_RICH\_ELEMENTS |  | 79 | 0.72 | 1.54 | 0.000 | 0.008 | 0.822 | 2626 | tags=62%, list=17%, signal=74% |
| 209 | CHEMNITZ\_RESPONSE\_TO\_PROSTAGLANDIN\_E2\_UP |  | 125 | 0.70 | 1.54 | 0.000 | 0.008 | 0.823 | 2985 | tags=59%, list=19%, signal=72% |
| 210 | BLUM\_RESPONSE\_TO\_SALIRASIB\_DN |  | 309 | 0.68 | 1.54 | 0.000 | 0.008 | 0.839 | 2844 | tags=56%, list=18%, signal=67% |
| 211 | NAKAYAMA\_SOFT\_TISSUE\_TUMORS\_PCA2\_UP |  | 81 | 0.71 | 1.54 | 0.000 | 0.008 | 0.840 | 2266 | tags=48%, list=14%, signal=56% |
| 212 | MMS\_MOUSE\_LYMPH\_HIGH\_4HRS\_UP |  | 35 | 0.74 | 1.54 | 0.002 | 0.008 | 0.844 | 1996 | tags=63%, list=13%, signal=72% |
| 213 | CAFFAREL\_RESPONSE\_TO\_THC\_DN |  | 27 | 0.76 | 1.54 | 0.000 | 0.008 | 0.849 | 2366 | tags=56%, list=15%, signal=65% |
| 214 | BRACHAT\_RESPONSE\_TO\_CAMPTOTHECIN\_DN |  | 38 | 0.73 | 1.54 | 0.001 | 0.008 | 0.853 | 2510 | tags=47%, list=16%, signal=56% |
| 215 | REACTOME\_HIV\_LIFE\_CYCLE |  | 110 | 0.69 | 1.54 | 0.000 | 0.008 | 0.853 | 3475 | tags=68%, list=22%, signal=87% |
| 216 | CHEN\_ETV5\_TARGETS\_TESTIS |  | 21 | 0.78 | 1.54 | 0.003 | 0.008 | 0.854 | 3029 | tags=62%, list=19%, signal=77% |
| 217 | REACTOME\_PREFOLDIN\_MEDIATED\_TRANSFER\_OF\_SUBSTRATE\_TO\_CCT\_TRIC |  | 24 | 0.77 | 1.54 | 0.002 | 0.008 | 0.854 | 1792 | tags=63%, list=11%, signal=70% |
| 218 | DAIRKEE\_CANCER\_PRONE\_RESPONSE\_BPA |  | 48 | 0.72 | 1.54 | 0.000 | 0.008 | 0.861 | 1274 | tags=40%, list=8%, signal=43% |
| 219 | REACTOME\_TRANSPORT\_OF\_MATURE\_MRNA\_DERIVED\_FROM\_AN\_INTRONLESS\_TRANSCRIPT |  | 33 | 0.74 | 1.54 | 0.000 | 0.008 | 0.868 | 2311 | tags=70%, list=15%, signal=82% |
| 220 | SHEN\_SMARCA2\_TARGETS\_UP |  | 361 | 0.68 | 1.54 | 0.000 | 0.008 | 0.870 | 2934 | tags=58%, list=19%, signal=69% |
| 221 | REACTOME\_FORMATION\_OF\_THE\_TERNARY\_COMPLEX\_AND\_SUBSEQUENTLY\_THE\_43S\_COMPLEX |  | 33 | 0.75 | 1.54 | 0.000 | 0.008 | 0.870 | 2503 | tags=70%, list=16%, signal=83% |
| 222 | YAO\_TEMPORAL\_RESPONSE\_TO\_PROGESTERONE\_CLUSTER\_14 |  | 123 | 0.70 | 1.54 | 0.000 | 0.008 | 0.872 | 2925 | tags=58%, list=19%, signal=70% |
| 223 | CHIANG\_LIVER\_CANCER\_SUBCLASS\_UNANNOTATED\_DN |  | 169 | 0.69 | 1.54 | 0.000 | 0.008 | 0.873 | 3474 | tags=65%, list=22%, signal=83% |
| 224 | REACTOME\_APC\_CDC20\_MEDIATED\_DEGRADATION\_OF\_NEK2A |  | 20 | 0.79 | 1.53 | 0.001 | 0.009 | 0.889 | 1471 | tags=65%, list=9%, signal=72% |
| 225 | CHIARETTI\_T\_ALL\_RELAPSE\_PROGNOSIS |  | 18 | 0.80 | 1.53 | 0.000 | 0.009 | 0.900 | 2273 | tags=61%, list=14%, signal=71% |
| 226 | BASAKI\_YBX1\_TARGETS\_UP |  | 252 | 0.69 | 1.53 | 0.000 | 0.009 | 0.900 | 3139 | tags=61%, list=20%, signal=75% |
| 227 | MATTIOLI\_MGUS\_VS\_PCL |  | 90 | 0.70 | 1.53 | 0.000 | 0.010 | 0.913 | 2698 | tags=51%, list=17%, signal=61% |
| 228 | REACTOME\_RNA\_POL\_II\_TRANSCRIPTION |  | 90 | 0.70 | 1.53 | 0.000 | 0.010 | 0.918 | 3918 | tags=77%, list=25%, signal=101% |
| 229 | YANG\_BREAST\_CANCER\_ESR1\_LASER\_DN |  | 47 | 0.72 | 1.53 | 0.000 | 0.010 | 0.923 | 1672 | tags=40%, list=11%, signal=45% |
| 230 | ZIRN\_TRETINOIN\_RESPONSE\_UP |  | 17 | 0.79 | 1.53 | 0.001 | 0.010 | 0.923 | 1110 | tags=29%, list=7%, signal=32% |
| 231 | DAZARD\_RESPONSE\_TO\_UV\_SCC\_UP |  | 107 | 0.69 | 1.53 | 0.000 | 0.010 | 0.924 | 2504 | tags=49%, list=16%, signal=57% |
| 232 | YAMASHITA\_LIVER\_CANCER\_WITH\_EPCAM\_UP |  | 45 | 0.72 | 1.53 | 0.000 | 0.010 | 0.926 | 1444 | tags=49%, list=9%, signal=54% |
| 233 | MOLENAAR\_TARGETS\_OF\_CCND1\_AND\_CDK4\_DN |  | 48 | 0.72 | 1.53 | 0.000 | 0.010 | 0.926 | 2785 | tags=67%, list=18%, signal=81% |
| 234 | WONG\_PROTEASOME\_GENE\_MODULE |  | 46 | 0.73 | 1.53 | 0.000 | 0.010 | 0.927 | 3239 | tags=67%, list=21%, signal=85% |
| 235 | ZHOU\_TNF\_SIGNALING\_4HR |  | 48 | 0.71 | 1.52 | 0.000 | 0.010 | 0.927 | 3297 | tags=63%, list=21%, signal=79% |
| 236 | REACTOME\_KINESINS |  | 24 | 0.76 | 1.52 | 0.001 | 0.011 | 0.932 | 1883 | tags=54%, list=12%, signal=61% |
| 237 | REACTOME\_NEP\_NS2\_INTERACTS\_WITH\_THE\_CELLULAR\_EXPORT\_MACHINERY |  | 27 | 0.75 | 1.52 | 0.001 | 0.011 | 0.934 | 2311 | tags=70%, list=15%, signal=82% |
| 238 | BOYAULT\_LIVER\_CANCER\_SUBCLASS\_G123\_UP |  | 41 | 0.72 | 1.52 | 0.000 | 0.011 | 0.936 | 2359 | tags=54%, list=15%, signal=63% |
| 239 | KEGG\_AMINOACYL\_TRNA\_BIOSYNTHESIS |  | 41 | 0.73 | 1.52 | 0.000 | 0.011 | 0.939 | 2450 | tags=49%, list=16%, signal=58% |
| 240 | REACTOME\_GLOBAL\_GENOMIC\_NER\_GG\_NER |  | 31 | 0.74 | 1.52 | 0.000 | 0.011 | 0.939 | 2388 | tags=65%, list=15%, signal=76% |
| 241 | SHAFFER\_IRF4\_TARGETS\_IN\_ACTIVATED\_DENDRITIC\_CELL |  | 57 | 0.71 | 1.52 | 0.000 | 0.011 | 0.942 | 2548 | tags=47%, list=16%, signal=56% |
| 242 | IKEDA\_MIR1\_TARGETS\_UP |  | 49 | 0.71 | 1.52 | 0.000 | 0.011 | 0.942 | 3469 | tags=63%, list=22%, signal=81% |
| 243 | REACTOME\_LATE\_PHASE\_OF\_HIV\_LIFE\_CYCLE |  | 99 | 0.69 | 1.52 | 0.000 | 0.011 | 0.943 | 4184 | tags=78%, list=27%, signal=105% |
| 244 | LI\_WILMS\_TUMOR\_ANAPLASTIC\_UP |  | 17 | 0.79 | 1.52 | 0.002 | 0.011 | 0.943 | 3351 | tags=94%, list=21%, signal=119% |
| 245 | REACTOME\_DEADENYLATION\_DEPENDENT\_MRNA\_DECAY |  | 42 | 0.72 | 1.52 | 0.000 | 0.011 | 0.948 | 3709 | tags=79%, list=24%, signal=102% |
| 246 | KEGG\_CELL\_CYCLE |  | 113 | 0.69 | 1.52 | 0.000 | 0.011 | 0.948 | 2273 | tags=50%, list=14%, signal=59% |
| 247 | TIEN\_INTESTINE\_PROBIOTICS\_24HR\_UP |  | 491 | 0.67 | 1.52 | 0.000 | 0.011 | 0.948 | 3182 | tags=57%, list=20%, signal=69% |
| 248 | REACTOME\_ANTIVIRAL\_MECHANISM\_BY\_IFN\_STIMULATED\_GENES |  | 61 | 0.71 | 1.52 | 0.000 | 0.011 | 0.948 | 3666 | tags=74%, list=23%, signal=96% |
| 249 | BURTON\_ADIPOGENESIS\_12 |  | 27 | 0.75 | 1.52 | 0.002 | 0.011 | 0.951 | 3463 | tags=78%, list=22%, signal=100% |
| 250 | CHIN\_BREAST\_CANCER\_COPY\_NUMBER\_UP |  | 25 | 0.76 | 1.52 | 0.001 | 0.011 | 0.957 | 2181 | tags=40%, list=14%, signal=46% |
| 251 | WANG\_TUMOR\_INVASIVENESS\_UP |  | 332 | 0.68 | 1.52 | 0.000 | 0.012 | 0.957 | 3194 | tags=57%, list=20%, signal=70% |
| 252 | STEIN\_ESRRA\_TARGETS\_RESPONSIVE\_TO\_ESTROGEN\_DN |  | 39 | 0.72 | 1.52 | 0.001 | 0.012 | 0.960 | 3705 | tags=72%, list=24%, signal=94% |
| 253 | FUJII\_YBX1\_TARGETS\_DN |  | 189 | 0.68 | 1.52 | 0.000 | 0.012 | 0.961 | 3000 | tags=56%, list=19%, signal=68% |
| 254 | REACTOME\_REGULATION\_OF\_GLUCOKINASE\_BY\_GLUCOKINASE\_REGULATORY\_PROTEIN |  | 27 | 0.74 | 1.52 | 0.002 | 0.012 | 0.962 | 3343 | tags=74%, list=21%, signal=94% |
| 255 | REACTOME\_G2\_M\_CHECKPOINTS |  | 32 | 0.73 | 1.51 | 0.001 | 0.012 | 0.963 | 3705 | tags=91%, list=24%, signal=118% |
| 256 | SHEPARD\_BMYB\_TARGETS |  | 64 | 0.70 | 1.51 | 0.000 | 0.012 | 0.966 | 2953 | tags=50%, list=19%, signal=61% |
| 257 | WEST\_ADRENOCORTICAL\_TUMOR\_MARKERS\_UP |  | 19 | 0.78 | 1.51 | 0.001 | 0.012 | 0.968 | 2226 | tags=53%, list=14%, signal=61% |
| 258 | REACTOME\_APOPTOSIS |  | 138 | 0.68 | 1.51 | 0.000 | 0.012 | 0.968 | 2297 | tags=46%, list=15%, signal=53% |
| 259 | GENTILE\_UV\_RESPONSE\_CLUSTER\_D8 |  | 34 | 0.74 | 1.51 | 0.000 | 0.012 | 0.972 | 1717 | tags=38%, list=11%, signal=43% |
| 260 | GAZDA\_DIAMOND\_BLACKFAN\_ANEMIA\_PROGENITOR\_DN |  | 52 | 0.71 | 1.51 | 0.000 | 0.012 | 0.972 | 3492 | tags=63%, list=22%, signal=81% |
| 261 | REACTOME\_DOWNSTREAM\_SIGNALING\_EVENTS\_OF\_B\_CELL\_RECEPTOR\_BCR |  | 87 | 0.69 | 1.51 | 0.000 | 0.012 | 0.972 | 3201 | tags=56%, list=20%, signal=70% |
| 262 | ZWANG\_EGF\_PERSISTENTLY\_UP |  | 25 | 0.75 | 1.51 | 0.000 | 0.012 | 0.973 | 1981 | tags=44%, list=13%, signal=50% |
| 263 | REACTOME\_TRANSPORT\_OF\_RIBONUCLEOPROTEINS\_INTO\_THE\_HOST\_NUCLEUS |  | 27 | 0.75 | 1.51 | 0.002 | 0.013 | 0.973 | 2311 | tags=70%, list=15%, signal=82% |
| 264 | RIZ\_ERYTHROID\_DIFFERENTIATION |  | 75 | 0.70 | 1.51 | 0.000 | 0.013 | 0.973 | 3032 | tags=55%, list=19%, signal=67% |
| 265 | OXFORD\_RALA\_OR\_RALB\_TARGETS\_UP |  | 43 | 0.71 | 1.51 | 0.000 | 0.013 | 0.973 | 3705 | tags=74%, list=24%, signal=97% |
| 266 | KEGG\_RNA\_DEGRADATION |  | 52 | 0.71 | 1.51 | 0.000 | 0.013 | 0.974 | 3204 | tags=67%, list=20%, signal=84% |
| 267 | DE\_YY1\_TARGETS\_DN |  | 84 | 0.70 | 1.51 | 0.000 | 0.013 | 0.974 | 3284 | tags=58%, list=21%, signal=73% |
| 268 | REACTOME\_INHIBITION\_OF\_THE\_PROTEOLYTIC\_ACTIVITY\_OF\_APC\_C\_REQUIRED\_FOR\_THE\_ONSET\_OF\_ANAPHASE\_BY\_MITOTIC\_SPINDLE\_CHECKPOINT\_COMPONENTS |  | 17 | 0.78 | 1.51 | 0.004 | 0.013 | 0.974 | 1471 | tags=65%, list=9%, signal=71% |
| 269 | HAHTOLA\_MYCOSIS\_FUNGOIDES\_CD4\_DN |  | 101 | 0.69 | 1.50 | 0.000 | 0.014 | 0.978 | 3300 | tags=62%, list=21%, signal=78% |
| 270 | BURTON\_ADIPOGENESIS\_PEAK\_AT\_24HR |  | 36 | 0.72 | 1.50 | 0.003 | 0.014 | 0.978 | 1099 | tags=50%, list=7%, signal=54% |
| 271 | RUIZ\_TNC\_TARGETS\_DN |  | 128 | 0.68 | 1.50 | 0.000 | 0.014 | 0.978 | 3202 | tags=63%, list=20%, signal=79% |
| 272 | REACTOME\_MRNA\_3\_END\_PROCESSING |  | 24 | 0.75 | 1.50 | 0.004 | 0.014 | 0.978 | 3602 | tags=92%, list=23%, signal=119% |
| 273 | SCHLOSSER\_MYC\_TARGETS\_AND\_SERUM\_RESPONSE\_UP |  | 44 | 0.71 | 1.50 | 0.000 | 0.014 | 0.978 | 2586 | tags=59%, list=16%, signal=71% |
| 274 | SENGUPTA\_NASOPHARYNGEAL\_CARCINOMA\_UP |  | 245 | 0.67 | 1.50 | 0.000 | 0.014 | 0.979 | 3005 | tags=47%, list=19%, signal=57% |
| 275 | DEN\_INTERACT\_WITH\_LCA5 |  | 25 | 0.74 | 1.50 | 0.006 | 0.014 | 0.979 | 2215 | tags=68%, list=14%, signal=79% |
| 276 | MILI\_PSEUDOPODIA\_CHEMOTAXIS\_UP |  | 55 | 0.70 | 1.50 | 0.001 | 0.014 | 0.983 | 3641 | tags=69%, list=23%, signal=90% |
| 277 | APRELIKOVA\_BRCA1\_TARGETS |  | 48 | 0.70 | 1.50 | 0.000 | 0.014 | 0.985 | 2018 | tags=54%, list=13%, signal=62% |
| 278 | WHITFIELD\_CELL\_CYCLE\_G2 |  | 145 | 0.68 | 1.50 | 0.000 | 0.014 | 0.985 | 2914 | tags=53%, list=19%, signal=65% |
| 279 | HEDENFALK\_BREAST\_CANCER\_HEREDITARY\_VS\_SPORADIC |  | 46 | 0.71 | 1.50 | 0.000 | 0.014 | 0.986 | 1563 | tags=43%, list=10%, signal=48% |
| 280 | MOREAUX\_B\_LYMPHOCYTE\_MATURATION\_BY\_TACI\_DN |  | 57 | 0.70 | 1.50 | 0.000 | 0.014 | 0.986 | 4053 | tags=79%, list=26%, signal=106% |
| 281 | SAKAI\_TUMOR\_INFILTRATING\_MONOCYTES\_DN |  | 74 | 0.69 | 1.50 | 0.000 | 0.015 | 0.987 | 3597 | tags=61%, list=23%, signal=78% |
| 282 | WEST\_ADRENOCORTICAL\_TUMOR\_UP |  | 266 | 0.67 | 1.50 | 0.000 | 0.015 | 0.988 | 3717 | tags=62%, list=24%, signal=79% |
| 283 | LAIHO\_COLORECTAL\_CANCER\_SERRATED\_UP |  | 100 | 0.68 | 1.50 | 0.000 | 0.016 | 0.992 | 2684 | tags=53%, list=17%, signal=63% |
| 284 | MOREAUX\_MULTIPLE\_MYELOMA\_BY\_TACI\_DN |  | 139 | 0.68 | 1.50 | 0.000 | 0.015 | 0.993 | 3593 | tags=65%, list=23%, signal=84% |
| 285 | PELLICCIOTTA\_HDAC\_IN\_ANTIGEN\_PRESENTATION\_UP |  | 63 | 0.69 | 1.50 | 0.001 | 0.016 | 0.993 | 3261 | tags=65%, list=21%, signal=82% |
| 286 | SASAKI\_ADULT\_T\_CELL\_LEUKEMIA |  | 158 | 0.67 | 1.50 | 0.000 | 0.016 | 0.993 | 2917 | tags=49%, list=19%, signal=59% |
| 287 | WIERENGA\_PML\_INTERACTOME |  | 37 | 0.72 | 1.50 | 0.000 | 0.016 | 0.993 | 1945 | tags=51%, list=12%, signal=58% |
| 288 | PID\_TCRCALCIUMPATHWAY |  | 26 | 0.74 | 1.49 | 0.002 | 0.016 | 0.993 | 424 | tags=19%, list=3%, signal=20% |
| 289 | PIONTEK\_PKD1\_TARGETS\_DN |  | 17 | 0.78 | 1.49 | 0.007 | 0.017 | 0.993 | 1792 | tags=47%, list=11%, signal=53% |
| 290 | KEGG\_PROTEIN\_EXPORT |  | 19 | 0.76 | 1.49 | 0.003 | 0.017 | 0.993 | 2543 | tags=68%, list=16%, signal=81% |
| 291 | RODRIGUES\_NTN1\_TARGETS\_UP |  | 15 | 0.78 | 1.49 | 0.008 | 0.017 | 0.993 | 1485 | tags=33%, list=9%, signal=37% |
| 292 | PUJANA\_BREAST\_CANCER\_LIT\_INT\_NETWORK |  | 96 | 0.68 | 1.49 | 0.000 | 0.017 | 0.993 | 3916 | tags=73%, list=25%, signal=96% |
| 293 | GAVIN\_FOXP3\_TARGETS\_CLUSTER\_T7 |  | 87 | 0.68 | 1.49 | 0.000 | 0.017 | 0.993 | 2951 | tags=53%, list=19%, signal=65% |
| 294 | PYEON\_HPV\_POSITIVE\_TUMORS\_UP |  | 71 | 0.69 | 1.49 | 0.000 | 0.017 | 0.993 | 3515 | tags=56%, list=22%, signal=72% |
| 295 | NAM\_FXYD5\_TARGETS\_DN |  | 17 | 0.78 | 1.49 | 0.007 | 0.017 | 0.994 | 2082 | tags=59%, list=13%, signal=68% |
| 296 | REACTOME\_PROCESSING\_OF\_CAPPED\_INTRONLESS\_PRE\_MRNA |  | 22 | 0.76 | 1.49 | 0.001 | 0.017 | 0.994 | 3602 | tags=82%, list=23%, signal=106% |
| 297 | LINDGREN\_BLADDER\_CANCER\_CLUSTER\_3\_UP |  | 277 | 0.66 | 1.49 | 0.000 | 0.018 | 0.994 | 3223 | tags=55%, list=20%, signal=68% |
| 298 | ZHAN\_MULTIPLE\_MYELOMA\_SUBGROUPS |  | 29 | 0.73 | 1.49 | 0.003 | 0.018 | 0.994 | 2255 | tags=55%, list=14%, signal=64% |
| 299 | MORI\_EMU\_MYC\_LYMPHOMA\_BY\_ONSET\_TIME\_UP |  | 88 | 0.68 | 1.49 | 0.000 | 0.018 | 0.995 | 3109 | tags=59%, list=20%, signal=73% |
| 300 | KEGG\_OOCYTE\_MEIOSIS |  | 104 | 0.67 | 1.49 | 0.000 | 0.018 | 0.995 | 2587 | tags=42%, list=16%, signal=50% |
| 301 | REACTOME\_FORMATION\_OF\_TUBULIN\_FOLDING\_INTERMEDIATES\_BY\_CCT\_TRIC |  | 18 | 0.78 | 1.49 | 0.002 | 0.018 | 0.995 | 1975 | tags=61%, list=13%, signal=70% |
| 302 | ONDER\_CDH1\_TARGETS\_1\_DN |  | 142 | 0.68 | 1.49 | 0.000 | 0.019 | 0.995 | 2548 | tags=44%, list=16%, signal=52% |
| 303 | ENK\_UV\_RESPONSE\_KERATINOCYTE\_DN |  | 437 | 0.66 | 1.48 | 0.000 | 0.019 | 0.995 | 3247 | tags=55%, list=21%, signal=67% |
| 304 | SENESE\_HDAC2\_TARGETS\_UP |  | 99 | 0.68 | 1.48 | 0.000 | 0.019 | 0.995 | 2475 | tags=43%, list=16%, signal=51% |
| 305 | FAELT\_B\_CLL\_WITH\_VH3\_21\_DN |  | 43 | 0.70 | 1.48 | 0.001 | 0.019 | 0.995 | 2619 | tags=47%, list=17%, signal=56% |
| 306 | KIM\_MYC\_AMPLIFICATION\_TARGETS\_UP |  | 176 | 0.67 | 1.48 | 0.000 | 0.019 | 0.995 | 2976 | tags=45%, list=19%, signal=55% |
| 307 | HSIAO\_HOUSEKEEPING\_GENES |  | 356 | 0.66 | 1.48 | 0.000 | 0.020 | 0.996 | 2224 | tags=46%, list=14%, signal=53% |
| 308 | CHIANG\_LIVER\_CANCER\_SUBCLASS\_PROLIFERATION\_UP |  | 153 | 0.67 | 1.48 | 0.000 | 0.020 | 0.996 | 2273 | tags=47%, list=14%, signal=54% |
| 309 | GROSS\_HYPOXIA\_VIA\_HIF1A\_UP |  | 67 | 0.68 | 1.48 | 0.000 | 0.020 | 0.996 | 1511 | tags=45%, list=10%, signal=49% |
| 310 | GRAHAM\_CML\_QUIESCENT\_VS\_CML\_DIVIDING\_UP |  | 19 | 0.76 | 1.48 | 0.002 | 0.020 | 0.996 | 1118 | tags=16%, list=7%, signal=17% |
| 311 | DER\_IFN\_BETA\_RESPONSE\_UP |  | 88 | 0.68 | 1.48 | 0.000 | 0.020 | 0.996 | 3036 | tags=45%, list=19%, signal=56% |
| 312 | PID\_ATR\_PATHWAY |  | 38 | 0.71 | 1.48 | 0.001 | 0.020 | 0.996 | 3705 | tags=76%, list=24%, signal=100% |
| 313 | SANA\_RESPONSE\_TO\_IFNG\_DN |  | 79 | 0.68 | 1.48 | 0.001 | 0.020 | 0.996 | 3179 | tags=63%, list=20%, signal=79% |
| 314 | XU\_HGF\_TARGETS\_INDUCED\_BY\_AKT1\_48HR\_DN |  | 20 | 0.76 | 1.48 | 0.004 | 0.020 | 0.996 | 1918 | tags=50%, list=12%, signal=57% |
| 315 | HEDENFALK\_BREAST\_CANCER\_BRACX\_DN |  | 18 | 0.77 | 1.48 | 0.006 | 0.020 | 0.996 | 1525 | tags=44%, list=10%, signal=49% |
| 316 | ZHAN\_VARIABLE\_EARLY\_DIFFERENTIATION\_GENES\_DN |  | 29 | 0.73 | 1.48 | 0.002 | 0.021 | 0.997 | 3166 | tags=76%, list=20%, signal=95% |
| 317 | TURASHVILI\_BREAST\_DUCTAL\_CARCINOMA\_VS\_LOBULAR\_NORMAL\_UP |  | 63 | 0.69 | 1.48 | 0.001 | 0.021 | 0.997 | 3712 | tags=56%, list=24%, signal=72% |
| 318 | PRAMOONJAGO\_SOX4\_TARGETS\_DN |  | 45 | 0.70 | 1.48 | 0.004 | 0.021 | 0.997 | 2917 | tags=49%, list=19%, signal=60% |
| 319 | PID\_ATM\_PATHWAY |  | 32 | 0.72 | 1.48 | 0.002 | 0.021 | 0.997 | 3621 | tags=78%, list=23%, signal=101% |
| 320 | BILANGES\_RAPAMYCIN\_SENSITIVE\_VIA\_TSC1\_AND\_TSC2 |  | 60 | 0.68 | 1.48 | 0.000 | 0.021 | 0.997 | 1998 | tags=45%, list=13%, signal=51% |
| 321 | HOLLEMAN\_VINCRISTINE\_RESISTANCE\_ALL\_DN |  | 18 | 0.76 | 1.47 | 0.010 | 0.022 | 0.997 | 1866 | tags=61%, list=12%, signal=69% |
| 322 | GARGALOVIC\_RESPONSE\_TO\_OXIDIZED\_PHOSPHOLIPIDS\_TURQUOISE\_DN |  | 47 | 0.69 | 1.47 | 0.002 | 0.021 | 0.997 | 2674 | tags=57%, list=17%, signal=69% |
| 323 | DOANE\_BREAST\_CANCER\_CLASSES\_DN |  | 33 | 0.72 | 1.47 | 0.001 | 0.021 | 0.997 | 1852 | tags=42%, list=12%, signal=48% |
| 324 | ABDELMOHSEN\_ELAVL4\_TARGETS |  | 16 | 0.79 | 1.47 | 0.008 | 0.022 | 0.997 | 1570 | tags=56%, list=10%, signal=62% |
| 325 | MORI\_MATURE\_B\_LYMPHOCYTE\_DN |  | 71 | 0.68 | 1.47 | 0.000 | 0.022 | 0.997 | 3040 | tags=54%, list=19%, signal=66% |
| 326 | CHANG\_CORE\_SERUM\_RESPONSE\_UP |  | 185 | 0.66 | 1.47 | 0.000 | 0.022 | 0.997 | 3280 | tags=57%, list=21%, signal=72% |
| 327 | DUTERTRE\_ESTRADIOL\_RESPONSE\_24HR\_UP |  | 294 | 0.66 | 1.47 | 0.000 | 0.022 | 0.997 | 3712 | tags=59%, list=24%, signal=76% |
| 328 | SHIPP\_DLBCL\_VS\_FOLLICULAR\_LYMPHOMA\_UP |  | 43 | 0.70 | 1.47 | 0.002 | 0.022 | 0.997 | 2266 | tags=60%, list=14%, signal=70% |
| 329 | YAO\_TEMPORAL\_RESPONSE\_TO\_PROGESTERONE\_CLUSTER\_10 |  | 55 | 0.69 | 1.47 | 0.001 | 0.022 | 0.998 | 2945 | tags=62%, list=19%, signal=76% |
| 330 | ZAMORA\_NOS2\_TARGETS\_UP |  | 65 | 0.68 | 1.47 | 0.000 | 0.022 | 0.999 | 3182 | tags=66%, list=20%, signal=83% |
| 331 | SAKAI\_CHRONIC\_HEPATITIS\_VS\_LIVER\_CANCER\_UP |  | 72 | 0.68 | 1.47 | 0.000 | 0.022 | 0.999 | 2228 | tags=50%, list=14%, signal=58% |
| 332 | MARTINEZ\_RESPONSE\_TO\_TRABECTEDIN\_DN |  | 237 | 0.66 | 1.47 | 0.000 | 0.022 | 0.999 | 4121 | tags=66%, list=26%, signal=88% |
| 333 | TRACEY\_RESISTANCE\_TO\_IFNA2\_DN |  | 26 | 0.72 | 1.47 | 0.001 | 0.023 | 0.999 | 2027 | tags=35%, list=13%, signal=40% |
| 334 | MACLACHLAN\_BRCA1\_TARGETS\_UP |  | 20 | 0.75 | 1.47 | 0.009 | 0.022 | 0.999 | 2165 | tags=60%, list=14%, signal=69% |
| 335 | JIANG\_AGING\_CEREBRAL\_CORTEX\_UP |  | 32 | 0.71 | 1.47 | 0.004 | 0.023 | 0.999 | 2909 | tags=59%, list=18%, signal=73% |
| 336 | REACTOME\_E2F\_MEDIATED\_REGULATION\_OF\_DNA\_REPLICATION |  | 23 | 0.73 | 1.47 | 0.006 | 0.023 | 0.999 | 3705 | tags=83%, list=24%, signal=108% |
| 337 | BACOLOD\_RESISTANCE\_TO\_ALKYLATING\_AGENTS\_DN |  | 49 | 0.69 | 1.47 | 0.002 | 0.023 | 0.999 | 2385 | tags=43%, list=15%, signal=50% |
| 338 | LU\_TUMOR\_ANGIOGENESIS\_UP |  | 24 | 0.73 | 1.47 | 0.004 | 0.024 | 1.000 | 1100 | tags=29%, list=7%, signal=31% |
| 339 | SMITH\_LIVER\_CANCER |  | 40 | 0.69 | 1.47 | 0.003 | 0.024 | 1.000 | 3303 | tags=55%, list=21%, signal=69% |
| 340 | SESTO\_RESPONSE\_TO\_UV\_C2 |  | 46 | 0.69 | 1.47 | 0.000 | 0.024 | 1.000 | 2775 | tags=54%, list=18%, signal=66% |
| 341 | TERAMOTO\_OPN\_TARGETS\_CLUSTER\_7 |  | 17 | 0.76 | 1.47 | 0.011 | 0.024 | 1.000 | 1809 | tags=47%, list=11%, signal=53% |
| 342 | SHAFFER\_IRF4\_TARGETS\_IN\_ACTIVATED\_B\_LYMPHOCYTE |  | 74 | 0.68 | 1.47 | 0.000 | 0.024 | 1.000 | 1741 | tags=45%, list=11%, signal=50% |
| 343 | MANN\_RESPONSE\_TO\_AMIFOSTINE\_UP |  | 20 | 0.75 | 1.47 | 0.007 | 0.024 | 1.000 | 2483 | tags=60%, list=16%, signal=71% |
| 344 | SIMBULAN\_UV\_RESPONSE\_IMMORTALIZED\_DN |  | 30 | 0.72 | 1.47 | 0.003 | 0.024 | 1.000 | 2977 | tags=63%, list=19%, signal=78% |
| 345 | JIANG\_TIP30\_TARGETS\_UP |  | 43 | 0.69 | 1.47 | 0.002 | 0.024 | 1.000 | 1656 | tags=35%, list=11%, signal=39% |
| 346 | FLECHNER\_BIOPSY\_KIDNEY\_TRANSPLANT\_OK\_VS\_DONOR\_UP |  | 489 | 0.65 | 1.46 | 0.000 | 0.024 | 1.000 | 3393 | tags=55%, list=22%, signal=67% |
| 347 | REACTOME\_TRANSCRIPTION\_COUPLED\_NER\_TC\_NER |  | 42 | 0.71 | 1.46 | 0.002 | 0.025 | 1.000 | 2388 | tags=60%, list=15%, signal=70% |
| 348 | REACTOME\_NUCLEOTIDE\_EXCISION\_REPAIR |  | 47 | 0.69 | 1.46 | 0.001 | 0.025 | 1.000 | 3802 | tags=72%, list=24%, signal=95% |
| 349 | COLLER\_MYC\_TARGETS\_UP |  | 22 | 0.74 | 1.46 | 0.007 | 0.026 | 1.000 | 2106 | tags=68%, list=13%, signal=79% |
| 350 | JISON\_SICKLE\_CELL\_DISEASE\_DN |  | 143 | 0.66 | 1.46 | 0.000 | 0.027 | 1.000 | 2425 | tags=41%, list=15%, signal=48% |
| 351 | LIANG\_HEMATOPOIESIS\_STEM\_CELL\_NUMBER\_LARGE\_VS\_TINY\_UP |  | 39 | 0.70 | 1.46 | 0.003 | 0.027 | 1.000 | 2917 | tags=46%, list=19%, signal=57% |
| 352 | REACTOME\_RNA\_POL\_II\_PRE\_TRANSCRIPTION\_EVENTS |  | 57 | 0.68 | 1.46 | 0.001 | 0.027 | 1.000 | 4184 | tags=75%, list=27%, signal=102% |
| 353 | KIM\_WT1\_TARGETS\_12HR\_DN |  | 179 | 0.65 | 1.46 | 0.000 | 0.027 | 1.000 | 3223 | tags=46%, list=20%, signal=58% |
| 354 | STONER\_ESOPHAGEAL\_CARCINOGENESIS\_UP |  | 33 | 0.71 | 1.46 | 0.003 | 0.027 | 1.000 | 2205 | tags=48%, list=14%, signal=56% |
| 355 | REACTOME\_PERK\_REGULATED\_GENE\_EXPRESSION |  | 26 | 0.73 | 1.46 | 0.001 | 0.027 | 1.000 | 2040 | tags=54%, list=13%, signal=62% |
| 356 | PID\_LIS1PATHWAY |  | 27 | 0.72 | 1.46 | 0.003 | 0.027 | 1.000 | 3665 | tags=63%, list=23%, signal=82% |
| 357 | GEORGES\_CELL\_CYCLE\_MIR192\_TARGETS |  | 57 | 0.68 | 1.46 | 0.001 | 0.027 | 1.000 | 3599 | tags=68%, list=23%, signal=88% |
| 358 | SU\_TESTIS |  | 65 | 0.68 | 1.46 | 0.000 | 0.028 | 1.000 | 3589 | tags=46%, list=23%, signal=60% |
| 359 | FOURNIER\_ACINAR\_DEVELOPMENT\_LATE\_DN |  | 21 | 0.74 | 1.46 | 0.008 | 0.028 | 1.000 | 1959 | tags=67%, list=12%, signal=76% |
| 360 | CHANDRAN\_METASTASIS\_TOP50\_UP |  | 31 | 0.71 | 1.46 | 0.002 | 0.028 | 1.000 | 3115 | tags=61%, list=20%, signal=76% |
| 361 | CHAUHAN\_RESPONSE\_TO\_METHOXYESTRADIOL\_DN |  | 89 | 0.66 | 1.45 | 0.000 | 0.029 | 1.000 | 2210 | tags=42%, list=14%, signal=48% |
| 362 | WHITFIELD\_CELL\_CYCLE\_G2\_M |  | 192 | 0.65 | 1.45 | 0.000 | 0.029 | 1.000 | 3420 | tags=55%, list=22%, signal=70% |
| 363 | MAYBURD\_RESPONSE\_TO\_L663536\_DN |  | 45 | 0.69 | 1.45 | 0.004 | 0.029 | 1.000 | 2449 | tags=53%, list=16%, signal=63% |
| 364 | CAFFAREL\_RESPONSE\_TO\_THC\_24HR\_5\_UP |  | 29 | 0.71 | 1.45 | 0.006 | 0.029 | 1.000 | 1980 | tags=52%, list=13%, signal=59% |
| 365 | IWANAGA\_E2F1\_TARGETS\_INDUCED\_BY\_SERUM |  | 25 | 0.73 | 1.45 | 0.007 | 0.030 | 1.000 | 3424 | tags=76%, list=22%, signal=97% |
| 366 | IIZUKA\_LIVER\_CANCER\_PROGRESSION\_G1\_G2\_DN |  | 25 | 0.73 | 1.45 | 0.006 | 0.030 | 1.000 | 3219 | tags=72%, list=20%, signal=90% |
| 367 | TIEN\_INTESTINE\_PROBIOTICS\_24HR\_DN |  | 192 | 0.65 | 1.45 | 0.000 | 0.031 | 1.000 | 3063 | tags=54%, list=19%, signal=66% |
| 368 | MILI\_PSEUDOPODIA |  | 34 | 0.71 | 1.45 | 0.004 | 0.031 | 1.000 | 3641 | tags=71%, list=23%, signal=92% |
| 369 | REACTOME\_DEADENYLATION\_OF\_MRNA |  | 18 | 0.75 | 1.45 | 0.010 | 0.031 | 1.000 | 3955 | tags=100%, list=25%, signal=133% |
| 370 | FRASOR\_RESPONSE\_TO\_SERM\_OR\_FULVESTRANT\_DN |  | 49 | 0.68 | 1.45 | 0.002 | 0.032 | 1.000 | 3229 | tags=67%, list=21%, signal=84% |
| 371 | MA\_PITUITARY\_FETAL\_VS\_ADULT\_UP |  | 27 | 0.70 | 1.45 | 0.007 | 0.032 | 1.000 | 2307 | tags=33%, list=15%, signal=39% |
| 372 | REACTOME\_FORMATION\_OF\_RNA\_POL\_II\_ELONGATION\_COMPLEX\_ |  | 41 | 0.68 | 1.45 | 0.001 | 0.032 | 1.000 | 3841 | tags=71%, list=24%, signal=93% |
| 373 | LABBE\_WNT3A\_TARGETS\_UP |  | 106 | 0.66 | 1.45 | 0.000 | 0.033 | 1.000 | 1996 | tags=38%, list=13%, signal=43% |
| 374 | REACTOME\_CHROMOSOME\_MAINTENANCE |  | 93 | 0.66 | 1.45 | 0.000 | 0.032 | 1.000 | 3215 | tags=49%, list=20%, signal=62% |
| 375 | SHEPARD\_CRUSH\_AND\_BURN\_MUTANT\_DN |  | 165 | 0.65 | 1.44 | 0.000 | 0.033 | 1.000 | 2822 | tags=39%, list=18%, signal=47% |
| 376 | SESTO\_RESPONSE\_TO\_UV\_C7 |  | 64 | 0.67 | 1.44 | 0.000 | 0.033 | 1.000 | 3040 | tags=56%, list=19%, signal=69% |
| 377 | PARK\_HSC\_VS\_MULTIPOTENT\_PROGENITORS\_UP |  | 18 | 0.74 | 1.44 | 0.013 | 0.033 | 1.000 | 3377 | tags=67%, list=21%, signal=85% |
| 378 | WILLERT\_WNT\_SIGNALING |  | 21 | 0.73 | 1.44 | 0.008 | 0.033 | 1.000 | 1387 | tags=33%, list=9%, signal=37% |
| 379 | AFFAR\_YY1\_TARGETS\_DN |  | 223 | 0.65 | 1.44 | 0.000 | 0.034 | 1.000 | 2822 | tags=40%, list=18%, signal=48% |
| 380 | KOBAYASHI\_EGFR\_SIGNALING\_6HR\_DN |  | 18 | 0.75 | 1.44 | 0.014 | 0.034 | 1.000 | 946 | tags=39%, list=6%, signal=41% |
| 381 | GAVIN\_FOXP3\_TARGETS\_CLUSTER\_P6 |  | 86 | 0.66 | 1.44 | 0.000 | 0.034 | 1.000 | 3182 | tags=56%, list=20%, signal=70% |
| 382 | HU\_GENOTOXIC\_DAMAGE\_24HR |  | 32 | 0.70 | 1.44 | 0.007 | 0.034 | 1.000 | 2484 | tags=53%, list=16%, signal=63% |
| 383 | REACTOME\_ELONGATION\_ARREST\_AND\_RECOVERY |  | 31 | 0.70 | 1.44 | 0.007 | 0.034 | 1.000 | 3841 | tags=74%, list=24%, signal=98% |
| 384 | GARCIA\_TARGETS\_OF\_FLI1\_AND\_DAX1\_DN |  | 149 | 0.65 | 1.44 | 0.000 | 0.035 | 1.000 | 3817 | tags=56%, list=24%, signal=74% |
| 385 | SHAFFER\_IRF4\_MULTIPLE\_MYELOMA\_PROGRAM |  | 35 | 0.68 | 1.44 | 0.005 | 0.035 | 1.000 | 3597 | tags=46%, list=23%, signal=59% |
| 386 | LI\_LUNG\_CANCER |  | 39 | 0.69 | 1.44 | 0.006 | 0.035 | 1.000 | 1871 | tags=46%, list=12%, signal=52% |
| 387 | PUIFFE\_INVASION\_INHIBITED\_BY\_ASCITES\_UP |  | 74 | 0.67 | 1.44 | 0.000 | 0.035 | 1.000 | 3525 | tags=57%, list=22%, signal=73% |
| 388 | XU\_HGF\_SIGNALING\_NOT\_VIA\_AKT1\_48HR\_DN |  | 15 | 0.77 | 1.44 | 0.010 | 0.036 | 1.000 | 1272 | tags=53%, list=8%, signal=58% |
| 389 | JUBAN\_TARGETS\_OF\_SPI1\_AND\_FLI1\_DN |  | 76 | 0.67 | 1.44 | 0.000 | 0.036 | 1.000 | 2384 | tags=49%, list=15%, signal=57% |
| 390 | KAUFFMANN\_DNA\_REPAIR\_GENES |  | 211 | 0.64 | 1.44 | 0.000 | 0.036 | 1.000 | 4302 | tags=63%, list=27%, signal=86% |
| 391 | HONRADO\_BREAST\_CANCER\_BRCA1\_VS\_BRCA2 |  | 18 | 0.75 | 1.44 | 0.014 | 0.036 | 1.000 | 2481 | tags=67%, list=16%, signal=79% |
| 392 | REACTOME\_ANTIGEN\_PROCESSING\_UBIQUITINATION\_PROTEASOME\_DEGRADATION |  | 184 | 0.65 | 1.44 | 0.000 | 0.037 | 1.000 | 2635 | tags=43%, list=17%, signal=51% |
| 393 | WAKASUGI\_HAVE\_ZNF143\_BINDING\_SITES |  | 57 | 0.67 | 1.44 | 0.002 | 0.037 | 1.000 | 4114 | tags=65%, list=26%, signal=88% |
| 394 | REACTOME\_DARPP\_32\_EVENTS |  | 23 | 0.71 | 1.44 | 0.011 | 0.037 | 1.000 | 2582 | tags=52%, list=16%, signal=62% |
| 395 | RAO\_BOUND\_BY\_SALL4\_ISOFORM\_A |  | 150 | 0.65 | 1.44 | 0.000 | 0.037 | 1.000 | 2942 | tags=30%, list=19%, signal=37% |
| 396 | WELCSH\_BRCA1\_TARGETS\_DN |  | 123 | 0.65 | 1.44 | 0.000 | 0.037 | 1.000 | 2692 | tags=52%, list=17%, signal=62% |
| 397 | FINETTI\_BREAST\_CANCER\_KINOME\_RED |  | 15 | 0.76 | 1.43 | 0.023 | 0.037 | 1.000 | 3707 | tags=93%, list=24%, signal=122% |
| 398 | MULLIGAN\_NTF3\_SIGNALING\_VIA\_INSR\_AND\_IGF1R\_UP |  | 21 | 0.73 | 1.43 | 0.007 | 0.038 | 1.000 | 2235 | tags=57%, list=14%, signal=67% |
| 399 | DACOSTA\_UV\_RESPONSE\_VIA\_ERCC3\_COMMON\_DN |  | 418 | 0.64 | 1.43 | 0.000 | 0.038 | 1.000 | 4772 | tags=68%, list=30%, signal=96% |
| 400 | ROME\_INSULIN\_TARGETS\_IN\_MUSCLE\_UP |  | 369 | 0.64 | 1.43 | 0.000 | 0.038 | 1.000 | 3324 | tags=49%, list=21%, signal=61% |
| 401 | PENG\_GLUCOSE\_DEPRIVATION\_DN |  | 152 | 0.64 | 1.43 | 0.000 | 0.039 | 1.000 | 2357 | tags=41%, list=15%, signal=48% |
| 402 | VANHARANTA\_UTERINE\_FIBROID\_WITH\_7Q\_DELETION\_UP |  | 60 | 0.67 | 1.43 | 0.000 | 0.039 | 1.000 | 3275 | tags=58%, list=21%, signal=73% |
| 403 | CAFFAREL\_RESPONSE\_TO\_THC\_24HR\_5\_DN |  | 51 | 0.68 | 1.43 | 0.003 | 0.039 | 1.000 | 3012 | tags=55%, list=19%, signal=68% |
| 404 | REACTOME\_RNA\_POL\_II\_TRANSCRIPTION\_PRE\_INITIATION\_AND\_PROMOTER\_OPENING |  | 38 | 0.69 | 1.43 | 0.004 | 0.039 | 1.000 | 4184 | tags=82%, list=27%, signal=111% |
| 405 | REACTOME\_GLUCOSE\_TRANSPORT |  | 38 | 0.69 | 1.43 | 0.003 | 0.039 | 1.000 | 2311 | tags=45%, list=15%, signal=52% |
| 406 | REACTOME\_MYOGENESIS |  | 24 | 0.71 | 1.43 | 0.015 | 0.039 | 1.000 | 2626 | tags=42%, list=17%, signal=50% |
| 407 | REACTOME\_DNA\_REPAIR |  | 100 | 0.65 | 1.43 | 0.000 | 0.040 | 1.000 | 3868 | tags=63%, list=25%, signal=83% |
| 408 | SUNG\_METASTASIS\_STROMA\_DN |  | 41 | 0.68 | 1.43 | 0.002 | 0.040 | 1.000 | 2974 | tags=54%, list=19%, signal=66% |
| 409 | REACTOME\_ACTIVATED\_POINT\_MUTANTS\_OF\_FGFR2 |  | 16 | 0.75 | 1.43 | 0.013 | 0.040 | 1.000 | 129 | tags=6%, list=1%, signal=6% |
| 410 | WHITFIELD\_CELL\_CYCLE\_M\_G1 |  | 124 | 0.65 | 1.43 | 0.000 | 0.041 | 1.000 | 3195 | tags=51%, list=20%, signal=63% |
| 411 | IKEDA\_MIR133\_TARGETS\_UP |  | 43 | 0.68 | 1.43 | 0.001 | 0.041 | 1.000 | 3396 | tags=60%, list=22%, signal=77% |
| 412 | REACTOME\_INTRINSIC\_PATHWAY\_FOR\_APOPTOSIS |  | 28 | 0.70 | 1.43 | 0.009 | 0.041 | 1.000 | 2178 | tags=43%, list=14%, signal=50% |
| 413 | NAKAMURA\_TUMOR\_ZONE\_PERIPHERAL\_VS\_CENTRAL\_UP |  | 239 | 0.64 | 1.43 | 0.000 | 0.041 | 1.000 | 3201 | tags=52%, list=20%, signal=64% |
| 414 | ZUCCHI\_METASTASIS\_UP |  | 36 | 0.69 | 1.43 | 0.001 | 0.042 | 1.000 | 1725 | tags=42%, list=11%, signal=47% |
| 415 | VECCHI\_GASTRIC\_CANCER\_EARLY\_UP |  | 369 | 0.63 | 1.43 | 0.000 | 0.042 | 1.000 | 3012 | tags=50%, list=19%, signal=60% |
| 416 | WATANABE\_RECTAL\_CANCER\_RADIOTHERAPY\_RESPONSIVE\_DN |  | 85 | 0.65 | 1.43 | 0.000 | 0.042 | 1.000 | 2318 | tags=45%, list=15%, signal=52% |
| 417 | PID\_TELOMERASEPATHWAY |  | 65 | 0.66 | 1.43 | 0.001 | 0.042 | 1.000 | 3320 | tags=57%, list=21%, signal=72% |
| 418 | PID\_BARD1PATHWAY |  | 29 | 0.70 | 1.43 | 0.009 | 0.042 | 1.000 | 4275 | tags=83%, list=27%, signal=113% |
| 419 | REACTOME\_G1\_PHASE |  | 33 | 0.69 | 1.43 | 0.008 | 0.042 | 1.000 | 3189 | tags=61%, list=20%, signal=76% |
| 420 | SCHAEFFER\_PROSTATE\_DEVELOPMENT\_AND\_CANCER\_BOX4\_DN |  | 28 | 0.70 | 1.43 | 0.016 | 0.042 | 1.000 | 1609 | tags=43%, list=10%, signal=48% |
| 421 | ZHAN\_MULTIPLE\_MYELOMA\_HP\_DN |  | 38 | 0.69 | 1.43 | 0.007 | 0.042 | 1.000 | 2217 | tags=32%, list=14%, signal=37% |
| 422 | HEDENFALK\_BREAST\_CANCER\_BRCA1\_VS\_BRCA2 |  | 149 | 0.64 | 1.42 | 0.000 | 0.042 | 1.000 | 3189 | tags=53%, list=20%, signal=66% |
| 423 | KEGG\_MISMATCH\_REPAIR |  | 22 | 0.72 | 1.42 | 0.013 | 0.042 | 1.000 | 2302 | tags=64%, list=15%, signal=74% |
| 424 | GOLDRATH\_ANTIGEN\_RESPONSE |  | 315 | 0.63 | 1.42 | 0.000 | 0.042 | 1.000 | 3057 | tags=49%, list=19%, signal=59% |
| 425 | OSMAN\_BLADDER\_CANCER\_UP |  | 349 | 0.63 | 1.42 | 0.000 | 0.042 | 1.000 | 3490 | tags=49%, list=22%, signal=61% |
| 426 | SILIGAN\_TARGETS\_OF\_EWS\_FLI1\_FUSION\_DN |  | 16 | 0.75 | 1.42 | 0.020 | 0.042 | 1.000 | 835 | tags=31%, list=5%, signal=33% |
| 427 | REACTOME\_DOWNREGULATION\_OF\_SMAD2\_3\_SMAD4\_TRANSCRIPTIONAL\_ACTIVITY |  | 19 | 0.72 | 1.42 | 0.020 | 0.042 | 1.000 | 3865 | tags=79%, list=25%, signal=104% |
| 428 | ZHANG\_PROLIFERATING\_VS\_QUIESCENT |  | 50 | 0.67 | 1.42 | 0.002 | 0.042 | 1.000 | 2266 | tags=46%, list=14%, signal=54% |
| 429 | REACTOME\_G0\_AND\_EARLY\_G1 |  | 22 | 0.71 | 1.42 | 0.010 | 0.042 | 1.000 | 3705 | tags=73%, list=24%, signal=95% |
| 430 | NIKOLSKY\_BREAST\_CANCER\_7Q21\_Q22\_AMPLICON |  | 61 | 0.66 | 1.42 | 0.001 | 0.044 | 1.000 | 3079 | tags=38%, list=20%, signal=47% |
| 431 | PID\_PRLSIGNALINGEVENTSPATHWAY |  | 23 | 0.71 | 1.42 | 0.016 | 0.044 | 1.000 | 2587 | tags=57%, list=16%, signal=68% |
| 432 | FLECHNER\_PBL\_KIDNEY\_TRANSPLANT\_OK\_VS\_DONOR\_UP |  | 139 | 0.64 | 1.42 | 0.000 | 0.044 | 1.000 | 3191 | tags=51%, list=20%, signal=63% |
| 433 | RAHMAN\_TP53\_TARGETS\_PHOSPHORYLATED |  | 18 | 0.74 | 1.42 | 0.014 | 0.045 | 1.000 | 1808 | tags=61%, list=11%, signal=69% |
| 434 | CAFFAREL\_RESPONSE\_TO\_THC\_UP |  | 29 | 0.70 | 1.42 | 0.010 | 0.045 | 1.000 | 3750 | tags=66%, list=24%, signal=86% |
| 435 | SCHRAMM\_INHBA\_TARGETS\_DN |  | 24 | 0.71 | 1.42 | 0.016 | 0.045 | 1.000 | 2048 | tags=46%, list=13%, signal=53% |
| 436 | BENPORATH\_ES\_1 |  | 323 | 0.63 | 1.42 | 0.000 | 0.045 | 1.000 | 3177 | tags=44%, list=20%, signal=54% |
| 437 | OLSSON\_E2F3\_TARGETS\_DN |  | 37 | 0.68 | 1.42 | 0.011 | 0.045 | 1.000 | 3189 | tags=65%, list=20%, signal=81% |
| 438 | KEGG\_BASAL\_TRANSCRIPTION\_FACTORS |  | 31 | 0.69 | 1.42 | 0.014 | 0.045 | 1.000 | 4184 | tags=71%, list=27%, signal=96% |
| 439 | REACTOME\_METABOLISM\_OF\_PROTEINS |  | 377 | 0.63 | 1.42 | 0.000 | 0.045 | 1.000 | 3198 | tags=46%, list=20%, signal=56% |
| 440 | PID\_E2F\_PATHWAY |  | 65 | 0.65 | 1.42 | 0.003 | 0.045 | 1.000 | 3189 | tags=55%, list=20%, signal=69% |
| 441 | KANG\_DOXORUBICIN\_RESISTANCE\_DN |  | 17 | 0.73 | 1.42 | 0.021 | 0.045 | 1.000 | 3172 | tags=76%, list=20%, signal=96% |
| 442 | LIANG\_HEMATOPOIESIS\_STEM\_CELL\_NUMBER\_LARGE\_VS\_TINY\_DN |  | 39 | 0.69 | 1.42 | 0.008 | 0.045 | 1.000 | 2109 | tags=38%, list=13%, signal=44% |
| 443 | KEGG\_RNA\_POLYMERASE |  | 27 | 0.71 | 1.42 | 0.008 | 0.045 | 1.000 | 3943 | tags=85%, list=25%, signal=113% |
| 444 | WILCOX\_PRESPONSE\_TO\_ROGESTERONE\_UP |  | 127 | 0.64 | 1.42 | 0.000 | 0.046 | 1.000 | 3792 | tags=52%, list=24%, signal=68% |
| 445 | LE\_NEURONAL\_DIFFERENTIATION\_DN |  | 19 | 0.73 | 1.42 | 0.025 | 0.046 | 1.000 | 3373 | tags=63%, list=21%, signal=80% |
| 446 | IKEDA\_MIR30\_TARGETS\_UP |  | 111 | 0.64 | 1.42 | 0.000 | 0.046 | 1.000 | 4228 | tags=55%, list=27%, signal=75% |
| 447 | AZARE\_STAT3\_TARGETS |  | 21 | 0.72 | 1.42 | 0.022 | 0.046 | 1.000 | 3216 | tags=62%, list=20%, signal=78% |
| 448 | REACTOME\_SIGNALING\_BY\_THE\_B\_CELL\_RECEPTOR\_BCR |  | 115 | 0.65 | 1.42 | 0.000 | 0.047 | 1.000 | 3201 | tags=46%, list=20%, signal=57% |
| 449 | SHAFFER\_IRF4\_TARGETS\_IN\_MYELOMA\_VS\_MATURE\_B\_LYMPHOCYTE |  | 94 | 0.65 | 1.41 | 0.000 | 0.047 | 1.000 | 2017 | tags=41%, list=13%, signal=47% |
| 450 | CROMER\_METASTASIS\_UP |  | 66 | 0.65 | 1.41 | 0.003 | 0.047 | 1.000 | 2341 | tags=41%, list=15%, signal=48% |
| 451 | MORI\_EMU\_MYC\_LYMPHOMA\_BY\_ONSET\_TIME\_DN |  | 16 | 0.75 | 1.41 | 0.012 | 0.047 | 1.000 | 1985 | tags=44%, list=13%, signal=50% |
| 452 | SENESE\_HDAC1\_TARGETS\_UP |  | 386 | 0.63 | 1.41 | 0.000 | 0.049 | 1.000 | 3312 | tags=43%, list=21%, signal=53% |
| 453 | REACTOME\_CREB\_PHOSPHORYLATION\_THROUGH\_THE\_ACTIVATION\_OF\_CAMKII |  | 15 | 0.76 | 1.41 | 0.022 | 0.049 | 1.000 | 3256 | tags=40%, list=21%, signal=50% |
| 454 | PID\_FOXM1PATHWAY |  | 39 | 0.68 | 1.41 | 0.003 | 0.049 | 1.000 | 3439 | tags=59%, list=22%, signal=75% |
| 455 | SENGUPTA\_NASOPHARYNGEAL\_CARCINOMA\_WITH\_LMP1\_UP |  | 325 | 0.63 | 1.41 | 0.000 | 0.049 | 1.000 | 3321 | tags=39%, list=21%, signal=49% |
| 456 | PECE\_MAMMARY\_STEM\_CELL\_UP |  | 115 | 0.64 | 1.41 | 0.000 | 0.050 | 1.000 | 3069 | tags=44%, list=19%, signal=55% |
| 457 | HESS\_TARGETS\_OF\_HOXA9\_AND\_MEIS1\_UP |  | 58 | 0.66 | 1.41 | 0.003 | 0.049 | 1.000 | 3105 | tags=59%, list=20%, signal=73% |
| 458 | MARKEY\_RB1\_CHRONIC\_LOF\_UP |  | 110 | 0.64 | 1.41 | 0.000 | 0.049 | 1.000 | 3196 | tags=55%, list=20%, signal=68% |
| 459 | CHICAS\_RB1\_TARGETS\_LOW\_SERUM |  | 71 | 0.65 | 1.41 | 0.001 | 0.050 | 1.000 | 3268 | tags=49%, list=21%, signal=62% |
| 460 | LANG\_MYB\_FAMILY\_TARGETS |  | 28 | 0.69 | 1.41 | 0.012 | 0.051 | 1.000 | 3711 | tags=61%, list=24%, signal=79% |
| 461 | REACTOME\_MITOTIC\_G2\_G2\_M\_PHASES |  | 71 | 0.65 | 1.41 | 0.002 | 0.051 | 1.000 | 4217 | tags=70%, list=27%, signal=96% |
| 462 | KIM\_WT1\_TARGETS\_DN |  | 398 | 0.63 | 1.41 | 0.000 | 0.051 | 1.000 | 3481 | tags=49%, list=22%, signal=62% |
| 463 | RIZ\_ERYTHROID\_DIFFERENTIATION\_CCNE1 |  | 38 | 0.67 | 1.41 | 0.014 | 0.052 | 1.000 | 3009 | tags=47%, list=19%, signal=58% |
| 464 | BERENJENO\_ROCK\_SIGNALING\_NOT\_VIA\_RHOA\_DN |  | 43 | 0.68 | 1.41 | 0.009 | 0.052 | 1.000 | 1985 | tags=44%, list=13%, signal=50% |
| 465 | WAMUNYOKOLI\_OVARIAN\_CANCER\_LMP\_DN |  | 166 | 0.63 | 1.41 | 0.000 | 0.053 | 1.000 | 2987 | tags=39%, list=19%, signal=48% |
| 466 | REACTOME\_ACTIVATION\_OF\_GENES\_BY\_ATF4 |  | 23 | 0.71 | 1.41 | 0.019 | 0.053 | 1.000 | 2040 | tags=52%, list=13%, signal=60% |
| 467 | BENPORATH\_ES\_2 |  | 29 | 0.69 | 1.41 | 0.009 | 0.052 | 1.000 | 3621 | tags=38%, list=23%, signal=49% |
| 468 | WHITFIELD\_CELL\_CYCLE\_S |  | 123 | 0.64 | 1.41 | 0.000 | 0.053 | 1.000 | 4694 | tags=62%, list=30%, signal=87% |
| 469 | REACTOME\_INTERACTION\_BETWEEN\_L1\_AND\_ANKYRINS |  | 20 | 0.73 | 1.41 | 0.013 | 0.053 | 1.000 | 1306 | tags=10%, list=8%, signal=11% |
| 470 | BLALOCK\_ALZHEIMERS\_DISEASE\_INCIPIENT\_DN |  | 156 | 0.64 | 1.40 | 0.000 | 0.054 | 1.000 | 3664 | tags=53%, list=23%, signal=69% |
| 471 | REACTOME\_PROTEIN\_FOLDING |  | 47 | 0.66 | 1.40 | 0.003 | 0.054 | 1.000 | 3198 | tags=55%, list=20%, signal=69% |
| 472 | TOOKER\_GEMCITABINE\_RESISTANCE\_DN |  | 114 | 0.64 | 1.40 | 0.000 | 0.054 | 1.000 | 2434 | tags=44%, list=15%, signal=52% |
| 473 | WHITFIELD\_CELL\_CYCLE\_G1\_S |  | 114 | 0.63 | 1.40 | 0.000 | 0.054 | 1.000 | 4223 | tags=60%, list=27%, signal=81% |
| 474 | KEGG\_OLFACTORY\_TRANSDUCTION |  | 293 | 0.63 | 1.40 | 0.000 | 0.054 | 1.000 | 566 | tags=1%, list=4%, signal=1% |
| 475 | WANG\_RESPONSE\_TO\_GSK3\_INHIBITOR\_SB216763\_DN |  | 312 | 0.63 | 1.40 | 0.000 | 0.055 | 1.000 | 3310 | tags=50%, list=21%, signal=62% |
| 476 | HAHTOLA\_SEZARY\_SYNDROM\_UP |  | 83 | 0.64 | 1.40 | 0.000 | 0.055 | 1.000 | 2400 | tags=37%, list=15%, signal=44% |
| 477 | REACTOME\_CLASS\_I\_MHC\_MEDIATED\_ANTIGEN\_PROCESSING\_PRESENTATION |  | 217 | 0.63 | 1.40 | 0.000 | 0.055 | 1.000 | 3052 | tags=44%, list=19%, signal=54% |
| 478 | HOSHIDA\_LIVER\_CANCER\_SUBCLASS\_S2 |  | 109 | 0.64 | 1.40 | 0.000 | 0.056 | 1.000 | 3156 | tags=48%, list=20%, signal=59% |
| 479 | WANG\_TUMOR\_INVASIVENESS\_DN |  | 190 | 0.63 | 1.40 | 0.000 | 0.056 | 1.000 | 3231 | tags=53%, list=21%, signal=66% |
| 480 | PARK\_HSC\_AND\_MULTIPOTENT\_PROGENITORS |  | 42 | 0.67 | 1.40 | 0.006 | 0.056 | 1.000 | 3223 | tags=52%, list=20%, signal=66% |
| 481 | LIU\_COMMON\_CANCER\_GENES |  | 57 | 0.65 | 1.40 | 0.002 | 0.056 | 1.000 | 3041 | tags=49%, list=19%, signal=61% |
| 482 | TAKAO\_RESPONSE\_TO\_UVB\_RADIATION\_DN |  | 91 | 0.64 | 1.40 | 0.001 | 0.057 | 1.000 | 1921 | tags=37%, list=12%, signal=42% |
| 483 | REACTOME\_UNFOLDED\_PROTEIN\_RESPONSE |  | 73 | 0.64 | 1.40 | 0.003 | 0.057 | 1.000 | 3664 | tags=53%, list=23%, signal=69% |
| 484 | HOLLEMAN\_ASPARAGINASE\_RESISTANCE\_ALL\_UP |  | 18 | 0.73 | 1.40 | 0.024 | 0.057 | 1.000 | 1091 | tags=33%, list=7%, signal=36% |
| 485 | FALVELLA\_SMOKERS\_WITH\_LUNG\_CANCER |  | 68 | 0.65 | 1.40 | 0.000 | 0.057 | 1.000 | 2945 | tags=38%, list=19%, signal=47% |
| 486 | BIOCARTA\_G1\_PATHWAY |  | 27 | 0.69 | 1.40 | 0.012 | 0.057 | 1.000 | 4113 | tags=70%, list=26%, signal=95% |
| 487 | ZHENG\_GLIOBLASTOMA\_PLASTICITY\_UP |  | 231 | 0.63 | 1.40 | 0.000 | 0.057 | 1.000 | 2965 | tags=43%, list=19%, signal=53% |
| 488 | REACTOME\_ABORTIVE\_ELONGATION\_OF\_HIV1\_TRANSCRIPT\_IN\_THE\_ABSENCE\_OF\_TAT |  | 23 | 0.70 | 1.40 | 0.019 | 0.057 | 1.000 | 3841 | tags=78%, list=24%, signal=103% |
| 489 | SCIBETTA\_KDM5B\_TARGETS\_DN |  | 67 | 0.64 | 1.40 | 0.005 | 0.058 | 1.000 | 2844 | tags=51%, list=18%, signal=62% |
| 490 | TSENG\_IRS1\_TARGETS\_UP |  | 106 | 0.63 | 1.39 | 0.001 | 0.061 | 1.000 | 1534 | tags=33%, list=10%, signal=36% |
| 491 | GENTILE\_UV\_RESPONSE\_CLUSTER\_D7 |  | 38 | 0.67 | 1.39 | 0.006 | 0.061 | 1.000 | 1884 | tags=42%, list=12%, signal=48% |
| 492 | BIOCARTA\_CELLCYCLE\_PATHWAY |  | 22 | 0.70 | 1.39 | 0.022 | 0.062 | 1.000 | 3860 | tags=73%, list=25%, signal=96% |
| 493 | JOHANSSON\_GLIOMAGENESIS\_BY\_PDGFB\_UP |  | 50 | 0.65 | 1.39 | 0.005 | 0.062 | 1.000 | 1281 | tags=42%, list=8%, signal=46% |
| 494 | KEGG\_ALANINE\_ASPARTATE\_AND\_GLUTAMATE\_METABOLISM |  | 31 | 0.68 | 1.39 | 0.013 | 0.061 | 1.000 | 1463 | tags=32%, list=9%, signal=35% |
| 495 | MATTHEWS\_AP1\_TARGETS |  | 17 | 0.72 | 1.39 | 0.027 | 0.062 | 1.000 | 2088 | tags=53%, list=13%, signal=61% |
| 496 | REACTOME\_PKB\_MEDIATED\_EVENTS |  | 23 | 0.70 | 1.39 | 0.028 | 0.063 | 1.000 | 1776 | tags=48%, list=11%, signal=54% |
| 497 | SMID\_BREAST\_CANCER\_RELAPSE\_IN\_LUNG\_UP |  | 21 | 0.70 | 1.39 | 0.018 | 0.063 | 1.000 | 935 | tags=24%, list=6%, signal=25% |
| 498 | MARIADASON\_RESPONSE\_TO\_BUTYRATE\_SULINDAC\_4 |  | 17 | 0.72 | 1.39 | 0.023 | 0.062 | 1.000 | 3737 | tags=59%, list=24%, signal=77% |
| 499 | REACTOME\_MICRORNA\_MIRNA\_BIOGENESIS |  | 21 | 0.70 | 1.39 | 0.024 | 0.063 | 1.000 | 3802 | tags=71%, list=24%, signal=94% |
| 500 | REACTOME\_RECRUITMENT\_OF\_MITOTIC\_CENTROSOME\_PROTEINS\_AND\_COMPLEXES |  | 56 | 0.65 | 1.39 | 0.003 | 0.063 | 1.000 | 5077 | tags=86%, list=32%, signal=126% |
| 501 | BURTON\_ADIPOGENESIS\_11 |  | 52 | 0.65 | 1.39 | 0.006 | 0.064 | 1.000 | 3953 | tags=67%, list=25%, signal=90% |
| 502 | STEARMAN\_LUNG\_CANCER\_EARLY\_VS\_LATE\_UP |  | 112 | 0.63 | 1.39 | 0.000 | 0.064 | 1.000 | 3607 | tags=50%, list=23%, signal=64% |
| 503 | MONNIER\_POSTRADIATION\_TUMOR\_ESCAPE\_UP |  | 334 | 0.62 | 1.39 | 0.000 | 0.064 | 1.000 | 3412 | tags=50%, list=22%, signal=62% |
| 504 | NADERI\_BREAST\_CANCER\_PROGNOSIS\_UP |  | 45 | 0.66 | 1.39 | 0.004 | 0.064 | 1.000 | 2302 | tags=44%, list=15%, signal=52% |
| 505 | BENPORATH\_ES\_CORE\_NINE\_CORRELATED |  | 95 | 0.63 | 1.39 | 0.001 | 0.065 | 1.000 | 3659 | tags=49%, list=23%, signal=64% |
| 506 | SEIDEN\_MET\_SIGNALING |  | 18 | 0.72 | 1.39 | 0.021 | 0.065 | 1.000 | 2002 | tags=72%, list=13%, signal=83% |
| 507 | REACTOME\_RNA\_POL\_III\_TRANSCRIPTION\_TERMINATION |  | 19 | 0.71 | 1.39 | 0.022 | 0.065 | 1.000 | 3943 | tags=79%, list=25%, signal=105% |
| 508 | DAZARD\_UV\_RESPONSE\_CLUSTER\_G6 |  | 131 | 0.63 | 1.39 | 0.000 | 0.065 | 1.000 | 3872 | tags=54%, list=25%, signal=71% |
| 509 | REACTOME\_APC\_C\_CDC20\_MEDIATED\_DEGRADATION\_OF\_CYCLIN\_B |  | 17 | 0.71 | 1.39 | 0.036 | 0.065 | 1.000 | 3551 | tags=76%, list=23%, signal=99% |
| 510 | GENTILE\_UV\_HIGH\_DOSE\_DN |  | 284 | 0.62 | 1.39 | 0.000 | 0.065 | 1.000 | 3352 | tags=49%, list=21%, signal=61% |
| 511 | BYSTROEM\_CORRELATED\_WITH\_IL5\_DN |  | 61 | 0.65 | 1.39 | 0.005 | 0.065 | 1.000 | 2318 | tags=38%, list=15%, signal=44% |
| 512 | JOHNSTONE\_PARVB\_TARGETS\_2\_DN |  | 278 | 0.62 | 1.39 | 0.000 | 0.065 | 1.000 | 4248 | tags=62%, list=27%, signal=83% |
| 513 | WANG\_RESPONSE\_TO\_FORSKOLIN\_UP |  | 22 | 0.70 | 1.39 | 0.030 | 0.065 | 1.000 | 2497 | tags=50%, list=16%, signal=59% |
| 514 | HU\_GENOTOXIN\_ACTION\_DIRECT\_VS\_INDIRECT\_24HR |  | 46 | 0.65 | 1.39 | 0.010 | 0.066 | 1.000 | 1629 | tags=37%, list=10%, signal=41% |
| 515 | FLECHNER\_PBL\_KIDNEY\_TRANSPLANT\_OK\_VS\_DONOR\_DN |  | 37 | 0.67 | 1.39 | 0.015 | 0.066 | 1.000 | 2874 | tags=46%, list=18%, signal=56% |
| 516 | ZHU\_CMV\_ALL\_UP |  | 106 | 0.63 | 1.39 | 0.000 | 0.066 | 1.000 | 3542 | tags=54%, list=22%, signal=69% |
| 517 | YAO\_TEMPORAL\_RESPONSE\_TO\_PROGESTERONE\_CLUSTER\_13 |  | 144 | 0.63 | 1.39 | 0.000 | 0.066 | 1.000 | 3544 | tags=58%, list=23%, signal=74% |
| 518 | BURTON\_ADIPOGENESIS\_2 |  | 69 | 0.64 | 1.39 | 0.001 | 0.066 | 1.000 | 3312 | tags=49%, list=21%, signal=62% |
| 519 | PROVENZANI\_METASTASIS\_UP |  | 168 | 0.62 | 1.39 | 0.000 | 0.066 | 1.000 | 2450 | tags=38%, list=16%, signal=45% |
| 520 | MCGOWAN\_RSP6\_TARGETS\_UP |  | 16 | 0.72 | 1.39 | 0.032 | 0.066 | 1.000 | 1852 | tags=31%, list=12%, signal=35% |
| 521 | BLUM\_RESPONSE\_TO\_SALIRASIB\_UP |  | 227 | 0.62 | 1.39 | 0.000 | 0.067 | 1.000 | 3894 | tags=53%, list=25%, signal=70% |
| 522 | GARGALOVIC\_RESPONSE\_TO\_OXIDIZED\_PHOSPHOLIPIDS\_RED\_UP |  | 16 | 0.73 | 1.38 | 0.035 | 0.067 | 1.000 | 2281 | tags=50%, list=14%, signal=58% |
| 523 | HOFMANN\_CELL\_LYMPHOMA\_UP |  | 44 | 0.66 | 1.38 | 0.011 | 0.067 | 1.000 | 2925 | tags=43%, list=19%, signal=53% |
| 524 | PID\_RB\_1PATHWAY |  | 64 | 0.64 | 1.38 | 0.003 | 0.067 | 1.000 | 3673 | tags=50%, list=23%, signal=65% |
| 525 | GERHOLD\_ADIPOGENESIS\_DN |  | 63 | 0.65 | 1.38 | 0.003 | 0.067 | 1.000 | 2202 | tags=48%, list=14%, signal=55% |
| 526 | JAEGER\_METASTASIS\_UP |  | 39 | 0.66 | 1.38 | 0.008 | 0.067 | 1.000 | 2577 | tags=46%, list=16%, signal=55% |
| 527 | REACTOME\_PURINE\_METABOLISM |  | 32 | 0.66 | 1.38 | 0.019 | 0.067 | 1.000 | 2163 | tags=44%, list=14%, signal=51% |
| 528 | SCHAEFFER\_SOX9\_TARGETS\_IN\_PROSTATE\_DEVELOPMENT\_DN |  | 43 | 0.66 | 1.38 | 0.006 | 0.067 | 1.000 | 3067 | tags=47%, list=19%, signal=58% |
| 529 | REACTOME\_DOUBLE\_STRAND\_BREAK\_REPAIR |  | 22 | 0.70 | 1.38 | 0.020 | 0.067 | 1.000 | 3196 | tags=68%, list=20%, signal=85% |
| 530 | SHAFFER\_IRF4\_TARGETS\_IN\_PLASMA\_CELL\_VS\_MATURE\_B\_LYMPHOCYTE |  | 63 | 0.64 | 1.38 | 0.004 | 0.067 | 1.000 | 2839 | tags=43%, list=18%, signal=52% |
| 531 | PEPPER\_CHRONIC\_LYMPHOCYTIC\_LEUKEMIA\_DN |  | 18 | 0.72 | 1.38 | 0.028 | 0.067 | 1.000 | 1574 | tags=22%, list=10%, signal=25% |
| 532 | STARK\_PREFRONTAL\_CORTEX\_22Q11\_DELETION\_DN |  | 422 | 0.61 | 1.38 | 0.000 | 0.067 | 1.000 | 3270 | tags=45%, list=21%, signal=55% |
| 533 | BIOCARTA\_NDKDYNAMIN\_PATHWAY |  | 18 | 0.71 | 1.38 | 0.042 | 0.068 | 1.000 | 2455 | tags=61%, list=16%, signal=72% |
| 534 | REACTOME\_TRANSCRIPTIONAL\_ACTIVITY\_OF\_SMAD2\_SMAD3\_SMAD4\_HETEROTRIMER |  | 36 | 0.67 | 1.38 | 0.010 | 0.068 | 1.000 | 3865 | tags=67%, list=25%, signal=88% |
| 535 | SLEBOS\_HEAD\_AND\_NECK\_CANCER\_WITH\_HPV\_UP |  | 69 | 0.64 | 1.38 | 0.003 | 0.068 | 1.000 | 4511 | tags=57%, list=29%, signal=79% |
| 536 | AMIT\_EGF\_RESPONSE\_60\_MCF10A |  | 35 | 0.66 | 1.38 | 0.009 | 0.068 | 1.000 | 1863 | tags=29%, list=12%, signal=32% |
| 537 | MELLMAN\_TUT1\_TARGETS\_UP |  | 18 | 0.72 | 1.38 | 0.029 | 0.068 | 1.000 | 2787 | tags=61%, list=18%, signal=74% |
| 538 | MARTORIATI\_MDM4\_TARGETS\_FETAL\_LIVER\_DN |  | 461 | 0.61 | 1.38 | 0.000 | 0.068 | 1.000 | 3883 | tags=51%, list=25%, signal=65% |
| 539 | REACTOME\_LOSS\_OF\_NLP\_FROM\_MITOTIC\_CENTROSOMES |  | 49 | 0.65 | 1.38 | 0.008 | 0.068 | 1.000 | 5077 | tags=86%, list=32%, signal=126% |
| 540 | TOYOTA\_TARGETS\_OF\_MIR34B\_AND\_MIR34C |  | 348 | 0.62 | 1.38 | 0.000 | 0.068 | 1.000 | 3957 | tags=55%, list=25%, signal=71% |
| 541 | REACTOME\_TRANSCRIPTION |  | 165 | 0.62 | 1.38 | 0.000 | 0.069 | 1.000 | 3996 | tags=58%, list=25%, signal=77% |
| 542 | SESTO\_RESPONSE\_TO\_UV\_C1 |  | 65 | 0.65 | 1.38 | 0.005 | 0.069 | 1.000 | 2542 | tags=48%, list=16%, signal=57% |
| 543 | BURTON\_ADIPOGENESIS\_7 |  | 46 | 0.65 | 1.38 | 0.005 | 0.069 | 1.000 | 1801 | tags=39%, list=11%, signal=44% |
| 544 | BOYLAN\_MULTIPLE\_MYELOMA\_C\_D\_UP |  | 122 | 0.62 | 1.38 | 0.001 | 0.069 | 1.000 | 4227 | tags=50%, list=27%, signal=68% |
| 545 | REACTOME\_MRNA\_CAPPING |  | 27 | 0.68 | 1.38 | 0.015 | 0.069 | 1.000 | 3802 | tags=78%, list=24%, signal=102% |
| 546 | PID\_P53REGULATIONPATHWAY |  | 55 | 0.64 | 1.38 | 0.002 | 0.070 | 1.000 | 4395 | tags=69%, list=28%, signal=95% |
| 547 | REACTOME\_CONVERSION\_FROM\_APC\_C\_CDC20\_TO\_APC\_C\_CDH1\_IN\_LATE\_ANAPHASE |  | 15 | 0.74 | 1.38 | 0.040 | 0.070 | 1.000 | 1471 | tags=53%, list=9%, signal=59% |
| 548 | LOPEZ\_TRANSLATION\_VIA\_FN1\_SIGNALING |  | 34 | 0.66 | 1.38 | 0.017 | 0.070 | 1.000 | 3314 | tags=47%, list=21%, signal=59% |
| 549 | AMIT\_EGF\_RESPONSE\_60\_HELA |  | 40 | 0.66 | 1.38 | 0.012 | 0.070 | 1.000 | 2667 | tags=40%, list=17%, signal=48% |
| 550 | HOEBEKE\_LYMPHOID\_STEM\_CELL\_UP |  | 78 | 0.63 | 1.38 | 0.004 | 0.070 | 1.000 | 3197 | tags=42%, list=20%, signal=53% |
| 551 | NIKOLSKY\_BREAST\_CANCER\_12Q13\_Q21\_AMPLICON |  | 38 | 0.66 | 1.38 | 0.012 | 0.070 | 1.000 | 2804 | tags=39%, list=18%, signal=48% |
| 552 | BIOCARTA\_NO1\_PATHWAY |  | 29 | 0.67 | 1.38 | 0.034 | 0.071 | 1.000 | 1173 | tags=28%, list=7%, signal=30% |
| 553 | WENDT\_COHESIN\_TARGETS\_UP |  | 27 | 0.68 | 1.38 | 0.028 | 0.071 | 1.000 | 4039 | tags=78%, list=26%, signal=104% |
| 554 | MATZUK\_CENTRAL\_FOR\_FEMALE\_FERTILITY |  | 29 | 0.67 | 1.38 | 0.029 | 0.071 | 1.000 | 1389 | tags=17%, list=9%, signal=19% |
| 555 | JIANG\_VHL\_TARGETS |  | 108 | 0.63 | 1.38 | 0.000 | 0.071 | 1.000 | 3166 | tags=49%, list=20%, signal=61% |
| 556 | REACTOME\_FORMATION\_OF\_INCISION\_COMPLEX\_IN\_GG\_NER |  | 19 | 0.71 | 1.38 | 0.021 | 0.071 | 1.000 | 2388 | tags=58%, list=15%, signal=68% |
| 557 | PID\_CMYB\_PATHWAY |  | 79 | 0.63 | 1.38 | 0.003 | 0.071 | 1.000 | 2668 | tags=35%, list=17%, signal=42% |
| 558 | REACTOME\_FORMATION\_OF\_TRANSCRIPTION\_COUPLED\_NER\_TC\_NER\_REPAIR\_COMPLEX |  | 27 | 0.67 | 1.38 | 0.029 | 0.072 | 1.000 | 3802 | tags=67%, list=24%, signal=88% |
| 559 | AMIT\_EGF\_RESPONSE\_120\_MCF10A |  | 40 | 0.66 | 1.38 | 0.014 | 0.072 | 1.000 | 4212 | tags=63%, list=27%, signal=85% |
| 560 | PID\_AURORA\_A\_PATHWAY |  | 30 | 0.67 | 1.38 | 0.024 | 0.072 | 1.000 | 4113 | tags=70%, list=26%, signal=95% |
| 561 | BILD\_CTNNB1\_ONCOGENIC\_SIGNATURE |  | 62 | 0.64 | 1.38 | 0.005 | 0.072 | 1.000 | 3677 | tags=47%, list=23%, signal=61% |
| 562 | NOUZOVA\_TRETINOIN\_AND\_H4\_ACETYLATION |  | 108 | 0.62 | 1.38 | 0.001 | 0.072 | 1.000 | 3590 | tags=52%, list=23%, signal=67% |
| 563 | GENTILE\_UV\_RESPONSE\_CLUSTER\_D4 |  | 51 | 0.65 | 1.38 | 0.005 | 0.072 | 1.000 | 3794 | tags=63%, list=24%, signal=82% |
| 564 | REACTOME\_RNA\_POL\_III\_TRANSCRIPTION\_INITIATION\_FROM\_TYPE\_2\_PROMOTER |  | 23 | 0.69 | 1.38 | 0.016 | 0.072 | 1.000 | 4487 | tags=83%, list=28%, signal=115% |
| 565 | KRIGE\_AMINO\_ACID\_DEPRIVATION |  | 28 | 0.68 | 1.38 | 0.015 | 0.072 | 1.000 | 2165 | tags=50%, list=14%, signal=58% |
| 566 | BILANGES\_RAPAMYCIN\_SENSITIVE\_GENES |  | 34 | 0.66 | 1.38 | 0.014 | 0.071 | 1.000 | 2624 | tags=41%, list=17%, signal=49% |
| 567 | REACTOME\_METABOLISM\_OF\_NUCLEOTIDES |  | 67 | 0.64 | 1.38 | 0.004 | 0.072 | 1.000 | 2169 | tags=40%, list=14%, signal=47% |
| 568 | SESTO\_RESPONSE\_TO\_UV\_C5 |  | 46 | 0.65 | 1.37 | 0.012 | 0.073 | 1.000 | 2827 | tags=50%, list=18%, signal=61% |
| 569 | REACTOME\_ASSOCIATION\_OF\_TRIC\_CCT\_WITH\_TARGET\_PROTEINS\_DURING\_BIOSYNTHESIS |  | 25 | 0.69 | 1.37 | 0.030 | 0.073 | 1.000 | 3198 | tags=60%, list=20%, signal=75% |
| 570 | PID\_AP1\_PATHWAY |  | 68 | 0.64 | 1.37 | 0.003 | 0.073 | 1.000 | 3063 | tags=35%, list=19%, signal=44% |
| 571 | DEBIASI\_APOPTOSIS\_BY\_REOVIRUS\_INFECTION\_UP |  | 264 | 0.62 | 1.37 | 0.000 | 0.073 | 1.000 | 3593 | tags=50%, list=23%, signal=64% |
| 572 | REACTOME\_REGULATORY\_RNA\_PATHWAYS |  | 24 | 0.69 | 1.37 | 0.027 | 0.073 | 1.000 | 3802 | tags=67%, list=24%, signal=88% |
| 573 | BIOCARTA\_NOS1\_PATHWAY |  | 19 | 0.71 | 1.37 | 0.029 | 0.074 | 1.000 | 2582 | tags=37%, list=16%, signal=44% |
| 574 | CARD\_MIR302A\_TARGETS |  | 72 | 0.64 | 1.37 | 0.002 | 0.074 | 1.000 | 3079 | tags=42%, list=20%, signal=52% |
| 575 | VANTVEER\_BREAST\_CANCER\_BRCA1\_UP |  | 32 | 0.67 | 1.37 | 0.018 | 0.073 | 1.000 | 4149 | tags=66%, list=26%, signal=89% |
| 576 | REACTOME\_FORMATION\_OF\_THE\_HIV1\_EARLY\_ELONGATION\_COMPLEX |  | 31 | 0.67 | 1.37 | 0.028 | 0.074 | 1.000 | 3841 | tags=71%, list=24%, signal=94% |
| 577 | PUIFFE\_INVASION\_INHIBITED\_BY\_ASCITES\_DN |  | 118 | 0.62 | 1.37 | 0.000 | 0.074 | 1.000 | 3793 | tags=53%, list=24%, signal=69% |
| 578 | KOINUMA\_COLON\_CANCER\_MSI\_DN |  | 15 | 0.73 | 1.37 | 0.038 | 0.074 | 1.000 | 2223 | tags=60%, list=14%, signal=70% |
| 579 | GENTILE\_RESPONSE\_CLUSTER\_D3 |  | 58 | 0.64 | 1.37 | 0.004 | 0.075 | 1.000 | 2384 | tags=47%, list=15%, signal=55% |
| 580 | REACTOME\_UNBLOCKING\_OF\_NMDA\_RECEPTOR\_GLUTAMATE\_BINDING\_AND\_ACTIVATION |  | 15 | 0.74 | 1.37 | 0.022 | 0.075 | 1.000 | 3256 | tags=20%, list=21%, signal=25% |
| 581 | ZHU\_CMV\_24\_HR\_UP |  | 82 | 0.63 | 1.37 | 0.001 | 0.075 | 1.000 | 2622 | tags=46%, list=17%, signal=55% |
| 582 | GOLDRATH\_HOMEOSTATIC\_PROLIFERATION |  | 150 | 0.62 | 1.37 | 0.000 | 0.076 | 1.000 | 3828 | tags=59%, list=24%, signal=78% |
| 583 | REACTOME\_TELOMERE\_MAINTENANCE |  | 54 | 0.64 | 1.37 | 0.010 | 0.076 | 1.000 | 2363 | tags=41%, list=15%, signal=48% |
| 584 | TERAO\_AOX4\_TARGETS\_SKIN\_UP |  | 36 | 0.66 | 1.37 | 0.020 | 0.076 | 1.000 | 4239 | tags=64%, list=27%, signal=87% |
| 585 | PACHER\_TARGETS\_OF\_IGF1\_AND\_IGF2\_UP |  | 33 | 0.66 | 1.37 | 0.028 | 0.077 | 1.000 | 2436 | tags=42%, list=15%, signal=50% |
| 586 | JIANG\_AGING\_CEREBRAL\_CORTEX\_DN |  | 48 | 0.64 | 1.37 | 0.008 | 0.077 | 1.000 | 2807 | tags=42%, list=18%, signal=51% |
| 587 | WENG\_POR\_TARGETS\_GLOBAL\_DN |  | 22 | 0.69 | 1.37 | 0.038 | 0.077 | 1.000 | 2036 | tags=32%, list=13%, signal=36% |
| 588 | BARRIER\_COLON\_CANCER\_RECURRENCE\_UP |  | 39 | 0.65 | 1.37 | 0.016 | 0.077 | 1.000 | 4350 | tags=64%, list=28%, signal=88% |
| 589 | REACTOME\_DESTABILIZATION\_OF\_MRNA\_BY\_TRISTETRAPROLIN\_TTP |  | 17 | 0.72 | 1.37 | 0.038 | 0.078 | 1.000 | 2040 | tags=53%, list=13%, signal=61% |
| 590 | LASTOWSKA\_COAMPLIFIED\_WITH\_MYCN |  | 36 | 0.66 | 1.37 | 0.021 | 0.078 | 1.000 | 1084 | tags=22%, list=7%, signal=24% |
| 591 | BERENJENO\_TRANSFORMED\_BY\_RHOA\_FOREVER\_DN |  | 29 | 0.68 | 1.37 | 0.017 | 0.079 | 1.000 | 1699 | tags=48%, list=11%, signal=54% |
| 592 | BROWN\_MYELOID\_CELL\_DEVELOPMENT\_DN |  | 118 | 0.62 | 1.37 | 0.000 | 0.080 | 1.000 | 2688 | tags=42%, list=17%, signal=51% |
| 593 | POMEROY\_MEDULLOBLASTOMA\_DESMOPLASIC\_VS\_CLASSIC\_DN |  | 53 | 0.64 | 1.36 | 0.009 | 0.080 | 1.000 | 2796 | tags=38%, list=18%, signal=46% |
| 594 | POTTI\_CYTOXAN\_SENSITIVITY |  | 32 | 0.66 | 1.36 | 0.022 | 0.081 | 1.000 | 1032 | tags=28%, list=7%, signal=30% |
| 595 | TIEN\_INTESTINE\_PROBIOTICS\_2HR\_DN |  | 78 | 0.64 | 1.36 | 0.000 | 0.081 | 1.000 | 3708 | tags=58%, list=24%, signal=75% |
| 596 | TIEN\_INTESTINE\_PROBIOTICS\_6HR\_DN |  | 147 | 0.62 | 1.36 | 0.000 | 0.083 | 1.000 | 3364 | tags=54%, list=21%, signal=68% |
| 597 | YAMAZAKI\_TCEB3\_TARGETS\_DN |  | 191 | 0.61 | 1.36 | 0.000 | 0.084 | 1.000 | 2246 | tags=38%, list=14%, signal=43% |
| 598 | DING\_LUNG\_CANCER\_EXPRESSION\_BY\_COPY\_NUMBER |  | 91 | 0.62 | 1.36 | 0.003 | 0.085 | 1.000 | 4559 | tags=74%, list=29%, signal=103% |
| 599 | YIH\_RESPONSE\_TO\_ARSENITE\_C1 |  | 24 | 0.68 | 1.36 | 0.026 | 0.085 | 1.000 | 3239 | tags=58%, list=21%, signal=73% |
| 600 | FLECHNER\_PBL\_KIDNEY\_TRANSPLANT\_REJECTED\_VS\_OK\_DN |  | 48 | 0.65 | 1.36 | 0.014 | 0.085 | 1.000 | 2118 | tags=40%, list=13%, signal=46% |
| 601 | GINESTIER\_BREAST\_CANCER\_ZNF217\_AMPLIFIED\_UP |  | 66 | 0.63 | 1.36 | 0.002 | 0.085 | 1.000 | 3916 | tags=58%, list=25%, signal=76% |
| 602 | BAE\_BRCA1\_TARGETS\_DN |  | 29 | 0.67 | 1.36 | 0.021 | 0.086 | 1.000 | 2977 | tags=55%, list=19%, signal=68% |
| 603 | BIOCARTA\_P53\_PATHWAY |  | 16 | 0.71 | 1.36 | 0.043 | 0.087 | 1.000 | 2925 | tags=69%, list=19%, signal=84% |
| 604 | WANG\_LMO4\_TARGETS\_DN |  | 294 | 0.61 | 1.36 | 0.000 | 0.087 | 1.000 | 3988 | tags=55%, list=25%, signal=72% |
| 605 | PYEON\_CANCER\_HEAD\_AND\_NECK\_VS\_CERVICAL\_UP |  | 151 | 0.61 | 1.36 | 0.000 | 0.088 | 1.000 | 4028 | tags=55%, list=26%, signal=73% |
| 606 | KEGG\_UBIQUITIN\_MEDIATED\_PROTEOLYSIS |  | 121 | 0.61 | 1.36 | 0.001 | 0.089 | 1.000 | 4552 | tags=62%, list=29%, signal=87% |
| 607 | RADAEVA\_RESPONSE\_TO\_IFNA1\_UP |  | 47 | 0.64 | 1.36 | 0.014 | 0.089 | 1.000 | 3036 | tags=40%, list=19%, signal=50% |
| 608 | CHIBA\_RESPONSE\_TO\_TSA\_DN |  | 21 | 0.68 | 1.36 | 0.038 | 0.089 | 1.000 | 3196 | tags=52%, list=20%, signal=66% |
| 609 | BIOCARTA\_FAS\_PATHWAY |  | 29 | 0.66 | 1.36 | 0.030 | 0.090 | 1.000 | 3630 | tags=48%, list=23%, signal=63% |
| 610 | SMITH\_TERT\_TARGETS\_UP |  | 134 | 0.62 | 1.36 | 0.000 | 0.090 | 1.000 | 3253 | tags=51%, list=21%, signal=63% |
| 611 | GRADE\_COLON\_VS\_RECTAL\_CANCER\_UP |  | 33 | 0.65 | 1.36 | 0.031 | 0.090 | 1.000 | 2922 | tags=45%, list=19%, signal=56% |
| 612 | CEBALLOS\_TARGETS\_OF\_TP53\_AND\_MYC\_UP |  | 19 | 0.69 | 1.35 | 0.048 | 0.091 | 1.000 | 1265 | tags=32%, list=8%, signal=34% |
| 613 | CREIGHTON\_ENDOCRINE\_THERAPY\_RESISTANCE\_1 |  | 432 | 0.60 | 1.35 | 0.000 | 0.091 | 1.000 | 4066 | tags=47%, list=26%, signal=62% |
| 614 | AZARE\_NEOPLASTIC\_TRANSFORMATION\_BY\_STAT3\_DN |  | 109 | 0.62 | 1.35 | 0.001 | 0.092 | 1.000 | 3304 | tags=41%, list=21%, signal=52% |
| 615 | CHOI\_ATL\_CHRONIC\_VS\_ACUTE\_DN |  | 17 | 0.71 | 1.35 | 0.040 | 0.091 | 1.000 | 1913 | tags=53%, list=12%, signal=60% |
| 616 | BONCI\_TARGETS\_OF\_MIR15A\_AND\_MIR16\_1 |  | 85 | 0.62 | 1.35 | 0.002 | 0.091 | 1.000 | 3210 | tags=35%, list=20%, signal=44% |
| 617 | BIOCARTA\_ATRBRCA\_PATHWAY |  | 21 | 0.69 | 1.35 | 0.049 | 0.093 | 1.000 | 4114 | tags=76%, list=26%, signal=103% |
| 618 | KEGG\_HOMOLOGOUS\_RECOMBINATION |  | 25 | 0.67 | 1.35 | 0.047 | 0.095 | 1.000 | 3544 | tags=60%, list=23%, signal=77% |
| 619 | PID\_NFAT\_3PATHWAY |  | 50 | 0.64 | 1.35 | 0.011 | 0.095 | 1.000 | 2737 | tags=38%, list=17%, signal=46% |
| 620 | PID\_FANCONI\_PATHWAY |  | 43 | 0.64 | 1.35 | 0.015 | 0.097 | 1.000 | 4643 | tags=77%, list=29%, signal=109% |
| 621 | VETTER\_TARGETS\_OF\_PRKCA\_AND\_ETS1\_UP |  | 15 | 0.71 | 1.35 | 0.052 | 0.097 | 1.000 | 3146 | tags=53%, list=20%, signal=67% |
| 622 | REACTOME\_L1CAM\_INTERACTIONS |  | 81 | 0.62 | 1.35 | 0.003 | 0.097 | 1.000 | 2842 | tags=28%, list=18%, signal=34% |
| 623 | MARIADASON\_RESPONSE\_TO\_BUTYRATE\_SULINDAC\_6 |  | 42 | 0.64 | 1.35 | 0.030 | 0.098 | 1.000 | 3079 | tags=40%, list=20%, signal=50% |
| 624 | GENTILE\_UV\_RESPONSE\_CLUSTER\_D5 |  | 34 | 0.65 | 1.35 | 0.026 | 0.099 | 1.000 | 3418 | tags=50%, list=22%, signal=64% |
| 625 | EHLERS\_ANEUPLOIDY\_UP |  | 34 | 0.65 | 1.35 | 0.022 | 0.100 | 1.000 | 3599 | tags=47%, list=23%, signal=61% |
| 626 | REACTOME\_RNA\_POL\_III\_CHAIN\_ELONGATION |  | 17 | 0.69 | 1.35 | 0.063 | 0.100 | 1.000 | 4487 | tags=82%, list=28%, signal=115% |
| 627 | REACTOME\_MITOCHONDRIAL\_PROTEIN\_IMPORT |  | 46 | 0.63 | 1.35 | 0.012 | 0.100 | 1.000 | 2653 | tags=46%, list=17%, signal=55% |
| 628 | LIU\_BREAST\_CANCER |  | 20 | 0.69 | 1.35 | 0.039 | 0.100 | 1.000 | 2573 | tags=40%, list=16%, signal=48% |
| 629 | WANG\_CLIM2\_TARGETS\_DN |  | 150 | 0.61 | 1.35 | 0.000 | 0.100 | 1.000 | 4027 | tags=53%, list=26%, signal=70% |
| 630 | ZHONG\_RESPONSE\_TO\_AZACITIDINE\_AND\_TSA\_DN |  | 54 | 0.64 | 1.35 | 0.008 | 0.101 | 1.000 | 4144 | tags=65%, list=26%, signal=88% |
| 631 | CHOW\_RASSF1\_TARGETS\_UP |  | 26 | 0.66 | 1.35 | 0.025 | 0.101 | 1.000 | 2921 | tags=54%, list=19%, signal=66% |
| 632 | REACTOME\_APOPTOTIC\_EXECUTION\_PHASE |  | 50 | 0.63 | 1.34 | 0.009 | 0.101 | 1.000 | 2297 | tags=38%, list=15%, signal=44% |
| 633 | APPIERTO\_RESPONSE\_TO\_FENRETINIDE\_UP |  | 36 | 0.64 | 1.34 | 0.032 | 0.102 | 1.000 | 2692 | tags=47%, list=17%, signal=57% |
| 634 | PID\_HIF1APATHWAY |  | 18 | 0.70 | 1.34 | 0.041 | 0.102 | 1.000 | 2044 | tags=39%, list=13%, signal=45% |
| 635 | LAU\_APOPTOSIS\_CDKN2A\_UP |  | 53 | 0.63 | 1.34 | 0.019 | 0.102 | 1.000 | 3296 | tags=51%, list=21%, signal=64% |
| 636 | YAO\_TEMPORAL\_RESPONSE\_TO\_PROGESTERONE\_CLUSTER\_17 |  | 164 | 0.60 | 1.34 | 0.001 | 0.102 | 1.000 | 4145 | tags=58%, list=26%, signal=78% |
| 637 | MARSHALL\_VIRAL\_INFECTION\_RESPONSE\_DN |  | 26 | 0.66 | 1.34 | 0.031 | 0.103 | 1.000 | 2001 | tags=31%, list=13%, signal=35% |
| 638 | CHICAS\_RB1\_TARGETS\_SENESCENT |  | 485 | 0.60 | 1.34 | 0.000 | 0.103 | 1.000 | 3712 | tags=47%, list=24%, signal=59% |
| 639 | PARK\_HSC\_MARKERS |  | 39 | 0.64 | 1.34 | 0.025 | 0.104 | 1.000 | 2822 | tags=46%, list=18%, signal=56% |
| 640 | HOLLEMAN\_PREDNISOLONE\_RESISTANCE\_B\_ALL\_UP |  | 19 | 0.69 | 1.34 | 0.058 | 0.104 | 1.000 | 3114 | tags=58%, list=20%, signal=72% |
| 641 | CHEOK\_RESPONSE\_TO\_HD\_MTX\_UP |  | 17 | 0.69 | 1.34 | 0.059 | 0.106 | 1.000 | 2026 | tags=35%, list=13%, signal=40% |
| 642 | REACTOME\_SYNTHESIS\_AND\_INTERCONVERSION\_OF\_NUCLEOTIDE\_DI\_AND\_TRIPHOSPHATES |  | 16 | 0.71 | 1.34 | 0.046 | 0.106 | 1.000 | 2483 | tags=56%, list=16%, signal=67% |
| 643 | REACTOME\_RAS\_ACTIVATION\_UOPN\_CA2\_INFUX\_THROUGH\_NMDA\_RECEPTOR |  | 17 | 0.70 | 1.34 | 0.047 | 0.106 | 1.000 | 3256 | tags=29%, list=21%, signal=37% |
| 644 | PID\_TGFBRPATHWAY |  | 52 | 0.63 | 1.34 | 0.014 | 0.106 | 1.000 | 4025 | tags=60%, list=26%, signal=80% |
| 645 | VECCHI\_GASTRIC\_CANCER\_ADVANCED\_VS\_EARLY\_UP |  | 154 | 0.60 | 1.34 | 0.000 | 0.108 | 1.000 | 1908 | tags=21%, list=12%, signal=24% |
| 646 | GABRIELY\_MIR21\_TARGETS |  | 258 | 0.60 | 1.34 | 0.000 | 0.109 | 1.000 | 4709 | tags=55%, list=30%, signal=77% |
| 647 | LIU\_VAV3\_PROSTATE\_CARCINOGENESIS\_DN |  | 16 | 0.71 | 1.34 | 0.051 | 0.109 | 1.000 | 2667 | tags=31%, list=17%, signal=38% |
| 648 | GENTILE\_UV\_RESPONSE\_CLUSTER\_D9 |  | 21 | 0.67 | 1.34 | 0.066 | 0.110 | 1.000 | 2001 | tags=48%, list=13%, signal=54% |
| 649 | JACKSON\_DNMT1\_TARGETS\_UP |  | 73 | 0.61 | 1.34 | 0.004 | 0.110 | 1.000 | 3760 | tags=51%, list=24%, signal=66% |
| 650 | REACTOME\_DIABETES\_PATHWAYS |  | 123 | 0.60 | 1.34 | 0.000 | 0.110 | 1.000 | 3731 | tags=46%, list=24%, signal=59% |
| 651 | SENESE\_HDAC3\_TARGETS\_UP |  | 428 | 0.59 | 1.34 | 0.000 | 0.110 | 1.000 | 3310 | tags=39%, list=21%, signal=47% |
| 652 | PETROVA\_PROX1\_TARGETS\_UP |  | 26 | 0.67 | 1.34 | 0.039 | 0.110 | 1.000 | 2999 | tags=46%, list=19%, signal=57% |
| 653 | GALE\_APL\_WITH\_FLT3\_MUTATED\_UP |  | 45 | 0.63 | 1.34 | 0.017 | 0.112 | 1.000 | 3197 | tags=49%, list=20%, signal=61% |
| 654 | ALONSO\_METASTASIS\_UP |  | 168 | 0.60 | 1.34 | 0.000 | 0.112 | 1.000 | 3156 | tags=44%, list=20%, signal=54% |
| 655 | KIM\_PTEN\_TARGETS\_UP |  | 16 | 0.69 | 1.34 | 0.067 | 0.112 | 1.000 | 3408 | tags=56%, list=22%, signal=72% |
| 656 | STEIN\_ESR1\_TARGETS |  | 80 | 0.61 | 1.34 | 0.003 | 0.112 | 1.000 | 3705 | tags=45%, list=24%, signal=59% |
| 657 | MCBRYAN\_PUBERTAL\_BREAST\_5\_6WK\_DN |  | 119 | 0.61 | 1.34 | 0.001 | 0.112 | 1.000 | 3497 | tags=46%, list=22%, signal=59% |
| 658 | ZHAN\_LATE\_DIFFERENTIATION\_GENES\_UP |  | 32 | 0.65 | 1.34 | 0.029 | 0.112 | 1.000 | 1861 | tags=41%, list=12%, signal=46% |
| 659 | ZHOU\_TNF\_SIGNALING\_30MIN |  | 48 | 0.63 | 1.34 | 0.014 | 0.112 | 1.000 | 2622 | tags=44%, list=17%, signal=52% |
| 660 | PID\_NFAT\_TFPATHWAY |  | 44 | 0.63 | 1.34 | 0.022 | 0.112 | 1.000 | 1172 | tags=16%, list=7%, signal=17% |
| 661 | SETLUR\_PROSTATE\_CANCER\_TMPRSS2\_ERG\_FUSION\_UP |  | 64 | 0.62 | 1.33 | 0.014 | 0.113 | 1.000 | 3492 | tags=42%, list=22%, signal=54% |
| 662 | HIRSCH\_CELLULAR\_TRANSFORMATION\_SIGNATURE\_DN |  | 88 | 0.61 | 1.33 | 0.003 | 0.112 | 1.000 | 1717 | tags=34%, list=11%, signal=38% |
| 663 | DANG\_REGULATED\_BY\_MYC\_UP |  | 63 | 0.62 | 1.33 | 0.011 | 0.112 | 1.000 | 3730 | tags=60%, list=24%, signal=79% |
| 664 | REACTOME\_SIGNALING\_BY\_HIPPO |  | 18 | 0.69 | 1.33 | 0.055 | 0.112 | 1.000 | 3362 | tags=72%, list=21%, signal=92% |
| 665 | FAELT\_B\_CLL\_WITH\_VH\_REARRANGEMENTS\_DN |  | 41 | 0.64 | 1.33 | 0.025 | 0.112 | 1.000 | 2504 | tags=39%, list=16%, signal=46% |
| 666 | TAKAO\_RESPONSE\_TO\_UVB\_RADIATION\_UP |  | 77 | 0.61 | 1.33 | 0.007 | 0.113 | 1.000 | 3369 | tags=48%, list=21%, signal=61% |
| 667 | DACOSTA\_UV\_RESPONSE\_VIA\_ERCC3\_XPCS\_DN |  | 75 | 0.62 | 1.33 | 0.008 | 0.113 | 1.000 | 4399 | tags=63%, list=28%, signal=87% |
| 668 | REACTOME\_INTERFERON\_SIGNALING |  | 128 | 0.60 | 1.33 | 0.001 | 0.114 | 1.000 | 3343 | tags=45%, list=21%, signal=57% |
| 669 | BIOCARTA\_GCR\_PATHWAY |  | 19 | 0.68 | 1.33 | 0.053 | 0.115 | 1.000 | 1168 | tags=26%, list=7%, signal=28% |
| 670 | NEMETH\_INFLAMMATORY\_RESPONSE\_LPS\_DN |  | 30 | 0.66 | 1.33 | 0.033 | 0.115 | 1.000 | 2322 | tags=40%, list=15%, signal=47% |
| 671 | PID\_ECADHERIN\_NASCENTAJ\_PATHWAY |  | 39 | 0.63 | 1.33 | 0.030 | 0.115 | 1.000 | 2223 | tags=46%, list=14%, signal=54% |
| 672 | BIOCARTA\_ARF\_PATHWAY |  | 17 | 0.70 | 1.33 | 0.059 | 0.115 | 1.000 | 3066 | tags=59%, list=19%, signal=73% |
| 673 | REACTOME\_HOMOLOGOUS\_RECOMBINATION\_REPAIR\_OF\_REPLICATION\_INDEPENDENT\_DOUBLE\_STRAND\_BREAKS |  | 16 | 0.69 | 1.33 | 0.071 | 0.115 | 1.000 | 4037 | tags=81%, list=26%, signal=109% |
| 674 | CHARAFE\_BREAST\_CANCER\_LUMINAL\_VS\_MESENCHYMAL\_DN |  | 410 | 0.59 | 1.33 | 0.000 | 0.116 | 1.000 | 3216 | tags=40%, list=20%, signal=50% |
| 675 | BARIS\_THYROID\_CANCER\_UP |  | 22 | 0.67 | 1.33 | 0.051 | 0.116 | 1.000 | 3175 | tags=68%, list=20%, signal=85% |
| 676 | KIM\_ALL\_DISORDERS\_CALB1\_CORR\_UP |  | 499 | 0.59 | 1.33 | 0.000 | 0.117 | 1.000 | 3028 | tags=36%, list=19%, signal=43% |
| 677 | GRABARCZYK\_BCL11B\_TARGETS\_UP |  | 63 | 0.62 | 1.33 | 0.014 | 0.117 | 1.000 | 4233 | tags=52%, list=27%, signal=71% |
| 678 | BIOCARTA\_MEF2D\_PATHWAY |  | 17 | 0.70 | 1.33 | 0.061 | 0.117 | 1.000 | 2445 | tags=41%, list=16%, signal=49% |
| 679 | MCMURRAY\_TP53\_HRAS\_COOPERATION\_RESPONSE\_UP |  | 23 | 0.67 | 1.33 | 0.057 | 0.118 | 1.000 | 2818 | tags=57%, list=18%, signal=69% |
| 680 | HAHTOLA\_MYCOSIS\_FUNGOIDES\_SKIN\_DN |  | 26 | 0.66 | 1.33 | 0.052 | 0.119 | 1.000 | 2623 | tags=46%, list=17%, signal=55% |
| 681 | DER\_IFN\_GAMMA\_RESPONSE\_UP |  | 65 | 0.61 | 1.33 | 0.015 | 0.119 | 1.000 | 3296 | tags=40%, list=21%, signal=50% |
| 682 | REACTOME\_PHOSPHORYLATION\_OF\_THE\_APC\_C |  | 15 | 0.70 | 1.33 | 0.060 | 0.119 | 1.000 | 4379 | tags=87%, list=28%, signal=120% |
| 683 | COLIN\_PILOCYTIC\_ASTROCYTOMA\_VS\_GLIOBLASTOMA\_DN |  | 26 | 0.66 | 1.33 | 0.048 | 0.120 | 1.000 | 2577 | tags=46%, list=16%, signal=55% |
| 684 | RAMASWAMY\_METASTASIS\_UP |  | 56 | 0.62 | 1.33 | 0.009 | 0.120 | 1.000 | 4064 | tags=55%, list=26%, signal=74% |
| 685 | DAZARD\_RESPONSE\_TO\_UV\_NHEK\_DN |  | 273 | 0.59 | 1.33 | 0.000 | 0.120 | 1.000 | 4454 | tags=54%, list=28%, signal=74% |
| 686 | ZHENG\_FOXP3\_TARGETS\_IN\_THYMUS\_UP |  | 171 | 0.60 | 1.33 | 0.000 | 0.121 | 1.000 | 4880 | tags=55%, list=31%, signal=79% |
| 687 | YUAN\_ZNF143\_PARTNERS |  | 18 | 0.69 | 1.33 | 0.063 | 0.121 | 1.000 | 2786 | tags=61%, list=18%, signal=74% |
| 688 | REACTOME\_RNA\_POL\_III\_TRANSCRIPTION |  | 33 | 0.65 | 1.33 | 0.038 | 0.122 | 1.000 | 4179 | tags=64%, list=27%, signal=86% |
| 689 | TCGA\_GLIOBLASTOMA\_COPY\_NUMBER\_UP |  | 62 | 0.62 | 1.33 | 0.011 | 0.122 | 1.000 | 3390 | tags=48%, list=22%, signal=61% |
| 690 | YU\_BAP1\_TARGETS |  | 24 | 0.66 | 1.33 | 0.050 | 0.122 | 1.000 | 4646 | tags=79%, list=29%, signal=112% |
| 691 | REACTOME\_METABOLISM\_OF\_AMINO\_ACIDS\_AND\_DERIVATIVES |  | 189 | 0.60 | 1.33 | 0.000 | 0.123 | 1.000 | 3191 | tags=37%, list=20%, signal=46% |
| 692 | CHIARADONNA\_NEOPLASTIC\_TRANSFORMATION\_KRAS\_CDC25\_UP |  | 53 | 0.62 | 1.33 | 0.024 | 0.123 | 1.000 | 2798 | tags=47%, list=18%, signal=57% |
| 693 | SESTO\_RESPONSE\_TO\_UV\_C0 |  | 101 | 0.61 | 1.32 | 0.001 | 0.123 | 1.000 | 2692 | tags=45%, list=17%, signal=53% |
| 694 | CHEN\_HOXA5\_TARGETS\_9HR\_UP |  | 176 | 0.60 | 1.32 | 0.000 | 0.126 | 1.000 | 3750 | tags=48%, list=24%, signal=62% |
| 695 | YIH\_RESPONSE\_TO\_ARSENITE\_C2 |  | 18 | 0.68 | 1.32 | 0.067 | 0.126 | 1.000 | 2850 | tags=50%, list=18%, signal=61% |
| 696 | PID\_INTEGRIN\_A4B1\_PATHWAY |  | 33 | 0.64 | 1.32 | 0.042 | 0.126 | 1.000 | 3891 | tags=45%, list=25%, signal=60% |
| 697 | STANELLE\_E2F1\_TARGETS |  | 26 | 0.67 | 1.32 | 0.044 | 0.126 | 1.000 | 2115 | tags=35%, list=13%, signal=40% |
| 698 | JIANG\_HYPOXIA\_CANCER |  | 72 | 0.61 | 1.32 | 0.011 | 0.127 | 1.000 | 2945 | tags=43%, list=19%, signal=53% |
| 699 | KUNINGER\_IGF1\_VS\_PDGFB\_TARGETS\_DN |  | 40 | 0.63 | 1.32 | 0.029 | 0.127 | 1.000 | 1751 | tags=40%, list=11%, signal=45% |
| 700 | DAZARD\_RESPONSE\_TO\_UV\_SCC\_DN |  | 109 | 0.60 | 1.32 | 0.001 | 0.128 | 1.000 | 4349 | tags=62%, list=28%, signal=86% |
| 701 | DORSAM\_HOXA9\_TARGETS\_UP |  | 32 | 0.65 | 1.32 | 0.040 | 0.127 | 1.000 | 3502 | tags=53%, list=22%, signal=68% |
| 702 | REACTOME\_AMINO\_ACID\_SYNTHESIS\_AND\_INTERCONVERSION\_TRANSAMINATION |  | 16 | 0.71 | 1.32 | 0.062 | 0.127 | 1.000 | 1616 | tags=56%, list=10%, signal=63% |
| 703 | CHNG\_MULTIPLE\_MYELOMA\_HYPERPLOID\_DN |  | 28 | 0.65 | 1.32 | 0.041 | 0.128 | 1.000 | 3816 | tags=61%, list=24%, signal=80% |
| 704 | JIANG\_AGING\_HYPOTHALAMUS\_DN |  | 40 | 0.62 | 1.32 | 0.046 | 0.129 | 1.000 | 1501 | tags=38%, list=10%, signal=41% |
| 705 | CAIRO\_LIVER\_DEVELOPMENT\_UP |  | 152 | 0.59 | 1.32 | 0.000 | 0.129 | 1.000 | 2890 | tags=32%, list=18%, signal=39% |
| 706 | OSMAN\_BLADDER\_CANCER\_DN |  | 314 | 0.59 | 1.32 | 0.000 | 0.130 | 1.000 | 3983 | tags=48%, list=25%, signal=63% |
| 707 | SPIRA\_SMOKERS\_LUNG\_CANCER\_UP |  | 35 | 0.64 | 1.32 | 0.040 | 0.130 | 1.000 | 817 | tags=26%, list=5%, signal=27% |
| 708 | REACTOME\_DESTABILIZATION\_OF\_MRNA\_BY\_KSRP |  | 17 | 0.70 | 1.32 | 0.069 | 0.130 | 1.000 | 4425 | tags=82%, list=28%, signal=114% |
| 709 | DAZARD\_UV\_RESPONSE\_CLUSTER\_G28 |  | 16 | 0.69 | 1.32 | 0.068 | 0.132 | 1.000 | 435 | tags=19%, list=3%, signal=19% |
| 710 | KAPOSI\_LIVER\_CANCER\_MET\_UP |  | 17 | 0.69 | 1.32 | 0.063 | 0.132 | 1.000 | 2842 | tags=53%, list=18%, signal=65% |
| 711 | SARTIPY\_BLUNTED\_BY\_INSULIN\_RESISTANCE\_UP |  | 16 | 0.69 | 1.32 | 0.077 | 0.132 | 1.000 | 3491 | tags=63%, list=22%, signal=80% |
| 712 | KEGG\_BASE\_EXCISION\_REPAIR |  | 33 | 0.64 | 1.32 | 0.038 | 0.132 | 1.000 | 4607 | tags=64%, list=29%, signal=90% |
| 713 | SWEET\_KRAS\_TARGETS\_DN |  | 56 | 0.61 | 1.32 | 0.032 | 0.132 | 1.000 | 3489 | tags=41%, list=22%, signal=53% |
| 714 | KYNG\_ENVIRONMENTAL\_STRESS\_RESPONSE\_UP |  | 52 | 0.62 | 1.32 | 0.024 | 0.132 | 1.000 | 2881 | tags=40%, list=18%, signal=49% |
| 715 | REACTOME\_ENOS\_ACTIVATION\_AND\_REGULATION |  | 19 | 0.68 | 1.32 | 0.061 | 0.132 | 1.000 | 3154 | tags=53%, list=20%, signal=66% |
| 716 | YOSHIMURA\_MAPK8\_TARGETS\_DN |  | 338 | 0.59 | 1.32 | 0.000 | 0.133 | 1.000 | 3310 | tags=42%, list=21%, signal=52% |
| 717 | KEGG\_PYRIMIDINE\_METABOLISM |  | 89 | 0.61 | 1.32 | 0.005 | 0.134 | 1.000 | 3982 | tags=60%, list=25%, signal=79% |
| 718 | RIZ\_ERYTHROID\_DIFFERENTIATION\_HBZ |  | 38 | 0.63 | 1.32 | 0.037 | 0.134 | 1.000 | 2925 | tags=29%, list=19%, signal=35% |
| 719 | DAVICIONI\_PAX\_FOXO1\_SIGNATURE\_IN\_ARMS\_DN |  | 18 | 0.69 | 1.32 | 0.065 | 0.134 | 1.000 | 3209 | tags=39%, list=20%, signal=49% |
| 720 | PID\_MYC\_PATHWAY |  | 23 | 0.66 | 1.32 | 0.062 | 0.134 | 1.000 | 3146 | tags=52%, list=20%, signal=65% |
| 721 | ABRAHAM\_ALPC\_VS\_MULTIPLE\_MYELOMA\_DN |  | 18 | 0.69 | 1.32 | 0.080 | 0.135 | 1.000 | 2895 | tags=50%, list=18%, signal=61% |
| 722 | ACEVEDO\_NORMAL\_TISSUE\_ADJACENT\_TO\_LIVER\_TUMOR\_UP |  | 139 | 0.59 | 1.32 | 0.001 | 0.135 | 1.000 | 2864 | tags=38%, list=18%, signal=46% |
| 723 | ZHAN\_EARLY\_DIFFERENTIATION\_GENES\_DN |  | 39 | 0.63 | 1.31 | 0.034 | 0.136 | 1.000 | 2393 | tags=41%, list=15%, signal=48% |
| 724 | GARGALOVIC\_RESPONSE\_TO\_OXIDIZED\_PHOSPHOLIPIDS\_YELLOW\_UP |  | 24 | 0.65 | 1.31 | 0.069 | 0.136 | 1.000 | 3302 | tags=50%, list=21%, signal=63% |
| 725 | BILANGES\_SERUM\_SENSITIVE\_VIA\_TSC2 |  | 35 | 0.64 | 1.31 | 0.039 | 0.137 | 1.000 | 3210 | tags=54%, list=20%, signal=68% |
| 726 | OXFORD\_RALA\_OR\_RALB\_TARGETS\_DN |  | 23 | 0.66 | 1.31 | 0.055 | 0.137 | 1.000 | 1426 | tags=30%, list=9%, signal=33% |
| 727 | HOLLMANN\_APOPTOSIS\_VIA\_CD40\_UP |  | 177 | 0.59 | 1.31 | 0.000 | 0.137 | 1.000 | 3687 | tags=46%, list=23%, signal=60% |
| 728 | TAYLOR\_METHYLATED\_IN\_ACUTE\_LYMPHOBLASTIC\_LEUKEMIA |  | 62 | 0.61 | 1.31 | 0.012 | 0.137 | 1.000 | 3110 | tags=40%, list=20%, signal=50% |
| 729 | CHEOK\_RESPONSE\_TO\_HD\_MTX\_DN |  | 21 | 0.67 | 1.31 | 0.073 | 0.138 | 1.000 | 4194 | tags=62%, list=27%, signal=84% |
| 730 | CHESLER\_BRAIN\_HIGHEST\_EXPRESSION |  | 36 | 0.63 | 1.31 | 0.040 | 0.138 | 1.000 | 3457 | tags=47%, list=22%, signal=60% |
| 731 | YORDY\_RECIPROCAL\_REGULATION\_BY\_ETS1\_AND\_SP100\_UP |  | 20 | 0.67 | 1.31 | 0.063 | 0.139 | 1.000 | 2625 | tags=50%, list=17%, signal=60% |
| 732 | GRADE\_METASTASIS\_DN |  | 39 | 0.63 | 1.31 | 0.033 | 0.139 | 1.000 | 3191 | tags=59%, list=20%, signal=74% |
| 733 | HOLLEMAN\_VINCRISTINE\_RESISTANCE\_ALL\_UP |  | 23 | 0.66 | 1.31 | 0.061 | 0.139 | 1.000 | 818 | tags=22%, list=5%, signal=23% |
| 734 | GARGALOVIC\_RESPONSE\_TO\_OXIDIZED\_PHOSPHOLIPIDS\_MAGENTA\_UP |  | 22 | 0.66 | 1.31 | 0.067 | 0.139 | 1.000 | 4160 | tags=73%, list=26%, signal=99% |
| 735 | BROWNE\_HCMV\_INFECTION\_2HR\_DN |  | 47 | 0.61 | 1.31 | 0.044 | 0.139 | 1.000 | 3209 | tags=45%, list=20%, signal=56% |
| 736 | BURTON\_ADIPOGENESIS\_PEAK\_AT\_8HR |  | 36 | 0.63 | 1.31 | 0.036 | 0.140 | 1.000 | 1314 | tags=36%, list=8%, signal=39% |
| 737 | WELCSH\_BRCA1\_TARGETS\_UP |  | 179 | 0.59 | 1.31 | 0.002 | 0.140 | 1.000 | 2332 | tags=35%, list=15%, signal=41% |
| 738 | PID\_VEGFR1\_PATHWAY |  | 25 | 0.65 | 1.31 | 0.053 | 0.141 | 1.000 | 4098 | tags=64%, list=26%, signal=86% |
| 739 | BHATI\_G2M\_ARREST\_BY\_2METHOXYESTRADIOL\_UP |  | 102 | 0.60 | 1.31 | 0.004 | 0.141 | 1.000 | 2587 | tags=38%, list=16%, signal=45% |
| 740 | BIOCARTA\_CASPASE\_PATHWAY |  | 21 | 0.66 | 1.31 | 0.073 | 0.140 | 1.000 | 3711 | tags=43%, list=24%, signal=56% |
| 741 | LEE\_METASTASIS\_AND\_ALTERNATIVE\_SPLICING\_DN |  | 43 | 0.62 | 1.31 | 0.038 | 0.141 | 1.000 | 2209 | tags=26%, list=14%, signal=30% |
| 742 | DAZARD\_UV\_RESPONSE\_CLUSTER\_G1 |  | 60 | 0.61 | 1.31 | 0.019 | 0.142 | 1.000 | 3003 | tags=50%, list=19%, signal=62% |
| 743 | REACTOME\_DESTABILIZATION\_OF\_MRNA\_BY\_BRF1 |  | 17 | 0.69 | 1.31 | 0.079 | 0.142 | 1.000 | 2040 | tags=47%, list=13%, signal=54% |
| 744 | REACTOME\_TRAFFICKING\_OF\_AMPA\_RECEPTORS |  | 24 | 0.66 | 1.31 | 0.053 | 0.143 | 1.000 | 3484 | tags=33%, list=22%, signal=43% |
| 745 | SHEPARD\_BMYB\_MORPHOLINO\_DN |  | 176 | 0.59 | 1.31 | 0.001 | 0.143 | 1.000 | 2645 | tags=35%, list=17%, signal=42% |
| 746 | DUTERTRE\_ESTRADIOL\_RESPONSE\_6HR\_UP |  | 206 | 0.59 | 1.31 | 0.000 | 0.143 | 1.000 | 4164 | tags=50%, list=26%, signal=68% |
| 747 | UDAYAKUMAR\_MED1\_TARGETS\_UP |  | 119 | 0.59 | 1.31 | 0.001 | 0.143 | 1.000 | 4186 | tags=64%, list=27%, signal=86% |
| 748 | HELLER\_HDAC\_TARGETS\_DN |  | 253 | 0.59 | 1.31 | 0.000 | 0.143 | 1.000 | 2869 | tags=35%, list=18%, signal=42% |
| 749 | BHATI\_G2M\_ARREST\_BY\_2METHOXYESTRADIOL\_DN |  | 107 | 0.59 | 1.31 | 0.003 | 0.143 | 1.000 | 2045 | tags=28%, list=13%, signal=32% |
| 750 | MARCINIAK\_ER\_STRESS\_RESPONSE\_VIA\_CHOP |  | 24 | 0.65 | 1.31 | 0.064 | 0.143 | 1.000 | 3061 | tags=54%, list=19%, signal=67% |
| 751 | SPIELMAN\_LYMPHOBLAST\_EUROPEAN\_VS\_ASIAN\_UP |  | 431 | 0.58 | 1.31 | 0.000 | 0.143 | 1.000 | 3031 | tags=41%, list=19%, signal=49% |
| 752 | BROWNE\_HCMV\_INFECTION\_14HR\_UP |  | 131 | 0.59 | 1.31 | 0.000 | 0.144 | 1.000 | 3986 | tags=51%, list=25%, signal=68% |
| 753 | THEILGAARD\_NEUTROPHIL\_AT\_SKIN\_WOUND\_UP |  | 68 | 0.60 | 1.31 | 0.022 | 0.144 | 1.000 | 3144 | tags=41%, list=20%, signal=51% |
| 754 | GAZIN\_EPIGENETIC\_SILENCING\_BY\_KRAS |  | 25 | 0.65 | 1.31 | 0.071 | 0.144 | 1.000 | 2587 | tags=40%, list=16%, signal=48% |
| 755 | WHITEHURST\_PACLITAXEL\_SENSITIVITY |  | 33 | 0.63 | 1.31 | 0.048 | 0.145 | 1.000 | 2328 | tags=27%, list=15%, signal=32% |
| 756 | AMUNDSON\_POOR\_SURVIVAL\_AFTER\_GAMMA\_RADIATION\_2G |  | 147 | 0.59 | 1.31 | 0.002 | 0.146 | 1.000 | 3534 | tags=42%, list=22%, signal=54% |
| 757 | SHIPP\_DLBCL\_CURED\_VS\_FATAL\_DN |  | 41 | 0.62 | 1.31 | 0.048 | 0.146 | 1.000 | 1424 | tags=27%, list=9%, signal=29% |
| 758 | HOWLIN\_CITED1\_TARGETS\_1\_DN |  | 35 | 0.63 | 1.31 | 0.039 | 0.147 | 1.000 | 3828 | tags=49%, list=24%, signal=64% |
| 759 | COLLIS\_PRKDC\_SUBSTRATES |  | 18 | 0.68 | 1.31 | 0.069 | 0.147 | 1.000 | 2895 | tags=50%, list=18%, signal=61% |
| 760 | HE\_PTEN\_TARGETS\_UP |  | 16 | 0.68 | 1.30 | 0.090 | 0.147 | 1.000 | 3079 | tags=50%, list=20%, signal=62% |
| 761 | BIOCARTA\_ACTINY\_PATHWAY |  | 20 | 0.67 | 1.30 | 0.066 | 0.147 | 1.000 | 3394 | tags=50%, list=22%, signal=64% |
| 762 | WANG\_RESPONSE\_TO\_ANDROGEN\_UP |  | 28 | 0.64 | 1.30 | 0.069 | 0.147 | 1.000 | 3249 | tags=46%, list=21%, signal=58% |
| 763 | LUI\_TARGETS\_OF\_PAX8\_PPARG\_FUSION |  | 27 | 0.64 | 1.30 | 0.084 | 0.147 | 1.000 | 2018 | tags=48%, list=13%, signal=55% |
| 764 | REACTOME\_FATTY\_ACYL\_COA\_BIOSYNTHESIS |  | 17 | 0.69 | 1.30 | 0.075 | 0.148 | 1.000 | 2187 | tags=35%, list=14%, signal=41% |
| 765 | KYNG\_DNA\_DAMAGE\_BY\_GAMMA\_AND\_UV\_RADIATION |  | 84 | 0.60 | 1.30 | 0.011 | 0.149 | 1.000 | 3290 | tags=39%, list=21%, signal=49% |
| 766 | DORN\_ADENOVIRUS\_INFECTION\_24HR\_DN |  | 38 | 0.62 | 1.30 | 0.035 | 0.149 | 1.000 | 3036 | tags=50%, list=19%, signal=62% |
| 767 | YANAGIHARA\_ESX1\_TARGETS |  | 28 | 0.63 | 1.30 | 0.065 | 0.149 | 1.000 | 1801 | tags=29%, list=11%, signal=32% |
| 768 | MARIADASON\_RESPONSE\_TO\_CURCUMIN\_SULINDAC\_5 |  | 23 | 0.66 | 1.30 | 0.055 | 0.150 | 1.000 | 1971 | tags=39%, list=13%, signal=45% |
| 769 | CROONQUIST\_NRAS\_VS\_STROMAL\_STIMULATION\_DN |  | 87 | 0.60 | 1.30 | 0.007 | 0.151 | 1.000 | 2917 | tags=45%, list=19%, signal=55% |
| 770 | REACTOME\_SIGNALING\_BY\_TGF\_BETA\_RECEPTOR\_COMPLEX |  | 59 | 0.60 | 1.30 | 0.024 | 0.150 | 1.000 | 4111 | tags=61%, list=26%, signal=82% |
| 771 | REACTOME\_INSULIN\_SYNTHESIS\_AND\_PROCESSING |  | 20 | 0.66 | 1.30 | 0.071 | 0.150 | 1.000 | 2352 | tags=40%, list=15%, signal=47% |
| 772 | TONKS\_TARGETS\_OF\_RUNX1\_RUNX1T1\_FUSION\_MONOCYTE\_UP |  | 176 | 0.59 | 1.30 | 0.000 | 0.151 | 1.000 | 2979 | tags=40%, list=19%, signal=49% |
| 773 | BOUDOUKHA\_BOUND\_BY\_IGF2BP2 |  | 96 | 0.59 | 1.30 | 0.006 | 0.151 | 1.000 | 3270 | tags=49%, list=21%, signal=61% |
| 774 | REACTOME\_GRB2\_SOS\_PROVIDES\_LINKAGE\_TO\_MAPK\_SIGNALING\_FOR\_INTERGRINS\_ |  | 15 | 0.69 | 1.30 | 0.085 | 0.151 | 1.000 | 3328 | tags=47%, list=21%, signal=59% |
| 775 | WU\_HBX\_TARGETS\_2\_UP |  | 20 | 0.67 | 1.30 | 0.075 | 0.152 | 1.000 | 3078 | tags=50%, list=20%, signal=62% |
| 776 | MOHANKUMAR\_TLX1\_TARGETS\_UP |  | 362 | 0.58 | 1.30 | 0.000 | 0.152 | 1.000 | 3227 | tags=42%, list=20%, signal=51% |
| 777 | GENTILE\_UV\_RESPONSE\_CLUSTER\_D2 |  | 38 | 0.63 | 1.30 | 0.035 | 0.152 | 1.000 | 3352 | tags=50%, list=21%, signal=63% |
| 778 | NUTT\_GBM\_VS\_AO\_GLIOMA\_DN |  | 40 | 0.62 | 1.30 | 0.045 | 0.152 | 1.000 | 4015 | tags=48%, list=25%, signal=64% |
| 779 | WOOD\_EBV\_EBNA1\_TARGETS\_DN |  | 41 | 0.62 | 1.30 | 0.050 | 0.152 | 1.000 | 993 | tags=29%, list=6%, signal=31% |
| 780 | WENG\_POR\_DOSAGE |  | 18 | 0.68 | 1.30 | 0.069 | 0.152 | 1.000 | 709 | tags=22%, list=5%, signal=23% |
| 781 | WARTERS\_IR\_RESPONSE\_5GY |  | 39 | 0.63 | 1.30 | 0.037 | 0.153 | 1.000 | 2281 | tags=23%, list=14%, signal=27% |
| 782 | BIOCARTA\_UCALPAIN\_PATHWAY |  | 17 | 0.68 | 1.30 | 0.070 | 0.153 | 1.000 | 956 | tags=24%, list=6%, signal=25% |
| 783 | BECKER\_TAMOXIFEN\_RESISTANCE\_DN |  | 46 | 0.61 | 1.30 | 0.037 | 0.153 | 1.000 | 3922 | tags=46%, list=25%, signal=61% |
| 784 | CHIARADONNA\_NEOPLASTIC\_TRANSFORMATION\_CDC25\_UP |  | 112 | 0.59 | 1.30 | 0.003 | 0.154 | 1.000 | 3307 | tags=44%, list=21%, signal=55% |
| 785 | AMIT\_SERUM\_RESPONSE\_60\_MCF10A |  | 52 | 0.61 | 1.30 | 0.020 | 0.154 | 1.000 | 2001 | tags=29%, list=13%, signal=33% |
| 786 | KEGG\_AMYOTROPHIC\_LATERAL\_SCLEROSIS\_ALS |  | 52 | 0.61 | 1.30 | 0.030 | 0.154 | 1.000 | 2626 | tags=31%, list=17%, signal=37% |
| 787 | BIOCARTA\_CALCINEURIN\_PATHWAY |  | 17 | 0.68 | 1.30 | 0.085 | 0.154 | 1.000 | 4482 | tags=71%, list=28%, signal=99% |
| 788 | BAELDE\_DIABETIC\_NEPHROPATHY\_DN |  | 393 | 0.58 | 1.30 | 0.000 | 0.154 | 1.000 | 3093 | tags=38%, list=20%, signal=46% |
| 789 | LEE\_LIVER\_CANCER\_DENA\_UP |  | 55 | 0.61 | 1.30 | 0.034 | 0.155 | 1.000 | 2977 | tags=36%, list=19%, signal=45% |
| 790 | KUROKAWA\_LIVER\_CANCER\_CHEMOTHERAPY\_DN |  | 38 | 0.63 | 1.30 | 0.040 | 0.156 | 1.000 | 3196 | tags=50%, list=20%, signal=63% |
| 791 | REACTOME\_ACTIVATION\_OF\_BH3\_ONLY\_PROTEINS |  | 16 | 0.69 | 1.30 | 0.073 | 0.157 | 1.000 | 2178 | tags=44%, list=14%, signal=51% |
| 792 | SINGH\_NFE2L2\_TARGETS |  | 15 | 0.69 | 1.30 | 0.083 | 0.157 | 1.000 | 2724 | tags=60%, list=17%, signal=72% |
| 793 | ZHAN\_MULTIPLE\_MYELOMA\_HP\_UP |  | 41 | 0.61 | 1.30 | 0.040 | 0.159 | 1.000 | 4047 | tags=51%, list=26%, signal=69% |
| 794 | OKAMOTO\_LIVER\_CANCER\_MULTICENTRIC\_OCCURRENCE\_UP |  | 22 | 0.65 | 1.30 | 0.082 | 0.159 | 1.000 | 2615 | tags=45%, list=17%, signal=54% |
| 795 | ZHENG\_RESPONSE\_TO\_ARSENITE\_DN |  | 17 | 0.68 | 1.30 | 0.089 | 0.160 | 1.000 | 3659 | tags=53%, list=23%, signal=69% |
| 796 | JAIN\_NFKB\_SIGNALING |  | 66 | 0.60 | 1.29 | 0.016 | 0.160 | 1.000 | 4656 | tags=65%, list=30%, signal=92% |
| 797 | EINAV\_INTERFERON\_SIGNATURE\_IN\_CANCER |  | 22 | 0.66 | 1.29 | 0.082 | 0.160 | 1.000 | 3057 | tags=50%, list=19%, signal=62% |
| 798 | KEGG\_P53\_SIGNALING\_PATHWAY |  | 62 | 0.60 | 1.29 | 0.024 | 0.161 | 1.000 | 4063 | tags=50%, list=26%, signal=67% |
| 799 | LINDGREN\_BLADDER\_CANCER\_WITH\_LOH\_IN\_CHR9Q |  | 94 | 0.59 | 1.29 | 0.003 | 0.163 | 1.000 | 3250 | tags=43%, list=21%, signal=53% |
| 800 | BUCKANOVICH\_T\_LYMPHOCYTE\_HOMING\_ON\_TUMOR\_UP |  | 19 | 0.67 | 1.29 | 0.080 | 0.163 | 1.000 | 1942 | tags=37%, list=12%, signal=42% |
| 801 | KYNG\_RESPONSE\_TO\_H2O2\_VIA\_ERCC6\_UP |  | 36 | 0.62 | 1.29 | 0.065 | 0.163 | 1.000 | 3941 | tags=53%, list=25%, signal=70% |
| 802 | REACTOME\_NEGATIVE\_REGULATORS\_OF\_RIG\_I\_MDA5\_SIGNALING |  | 26 | 0.64 | 1.29 | 0.067 | 0.163 | 1.000 | 2644 | tags=42%, list=17%, signal=51% |
| 803 | LOPES\_METHYLATED\_IN\_COLON\_CANCER\_DN |  | 28 | 0.64 | 1.29 | 0.067 | 0.163 | 1.000 | 4483 | tags=46%, list=28%, signal=65% |
| 804 | CHENG\_RESPONSE\_TO\_NICKEL\_ACETATE |  | 40 | 0.62 | 1.29 | 0.049 | 0.164 | 1.000 | 3882 | tags=55%, list=25%, signal=73% |
| 805 | TAKADA\_GASTRIC\_CANCER\_COPY\_NUMBER\_DN |  | 26 | 0.64 | 1.29 | 0.070 | 0.164 | 1.000 | 4393 | tags=38%, list=28%, signal=53% |
| 806 | DEBOSSCHER\_NFKB\_TARGETS\_REPRESSED\_BY\_GLUCOCORTICOIDS |  | 21 | 0.66 | 1.29 | 0.096 | 0.164 | 1.000 | 1626 | tags=19%, list=10%, signal=21% |
| 807 | BIOCARTA\_CHREBP2\_PATHWAY |  | 40 | 0.62 | 1.29 | 0.051 | 0.164 | 1.000 | 2582 | tags=43%, list=16%, signal=51% |
| 808 | NGUYEN\_NOTCH1\_TARGETS\_DN |  | 81 | 0.59 | 1.29 | 0.010 | 0.164 | 1.000 | 2954 | tags=42%, list=19%, signal=51% |
| 809 | GROSS\_HYPOXIA\_VIA\_ELK3\_DN |  | 147 | 0.58 | 1.29 | 0.003 | 0.164 | 1.000 | 2704 | tags=37%, list=17%, signal=44% |
| 810 | BURTON\_ADIPOGENESIS\_8 |  | 82 | 0.59 | 1.29 | 0.019 | 0.164 | 1.000 | 3641 | tags=57%, list=23%, signal=74% |
| 811 | OSWALD\_HEMATOPOIETIC\_STEM\_CELL\_IN\_COLLAGEN\_GEL\_UP |  | 207 | 0.58 | 1.29 | 0.000 | 0.164 | 1.000 | 2507 | tags=29%, list=16%, signal=34% |
| 812 | BOYLAN\_MULTIPLE\_MYELOMA\_C\_UP |  | 38 | 0.62 | 1.29 | 0.050 | 0.164 | 1.000 | 3595 | tags=45%, list=23%, signal=58% |
| 813 | BROWNE\_HCMV\_INFECTION\_18HR\_UP |  | 165 | 0.58 | 1.29 | 0.000 | 0.166 | 1.000 | 4328 | tags=47%, list=27%, signal=65% |
| 814 | APPIERTO\_RESPONSE\_TO\_FENRETINIDE\_DN |  | 43 | 0.61 | 1.29 | 0.040 | 0.167 | 1.000 | 2951 | tags=42%, list=19%, signal=51% |
| 815 | TANAKA\_METHYLATED\_IN\_ESOPHAGEAL\_CARCINOMA |  | 94 | 0.59 | 1.29 | 0.008 | 0.168 | 1.000 | 1908 | tags=19%, list=12%, signal=22% |
| 816 | TOMIDA\_METASTASIS\_UP |  | 21 | 0.65 | 1.29 | 0.087 | 0.168 | 1.000 | 2056 | tags=38%, list=13%, signal=44% |
| 817 | MUNSHI\_MULTIPLE\_MYELOMA\_UP |  | 78 | 0.59 | 1.29 | 0.018 | 0.169 | 1.000 | 2216 | tags=35%, list=14%, signal=40% |
| 818 | NOJIMA\_SFRP2\_TARGETS\_DN |  | 23 | 0.64 | 1.29 | 0.094 | 0.169 | 1.000 | 3701 | tags=52%, list=23%, signal=68% |
| 819 | PID\_ERA\_GENOMIC\_PATHWAY |  | 62 | 0.60 | 1.29 | 0.031 | 0.171 | 1.000 | 3078 | tags=35%, list=20%, signal=44% |
| 820 | MARSON\_FOXP3\_TARGETS\_DN |  | 47 | 0.61 | 1.29 | 0.047 | 0.171 | 1.000 | 4056 | tags=40%, list=26%, signal=54% |
| 821 | BIOCARTA\_RACCYCD\_PATHWAY |  | 26 | 0.64 | 1.29 | 0.069 | 0.171 | 1.000 | 3201 | tags=58%, list=20%, signal=72% |
| 822 | FRASOR\_RESPONSE\_TO\_SERM\_OR\_FULVESTRANT\_UP |  | 18 | 0.66 | 1.29 | 0.101 | 0.172 | 1.000 | 2912 | tags=44%, list=18%, signal=54% |
| 823 | YIH\_RESPONSE\_TO\_ARSENITE\_C3 |  | 33 | 0.62 | 1.29 | 0.059 | 0.172 | 1.000 | 3621 | tags=48%, list=23%, signal=63% |
| 824 | RAY\_TUMORIGENESIS\_BY\_ERBB2\_CDC25A\_UP |  | 94 | 0.59 | 1.29 | 0.009 | 0.173 | 1.000 | 2286 | tags=28%, list=15%, signal=32% |
| 825 | FERRANDO\_HOX11\_NEIGHBORS |  | 19 | 0.66 | 1.29 | 0.088 | 0.173 | 1.000 | 815 | tags=37%, list=5%, signal=39% |
| 826 | CAIRO\_HEPATOBLASTOMA\_UP |  | 179 | 0.58 | 1.29 | 0.000 | 0.174 | 1.000 | 2862 | tags=41%, list=18%, signal=49% |
| 827 | THILLAINADESAN\_ZNF217\_TARGETS\_UP |  | 35 | 0.63 | 1.29 | 0.056 | 0.174 | 1.000 | 2974 | tags=54%, list=19%, signal=67% |
| 828 | NEMETH\_INFLAMMATORY\_RESPONSE\_LPS\_UP |  | 83 | 0.59 | 1.28 | 0.019 | 0.175 | 1.000 | 3561 | tags=43%, list=23%, signal=56% |
| 829 | BRACHAT\_RESPONSE\_TO\_METHOTREXATE\_DN |  | 23 | 0.65 | 1.28 | 0.081 | 0.175 | 1.000 | 2906 | tags=39%, list=18%, signal=48% |
| 830 | SAGIV\_CD24\_TARGETS\_UP |  | 22 | 0.65 | 1.28 | 0.088 | 0.175 | 1.000 | 3006 | tags=45%, list=19%, signal=56% |
| 831 | GRAHAM\_NORMAL\_QUIESCENT\_VS\_NORMAL\_DIVIDING\_UP |  | 58 | 0.60 | 1.28 | 0.038 | 0.175 | 1.000 | 3605 | tags=31%, list=23%, signal=40% |
| 832 | RICKMAN\_METASTASIS\_UP |  | 263 | 0.57 | 1.28 | 0.000 | 0.177 | 1.000 | 4236 | tags=47%, list=27%, signal=63% |
| 833 | LU\_AGING\_BRAIN\_DN |  | 141 | 0.58 | 1.28 | 0.003 | 0.177 | 1.000 | 2880 | tags=33%, list=18%, signal=40% |
| 834 | PID\_LKB1\_PATHWAY |  | 38 | 0.61 | 1.28 | 0.055 | 0.177 | 1.000 | 4113 | tags=50%, list=26%, signal=68% |
| 835 | BIOCARTA\_HIF\_PATHWAY |  | 15 | 0.69 | 1.28 | 0.087 | 0.178 | 1.000 | 946 | tags=33%, list=6%, signal=35% |
| 836 | PID\_HDAC\_CLASSII\_PATHWAY |  | 33 | 0.62 | 1.28 | 0.066 | 0.178 | 1.000 | 2565 | tags=39%, list=16%, signal=47% |
| 837 | HERNANDEZ\_MITOTIC\_ARREST\_BY\_DOCETAXEL\_1\_DN |  | 35 | 0.62 | 1.28 | 0.053 | 0.178 | 1.000 | 3927 | tags=49%, list=25%, signal=65% |
| 838 | ZHONG\_SECRETOME\_OF\_LUNG\_CANCER\_AND\_FIBROBLAST |  | 129 | 0.58 | 1.28 | 0.004 | 0.178 | 1.000 | 2577 | tags=43%, list=16%, signal=51% |
| 839 | DIRMEIER\_LMP1\_RESPONSE\_LATE\_UP |  | 52 | 0.60 | 1.28 | 0.041 | 0.180 | 1.000 | 2563 | tags=42%, list=16%, signal=50% |
| 840 | GOTZMANN\_EPITHELIAL\_TO\_MESENCHYMAL\_TRANSITION\_UP |  | 67 | 0.59 | 1.28 | 0.027 | 0.180 | 1.000 | 3179 | tags=45%, list=20%, signal=56% |
| 841 | KEGG\_CITRATE\_CYCLE\_TCA\_CYCLE |  | 30 | 0.63 | 1.28 | 0.090 | 0.180 | 1.000 | 3882 | tags=60%, list=25%, signal=79% |
| 842 | ACOSTA\_PROLIFERATION\_INDEPENDENT\_MYC\_TARGETS\_UP |  | 74 | 0.59 | 1.28 | 0.027 | 0.180 | 1.000 | 3964 | tags=49%, list=25%, signal=65% |
| 843 | HONMA\_DOCETAXEL\_RESISTANCE |  | 32 | 0.62 | 1.28 | 0.066 | 0.180 | 1.000 | 4690 | tags=78%, list=30%, signal=111% |
| 844 | ZHAN\_V2\_LATE\_DIFFERENTIATION\_GENES |  | 43 | 0.61 | 1.28 | 0.046 | 0.182 | 1.000 | 3179 | tags=49%, list=20%, signal=61% |
| 845 | TURASHVILI\_BREAST\_DUCTAL\_CARCINOMA\_VS\_DUCTAL\_NORMAL\_UP |  | 38 | 0.62 | 1.28 | 0.064 | 0.182 | 1.000 | 3435 | tags=39%, list=22%, signal=50% |
| 846 | REACTOME\_SMAD2\_SMAD3\_SMAD4\_HETEROTRIMER\_REGULATES\_TRANSCRIPTION |  | 25 | 0.63 | 1.28 | 0.094 | 0.182 | 1.000 | 3865 | tags=60%, list=25%, signal=79% |
| 847 | BENPORATH\_MYC\_TARGETS\_WITH\_EBOX |  | 214 | 0.57 | 1.28 | 0.000 | 0.183 | 1.000 | 2820 | tags=36%, list=18%, signal=43% |
| 848 | KEGG\_SMALL\_CELL\_LUNG\_CANCER |  | 81 | 0.58 | 1.28 | 0.012 | 0.183 | 1.000 | 3201 | tags=40%, list=20%, signal=49% |
| 849 | XU\_HGF\_TARGETS\_INDUCED\_BY\_AKT1\_6HR |  | 17 | 0.67 | 1.28 | 0.104 | 0.182 | 1.000 | 3393 | tags=59%, list=22%, signal=75% |
| 850 | DER\_IFN\_ALPHA\_RESPONSE\_UP |  | 64 | 0.60 | 1.28 | 0.029 | 0.182 | 1.000 | 3296 | tags=38%, list=21%, signal=47% |
| 851 | LIANG\_HEMATOPOIESIS\_STEM\_CELL\_NUMBER\_SMALL\_VS\_HUGE\_UP |  | 33 | 0.62 | 1.28 | 0.063 | 0.185 | 1.000 | 3029 | tags=42%, list=19%, signal=52% |
| 852 | LINDGREN\_BLADDER\_CANCER\_CLUSTER\_1\_UP |  | 96 | 0.58 | 1.28 | 0.011 | 0.185 | 1.000 | 3437 | tags=42%, list=22%, signal=53% |
| 853 | MARKS\_ACETYLATED\_NON\_HISTONE\_PROTEINS |  | 15 | 0.67 | 1.28 | 0.122 | 0.185 | 1.000 | 3442 | tags=47%, list=22%, signal=60% |
| 854 | JOHNSTONE\_PARVB\_TARGETS\_1\_DN |  | 45 | 0.60 | 1.28 | 0.057 | 0.185 | 1.000 | 3752 | tags=42%, list=24%, signal=55% |
| 855 | LEE\_AGING\_MUSCLE\_DN |  | 42 | 0.61 | 1.28 | 0.051 | 0.185 | 1.000 | 2313 | tags=31%, list=15%, signal=36% |
| 856 | LEE\_CALORIE\_RESTRICTION\_NEOCORTEX\_DN |  | 84 | 0.59 | 1.28 | 0.021 | 0.185 | 1.000 | 1665 | tags=26%, list=11%, signal=29% |
| 857 | REACTOME\_DOWNREGULATION\_OF\_TGF\_BETA\_RECEPTOR\_SIGNALING |  | 20 | 0.66 | 1.28 | 0.094 | 0.186 | 1.000 | 4435 | tags=75%, list=28%, signal=104% |
| 858 | XU\_AKT1\_TARGETS\_6HR |  | 22 | 0.64 | 1.28 | 0.090 | 0.185 | 1.000 | 2844 | tags=36%, list=18%, signal=44% |
| 859 | GENTILE\_UV\_RESPONSE\_CLUSTER\_D6 |  | 30 | 0.62 | 1.28 | 0.071 | 0.187 | 1.000 | 2869 | tags=50%, list=18%, signal=61% |
| 860 | PID\_SYNDECAN\_2\_PATHWAY |  | 31 | 0.62 | 1.28 | 0.088 | 0.187 | 1.000 | 2587 | tags=35%, list=16%, signal=42% |
| 861 | REACTOME\_RNA\_POL\_I\_TRANSCRIPTION\_TERMINATION |  | 19 | 0.65 | 1.28 | 0.117 | 0.188 | 1.000 | 2337 | tags=53%, list=15%, signal=62% |
| 862 | SIMBULAN\_PARP1\_TARGETS\_UP |  | 31 | 0.63 | 1.28 | 0.066 | 0.189 | 1.000 | 2645 | tags=55%, list=17%, signal=66% |
| 863 | GRAHAM\_CML\_QUIESCENT\_VS\_NORMAL\_QUIESCENT\_UP |  | 79 | 0.59 | 1.27 | 0.017 | 0.191 | 1.000 | 3006 | tags=39%, list=19%, signal=48% |
| 864 | REACTOME\_TGF\_BETA\_RECEPTOR\_SIGNALING\_ACTIVATES\_SMADS |  | 23 | 0.64 | 1.27 | 0.104 | 0.191 | 1.000 | 4111 | tags=70%, list=26%, signal=94% |
| 865 | ZHAN\_MULTIPLE\_MYELOMA\_CD1\_AND\_CD2\_UP |  | 68 | 0.59 | 1.27 | 0.025 | 0.192 | 1.000 | 3025 | tags=32%, list=19%, signal=40% |
| 866 | LI\_DCP2\_BOUND\_MRNA |  | 80 | 0.59 | 1.27 | 0.026 | 0.192 | 1.000 | 3366 | tags=49%, list=21%, signal=62% |
| 867 | PID\_TRKRPATHWAY |  | 58 | 0.59 | 1.27 | 0.035 | 0.192 | 1.000 | 4031 | tags=48%, list=26%, signal=65% |
| 868 | DURCHDEWALD\_SKIN\_CARCINOGENESIS\_DN |  | 239 | 0.57 | 1.27 | 0.000 | 0.192 | 1.000 | 3295 | tags=38%, list=21%, signal=48% |
| 869 | REACTOME\_ACTIVATION\_OF\_NMDA\_RECEPTOR\_UPON\_GLUTAMATE\_BINDING\_AND\_POSTSYNAPTIC\_EVENTS |  | 37 | 0.61 | 1.27 | 0.070 | 0.193 | 1.000 | 3450 | tags=30%, list=22%, signal=38% |
| 870 | VANTVEER\_BREAST\_CANCER\_ESR1\_DN |  | 211 | 0.57 | 1.27 | 0.000 | 0.193 | 1.000 | 2447 | tags=33%, list=16%, signal=38% |
| 871 | ASTON\_MAJOR\_DEPRESSIVE\_DISORDER\_UP |  | 40 | 0.61 | 1.27 | 0.062 | 0.194 | 1.000 | 1928 | tags=20%, list=12%, signal=23% |
| 872 | REACTOME\_NEPHRIN\_INTERACTIONS |  | 19 | 0.65 | 1.27 | 0.108 | 0.193 | 1.000 | 3899 | tags=47%, list=25%, signal=63% |
| 873 | FERNANDEZ\_BOUND\_BY\_MYC |  | 175 | 0.58 | 1.27 | 0.001 | 0.195 | 1.000 | 2408 | tags=32%, list=15%, signal=37% |
| 874 | WAMUNYOKOLI\_OVARIAN\_CANCER\_GRADES\_1\_2\_DN |  | 56 | 0.59 | 1.27 | 0.039 | 0.195 | 1.000 | 2966 | tags=32%, list=19%, signal=39% |
| 875 | DEURIG\_T\_CELL\_PROLYMPHOCYTIC\_LEUKEMIA\_DN |  | 256 | 0.57 | 1.27 | 0.000 | 0.196 | 1.000 | 4429 | tags=45%, list=28%, signal=61% |
| 876 | YAGI\_AML\_RELAPSE\_PROGNOSIS |  | 31 | 0.61 | 1.27 | 0.084 | 0.196 | 1.000 | 3079 | tags=42%, list=20%, signal=52% |
| 877 | GRESHOCK\_CANCER\_COPY\_NUMBER\_UP |  | 304 | 0.57 | 1.27 | 0.000 | 0.198 | 1.000 | 3752 | tags=42%, list=24%, signal=54% |
| 878 | BROWNE\_HCMV\_INFECTION\_10HR\_DN |  | 51 | 0.60 | 1.27 | 0.053 | 0.198 | 1.000 | 4406 | tags=53%, list=28%, signal=73% |
| 879 | KEGG\_MTOR\_SIGNALING\_PATHWAY |  | 48 | 0.60 | 1.27 | 0.055 | 0.198 | 1.000 | 3066 | tags=40%, list=19%, signal=49% |
| 880 | GAVIN\_FOXP3\_TARGETS\_CLUSTER\_T4 |  | 84 | 0.59 | 1.27 | 0.024 | 0.198 | 1.000 | 3578 | tags=42%, list=23%, signal=54% |
| 881 | PID\_MTOR\_4PATHWAY |  | 63 | 0.59 | 1.27 | 0.036 | 0.201 | 1.000 | 4295 | tags=56%, list=27%, signal=76% |
| 882 | BIOCARTA\_MTOR\_PATHWAY |  | 21 | 0.65 | 1.27 | 0.109 | 0.202 | 1.000 | 3450 | tags=62%, list=22%, signal=79% |
| 883 | LI\_CYTIDINE\_ANALOG\_PATHWAY |  | 16 | 0.66 | 1.27 | 0.141 | 0.202 | 1.000 | 3927 | tags=50%, list=25%, signal=67% |
| 884 | LI\_AMPLIFIED\_IN\_LUNG\_CANCER |  | 170 | 0.57 | 1.27 | 0.006 | 0.204 | 1.000 | 2217 | tags=41%, list=14%, signal=47% |
| 885 | ALCALAY\_AML\_BY\_NPM1\_LOCALIZATION\_DN |  | 170 | 0.57 | 1.27 | 0.003 | 0.205 | 1.000 | 3182 | tags=38%, list=20%, signal=47% |
| 886 | MARSON\_FOXP3\_TARGETS\_STIMULATED\_UP |  | 25 | 0.64 | 1.27 | 0.091 | 0.208 | 1.000 | 2172 | tags=32%, list=14%, signal=37% |
| 887 | BIOCARTA\_VIP\_PATHWAY |  | 25 | 0.63 | 1.26 | 0.092 | 0.209 | 1.000 | 2582 | tags=36%, list=16%, signal=43% |
| 888 | WONG\_IFNA2\_RESISTANCE\_UP |  | 15 | 0.66 | 1.26 | 0.121 | 0.209 | 1.000 | 2858 | tags=40%, list=18%, signal=49% |
| 889 | ZHONG\_SECRETOME\_OF\_LUNG\_CANCER\_AND\_ENDOTHELIUM |  | 64 | 0.59 | 1.26 | 0.040 | 0.209 | 1.000 | 2112 | tags=39%, list=13%, signal=45% |
| 890 | KEGG\_LONG\_TERM\_POTENTIATION |  | 65 | 0.58 | 1.26 | 0.050 | 0.210 | 1.000 | 4729 | tags=46%, list=30%, signal=66% |
| 891 | BIOCARTA\_CHEMICAL\_PATHWAY |  | 21 | 0.65 | 1.26 | 0.092 | 0.210 | 1.000 | 2044 | tags=43%, list=13%, signal=49% |
| 892 | SCHLOSSER\_SERUM\_RESPONSE\_AUGMENTED\_BY\_MYC |  | 94 | 0.58 | 1.26 | 0.013 | 0.211 | 1.000 | 2862 | tags=35%, list=18%, signal=43% |
| 893 | MATZUK\_MEIOTIC\_AND\_DNA\_REPAIR |  | 39 | 0.60 | 1.26 | 0.066 | 0.211 | 1.000 | 4516 | tags=46%, list=29%, signal=65% |
| 894 | WANG\_METHYLATED\_IN\_BREAST\_CANCER |  | 32 | 0.62 | 1.26 | 0.075 | 0.211 | 1.000 | 828 | tags=28%, list=5%, signal=30% |
| 895 | KAN\_RESPONSE\_TO\_ARSENIC\_TRIOXIDE |  | 105 | 0.57 | 1.26 | 0.013 | 0.211 | 1.000 | 2977 | tags=34%, list=19%, signal=42% |
| 896 | BYSTROEM\_CORRELATED\_WITH\_IL5\_UP |  | 51 | 0.60 | 1.26 | 0.060 | 0.212 | 1.000 | 3760 | tags=45%, list=24%, signal=59% |
| 897 | BIOCARTA\_TEL\_PATHWAY |  | 18 | 0.66 | 1.26 | 0.114 | 0.212 | 1.000 | 4429 | tags=78%, list=28%, signal=108% |
| 898 | AIYAR\_COBRA1\_TARGETS\_UP |  | 36 | 0.60 | 1.26 | 0.071 | 0.211 | 1.000 | 759 | tags=19%, list=5%, signal=20% |
| 899 | REACTOME\_RNA\_POL\_III\_TRANSCRIPTION\_INITIATION\_FROM\_TYPE\_3\_PROMOTER |  | 26 | 0.62 | 1.26 | 0.104 | 0.212 | 1.000 | 5607 | tags=81%, list=36%, signal=125% |
| 900 | CUI\_GLUCOSE\_DEPRIVATION |  | 56 | 0.59 | 1.26 | 0.054 | 0.212 | 1.000 | 2063 | tags=36%, list=13%, signal=41% |
| 901 | BENPORATH\_OCT4\_TARGETS |  | 260 | 0.57 | 1.26 | 0.002 | 0.212 | 1.000 | 3594 | tags=39%, list=23%, signal=50% |
| 902 | GUTIERREZ\_MULTIPLE\_MYELOMA\_DN |  | 30 | 0.62 | 1.26 | 0.079 | 0.213 | 1.000 | 4186 | tags=70%, list=27%, signal=95% |
| 903 | PID\_RAC1\_REG\_PATHWAY |  | 36 | 0.61 | 1.26 | 0.077 | 0.213 | 1.000 | 2118 | tags=28%, list=13%, signal=32% |
| 904 | HASLINGER\_B\_CLL\_WITH\_CHROMOSOME\_12\_TRISOMY |  | 22 | 0.64 | 1.26 | 0.103 | 0.213 | 1.000 | 3754 | tags=59%, list=24%, signal=77% |
| 905 | GOTZMANN\_EPITHELIAL\_TO\_MESENCHYMAL\_TRANSITION\_DN |  | 200 | 0.56 | 1.26 | 0.002 | 0.218 | 1.000 | 2586 | tags=38%, list=16%, signal=45% |
| 906 | TONG\_INTERACT\_WITH\_PTTG1 |  | 42 | 0.60 | 1.26 | 0.064 | 0.218 | 1.000 | 2931 | tags=43%, list=19%, signal=53% |
| 907 | PID\_SMAD2\_3PATHWAY |  | 17 | 0.66 | 1.26 | 0.109 | 0.220 | 1.000 | 3865 | tags=59%, list=25%, signal=78% |
| 908 | NGUYEN\_NOTCH1\_TARGETS\_UP |  | 28 | 0.62 | 1.26 | 0.096 | 0.221 | 1.000 | 2844 | tags=39%, list=18%, signal=48% |
| 909 | KYNG\_WERNER\_SYNDROM\_AND\_NORMAL\_AGING\_DN |  | 190 | 0.57 | 1.26 | 0.005 | 0.221 | 1.000 | 3239 | tags=37%, list=21%, signal=46% |
| 910 | SCHAEFFER\_PROSTATE\_DEVELOPMENT\_6HR\_DN |  | 453 | 0.56 | 1.26 | 0.000 | 0.221 | 1.000 | 3292 | tags=40%, list=21%, signal=49% |
| 911 | BIOCARTA\_CDC42RAC\_PATHWAY |  | 16 | 0.65 | 1.26 | 0.130 | 0.222 | 1.000 | 1307 | tags=38%, list=8%, signal=41% |
| 912 | REACTOME\_CTLA4\_INHIBITORY\_SIGNALING |  | 21 | 0.64 | 1.26 | 0.109 | 0.224 | 1.000 | 4124 | tags=57%, list=26%, signal=77% |
| 913 | DACOSTA\_UV\_RESPONSE\_VIA\_ERCC3\_TTD\_DN |  | 69 | 0.58 | 1.26 | 0.043 | 0.224 | 1.000 | 4399 | tags=52%, list=28%, signal=72% |
| 914 | IVANOVA\_HEMATOPOIESIS\_LATE\_PROGENITOR |  | 444 | 0.56 | 1.26 | 0.000 | 0.224 | 1.000 | 3458 | tags=39%, list=22%, signal=49% |
| 915 | HAHTOLA\_MYCOSIS\_FUNGOIDES\_SKIN\_UP |  | 155 | 0.57 | 1.26 | 0.005 | 0.226 | 1.000 | 3979 | tags=51%, list=25%, signal=68% |
| 916 | MCDOWELL\_ACUTE\_LUNG\_INJURY\_UP |  | 44 | 0.60 | 1.26 | 0.071 | 0.226 | 1.000 | 2165 | tags=32%, list=14%, signal=37% |
| 917 | BIOCARTA\_VEGF\_PATHWAY |  | 28 | 0.62 | 1.26 | 0.108 | 0.226 | 1.000 | 4475 | tags=61%, list=28%, signal=85% |
| 918 | KEGG\_GLIOMA |  | 61 | 0.58 | 1.25 | 0.048 | 0.228 | 1.000 | 5031 | tags=54%, list=32%, signal=79% |
| 919 | HOLLEMAN\_PREDNISOLONE\_RESISTANCE\_ALL\_DN |  | 17 | 0.65 | 1.25 | 0.135 | 0.228 | 1.000 | 4087 | tags=65%, list=26%, signal=87% |
| 920 | REACTOME\_P130CAS\_LINKAGE\_TO\_MAPK\_SIGNALING\_FOR\_INTEGRINS |  | 15 | 0.66 | 1.25 | 0.136 | 0.228 | 1.000 | 3891 | tags=47%, list=25%, signal=62% |
| 921 | BOYLAN\_MULTIPLE\_MYELOMA\_C\_CLUSTER\_UP |  | 32 | 0.61 | 1.25 | 0.101 | 0.228 | 1.000 | 3595 | tags=50%, list=23%, signal=65% |
| 922 | HEIDENBLAD\_AMPLICON\_8Q24\_UP |  | 29 | 0.62 | 1.25 | 0.094 | 0.229 | 1.000 | 2820 | tags=41%, list=18%, signal=50% |
| 923 | WANG\_RESPONSE\_TO\_BEXAROTENE\_UP |  | 31 | 0.62 | 1.25 | 0.109 | 0.228 | 1.000 | 1632 | tags=23%, list=10%, signal=25% |
| 924 | REACTOME\_POST\_NMDA\_RECEPTOR\_ACTIVATION\_EVENTS |  | 33 | 0.61 | 1.25 | 0.089 | 0.229 | 1.000 | 3450 | tags=33%, list=22%, signal=43% |
| 925 | GEISS\_RESPONSE\_TO\_DSRNA\_UP |  | 36 | 0.59 | 1.25 | 0.090 | 0.229 | 1.000 | 1998 | tags=33%, list=13%, signal=38% |
| 926 | SHEPARD\_CRUSH\_AND\_BURN\_MUTANT\_UP |  | 170 | 0.57 | 1.25 | 0.002 | 0.230 | 1.000 | 3172 | tags=41%, list=20%, signal=50% |
| 927 | CONCANNON\_APOPTOSIS\_BY\_EPOXOMICIN\_DN |  | 148 | 0.56 | 1.25 | 0.002 | 0.232 | 1.000 | 3031 | tags=33%, list=19%, signal=41% |
| 928 | ZHU\_CMV\_8\_HR\_UP |  | 40 | 0.59 | 1.25 | 0.094 | 0.233 | 1.000 | 3811 | tags=48%, list=24%, signal=63% |
| 929 | LINDSTEDT\_DENDRITIC\_CELL\_MATURATION\_A |  | 60 | 0.59 | 1.25 | 0.047 | 0.233 | 1.000 | 1688 | tags=20%, list=11%, signal=22% |
| 930 | LIN\_MELANOMA\_COPY\_NUMBER\_DN |  | 35 | 0.60 | 1.25 | 0.095 | 0.234 | 1.000 | 4675 | tags=71%, list=30%, signal=101% |
| 931 | BIOCARTA\_TNFR1\_PATHWAY |  | 29 | 0.62 | 1.25 | 0.101 | 0.234 | 1.000 | 3630 | tags=45%, list=23%, signal=58% |
| 932 | PURBEY\_TARGETS\_OF\_CTBP1\_AND\_SATB1\_UP |  | 73 | 0.58 | 1.25 | 0.039 | 0.235 | 1.000 | 2116 | tags=33%, list=13%, signal=38% |
| 933 | GARGALOVIC\_RESPONSE\_TO\_OXIDIZED\_PHOSPHOLIPIDS\_BLUE\_UP |  | 108 | 0.57 | 1.25 | 0.019 | 0.235 | 1.000 | 3556 | tags=42%, list=23%, signal=53% |
| 934 | GRUETZMANN\_PANCREATIC\_CANCER\_UP |  | 325 | 0.56 | 1.25 | 0.000 | 0.234 | 1.000 | 2895 | tags=38%, list=18%, signal=45% |
| 935 | STAMBOLSKY\_BOUND\_BY\_MUTATED\_TP53 |  | 15 | 0.67 | 1.25 | 0.132 | 0.235 | 1.000 | 2682 | tags=33%, list=17%, signal=40% |
| 936 | PID\_P73PATHWAY |  | 71 | 0.58 | 1.25 | 0.038 | 0.235 | 1.000 | 4553 | tags=49%, list=29%, signal=69% |
| 937 | PENG\_GLUTAMINE\_DEPRIVATION\_UP |  | 36 | 0.60 | 1.25 | 0.097 | 0.236 | 1.000 | 1588 | tags=28%, list=10%, signal=31% |
| 938 | TOMLINS\_PROSTATE\_CANCER\_UP |  | 36 | 0.61 | 1.25 | 0.078 | 0.235 | 1.000 | 1893 | tags=39%, list=12%, signal=44% |
| 939 | WANG\_SMARCE1\_TARGETS\_DN |  | 307 | 0.56 | 1.25 | 0.000 | 0.236 | 1.000 | 4086 | tags=47%, list=26%, signal=62% |
| 940 | GALLUZZI\_PERMEABILIZE\_MITOCHONDRIA |  | 42 | 0.59 | 1.25 | 0.083 | 0.238 | 1.000 | 4429 | tags=50%, list=28%, signal=69% |
| 941 | BIOCARTA\_G2\_PATHWAY |  | 23 | 0.63 | 1.25 | 0.110 | 0.239 | 1.000 | 4281 | tags=74%, list=27%, signal=101% |
| 942 | BIOCARTA\_IGF1MTOR\_PATHWAY |  | 19 | 0.64 | 1.25 | 0.143 | 0.239 | 1.000 | 4113 | tags=74%, list=26%, signal=100% |
| 943 | ADDYA\_ERYTHROID\_DIFFERENTIATION\_BY\_HEMIN |  | 65 | 0.57 | 1.25 | 0.046 | 0.240 | 1.000 | 3032 | tags=31%, list=19%, signal=38% |
| 944 | CHANDRAN\_METASTASIS\_UP |  | 169 | 0.56 | 1.25 | 0.001 | 0.240 | 1.000 | 4113 | tags=47%, list=26%, signal=63% |
| 945 | GYORFFY\_MITOXANTRONE\_RESISTANCE |  | 47 | 0.59 | 1.25 | 0.075 | 0.240 | 1.000 | 3667 | tags=34%, list=23%, signal=44% |
| 946 | BIOCARTA\_NFAT\_PATHWAY |  | 51 | 0.58 | 1.25 | 0.073 | 0.240 | 1.000 | 4466 | tags=45%, list=28%, signal=63% |
| 947 | MATZUK\_SPERMATOCYTE |  | 69 | 0.58 | 1.25 | 0.042 | 0.240 | 1.000 | 4429 | tags=46%, list=28%, signal=64% |
| 948 | SCHAEFFER\_PROSTATE\_DEVELOPMENT\_6HR\_UP |  | 155 | 0.56 | 1.25 | 0.006 | 0.240 | 1.000 | 3533 | tags=38%, list=22%, signal=49% |
| 949 | MARZEC\_IL2\_SIGNALING\_UP |  | 107 | 0.57 | 1.25 | 0.018 | 0.241 | 1.000 | 3960 | tags=42%, list=25%, signal=56% |
| 950 | MCCLUNG\_COCAIN\_REWARD\_4WK |  | 72 | 0.57 | 1.25 | 0.049 | 0.241 | 1.000 | 4034 | tags=36%, list=26%, signal=48% |
| 951 | ROSS\_LEUKEMIA\_WITH\_MLL\_FUSIONS |  | 69 | 0.58 | 1.25 | 0.050 | 0.241 | 1.000 | 1424 | tags=25%, list=9%, signal=27% |
| 952 | CHEN\_PDGF\_TARGETS |  | 17 | 0.65 | 1.25 | 0.116 | 0.241 | 1.000 | 4378 | tags=65%, list=28%, signal=90% |
| 953 | REACTOME\_AMINO\_ACID\_TRANSPORT\_ACROSS\_THE\_PLASMA\_MEMBRANE |  | 30 | 0.61 | 1.25 | 0.107 | 0.241 | 1.000 | 3312 | tags=30%, list=21%, signal=38% |
| 954 | BIOCARTA\_ATM\_PATHWAY |  | 20 | 0.63 | 1.25 | 0.138 | 0.242 | 1.000 | 4360 | tags=70%, list=28%, signal=97% |
| 955 | ONKEN\_UVEAL\_MELANOMA\_DN |  | 469 | 0.55 | 1.25 | 0.000 | 0.242 | 1.000 | 3457 | tags=37%, list=22%, signal=46% |
| 956 | LEE\_AGING\_NEOCORTEX\_UP |  | 85 | 0.57 | 1.25 | 0.039 | 0.242 | 1.000 | 1817 | tags=29%, list=12%, signal=33% |
| 957 | PID\_NFKAPPABCANONICALPATHWAY |  | 23 | 0.62 | 1.24 | 0.120 | 0.246 | 1.000 | 4037 | tags=52%, list=26%, signal=70% |
| 958 | BROWNE\_HCMV\_INFECTION\_8HR\_UP |  | 89 | 0.57 | 1.24 | 0.028 | 0.246 | 1.000 | 2925 | tags=31%, list=19%, signal=38% |
| 959 | GROSS\_HYPOXIA\_VIA\_ELK3\_UP |  | 189 | 0.56 | 1.24 | 0.001 | 0.246 | 1.000 | 3090 | tags=42%, list=20%, signal=52% |
| 960 | HILLION\_HMGA1B\_TARGETS |  | 89 | 0.57 | 1.24 | 0.035 | 0.246 | 1.000 | 3257 | tags=42%, list=21%, signal=52% |
| 961 | SMIRNOV\_RESPONSE\_TO\_IR\_2HR\_UP |  | 42 | 0.59 | 1.24 | 0.092 | 0.247 | 1.000 | 3621 | tags=43%, list=23%, signal=56% |
| 962 | PID\_ILK\_PATHWAY |  | 45 | 0.59 | 1.24 | 0.099 | 0.248 | 1.000 | 3674 | tags=60%, list=23%, signal=78% |
| 963 | PID\_IL2\_STAT5PATHWAY |  | 30 | 0.61 | 1.24 | 0.120 | 0.248 | 1.000 | 3328 | tags=33%, list=21%, signal=42% |
| 964 | IVANOVA\_HEMATOPOIESIS\_STEM\_CELL\_SHORT\_TERM |  | 27 | 0.62 | 1.24 | 0.117 | 0.248 | 1.000 | 4079 | tags=59%, list=26%, signal=80% |
| 965 | ZHAN\_V1\_LATE\_DIFFERENTIATION\_GENES\_DN |  | 15 | 0.65 | 1.24 | 0.154 | 0.248 | 1.000 | 2736 | tags=47%, list=17%, signal=56% |
| 966 | PICCALUGA\_ANGIOIMMUNOBLASTIC\_LYMPHOMA\_DN |  | 111 | 0.57 | 1.24 | 0.020 | 0.248 | 1.000 | 4671 | tags=50%, list=30%, signal=71% |
| 967 | CHARAFE\_BREAST\_CANCER\_LUMINAL\_VS\_BASAL\_DN |  | 392 | 0.55 | 1.24 | 0.000 | 0.248 | 1.000 | 3242 | tags=37%, list=21%, signal=45% |
| 968 | RIGGINS\_TAMOXIFEN\_RESISTANCE\_DN |  | 193 | 0.56 | 1.24 | 0.002 | 0.248 | 1.000 | 4208 | tags=44%, list=27%, signal=59% |
| 969 | REACTOME\_CREB\_PHOSPHORYLATION\_THROUGH\_THE\_ACTIVATION\_OF\_RAS |  | 27 | 0.61 | 1.24 | 0.117 | 0.248 | 1.000 | 3450 | tags=37%, list=22%, signal=47% |
| 970 | REACTOME\_MEIOSIS |  | 83 | 0.57 | 1.24 | 0.031 | 0.248 | 1.000 | 3215 | tags=35%, list=20%, signal=44% |
| 971 | BROWNE\_HCMV\_INFECTION\_20HR\_DN |  | 93 | 0.57 | 1.24 | 0.027 | 0.248 | 1.000 | 3323 | tags=40%, list=21%, signal=50% |
| 972 | MOSERLE\_IFNA\_RESPONSE |  | 22 | 0.62 | 1.24 | 0.131 | 0.249 | 1.000 | 3036 | tags=36%, list=19%, signal=45% |
| 973 | REACTOME\_ADAPTIVE\_IMMUNE\_SYSTEM |  | 467 | 0.55 | 1.24 | 0.000 | 0.249 | 1.000 | 3073 | tags=34%, list=20%, signal=41% |
| 974 | MIDORIKAWA\_AMPLIFIED\_IN\_LIVER\_CANCER |  | 40 | 0.59 | 1.24 | 0.079 | 0.249 | 1.000 | 3431 | tags=40%, list=22%, signal=51% |
| 975 | PODAR\_RESPONSE\_TO\_ADAPHOSTIN\_UP |  | 129 | 0.56 | 1.24 | 0.013 | 0.251 | 1.000 | 3462 | tags=39%, list=22%, signal=49% |
| 976 | FLECHNER\_PBL\_KIDNEY\_TRANSPLANT\_REJECTED\_VS\_OK\_UP |  | 59 | 0.58 | 1.24 | 0.060 | 0.252 | 1.000 | 2427 | tags=41%, list=15%, signal=48% |
| 977 | ZHANG\_INTERFERON\_RESPONSE |  | 20 | 0.63 | 1.24 | 0.141 | 0.253 | 1.000 | 3036 | tags=45%, list=19%, signal=56% |
| 978 | CLASPER\_LYMPHATIC\_VESSELS\_DURING\_METASTASIS\_DN |  | 33 | 0.60 | 1.24 | 0.088 | 0.252 | 1.000 | 1357 | tags=27%, list=9%, signal=30% |
| 979 | HELLER\_HDAC\_TARGETS\_SILENCED\_BY\_METHYLATION\_DN |  | 242 | 0.56 | 1.24 | 0.000 | 0.252 | 1.000 | 2386 | tags=31%, list=15%, signal=35% |
| 980 | PID\_FAK\_PATHWAY |  | 57 | 0.58 | 1.24 | 0.070 | 0.252 | 1.000 | 3899 | tags=47%, list=25%, signal=63% |
| 981 | MAHAJAN\_RESPONSE\_TO\_IL1A\_DN |  | 73 | 0.58 | 1.24 | 0.047 | 0.252 | 1.000 | 4396 | tags=51%, list=28%, signal=70% |
| 982 | WINTER\_HYPOXIA\_UP |  | 78 | 0.57 | 1.24 | 0.050 | 0.252 | 1.000 | 2645 | tags=36%, list=17%, signal=43% |
| 983 | LANDIS\_ERBB2\_BREAST\_TUMORS\_65\_UP |  | 20 | 0.63 | 1.24 | 0.136 | 0.252 | 1.000 | 2009 | tags=30%, list=13%, signal=34% |
| 984 | ALCALA\_APOPTOSIS |  | 79 | 0.57 | 1.24 | 0.040 | 0.253 | 1.000 | 3176 | tags=35%, list=20%, signal=44% |
| 985 | JAZAERI\_BREAST\_CANCER\_BRCA1\_VS\_BRCA2\_UP |  | 43 | 0.59 | 1.24 | 0.079 | 0.254 | 1.000 | 2643 | tags=40%, list=17%, signal=47% |
| 986 | ZHENG\_BOUND\_BY\_FOXP3 |  | 427 | 0.55 | 1.24 | 0.000 | 0.254 | 1.000 | 4598 | tags=39%, list=29%, signal=54% |
| 987 | HUANG\_DASATINIB\_RESISTANCE\_UP |  | 75 | 0.57 | 1.24 | 0.056 | 0.255 | 1.000 | 2389 | tags=33%, list=15%, signal=39% |
| 988 | NUNODA\_RESPONSE\_TO\_DASATINIB\_IMATINIB\_UP |  | 28 | 0.61 | 1.24 | 0.122 | 0.256 | 1.000 | 3673 | tags=54%, list=23%, signal=70% |
| 989 | PID\_CASPASE\_PATHWAY |  | 49 | 0.58 | 1.24 | 0.083 | 0.256 | 1.000 | 2112 | tags=29%, list=13%, signal=33% |
| 990 | POOLA\_INVASIVE\_BREAST\_CANCER\_UP |  | 247 | 0.55 | 1.24 | 0.004 | 0.258 | 1.000 | 3202 | tags=26%, list=20%, signal=32% |
| 991 | ELVIDGE\_HYPOXIA\_DN |  | 129 | 0.56 | 1.24 | 0.019 | 0.258 | 1.000 | 2681 | tags=38%, list=17%, signal=45% |
| 992 | MYLLYKANGAS\_AMPLIFICATION\_HOT\_SPOT\_17 |  | 17 | 0.64 | 1.24 | 0.157 | 0.258 | 1.000 | 3960 | tags=53%, list=25%, signal=71% |
| 993 | BROWNE\_HCMV\_INFECTION\_4HR\_UP |  | 50 | 0.57 | 1.24 | 0.087 | 0.261 | 1.000 | 3378 | tags=32%, list=21%, signal=41% |
| 994 | YAO\_TEMPORAL\_RESPONSE\_TO\_PROGESTERONE\_CLUSTER\_2 |  | 76 | 0.57 | 1.23 | 0.049 | 0.262 | 1.000 | 1846 | tags=24%, list=12%, signal=27% |
| 995 | DORSAM\_HOXA9\_TARGETS\_DN |  | 31 | 0.59 | 1.23 | 0.125 | 0.262 | 1.000 | 1681 | tags=35%, list=11%, signal=40% |
| 996 | KORKOLA\_SEMINOMA\_UP |  | 36 | 0.59 | 1.23 | 0.127 | 0.262 | 1.000 | 4358 | tags=53%, list=28%, signal=73% |
| 997 | BREDEMEYER\_RAG\_SIGNALING\_NOT\_VIA\_ATM\_UP |  | 44 | 0.59 | 1.23 | 0.089 | 0.262 | 1.000 | 3416 | tags=34%, list=22%, signal=43% |
| 998 | HUANG\_GATA2\_TARGETS\_DN |  | 67 | 0.57 | 1.23 | 0.068 | 0.262 | 1.000 | 3307 | tags=43%, list=21%, signal=55% |
| 999 | RICKMAN\_TUMOR\_DIFFERENTIATED\_WELL\_VS\_POORLY\_UP |  | 198 | 0.56 | 1.23 | 0.003 | 0.262 | 1.000 | 4352 | tags=47%, list=28%, signal=65% |
| 1000 | REACTOME\_RNA\_POL\_I\_TRANSCRIPTION\_INITIATION |  | 21 | 0.63 | 1.23 | 0.144 | 0.262 | 1.000 | 2337 | tags=48%, list=15%, signal=56% |
| 1001 | HOWLIN\_CITED1\_TARGETS\_1\_UP |  | 29 | 0.61 | 1.23 | 0.117 | 0.262 | 1.000 | 2637 | tags=34%, list=17%, signal=41% |
| 1002 | IVANOVA\_HEMATOPOIESIS\_EARLY\_PROGENITOR |  | 438 | 0.55 | 1.23 | 0.000 | 0.263 | 1.000 | 4445 | tags=45%, list=28%, signal=62% |
| 1003 | KARAKAS\_TGFB1\_SIGNALING |  | 17 | 0.65 | 1.23 | 0.146 | 0.263 | 1.000 | 2953 | tags=47%, list=19%, signal=58% |
| 1004 | DARWICHE\_PAPILLOMA\_RISK\_LOW\_UP |  | 137 | 0.56 | 1.23 | 0.019 | 0.264 | 1.000 | 3985 | tags=38%, list=25%, signal=50% |
| 1005 | BIOCARTA\_EIF4\_PATHWAY |  | 22 | 0.63 | 1.23 | 0.159 | 0.264 | 1.000 | 4395 | tags=82%, list=28%, signal=113% |
| 1006 | WATANABE\_COLON\_CANCER\_MSI\_VS\_MSS\_UP |  | 24 | 0.61 | 1.23 | 0.143 | 0.265 | 1.000 | 2092 | tags=29%, list=13%, signal=34% |
| 1007 | REACTOME\_OXYGEN\_DEPENDENT\_PROLINE\_HYDROXYLATION\_OF\_HYPOXIA\_INDUCIBLE\_FACTOR\_ALPHA |  | 16 | 0.64 | 1.23 | 0.149 | 0.265 | 1.000 | 2005 | tags=44%, list=13%, signal=50% |
| 1008 | LEE\_AGING\_NEOCORTEX\_DN |  | 74 | 0.56 | 1.23 | 0.054 | 0.267 | 1.000 | 3566 | tags=38%, list=23%, signal=49% |
| 1009 | KIM\_GERMINAL\_CENTER\_T\_HELPER\_DN |  | 16 | 0.65 | 1.23 | 0.173 | 0.267 | 1.000 | 3014 | tags=38%, list=19%, signal=46% |
| 1010 | REACTOME\_NOTCH1\_INTRACELLULAR\_DOMAIN\_REGULATES\_TRANSCRIPTION |  | 39 | 0.59 | 1.23 | 0.097 | 0.267 | 1.000 | 1780 | tags=31%, list=11%, signal=35% |
| 1011 | CHUANG\_OXIDATIVE\_STRESS\_RESPONSE\_UP |  | 24 | 0.61 | 1.23 | 0.153 | 0.268 | 1.000 | 2170 | tags=29%, list=14%, signal=34% |
| 1012 | SHEPARD\_BMYB\_MORPHOLINO\_UP |  | 181 | 0.55 | 1.23 | 0.010 | 0.268 | 1.000 | 3296 | tags=38%, list=21%, signal=47% |
| 1013 | NAGY\_TFTC\_COMPONENTS\_HUMAN |  | 16 | 0.65 | 1.23 | 0.162 | 0.269 | 1.000 | 4087 | tags=63%, list=26%, signal=84% |
| 1014 | WALLACE\_JAK2\_TARGETS\_UP |  | 23 | 0.63 | 1.23 | 0.133 | 0.270 | 1.000 | 2239 | tags=43%, list=14%, signal=51% |
| 1015 | MARTINEZ\_RESPONSE\_TO\_TRABECTEDIN |  | 47 | 0.58 | 1.23 | 0.096 | 0.270 | 1.000 | 4738 | tags=68%, list=30%, signal=97% |
| 1016 | KRIEG\_KDM3A\_TARGETS\_NOT\_HYPOXIA |  | 161 | 0.56 | 1.23 | 0.017 | 0.270 | 1.000 | 4037 | tags=46%, list=26%, signal=61% |
| 1017 | MEINHOLD\_OVARIAN\_CANCER\_LOW\_GRADE\_DN |  | 20 | 0.62 | 1.23 | 0.153 | 0.270 | 1.000 | 2049 | tags=45%, list=13%, signal=52% |
| 1018 | YAO\_TEMPORAL\_RESPONSE\_TO\_PROGESTERONE\_CLUSTER\_7 |  | 67 | 0.57 | 1.23 | 0.055 | 0.270 | 1.000 | 2891 | tags=40%, list=18%, signal=49% |
| 1019 | PASINI\_SUZ12\_TARGETS\_DN |  | 300 | 0.55 | 1.23 | 0.002 | 0.270 | 1.000 | 2977 | tags=38%, list=19%, signal=46% |
| 1020 | COLINA\_TARGETS\_OF\_4EBP1\_AND\_4EBP2 |  | 334 | 0.55 | 1.23 | 0.000 | 0.270 | 1.000 | 3216 | tags=37%, list=20%, signal=46% |
| 1021 | VANASSE\_BCL2\_TARGETS\_DN |  | 64 | 0.57 | 1.23 | 0.059 | 0.270 | 1.000 | 2577 | tags=27%, list=16%, signal=32% |
| 1022 | KAYO\_AGING\_MUSCLE\_DN |  | 117 | 0.56 | 1.23 | 0.027 | 0.270 | 1.000 | 3478 | tags=47%, list=22%, signal=60% |
| 1023 | PID\_ERBB1\_INTERNALIZATION\_PATHWAY |  | 40 | 0.58 | 1.23 | 0.115 | 0.270 | 1.000 | 3435 | tags=43%, list=22%, signal=54% |
| 1024 | DORN\_ADENOVIRUS\_INFECTION\_32HR\_DN |  | 34 | 0.59 | 1.23 | 0.128 | 0.270 | 1.000 | 2839 | tags=47%, list=18%, signal=57% |
| 1025 | CHESLER\_BRAIN\_HIGHEST\_GENETIC\_VARIANCE |  | 32 | 0.60 | 1.23 | 0.121 | 0.270 | 1.000 | 3457 | tags=44%, list=22%, signal=56% |
| 1026 | IKEDA\_MIR30\_TARGETS\_DN |  | 25 | 0.61 | 1.23 | 0.147 | 0.270 | 1.000 | 4070 | tags=36%, list=26%, signal=48% |
| 1027 | KORKOLA\_EMBRYONAL\_CARCINOMA\_UP |  | 35 | 0.60 | 1.23 | 0.123 | 0.270 | 1.000 | 4222 | tags=49%, list=27%, signal=66% |
| 1028 | REACTOME\_PI3K\_AKT\_ACTIVATION |  | 32 | 0.59 | 1.23 | 0.131 | 0.270 | 1.000 | 4197 | tags=50%, list=27%, signal=68% |
| 1029 | BURTON\_ADIPOGENESIS\_9 |  | 83 | 0.56 | 1.23 | 0.042 | 0.270 | 1.000 | 2669 | tags=39%, list=17%, signal=46% |
| 1030 | BRUECKNER\_TARGETS\_OF\_MIRLET7A3\_DN |  | 67 | 0.57 | 1.23 | 0.053 | 0.271 | 1.000 | 2953 | tags=36%, list=19%, signal=44% |
| 1031 | LEE\_LIVER\_CANCER\_MYC\_E2F1\_UP |  | 54 | 0.57 | 1.23 | 0.089 | 0.271 | 1.000 | 3036 | tags=35%, list=19%, signal=43% |
| 1032 | REACTOME\_RECYCLING\_PATHWAY\_OF\_L1 |  | 26 | 0.61 | 1.23 | 0.152 | 0.272 | 1.000 | 3119 | tags=42%, list=20%, signal=53% |
| 1033 | IGLESIAS\_E2F\_TARGETS\_UP |  | 143 | 0.55 | 1.23 | 0.017 | 0.274 | 1.000 | 2133 | tags=36%, list=14%, signal=41% |
| 1034 | KONDO\_COLON\_CANCER\_HCP\_WITH\_H3K27ME1 |  | 21 | 0.63 | 1.23 | 0.144 | 0.274 | 1.000 | 3826 | tags=43%, list=24%, signal=57% |
| 1035 | GROSS\_ELK3\_TARGETS\_DN |  | 29 | 0.60 | 1.23 | 0.144 | 0.277 | 1.000 | 788 | tags=21%, list=5%, signal=22% |
| 1036 | ABE\_VEGFA\_TARGETS\_30MIN |  | 27 | 0.60 | 1.23 | 0.140 | 0.277 | 1.000 | 2668 | tags=30%, list=17%, signal=36% |
| 1037 | NIKOLSKY\_BREAST\_CANCER\_8P12\_P11\_AMPLICON |  | 48 | 0.58 | 1.22 | 0.088 | 0.280 | 1.000 | 3794 | tags=38%, list=24%, signal=49% |
| 1038 | JAZAG\_TGFB1\_SIGNALING\_DN |  | 30 | 0.60 | 1.22 | 0.143 | 0.281 | 1.000 | 419 | tags=13%, list=3%, signal=14% |
| 1039 | KEGG\_APOPTOSIS |  | 78 | 0.56 | 1.22 | 0.062 | 0.281 | 1.000 | 4226 | tags=41%, list=27%, signal=56% |
| 1040 | BIOCARTA\_MCALPAIN\_PATHWAY |  | 23 | 0.62 | 1.22 | 0.148 | 0.281 | 1.000 | 2587 | tags=30%, list=16%, signal=36% |
| 1041 | BROWNE\_HCMV\_INFECTION\_48HR\_UP |  | 160 | 0.55 | 1.22 | 0.015 | 0.281 | 1.000 | 3562 | tags=40%, list=23%, signal=51% |
| 1042 | REACTOME\_APOPTOTIC\_CLEAVAGE\_OF\_CELLULAR\_PROTEINS |  | 36 | 0.59 | 1.22 | 0.115 | 0.282 | 1.000 | 2223 | tags=36%, list=14%, signal=42% |
| 1043 | LOCKWOOD\_AMPLIFIED\_IN\_LUNG\_CANCER |  | 184 | 0.55 | 1.22 | 0.014 | 0.282 | 1.000 | 4519 | tags=56%, list=29%, signal=78% |
| 1044 | MARTORIATI\_MDM4\_TARGETS\_NEUROEPITHELIUM\_UP |  | 150 | 0.55 | 1.22 | 0.014 | 0.282 | 1.000 | 3349 | tags=32%, list=21%, signal=40% |
| 1045 | SCHMIDT\_POR\_TARGETS\_IN\_LIMB\_BUD\_UP |  | 25 | 0.60 | 1.22 | 0.148 | 0.282 | 1.000 | 2187 | tags=32%, list=14%, signal=37% |
| 1046 | MAYBURD\_RESPONSE\_TO\_L663536\_UP |  | 25 | 0.61 | 1.22 | 0.172 | 0.282 | 1.000 | 3451 | tags=44%, list=22%, signal=56% |
| 1047 | RODRIGUES\_THYROID\_CARCINOMA\_DN |  | 65 | 0.57 | 1.22 | 0.080 | 0.283 | 1.000 | 4073 | tags=40%, list=26%, signal=54% |
| 1048 | WU\_HBX\_TARGETS\_3\_UP |  | 18 | 0.64 | 1.22 | 0.159 | 0.283 | 1.000 | 3182 | tags=44%, list=20%, signal=56% |
| 1049 | WAKABAYASHI\_ADIPOGENESIS\_PPARG\_BOUND\_36HR |  | 27 | 0.61 | 1.22 | 0.154 | 0.283 | 1.000 | 2564 | tags=30%, list=16%, signal=35% |
| 1050 | REACTOME\_INSULIN\_RECEPTOR\_RECYCLING |  | 23 | 0.62 | 1.22 | 0.148 | 0.284 | 1.000 | 2361 | tags=26%, list=15%, signal=31% |
| 1051 | MULLIGHAN\_NPM1\_MUTATED\_SIGNATURE\_1\_UP |  | 234 | 0.55 | 1.22 | 0.002 | 0.284 | 1.000 | 4345 | tags=41%, list=28%, signal=56% |
| 1052 | KEGG\_ETHER\_LIPID\_METABOLISM |  | 28 | 0.60 | 1.22 | 0.134 | 0.284 | 1.000 | 1175 | tags=21%, list=7%, signal=23% |
| 1053 | MARTINEZ\_RESPONSE\_TO\_TRABECTEDIN\_UP |  | 63 | 0.57 | 1.22 | 0.084 | 0.284 | 1.000 | 3110 | tags=43%, list=20%, signal=53% |
| 1054 | PID\_PI3KCIAKTPATHWAY |  | 33 | 0.59 | 1.22 | 0.135 | 0.284 | 1.000 | 2215 | tags=36%, list=14%, signal=42% |
| 1055 | GAUSSMANN\_MLL\_AF4\_FUSION\_TARGETS\_G\_DN |  | 32 | 0.59 | 1.22 | 0.140 | 0.285 | 1.000 | 4925 | tags=53%, list=31%, signal=77% |
| 1056 | KYNG\_DNA\_DAMAGE\_DN |  | 178 | 0.55 | 1.22 | 0.010 | 0.287 | 1.000 | 2947 | tags=35%, list=19%, signal=42% |
| 1057 | MULLIGHAN\_NPM1\_SIGNATURE\_3\_UP |  | 288 | 0.55 | 1.22 | 0.001 | 0.287 | 1.000 | 4345 | tags=41%, list=28%, signal=56% |
| 1058 | BENPORATH\_NOS\_TARGETS |  | 158 | 0.55 | 1.22 | 0.016 | 0.287 | 1.000 | 3283 | tags=33%, list=21%, signal=41% |
| 1059 | JIANG\_AGING\_HYPOTHALAMUS\_UP |  | 42 | 0.58 | 1.22 | 0.109 | 0.287 | 1.000 | 3003 | tags=55%, list=19%, signal=67% |
| 1060 | CHEOK\_RESPONSE\_TO\_MERCAPTOPURINE\_AND\_HD\_MTX\_DN |  | 22 | 0.61 | 1.22 | 0.162 | 0.287 | 1.000 | 4190 | tags=55%, list=27%, signal=74% |
| 1061 | ROZANOV\_MMP14\_TARGETS\_SUBSET |  | 32 | 0.59 | 1.22 | 0.137 | 0.288 | 1.000 | 2708 | tags=34%, list=17%, signal=41% |
| 1062 | PID\_FASPATHWAY |  | 36 | 0.59 | 1.22 | 0.122 | 0.288 | 1.000 | 4395 | tags=53%, list=28%, signal=73% |
| 1063 | PID\_ERBB1\_DOWNSTREAM\_PATHWAY |  | 102 | 0.55 | 1.22 | 0.050 | 0.288 | 1.000 | 4185 | tags=47%, list=27%, signal=64% |
| 1064 | PID\_PI3KPLCTRKPATHWAY |  | 33 | 0.60 | 1.22 | 0.143 | 0.289 | 1.000 | 4113 | tags=52%, list=26%, signal=70% |
| 1065 | KEGG\_PATHOGENIC\_ESCHERICHIA\_COLI\_INFECTION |  | 50 | 0.58 | 1.22 | 0.102 | 0.289 | 1.000 | 2223 | tags=36%, list=14%, signal=42% |
| 1066 | MARIADASON\_REGULATED\_BY\_HISTONE\_ACETYLATION\_DN |  | 43 | 0.57 | 1.22 | 0.111 | 0.290 | 1.000 | 3954 | tags=47%, list=25%, signal=62% |
| 1067 | POTTI\_PACLITAXEL\_SENSITIVITY |  | 36 | 0.58 | 1.22 | 0.139 | 0.290 | 1.000 | 2787 | tags=33%, list=18%, signal=40% |
| 1068 | DUTTA\_APOPTOSIS\_VIA\_NFKB |  | 29 | 0.59 | 1.22 | 0.138 | 0.290 | 1.000 | 4508 | tags=48%, list=29%, signal=68% |
| 1069 | PID\_NCADHERINPATHWAY |  | 33 | 0.59 | 1.22 | 0.120 | 0.290 | 1.000 | 2223 | tags=36%, list=14%, signal=42% |
| 1070 | PID\_TCPTP\_PATHWAY |  | 43 | 0.58 | 1.22 | 0.121 | 0.291 | 1.000 | 3328 | tags=35%, list=21%, signal=44% |
| 1071 | KYNG\_WERNER\_SYNDROM\_AND\_NORMAL\_AGING\_UP |  | 78 | 0.56 | 1.22 | 0.050 | 0.291 | 1.000 | 3835 | tags=40%, list=24%, signal=52% |
| 1072 | BOGNI\_TREATMENT\_RELATED\_MYELOID\_LEUKEMIA\_DN |  | 26 | 0.61 | 1.22 | 0.142 | 0.291 | 1.000 | 1422 | tags=27%, list=9%, signal=30% |
| 1073 | WILSON\_PROTEASES\_AT\_TUMOR\_BONE\_INTERFACE\_UP |  | 21 | 0.61 | 1.22 | 0.160 | 0.291 | 1.000 | 3872 | tags=48%, list=25%, signal=63% |
| 1074 | BIOCARTA\_GPCR\_PATHWAY |  | 32 | 0.59 | 1.22 | 0.149 | 0.292 | 1.000 | 5031 | tags=59%, list=32%, signal=87% |
| 1075 | MORI\_SMALL\_PRE\_BII\_LYMPHOCYTE\_DN |  | 71 | 0.56 | 1.22 | 0.082 | 0.292 | 1.000 | 2820 | tags=39%, list=18%, signal=48% |
| 1076 | REACTOME\_FACTORS\_INVOLVED\_IN\_MEGAKARYOCYTE\_DEVELOPMENT\_AND\_PLATELET\_PRODUCTION |  | 105 | 0.55 | 1.22 | 0.036 | 0.293 | 1.000 | 3539 | tags=38%, list=22%, signal=49% |
| 1077 | MULLIGHAN\_NPM1\_MUTATED\_SIGNATURE\_2\_DN |  | 67 | 0.57 | 1.22 | 0.077 | 0.294 | 1.000 | 2082 | tags=30%, list=13%, signal=34% |
| 1078 | HOOI\_ST7\_TARGETS\_UP |  | 69 | 0.56 | 1.22 | 0.072 | 0.294 | 1.000 | 2420 | tags=26%, list=15%, signal=31% |
| 1079 | STEIN\_ESRRA\_TARGETS\_RESPONSIVE\_TO\_ESTROGEN\_UP |  | 25 | 0.61 | 1.22 | 0.168 | 0.294 | 1.000 | 4053 | tags=52%, list=26%, signal=70% |
| 1080 | REN\_ALVEOLAR\_RHABDOMYOSARCOMA\_DN |  | 394 | 0.54 | 1.22 | 0.000 | 0.293 | 1.000 | 2892 | tags=38%, list=18%, signal=45% |
| 1081 | DORMOY\_ELAVL1\_TARGETS |  | 16 | 0.64 | 1.22 | 0.166 | 0.293 | 1.000 | 2917 | tags=44%, list=19%, signal=54% |
| 1082 | KEGG\_PURINE\_METABOLISM |  | 151 | 0.55 | 1.22 | 0.022 | 0.294 | 1.000 | 3982 | tags=44%, list=25%, signal=59% |
| 1083 | DORN\_ADENOVIRUS\_INFECTION\_48HR\_DN |  | 35 | 0.58 | 1.22 | 0.137 | 0.295 | 1.000 | 2938 | tags=46%, list=19%, signal=56% |
| 1084 | SHIN\_B\_CELL\_LYMPHOMA\_CLUSTER\_5 |  | 17 | 0.64 | 1.22 | 0.176 | 0.296 | 1.000 | 1279 | tags=18%, list=8%, signal=19% |
| 1085 | BAE\_BRCA1\_TARGETS\_UP |  | 73 | 0.56 | 1.21 | 0.078 | 0.297 | 1.000 | 3258 | tags=49%, list=21%, signal=62% |
| 1086 | GENTILE\_UV\_LOW\_DOSE\_DN |  | 60 | 0.57 | 1.21 | 0.100 | 0.297 | 1.000 | 2934 | tags=38%, list=19%, signal=47% |
| 1087 | BEGUM\_TARGETS\_OF\_PAX3\_FOXO1\_FUSION\_UP |  | 55 | 0.56 | 1.21 | 0.103 | 0.299 | 1.000 | 3887 | tags=27%, list=25%, signal=36% |
| 1088 | GARGALOVIC\_RESPONSE\_TO\_OXIDIZED\_PHOSPHOLIPIDS\_TURQUOISE\_UP |  | 66 | 0.56 | 1.21 | 0.081 | 0.300 | 1.000 | 4775 | tags=56%, list=30%, signal=80% |
| 1089 | OSWALD\_HEMATOPOIETIC\_STEM\_CELL\_IN\_COLLAGEN\_GEL\_DN |  | 219 | 0.54 | 1.21 | 0.007 | 0.299 | 1.000 | 3553 | tags=37%, list=23%, signal=47% |
| 1090 | REACTOME\_SIGNALING\_BY\_INSULIN\_RECEPTOR |  | 99 | 0.55 | 1.21 | 0.042 | 0.301 | 1.000 | 3450 | tags=32%, list=22%, signal=41% |
| 1091 | REACTOME\_IMMUNOREGULATORY\_INTERACTIONS\_BETWEEN\_A\_LYMPHOID\_AND\_A\_NON\_LYMPHOID\_CELL |  | 48 | 0.57 | 1.21 | 0.114 | 0.302 | 1.000 | 1037 | tags=15%, list=7%, signal=16% |
| 1092 | REACTOME\_FANCONI\_ANEMIA\_PATHWAY |  | 19 | 0.63 | 1.21 | 0.183 | 0.302 | 1.000 | 4114 | tags=63%, list=26%, signal=85% |
| 1093 | DITTMER\_PTHLH\_TARGETS\_DN |  | 66 | 0.56 | 1.21 | 0.092 | 0.302 | 1.000 | 3643 | tags=52%, list=23%, signal=67% |
| 1094 | WONG\_MITOCHONDRIA\_GENE\_MODULE |  | 207 | 0.54 | 1.21 | 0.006 | 0.303 | 1.000 | 3415 | tags=43%, list=22%, signal=55% |
| 1095 | BANDRES\_RESPONSE\_TO\_CARMUSTIN\_MGMT\_48HR\_UP |  | 17 | 0.63 | 1.21 | 0.188 | 0.305 | 1.000 | 3420 | tags=35%, list=22%, signal=45% |
| 1096 | PID\_RHOA\_PATHWAY |  | 43 | 0.57 | 1.21 | 0.123 | 0.305 | 1.000 | 2880 | tags=37%, list=18%, signal=45% |
| 1097 | BILD\_SRC\_ONCOGENIC\_SIGNATURE |  | 55 | 0.57 | 1.21 | 0.090 | 0.305 | 1.000 | 4150 | tags=51%, list=26%, signal=69% |
| 1098 | OUELLET\_CULTURED\_OVARIAN\_CANCER\_INVASIVE\_VS\_LMP\_UP |  | 63 | 0.56 | 1.21 | 0.095 | 0.304 | 1.000 | 2359 | tags=44%, list=15%, signal=52% |
| 1099 | JECHLINGER\_EPITHELIAL\_TO\_MESENCHYMAL\_TRANSITION\_UP |  | 70 | 0.56 | 1.21 | 0.083 | 0.304 | 1.000 | 1732 | tags=29%, list=11%, signal=32% |
| 1100 | WEI\_MIR34A\_TARGETS |  | 126 | 0.55 | 1.21 | 0.037 | 0.304 | 1.000 | 3492 | tags=37%, list=22%, signal=48% |
| 1101 | REACTOME\_PROLONGED\_ERK\_ACTIVATION\_EVENTS |  | 17 | 0.63 | 1.21 | 0.167 | 0.304 | 1.000 | 5031 | tags=76%, list=32%, signal=112% |
| 1102 | XU\_CREBBP\_TARGETS\_UP |  | 25 | 0.61 | 1.21 | 0.167 | 0.305 | 1.000 | 2134 | tags=44%, list=14%, signal=51% |
| 1103 | ZHENG\_IL22\_SIGNALING\_DN |  | 38 | 0.58 | 1.21 | 0.137 | 0.305 | 1.000 | 3540 | tags=34%, list=22%, signal=44% |
| 1104 | YAO\_TEMPORAL\_RESPONSE\_TO\_PROGESTERONE\_CLUSTER\_4 |  | 15 | 0.63 | 1.21 | 0.191 | 0.306 | 1.000 | 2744 | tags=33%, list=17%, signal=40% |
| 1105 | SARTIPY\_NORMAL\_AT\_INSULIN\_RESISTANCE\_UP |  | 29 | 0.59 | 1.21 | 0.168 | 0.306 | 1.000 | 3957 | tags=48%, list=25%, signal=64% |
| 1106 | CHUNG\_BLISTER\_CYTOTOXICITY\_UP |  | 108 | 0.55 | 1.21 | 0.048 | 0.306 | 1.000 | 4282 | tags=48%, list=27%, signal=66% |
| 1107 | PECE\_MAMMARY\_STEM\_CELL\_DN |  | 121 | 0.55 | 1.21 | 0.038 | 0.306 | 1.000 | 3006 | tags=42%, list=19%, signal=52% |
| 1108 | REACTOME\_CYTOKINE\_SIGNALING\_IN\_IMMUNE\_SYSTEM |  | 229 | 0.54 | 1.21 | 0.009 | 0.306 | 1.000 | 3343 | tags=35%, list=21%, signal=44% |
| 1109 | HASLINGER\_B\_CLL\_WITH\_6Q21\_DELETION |  | 15 | 0.65 | 1.21 | 0.181 | 0.306 | 1.000 | 2068 | tags=40%, list=13%, signal=46% |
| 1110 | YAGI\_AML\_WITH\_INV\_16\_TRANSLOCATION |  | 375 | 0.54 | 1.21 | 0.000 | 0.306 | 1.000 | 4459 | tags=40%, list=28%, signal=55% |
| 1111 | LUI\_THYROID\_CANCER\_CLUSTER\_3 |  | 22 | 0.61 | 1.21 | 0.163 | 0.307 | 1.000 | 2504 | tags=50%, list=16%, signal=59% |
| 1112 | REACTOME\_BASIGIN\_INTERACTIONS |  | 23 | 0.61 | 1.21 | 0.173 | 0.307 | 1.000 | 832 | tags=22%, list=5%, signal=23% |
| 1113 | NICK\_RESPONSE\_TO\_PROC\_TREATMENT\_DN |  | 22 | 0.61 | 1.21 | 0.170 | 0.307 | 1.000 | 4683 | tags=59%, list=30%, signal=84% |
| 1114 | BIOCARTA\_GATA3\_PATHWAY |  | 15 | 0.65 | 1.21 | 0.191 | 0.307 | 1.000 | 2582 | tags=27%, list=16%, signal=32% |
| 1115 | SHARMA\_PILOCYTIC\_ASTROCYTOMA\_LOCATION\_UP |  | 21 | 0.61 | 1.21 | 0.183 | 0.307 | 1.000 | 1207 | tags=14%, list=8%, signal=15% |
| 1116 | HEDENFALK\_BREAST\_CANCER\_BRACX\_UP |  | 18 | 0.63 | 1.21 | 0.174 | 0.310 | 1.000 | 3152 | tags=56%, list=20%, signal=69% |
| 1117 | TCGA\_GLIOBLASTOMA\_COPY\_NUMBER\_DN |  | 28 | 0.59 | 1.21 | 0.152 | 0.310 | 1.000 | 3357 | tags=43%, list=21%, signal=54% |
| 1118 | LU\_EZH2\_TARGETS\_DN |  | 315 | 0.54 | 1.21 | 0.002 | 0.311 | 1.000 | 4111 | tags=39%, list=26%, signal=52% |
| 1119 | PID\_IL2\_PI3KPATHWAY |  | 32 | 0.59 | 1.21 | 0.164 | 0.312 | 1.000 | 3328 | tags=38%, list=21%, signal=47% |
| 1120 | GARGALOVIC\_RESPONSE\_TO\_OXIDIZED\_PHOSPHOLIPIDS\_GREEN\_DN |  | 24 | 0.60 | 1.21 | 0.180 | 0.315 | 1.000 | 3160 | tags=33%, list=20%, signal=42% |
| 1121 | COLDREN\_GEFITINIB\_RESISTANCE\_UP |  | 70 | 0.56 | 1.21 | 0.090 | 0.315 | 1.000 | 3739 | tags=49%, list=24%, signal=63% |
| 1122 | WONG\_IFNA2\_RESISTANCE\_DN |  | 30 | 0.59 | 1.21 | 0.161 | 0.315 | 1.000 | 2582 | tags=30%, list=16%, signal=36% |
| 1123 | GENTILE\_UV\_RESPONSE\_CLUSTER\_D1 |  | 15 | 0.64 | 1.21 | 0.189 | 0.315 | 1.000 | 3304 | tags=60%, list=21%, signal=76% |
| 1124 | SESTO\_RESPONSE\_TO\_UV\_C8 |  | 70 | 0.55 | 1.21 | 0.078 | 0.316 | 1.000 | 2953 | tags=43%, list=19%, signal=53% |
| 1125 | PID\_BETACATENIN\_NUC\_PATHWAY |  | 78 | 0.56 | 1.21 | 0.073 | 0.316 | 1.000 | 2647 | tags=28%, list=17%, signal=34% |
| 1126 | BIOCARTA\_HIVNEF\_PATHWAY |  | 55 | 0.56 | 1.20 | 0.100 | 0.318 | 1.000 | 3711 | tags=40%, list=24%, signal=52% |
| 1127 | REACTOME\_PI3K\_EVENTS\_IN\_ERBB4\_SIGNALING |  | 33 | 0.59 | 1.20 | 0.156 | 0.318 | 1.000 | 4185 | tags=48%, list=27%, signal=66% |
| 1128 | KEGG\_PROGESTERONE\_MEDIATED\_OOCYTE\_MATURATION |  | 81 | 0.55 | 1.20 | 0.076 | 0.319 | 1.000 | 4395 | tags=47%, list=28%, signal=65% |
| 1129 | HUMMEL\_BURKITTS\_LYMPHOMA\_UP |  | 34 | 0.58 | 1.20 | 0.165 | 0.320 | 1.000 | 3408 | tags=35%, list=22%, signal=45% |
| 1130 | LINDGREN\_BLADDER\_CANCER\_CLUSTER\_2A\_DN |  | 112 | 0.55 | 1.20 | 0.040 | 0.321 | 1.000 | 3600 | tags=42%, list=23%, signal=54% |
| 1131 | AMIT\_DELAYED\_EARLY\_GENES |  | 17 | 0.63 | 1.20 | 0.206 | 0.321 | 1.000 | 294 | tags=18%, list=2%, signal=18% |
| 1132 | BIOCARTA\_TFF\_PATHWAY |  | 20 | 0.61 | 1.20 | 0.178 | 0.322 | 1.000 | 5230 | tags=75%, list=33%, signal=112% |
| 1133 | ELVIDGE\_HIF1A\_TARGETS\_UP |  | 61 | 0.56 | 1.20 | 0.109 | 0.322 | 1.000 | 2069 | tags=33%, list=13%, signal=38% |
| 1134 | KIM\_WT1\_TARGETS\_8HR\_DN |  | 109 | 0.54 | 1.20 | 0.050 | 0.322 | 1.000 | 3643 | tags=38%, list=23%, signal=49% |
| 1135 | PID\_FOXOPATHWAY |  | 47 | 0.56 | 1.20 | 0.123 | 0.323 | 1.000 | 2521 | tags=36%, list=16%, signal=43% |
| 1136 | SENESE\_HDAC1\_AND\_HDAC2\_TARGETS\_UP |  | 195 | 0.54 | 1.20 | 0.014 | 0.324 | 1.000 | 2839 | tags=26%, list=18%, signal=32% |
| 1137 | REACTOME\_PRE\_NOTCH\_TRANSCRIPTION\_AND\_TRANSLATION |  | 24 | 0.59 | 1.20 | 0.193 | 0.324 | 1.000 | 3416 | tags=38%, list=22%, signal=48% |
| 1138 | YAGI\_AML\_FAB\_MARKERS |  | 175 | 0.54 | 1.20 | 0.020 | 0.324 | 1.000 | 2967 | tags=31%, list=19%, signal=38% |
| 1139 | SPIRA\_SMOKERS\_LUNG\_CANCER\_DN |  | 15 | 0.64 | 1.20 | 0.202 | 0.325 | 1.000 | 3010 | tags=47%, list=19%, signal=58% |
| 1140 | GINESTIER\_BREAST\_CANCER\_20Q13\_AMPLIFICATION\_UP |  | 95 | 0.55 | 1.20 | 0.055 | 0.325 | 1.000 | 3987 | tags=44%, list=25%, signal=59% |
| 1141 | GEISS\_RESPONSE\_TO\_DSRNA\_DN |  | 15 | 0.63 | 1.20 | 0.202 | 0.326 | 1.000 | 3958 | tags=40%, list=25%, signal=53% |
| 1142 | GUO\_HEX\_TARGETS\_UP |  | 74 | 0.55 | 1.20 | 0.097 | 0.326 | 1.000 | 4024 | tags=43%, list=26%, signal=58% |
| 1143 | KEGG\_THYROID\_CANCER |  | 29 | 0.58 | 1.20 | 0.168 | 0.327 | 1.000 | 3156 | tags=34%, list=20%, signal=43% |
| 1144 | REACTOME\_PI3K\_CASCADE |  | 63 | 0.56 | 1.20 | 0.098 | 0.327 | 1.000 | 3450 | tags=33%, list=22%, signal=43% |
| 1145 | SIG\_CHEMOTAXIS |  | 45 | 0.56 | 1.20 | 0.142 | 0.330 | 1.000 | 4098 | tags=42%, list=26%, signal=57% |
| 1146 | SA\_CASPASE\_CASCADE |  | 17 | 0.63 | 1.20 | 0.188 | 0.329 | 1.000 | 444 | tags=12%, list=3%, signal=12% |
| 1147 | THUM\_SYSTOLIC\_HEART\_FAILURE\_UP |  | 360 | 0.54 | 1.20 | 0.001 | 0.330 | 1.000 | 2726 | tags=28%, list=17%, signal=33% |
| 1148 | WANG\_CISPLATIN\_RESPONSE\_AND\_XPC\_DN |  | 196 | 0.54 | 1.20 | 0.015 | 0.330 | 1.000 | 3477 | tags=34%, list=22%, signal=43% |
| 1149 | PID\_SHP2\_PATHWAY |  | 55 | 0.57 | 1.20 | 0.102 | 0.331 | 1.000 | 5041 | tags=47%, list=32%, signal=69% |
| 1150 | TING\_SILENCED\_BY\_DICER |  | 24 | 0.59 | 1.20 | 0.178 | 0.331 | 1.000 | 1838 | tags=21%, list=12%, signal=24% |
| 1151 | WATANABE\_RECTAL\_CANCER\_RADIOTHERAPY\_RESPONSIVE\_UP |  | 95 | 0.55 | 1.20 | 0.071 | 0.332 | 1.000 | 3239 | tags=42%, list=21%, signal=53% |
| 1152 | BILD\_MYC\_ONCOGENIC\_SIGNATURE |  | 167 | 0.54 | 1.20 | 0.020 | 0.332 | 1.000 | 1938 | tags=30%, list=12%, signal=34% |
| 1153 | KYNG\_DNA\_DAMAGE\_BY\_4NQO |  | 33 | 0.59 | 1.20 | 0.161 | 0.332 | 1.000 | 4057 | tags=48%, list=26%, signal=65% |
| 1154 | CHESLER\_BRAIN\_QTL\_CIS |  | 65 | 0.55 | 1.20 | 0.105 | 0.332 | 1.000 | 3999 | tags=45%, list=25%, signal=60% |
| 1155 | REACTOME\_RESOLUTION\_OF\_AP\_SITES\_VIA\_THE\_MULTIPLE\_NUCLEOTIDE\_PATCH\_REPLACEMENT\_PATHWAY |  | 16 | 0.63 | 1.20 | 0.211 | 0.332 | 1.000 | 3762 | tags=50%, list=24%, signal=66% |
| 1156 | SNIJDERS\_AMPLIFIED\_IN\_HEAD\_AND\_NECK\_TUMORS |  | 35 | 0.58 | 1.20 | 0.148 | 0.333 | 1.000 | 4029 | tags=43%, list=26%, signal=57% |
| 1157 | VIETOR\_IFRD1\_TARGETS |  | 20 | 0.62 | 1.20 | 0.187 | 0.333 | 1.000 | 259 | tags=25%, list=2%, signal=25% |
| 1158 | PASQUALUCCI\_LYMPHOMA\_BY\_GC\_STAGE\_DN |  | 148 | 0.54 | 1.20 | 0.037 | 0.333 | 1.000 | 3025 | tags=31%, list=19%, signal=38% |
| 1159 | SMIRNOV\_RESPONSE\_TO\_IR\_6HR\_DN |  | 100 | 0.55 | 1.20 | 0.057 | 0.333 | 1.000 | 2922 | tags=28%, list=19%, signal=34% |
| 1160 | KENNY\_CTNNB1\_TARGETS\_UP |  | 46 | 0.56 | 1.20 | 0.134 | 0.335 | 1.000 | 2762 | tags=43%, list=18%, signal=53% |
| 1161 | CARDOSO\_RESPONSE\_TO\_GAMMA\_RADIATION\_AND\_3AB |  | 15 | 0.64 | 1.20 | 0.195 | 0.337 | 1.000 | 1052 | tags=20%, list=7%, signal=21% |
| 1162 | MURAKAMI\_UV\_RESPONSE\_6HR\_DN |  | 20 | 0.61 | 1.20 | 0.218 | 0.337 | 1.000 | 2387 | tags=25%, list=15%, signal=29% |
| 1163 | REACTOME\_CD28\_DEPENDENT\_PI3K\_AKT\_SIGNALING |  | 19 | 0.61 | 1.20 | 0.188 | 0.337 | 1.000 | 5623 | tags=68%, list=36%, signal=106% |
| 1164 | LINDGREN\_BLADDER\_CANCER\_CLUSTER\_1\_DN |  | 339 | 0.53 | 1.20 | 0.003 | 0.337 | 1.000 | 2549 | tags=35%, list=16%, signal=40% |
| 1165 | HOFFMANN\_PRE\_BI\_TO\_LARGE\_PRE\_BII\_LYMPHOCYTE\_UP |  | 32 | 0.58 | 1.20 | 0.179 | 0.337 | 1.000 | 2686 | tags=28%, list=17%, signal=34% |
| 1166 | REACTOME\_STRIATED\_MUSCLE\_CONTRACTION |  | 27 | 0.59 | 1.19 | 0.180 | 0.340 | 1.000 | 993 | tags=15%, list=6%, signal=16% |
| 1167 | MATSUDA\_NATURAL\_KILLER\_DIFFERENTIATION |  | 437 | 0.53 | 1.19 | 0.001 | 0.339 | 1.000 | 3000 | tags=30%, list=19%, signal=36% |
| 1168 | IVANOVA\_HEMATOPOIESIS\_INTERMEDIATE\_PROGENITOR |  | 122 | 0.54 | 1.19 | 0.049 | 0.340 | 1.000 | 4180 | tags=51%, list=27%, signal=69% |
| 1169 | KEGG\_CHRONIC\_MYELOID\_LEUKEMIA |  | 72 | 0.55 | 1.19 | 0.082 | 0.340 | 1.000 | 4466 | tags=47%, list=28%, signal=66% |
| 1170 | PID\_VEGFR1\_2\_PATHWAY |  | 67 | 0.55 | 1.19 | 0.114 | 0.342 | 1.000 | 3830 | tags=43%, list=24%, signal=57% |
| 1171 | REACTOME\_ENDOGENOUS\_STEROLS |  | 15 | 0.64 | 1.19 | 0.203 | 0.342 | 1.000 | 390 | tags=7%, list=2%, signal=7% |
| 1172 | REACTOME\_ANTIGEN\_PRESENTATION\_FOLDING\_ASSEMBLY\_AND\_PEPTIDE\_LOADING\_OF\_CLASS\_I\_MHC |  | 17 | 0.63 | 1.19 | 0.199 | 0.342 | 1.000 | 4498 | tags=71%, list=29%, signal=99% |
| 1173 | ZHAN\_MULTIPLE\_MYELOMA\_CD1\_VS\_CD2\_UP |  | 60 | 0.55 | 1.19 | 0.112 | 0.342 | 1.000 | 1665 | tags=27%, list=11%, signal=30% |
| 1174 | ZHAN\_MULTIPLE\_MYELOMA\_CD2\_DN |  | 39 | 0.57 | 1.19 | 0.155 | 0.343 | 1.000 | 3914 | tags=44%, list=25%, signal=58% |
| 1175 | AGUIRRE\_PANCREATIC\_CANCER\_COPY\_NUMBER\_UP |  | 258 | 0.53 | 1.19 | 0.006 | 0.344 | 1.000 | 4005 | tags=45%, list=25%, signal=59% |
| 1176 | ST\_DIFFERENTIATION\_PATHWAY\_IN\_PC12\_CELLS |  | 45 | 0.57 | 1.19 | 0.150 | 0.344 | 1.000 | 5018 | tags=56%, list=32%, signal=81% |
| 1177 | IIZUKA\_LIVER\_CANCER\_PROGRESSION\_L0\_L1\_DN |  | 19 | 0.62 | 1.19 | 0.195 | 0.344 | 1.000 | 1041 | tags=32%, list=7%, signal=34% |
| 1178 | ZHANG\_BREAST\_CANCER\_PROGENITORS\_DN |  | 134 | 0.54 | 1.19 | 0.044 | 0.344 | 1.000 | 3756 | tags=39%, list=24%, signal=51% |
| 1179 | BIOCARTA\_P53HYPOXIA\_PATHWAY |  | 22 | 0.60 | 1.19 | 0.212 | 0.344 | 1.000 | 4360 | tags=68%, list=28%, signal=94% |
| 1180 | BERNARD\_PPAPDC1B\_TARGETS\_UP |  | 35 | 0.57 | 1.19 | 0.174 | 0.344 | 1.000 | 4751 | tags=60%, list=30%, signal=86% |
| 1181 | JIANG\_HYPOXIA\_NORMAL |  | 269 | 0.53 | 1.19 | 0.012 | 0.344 | 1.000 | 3075 | tags=36%, list=20%, signal=44% |
| 1182 | RASHI\_RESPONSE\_TO\_IONIZING\_RADIATION\_4 |  | 52 | 0.56 | 1.19 | 0.127 | 0.345 | 1.000 | 3191 | tags=35%, list=20%, signal=43% |
| 1183 | KIM\_TIAL1\_TARGETS |  | 29 | 0.58 | 1.19 | 0.188 | 0.345 | 1.000 | 3593 | tags=59%, list=23%, signal=76% |
| 1184 | LU\_AGING\_BRAIN\_UP |  | 235 | 0.53 | 1.19 | 0.014 | 0.345 | 1.000 | 2001 | tags=26%, list=13%, signal=29% |
| 1185 | AKL\_HTLV1\_INFECTION\_UP |  | 24 | 0.60 | 1.19 | 0.190 | 0.346 | 1.000 | 3040 | tags=54%, list=19%, signal=67% |
| 1186 | PID\_ARF6DOWNSTREAMPATHWAY |  | 15 | 0.63 | 1.19 | 0.225 | 0.347 | 1.000 | 2587 | tags=40%, list=16%, signal=48% |
| 1187 | KAAB\_FAILED\_HEART\_ATRIUM\_DN |  | 132 | 0.54 | 1.19 | 0.039 | 0.346 | 1.000 | 2522 | tags=36%, list=16%, signal=43% |
| 1188 | WANG\_CISPLATIN\_RESPONSE\_AND\_XPC\_UP |  | 180 | 0.53 | 1.19 | 0.024 | 0.347 | 1.000 | 2443 | tags=31%, list=16%, signal=36% |
| 1189 | BRUINS\_UVC\_RESPONSE\_EARLY\_LATE |  | 255 | 0.53 | 1.19 | 0.006 | 0.347 | 1.000 | 4567 | tags=49%, list=29%, signal=67% |
| 1190 | MOOTHA\_PGC |  | 390 | 0.53 | 1.19 | 0.003 | 0.347 | 1.000 | 4078 | tags=47%, list=26%, signal=61% |
| 1191 | ZHAN\_MULTIPLE\_MYELOMA\_CD1\_AND\_CD2\_DN |  | 43 | 0.57 | 1.19 | 0.136 | 0.347 | 1.000 | 3079 | tags=35%, list=20%, signal=43% |
| 1192 | KEGG\_CYSTEINE\_AND\_METHIONINE\_METABOLISM |  | 32 | 0.58 | 1.19 | 0.168 | 0.347 | 1.000 | 3857 | tags=47%, list=24%, signal=62% |
| 1193 | REACTOME\_BASE\_EXCISION\_REPAIR |  | 18 | 0.61 | 1.19 | 0.228 | 0.347 | 1.000 | 3762 | tags=50%, list=24%, signal=66% |
| 1194 | PID\_INSULIN\_PATHWAY |  | 44 | 0.56 | 1.19 | 0.157 | 0.347 | 1.000 | 5018 | tags=61%, list=32%, signal=90% |
| 1195 | SCIAN\_INVERSED\_TARGETS\_OF\_TP53\_AND\_TP73\_DN |  | 26 | 0.59 | 1.19 | 0.197 | 0.349 | 1.000 | 3396 | tags=42%, list=22%, signal=54% |
| 1196 | HELLER\_SILENCED\_BY\_METHYLATION\_DN |  | 94 | 0.54 | 1.19 | 0.091 | 0.349 | 1.000 | 2977 | tags=37%, list=19%, signal=46% |
| 1197 | KIM\_GERMINAL\_CENTER\_T\_HELPER\_UP |  | 54 | 0.55 | 1.19 | 0.141 | 0.350 | 1.000 | 4591 | tags=50%, list=29%, signal=70% |
| 1198 | IGARASHI\_ATF4\_TARGETS\_DN |  | 79 | 0.55 | 1.19 | 0.111 | 0.351 | 1.000 | 2475 | tags=41%, list=16%, signal=48% |
| 1199 | REACTOME\_ENDOSOMAL\_SORTING\_COMPLEX\_REQUIRED\_FOR\_TRANSPORT\_ESCRT |  | 24 | 0.60 | 1.19 | 0.179 | 0.351 | 1.000 | 3980 | tags=58%, list=25%, signal=78% |
| 1200 | LEE\_RECENT\_THYMIC\_EMIGRANT |  | 184 | 0.53 | 1.19 | 0.019 | 0.351 | 1.000 | 5338 | tags=51%, list=34%, signal=76% |
| 1201 | WILLIAMS\_ESR2\_TARGETS\_UP |  | 24 | 0.60 | 1.19 | 0.195 | 0.351 | 1.000 | 2365 | tags=33%, list=15%, signal=39% |
| 1202 | KEGG\_ONE\_CARBON\_POOL\_BY\_FOLATE |  | 17 | 0.62 | 1.19 | 0.217 | 0.351 | 1.000 | 3182 | tags=59%, list=20%, signal=74% |
| 1203 | FLECHNER\_BIOPSY\_KIDNEY\_TRANSPLANT\_REJECTED\_VS\_OK\_DN |  | 497 | 0.53 | 1.19 | 0.001 | 0.350 | 1.000 | 3776 | tags=39%, list=24%, signal=49% |
| 1204 | AMIT\_EGF\_RESPONSE\_240\_HELA |  | 58 | 0.55 | 1.19 | 0.136 | 0.350 | 1.000 | 3489 | tags=40%, list=22%, signal=51% |
| 1205 | REACTOME\_INSULIN\_RECEPTOR\_SIGNALLING\_CASCADE |  | 78 | 0.55 | 1.19 | 0.101 | 0.350 | 1.000 | 3450 | tags=33%, list=22%, signal=42% |
| 1206 | VANDESLUIS\_NORMAL\_EMBRYOS\_DN |  | 22 | 0.60 | 1.19 | 0.208 | 0.351 | 1.000 | 1357 | tags=18%, list=9%, signal=20% |
| 1207 | THUM\_SYSTOLIC\_HEART\_FAILURE\_DN |  | 191 | 0.53 | 1.19 | 0.025 | 0.351 | 1.000 | 4838 | tags=44%, list=31%, signal=63% |
| 1208 | AGUIRRE\_PANCREATIC\_CANCER\_COPY\_NUMBER\_DN |  | 198 | 0.53 | 1.19 | 0.018 | 0.353 | 1.000 | 4371 | tags=48%, list=28%, signal=66% |
| 1209 | JAERVINEN\_AMPLIFIED\_IN\_LARYNGEAL\_CANCER |  | 33 | 0.58 | 1.19 | 0.183 | 0.354 | 1.000 | 4406 | tags=58%, list=28%, signal=80% |
| 1210 | ST\_FAS\_SIGNALING\_PATHWAY |  | 57 | 0.55 | 1.19 | 0.130 | 0.355 | 1.000 | 3437 | tags=40%, list=22%, signal=51% |
| 1211 | JOSEPH\_RESPONSE\_TO\_SODIUM\_BUTYRATE\_DN |  | 58 | 0.55 | 1.19 | 0.131 | 0.355 | 1.000 | 1038 | tags=17%, list=7%, signal=18% |
| 1212 | SANSOM\_APC\_TARGETS\_REQUIRE\_MYC |  | 176 | 0.54 | 1.19 | 0.024 | 0.354 | 1.000 | 4142 | tags=47%, list=26%, signal=63% |
| 1213 | CONCANNON\_APOPTOSIS\_BY\_EPOXOMICIN\_UP |  | 212 | 0.53 | 1.19 | 0.021 | 0.354 | 1.000 | 3698 | tags=41%, list=23%, signal=53% |
| 1214 | KEGG\_COLORECTAL\_CANCER |  | 62 | 0.55 | 1.19 | 0.122 | 0.354 | 1.000 | 4435 | tags=50%, list=28%, signal=69% |
| 1215 | BIOCARTA\_MITOCHONDRIA\_PATHWAY |  | 20 | 0.61 | 1.19 | 0.225 | 0.356 | 1.000 | 2026 | tags=30%, list=13%, signal=34% |
| 1216 | HUMMERICH\_SKIN\_CANCER\_PROGRESSION\_UP |  | 81 | 0.54 | 1.19 | 0.107 | 0.356 | 1.000 | 3296 | tags=32%, list=21%, signal=40% |
| 1217 | RAO\_BOUND\_BY\_SALL4\_ISOFORM\_B |  | 459 | 0.53 | 1.18 | 0.003 | 0.357 | 1.000 | 3863 | tags=34%, list=25%, signal=44% |
| 1218 | SUZUKI\_RESPONSE\_TO\_TSA |  | 16 | 0.63 | 1.18 | 0.219 | 0.357 | 1.000 | 2869 | tags=38%, list=18%, signal=46% |
| 1219 | LOPES\_METHYLATED\_IN\_COLON\_CANCER\_UP |  | 23 | 0.60 | 1.18 | 0.207 | 0.359 | 1.000 | 1338 | tags=13%, list=8%, signal=14% |
| 1220 | YAGI\_AML\_WITH\_T\_9\_11\_TRANSLOCATION |  | 116 | 0.54 | 1.18 | 0.056 | 0.359 | 1.000 | 4084 | tags=40%, list=26%, signal=53% |
| 1221 | REACTOME\_CITRIC\_ACID\_CYCLE\_TCA\_CYCLE |  | 19 | 0.61 | 1.18 | 0.208 | 0.361 | 1.000 | 3849 | tags=58%, list=24%, signal=77% |
| 1222 | WANG\_LMO4\_TARGETS\_UP |  | 287 | 0.52 | 1.18 | 0.010 | 0.362 | 1.000 | 4002 | tags=44%, list=25%, signal=58% |
| 1223 | GROSS\_HYPOXIA\_VIA\_ELK3\_AND\_HIF1A\_UP |  | 129 | 0.53 | 1.18 | 0.056 | 0.362 | 1.000 | 3461 | tags=39%, list=22%, signal=49% |
| 1224 | BALDWIN\_PRKCI\_TARGETS\_UP |  | 33 | 0.57 | 1.18 | 0.201 | 0.362 | 1.000 | 3176 | tags=36%, list=20%, signal=45% |
| 1225 | LIAO\_HAVE\_SOX4\_BINDING\_SITES |  | 40 | 0.57 | 1.18 | 0.169 | 0.363 | 1.000 | 2676 | tags=30%, list=17%, signal=36% |
| 1226 | XU\_GH1\_AUTOCRINE\_TARGETS\_DN |  | 120 | 0.53 | 1.18 | 0.065 | 0.362 | 1.000 | 3428 | tags=34%, list=22%, signal=43% |
| 1227 | AMIT\_EGF\_RESPONSE\_40\_MCF10A |  | 18 | 0.61 | 1.18 | 0.234 | 0.362 | 1.000 | 1985 | tags=39%, list=13%, signal=44% |
| 1228 | SHI\_SPARC\_TARGETS\_UP |  | 22 | 0.59 | 1.18 | 0.237 | 0.362 | 1.000 | 2430 | tags=23%, list=15%, signal=27% |
| 1229 | BURTON\_ADIPOGENESIS\_10 |  | 27 | 0.59 | 1.18 | 0.201 | 0.363 | 1.000 | 2676 | tags=44%, list=17%, signal=53% |
| 1230 | SMID\_BREAST\_CANCER\_RELAPSE\_IN\_BONE\_DN |  | 279 | 0.53 | 1.18 | 0.006 | 0.366 | 1.000 | 2080 | tags=21%, list=13%, signal=24% |
| 1231 | DITTMER\_PTHLH\_TARGETS\_UP |  | 102 | 0.54 | 1.18 | 0.074 | 0.366 | 1.000 | 3701 | tags=46%, list=23%, signal=60% |
| 1232 | OUILLETTE\_CLL\_13Q14\_DELETION\_UP |  | 58 | 0.55 | 1.18 | 0.137 | 0.366 | 1.000 | 3712 | tags=48%, list=24%, signal=63% |
| 1233 | REACTOME\_SHC1\_EVENTS\_IN\_ERBB4\_SIGNALING |  | 19 | 0.61 | 1.18 | 0.217 | 0.371 | 1.000 | 5230 | tags=68%, list=33%, signal=102% |
| 1234 | PID\_MET\_PATHWAY |  | 76 | 0.54 | 1.18 | 0.134 | 0.372 | 1.000 | 4098 | tags=47%, list=26%, signal=64% |
| 1235 | REACTOME\_ARMS\_MEDIATED\_ACTIVATION |  | 15 | 0.62 | 1.18 | 0.219 | 0.372 | 1.000 | 5031 | tags=73%, list=32%, signal=108% |
| 1236 | PID\_HDAC\_CLASSI\_PATHWAY |  | 62 | 0.55 | 1.18 | 0.128 | 0.371 | 1.000 | 2758 | tags=35%, list=18%, signal=43% |
| 1237 | STAMBOLSKY\_RESPONSE\_TO\_VITAMIN\_D3\_UP |  | 71 | 0.54 | 1.18 | 0.131 | 0.371 | 1.000 | 3987 | tags=34%, list=25%, signal=45% |
| 1238 | REACTOME\_P75\_NTR\_RECEPTOR\_MEDIATED\_SIGNALLING |  | 77 | 0.54 | 1.18 | 0.125 | 0.372 | 1.000 | 3328 | tags=29%, list=21%, signal=36% |
| 1239 | LIU\_VMYB\_TARGETS\_UP |  | 112 | 0.54 | 1.18 | 0.084 | 0.372 | 1.000 | 3735 | tags=38%, list=24%, signal=49% |
| 1240 | CLIMENT\_BREAST\_CANCER\_COPY\_NUMBER\_UP |  | 23 | 0.59 | 1.18 | 0.207 | 0.374 | 1.000 | 2666 | tags=26%, list=17%, signal=31% |
| 1241 | CHOW\_RASSF1\_TARGETS\_DN |  | 25 | 0.59 | 1.18 | 0.210 | 0.375 | 1.000 | 4483 | tags=52%, list=28%, signal=73% |
| 1242 | WU\_HBX\_TARGETS\_1\_DN |  | 21 | 0.60 | 1.18 | 0.227 | 0.375 | 1.000 | 3349 | tags=43%, list=21%, signal=54% |
| 1243 | PENG\_LEUCINE\_DEPRIVATION\_UP |  | 128 | 0.53 | 1.18 | 0.067 | 0.375 | 1.000 | 3242 | tags=37%, list=21%, signal=46% |
| 1244 | REACTOME\_PIP3\_ACTIVATES\_AKT\_SIGNALING |  | 24 | 0.59 | 1.18 | 0.214 | 0.378 | 1.000 | 4185 | tags=50%, list=27%, signal=68% |
| 1245 | BOYLAN\_MULTIPLE\_MYELOMA\_D\_CLUSTER\_UP |  | 26 | 0.59 | 1.18 | 0.211 | 0.378 | 1.000 | 1995 | tags=23%, list=13%, signal=26% |
| 1246 | REACTOME\_NUCLEAR\_EVENTS\_KINASE\_AND\_TRANSCRIPTION\_FACTOR\_ACTIVATION |  | 24 | 0.59 | 1.18 | 0.218 | 0.378 | 1.000 | 4395 | tags=63%, list=28%, signal=87% |
| 1247 | SCHLOSSER\_MYC\_AND\_SERUM\_RESPONSE\_SYNERGY |  | 31 | 0.57 | 1.18 | 0.196 | 0.381 | 1.000 | 2992 | tags=35%, list=19%, signal=44% |
| 1248 | PROVENZANI\_METASTASIS\_DN |  | 125 | 0.53 | 1.18 | 0.067 | 0.381 | 1.000 | 2286 | tags=32%, list=15%, signal=37% |
| 1249 | LU\_TUMOR\_ENDOTHELIAL\_MARKERS\_UP |  | 17 | 0.62 | 1.18 | 0.241 | 0.381 | 1.000 | 1338 | tags=18%, list=8%, signal=19% |
| 1250 | WOTTON\_RUNX\_TARGETS\_DN |  | 28 | 0.58 | 1.18 | 0.213 | 0.381 | 1.000 | 2031 | tags=29%, list=13%, signal=33% |
| 1251 | KRASNOSELSKAYA\_ILF3\_TARGETS\_DN |  | 44 | 0.56 | 1.18 | 0.174 | 0.381 | 1.000 | 2607 | tags=30%, list=17%, signal=35% |
| 1252 | GUO\_TARGETS\_OF\_IRS1\_AND\_IRS2 |  | 89 | 0.54 | 1.17 | 0.088 | 0.381 | 1.000 | 3335 | tags=33%, list=21%, signal=41% |
| 1253 | ST\_PHOSPHOINOSITIDE\_3\_KINASE\_PATHWAY |  | 36 | 0.57 | 1.17 | 0.164 | 0.383 | 1.000 | 4113 | tags=50%, list=26%, signal=68% |
| 1254 | VANOEVELEN\_MYOGENESIS\_SIN3A\_TARGETS |  | 185 | 0.53 | 1.17 | 0.032 | 0.383 | 1.000 | 4329 | tags=48%, list=27%, signal=65% |
| 1255 | AMUNDSON\_GENOTOXIC\_SIGNATURE |  | 96 | 0.54 | 1.17 | 0.082 | 0.383 | 1.000 | 3209 | tags=35%, list=20%, signal=44% |
| 1256 | ROVERSI\_GLIOMA\_LOH\_REGIONS |  | 40 | 0.56 | 1.17 | 0.186 | 0.383 | 1.000 | 3479 | tags=33%, list=22%, signal=42% |
| 1257 | PID\_P38ALPHABETADOWNSTREAMPATHWAY |  | 37 | 0.56 | 1.17 | 0.181 | 0.385 | 1.000 | 4395 | tags=65%, list=28%, signal=90% |
| 1258 | GAUSSMANN\_MLL\_AF4\_FUSION\_TARGETS\_A\_UP |  | 167 | 0.53 | 1.17 | 0.036 | 0.385 | 1.000 | 4971 | tags=49%, list=32%, signal=70% |
| 1259 | NIKOLSKY\_BREAST\_CANCER\_8Q12\_Q22\_AMPLICON |  | 106 | 0.53 | 1.17 | 0.081 | 0.385 | 1.000 | 4142 | tags=37%, list=26%, signal=50% |
| 1260 | HOWLIN\_PUBERTAL\_MAMMARY\_GLAND |  | 65 | 0.54 | 1.17 | 0.148 | 0.386 | 1.000 | 3491 | tags=28%, list=22%, signal=35% |
| 1261 | KEGG\_NEUROTROPHIN\_SIGNALING\_PATHWAY |  | 123 | 0.53 | 1.17 | 0.082 | 0.386 | 1.000 | 5031 | tags=53%, list=32%, signal=77% |
| 1262 | BERTUCCI\_MEDULLARY\_VS\_DUCTAL\_BREAST\_CANCER\_UP |  | 168 | 0.53 | 1.17 | 0.034 | 0.386 | 1.000 | 2931 | tags=30%, list=19%, signal=37% |
| 1263 | AMUNDSON\_POOR\_SURVIVAL\_AFTER\_GAMMA\_RADIATION\_8G |  | 88 | 0.54 | 1.17 | 0.100 | 0.386 | 1.000 | 2842 | tags=34%, list=18%, signal=41% |
| 1264 | BIOCARTA\_P38MAPK\_PATHWAY |  | 39 | 0.56 | 1.17 | 0.190 | 0.386 | 1.000 | 5391 | tags=67%, list=34%, signal=101% |
| 1265 | BROWNE\_HCMV\_INFECTION\_6HR\_DN |  | 141 | 0.53 | 1.17 | 0.068 | 0.386 | 1.000 | 4591 | tags=53%, list=29%, signal=74% |
| 1266 | NING\_CHRONIC\_OBSTRUCTIVE\_PULMONARY\_DISEASE\_UP |  | 145 | 0.53 | 1.17 | 0.054 | 0.386 | 1.000 | 2992 | tags=32%, list=19%, signal=40% |
| 1267 | KAMMINGA\_SENESCENCE |  | 36 | 0.56 | 1.17 | 0.200 | 0.386 | 1.000 | 738 | tags=17%, list=5%, signal=17% |
| 1268 | BIOCARTA\_SPPA\_PATHWAY |  | 21 | 0.60 | 1.17 | 0.231 | 0.387 | 1.000 | 3939 | tags=43%, list=25%, signal=57% |
| 1269 | SCHAEFFER\_PROSTATE\_DEVELOPMENT\_12HR\_DN |  | 54 | 0.55 | 1.17 | 0.166 | 0.387 | 1.000 | 1366 | tags=20%, list=9%, signal=22% |
| 1270 | GROSS\_ELK3\_TARGETS\_UP |  | 27 | 0.58 | 1.17 | 0.211 | 0.388 | 1.000 | 2669 | tags=37%, list=17%, signal=45% |
| 1271 | MULLIGHAN\_NPM1\_MUTATED\_SIGNATURE\_2\_UP |  | 117 | 0.53 | 1.17 | 0.087 | 0.388 | 1.000 | 4303 | tags=43%, list=27%, signal=58% |
| 1272 | REACTOME\_SIGNALING\_BY\_ERBB4 |  | 78 | 0.54 | 1.17 | 0.116 | 0.387 | 1.000 | 4715 | tags=47%, list=30%, signal=67% |
| 1273 | ROYLANCE\_BREAST\_CANCER\_16Q\_COPY\_NUMBER\_DN |  | 18 | 0.60 | 1.17 | 0.232 | 0.387 | 1.000 | 1805 | tags=28%, list=11%, signal=31% |
| 1274 | ROESSLER\_LIVER\_CANCER\_METASTASIS\_UP |  | 101 | 0.53 | 1.17 | 0.102 | 0.390 | 1.000 | 3144 | tags=36%, list=20%, signal=44% |
| 1275 | HERNANDEZ\_MITOTIC\_ARREST\_BY\_DOCETAXEL\_1\_UP |  | 32 | 0.57 | 1.17 | 0.211 | 0.390 | 1.000 | 346 | tags=9%, list=2%, signal=10% |
| 1276 | BIOCARTA\_CK1\_PATHWAY |  | 16 | 0.62 | 1.17 | 0.246 | 0.390 | 1.000 | 3939 | tags=44%, list=25%, signal=58% |
| 1277 | BIOCARTA\_AMI\_PATHWAY |  | 19 | 0.61 | 1.17 | 0.237 | 0.391 | 1.000 | 2979 | tags=16%, list=19%, signal=19% |
| 1278 | DARWICHE\_SQUAMOUS\_CELL\_CARCINOMA\_UP |  | 120 | 0.53 | 1.17 | 0.082 | 0.392 | 1.000 | 1610 | tags=24%, list=10%, signal=27% |
| 1279 | SESTO\_RESPONSE\_TO\_UV\_C6 |  | 35 | 0.56 | 1.17 | 0.186 | 0.392 | 1.000 | 1732 | tags=29%, list=11%, signal=32% |
| 1280 | AKL\_HTLV1\_INFECTION\_DN |  | 60 | 0.55 | 1.17 | 0.160 | 0.392 | 1.000 | 4204 | tags=42%, list=27%, signal=57% |
| 1281 | SCHLINGEMANN\_SKIN\_CARCINOGENESIS\_TPA\_UP |  | 34 | 0.56 | 1.17 | 0.215 | 0.393 | 1.000 | 2953 | tags=35%, list=19%, signal=43% |
| 1282 | LIU\_SOX4\_TARGETS\_DN |  | 265 | 0.52 | 1.17 | 0.024 | 0.393 | 1.000 | 3741 | tags=44%, list=24%, signal=56% |
| 1283 | ROESSLER\_LIVER\_CANCER\_METASTASIS\_DN |  | 42 | 0.56 | 1.17 | 0.182 | 0.393 | 1.000 | 3780 | tags=45%, list=24%, signal=59% |
| 1284 | MORI\_PLASMA\_CELL\_UP |  | 41 | 0.56 | 1.17 | 0.191 | 0.394 | 1.000 | 2685 | tags=29%, list=17%, signal=35% |
| 1285 | GREGORY\_SYNTHETIC\_LETHAL\_WITH\_IMATINIB |  | 120 | 0.53 | 1.17 | 0.091 | 0.395 | 1.000 | 2017 | tags=23%, list=13%, signal=26% |
| 1286 | MA\_MYELOID\_DIFFERENTIATION\_DN |  | 43 | 0.56 | 1.17 | 0.206 | 0.395 | 1.000 | 3469 | tags=37%, list=22%, signal=48% |
| 1287 | BROWNE\_HCMV\_INFECTION\_12HR\_DN |  | 88 | 0.54 | 1.17 | 0.124 | 0.396 | 1.000 | 4553 | tags=48%, list=29%, signal=67% |
| 1288 | KOKKINAKIS\_METHIONINE\_DEPRIVATION\_48HR\_DN |  | 63 | 0.54 | 1.17 | 0.153 | 0.396 | 1.000 | 3484 | tags=44%, list=22%, signal=57% |
| 1289 | WANG\_METASTASIS\_OF\_BREAST\_CANCER\_ESR1\_DN |  | 25 | 0.58 | 1.17 | 0.227 | 0.397 | 1.000 | 1871 | tags=28%, list=12%, signal=32% |
| 1290 | LIN\_APC\_TARGETS |  | 66 | 0.54 | 1.17 | 0.137 | 0.397 | 1.000 | 2586 | tags=36%, list=16%, signal=43% |
| 1291 | GRAHAM\_CML\_DIVIDING\_VS\_NORMAL\_QUIESCENT\_DN |  | 77 | 0.54 | 1.17 | 0.142 | 0.398 | 1.000 | 2269 | tags=22%, list=14%, signal=26% |
| 1292 | STARK\_PREFRONTAL\_CORTEX\_22Q11\_DELETION\_UP |  | 170 | 0.53 | 1.17 | 0.044 | 0.399 | 1.000 | 4196 | tags=41%, list=27%, signal=56% |
| 1293 | MODY\_HIPPOCAMPUS\_NEONATAL |  | 32 | 0.57 | 1.17 | 0.222 | 0.399 | 1.000 | 1792 | tags=38%, list=11%, signal=42% |
| 1294 | BACOLOD\_RESISTANCE\_TO\_ALKYLATING\_AGENTS\_UP |  | 20 | 0.60 | 1.17 | 0.237 | 0.399 | 1.000 | 4161 | tags=40%, list=26%, signal=54% |
| 1295 | DAIRKEE\_TERT\_TARGETS\_UP |  | 313 | 0.52 | 1.17 | 0.011 | 0.399 | 1.000 | 3707 | tags=40%, list=24%, signal=51% |
| 1296 | MOOTHA\_HUMAN\_MITODB\_6\_2002 |  | 411 | 0.52 | 1.17 | 0.006 | 0.399 | 1.000 | 3770 | tags=42%, list=24%, signal=54% |
| 1297 | REACTOME\_CELL\_DEATH\_SIGNALLING\_VIA\_NRAGE\_NRIF\_AND\_NADE |  | 56 | 0.54 | 1.17 | 0.174 | 0.400 | 1.000 | 3328 | tags=25%, list=21%, signal=32% |
| 1298 | CHEN\_NEUROBLASTOMA\_COPY\_NUMBER\_GAINS |  | 45 | 0.55 | 1.17 | 0.197 | 0.400 | 1.000 | 4393 | tags=44%, list=28%, signal=61% |
| 1299 | REACTOME\_ACTIVATION\_OF\_CHAPERONE\_GENES\_BY\_XBP1S |  | 42 | 0.56 | 1.17 | 0.217 | 0.400 | 1.000 | 3664 | tags=43%, list=23%, signal=56% |
| 1300 | GUENTHER\_GROWTH\_SPHERICAL\_VS\_ADHERENT\_DN |  | 25 | 0.58 | 1.17 | 0.242 | 0.401 | 1.000 | 1168 | tags=28%, list=7%, signal=30% |
| 1301 | PID\_INTEGRIN5\_PATHWAY |  | 17 | 0.61 | 1.17 | 0.250 | 0.401 | 1.000 | 2842 | tags=35%, list=18%, signal=43% |
| 1302 | VALK\_AML\_CLUSTER\_6 |  | 27 | 0.58 | 1.17 | 0.219 | 0.401 | 1.000 | 3932 | tags=41%, list=25%, signal=54% |
| 1303 | HOEBEKE\_LYMPHOID\_STEM\_CELL\_DN |  | 80 | 0.54 | 1.17 | 0.139 | 0.400 | 1.000 | 3305 | tags=34%, list=21%, signal=42% |
| 1304 | PID\_CD8TCRDOWNSTREAMPATHWAY |  | 56 | 0.54 | 1.17 | 0.170 | 0.400 | 1.000 | 2737 | tags=18%, list=17%, signal=22% |
| 1305 | ZEMBUTSU\_SENSITIVITY\_TO\_METHOTREXATE |  | 17 | 0.61 | 1.16 | 0.244 | 0.401 | 1.000 | 3332 | tags=35%, list=21%, signal=45% |
| 1306 | VERNELL\_RETINOBLASTOMA\_PATHWAY\_DN |  | 18 | 0.61 | 1.16 | 0.238 | 0.401 | 1.000 | 2066 | tags=28%, list=13%, signal=32% |
| 1307 | GAZDA\_DIAMOND\_BLACKFAN\_ANEMIA\_ERYTHROID\_UP |  | 19 | 0.60 | 1.16 | 0.234 | 0.401 | 1.000 | 4675 | tags=58%, list=30%, signal=82% |
| 1308 | ZHANG\_ANTIVIRAL\_RESPONSE\_TO\_RIBAVIRIN\_DN |  | 49 | 0.55 | 1.16 | 0.172 | 0.400 | 1.000 | 1914 | tags=20%, list=12%, signal=23% |
| 1309 | SERVITJA\_LIVER\_HNF1A\_TARGETS\_UP |  | 129 | 0.52 | 1.16 | 0.094 | 0.401 | 1.000 | 2977 | tags=32%, list=19%, signal=39% |
| 1310 | GAUSSMANN\_MLL\_AF4\_FUSION\_TARGETS\_F\_DN |  | 31 | 0.57 | 1.16 | 0.223 | 0.401 | 1.000 | 3216 | tags=35%, list=20%, signal=45% |
| 1311 | PEART\_HDAC\_PROLIFERATION\_CLUSTER\_UP |  | 54 | 0.55 | 1.16 | 0.177 | 0.402 | 1.000 | 4216 | tags=52%, list=27%, signal=71% |
| 1312 | PID\_ARF6\_TRAFFICKINGPATHWAY |  | 47 | 0.54 | 1.16 | 0.199 | 0.403 | 1.000 | 2427 | tags=32%, list=15%, signal=38% |
| 1313 | INGA\_TP53\_TARGETS |  | 15 | 0.62 | 1.16 | 0.258 | 0.406 | 1.000 | 3561 | tags=47%, list=23%, signal=60% |
| 1314 | KYNG\_WERNER\_SYNDROM\_UP |  | 15 | 0.62 | 1.16 | 0.252 | 0.406 | 1.000 | 1625 | tags=33%, list=10%, signal=37% |
| 1315 | LEE\_TARGETS\_OF\_PTCH1\_AND\_SUFU\_UP |  | 48 | 0.54 | 1.16 | 0.198 | 0.408 | 1.000 | 1281 | tags=29%, list=8%, signal=32% |
| 1316 | HERNANDEZ\_ABERRANT\_MITOSIS\_BY\_DOCETACEL\_2NM\_DN |  | 24 | 0.58 | 1.16 | 0.237 | 0.409 | 1.000 | 1516 | tags=25%, list=10%, signal=28% |
| 1317 | LIN\_NPAS4\_TARGETS\_DN |  | 61 | 0.54 | 1.16 | 0.159 | 0.411 | 1.000 | 5013 | tags=38%, list=32%, signal=55% |
| 1318 | LI\_CISPLATIN\_RESISTANCE\_UP |  | 23 | 0.58 | 1.16 | 0.229 | 0.411 | 1.000 | 3776 | tags=30%, list=24%, signal=40% |
| 1319 | KEGG\_MELANOMA |  | 71 | 0.54 | 1.16 | 0.146 | 0.413 | 1.000 | 3189 | tags=27%, list=20%, signal=33% |
| 1320 | BRUINS\_UVC\_RESPONSE\_VIA\_TP53\_GROUP\_D |  | 232 | 0.52 | 1.16 | 0.030 | 0.412 | 1.000 | 3297 | tags=26%, list=21%, signal=33% |
| 1321 | LUI\_THYROID\_CANCER\_PAX8\_PPARG\_DN |  | 38 | 0.56 | 1.16 | 0.214 | 0.412 | 1.000 | 2504 | tags=47%, list=16%, signal=56% |
| 1322 | KEGG\_SNARE\_INTERACTIONS\_IN\_VESICULAR\_TRANSPORT |  | 32 | 0.56 | 1.16 | 0.224 | 0.415 | 1.000 | 5504 | tags=66%, list=35%, signal=101% |
| 1323 | XU\_RESPONSE\_TO\_TRETINOIN\_AND\_NSC682994\_UP |  | 15 | 0.62 | 1.16 | 0.254 | 0.415 | 1.000 | 1780 | tags=40%, list=11%, signal=45% |
| 1324 | CHO\_NR4A1\_TARGETS |  | 30 | 0.56 | 1.16 | 0.222 | 0.417 | 1.000 | 2195 | tags=27%, list=14%, signal=31% |
| 1325 | REACTOME\_DEPOSITION\_OF\_NEW\_CENPA\_CONTAINING\_NUCLEOSOMES\_AT\_THE\_CENTROMERE |  | 43 | 0.55 | 1.16 | 0.204 | 0.417 | 1.000 | 3065 | tags=33%, list=19%, signal=40% |
| 1326 | MARTIN\_INTERACT\_WITH\_HDAC |  | 43 | 0.55 | 1.16 | 0.195 | 0.417 | 1.000 | 4483 | tags=56%, list=28%, signal=78% |
| 1327 | IZADPANAH\_STEM\_CELL\_ADIPOSE\_VS\_BONE\_UP |  | 105 | 0.53 | 1.16 | 0.104 | 0.419 | 1.000 | 3643 | tags=39%, list=23%, signal=50% |
| 1328 | REACTOME\_PYRIMIDINE\_METABOLISM |  | 23 | 0.58 | 1.16 | 0.247 | 0.419 | 1.000 | 3182 | tags=39%, list=20%, signal=49% |
| 1329 | MCCLUNG\_COCAINE\_REWARD\_5D |  | 75 | 0.54 | 1.16 | 0.130 | 0.421 | 1.000 | 3319 | tags=29%, list=21%, signal=37% |
| 1330 | SIG\_INSULIN\_RECEPTOR\_PATHWAY\_IN\_CARDIAC\_MYOCYTES |  | 50 | 0.55 | 1.16 | 0.181 | 0.422 | 1.000 | 5031 | tags=56%, list=32%, signal=82% |
| 1331 | RORIE\_TARGETS\_OF\_EWSR1\_FLI1\_FUSION\_UP |  | 28 | 0.57 | 1.16 | 0.244 | 0.423 | 1.000 | 1297 | tags=25%, list=8%, signal=27% |
| 1332 | VANHARANTA\_UTERINE\_FIBROID\_WITH\_7Q\_DELETION\_DN |  | 35 | 0.56 | 1.16 | 0.223 | 0.424 | 1.000 | 4576 | tags=51%, list=29%, signal=72% |
| 1333 | LEE\_CALORIE\_RESTRICTION\_MUSCLE\_DN |  | 49 | 0.55 | 1.16 | 0.191 | 0.424 | 1.000 | 3581 | tags=41%, list=23%, signal=53% |
| 1334 | GINESTIER\_BREAST\_CANCER\_20Q13\_AMPLIFICATION\_DN |  | 132 | 0.52 | 1.16 | 0.088 | 0.425 | 1.000 | 4202 | tags=45%, list=27%, signal=61% |
| 1335 | STAEGE\_EWING\_FAMILY\_TUMOR |  | 28 | 0.57 | 1.16 | 0.241 | 0.425 | 1.000 | 4253 | tags=32%, list=27%, signal=44% |
| 1336 | LINDSTEDT\_DENDRITIC\_CELL\_MATURATION\_C |  | 61 | 0.54 | 1.16 | 0.190 | 0.425 | 1.000 | 1526 | tags=21%, list=10%, signal=24% |
| 1337 | REACTOME\_TRANSFERRIN\_ENDOCYTOSIS\_AND\_RECYCLING |  | 25 | 0.58 | 1.16 | 0.229 | 0.425 | 1.000 | 2361 | tags=28%, list=15%, signal=33% |
| 1338 | BIOCARTA\_PGC1A\_PATHWAY |  | 22 | 0.57 | 1.16 | 0.275 | 0.425 | 1.000 | 1183 | tags=18%, list=8%, signal=20% |
| 1339 | PETROVA\_ENDOTHELIUM\_LYMPHATIC\_VS\_BLOOD\_UP |  | 124 | 0.53 | 1.16 | 0.083 | 0.426 | 1.000 | 2999 | tags=27%, list=19%, signal=33% |
| 1340 | BIOCARTA\_MPR\_PATHWAY |  | 32 | 0.56 | 1.16 | 0.253 | 0.426 | 1.000 | 3457 | tags=41%, list=22%, signal=52% |
| 1341 | WOOD\_EBV\_EBNA1\_TARGETS\_UP |  | 103 | 0.53 | 1.16 | 0.113 | 0.426 | 1.000 | 2950 | tags=36%, list=19%, signal=44% |
| 1342 | REACTOME\_MAPK\_TARGETS\_NUCLEAR\_EVENTS\_MEDIATED\_BY\_MAP\_KINASES |  | 30 | 0.57 | 1.15 | 0.227 | 0.426 | 1.000 | 4820 | tags=67%, list=31%, signal=96% |
| 1343 | BIOCARTA\_CCR3\_PATHWAY |  | 22 | 0.58 | 1.15 | 0.244 | 0.430 | 1.000 | 4482 | tags=45%, list=28%, signal=63% |
| 1344 | PID\_ANTHRAXPATHWAY |  | 17 | 0.61 | 1.15 | 0.261 | 0.432 | 1.000 | 3643 | tags=47%, list=23%, signal=61% |
| 1345 | IWANAGA\_CARCINOGENESIS\_BY\_KRAS\_PTEN\_UP |  | 154 | 0.52 | 1.15 | 0.084 | 0.431 | 1.000 | 3453 | tags=32%, list=22%, signal=41% |
| 1346 | JAZAG\_TGFB1\_SIGNALING\_UP |  | 98 | 0.53 | 1.15 | 0.127 | 0.431 | 1.000 | 3442 | tags=35%, list=22%, signal=44% |
| 1347 | PHONG\_TNF\_RESPONSE\_VIA\_P38\_COMPLETE |  | 201 | 0.52 | 1.15 | 0.045 | 0.432 | 1.000 | 3891 | tags=43%, list=25%, signal=56% |
| 1348 | HUTTMANN\_B\_CLL\_POOR\_SURVIVAL\_DN |  | 53 | 0.54 | 1.15 | 0.200 | 0.434 | 1.000 | 4263 | tags=40%, list=27%, signal=54% |
| 1349 | SA\_TRKA\_RECEPTOR |  | 16 | 0.61 | 1.15 | 0.282 | 0.435 | 1.000 | 5230 | tags=69%, list=33%, signal=103% |
| 1350 | FRASOR\_RESPONSE\_TO\_ESTRADIOL\_UP |  | 37 | 0.56 | 1.15 | 0.235 | 0.436 | 1.000 | 701 | tags=11%, list=4%, signal=11% |
| 1351 | STEIN\_ESRRA\_TARGETS\_DN |  | 86 | 0.53 | 1.15 | 0.127 | 0.436 | 1.000 | 3352 | tags=38%, list=21%, signal=48% |
| 1352 | LEE\_LIVER\_CANCER\_HEPATOBLAST |  | 15 | 0.62 | 1.15 | 0.270 | 0.438 | 1.000 | 1110 | tags=40%, list=7%, signal=43% |
| 1353 | KEGG\_WNT\_SIGNALING\_PATHWAY |  | 143 | 0.52 | 1.15 | 0.081 | 0.439 | 1.000 | 4360 | tags=39%, list=28%, signal=54% |
| 1354 | GROSS\_HYPOXIA\_VIA\_ELK3\_AND\_HIF1A\_DN |  | 91 | 0.52 | 1.15 | 0.148 | 0.438 | 1.000 | 4074 | tags=51%, list=26%, signal=68% |
| 1355 | VALK\_AML\_CLUSTER\_11 |  | 35 | 0.56 | 1.15 | 0.217 | 0.438 | 1.000 | 1501 | tags=26%, list=10%, signal=28% |
| 1356 | LUI\_THYROID\_CANCER\_PAX8\_PPARG\_UP |  | 36 | 0.55 | 1.15 | 0.234 | 0.439 | 1.000 | 3922 | tags=47%, list=25%, signal=63% |
| 1357 | ST\_JNK\_MAPK\_PATHWAY |  | 38 | 0.55 | 1.15 | 0.251 | 0.439 | 1.000 | 4807 | tags=61%, list=31%, signal=87% |
| 1358 | PLASARI\_TGFB1\_SIGNALING\_VIA\_NFIC\_10HR\_UP |  | 45 | 0.54 | 1.15 | 0.208 | 0.440 | 1.000 | 1048 | tags=18%, list=7%, signal=19% |
| 1359 | WALLACE\_PROSTATE\_CANCER\_RACE\_DN |  | 66 | 0.53 | 1.15 | 0.186 | 0.443 | 1.000 | 4360 | tags=47%, list=28%, signal=65% |
| 1360 | AMUNDSON\_RESPONSE\_TO\_ARSENITE |  | 187 | 0.51 | 1.15 | 0.065 | 0.444 | 1.000 | 4195 | tags=45%, list=27%, signal=61% |
| 1361 | BIOCARTA\_CTCF\_PATHWAY |  | 22 | 0.58 | 1.15 | 0.278 | 0.444 | 1.000 | 3297 | tags=41%, list=21%, signal=52% |
| 1362 | PID\_P38\_MKK3\_6PATHWAY |  | 24 | 0.58 | 1.15 | 0.244 | 0.446 | 1.000 | 6084 | tags=71%, list=39%, signal=115% |
| 1363 | PID\_REG\_GR\_PATHWAY |  | 79 | 0.53 | 1.15 | 0.143 | 0.446 | 1.000 | 4395 | tags=41%, list=28%, signal=56% |
| 1364 | PENG\_GLUCOSE\_DEPRIVATION\_UP |  | 41 | 0.55 | 1.15 | 0.237 | 0.447 | 1.000 | 1710 | tags=29%, list=11%, signal=33% |
| 1365 | POTTI\_DOCETAXEL\_SENSITIVITY |  | 43 | 0.54 | 1.15 | 0.222 | 0.448 | 1.000 | 2775 | tags=30%, list=18%, signal=37% |
| 1366 | HASLINGER\_B\_CLL\_WITH\_17P13\_DELETION |  | 20 | 0.58 | 1.15 | 0.270 | 0.448 | 1.000 | 2044 | tags=30%, list=13%, signal=34% |
| 1367 | REACTOME\_CA\_DEPENDENT\_EVENTS |  | 27 | 0.57 | 1.15 | 0.249 | 0.453 | 1.000 | 3830 | tags=37%, list=24%, signal=49% |
| 1368 | PLASARI\_TGFB1\_SIGNALING\_VIA\_NFIC\_1HR\_UP |  | 29 | 0.56 | 1.15 | 0.256 | 0.452 | 1.000 | 3449 | tags=38%, list=22%, signal=48% |
| 1369 | THEILGAARD\_NEUTROPHIL\_AT\_SKIN\_WOUND\_DN |  | 206 | 0.51 | 1.15 | 0.061 | 0.454 | 1.000 | 4482 | tags=45%, list=28%, signal=62% |
| 1370 | FRIDMAN\_SENESCENCE\_UP |  | 75 | 0.53 | 1.15 | 0.171 | 0.454 | 1.000 | 3469 | tags=37%, list=22%, signal=48% |
| 1371 | PID\_DNAPK\_PATHWAY |  | 15 | 0.60 | 1.15 | 0.268 | 0.453 | 1.000 | 3642 | tags=40%, list=23%, signal=52% |
| 1372 | REACTOME\_SIGNALING\_BY\_ROBO\_RECEPTOR |  | 29 | 0.56 | 1.14 | 0.248 | 0.455 | 1.000 | 3634 | tags=41%, list=23%, signal=54% |
| 1373 | PID\_UPA\_UPAR\_PATHWAY |  | 39 | 0.55 | 1.14 | 0.235 | 0.454 | 1.000 | 2842 | tags=21%, list=18%, signal=25% |
| 1374 | REACTOME\_PPARA\_ACTIVATES\_GENE\_EXPRESSION |  | 85 | 0.53 | 1.14 | 0.167 | 0.454 | 1.000 | 3117 | tags=33%, list=20%, signal=41% |
| 1375 | PID\_SMAD2\_3NUCLEARPATHWAY |  | 75 | 0.53 | 1.14 | 0.162 | 0.454 | 1.000 | 4281 | tags=45%, list=27%, signal=62% |
| 1376 | LASTOWSKA\_NEUROBLASTOMA\_COPY\_NUMBER\_UP |  | 151 | 0.52 | 1.14 | 0.094 | 0.454 | 1.000 | 4512 | tags=48%, list=29%, signal=67% |
| 1377 | KEGG\_PANCREATIC\_CANCER |  | 70 | 0.53 | 1.14 | 0.196 | 0.455 | 1.000 | 3201 | tags=37%, list=20%, signal=46% |
| 1378 | MOREIRA\_RESPONSE\_TO\_TSA\_UP |  | 26 | 0.57 | 1.14 | 0.259 | 0.455 | 1.000 | 3191 | tags=38%, list=20%, signal=48% |
| 1379 | POMEROY\_MEDULLOBLASTOMA\_DESMOPLASIC\_VS\_CLASSIC\_UP |  | 56 | 0.53 | 1.14 | 0.201 | 0.455 | 1.000 | 3051 | tags=30%, list=19%, signal=38% |
| 1380 | OZANNE\_AP1\_TARGETS\_UP |  | 15 | 0.61 | 1.14 | 0.291 | 0.455 | 1.000 | 1282 | tags=27%, list=8%, signal=29% |
| 1381 | KEGG\_HUNTINGTONS\_DISEASE |  | 164 | 0.51 | 1.14 | 0.080 | 0.455 | 1.000 | 4755 | tags=50%, list=30%, signal=71% |
| 1382 | OKUMURA\_INFLAMMATORY\_RESPONSE\_LPS |  | 160 | 0.51 | 1.14 | 0.077 | 0.454 | 1.000 | 3987 | tags=38%, list=25%, signal=50% |
| 1383 | SANSOM\_WNT\_PATHWAY\_REQUIRE\_MYC |  | 57 | 0.53 | 1.14 | 0.213 | 0.455 | 1.000 | 2839 | tags=28%, list=18%, signal=34% |
| 1384 | STEARMAN\_LUNG\_CANCER\_EARLY\_VS\_LATE\_DN |  | 55 | 0.53 | 1.14 | 0.213 | 0.456 | 1.000 | 589 | tags=13%, list=4%, signal=13% |
| 1385 | SCHEIDEREIT\_IKK\_TARGETS |  | 16 | 0.60 | 1.14 | 0.281 | 0.458 | 1.000 | 4197 | tags=69%, list=27%, signal=94% |
| 1386 | LEI\_MYB\_TARGETS |  | 295 | 0.51 | 1.14 | 0.044 | 0.458 | 1.000 | 3025 | tags=37%, list=19%, signal=45% |
| 1387 | REACTOME\_SIGNALING\_BY\_EGFR\_IN\_CANCER |  | 99 | 0.52 | 1.14 | 0.141 | 0.460 | 1.000 | 3677 | tags=36%, list=23%, signal=47% |
| 1388 | REACTOME\_PLATELET\_SENSITIZATION\_BY\_LDL |  | 16 | 0.60 | 1.14 | 0.291 | 0.460 | 1.000 | 4395 | tags=56%, list=28%, signal=78% |
| 1389 | BURTON\_ADIPOGENESIS\_4 |  | 43 | 0.55 | 1.14 | 0.216 | 0.461 | 1.000 | 2048 | tags=28%, list=13%, signal=32% |
| 1390 | LEE\_LIVER\_CANCER\_MYC\_UP |  | 51 | 0.54 | 1.14 | 0.226 | 0.464 | 1.000 | 2958 | tags=37%, list=19%, signal=46% |
| 1391 | BYSTRYKH\_HEMATOPOIESIS\_STEM\_CELL\_AND\_BRAIN\_QTL\_TRANS |  | 163 | 0.51 | 1.14 | 0.104 | 0.464 | 1.000 | 3787 | tags=37%, list=24%, signal=48% |
| 1392 | REACTOME\_REGULATION\_OF\_HYPOXIA\_INDUCIBLE\_FACTOR\_HIF\_BY\_OXYGEN |  | 23 | 0.58 | 1.14 | 0.296 | 0.465 | 1.000 | 2005 | tags=35%, list=13%, signal=40% |
| 1393 | REACTOME\_POST\_CHAPERONIN\_TUBULIN\_FOLDING\_PATHWAY |  | 15 | 0.60 | 1.14 | 0.313 | 0.465 | 1.000 | 1975 | tags=33%, list=13%, signal=38% |
| 1394 | ZHONG\_SECRETOME\_OF\_LUNG\_CANCER\_AND\_MACROPHAGE |  | 74 | 0.52 | 1.14 | 0.170 | 0.465 | 1.000 | 2779 | tags=38%, list=18%, signal=46% |
| 1395 | WEIGEL\_OXIDATIVE\_STRESS\_BY\_HNE\_AND\_H2O2 |  | 39 | 0.55 | 1.14 | 0.223 | 0.464 | 1.000 | 3860 | tags=51%, list=25%, signal=68% |
| 1396 | HOFMANN\_MYELODYSPLASTIC\_SYNDROM\_RISK\_DN |  | 17 | 0.60 | 1.14 | 0.286 | 0.464 | 1.000 | 2340 | tags=24%, list=15%, signal=28% |
| 1397 | SIG\_PIP3\_SIGNALING\_IN\_CARDIAC\_MYOCTES |  | 65 | 0.53 | 1.14 | 0.203 | 0.465 | 1.000 | 3066 | tags=34%, list=19%, signal=42% |
| 1398 | FORTSCHEGGER\_PHF8\_TARGETS\_UP |  | 219 | 0.51 | 1.14 | 0.047 | 0.468 | 1.000 | 3635 | tags=35%, list=23%, signal=44% |
| 1399 | REACTOME\_YAP1\_AND\_WWTR1\_TAZ\_STIMULATED\_GENE\_EXPRESSION |  | 22 | 0.58 | 1.14 | 0.294 | 0.469 | 1.000 | 3805 | tags=41%, list=24%, signal=54% |
| 1400 | KOYAMA\_SEMA3B\_TARGETS\_DN |  | 332 | 0.51 | 1.14 | 0.026 | 0.469 | 1.000 | 2675 | tags=24%, list=17%, signal=29% |
| 1401 | SMID\_BREAST\_CANCER\_RELAPSE\_IN\_BRAIN\_UP |  | 38 | 0.54 | 1.14 | 0.260 | 0.469 | 1.000 | 1526 | tags=18%, list=10%, signal=20% |
| 1402 | RAGHAVACHARI\_PLATELET\_SPECIFIC\_GENES |  | 62 | 0.53 | 1.14 | 0.189 | 0.470 | 1.000 | 3400 | tags=34%, list=22%, signal=43% |
| 1403 | KEGG\_NON\_SMALL\_CELL\_LUNG\_CANCER |  | 52 | 0.53 | 1.14 | 0.207 | 0.470 | 1.000 | 3666 | tags=38%, list=23%, signal=50% |
| 1404 | REACTOME\_RNA\_POL\_I\_RNA\_POL\_III\_AND\_MITOCHONDRIAL\_TRANSCRIPTION |  | 89 | 0.52 | 1.14 | 0.179 | 0.470 | 1.000 | 4184 | tags=44%, list=27%, signal=59% |
| 1405 | ST\_TUMOR\_NECROSIS\_FACTOR\_PATHWAY |  | 27 | 0.56 | 1.14 | 0.272 | 0.473 | 1.000 | 3711 | tags=52%, list=24%, signal=68% |
| 1406 | REACTOME\_CD28\_CO\_STIMULATION |  | 29 | 0.56 | 1.14 | 0.267 | 0.473 | 1.000 | 5623 | tags=62%, list=36%, signal=96% |
| 1407 | TOOKER\_GEMCITABINE\_RESISTANCE\_UP |  | 69 | 0.52 | 1.14 | 0.198 | 0.474 | 1.000 | 3029 | tags=35%, list=19%, signal=43% |
| 1408 | KOKKINAKIS\_METHIONINE\_DEPRIVATION\_96HR\_DN |  | 71 | 0.52 | 1.14 | 0.199 | 0.474 | 1.000 | 2839 | tags=39%, list=18%, signal=48% |
| 1409 | MATZUK\_MALE\_REPRODUCTION\_SERTOLI |  | 28 | 0.56 | 1.14 | 0.258 | 0.474 | 1.000 | 2868 | tags=29%, list=18%, signal=35% |
| 1410 | WANG\_IMMORTALIZED\_BY\_HOXA9\_AND\_MEIS1\_DN |  | 24 | 0.57 | 1.14 | 0.273 | 0.474 | 1.000 | 1857 | tags=17%, list=12%, signal=19% |
| 1411 | REACTOME\_PYRUVATE\_METABOLISM\_AND\_CITRIC\_ACID\_TCA\_CYCLE |  | 39 | 0.54 | 1.14 | 0.250 | 0.473 | 1.000 | 4053 | tags=51%, list=26%, signal=69% |
| 1412 | NATSUME\_RESPONSE\_TO\_INTERFERON\_BETA\_UP |  | 66 | 0.53 | 1.14 | 0.201 | 0.473 | 1.000 | 2587 | tags=24%, list=16%, signal=29% |
| 1413 | REACTOME\_TCA\_CYCLE\_AND\_RESPIRATORY\_ELECTRON\_TRANSPORT |  | 113 | 0.52 | 1.14 | 0.145 | 0.473 | 1.000 | 4053 | tags=46%, list=26%, signal=62% |
| 1414 | HINATA\_NFKB\_TARGETS\_FIBROBLAST\_UP |  | 80 | 0.52 | 1.14 | 0.181 | 0.474 | 1.000 | 2796 | tags=33%, list=18%, signal=39% |
| 1415 | DIRMEIER\_LMP1\_RESPONSE\_LATE\_DN |  | 29 | 0.56 | 1.14 | 0.274 | 0.474 | 1.000 | 3104 | tags=38%, list=20%, signal=47% |
| 1416 | BARIS\_THYROID\_CANCER\_DN |  | 56 | 0.53 | 1.14 | 0.231 | 0.475 | 1.000 | 2925 | tags=39%, list=19%, signal=48% |
| 1417 | REACTOME\_OPIOID\_SIGNALLING |  | 75 | 0.52 | 1.14 | 0.184 | 0.474 | 1.000 | 4271 | tags=33%, list=27%, signal=46% |
| 1418 | MA\_PITUITARY\_FETAL\_VS\_ADULT\_DN |  | 16 | 0.60 | 1.14 | 0.284 | 0.474 | 1.000 | 4818 | tags=63%, list=31%, signal=90% |
| 1419 | OUYANG\_PROSTATE\_CANCER\_PROGRESSION\_DN |  | 19 | 0.59 | 1.14 | 0.305 | 0.474 | 1.000 | 4310 | tags=47%, list=27%, signal=65% |
| 1420 | BIOCARTA\_CARM\_ER\_PATHWAY |  | 33 | 0.56 | 1.14 | 0.251 | 0.474 | 1.000 | 4948 | tags=55%, list=31%, signal=79% |
| 1421 | JAATINEN\_HEMATOPOIETIC\_STEM\_CELL\_UP |  | 257 | 0.51 | 1.14 | 0.051 | 0.474 | 1.000 | 3406 | tags=31%, list=22%, signal=39% |
| 1422 | RUTELLA\_RESPONSE\_TO\_CSF2RB\_AND\_IL4\_UP |  | 304 | 0.51 | 1.14 | 0.051 | 0.473 | 1.000 | 3454 | tags=33%, list=22%, signal=41% |
| 1423 | NAKAYAMA\_FRA2\_TARGETS |  | 38 | 0.54 | 1.14 | 0.265 | 0.474 | 1.000 | 4365 | tags=47%, list=28%, signal=65% |
| 1424 | LINSLEY\_MIR16\_TARGETS |  | 183 | 0.51 | 1.14 | 0.102 | 0.474 | 1.000 | 4330 | tags=45%, list=27%, signal=62% |
| 1425 | REACTOME\_SIGNAL\_TRANSDUCTION\_BY\_L1 |  | 34 | 0.55 | 1.14 | 0.256 | 0.474 | 1.000 | 3119 | tags=35%, list=20%, signal=44% |
| 1426 | PID\_INTEGRIN\_A9B1\_PATHWAY |  | 24 | 0.57 | 1.14 | 0.279 | 0.475 | 1.000 | 1523 | tags=21%, list=10%, signal=23% |
| 1427 | KUMAR\_AUTOPHAGY\_NETWORK |  | 52 | 0.54 | 1.13 | 0.243 | 0.476 | 1.000 | 1253 | tags=15%, list=8%, signal=17% |
| 1428 | SANSOM\_APC\_TARGETS\_UP |  | 112 | 0.52 | 1.13 | 0.137 | 0.476 | 1.000 | 2839 | tags=32%, list=18%, signal=39% |
| 1429 | DANG\_REGULATED\_BY\_MYC\_DN |  | 240 | 0.51 | 1.13 | 0.059 | 0.477 | 1.000 | 3035 | tags=30%, list=19%, signal=37% |
| 1430 | BILANGES\_SERUM\_SENSITIVE\_GENES |  | 80 | 0.52 | 1.13 | 0.174 | 0.477 | 1.000 | 3109 | tags=25%, list=20%, signal=31% |
| 1431 | KEGG\_PARKINSONS\_DISEASE |  | 106 | 0.51 | 1.13 | 0.149 | 0.478 | 1.000 | 4755 | tags=48%, list=30%, signal=68% |
| 1432 | AMIT\_SERUM\_RESPONSE\_480\_MCF10A |  | 32 | 0.55 | 1.13 | 0.263 | 0.478 | 1.000 | 3958 | tags=41%, list=25%, signal=54% |
| 1433 | REACTOME\_SYNTHESIS\_SECRETION\_AND\_INACTIVATION\_OF\_GLP1 |  | 17 | 0.59 | 1.13 | 0.289 | 0.478 | 1.000 | 2259 | tags=29%, list=14%, signal=34% |
| 1434 | MA\_MYELOID\_DIFFERENTIATION\_UP |  | 37 | 0.54 | 1.13 | 0.262 | 0.480 | 1.000 | 3893 | tags=51%, list=25%, signal=68% |
| 1435 | TURASHVILI\_BREAST\_LOBULAR\_CARCINOMA\_VS\_DUCTAL\_NORMAL\_UP |  | 60 | 0.53 | 1.13 | 0.231 | 0.481 | 1.000 | 3393 | tags=32%, list=22%, signal=40% |
| 1436 | MAEKAWA\_ATF2\_TARGETS |  | 22 | 0.57 | 1.13 | 0.277 | 0.480 | 1.000 | 3039 | tags=18%, list=19%, signal=22% |
| 1437 | DORN\_ADENOVIRUS\_INFECTION\_12HR\_UP |  | 26 | 0.56 | 1.13 | 0.289 | 0.482 | 1.000 | 3036 | tags=42%, list=19%, signal=52% |
| 1438 | REACTOME\_INTEGRIN\_ALPHAIIB\_BETA3\_SIGNALING |  | 27 | 0.55 | 1.13 | 0.288 | 0.483 | 1.000 | 4098 | tags=41%, list=26%, signal=55% |
| 1439 | GUO\_HEX\_TARGETS\_DN |  | 58 | 0.52 | 1.13 | 0.208 | 0.483 | 1.000 | 1916 | tags=21%, list=12%, signal=23% |
| 1440 | BIOCARTA\_FCER1\_PATHWAY |  | 37 | 0.54 | 1.13 | 0.260 | 0.483 | 1.000 | 5031 | tags=54%, list=32%, signal=79% |
| 1441 | REACTOME\_RNA\_POL\_I\_TRANSCRIPTION |  | 56 | 0.53 | 1.13 | 0.240 | 0.483 | 1.000 | 2384 | tags=29%, list=15%, signal=34% |
| 1442 | NAKAMURA\_METASTASIS |  | 40 | 0.54 | 1.13 | 0.270 | 0.484 | 1.000 | 2510 | tags=25%, list=16%, signal=30% |
| 1443 | FIRESTEIN\_CTNNB1\_PATHWAY |  | 32 | 0.55 | 1.13 | 0.262 | 0.484 | 1.000 | 2518 | tags=28%, list=16%, signal=33% |
| 1444 | VALK\_AML\_CLUSTER\_12 |  | 29 | 0.55 | 1.13 | 0.277 | 0.484 | 1.000 | 2920 | tags=28%, list=19%, signal=34% |
| 1445 | MILI\_PSEUDOPODIA\_CHEMOTAXIS\_DN |  | 410 | 0.50 | 1.13 | 0.030 | 0.484 | 1.000 | 4464 | tags=46%, list=28%, signal=63% |
| 1446 | PEPPER\_CHRONIC\_LYMPHOCYTIC\_LEUKEMIA\_UP |  | 31 | 0.55 | 1.13 | 0.287 | 0.485 | 1.000 | 2556 | tags=16%, list=16%, signal=19% |
| 1447 | IWANAGA\_CARCINOGENESIS\_BY\_KRAS\_DN |  | 105 | 0.52 | 1.13 | 0.166 | 0.487 | 1.000 | 4179 | tags=36%, list=27%, signal=49% |
| 1448 | PID\_ATF2\_PATHWAY |  | 55 | 0.53 | 1.13 | 0.241 | 0.486 | 1.000 | 4732 | tags=45%, list=30%, signal=65% |
| 1449 | HOLLEMAN\_ASPARAGINASE\_RESISTANCE\_ALL\_DN |  | 19 | 0.58 | 1.13 | 0.296 | 0.488 | 1.000 | 4916 | tags=68%, list=31%, signal=99% |
| 1450 | KORKOLA\_TERATOMA |  | 36 | 0.54 | 1.13 | 0.274 | 0.489 | 1.000 | 2384 | tags=42%, list=15%, signal=49% |
| 1451 | KYNG\_RESPONSE\_TO\_H2O2\_VIA\_ERCC6\_DN |  | 38 | 0.54 | 1.13 | 0.263 | 0.490 | 1.000 | 1554 | tags=26%, list=10%, signal=29% |
| 1452 | PID\_IGF1\_PATHWAY |  | 30 | 0.55 | 1.13 | 0.302 | 0.492 | 1.000 | 5230 | tags=60%, list=33%, signal=90% |
| 1453 | BROWNE\_HCMV\_INFECTION\_30MIN\_DN |  | 127 | 0.51 | 1.13 | 0.147 | 0.494 | 1.000 | 3768 | tags=32%, list=24%, signal=42% |
| 1454 | ZWANG\_CLASS\_2\_TRANSIENTLY\_INDUCED\_BY\_EGF |  | 40 | 0.54 | 1.13 | 0.273 | 0.495 | 1.000 | 1204 | tags=20%, list=8%, signal=22% |
| 1455 | FINAK\_BREAST\_CANCER\_SDPP\_SIGNATURE |  | 23 | 0.57 | 1.13 | 0.313 | 0.496 | 1.000 | 269 | tags=9%, list=2%, signal=9% |
| 1456 | LOPEZ\_MBD\_TARGETS\_IMPRINTED\_AND\_X\_LINKED |  | 15 | 0.60 | 1.13 | 0.312 | 0.497 | 1.000 | 1952 | tags=27%, list=12%, signal=30% |
| 1457 | ZHENG\_FOXP3\_TARGETS\_UP |  | 25 | 0.57 | 1.13 | 0.296 | 0.498 | 1.000 | 4407 | tags=40%, list=28%, signal=55% |
| 1458 | REACTOME\_INHIBITION\_OF\_VOLTAGE\_GATED\_CA2\_CHANNELS\_VIA\_GBETA\_GAMMA\_SUBUNITS |  | 25 | 0.57 | 1.13 | 0.296 | 0.499 | 1.000 | 5784 | tags=32%, list=37%, signal=50% |
| 1459 | BIOCARTA\_HDAC\_PATHWAY |  | 28 | 0.55 | 1.13 | 0.283 | 0.499 | 1.000 | 4461 | tags=39%, list=28%, signal=55% |
| 1460 | BROWNE\_HCMV\_INFECTION\_16HR\_UP |  | 200 | 0.51 | 1.13 | 0.098 | 0.499 | 1.000 | 4107 | tags=41%, list=26%, signal=55% |
| 1461 | REACTOME\_SIGNALING\_BY\_CONSTITUTIVELY\_ACTIVE\_EGFR |  | 17 | 0.59 | 1.13 | 0.295 | 0.499 | 1.000 | 4466 | tags=59%, list=28%, signal=82% |
| 1462 | QI\_PLASMACYTOMA\_DN |  | 93 | 0.51 | 1.13 | 0.184 | 0.499 | 1.000 | 2771 | tags=32%, list=18%, signal=39% |
| 1463 | KEGG\_ARGININE\_AND\_PROLINE\_METABOLISM |  | 51 | 0.53 | 1.13 | 0.246 | 0.499 | 1.000 | 2564 | tags=27%, list=16%, signal=33% |
| 1464 | SIMBULAN\_UV\_RESPONSE\_NORMAL\_DN |  | 29 | 0.55 | 1.13 | 0.297 | 0.499 | 1.000 | 3209 | tags=41%, list=20%, signal=52% |
| 1465 | LU\_TUMOR\_VASCULATURE\_UP |  | 24 | 0.56 | 1.13 | 0.313 | 0.502 | 1.000 | 1338 | tags=17%, list=8%, signal=18% |
| 1466 | WANG\_RECURRENT\_LIVER\_CANCER\_UP |  | 19 | 0.58 | 1.13 | 0.307 | 0.502 | 1.000 | 1271 | tags=37%, list=8%, signal=40% |
| 1467 | HILLION\_HMGA1\_TARGETS |  | 86 | 0.51 | 1.12 | 0.210 | 0.503 | 1.000 | 3257 | tags=33%, list=21%, signal=41% |
| 1468 | REACTOME\_LATENT\_INFECTION\_OF\_HOMO\_SAPIENS\_WITH\_MYCOBACTERIUM\_TUBERCULOSIS |  | 30 | 0.55 | 1.12 | 0.298 | 0.503 | 1.000 | 2361 | tags=20%, list=15%, signal=23% |
| 1469 | GARY\_CD5\_TARGETS\_UP |  | 421 | 0.50 | 1.12 | 0.027 | 0.504 | 1.000 | 3330 | tags=33%, list=21%, signal=41% |
| 1470 | DAZARD\_UV\_RESPONSE\_CLUSTER\_G2 |  | 26 | 0.56 | 1.12 | 0.305 | 0.505 | 1.000 | 656 | tags=15%, list=4%, signal=16% |
| 1471 | RIZKI\_TUMOR\_INVASIVENESS\_2D\_UP |  | 61 | 0.52 | 1.12 | 0.265 | 0.506 | 1.000 | 3297 | tags=34%, list=21%, signal=43% |
| 1472 | PID\_AR\_PATHWAY |  | 54 | 0.53 | 1.12 | 0.262 | 0.508 | 1.000 | 4154 | tags=48%, list=26%, signal=65% |
| 1473 | BERENJENO\_ROCK\_SIGNALING\_NOT\_VIA\_RHOA\_UP |  | 27 | 0.55 | 1.12 | 0.313 | 0.507 | 1.000 | 1712 | tags=26%, list=11%, signal=29% |
| 1474 | DORN\_ADENOVIRUS\_INFECTION\_12HR\_DN |  | 29 | 0.55 | 1.12 | 0.312 | 0.509 | 1.000 | 2938 | tags=45%, list=19%, signal=55% |
| 1475 | RODWELL\_AGING\_KIDNEY\_NO\_BLOOD\_DN |  | 126 | 0.51 | 1.12 | 0.156 | 0.510 | 1.000 | 3179 | tags=30%, list=20%, signal=37% |
| 1476 | NIKOLSKY\_BREAST\_CANCER\_6P24\_P22\_AMPLICON |  | 19 | 0.57 | 1.12 | 0.297 | 0.510 | 1.000 | 2315 | tags=26%, list=15%, signal=31% |
| 1477 | KYNG\_RESPONSE\_TO\_H2O2 |  | 64 | 0.52 | 1.12 | 0.220 | 0.511 | 1.000 | 2708 | tags=31%, list=17%, signal=38% |
| 1478 | TURASHVILI\_BREAST\_NORMAL\_DUCTAL\_VS\_LOBULAR\_UP |  | 51 | 0.53 | 1.12 | 0.261 | 0.513 | 1.000 | 4064 | tags=35%, list=26%, signal=47% |
| 1479 | YAGI\_AML\_SURVIVAL |  | 109 | 0.51 | 1.12 | 0.186 | 0.513 | 1.000 | 4658 | tags=46%, list=30%, signal=65% |
| 1480 | RASHI\_RESPONSE\_TO\_IONIZING\_RADIATION\_1 |  | 41 | 0.54 | 1.12 | 0.257 | 0.513 | 1.000 | 1951 | tags=29%, list=12%, signal=33% |
| 1481 | FERRARI\_RESPONSE\_TO\_FENRETINIDE\_UP |  | 19 | 0.58 | 1.12 | 0.307 | 0.512 | 1.000 | 1764 | tags=26%, list=11%, signal=30% |
| 1482 | PURBEY\_TARGETS\_OF\_CTBP1\_AND\_SATB1\_DN |  | 159 | 0.50 | 1.12 | 0.139 | 0.514 | 1.000 | 2528 | tags=23%, list=16%, signal=27% |
| 1483 | PID\_S1P\_S1P1\_PATHWAY |  | 20 | 0.58 | 1.12 | 0.295 | 0.514 | 1.000 | 2842 | tags=35%, list=18%, signal=43% |
| 1484 | PID\_P75NTRPATHWAY |  | 66 | 0.52 | 1.12 | 0.257 | 0.514 | 1.000 | 4725 | tags=45%, list=30%, signal=65% |
| 1485 | ZAMORA\_NOS2\_TARGETS\_DN |  | 91 | 0.51 | 1.12 | 0.198 | 0.513 | 1.000 | 2694 | tags=27%, list=17%, signal=33% |
| 1486 | SCIBETTA\_KDM5B\_TARGETS\_UP |  | 17 | 0.59 | 1.12 | 0.326 | 0.514 | 1.000 | 4390 | tags=53%, list=28%, signal=73% |
| 1487 | FAELT\_B\_CLL\_WITH\_VH\_REARRANGEMENTS\_UP |  | 42 | 0.53 | 1.12 | 0.279 | 0.515 | 1.000 | 4963 | tags=52%, list=32%, signal=76% |
| 1488 | PID\_NEPHRIN\_NEPH1\_PATHWAY |  | 31 | 0.54 | 1.12 | 0.300 | 0.516 | 1.000 | 5333 | tags=58%, list=34%, signal=88% |
| 1489 | DAIRKEE\_TERT\_TARGETS\_DN |  | 92 | 0.51 | 1.12 | 0.182 | 0.516 | 1.000 | 4591 | tags=36%, list=29%, signal=50% |
| 1490 | PID\_RHOA\_REG\_PATHWAY |  | 40 | 0.53 | 1.12 | 0.270 | 0.519 | 1.000 | 4740 | tags=43%, list=30%, signal=61% |
| 1491 | YAO\_TEMPORAL\_RESPONSE\_TO\_PROGESTERONE\_CLUSTER\_12 |  | 73 | 0.52 | 1.12 | 0.227 | 0.519 | 1.000 | 4108 | tags=47%, list=26%, signal=63% |
| 1492 | KYNG\_DNA\_DAMAGE\_BY\_4NQO\_OR\_UV |  | 60 | 0.52 | 1.12 | 0.251 | 0.520 | 1.000 | 2778 | tags=27%, list=18%, signal=32% |
| 1493 | CREIGHTON\_ENDOCRINE\_THERAPY\_RESISTANCE\_2 |  | 297 | 0.50 | 1.12 | 0.055 | 0.520 | 1.000 | 4696 | tags=32%, list=30%, signal=44% |
| 1494 | HASLINGER\_B\_CLL\_WITH\_11Q23\_DELETION |  | 21 | 0.57 | 1.12 | 0.319 | 0.520 | 1.000 | 4037 | tags=52%, list=26%, signal=70% |
| 1495 | REACTOME\_RIG\_I\_MDA5\_MEDIATED\_INDUCTION\_OF\_IFN\_ALPHA\_BETA\_PATHWAYS |  | 56 | 0.53 | 1.12 | 0.246 | 0.520 | 1.000 | 3435 | tags=34%, list=22%, signal=43% |
| 1496 | KIM\_WT1\_TARGETS\_8HR\_UP |  | 155 | 0.50 | 1.12 | 0.141 | 0.521 | 1.000 | 2125 | tags=27%, list=13%, signal=31% |
| 1497 | REACTOME\_CTNNB1\_PHOSPHORYLATION\_CASCADE |  | 16 | 0.59 | 1.12 | 0.319 | 0.521 | 1.000 | 4124 | tags=63%, list=26%, signal=85% |
| 1498 | HADDAD\_T\_LYMPHOCYTE\_AND\_NK\_PROGENITOR\_DN |  | 57 | 0.52 | 1.12 | 0.255 | 0.522 | 1.000 | 2245 | tags=16%, list=14%, signal=18% |
| 1499 | HOWLIN\_CITED1\_TARGETS\_2\_UP |  | 15 | 0.59 | 1.12 | 0.309 | 0.524 | 1.000 | 2871 | tags=40%, list=18%, signal=49% |
| 1500 | BOYAULT\_LIVER\_CANCER\_SUBCLASS\_G5\_DN |  | 19 | 0.57 | 1.12 | 0.311 | 0.525 | 1.000 | 1201 | tags=32%, list=8%, signal=34% |
| 1501 | NAKAMURA\_ADIPOGENESIS\_EARLY\_DN |  | 37 | 0.54 | 1.12 | 0.280 | 0.525 | 1.000 | 993 | tags=19%, list=6%, signal=20% |
| 1502 | BIOCARTA\_PPARA\_PATHWAY |  | 52 | 0.52 | 1.12 | 0.287 | 0.525 | 1.000 | 3484 | tags=37%, list=22%, signal=47% |
| 1503 | LEE\_AGING\_MUSCLE\_UP |  | 42 | 0.53 | 1.12 | 0.278 | 0.525 | 1.000 | 3093 | tags=33%, list=20%, signal=41% |
| 1504 | REACTOME\_NUCLEAR\_SIGNALING\_BY\_ERBB4 |  | 33 | 0.54 | 1.12 | 0.288 | 0.526 | 1.000 | 4715 | tags=42%, list=30%, signal=60% |
| 1505 | MAGRANGEAS\_MULTIPLE\_MYELOMA\_IGG\_VS\_IGA\_DN |  | 24 | 0.55 | 1.12 | 0.327 | 0.527 | 1.000 | 3605 | tags=33%, list=23%, signal=43% |
| 1506 | MARCHINI\_TRABECTEDIN\_RESISTANCE\_DN |  | 47 | 0.52 | 1.12 | 0.288 | 0.528 | 1.000 | 1776 | tags=30%, list=11%, signal=33% |
| 1507 | GARGALOVIC\_RESPONSE\_TO\_OXIDIZED\_PHOSPHOLIPIDS\_YELLOW\_DN |  | 15 | 0.59 | 1.12 | 0.334 | 0.528 | 1.000 | 4327 | tags=40%, list=27%, signal=55% |
| 1508 | KEGG\_PROSTATE\_CANCER |  | 88 | 0.51 | 1.12 | 0.221 | 0.527 | 1.000 | 5031 | tags=49%, list=32%, signal=71% |
| 1509 | KEGG\_LYSINE\_DEGRADATION |  | 44 | 0.53 | 1.12 | 0.284 | 0.528 | 1.000 | 4103 | tags=48%, list=26%, signal=64% |
| 1510 | BIOCARTA\_CERAMIDE\_PATHWAY |  | 22 | 0.56 | 1.12 | 0.328 | 0.528 | 1.000 | 4508 | tags=55%, list=29%, signal=76% |
| 1511 | DUNNE\_TARGETS\_OF\_AML1\_MTG8\_FUSION\_DN |  | 18 | 0.58 | 1.12 | 0.314 | 0.528 | 1.000 | 726 | tags=22%, list=5%, signal=23% |
| 1512 | KEGG\_PYRUVATE\_METABOLISM |  | 39 | 0.53 | 1.11 | 0.292 | 0.532 | 1.000 | 3758 | tags=44%, list=24%, signal=57% |
| 1513 | RIGGI\_EWING\_SARCOMA\_PROGENITOR\_DN |  | 160 | 0.50 | 1.11 | 0.151 | 0.532 | 1.000 | 2810 | tags=23%, list=18%, signal=28% |
| 1514 | REACTOME\_G\_BETA\_GAMMA\_SIGNALLING\_THROUGH\_PI3KGAMMA |  | 24 | 0.56 | 1.11 | 0.324 | 0.536 | 1.000 | 4271 | tags=38%, list=27%, signal=51% |
| 1515 | HUNSBERGER\_EXERCISE\_REGULATED\_GENES |  | 31 | 0.54 | 1.11 | 0.310 | 0.536 | 1.000 | 1448 | tags=19%, list=9%, signal=21% |
| 1516 | MOOTHA\_MITOCHONDRIA |  | 420 | 0.49 | 1.11 | 0.042 | 0.538 | 1.000 | 3867 | tags=41%, list=25%, signal=53% |
| 1517 | TRAYNOR\_RETT\_SYNDROM\_UP |  | 42 | 0.52 | 1.11 | 0.276 | 0.538 | 1.000 | 1235 | tags=10%, list=8%, signal=10% |
| 1518 | LINDVALL\_IMMORTALIZED\_BY\_TERT\_UP |  | 70 | 0.51 | 1.11 | 0.256 | 0.538 | 1.000 | 2017 | tags=26%, list=13%, signal=29% |
| 1519 | REACTOME\_SIGNALING\_BY\_ERBB2 |  | 91 | 0.51 | 1.11 | 0.230 | 0.538 | 1.000 | 4185 | tags=41%, list=27%, signal=55% |
| 1520 | KEGG\_RENAL\_CELL\_CARCINOMA |  | 70 | 0.51 | 1.11 | 0.234 | 0.542 | 1.000 | 5031 | tags=51%, list=32%, signal=75% |
| 1521 | NING\_CHRONIC\_OBSTRUCTIVE\_PULMONARY\_DISEASE\_DN |  | 106 | 0.51 | 1.11 | 0.211 | 0.542 | 1.000 | 3274 | tags=37%, list=21%, signal=46% |
| 1522 | KIM\_WT1\_TARGETS\_UP |  | 192 | 0.50 | 1.11 | 0.144 | 0.543 | 1.000 | 2238 | tags=25%, list=14%, signal=29% |
| 1523 | PID\_REELINPATHWAY |  | 29 | 0.55 | 1.11 | 0.323 | 0.543 | 1.000 | 4360 | tags=48%, list=28%, signal=67% |
| 1524 | PRAMOONJAGO\_SOX4\_TARGETS\_UP |  | 47 | 0.52 | 1.11 | 0.282 | 0.543 | 1.000 | 3605 | tags=34%, list=23%, signal=44% |
| 1525 | GOLUB\_ALL\_VS\_AML\_UP |  | 22 | 0.56 | 1.11 | 0.325 | 0.544 | 1.000 | 2647 | tags=45%, list=17%, signal=55% |
| 1526 | HOFFMANN\_SMALL\_PRE\_BII\_TO\_IMMATURE\_B\_LYMPHOCYTE\_DN |  | 46 | 0.52 | 1.11 | 0.267 | 0.544 | 1.000 | 4062 | tags=33%, list=26%, signal=44% |
| 1527 | LIN\_TUMOR\_ESCAPE\_FROM\_IMMUNE\_ATTACK |  | 16 | 0.58 | 1.11 | 0.327 | 0.545 | 1.000 | 2529 | tags=25%, list=16%, signal=30% |
| 1528 | ZEMBUTSU\_SENSITIVITY\_TO\_NIMUSTINE |  | 15 | 0.59 | 1.11 | 0.356 | 0.544 | 1.000 | 1959 | tags=27%, list=12%, signal=30% |
| 1529 | PID\_PDGFRBPATHWAY |  | 124 | 0.51 | 1.11 | 0.197 | 0.548 | 1.000 | 3484 | tags=36%, list=22%, signal=46% |
| 1530 | HOFMANN\_MYELODYSPLASTIC\_SYNDROM\_LOW\_RISK\_DN |  | 27 | 0.54 | 1.11 | 0.310 | 0.548 | 1.000 | 1828 | tags=33%, list=12%, signal=38% |
| 1531 | PID\_INSULIN\_GLUCOSE\_PATHWAY |  | 26 | 0.55 | 1.11 | 0.328 | 0.548 | 1.000 | 2215 | tags=31%, list=14%, signal=36% |
| 1532 | WANG\_CLIM2\_TARGETS\_UP |  | 200 | 0.50 | 1.11 | 0.136 | 0.548 | 1.000 | 2786 | tags=28%, list=18%, signal=34% |
| 1533 | REACTOME\_NGF\_SIGNALLING\_VIA\_TRKA\_FROM\_THE\_PLASMA\_MEMBRANE |  | 127 | 0.50 | 1.11 | 0.189 | 0.549 | 1.000 | 4197 | tags=41%, list=27%, signal=55% |
| 1534 | RAO\_BOUND\_BY\_SALL4 |  | 191 | 0.50 | 1.11 | 0.141 | 0.550 | 1.000 | 4387 | tags=37%, list=28%, signal=50% |
| 1535 | GAL\_LEUKEMIC\_STEM\_CELL\_DN |  | 199 | 0.50 | 1.11 | 0.135 | 0.550 | 1.000 | 2477 | tags=22%, list=16%, signal=26% |
| 1536 | BIOCARTA\_LAIR\_PATHWAY |  | 15 | 0.58 | 1.11 | 0.334 | 0.552 | 1.000 | 851 | tags=13%, list=5%, signal=14% |
| 1537 | REACTOME\_SIGNALLING\_BY\_NGF |  | 204 | 0.50 | 1.11 | 0.132 | 0.553 | 1.000 | 4740 | tags=42%, list=30%, signal=60% |
| 1538 | LIEN\_BREAST\_CARCINOMA\_METAPLASTIC\_VS\_DUCTAL\_UP |  | 74 | 0.51 | 1.11 | 0.264 | 0.552 | 1.000 | 4209 | tags=28%, list=27%, signal=39% |
| 1539 | REACTOME\_ERK\_MAPK\_TARGETS |  | 21 | 0.56 | 1.11 | 0.332 | 0.553 | 1.000 | 2810 | tags=43%, list=18%, signal=52% |
| 1540 | REACTOME\_INCRETIN\_SYNTHESIS\_SECRETION\_AND\_INACTIVATION |  | 20 | 0.57 | 1.11 | 0.337 | 0.553 | 1.000 | 2259 | tags=25%, list=14%, signal=29% |
| 1541 | REACTOME\_MAP\_KINASE\_ACTIVATION\_IN\_TLR\_CASCADE |  | 45 | 0.52 | 1.11 | 0.298 | 0.555 | 1.000 | 4820 | tags=60%, list=31%, signal=86% |
| 1542 | POTTI\_TOPOTECAN\_SENSITIVITY |  | 118 | 0.50 | 1.11 | 0.192 | 0.556 | 1.000 | 3216 | tags=33%, list=20%, signal=41% |
| 1543 | BIOCARTA\_DEATH\_PATHWAY |  | 30 | 0.53 | 1.11 | 0.323 | 0.556 | 1.000 | 3711 | tags=40%, list=24%, signal=52% |
| 1544 | BIOCARTA\_RAS\_PATHWAY |  | 23 | 0.55 | 1.11 | 0.324 | 0.558 | 1.000 | 5333 | tags=61%, list=34%, signal=92% |
| 1545 | NOUZOVA\_METHYLATED\_IN\_APL |  | 50 | 0.52 | 1.10 | 0.304 | 0.561 | 1.000 | 2535 | tags=22%, list=16%, signal=26% |
| 1546 | BIOCARTA\_FMLP\_PATHWAY |  | 35 | 0.53 | 1.10 | 0.330 | 0.561 | 1.000 | 4466 | tags=40%, list=28%, signal=56% |
| 1547 | MYLLYKANGAS\_AMPLIFICATION\_HOT\_SPOT\_23 |  | 19 | 0.56 | 1.10 | 0.359 | 0.562 | 1.000 | 3369 | tags=42%, list=21%, signal=53% |
| 1548 | KEGG\_GLYCINE\_SERINE\_AND\_THREONINE\_METABOLISM |  | 31 | 0.54 | 1.10 | 0.327 | 0.562 | 1.000 | 2654 | tags=29%, list=17%, signal=35% |
| 1549 | GENTILE\_UV\_LOW\_DOSE\_UP |  | 26 | 0.54 | 1.10 | 0.342 | 0.562 | 1.000 | 2452 | tags=35%, list=16%, signal=41% |
| 1550 | MANTOVANI\_VIRAL\_GPCR\_SIGNALING\_DN |  | 45 | 0.52 | 1.10 | 0.305 | 0.563 | 1.000 | 3167 | tags=27%, list=20%, signal=33% |
| 1551 | TARTE\_PLASMA\_CELL\_VS\_B\_LYMPHOCYTE\_UP |  | 74 | 0.51 | 1.10 | 0.272 | 0.564 | 1.000 | 2318 | tags=31%, list=15%, signal=36% |
| 1552 | WATTEL\_AUTONOMOUS\_THYROID\_ADENOMA\_DN |  | 49 | 0.52 | 1.10 | 0.301 | 0.565 | 1.000 | 2294 | tags=24%, list=15%, signal=29% |
| 1553 | LABBE\_TARGETS\_OF\_TGFB1\_AND\_WNT3A\_UP |  | 104 | 0.50 | 1.10 | 0.214 | 0.566 | 1.000 | 2082 | tags=23%, list=13%, signal=26% |
| 1554 | SANSOM\_APC\_MYC\_TARGETS |  | 184 | 0.50 | 1.10 | 0.164 | 0.565 | 1.000 | 4364 | tags=44%, list=28%, signal=60% |
| 1555 | ROSS\_AML\_OF\_FAB\_M7\_TYPE |  | 67 | 0.51 | 1.10 | 0.271 | 0.567 | 1.000 | 4009 | tags=34%, list=25%, signal=46% |
| 1556 | PID\_ERBB4\_PATHWAY |  | 37 | 0.53 | 1.10 | 0.324 | 0.568 | 1.000 | 4715 | tags=49%, list=30%, signal=69% |
| 1557 | PLASARI\_TGFB1\_TARGETS\_10HR\_DN |  | 234 | 0.49 | 1.10 | 0.136 | 0.568 | 1.000 | 2917 | tags=24%, list=19%, signal=28% |
| 1558 | AUNG\_GASTRIC\_CANCER |  | 48 | 0.52 | 1.10 | 0.282 | 0.568 | 1.000 | 2007 | tags=21%, list=13%, signal=24% |
| 1559 | BIOCARTA\_CREB\_PATHWAY |  | 25 | 0.55 | 1.10 | 0.331 | 0.569 | 1.000 | 5595 | tags=76%, list=36%, signal=118% |
| 1560 | LIU\_NASOPHARYNGEAL\_CARCINOMA |  | 61 | 0.51 | 1.10 | 0.284 | 0.569 | 1.000 | 1798 | tags=23%, list=11%, signal=26% |
| 1561 | KYNG\_ENVIRONMENTAL\_STRESS\_RESPONSE\_DN |  | 19 | 0.57 | 1.10 | 0.364 | 0.570 | 1.000 | 3701 | tags=53%, list=23%, signal=69% |
| 1562 | LIAO\_METASTASIS |  | 449 | 0.49 | 1.10 | 0.068 | 0.574 | 1.000 | 2988 | tags=30%, list=19%, signal=36% |
| 1563 | ZHAN\_MULTIPLE\_MYELOMA\_MS\_DN |  | 33 | 0.53 | 1.10 | 0.319 | 0.573 | 1.000 | 3929 | tags=48%, list=25%, signal=64% |
| 1564 | CHEMELLO\_SOLEUS\_VS\_EDL\_MYOFIBERS\_DN |  | 19 | 0.56 | 1.10 | 0.359 | 0.573 | 1.000 | 54 | tags=5%, list=0%, signal=5% |
| 1565 | BLALOCK\_ALZHEIMERS\_DISEASE\_INCIPIENT\_UP |  | 342 | 0.49 | 1.10 | 0.088 | 0.574 | 1.000 | 3808 | tags=35%, list=24%, signal=45% |
| 1566 | YOSHIOKA\_LIVER\_CANCER\_EARLY\_RECURRENCE\_DN |  | 53 | 0.52 | 1.10 | 0.309 | 0.574 | 1.000 | 3539 | tags=36%, list=22%, signal=46% |
| 1567 | GAZDA\_DIAMOND\_BLACKFAN\_ANEMIA\_ERYTHROID\_DN |  | 387 | 0.49 | 1.10 | 0.080 | 0.575 | 1.000 | 3416 | tags=30%, list=22%, signal=38% |
| 1568 | VERHAAK\_GLIOBLASTOMA\_MESENCHYMAL |  | 186 | 0.49 | 1.10 | 0.166 | 0.577 | 1.000 | 3133 | tags=29%, list=20%, signal=36% |
| 1569 | LABBE\_TGFB1\_TARGETS\_UP |  | 95 | 0.50 | 1.10 | 0.247 | 0.580 | 1.000 | 2001 | tags=23%, list=13%, signal=26% |
| 1570 | BROWNE\_HCMV\_INFECTION\_24HR\_UP |  | 138 | 0.50 | 1.10 | 0.216 | 0.584 | 1.000 | 4421 | tags=39%, list=28%, signal=54% |
| 1571 | SWEET\_KRAS\_ONCOGENIC\_SIGNATURE |  | 82 | 0.50 | 1.10 | 0.265 | 0.584 | 1.000 | 3469 | tags=45%, list=22%, signal=58% |
| 1572 | RUTELLA\_RESPONSE\_TO\_HGF\_UP |  | 363 | 0.49 | 1.10 | 0.092 | 0.585 | 1.000 | 3454 | tags=31%, list=22%, signal=39% |
| 1573 | KEGG\_REGULATION\_OF\_AUTOPHAGY |  | 25 | 0.55 | 1.10 | 0.350 | 0.588 | 1.000 | 3816 | tags=32%, list=24%, signal=42% |
| 1574 | REACTOME\_RESPIRATORY\_ELECTRON\_TRANSPORT\_ATP\_SYNTHESIS\_BY\_CHEMIOSMOTIC\_COUPLING\_AND\_HEAT\_PRODUCTION\_BY\_UNCOUPLING\_PROTEINS\_ |  | 78 | 0.50 | 1.10 | 0.283 | 0.589 | 1.000 | 3401 | tags=37%, list=22%, signal=47% |
| 1575 | SEKI\_INFLAMMATORY\_RESPONSE\_LPS\_UP |  | 70 | 0.51 | 1.10 | 0.289 | 0.588 | 1.000 | 2051 | tags=19%, list=13%, signal=21% |
| 1576 | PID\_INTEGRIN3\_PATHWAY |  | 43 | 0.52 | 1.10 | 0.321 | 0.588 | 1.000 | 2977 | tags=30%, list=19%, signal=37% |
| 1577 | ACEVEDO\_LIVER\_CANCER\_WITH\_H3K27ME3\_UP |  | 213 | 0.49 | 1.10 | 0.143 | 0.588 | 1.000 | 4444 | tags=23%, list=28%, signal=31% |
| 1578 | HOLLEMAN\_VINCRISTINE\_RESISTANCE\_B\_ALL\_UP |  | 34 | 0.52 | 1.09 | 0.342 | 0.590 | 1.000 | 5367 | tags=53%, list=34%, signal=80% |
| 1579 | LEE\_AGING\_CEREBELLUM\_DN |  | 78 | 0.51 | 1.09 | 0.298 | 0.590 | 1.000 | 3239 | tags=23%, list=21%, signal=29% |
| 1580 | PID\_CDC42\_REG\_PATHWAY |  | 29 | 0.54 | 1.09 | 0.344 | 0.590 | 1.000 | 4696 | tags=45%, list=30%, signal=64% |
| 1581 | FIGUEROA\_AML\_METHYLATION\_CLUSTER\_2\_UP |  | 45 | 0.52 | 1.09 | 0.321 | 0.591 | 1.000 | 3424 | tags=36%, list=22%, signal=45% |
| 1582 | BOGNI\_TREATMENT\_RELATED\_MYELOID\_LEUKEMIA\_UP |  | 24 | 0.55 | 1.09 | 0.352 | 0.594 | 1.000 | 2058 | tags=25%, list=13%, signal=29% |
| 1583 | SHETH\_LIVER\_CANCER\_VS\_TXNIP\_LOSS\_PAM6 |  | 40 | 0.52 | 1.09 | 0.330 | 0.594 | 1.000 | 3063 | tags=25%, list=19%, signal=31% |
| 1584 | MOOTHA\_VOXPHOS |  | 79 | 0.50 | 1.09 | 0.277 | 0.596 | 1.000 | 4755 | tags=53%, list=30%, signal=76% |
| 1585 | YAGI\_AML\_WITH\_11Q23\_REARRANGED |  | 323 | 0.48 | 1.09 | 0.122 | 0.597 | 1.000 | 3103 | tags=26%, list=20%, signal=31% |
| 1586 | PURBEY\_TARGETS\_OF\_CTBP1\_NOT\_SATB1\_DN |  | 378 | 0.49 | 1.09 | 0.089 | 0.597 | 1.000 | 3918 | tags=32%, list=25%, signal=41% |
| 1587 | KYNG\_DNA\_DAMAGE\_UP |  | 210 | 0.49 | 1.09 | 0.180 | 0.597 | 1.000 | 4600 | tags=40%, list=29%, signal=55% |
| 1588 | YOKOE\_CANCER\_TESTIS\_ANTIGENS |  | 30 | 0.53 | 1.09 | 0.348 | 0.597 | 1.000 | 1336 | tags=20%, list=8%, signal=22% |
| 1589 | AMIT\_EGF\_RESPONSE\_120\_HELA |  | 59 | 0.51 | 1.09 | 0.297 | 0.597 | 1.000 | 3922 | tags=37%, list=25%, signal=49% |
| 1590 | PID\_P53DOWNSTREAMPATHWAY |  | 122 | 0.50 | 1.09 | 0.241 | 0.598 | 1.000 | 3325 | tags=34%, list=21%, signal=43% |
| 1591 | HOFMANN\_MYELODYSPLASTIC\_SYNDROM\_RISK\_UP |  | 19 | 0.56 | 1.09 | 0.370 | 0.599 | 1.000 | 1615 | tags=26%, list=10%, signal=29% |
| 1592 | ZHANG\_RESPONSE\_TO\_IKK\_INHIBITOR\_AND\_TNF\_DN |  | 81 | 0.50 | 1.09 | 0.297 | 0.602 | 1.000 | 4305 | tags=40%, list=27%, signal=54% |
| 1593 | GAZDA\_DIAMOND\_BLACKFAN\_ANEMIA\_PROGENITOR\_UP |  | 35 | 0.53 | 1.09 | 0.352 | 0.602 | 1.000 | 2880 | tags=29%, list=18%, signal=35% |
| 1594 | FERRANDO\_T\_ALL\_WITH\_MLL\_ENL\_FUSION\_UP |  | 79 | 0.50 | 1.09 | 0.296 | 0.602 | 1.000 | 3575 | tags=29%, list=23%, signal=37% |
| 1595 | CONRAD\_STEM\_CELL |  | 36 | 0.53 | 1.09 | 0.333 | 0.603 | 1.000 | 3229 | tags=31%, list=21%, signal=38% |
| 1596 | ONO\_FOXP3\_TARGETS\_DN |  | 38 | 0.52 | 1.09 | 0.342 | 0.605 | 1.000 | 3643 | tags=29%, list=23%, signal=38% |
| 1597 | DARWICHE\_PAPILLOMA\_RISK\_HIGH\_UP |  | 123 | 0.49 | 1.09 | 0.254 | 0.606 | 1.000 | 4757 | tags=43%, list=30%, signal=61% |
| 1598 | WINTER\_HYPOXIA\_METAGENE |  | 217 | 0.49 | 1.09 | 0.176 | 0.608 | 1.000 | 2645 | tags=29%, list=17%, signal=34% |
| 1599 | ABBUD\_LIF\_SIGNALING\_1\_DN |  | 22 | 0.55 | 1.09 | 0.363 | 0.609 | 1.000 | 2947 | tags=41%, list=19%, signal=50% |
| 1600 | ST\_ERK1\_ERK2\_MAPK\_PATHWAY |  | 31 | 0.52 | 1.09 | 0.383 | 0.609 | 1.000 | 4025 | tags=45%, list=26%, signal=61% |
| 1601 | MOOTHA\_GLYCOLYSIS |  | 21 | 0.55 | 1.09 | 0.363 | 0.609 | 1.000 | 2031 | tags=29%, list=13%, signal=33% |
| 1602 | GARGALOVIC\_RESPONSE\_TO\_OXIDIZED\_PHOSPHOLIPIDS\_GREEN\_UP |  | 20 | 0.55 | 1.09 | 0.367 | 0.612 | 1.000 | 4841 | tags=65%, list=31%, signal=94% |
| 1603 | KIM\_ALL\_DISORDERS\_OLIGODENDROCYTE\_NUMBER\_CORR\_DN |  | 24 | 0.55 | 1.09 | 0.365 | 0.613 | 1.000 | 4521 | tags=38%, list=29%, signal=53% |
| 1604 | HOLLMANN\_APOPTOSIS\_VIA\_CD40\_DN |  | 222 | 0.49 | 1.09 | 0.177 | 0.613 | 1.000 | 4293 | tags=39%, list=27%, signal=53% |
| 1605 | ELVIDGE\_HIF1A\_AND\_HIF2A\_TARGETS\_UP |  | 37 | 0.52 | 1.09 | 0.350 | 0.614 | 1.000 | 2930 | tags=35%, list=19%, signal=43% |
| 1606 | BOYLAN\_MULTIPLE\_MYELOMA\_D\_UP |  | 78 | 0.50 | 1.09 | 0.315 | 0.613 | 1.000 | 3378 | tags=29%, list=21%, signal=37% |
| 1607 | JEON\_SMAD6\_TARGETS\_DN |  | 18 | 0.57 | 1.09 | 0.369 | 0.613 | 1.000 | 2366 | tags=39%, list=15%, signal=46% |
| 1608 | NAKAMURA\_METASTASIS\_MODEL\_UP |  | 38 | 0.52 | 1.09 | 0.360 | 0.615 | 1.000 | 2510 | tags=26%, list=16%, signal=31% |
| 1609 | BIOCARTA\_TCR\_PATHWAY |  | 43 | 0.52 | 1.09 | 0.357 | 0.615 | 1.000 | 5333 | tags=49%, list=34%, signal=74% |
| 1610 | NAGASHIMA\_EGF\_SIGNALING\_UP |  | 52 | 0.51 | 1.09 | 0.331 | 0.616 | 1.000 | 3573 | tags=33%, list=23%, signal=42% |
| 1611 | YORDY\_RECIPROCAL\_REGULATION\_BY\_ETS1\_AND\_SP100\_DN |  | 67 | 0.50 | 1.09 | 0.328 | 0.618 | 1.000 | 3484 | tags=31%, list=22%, signal=40% |
| 1612 | VALK\_AML\_WITH\_11Q23\_REARRANGED |  | 17 | 0.57 | 1.09 | 0.377 | 0.619 | 1.000 | 1952 | tags=24%, list=12%, signal=27% |
| 1613 | BYSTRYKH\_HEMATOPOIESIS\_STEM\_CELL\_AND\_BRAIN\_QTL\_CIS |  | 53 | 0.51 | 1.09 | 0.332 | 0.619 | 1.000 | 3749 | tags=53%, list=24%, signal=69% |
| 1614 | YANG\_BCL3\_TARGETS\_UP |  | 328 | 0.48 | 1.09 | 0.131 | 0.619 | 1.000 | 3297 | tags=26%, list=21%, signal=32% |
| 1615 | CHIANG\_LIVER\_CANCER\_SUBCLASS\_INTERFERON\_UP |  | 23 | 0.55 | 1.08 | 0.350 | 0.620 | 1.000 | 3216 | tags=22%, list=20%, signal=27% |
| 1616 | PID\_IL2\_1PATHWAY |  | 54 | 0.50 | 1.08 | 0.336 | 0.620 | 1.000 | 5041 | tags=48%, list=32%, signal=71% |
| 1617 | XU\_HGF\_SIGNALING\_NOT\_VIA\_AKT1\_48HR\_UP |  | 34 | 0.52 | 1.08 | 0.351 | 0.621 | 1.000 | 2134 | tags=26%, list=14%, signal=31% |
| 1618 | PID\_AJDISS\_2PATHWAY |  | 48 | 0.51 | 1.08 | 0.338 | 0.621 | 1.000 | 1034 | tags=21%, list=7%, signal=22% |
| 1619 | ZHAN\_MULTIPLE\_MYELOMA\_DN |  | 35 | 0.53 | 1.08 | 0.362 | 0.621 | 1.000 | 916 | tags=11%, list=6%, signal=12% |
| 1620 | SENESE\_HDAC3\_TARGETS\_DN |  | 427 | 0.48 | 1.08 | 0.098 | 0.621 | 1.000 | 2991 | tags=31%, list=19%, signal=37% |
| 1621 | BIOCARTA\_IGF1R\_PATHWAY |  | 22 | 0.54 | 1.08 | 0.375 | 0.621 | 1.000 | 5440 | tags=73%, list=35%, signal=111% |
| 1622 | GHANDHI\_DIRECT\_IRRADIATION\_UP |  | 84 | 0.50 | 1.08 | 0.299 | 0.621 | 1.000 | 3788 | tags=23%, list=24%, signal=30% |
| 1623 | GALINDO\_IMMUNE\_RESPONSE\_TO\_ENTEROTOXIN |  | 78 | 0.50 | 1.08 | 0.306 | 0.623 | 1.000 | 3209 | tags=29%, list=20%, signal=37% |
| 1624 | TIAN\_TNF\_SIGNALING\_NOT\_VIA\_NFKB |  | 21 | 0.55 | 1.08 | 0.383 | 0.625 | 1.000 | 1985 | tags=29%, list=13%, signal=33% |
| 1625 | ALFANO\_MYC\_TARGETS |  | 222 | 0.48 | 1.08 | 0.210 | 0.627 | 1.000 | 3465 | tags=35%, list=22%, signal=44% |
| 1626 | BIOCARTA\_INTEGRIN\_PATHWAY |  | 38 | 0.53 | 1.08 | 0.360 | 0.627 | 1.000 | 4466 | tags=42%, list=28%, signal=59% |
| 1627 | FAELT\_B\_CLL\_WITH\_VH3\_21\_UP |  | 40 | 0.52 | 1.08 | 0.362 | 0.633 | 1.000 | 4313 | tags=52%, list=27%, signal=72% |
| 1628 | WIELAND\_UP\_BY\_HBV\_INFECTION |  | 83 | 0.50 | 1.08 | 0.317 | 0.633 | 1.000 | 1084 | tags=19%, list=7%, signal=21% |
| 1629 | ASTON\_MAJOR\_DEPRESSIVE\_DISORDER\_DN |  | 145 | 0.49 | 1.08 | 0.263 | 0.633 | 1.000 | 4740 | tags=39%, list=30%, signal=56% |
| 1630 | REACTOME\_NEUROTRANSMITTER\_RELEASE\_CYCLE |  | 34 | 0.52 | 1.08 | 0.368 | 0.632 | 1.000 | 2920 | tags=15%, list=19%, signal=18% |
| 1631 | PID\_EPHBFWDPATHWAY |  | 40 | 0.52 | 1.08 | 0.354 | 0.633 | 1.000 | 4025 | tags=45%, list=26%, signal=60% |
| 1632 | BOYAULT\_LIVER\_CANCER\_SUBCLASS\_G12\_UP |  | 34 | 0.52 | 1.08 | 0.371 | 0.633 | 1.000 | 5050 | tags=62%, list=32%, signal=91% |
| 1633 | PID\_AVB3\_OPN\_PATHWAY |  | 31 | 0.53 | 1.08 | 0.370 | 0.633 | 1.000 | 3144 | tags=35%, list=20%, signal=44% |
| 1634 | BIOCARTA\_ECM\_PATHWAY |  | 24 | 0.53 | 1.08 | 0.404 | 0.633 | 1.000 | 3265 | tags=38%, list=21%, signal=47% |
| 1635 | WANG\_PROSTATE\_CANCER\_ANDROGEN\_INDEPENDENT |  | 56 | 0.50 | 1.08 | 0.350 | 0.634 | 1.000 | 3360 | tags=38%, list=21%, signal=48% |
| 1636 | PID\_THROMBIN\_PAR4\_PATHWAY |  | 15 | 0.57 | 1.08 | 0.380 | 0.635 | 1.000 | 1682 | tags=20%, list=11%, signal=22% |
| 1637 | PID\_NECTIN\_PATHWAY |  | 30 | 0.53 | 1.08 | 0.377 | 0.636 | 1.000 | 3265 | tags=43%, list=21%, signal=55% |
| 1638 | ASGHARZADEH\_NEUROBLASTOMA\_POOR\_SURVIVAL\_DN |  | 37 | 0.51 | 1.08 | 0.365 | 0.636 | 1.000 | 4218 | tags=32%, list=27%, signal=44% |
| 1639 | BURTON\_ADIPOGENESIS\_5 |  | 110 | 0.50 | 1.08 | 0.289 | 0.636 | 1.000 | 3253 | tags=38%, list=21%, signal=48% |
| 1640 | GROSS\_HYPOXIA\_VIA\_ELK3\_ONLY\_DN |  | 42 | 0.51 | 1.08 | 0.367 | 0.636 | 1.000 | 3592 | tags=40%, list=23%, signal=52% |
| 1641 | YE\_METASTATIC\_LIVER\_CANCER |  | 24 | 0.54 | 1.08 | 0.383 | 0.639 | 1.000 | 2625 | tags=29%, list=17%, signal=35% |
| 1642 | BIOCARTA\_GSK3\_PATHWAY |  | 27 | 0.53 | 1.08 | 0.379 | 0.639 | 1.000 | 4226 | tags=48%, list=27%, signal=66% |
| 1643 | PID\_IL1PATHWAY |  | 32 | 0.52 | 1.08 | 0.377 | 0.639 | 1.000 | 5018 | tags=50%, list=32%, signal=73% |
| 1644 | UEDA\_CENTRAL\_CLOCK |  | 80 | 0.50 | 1.08 | 0.320 | 0.638 | 1.000 | 3592 | tags=39%, list=23%, signal=50% |
| 1645 | CREIGHTON\_ENDOCRINE\_THERAPY\_RESISTANCE\_4 |  | 252 | 0.48 | 1.08 | 0.194 | 0.638 | 1.000 | 3605 | tags=30%, list=23%, signal=38% |
| 1646 | HENDRICKS\_SMARCA4\_TARGETS\_DN |  | 46 | 0.52 | 1.08 | 0.351 | 0.639 | 1.000 | 2587 | tags=26%, list=16%, signal=31% |
| 1647 | PID\_LYMPHANGIOGENESIS\_PATHWAY |  | 25 | 0.53 | 1.08 | 0.387 | 0.640 | 1.000 | 5230 | tags=64%, list=33%, signal=96% |
| 1648 | REACTOME\_PYRUVATE\_METABOLISM |  | 17 | 0.56 | 1.08 | 0.391 | 0.643 | 1.000 | 155 | tags=18%, list=1%, signal=18% |
| 1649 | WANG\_RESPONSE\_TO\_BEXAROTENE\_DN |  | 28 | 0.53 | 1.08 | 0.364 | 0.643 | 1.000 | 2634 | tags=25%, list=17%, signal=30% |
| 1650 | ACEVEDO\_LIVER\_CANCER\_WITH\_H3K9ME3\_DN |  | 74 | 0.49 | 1.08 | 0.340 | 0.645 | 1.000 | 2663 | tags=27%, list=17%, signal=32% |
| 1651 | HOWLIN\_CITED1\_TARGETS\_2\_DN |  | 16 | 0.57 | 1.08 | 0.402 | 0.645 | 1.000 | 1616 | tags=25%, list=10%, signal=28% |
| 1652 | REACTOME\_RESPIRATORY\_ELECTRON\_TRANSPORT |  | 62 | 0.50 | 1.08 | 0.327 | 0.644 | 1.000 | 3003 | tags=32%, list=19%, signal=40% |
| 1653 | JISON\_SICKLE\_CELL\_DISEASE\_UP |  | 161 | 0.48 | 1.08 | 0.258 | 0.645 | 1.000 | 3389 | tags=33%, list=22%, signal=42% |
| 1654 | REACTOME\_GLUCONEOGENESIS |  | 30 | 0.52 | 1.08 | 0.358 | 0.647 | 1.000 | 2582 | tags=37%, list=16%, signal=44% |
| 1655 | PID\_NOTCH\_PATHWAY |  | 56 | 0.50 | 1.07 | 0.344 | 0.649 | 1.000 | 3416 | tags=32%, list=22%, signal=41% |
| 1656 | COULOUARN\_TEMPORAL\_TGFB1\_SIGNATURE\_UP |  | 94 | 0.50 | 1.07 | 0.291 | 0.649 | 1.000 | 5066 | tags=53%, list=32%, signal=78% |
| 1657 | BROWNE\_HCMV\_INFECTION\_16HR\_DN |  | 74 | 0.49 | 1.07 | 0.352 | 0.650 | 1.000 | 2977 | tags=24%, list=19%, signal=30% |
| 1658 | HADDAD\_T\_LYMPHOCYTE\_AND\_NK\_PROGENITOR\_UP |  | 72 | 0.49 | 1.07 | 0.351 | 0.651 | 1.000 | 2047 | tags=19%, list=13%, signal=22% |
| 1659 | REACTOME\_STEROID\_HORMONES |  | 28 | 0.53 | 1.07 | 0.386 | 0.651 | 1.000 | 2202 | tags=7%, list=14%, signal=8% |
| 1660 | YAO\_TEMPORAL\_RESPONSE\_TO\_PROGESTERONE\_CLUSTER\_8 |  | 44 | 0.51 | 1.07 | 0.378 | 0.651 | 1.000 | 2073 | tags=34%, list=13%, signal=39% |
| 1661 | ST\_GRANULE\_CELL\_SURVIVAL\_PATHWAY |  | 26 | 0.53 | 1.07 | 0.400 | 0.651 | 1.000 | 4725 | tags=50%, list=30%, signal=71% |
| 1662 | PID\_HEDGEHOG\_GLIPATHWAY |  | 47 | 0.51 | 1.07 | 0.358 | 0.652 | 1.000 | 4295 | tags=49%, list=27%, signal=67% |
| 1663 | MORI\_PLASMA\_CELL\_DN |  | 30 | 0.52 | 1.07 | 0.381 | 0.652 | 1.000 | 1261 | tags=17%, list=8%, signal=18% |
| 1664 | REACTOME\_MEMBRANE\_TRAFFICKING |  | 115 | 0.48 | 1.07 | 0.304 | 0.654 | 1.000 | 4286 | tags=48%, list=27%, signal=65% |
| 1665 | BOWIE\_RESPONSE\_TO\_TAMOXIFEN |  | 15 | 0.57 | 1.07 | 0.389 | 0.654 | 1.000 | 3036 | tags=40%, list=19%, signal=50% |
| 1666 | FONTAINE\_THYROID\_TUMOR\_UNCERTAIN\_MALIGNANCY\_UP |  | 33 | 0.52 | 1.07 | 0.365 | 0.654 | 1.000 | 2615 | tags=30%, list=17%, signal=36% |
| 1667 | ENK\_UV\_RESPONSE\_EPIDERMIS\_UP |  | 259 | 0.48 | 1.07 | 0.206 | 0.656 | 1.000 | 3258 | tags=36%, list=21%, signal=44% |
| 1668 | REACTOME\_DOWNSTREAM\_SIGNALING\_OF\_ACTIVATED\_FGFR |  | 91 | 0.49 | 1.07 | 0.336 | 0.656 | 1.000 | 4185 | tags=32%, list=27%, signal=43% |
| 1669 | RAMPON\_ENRICHED\_LEARNING\_ENVIRONMENT\_LATE\_UP |  | 22 | 0.54 | 1.07 | 0.400 | 0.656 | 1.000 | 1871 | tags=27%, list=12%, signal=31% |
| 1670 | REACTOME\_CHOLESTEROL\_BIOSYNTHESIS |  | 21 | 0.54 | 1.07 | 0.425 | 0.657 | 1.000 | 4519 | tags=52%, list=29%, signal=73% |
| 1671 | WEST\_ADRENOCORTICAL\_CARCINOMA\_VS\_ADENOMA\_DN |  | 17 | 0.55 | 1.07 | 0.396 | 0.657 | 1.000 | 1204 | tags=24%, list=8%, signal=25% |
| 1672 | TIAN\_TNF\_SIGNALING\_VIA\_NFKB |  | 26 | 0.54 | 1.07 | 0.377 | 0.658 | 1.000 | 2073 | tags=23%, list=13%, signal=27% |
| 1673 | VALK\_AML\_CLUSTER\_3 |  | 31 | 0.53 | 1.07 | 0.384 | 0.657 | 1.000 | 3378 | tags=29%, list=21%, signal=37% |
| 1674 | KIM\_MYCN\_AMPLIFICATION\_TARGETS\_UP |  | 85 | 0.49 | 1.07 | 0.330 | 0.657 | 1.000 | 2377 | tags=16%, list=15%, signal=19% |
| 1675 | PID\_FRA\_PATHWAY |  | 35 | 0.51 | 1.07 | 0.380 | 0.657 | 1.000 | 2737 | tags=31%, list=17%, signal=38% |
| 1676 | PID\_SYNDECAN\_4\_PATHWAY |  | 32 | 0.52 | 1.07 | 0.398 | 0.657 | 1.000 | 3484 | tags=28%, list=22%, signal=36% |
| 1677 | LE\_SKI\_TARGETS\_UP |  | 17 | 0.56 | 1.07 | 0.402 | 0.657 | 1.000 | 3306 | tags=47%, list=21%, signal=59% |
| 1678 | REACTOME\_IL1\_SIGNALING |  | 34 | 0.52 | 1.07 | 0.378 | 0.657 | 1.000 | 3201 | tags=29%, list=20%, signal=37% |
| 1679 | REACTOME\_RESPONSE\_TO\_ELEVATED\_PLATELET\_CYTOSOLIC\_CA2\_ |  | 77 | 0.49 | 1.07 | 0.340 | 0.659 | 1.000 | 3484 | tags=27%, list=22%, signal=35% |
| 1680 | BIOCARTA\_IL2RB\_PATHWAY |  | 37 | 0.51 | 1.07 | 0.376 | 0.661 | 1.000 | 5230 | tags=51%, list=33%, signal=77% |
| 1681 | REACTOME\_NFKB\_AND\_MAP\_KINASES\_ACTIVATION\_MEDIATED\_BY\_TLR4\_SIGNALING\_REPERTOIRE |  | 65 | 0.50 | 1.07 | 0.335 | 0.661 | 1.000 | 4834 | tags=52%, list=31%, signal=75% |
| 1682 | TAKEDA\_TARGETS\_OF\_NUP98\_HOXA9\_FUSION\_6HR\_UP |  | 71 | 0.49 | 1.07 | 0.349 | 0.661 | 1.000 | 3979 | tags=25%, list=25%, signal=34% |
| 1683 | PID\_CDC42\_PATHWAY |  | 68 | 0.49 | 1.07 | 0.332 | 0.661 | 1.000 | 4466 | tags=49%, list=28%, signal=67% |
| 1684 | BOYLAN\_MULTIPLE\_MYELOMA\_C\_CLUSTER\_DN |  | 30 | 0.52 | 1.07 | 0.410 | 0.661 | 1.000 | 1985 | tags=17%, list=13%, signal=19% |
| 1685 | REACTOME\_SIGNALING\_BY\_FGFR\_IN\_DISEASE |  | 114 | 0.48 | 1.07 | 0.303 | 0.662 | 1.000 | 4185 | tags=33%, list=27%, signal=45% |
| 1686 | TONKS\_TARGETS\_OF\_RUNX1\_RUNX1T1\_FUSION\_MONOCYTE\_DN |  | 42 | 0.51 | 1.07 | 0.377 | 0.662 | 1.000 | 4366 | tags=38%, list=28%, signal=53% |
| 1687 | CUI\_TCF21\_TARGETS\_2\_UP |  | 374 | 0.48 | 1.07 | 0.165 | 0.662 | 1.000 | 3006 | tags=30%, list=19%, signal=36% |
| 1688 | PID\_CD8TCRPATHWAY |  | 51 | 0.51 | 1.07 | 0.381 | 0.662 | 1.000 | 5821 | tags=45%, list=37%, signal=71% |
| 1689 | MCCLUNG\_DELTA\_FOSB\_TARGETS\_8WK |  | 45 | 0.51 | 1.07 | 0.386 | 0.662 | 1.000 | 3742 | tags=33%, list=24%, signal=44% |
| 1690 | REACTOME\_METABOLISM\_OF\_STEROID\_HORMONES\_AND\_VITAMINS\_A\_AND\_D |  | 34 | 0.52 | 1.07 | 0.405 | 0.662 | 1.000 | 3493 | tags=12%, list=22%, signal=15% |
| 1691 | DAWSON\_METHYLATED\_IN\_LYMPHOMA\_TCL1 |  | 48 | 0.50 | 1.07 | 0.382 | 0.662 | 1.000 | 4208 | tags=19%, list=27%, signal=26% |
| 1692 | KEGG\_PANTOTHENATE\_AND\_COA\_BIOSYNTHESIS |  | 16 | 0.56 | 1.07 | 0.416 | 0.664 | 1.000 | 2515 | tags=19%, list=16%, signal=22% |
| 1693 | REACTOME\_EGFR\_DOWNREGULATION |  | 24 | 0.53 | 1.07 | 0.403 | 0.665 | 1.000 | 3511 | tags=42%, list=22%, signal=54% |
| 1694 | ZWANG\_EGF\_INTERVAL\_UP |  | 63 | 0.49 | 1.07 | 0.371 | 0.665 | 1.000 | 3301 | tags=22%, list=21%, signal=28% |
| 1695 | PID\_P38\_MK2PATHWAY |  | 20 | 0.54 | 1.07 | 0.411 | 0.664 | 1.000 | 3752 | tags=50%, list=24%, signal=66% |
| 1696 | MATTIOLI\_MULTIPLE\_MYELOMA\_WITH\_14Q32\_TRANSLOCATIONS |  | 34 | 0.51 | 1.07 | 0.388 | 0.666 | 1.000 | 1100 | tags=15%, list=7%, signal=16% |
| 1697 | PID\_TRAIL\_PATHWAY |  | 24 | 0.53 | 1.07 | 0.410 | 0.666 | 1.000 | 4508 | tags=54%, list=29%, signal=76% |
| 1698 | BOYAULT\_LIVER\_CANCER\_SUBCLASS\_G6\_UP |  | 56 | 0.50 | 1.07 | 0.352 | 0.665 | 1.000 | 2985 | tags=25%, list=19%, signal=31% |
| 1699 | PEDERSEN\_METASTASIS\_BY\_ERBB2\_ISOFORM\_1 |  | 42 | 0.51 | 1.07 | 0.396 | 0.665 | 1.000 | 4138 | tags=40%, list=26%, signal=55% |
| 1700 | GAZDA\_DIAMOND\_BLACKFAN\_ANEMIA\_MYELOID\_DN |  | 32 | 0.52 | 1.07 | 0.394 | 0.665 | 1.000 | 5426 | tags=59%, list=34%, signal=90% |
| 1701 | REACTOME\_SIGNALING\_BY\_FGFR |  | 102 | 0.48 | 1.07 | 0.335 | 0.666 | 1.000 | 4185 | tags=31%, list=27%, signal=42% |
| 1702 | DOANE\_RESPONSE\_TO\_ANDROGEN\_DN |  | 215 | 0.48 | 1.07 | 0.219 | 0.666 | 1.000 | 4029 | tags=38%, list=26%, signal=51% |
| 1703 | BIOCARTA\_RHO\_PATHWAY |  | 32 | 0.51 | 1.07 | 0.397 | 0.666 | 1.000 | 4313 | tags=50%, list=27%, signal=69% |
| 1704 | IVANOVSKA\_MIR106B\_TARGETS |  | 80 | 0.49 | 1.07 | 0.349 | 0.666 | 1.000 | 4602 | tags=41%, list=29%, signal=58% |
| 1705 | BAKKER\_FOXO3\_TARGETS\_DN |  | 158 | 0.48 | 1.07 | 0.283 | 0.669 | 1.000 | 3604 | tags=28%, list=23%, signal=37% |
| 1706 | REACTOME\_BRANCHED\_CHAIN\_AMINO\_ACID\_CATABOLISM |  | 17 | 0.56 | 1.07 | 0.411 | 0.669 | 1.000 | 3121 | tags=41%, list=20%, signal=51% |
| 1707 | GRANDVAUX\_IRF3\_TARGETS\_DN |  | 17 | 0.56 | 1.07 | 0.410 | 0.670 | 1.000 | 1045 | tags=24%, list=7%, signal=25% |
| 1708 | DAVICIONI\_TARGETS\_OF\_PAX\_FOXO1\_FUSIONS\_UP |  | 233 | 0.48 | 1.07 | 0.248 | 0.670 | 1.000 | 2977 | tags=27%, list=19%, signal=33% |
| 1709 | REACTOME\_GRB2\_EVENTS\_IN\_ERBB2\_SIGNALING |  | 21 | 0.54 | 1.07 | 0.413 | 0.670 | 1.000 | 3328 | tags=38%, list=21%, signal=48% |
| 1710 | YAGI\_AML\_WITH\_T\_8\_21\_TRANSLOCATION |  | 323 | 0.47 | 1.07 | 0.221 | 0.670 | 1.000 | 3322 | tags=31%, list=21%, signal=38% |
| 1711 | JI\_RESPONSE\_TO\_FSH\_UP |  | 65 | 0.49 | 1.07 | 0.366 | 0.670 | 1.000 | 3838 | tags=38%, list=24%, signal=51% |
| 1712 | JACKSON\_DNMT1\_TARGETS\_DN |  | 25 | 0.53 | 1.06 | 0.400 | 0.670 | 1.000 | 2302 | tags=28%, list=15%, signal=33% |
| 1713 | MARKS\_HDAC\_TARGETS\_UP |  | 20 | 0.55 | 1.06 | 0.404 | 0.670 | 1.000 | 2044 | tags=20%, list=13%, signal=23% |
| 1714 | WILLIAMS\_ESR1\_TARGETS\_UP |  | 26 | 0.52 | 1.06 | 0.404 | 0.672 | 1.000 | 3306 | tags=38%, list=21%, signal=49% |
| 1715 | REACTOME\_MEIOTIC\_RECOMBINATION |  | 56 | 0.50 | 1.06 | 0.411 | 0.672 | 1.000 | 3202 | tags=29%, list=20%, signal=36% |
| 1716 | AMIT\_EGF\_RESPONSE\_40\_HELA |  | 39 | 0.51 | 1.06 | 0.397 | 0.673 | 1.000 | 1985 | tags=23%, list=13%, signal=26% |
| 1717 | MURAKAMI\_UV\_RESPONSE\_6HR\_UP |  | 32 | 0.52 | 1.06 | 0.387 | 0.674 | 1.000 | 2946 | tags=41%, list=19%, signal=50% |
| 1718 | REACTOME\_SEMAPHORIN\_INTERACTIONS |  | 62 | 0.49 | 1.06 | 0.372 | 0.676 | 1.000 | 1034 | tags=16%, list=7%, signal=17% |
| 1719 | MALONEY\_RESPONSE\_TO\_17AAG\_UP |  | 36 | 0.51 | 1.06 | 0.397 | 0.678 | 1.000 | 1656 | tags=22%, list=11%, signal=25% |
| 1720 | NAKAMURA\_ADIPOGENESIS\_LATE\_DN |  | 35 | 0.51 | 1.06 | 0.400 | 0.678 | 1.000 | 993 | tags=17%, list=6%, signal=18% |
| 1721 | HORTON\_SREBF\_TARGETS |  | 25 | 0.53 | 1.06 | 0.419 | 0.678 | 1.000 | 4519 | tags=52%, list=29%, signal=73% |
| 1722 | KEGG\_DRUG\_METABOLISM\_OTHER\_ENZYMES |  | 39 | 0.51 | 1.06 | 0.403 | 0.679 | 1.000 | 1161 | tags=15%, list=7%, signal=17% |
| 1723 | REACTOME\_AXON\_GUIDANCE |  | 235 | 0.48 | 1.06 | 0.250 | 0.679 | 1.000 | 3335 | tags=25%, list=21%, signal=31% |
| 1724 | KEGG\_VASOPRESSIN\_REGULATED\_WATER\_REABSORPTION |  | 43 | 0.50 | 1.06 | 0.402 | 0.679 | 1.000 | 4954 | tags=44%, list=31%, signal=64% |
| 1725 | PID\_RAC1\_PATHWAY |  | 53 | 0.49 | 1.06 | 0.396 | 0.679 | 1.000 | 2297 | tags=34%, list=15%, signal=40% |
| 1726 | KOKKINAKIS\_METHIONINE\_DEPRIVATION\_96HR\_UP |  | 113 | 0.48 | 1.06 | 0.336 | 0.679 | 1.000 | 3182 | tags=35%, list=20%, signal=44% |
| 1727 | SCHURINGA\_STAT5A\_TARGETS\_UP |  | 19 | 0.55 | 1.06 | 0.423 | 0.680 | 1.000 | 946 | tags=11%, list=6%, signal=11% |
| 1728 | HOSHIDA\_LIVER\_CANCER\_SUBCLASS\_S1 |  | 226 | 0.48 | 1.06 | 0.259 | 0.680 | 1.000 | 2586 | tags=28%, list=16%, signal=33% |
| 1729 | GUILLAUMOND\_KLF10\_TARGETS\_DN |  | 26 | 0.53 | 1.06 | 0.398 | 0.681 | 1.000 | 2798 | tags=35%, list=18%, signal=42% |
| 1730 | PEDERSEN\_METASTASIS\_BY\_ERBB2\_ISOFORM\_7 |  | 334 | 0.47 | 1.06 | 0.215 | 0.681 | 1.000 | 3560 | tags=31%, list=23%, signal=39% |
| 1731 | LUND\_SILENCED\_BY\_METHYLATION |  | 16 | 0.56 | 1.06 | 0.417 | 0.682 | 1.000 | 2134 | tags=44%, list=14%, signal=51% |
| 1732 | KEGG\_BLADDER\_CANCER |  | 40 | 0.50 | 1.06 | 0.407 | 0.682 | 1.000 | 3189 | tags=38%, list=20%, signal=47% |
| 1733 | AIYAR\_COBRA1\_TARGETS\_DN |  | 23 | 0.54 | 1.06 | 0.409 | 0.683 | 1.000 | 1485 | tags=26%, list=9%, signal=29% |
| 1734 | GAUSSMANN\_MLL\_AF4\_FUSION\_TARGETS\_C\_UP |  | 151 | 0.48 | 1.06 | 0.304 | 0.682 | 1.000 | 3357 | tags=26%, list=21%, signal=33% |
| 1735 | GRUETZMANN\_PANCREATIC\_CANCER\_DN |  | 185 | 0.48 | 1.06 | 0.294 | 0.683 | 1.000 | 3025 | tags=29%, list=19%, signal=36% |
| 1736 | MCCLUNG\_DELTA\_FOSB\_TARGETS\_2WK |  | 41 | 0.51 | 1.06 | 0.403 | 0.687 | 1.000 | 3051 | tags=20%, list=19%, signal=24% |
| 1737 | MULLIGHAN\_MLL\_SIGNATURE\_2\_DN |  | 238 | 0.47 | 1.06 | 0.264 | 0.688 | 1.000 | 2982 | tags=30%, list=19%, signal=36% |
| 1738 | KORKOLA\_YOLK\_SAC\_TUMOR\_UP |  | 18 | 0.55 | 1.06 | 0.420 | 0.688 | 1.000 | 4222 | tags=50%, list=27%, signal=68% |
| 1739 | SIG\_IL4RECEPTOR\_IN\_B\_LYPHOCYTES |  | 27 | 0.52 | 1.06 | 0.408 | 0.690 | 1.000 | 5230 | tags=63%, list=33%, signal=94% |
| 1740 | REACTOME\_MEIOTIC\_SYNAPSIS |  | 53 | 0.50 | 1.06 | 0.407 | 0.691 | 1.000 | 3215 | tags=28%, list=20%, signal=35% |
| 1741 | KORKOLA\_EMBRYONIC\_CARCINOMA\_VS\_SEMINOMA\_DN |  | 22 | 0.54 | 1.06 | 0.431 | 0.692 | 1.000 | 2959 | tags=27%, list=19%, signal=34% |
| 1742 | LEE\_AGING\_CEREBELLUM\_UP |  | 79 | 0.49 | 1.06 | 0.377 | 0.692 | 1.000 | 3297 | tags=33%, list=21%, signal=41% |
| 1743 | KAMIKUBO\_MYELOID\_MN1\_NETWORK |  | 18 | 0.55 | 1.06 | 0.409 | 0.692 | 1.000 | 3364 | tags=50%, list=21%, signal=64% |
| 1744 | REACTOME\_MUSCLE\_CONTRACTION |  | 45 | 0.50 | 1.06 | 0.409 | 0.692 | 1.000 | 993 | tags=16%, list=6%, signal=17% |
| 1745 | REACTOME\_DOWNSTREAM\_SIGNAL\_TRANSDUCTION |  | 85 | 0.48 | 1.06 | 0.372 | 0.692 | 1.000 | 4594 | tags=42%, list=29%, signal=59% |
| 1746 | FARMER\_BREAST\_CANCER\_APOCRINE\_VS\_BASAL |  | 291 | 0.47 | 1.06 | 0.250 | 0.691 | 1.000 | 3193 | tags=33%, list=20%, signal=40% |
| 1747 | FOSTER\_TOLERANT\_MACROPHAGE\_DN |  | 362 | 0.47 | 1.06 | 0.226 | 0.691 | 1.000 | 3519 | tags=32%, list=22%, signal=40% |
| 1748 | KENNY\_CTNNB1\_TARGETS\_DN |  | 42 | 0.50 | 1.06 | 0.416 | 0.691 | 1.000 | 1801 | tags=29%, list=11%, signal=32% |
| 1749 | REACTOME\_SHC\_RELATED\_EVENTS |  | 16 | 0.55 | 1.06 | 0.442 | 0.692 | 1.000 | 5230 | tags=56%, list=33%, signal=84% |
| 1750 | LIU\_SOX4\_TARGETS\_UP |  | 118 | 0.48 | 1.06 | 0.320 | 0.692 | 1.000 | 4083 | tags=42%, list=26%, signal=56% |
| 1751 | ROZANOV\_MMP14\_TARGETS\_UP |  | 227 | 0.47 | 1.06 | 0.266 | 0.692 | 1.000 | 2110 | tags=19%, list=13%, signal=22% |
| 1752 | KEGG\_TERPENOID\_BACKBONE\_BIOSYNTHESIS |  | 15 | 0.56 | 1.06 | 0.438 | 0.692 | 1.000 | 4519 | tags=60%, list=29%, signal=84% |
| 1753 | KEGG\_ALZHEIMERS\_DISEASE |  | 151 | 0.47 | 1.06 | 0.305 | 0.693 | 1.000 | 4755 | tags=41%, list=30%, signal=58% |
| 1754 | GROSS\_HYPOXIA\_VIA\_ELK3\_ONLY\_UP |  | 33 | 0.51 | 1.06 | 0.411 | 0.693 | 1.000 | 3491 | tags=36%, list=22%, signal=47% |
| 1755 | CAIRO\_HEPATOBLASTOMA\_POOR\_SURVIVAL |  | 15 | 0.56 | 1.06 | 0.444 | 0.693 | 1.000 | 2294 | tags=27%, list=15%, signal=31% |
| 1756 | RASHI\_RESPONSE\_TO\_IONIZING\_RADIATION\_5 |  | 134 | 0.48 | 1.06 | 0.330 | 0.693 | 1.000 | 5122 | tags=50%, list=33%, signal=73% |
| 1757 | VALK\_AML\_CLUSTER\_13 |  | 25 | 0.52 | 1.06 | 0.409 | 0.693 | 1.000 | 788 | tags=8%, list=5%, signal=8% |
| 1758 | REACTOME\_MHC\_CLASS\_II\_ANTIGEN\_PRESENTATION |  | 84 | 0.48 | 1.05 | 0.366 | 0.696 | 1.000 | 2178 | tags=31%, list=14%, signal=36% |
| 1759 | KOKKINAKIS\_METHIONINE\_DEPRIVATION\_48HR\_UP |  | 121 | 0.48 | 1.05 | 0.360 | 0.699 | 1.000 | 3069 | tags=36%, list=19%, signal=44% |
| 1760 | REACTOME\_SMOOTH\_MUSCLE\_CONTRACTION |  | 22 | 0.53 | 1.05 | 0.441 | 0.699 | 1.000 | 993 | tags=27%, list=6%, signal=29% |
| 1761 | REACTOME\_PLATELET\_AGGREGATION\_PLUG\_FORMATION |  | 36 | 0.51 | 1.05 | 0.413 | 0.699 | 1.000 | 4098 | tags=31%, list=26%, signal=41% |
| 1762 | NATSUME\_RESPONSE\_TO\_INTERFERON\_BETA\_DN |  | 47 | 0.50 | 1.05 | 0.408 | 0.700 | 1.000 | 2625 | tags=32%, list=17%, signal=38% |
| 1763 | MURAKAMI\_UV\_RESPONSE\_24HR |  | 19 | 0.54 | 1.05 | 0.419 | 0.702 | 1.000 | 2387 | tags=26%, list=15%, signal=31% |
| 1764 | REACTOME\_TRAF6\_MEDIATED\_INDUCTION\_OF\_NFKB\_AND\_MAP\_KINASES\_UPON\_TLR7\_8\_OR\_9\_ACTIVATION |  | 69 | 0.49 | 1.05 | 0.399 | 0.702 | 1.000 | 4820 | tags=46%, list=31%, signal=67% |
| 1765 | KEGG\_RIG\_I\_LIKE\_RECEPTOR\_SIGNALING\_PATHWAY |  | 55 | 0.49 | 1.05 | 0.404 | 0.702 | 1.000 | 4749 | tags=45%, list=30%, signal=65% |
| 1766 | REACTOME\_GLUTAMATE\_NEUROTRANSMITTER\_RELEASE\_CYCLE |  | 15 | 0.56 | 1.05 | 0.444 | 0.702 | 1.000 | 2920 | tags=20%, list=19%, signal=25% |
| 1767 | SMID\_BREAST\_CANCER\_RELAPSE\_IN\_PLEURA\_DN |  | 18 | 0.54 | 1.05 | 0.424 | 0.702 | 1.000 | 3184 | tags=17%, list=20%, signal=21% |
| 1768 | PASQUALUCCI\_LYMPHOMA\_BY\_GC\_STAGE\_UP |  | 251 | 0.47 | 1.05 | 0.289 | 0.703 | 1.000 | 2978 | tags=28%, list=19%, signal=34% |
| 1769 | BROWNE\_INTERFERON\_RESPONSIVE\_GENES |  | 55 | 0.50 | 1.05 | 0.406 | 0.705 | 1.000 | 3836 | tags=31%, list=24%, signal=41% |
| 1770 | STOSSI\_RESPONSE\_TO\_ESTRADIOL |  | 43 | 0.50 | 1.05 | 0.418 | 0.705 | 1.000 | 1971 | tags=19%, list=13%, signal=21% |
| 1771 | MCBRYAN\_PUBERTAL\_BREAST\_6\_7WK\_UP |  | 178 | 0.47 | 1.05 | 0.320 | 0.705 | 1.000 | 3550 | tags=35%, list=23%, signal=45% |
| 1772 | SA\_PTEN\_PATHWAY |  | 16 | 0.55 | 1.05 | 0.420 | 0.706 | 1.000 | 4098 | tags=56%, list=26%, signal=76% |
| 1773 | RIZKI\_TUMOR\_INVASIVENESS\_3D\_UP |  | 190 | 0.47 | 1.05 | 0.316 | 0.707 | 1.000 | 3929 | tags=31%, list=25%, signal=40% |
| 1774 | BIOCARTA\_WNT\_PATHWAY |  | 25 | 0.53 | 1.05 | 0.430 | 0.709 | 1.000 | 4113 | tags=48%, list=26%, signal=65% |
| 1775 | REACTOME\_NEUROTRANSMITTER\_RECEPTOR\_BINDING\_AND\_DOWNSTREAM\_TRANSMISSION\_IN\_THE\_POSTSYNAPTIC\_CELL |  | 131 | 0.47 | 1.05 | 0.356 | 0.714 | 1.000 | 3484 | tags=17%, list=22%, signal=21% |
| 1776 | BIOCARTA\_HCMV\_PATHWAY |  | 17 | 0.55 | 1.05 | 0.431 | 0.714 | 1.000 | 4395 | tags=53%, list=28%, signal=73% |
| 1777 | GREENBAUM\_E2A\_TARGETS\_DN |  | 16 | 0.55 | 1.05 | 0.453 | 0.715 | 1.000 | 1002 | tags=25%, list=6%, signal=27% |
| 1778 | PID\_EPOPATHWAY |  | 34 | 0.51 | 1.05 | 0.420 | 0.715 | 1.000 | 5230 | tags=53%, list=33%, signal=79% |
| 1779 | KANG\_FLUOROURACIL\_RESISTANCE\_UP |  | 21 | 0.53 | 1.05 | 0.441 | 0.716 | 1.000 | 4539 | tags=62%, list=29%, signal=87% |
| 1780 | REACTOME\_MYD88\_MAL\_CASCADE\_INITIATED\_ON\_PLASMA\_MEMBRANE |  | 75 | 0.48 | 1.05 | 0.405 | 0.720 | 1.000 | 4834 | tags=47%, list=31%, signal=67% |
| 1781 | CADWELL\_ATG16L1\_TARGETS\_DN |  | 54 | 0.49 | 1.05 | 0.409 | 0.720 | 1.000 | 2044 | tags=22%, list=13%, signal=25% |
| 1782 | KIM\_MYCL1\_AMPLIFICATION\_TARGETS\_DN |  | 17 | 0.56 | 1.05 | 0.450 | 0.720 | 1.000 | 1503 | tags=35%, list=10%, signal=39% |
| 1783 | BROWNE\_HCMV\_INFECTION\_10HR\_UP |  | 87 | 0.48 | 1.05 | 0.399 | 0.719 | 1.000 | 4918 | tags=45%, list=31%, signal=65% |
| 1784 | KYNG\_ENVIRONMENTAL\_STRESS\_RESPONSE\_NOT\_BY\_GAMMA\_IN\_WS |  | 32 | 0.51 | 1.05 | 0.428 | 0.720 | 1.000 | 3218 | tags=41%, list=20%, signal=51% |
| 1785 | DARWICHE\_SKIN\_TUMOR\_PROMOTER\_DN |  | 148 | 0.47 | 1.05 | 0.358 | 0.722 | 1.000 | 2400 | tags=22%, list=15%, signal=25% |
| 1786 | BIOCARTA\_MET\_PATHWAY |  | 37 | 0.51 | 1.05 | 0.432 | 0.723 | 1.000 | 5031 | tags=54%, list=32%, signal=79% |
| 1787 | REACTOME\_NETRIN1\_SIGNALING |  | 37 | 0.50 | 1.05 | 0.447 | 0.722 | 1.000 | 4986 | tags=38%, list=32%, signal=55% |
| 1788 | WANG\_BARRETTS\_ESOPHAGUS\_DN |  | 25 | 0.52 | 1.05 | 0.423 | 0.722 | 1.000 | 2779 | tags=36%, list=18%, signal=44% |
| 1789 | BOYLAN\_MULTIPLE\_MYELOMA\_D\_CLUSTER\_DN |  | 37 | 0.50 | 1.05 | 0.440 | 0.723 | 1.000 | 3242 | tags=35%, list=21%, signal=44% |
| 1790 | DASU\_IL6\_SIGNALING\_SCAR\_UP |  | 28 | 0.52 | 1.05 | 0.455 | 0.724 | 1.000 | 2266 | tags=25%, list=14%, signal=29% |
| 1791 | UEDA\_PERIFERAL\_CLOCK |  | 146 | 0.47 | 1.05 | 0.355 | 0.726 | 1.000 | 2792 | tags=32%, list=18%, signal=38% |
| 1792 | PID\_AMB2\_NEUTROPHILS\_PATHWAY |  | 40 | 0.50 | 1.05 | 0.411 | 0.726 | 1.000 | 3265 | tags=30%, list=21%, signal=38% |
| 1793 | LAMB\_CCND1\_TARGETS |  | 19 | 0.54 | 1.04 | 0.451 | 0.726 | 1.000 | 2164 | tags=37%, list=14%, signal=43% |
| 1794 | PID\_GLYPICAN\_1PATHWAY |  | 27 | 0.52 | 1.04 | 0.442 | 0.726 | 1.000 | 1957 | tags=19%, list=12%, signal=21% |
| 1795 | PID\_ER\_NONGENOMIC\_PATHWAY |  | 41 | 0.50 | 1.04 | 0.434 | 0.727 | 1.000 | 4482 | tags=46%, list=28%, signal=65% |
| 1796 | BIOCARTA\_SHH\_PATHWAY |  | 15 | 0.56 | 1.04 | 0.446 | 0.727 | 1.000 | 4194 | tags=47%, list=27%, signal=64% |
| 1797 | LEIN\_MEDULLA\_MARKERS |  | 73 | 0.48 | 1.04 | 0.393 | 0.729 | 1.000 | 3185 | tags=26%, list=20%, signal=32% |
| 1798 | HAHTOLA\_CTCL\_CUTANEOUS |  | 24 | 0.52 | 1.04 | 0.445 | 0.733 | 1.000 | 1129 | tags=25%, list=7%, signal=27% |
| 1799 | IWANAGA\_CARCINOGENESIS\_BY\_KRAS\_UP |  | 142 | 0.47 | 1.04 | 0.377 | 0.733 | 1.000 | 2634 | tags=21%, list=17%, signal=25% |
| 1800 | PID\_NETRIN\_PATHWAY |  | 31 | 0.51 | 1.04 | 0.447 | 0.733 | 1.000 | 5018 | tags=52%, list=32%, signal=76% |
| 1801 | REACTOME\_SIGNALING\_BY\_RHO\_GTPASES |  | 98 | 0.48 | 1.04 | 0.409 | 0.733 | 1.000 | 4740 | tags=37%, list=30%, signal=52% |
| 1802 | BIOCARTA\_PYK2\_PATHWAY |  | 27 | 0.52 | 1.04 | 0.452 | 0.732 | 1.000 | 5333 | tags=63%, list=34%, signal=95% |
| 1803 | MCCABE\_HOXC6\_TARGETS\_DN |  | 20 | 0.53 | 1.04 | 0.447 | 0.733 | 1.000 | 2291 | tags=20%, list=15%, signal=23% |
| 1804 | DAZARD\_RESPONSE\_TO\_UV\_NHEK\_UP |  | 211 | 0.47 | 1.04 | 0.341 | 0.733 | 1.000 | 3726 | tags=36%, list=24%, signal=46% |
| 1805 | BROWNE\_HCMV\_INFECTION\_12HR\_UP |  | 93 | 0.48 | 1.04 | 0.414 | 0.732 | 1.000 | 3511 | tags=28%, list=22%, signal=36% |
| 1806 | RATTENBACHER\_BOUND\_BY\_CELF1 |  | 336 | 0.46 | 1.04 | 0.299 | 0.733 | 1.000 | 4388 | tags=35%, list=28%, signal=48% |
| 1807 | PID\_AVB3\_INTEGRIN\_PATHWAY |  | 73 | 0.49 | 1.04 | 0.422 | 0.733 | 1.000 | 3296 | tags=30%, list=21%, signal=38% |
| 1808 | BIOCARTA\_BCR\_PATHWAY |  | 33 | 0.51 | 1.04 | 0.430 | 0.734 | 1.000 | 5333 | tags=55%, list=34%, signal=82% |
| 1809 | FLECHNER\_BIOPSY\_KIDNEY\_TRANSPLANT\_OK\_VS\_DONOR\_DN |  | 23 | 0.52 | 1.04 | 0.450 | 0.733 | 1.000 | 2991 | tags=48%, list=19%, signal=59% |
| 1810 | GAUSSMANN\_MLL\_AF4\_FUSION\_TARGETS\_E\_DN |  | 21 | 0.53 | 1.04 | 0.449 | 0.734 | 1.000 | 2656 | tags=29%, list=17%, signal=34% |
| 1811 | GUILLAUMOND\_KLF10\_TARGETS\_UP |  | 48 | 0.49 | 1.04 | 0.439 | 0.735 | 1.000 | 1852 | tags=17%, list=12%, signal=19% |
| 1812 | TERAO\_AOX4\_TARGETS\_HG\_UP |  | 24 | 0.52 | 1.04 | 0.447 | 0.736 | 1.000 | 5031 | tags=58%, list=32%, signal=86% |
| 1813 | WEIGEL\_OXIDATIVE\_STRESS\_BY\_TBH\_AND\_H2O2 |  | 33 | 0.50 | 1.04 | 0.445 | 0.737 | 1.000 | 2340 | tags=39%, list=15%, signal=46% |
| 1814 | GROSS\_HYPOXIA\_VIA\_HIF1A\_DN |  | 98 | 0.47 | 1.04 | 0.422 | 0.736 | 1.000 | 3258 | tags=32%, list=21%, signal=40% |
| 1815 | PID\_ECADHERIN\_STABILIZATION\_PATHWAY |  | 41 | 0.49 | 1.04 | 0.432 | 0.738 | 1.000 | 3428 | tags=37%, list=22%, signal=47% |
| 1816 | GOTTWEIN\_TARGETS\_OF\_KSHV\_MIR\_K12\_11 |  | 56 | 0.48 | 1.04 | 0.440 | 0.742 | 1.000 | 4498 | tags=45%, list=29%, signal=62% |
| 1817 | KEGG\_GLYCOLYSIS\_GLUCONEOGENESIS |  | 56 | 0.49 | 1.04 | 0.421 | 0.743 | 1.000 | 2670 | tags=25%, list=17%, signal=30% |
| 1818 | JEPSEN\_SMRT\_TARGETS |  | 31 | 0.51 | 1.04 | 0.460 | 0.743 | 1.000 | 2911 | tags=35%, list=18%, signal=43% |
| 1819 | PID\_BCR\_5PATHWAY |  | 65 | 0.48 | 1.04 | 0.445 | 0.743 | 1.000 | 5410 | tags=52%, list=34%, signal=79% |
| 1820 | PID\_FCER1PATHWAY |  | 59 | 0.48 | 1.04 | 0.426 | 0.742 | 1.000 | 4466 | tags=37%, list=28%, signal=52% |
| 1821 | DELASERNA\_MYOD\_TARGETS\_DN |  | 54 | 0.49 | 1.04 | 0.440 | 0.742 | 1.000 | 2869 | tags=35%, list=18%, signal=43% |
| 1822 | KATSANOU\_ELAVL1\_TARGETS\_DN |  | 131 | 0.47 | 1.04 | 0.386 | 0.743 | 1.000 | 3661 | tags=31%, list=23%, signal=40% |
| 1823 | BIOCARTA\_HSP27\_PATHWAY |  | 15 | 0.55 | 1.04 | 0.469 | 0.744 | 1.000 | 931 | tags=13%, list=6%, signal=14% |
| 1824 | KEGG\_OXIDATIVE\_PHOSPHORYLATION |  | 113 | 0.47 | 1.04 | 0.419 | 0.744 | 1.000 | 4755 | tags=43%, list=30%, signal=62% |
| 1825 | GRAHAM\_CML\_QUIESCENT\_VS\_NORMAL\_DIVIDING\_UP |  | 48 | 0.49 | 1.04 | 0.444 | 0.744 | 1.000 | 2723 | tags=21%, list=17%, signal=25% |
| 1826 | SUZUKI\_RESPONSE\_TO\_TSA\_AND\_DECITABINE\_1A |  | 20 | 0.53 | 1.04 | 0.466 | 0.743 | 1.000 | 1741 | tags=15%, list=11%, signal=17% |
| 1827 | BENNETT\_SYSTEMIC\_LUPUS\_ERYTHEMATOSUS |  | 25 | 0.51 | 1.04 | 0.446 | 0.744 | 1.000 | 2452 | tags=24%, list=16%, signal=28% |
| 1828 | HWANG\_PROSTATE\_CANCER\_MARKERS |  | 26 | 0.51 | 1.04 | 0.469 | 0.744 | 1.000 | 1449 | tags=27%, list=9%, signal=30% |
| 1829 | KEGG\_CYTOSOLIC\_DNA\_SENSING\_PATHWAY |  | 44 | 0.49 | 1.04 | 0.440 | 0.744 | 1.000 | 4487 | tags=43%, list=28%, signal=60% |
| 1830 | WEIGEL\_OXIDATIVE\_STRESS\_RESPONSE |  | 32 | 0.50 | 1.04 | 0.466 | 0.747 | 1.000 | 3561 | tags=31%, list=23%, signal=40% |
| 1831 | HOSHIDA\_LIVER\_CANCER\_SURVIVAL\_DN |  | 105 | 0.47 | 1.04 | 0.416 | 0.750 | 1.000 | 3945 | tags=34%, list=25%, signal=45% |
| 1832 | LEE\_CALORIE\_RESTRICTION\_NEOCORTEX\_UP |  | 78 | 0.47 | 1.04 | 0.420 | 0.750 | 1.000 | 2427 | tags=23%, list=15%, signal=27% |
| 1833 | PID\_DELTANP63PATHWAY |  | 46 | 0.49 | 1.04 | 0.444 | 0.752 | 1.000 | 4223 | tags=33%, list=27%, signal=44% |
| 1834 | BROCKE\_APOPTOSIS\_REVERSED\_BY\_IL6 |  | 122 | 0.47 | 1.04 | 0.409 | 0.753 | 1.000 | 3597 | tags=30%, list=23%, signal=38% |
| 1835 | KEEN\_RESPONSE\_TO\_ROSIGLITAZONE\_DN |  | 97 | 0.47 | 1.03 | 0.429 | 0.754 | 1.000 | 2082 | tags=26%, list=13%, signal=30% |
| 1836 | REACTOME\_GAB1\_SIGNALOSOME |  | 33 | 0.50 | 1.03 | 0.471 | 0.754 | 1.000 | 4185 | tags=39%, list=27%, signal=54% |
| 1837 | LIN\_MELANOMA\_COPY\_NUMBER\_UP |  | 62 | 0.49 | 1.03 | 0.449 | 0.758 | 1.000 | 4930 | tags=55%, list=31%, signal=80% |
| 1838 | REACTOME\_IRON\_UPTAKE\_AND\_TRANSPORT |  | 35 | 0.50 | 1.03 | 0.457 | 0.757 | 1.000 | 4676 | tags=40%, list=30%, signal=57% |
| 1839 | KEGG\_PATHWAYS\_IN\_CANCER |  | 317 | 0.46 | 1.03 | 0.363 | 0.757 | 1.000 | 3328 | tags=26%, list=21%, signal=33% |
| 1840 | GARGALOVIC\_RESPONSE\_TO\_OXIDIZED\_PHOSPHOLIPIDS\_BLACK\_UP |  | 32 | 0.50 | 1.03 | 0.472 | 0.758 | 1.000 | 2573 | tags=28%, list=16%, signal=34% |
| 1841 | DAVICIONI\_MOLECULAR\_ARMS\_VS\_ERMS\_UP |  | 287 | 0.46 | 1.03 | 0.347 | 0.759 | 1.000 | 4745 | tags=39%, list=30%, signal=55% |
| 1842 | ST\_GA13\_PATHWAY |  | 33 | 0.50 | 1.03 | 0.474 | 0.759 | 1.000 | 3201 | tags=36%, list=20%, signal=46% |
| 1843 | KEGG\_T\_CELL\_RECEPTOR\_SIGNALING\_PATHWAY |  | 107 | 0.47 | 1.03 | 0.426 | 0.759 | 1.000 | 3417 | tags=24%, list=22%, signal=31% |
| 1844 | YAN\_ESCAPE\_FROM\_ANOIKIS |  | 22 | 0.52 | 1.03 | 0.460 | 0.760 | 1.000 | 1010 | tags=23%, list=6%, signal=24% |
| 1845 | REACTOME\_TRIF\_MEDIATED\_TLR3\_SIGNALING |  | 67 | 0.47 | 1.03 | 0.435 | 0.760 | 1.000 | 4395 | tags=45%, list=28%, signal=62% |
| 1846 | FONTAINE\_PAPILLARY\_THYROID\_CARCINOMA\_UP |  | 59 | 0.49 | 1.03 | 0.448 | 0.761 | 1.000 | 2810 | tags=27%, list=18%, signal=33% |
| 1847 | PARENT\_MTOR\_SIGNALING\_DN |  | 41 | 0.49 | 1.03 | 0.450 | 0.761 | 1.000 | 3159 | tags=27%, list=20%, signal=33% |
| 1848 | FERRANDO\_LYL1\_NEIGHBORS |  | 15 | 0.55 | 1.03 | 0.471 | 0.761 | 1.000 | 1563 | tags=20%, list=10%, signal=22% |
| 1849 | ZHAN\_MULTIPLE\_MYELOMA\_MF\_DN |  | 32 | 0.51 | 1.03 | 0.455 | 0.762 | 1.000 | 4645 | tags=38%, list=29%, signal=53% |
| 1850 | MARTINEZ\_RB1\_TARGETS\_DN |  | 479 | 0.46 | 1.03 | 0.315 | 0.762 | 1.000 | 3520 | tags=28%, list=22%, signal=35% |
| 1851 | KEGG\_STEROID\_BIOSYNTHESIS |  | 16 | 0.55 | 1.03 | 0.458 | 0.761 | 1.000 | 2284 | tags=31%, list=15%, signal=37% |
| 1852 | KYNG\_WERNER\_SYNDROM\_DN |  | 21 | 0.52 | 1.03 | 0.432 | 0.761 | 1.000 | 4264 | tags=33%, list=27%, signal=46% |
| 1853 | PID\_WNT\_CANONICAL\_PATHWAY |  | 19 | 0.53 | 1.03 | 0.464 | 0.761 | 1.000 | 4272 | tags=47%, list=27%, signal=65% |
| 1854 | PID\_TOLL\_ENDOGENOUS\_PATHWAY |  | 24 | 0.52 | 1.03 | 0.466 | 0.761 | 1.000 | 956 | tags=17%, list=6%, signal=18% |
| 1855 | TORCHIA\_TARGETS\_OF\_EWSR1\_FLI1\_FUSION\_TOP20\_DN |  | 17 | 0.54 | 1.03 | 0.474 | 0.762 | 1.000 | 2779 | tags=29%, list=18%, signal=36% |
| 1856 | SA\_MMP\_CYTOKINE\_CONNECTION |  | 15 | 0.55 | 1.03 | 0.449 | 0.763 | 1.000 | 891 | tags=13%, list=6%, signal=14% |
| 1857 | HARRIS\_HYPOXIA |  | 78 | 0.47 | 1.03 | 0.436 | 0.764 | 1.000 | 2165 | tags=24%, list=14%, signal=28% |
| 1858 | RASHI\_RESPONSE\_TO\_IONIZING\_RADIATION\_3 |  | 46 | 0.49 | 1.03 | 0.464 | 0.765 | 1.000 | 4518 | tags=48%, list=29%, signal=67% |
| 1859 | NAKAJIMA\_MAST\_CELL |  | 41 | 0.49 | 1.03 | 0.470 | 0.764 | 1.000 | 2844 | tags=29%, list=18%, signal=36% |
| 1860 | PID\_PDGFRAPATHWAY |  | 22 | 0.52 | 1.03 | 0.457 | 0.765 | 1.000 | 4031 | tags=50%, list=26%, signal=67% |
| 1861 | REACTOME\_PLC\_BETA\_MEDIATED\_EVENTS |  | 40 | 0.50 | 1.03 | 0.461 | 0.764 | 1.000 | 4208 | tags=33%, list=27%, signal=44% |
| 1862 | VALK\_AML\_CLUSTER\_15 |  | 27 | 0.51 | 1.03 | 0.489 | 0.765 | 1.000 | 4268 | tags=48%, list=27%, signal=66% |
| 1863 | NIKOLSKY\_OVERCONNECTED\_IN\_BREAST\_CANCER |  | 19 | 0.53 | 1.03 | 0.484 | 0.765 | 1.000 | 5476 | tags=37%, list=35%, signal=56% |
| 1864 | KEGG\_VIBRIO\_CHOLERAE\_INFECTION |  | 51 | 0.48 | 1.03 | 0.456 | 0.764 | 1.000 | 2582 | tags=24%, list=16%, signal=28% |
| 1865 | FRASOR\_TAMOXIFEN\_RESPONSE\_UP |  | 44 | 0.49 | 1.03 | 0.466 | 0.764 | 1.000 | 4402 | tags=45%, list=28%, signal=63% |
| 1866 | CHANGOLKAR\_H2AFY\_TARGETS\_DN |  | 35 | 0.50 | 1.03 | 0.473 | 0.764 | 1.000 | 4022 | tags=37%, list=26%, signal=50% |
| 1867 | STAMBOLSKY\_TARGETS\_OF\_MUTATED\_TP53\_UP |  | 42 | 0.49 | 1.03 | 0.489 | 0.765 | 1.000 | 4511 | tags=40%, list=29%, signal=57% |
| 1868 | LIN\_NPAS4\_TARGETS\_UP |  | 133 | 0.47 | 1.03 | 0.424 | 0.765 | 1.000 | 4029 | tags=35%, list=26%, signal=46% |
| 1869 | DING\_LUNG\_CANCER\_MUTATED\_SIGNIFICANTLY |  | 26 | 0.52 | 1.03 | 0.460 | 0.766 | 1.000 | 3284 | tags=23%, list=21%, signal=29% |
| 1870 | GENTLES\_LEUKEMIC\_STEM\_CELL\_UP |  | 24 | 0.51 | 1.03 | 0.460 | 0.766 | 1.000 | 2171 | tags=13%, list=14%, signal=14% |
| 1871 | BYSTRYKH\_HEMATOPOIESIS\_STEM\_CELL\_QTL\_CIS |  | 111 | 0.47 | 1.03 | 0.427 | 0.766 | 1.000 | 4424 | tags=49%, list=28%, signal=67% |
| 1872 | WEIGEL\_OXIDATIVE\_STRESS\_BY\_HNE\_AND\_TBH |  | 55 | 0.48 | 1.03 | 0.456 | 0.768 | 1.000 | 3197 | tags=38%, list=20%, signal=48% |
| 1873 | ZWANG\_CLASS\_1\_TRANSIENTLY\_INDUCED\_BY\_EGF |  | 420 | 0.46 | 1.03 | 0.370 | 0.771 | 1.000 | 4434 | tags=35%, list=28%, signal=47% |
| 1874 | KORKOLA\_EMBRYONIC\_CARCINOMA\_VS\_SEMINOMA\_UP |  | 21 | 0.52 | 1.03 | 0.475 | 0.770 | 1.000 | 1004 | tags=19%, list=6%, signal=20% |
| 1875 | PID\_MYC\_REPRESSPATHWAY |  | 61 | 0.48 | 1.03 | 0.458 | 0.770 | 1.000 | 3230 | tags=39%, list=21%, signal=49% |
| 1876 | LU\_EZH2\_TARGETS\_UP |  | 215 | 0.46 | 1.03 | 0.416 | 0.772 | 1.000 | 4003 | tags=36%, list=25%, signal=48% |
| 1877 | ZHENG\_FOXP3\_TARGETS\_IN\_T\_LYMPHOCYTE\_DN |  | 33 | 0.50 | 1.03 | 0.469 | 0.773 | 1.000 | 4429 | tags=36%, list=28%, signal=50% |
| 1878 | MARTORIATI\_MDM4\_TARGETS\_FETAL\_LIVER\_UP |  | 192 | 0.46 | 1.03 | 0.403 | 0.773 | 1.000 | 4206 | tags=41%, list=27%, signal=55% |
| 1879 | FIGUEROA\_AML\_METHYLATION\_CLUSTER\_3\_UP |  | 137 | 0.46 | 1.03 | 0.437 | 0.774 | 1.000 | 4692 | tags=35%, list=30%, signal=49% |
| 1880 | PID\_ANGIOPOIETINRECEPTOR\_PATHWAY |  | 50 | 0.48 | 1.03 | 0.479 | 0.774 | 1.000 | 5362 | tags=48%, list=34%, signal=73% |
| 1881 | FLOTHO\_PEDIATRIC\_ALL\_THERAPY\_RESPONSE\_DN |  | 23 | 0.52 | 1.02 | 0.492 | 0.778 | 1.000 | 4306 | tags=39%, list=27%, signal=54% |
| 1882 | SYED\_ESTRADIOL\_RESPONSE |  | 18 | 0.53 | 1.02 | 0.468 | 0.779 | 1.000 | 2326 | tags=44%, list=15%, signal=52% |
| 1883 | AMIT\_EGF\_RESPONSE\_480\_HELA |  | 144 | 0.46 | 1.02 | 0.438 | 0.779 | 1.000 | 2953 | tags=30%, list=19%, signal=36% |
| 1884 | LEIN\_LOCALIZED\_TO\_PROXIMAL\_DENDRITES |  | 33 | 0.49 | 1.02 | 0.481 | 0.779 | 1.000 | 2951 | tags=24%, list=19%, signal=30% |
| 1885 | LUI\_THYROID\_CANCER\_CLUSTER\_1 |  | 43 | 0.48 | 1.02 | 0.474 | 0.779 | 1.000 | 2071 | tags=26%, list=13%, signal=29% |
| 1886 | REACTOME\_SHC\_MEDIATED\_CASCADE |  | 28 | 0.51 | 1.02 | 0.480 | 0.780 | 1.000 | 137 | tags=7%, list=1%, signal=7% |
| 1887 | CHEN\_LUNG\_CANCER\_SURVIVAL |  | 25 | 0.51 | 1.02 | 0.482 | 0.780 | 1.000 | 1815 | tags=36%, list=12%, signal=41% |
| 1888 | LAIHO\_COLORECTAL\_CANCER\_SERRATED\_DN |  | 65 | 0.47 | 1.02 | 0.473 | 0.780 | 1.000 | 3342 | tags=35%, list=21%, signal=45% |
| 1889 | BILD\_E2F3\_ONCOGENIC\_SIGNATURE |  | 200 | 0.46 | 1.02 | 0.419 | 0.782 | 1.000 | 3685 | tags=30%, list=23%, signal=39% |
| 1890 | KRIEG\_HYPOXIA\_VIA\_KDM3A |  | 47 | 0.48 | 1.02 | 0.472 | 0.782 | 1.000 | 2529 | tags=30%, list=16%, signal=35% |
| 1891 | REACTOME\_TRANSCRIPTIONAL\_REGULATION\_OF\_WHITE\_ADIPOCYTE\_DIFFERENTIATION |  | 54 | 0.48 | 1.02 | 0.464 | 0.783 | 1.000 | 2799 | tags=22%, list=18%, signal=27% |
| 1892 | NOJIMA\_SFRP2\_TARGETS\_UP |  | 28 | 0.50 | 1.02 | 0.472 | 0.783 | 1.000 | 1809 | tags=21%, list=11%, signal=24% |
| 1893 | ONO\_AML1\_TARGETS\_UP |  | 23 | 0.51 | 1.02 | 0.477 | 0.783 | 1.000 | 1172 | tags=13%, list=7%, signal=14% |
| 1894 | REACTOME\_DEVELOPMENTAL\_BIOLOGY |  | 356 | 0.46 | 1.02 | 0.383 | 0.784 | 1.000 | 3335 | tags=24%, list=21%, signal=30% |
| 1895 | MCCABE\_BOUND\_BY\_HOXC6 |  | 328 | 0.46 | 1.02 | 0.393 | 0.784 | 1.000 | 5118 | tags=34%, list=32%, signal=49% |
| 1896 | SIG\_REGULATION\_OF\_THE\_ACTIN\_CYTOSKELETON\_BY\_RHO\_GTPASES |  | 35 | 0.50 | 1.02 | 0.479 | 0.785 | 1.000 | 4098 | tags=49%, list=26%, signal=66% |
| 1897 | PID\_ERBB1\_RECEPTOR\_PROXIMAL\_PATHWAY |  | 35 | 0.49 | 1.02 | 0.499 | 0.785 | 1.000 | 4025 | tags=43%, list=26%, signal=57% |
| 1898 | WENG\_POR\_TARGETS\_LIVER\_UP |  | 36 | 0.49 | 1.02 | 0.483 | 0.786 | 1.000 | 2134 | tags=31%, list=14%, signal=35% |
| 1899 | PID\_CXCR3PATHWAY |  | 41 | 0.49 | 1.02 | 0.480 | 0.785 | 1.000 | 4466 | tags=39%, list=28%, signal=54% |
| 1900 | FARMER\_BREAST\_CANCER\_CLUSTER\_1 |  | 34 | 0.49 | 1.02 | 0.494 | 0.791 | 1.000 | 2871 | tags=15%, list=18%, signal=18% |
| 1901 | GAUSSMANN\_MLL\_AF4\_FUSION\_TARGETS\_C\_DN |  | 18 | 0.53 | 1.02 | 0.489 | 0.790 | 1.000 | 3301 | tags=33%, list=21%, signal=42% |
| 1902 | WANG\_BARRETTS\_ESOPHAGUS\_AND\_ESOPHAGUS\_CANCER\_DN |  | 35 | 0.49 | 1.02 | 0.494 | 0.791 | 1.000 | 1684 | tags=20%, list=11%, signal=22% |
| 1903 | SANSOM\_APC\_TARGETS |  | 172 | 0.46 | 1.02 | 0.429 | 0.791 | 1.000 | 3182 | tags=27%, list=20%, signal=34% |
| 1904 | GINESTIER\_BREAST\_CANCER\_ZNF217\_AMPLIFIED\_DN |  | 260 | 0.46 | 1.02 | 0.405 | 0.792 | 1.000 | 4508 | tags=39%, list=29%, signal=54% |
| 1905 | KEGG\_EPITHELIAL\_CELL\_SIGNALING\_IN\_HELICOBACTER\_PYLORI\_INFECTION |  | 64 | 0.47 | 1.02 | 0.474 | 0.792 | 1.000 | 4430 | tags=41%, list=28%, signal=56% |
| 1906 | JAZAERI\_BREAST\_CANCER\_BRCA1\_VS\_BRCA2\_DN |  | 37 | 0.49 | 1.02 | 0.503 | 0.793 | 1.000 | 3926 | tags=32%, list=25%, signal=43% |
| 1907 | REACTOME\_CIRCADIAN\_CLOCK |  | 47 | 0.48 | 1.02 | 0.486 | 0.794 | 1.000 | 4553 | tags=36%, list=29%, signal=51% |
| 1908 | PEDERSEN\_TARGETS\_OF\_611CTF\_ISOFORM\_OF\_ERBB2 |  | 69 | 0.47 | 1.02 | 0.484 | 0.794 | 1.000 | 1993 | tags=22%, list=13%, signal=25% |
| 1909 | PID\_PI3KCIPATHWAY |  | 42 | 0.49 | 1.02 | 0.485 | 0.793 | 1.000 | 3666 | tags=31%, list=23%, signal=40% |
| 1910 | REACTOME\_GLUCOSE\_METABOLISM |  | 62 | 0.48 | 1.02 | 0.469 | 0.794 | 1.000 | 4206 | tags=47%, list=27%, signal=64% |
| 1911 | NIKOLSKY\_BREAST\_CANCER\_22Q13\_AMPLICON |  | 15 | 0.54 | 1.02 | 0.499 | 0.795 | 1.000 | 5372 | tags=53%, list=34%, signal=81% |
| 1912 | KEGG\_ANTIGEN\_PROCESSING\_AND\_PRESENTATION |  | 54 | 0.48 | 1.02 | 0.490 | 0.795 | 1.000 | 1904 | tags=24%, list=12%, signal=27% |
| 1913 | TONKS\_TARGETS\_OF\_RUNX1\_RUNX1T1\_FUSION\_SUSTAINED\_IN\_MONOCYTE\_UP |  | 20 | 0.52 | 1.02 | 0.476 | 0.795 | 1.000 | 3922 | tags=40%, list=25%, signal=53% |
| 1914 | PID\_BETACATENIN\_DEG\_PATHWAY |  | 17 | 0.53 | 1.02 | 0.492 | 0.796 | 1.000 | 4113 | tags=53%, list=26%, signal=72% |
| 1915 | BIOCARTA\_NKCELLS\_PATHWAY |  | 19 | 0.52 | 1.02 | 0.495 | 0.798 | 1.000 | 879 | tags=11%, list=6%, signal=11% |
| 1916 | MITSIADES\_RESPONSE\_TO\_APLIDIN\_UP |  | 369 | 0.45 | 1.02 | 0.411 | 0.798 | 1.000 | 4248 | tags=35%, list=27%, signal=46% |
| 1917 | ABE\_VEGFA\_TARGETS\_2HR |  | 31 | 0.49 | 1.02 | 0.499 | 0.798 | 1.000 | 2855 | tags=32%, list=18%, signal=39% |
| 1918 | NAKAMURA\_ADIPOGENESIS\_LATE\_UP |  | 94 | 0.47 | 1.02 | 0.472 | 0.799 | 1.000 | 3303 | tags=32%, list=21%, signal=40% |
| 1919 | MCCABE\_HOXC6\_TARGETS\_CANCER\_DN |  | 18 | 0.53 | 1.02 | 0.509 | 0.799 | 1.000 | 2328 | tags=33%, list=15%, signal=39% |
| 1920 | SIG\_BCR\_SIGNALING\_PATHWAY |  | 46 | 0.48 | 1.02 | 0.488 | 0.799 | 1.000 | 5410 | tags=46%, list=34%, signal=69% |
| 1921 | HEIDENBLAD\_AMPLIFIED\_IN\_PANCREATIC\_CANCER |  | 27 | 0.50 | 1.02 | 0.494 | 0.799 | 1.000 | 5322 | tags=63%, list=34%, signal=95% |
| 1922 | OUYANG\_PROSTATE\_CANCER\_MARKERS |  | 19 | 0.52 | 1.02 | 0.504 | 0.799 | 1.000 | 2577 | tags=42%, list=16%, signal=50% |
| 1923 | NAGASHIMA\_NRG1\_SIGNALING\_DN |  | 48 | 0.48 | 1.02 | 0.489 | 0.800 | 1.000 | 3650 | tags=38%, list=23%, signal=49% |
| 1924 | TAVOR\_CEBPA\_TARGETS\_DN |  | 29 | 0.50 | 1.02 | 0.515 | 0.800 | 1.000 | 3786 | tags=41%, list=24%, signal=54% |
| 1925 | HERNANDEZ\_MITOTIC\_ARREST\_BY\_DOCETAXEL\_2\_UP |  | 55 | 0.47 | 1.02 | 0.478 | 0.800 | 1.000 | 3209 | tags=24%, list=20%, signal=30% |
| 1926 | HU\_GENOTOXIN\_ACTION\_DIRECT\_VS\_INDIRECT\_4HR |  | 33 | 0.50 | 1.01 | 0.506 | 0.802 | 1.000 | 2873 | tags=24%, list=18%, signal=30% |
| 1927 | REACTOME\_ACTIVATED\_TLR4\_SIGNALLING |  | 85 | 0.46 | 1.01 | 0.486 | 0.803 | 1.000 | 4834 | tags=44%, list=31%, signal=62% |
| 1928 | LU\_IL4\_SIGNALING |  | 84 | 0.47 | 1.01 | 0.501 | 0.805 | 1.000 | 2994 | tags=23%, list=19%, signal=28% |
| 1929 | NIELSEN\_MALIGNAT\_FIBROUS\_HISTIOCYTOMA\_UP |  | 17 | 0.53 | 1.01 | 0.491 | 0.806 | 1.000 | 1751 | tags=18%, list=11%, signal=20% |
| 1930 | HEIDENBLAD\_AMPLICON\_12P11\_12\_UP |  | 30 | 0.50 | 1.01 | 0.508 | 0.806 | 1.000 | 2731 | tags=30%, list=17%, signal=36% |
| 1931 | STEINER\_ERYTHROCYTE\_MEMBRANE\_GENES |  | 15 | 0.54 | 1.01 | 0.498 | 0.806 | 1.000 | 3322 | tags=20%, list=21%, signal=25% |
| 1932 | KEGG\_ENDOMETRIAL\_CANCER |  | 52 | 0.48 | 1.01 | 0.486 | 0.806 | 1.000 | 5031 | tags=52%, list=32%, signal=76% |
| 1933 | PILON\_KLF1\_TARGETS\_UP |  | 445 | 0.45 | 1.01 | 0.421 | 0.808 | 1.000 | 2996 | tags=24%, list=19%, signal=29% |
| 1934 | KEGG\_TGF\_BETA\_SIGNALING\_PATHWAY |  | 84 | 0.46 | 1.01 | 0.500 | 0.808 | 1.000 | 3673 | tags=33%, list=23%, signal=43% |
| 1935 | MOOTHA\_GLYCOGEN\_METABOLISM |  | 19 | 0.52 | 1.01 | 0.485 | 0.808 | 1.000 | 4206 | tags=42%, list=27%, signal=57% |
| 1936 | REACTOME\_INTEGRIN\_CELL\_SURFACE\_INTERACTIONS |  | 78 | 0.47 | 1.01 | 0.494 | 0.808 | 1.000 | 3450 | tags=24%, list=22%, signal=31% |
| 1937 | POTTI\_5FU\_SENSITIVITY |  | 35 | 0.49 | 1.01 | 0.513 | 0.808 | 1.000 | 2383 | tags=29%, list=15%, signal=34% |
| 1938 | RICKMAN\_HEAD\_AND\_NECK\_CANCER\_C |  | 86 | 0.46 | 1.01 | 0.471 | 0.808 | 1.000 | 1865 | tags=9%, list=12%, signal=10% |
| 1939 | ACEVEDO\_LIVER\_CANCER\_WITH\_H3K27ME3\_DN |  | 158 | 0.46 | 1.01 | 0.495 | 0.808 | 1.000 | 3364 | tags=24%, list=21%, signal=30% |
| 1940 | ZHANG\_ANTIVIRAL\_RESPONSE\_TO\_RIBAVIRIN\_UP |  | 27 | 0.50 | 1.01 | 0.506 | 0.808 | 1.000 | 2775 | tags=30%, list=18%, signal=36% |
| 1941 | DAIRKEE\_CANCER\_PRONE\_RESPONSE\_E2 |  | 27 | 0.50 | 1.01 | 0.512 | 0.808 | 1.000 | 2586 | tags=30%, list=16%, signal=35% |
| 1942 | LINDVALL\_IMMORTALIZED\_BY\_TERT\_DN |  | 72 | 0.47 | 1.01 | 0.489 | 0.808 | 1.000 | 3216 | tags=31%, list=20%, signal=38% |
| 1943 | IVANOVA\_HEMATOPOIESIS\_MATURE\_CELL |  | 243 | 0.45 | 1.01 | 0.454 | 0.808 | 1.000 | 4117 | tags=35%, list=26%, signal=47% |
| 1944 | REACTOME\_PI3K\_EVENTS\_IN\_ERBB2\_SIGNALING |  | 39 | 0.48 | 1.01 | 0.493 | 0.809 | 1.000 | 4185 | tags=41%, list=27%, signal=56% |
| 1945 | REACTOME\_METABOLISM\_OF\_CARBOHYDRATES |  | 218 | 0.45 | 1.01 | 0.478 | 0.809 | 1.000 | 4206 | tags=36%, list=27%, signal=48% |
| 1946 | BOYLAN\_MULTIPLE\_MYELOMA\_PCA3\_DN |  | 59 | 0.47 | 1.01 | 0.500 | 0.809 | 1.000 | 3646 | tags=31%, list=23%, signal=40% |
| 1947 | TSAI\_RESPONSE\_TO\_IONIZING\_RADIATION |  | 138 | 0.46 | 1.01 | 0.485 | 0.810 | 1.000 | 4282 | tags=41%, list=27%, signal=55% |
| 1948 | RIZ\_ERYTHROID\_DIFFERENTIATION\_HEMGN |  | 30 | 0.49 | 1.01 | 0.505 | 0.810 | 1.000 | 2925 | tags=17%, list=19%, signal=20% |
| 1949 | REACTOME\_FRS2\_MEDIATED\_CASCADE |  | 35 | 0.49 | 1.01 | 0.499 | 0.811 | 1.000 | 2668 | tags=17%, list=17%, signal=21% |
| 1950 | REACTOME\_PI\_3K\_CASCADE |  | 51 | 0.48 | 1.01 | 0.503 | 0.811 | 1.000 | 4185 | tags=29%, list=27%, signal=40% |
| 1951 | REACTOME\_NRAGE\_SIGNALS\_DEATH\_THROUGH\_JNK |  | 41 | 0.48 | 1.01 | 0.506 | 0.813 | 1.000 | 4740 | tags=34%, list=30%, signal=49% |
| 1952 | RIZKI\_TUMOR\_INVASIVENESS\_2D\_DN |  | 59 | 0.47 | 1.01 | 0.509 | 0.813 | 1.000 | 2791 | tags=31%, list=18%, signal=37% |
| 1953 | MASSARWEH\_RESPONSE\_TO\_ESTRADIOL |  | 55 | 0.47 | 1.01 | 0.499 | 0.813 | 1.000 | 4878 | tags=36%, list=31%, signal=52% |
| 1954 | ZWANG\_DOWN\_BY\_2ND\_EGF\_PULSE |  | 139 | 0.46 | 1.01 | 0.477 | 0.812 | 1.000 | 5986 | tags=46%, list=38%, signal=74% |
| 1955 | MENSE\_HYPOXIA\_UP |  | 80 | 0.46 | 1.01 | 0.498 | 0.813 | 1.000 | 5011 | tags=48%, list=32%, signal=69% |
| 1956 | REACTOME\_GENERIC\_TRANSCRIPTION\_PATHWAY |  | 194 | 0.46 | 1.01 | 0.469 | 0.813 | 1.000 | 4866 | tags=36%, list=31%, signal=52% |
| 1957 | CAVARD\_LIVER\_CANCER\_MALIGNANT\_VS\_BENIGN |  | 24 | 0.50 | 1.01 | 0.506 | 0.817 | 1.000 | 2211 | tags=21%, list=14%, signal=24% |
| 1958 | PID\_ALPHASYNUCLEIN\_PATHWAY |  | 33 | 0.49 | 1.01 | 0.530 | 0.817 | 1.000 | 2587 | tags=18%, list=16%, signal=22% |
| 1959 | NAGASHIMA\_NRG1\_SIGNALING\_UP |  | 159 | 0.46 | 1.01 | 0.487 | 0.817 | 1.000 | 3760 | tags=30%, list=24%, signal=38% |
| 1960 | PID\_PS1PATHWAY |  | 44 | 0.48 | 1.01 | 0.507 | 0.817 | 1.000 | 2587 | tags=30%, list=16%, signal=35% |
| 1961 | PID\_MAPKTRKPATHWAY |  | 34 | 0.49 | 1.01 | 0.516 | 0.817 | 1.000 | 3345 | tags=32%, list=21%, signal=41% |
| 1962 | BIOCARTA\_AKT\_PATHWAY |  | 21 | 0.52 | 1.01 | 0.494 | 0.816 | 1.000 | 3450 | tags=33%, list=22%, signal=43% |
| 1963 | REACTOME\_G\_PROTEIN\_BETA\_GAMMA\_SIGNALLING |  | 27 | 0.50 | 1.01 | 0.504 | 0.817 | 1.000 | 4271 | tags=37%, list=27%, signal=51% |
| 1964 | AMIT\_EGF\_RESPONSE\_240\_MCF10A |  | 17 | 0.52 | 1.01 | 0.514 | 0.819 | 1.000 | 3542 | tags=35%, list=22%, signal=45% |
| 1965 | SMITH\_TERT\_TARGETS\_DN |  | 83 | 0.46 | 1.01 | 0.498 | 0.818 | 1.000 | 3179 | tags=33%, list=20%, signal=41% |
| 1966 | SMIRNOV\_RESPONSE\_TO\_IR\_2HR\_DN |  | 49 | 0.47 | 1.01 | 0.506 | 0.820 | 1.000 | 5096 | tags=41%, list=32%, signal=60% |
| 1967 | REACTOME\_DAG\_AND\_IP3\_SIGNALING |  | 29 | 0.50 | 1.01 | 0.537 | 0.822 | 1.000 | 3830 | tags=31%, list=24%, signal=41% |
| 1968 | FONTAINE\_THYROID\_TUMOR\_UNCERTAIN\_MALIGNANCY\_DN |  | 25 | 0.50 | 1.01 | 0.514 | 0.823 | 1.000 | 3197 | tags=24%, list=20%, signal=30% |
| 1969 | REACTOME\_GABA\_SYNTHESIS\_RELEASE\_REUPTAKE\_AND\_DEGRADATION |  | 17 | 0.53 | 1.01 | 0.524 | 0.823 | 1.000 | 2920 | tags=18%, list=19%, signal=22% |
| 1970 | PID\_TNFPATHWAY |  | 44 | 0.47 | 1.01 | 0.508 | 0.824 | 1.000 | 5081 | tags=52%, list=32%, signal=77% |
| 1971 | BIOCARTA\_IGF1\_PATHWAY |  | 21 | 0.51 | 1.01 | 0.512 | 0.824 | 1.000 | 5230 | tags=67%, list=33%, signal=100% |
| 1972 | WIKMAN\_ASBESTOS\_LUNG\_CANCER\_DN |  | 26 | 0.50 | 1.01 | 0.504 | 0.823 | 1.000 | 907 | tags=15%, list=6%, signal=16% |
| 1973 | DACOSTA\_UV\_RESPONSE\_VIA\_ERCC3\_COMMON\_UP |  | 69 | 0.46 | 1.00 | 0.516 | 0.823 | 1.000 | 3758 | tags=35%, list=24%, signal=45% |
| 1974 | HOSHIDA\_LIVER\_CANCER\_SURVIVAL\_UP |  | 71 | 0.47 | 1.00 | 0.503 | 0.824 | 1.000 | 3219 | tags=32%, list=20%, signal=41% |
| 1975 | REACTOME\_GLYCOLYSIS |  | 27 | 0.49 | 1.00 | 0.523 | 0.824 | 1.000 | 2170 | tags=33%, list=14%, signal=39% |
| 1976 | PODAR\_RESPONSE\_TO\_ADAPHOSTIN\_DN |  | 16 | 0.53 | 1.00 | 0.522 | 0.824 | 1.000 | 1550 | tags=31%, list=10%, signal=35% |
| 1977 | LANDIS\_ERBB2\_BREAST\_PRENEOPLASTIC\_UP |  | 17 | 0.52 | 1.00 | 0.509 | 0.823 | 1.000 | 3752 | tags=41%, list=24%, signal=54% |
| 1978 | ACEVEDO\_NORMAL\_TISSUE\_ADJACENT\_TO\_LIVER\_TUMOR\_DN |  | 306 | 0.45 | 1.00 | 0.495 | 0.824 | 1.000 | 3606 | tags=31%, list=23%, signal=39% |
| 1979 | MAGRANGEAS\_MULTIPLE\_MYELOMA\_IGG\_VS\_IGA\_UP |  | 18 | 0.52 | 1.00 | 0.504 | 0.824 | 1.000 | 1696 | tags=22%, list=11%, signal=25% |
| 1980 | REACTOME\_ANTIGEN\_ACTIVATES\_B\_CELL\_RECEPTOR\_LEADING\_TO\_GENERATION\_OF\_SECOND\_MESSENGERS |  | 28 | 0.50 | 1.00 | 0.518 | 0.824 | 1.000 | 566 | tags=11%, list=4%, signal=11% |
| 1981 | ZHU\_CMV\_8\_HR\_DN |  | 50 | 0.47 | 1.00 | 0.506 | 0.825 | 1.000 | 4395 | tags=52%, list=28%, signal=72% |
| 1982 | MULLIGHAN\_NPM1\_MUTATED\_SIGNATURE\_1\_DN |  | 111 | 0.46 | 1.00 | 0.515 | 0.824 | 1.000 | 2357 | tags=23%, list=15%, signal=27% |
| 1983 | PID\_CERAMIDE\_PATHWAY |  | 48 | 0.47 | 1.00 | 0.515 | 0.826 | 1.000 | 4508 | tags=44%, list=29%, signal=61% |
| 1984 | KEGG\_GLYOXYLATE\_AND\_DICARBOXYLATE\_METABOLISM |  | 16 | 0.53 | 1.00 | 0.526 | 0.826 | 1.000 | 815 | tags=19%, list=5%, signal=20% |
| 1985 | BORLAK\_LIVER\_CANCER\_EGF\_UP |  | 55 | 0.47 | 1.00 | 0.509 | 0.826 | 1.000 | 2455 | tags=29%, list=16%, signal=34% |
| 1986 | KIM\_ALL\_DISORDERS\_DURATION\_CORR\_DN |  | 130 | 0.45 | 1.00 | 0.514 | 0.826 | 1.000 | 4889 | tags=42%, list=31%, signal=60% |
| 1987 | BIOCARTA\_BAD\_PATHWAY |  | 25 | 0.50 | 1.00 | 0.513 | 0.826 | 1.000 | 5018 | tags=52%, list=32%, signal=76% |
| 1988 | SUBTIL\_PROGESTIN\_TARGETS |  | 34 | 0.48 | 1.00 | 0.516 | 0.827 | 1.000 | 3983 | tags=41%, list=25%, signal=55% |
| 1989 | BROWNE\_HCMV\_INFECTION\_6HR\_UP |  | 59 | 0.47 | 1.00 | 0.527 | 0.827 | 1.000 | 4360 | tags=37%, list=28%, signal=51% |
| 1990 | GRABARCZYK\_BCL11B\_TARGETS\_DN |  | 49 | 0.47 | 1.00 | 0.516 | 0.828 | 1.000 | 2531 | tags=22%, list=16%, signal=27% |
| 1991 | PID\_IL6\_7PATHWAY |  | 47 | 0.47 | 1.00 | 0.521 | 0.828 | 1.000 | 5380 | tags=49%, list=34%, signal=74% |
| 1992 | MARSON\_FOXP3\_TARGETS\_UP |  | 60 | 0.47 | 1.00 | 0.497 | 0.829 | 1.000 | 3756 | tags=40%, list=24%, signal=52% |
| 1993 | VANTVEER\_BREAST\_CANCER\_BRCA1\_DN |  | 34 | 0.48 | 1.00 | 0.521 | 0.828 | 1.000 | 3790 | tags=38%, list=24%, signal=50% |
| 1994 | PID\_RETINOIC\_ACID\_PATHWAY |  | 27 | 0.50 | 1.00 | 0.527 | 0.829 | 1.000 | 4395 | tags=48%, list=28%, signal=67% |
| 1995 | REACTOME\_TOLL\_RECEPTOR\_CASCADES |  | 105 | 0.46 | 1.00 | 0.515 | 0.829 | 1.000 | 4834 | tags=40%, list=31%, signal=57% |
| 1996 | PID\_INTEGRIN\_CS\_PATHWAY |  | 26 | 0.50 | 1.00 | 0.526 | 0.830 | 1.000 | 1334 | tags=12%, list=8%, signal=13% |
| 1997 | DAUER\_STAT3\_TARGETS\_DN |  | 40 | 0.47 | 1.00 | 0.520 | 0.830 | 1.000 | 5290 | tags=50%, list=34%, signal=75% |
| 1998 | SILIGAN\_BOUND\_BY\_EWS\_FLT1\_FUSION |  | 41 | 0.48 | 1.00 | 0.530 | 0.830 | 1.000 | 4698 | tags=32%, list=30%, signal=45% |
| 1999 | BONOME\_OVARIAN\_CANCER\_SURVIVAL\_OPTIMAL\_DEBULKING |  | 203 | 0.45 | 1.00 | 0.514 | 0.829 | 1.000 | 3268 | tags=22%, list=21%, signal=28% |
| 2000 | POS\_HISTAMINE\_RESPONSE\_NETWORK |  | 30 | 0.49 | 1.00 | 0.516 | 0.829 | 1.000 | 1781 | tags=23%, list=11%, signal=26% |
| 2001 | KESHELAVA\_MULTIPLE\_DRUG\_RESISTANCE |  | 68 | 0.46 | 1.00 | 0.534 | 0.830 | 1.000 | 2875 | tags=32%, list=18%, signal=39% |
| 2002 | BROWNE\_HCMV\_INFECTION\_4HR\_DN |  | 223 | 0.45 | 1.00 | 0.524 | 0.830 | 1.000 | 4223 | tags=35%, list=27%, signal=47% |
| 2003 | SASSON\_RESPONSE\_TO\_FORSKOLIN\_UP |  | 82 | 0.46 | 1.00 | 0.530 | 0.831 | 1.000 | 3680 | tags=32%, list=23%, signal=41% |
| 2004 | REACTOME\_ACTIVATION\_OF\_KAINATE\_RECEPTORS\_UPON\_GLUTAMATE\_BINDING |  | 31 | 0.49 | 1.00 | 0.521 | 0.832 | 1.000 | 4271 | tags=26%, list=27%, signal=35% |
| 2005 | RICKMAN\_TUMOR\_DIFFERENTIATED\_WELL\_VS\_MODERATELY\_DN |  | 99 | 0.46 | 1.00 | 0.527 | 0.832 | 1.000 | 3185 | tags=24%, list=20%, signal=30% |
| 2006 | SUZUKI\_AMPLIFIED\_IN\_ORAL\_CANCER |  | 16 | 0.53 | 1.00 | 0.536 | 0.833 | 1.000 | 2461 | tags=19%, list=16%, signal=22% |
| 2007 | REACTOME\_HEMOSTASIS |  | 419 | 0.44 | 1.00 | 0.515 | 0.834 | 1.000 | 3539 | tags=25%, list=22%, signal=31% |
| 2008 | NUMATA\_CSF3\_SIGNALING\_VIA\_STAT3 |  | 21 | 0.50 | 1.00 | 0.529 | 0.834 | 1.000 | 4429 | tags=48%, list=28%, signal=66% |
| 2009 | MELLMAN\_TUT1\_TARGETS\_DN |  | 46 | 0.47 | 1.00 | 0.518 | 0.834 | 1.000 | 3492 | tags=35%, list=22%, signal=45% |
| 2010 | FIGUEROA\_AML\_METHYLATION\_CLUSTER\_1\_DN |  | 36 | 0.48 | 1.00 | 0.534 | 0.836 | 1.000 | 1266 | tags=14%, list=8%, signal=15% |
| 2011 | KEGG\_AXON\_GUIDANCE |  | 127 | 0.46 | 1.00 | 0.531 | 0.837 | 1.000 | 2800 | tags=20%, list=18%, signal=25% |
| 2012 | HADDAD\_B\_LYMPHOCYTE\_PROGENITOR |  | 231 | 0.45 | 1.00 | 0.539 | 0.837 | 1.000 | 4104 | tags=28%, list=26%, signal=37% |
| 2013 | GHANDHI\_BYSTANDER\_IRRADIATION\_UP |  | 68 | 0.46 | 1.00 | 0.523 | 0.837 | 1.000 | 2109 | tags=15%, list=13%, signal=17% |
| 2014 | XU\_GH1\_AUTOCRINE\_TARGETS\_UP |  | 199 | 0.45 | 1.00 | 0.524 | 0.837 | 1.000 | 3277 | tags=26%, list=21%, signal=32% |
| 2015 | GRAESSMANN\_APOPTOSIS\_BY\_SERUM\_DEPRIVATION\_UP |  | 476 | 0.44 | 1.00 | 0.520 | 0.837 | 1.000 | 4024 | tags=32%, list=26%, signal=41% |
| 2016 | PETRETTO\_CARDIAC\_HYPERTROPHY |  | 33 | 0.48 | 1.00 | 0.533 | 0.838 | 1.000 | 2577 | tags=36%, list=16%, signal=43% |
| 2017 | RHEIN\_ALL\_GLUCOCORTICOID\_THERAPY\_UP |  | 61 | 0.46 | 1.00 | 0.541 | 0.841 | 1.000 | 3892 | tags=30%, list=25%, signal=39% |
| 2018 | FARMER\_BREAST\_CANCER\_BASAL\_VS\_LULMINAL |  | 292 | 0.44 | 1.00 | 0.547 | 0.843 | 1.000 | 4209 | tags=35%, list=27%, signal=47% |
| 2019 | AMIT\_SERUM\_RESPONSE\_20\_MCF10A |  | 20 | 0.51 | 1.00 | 0.531 | 0.842 | 1.000 | 4269 | tags=50%, list=27%, signal=69% |
| 2020 | CHICAS\_RB1\_TARGETS\_CONFLUENT |  | 488 | 0.44 | 1.00 | 0.547 | 0.842 | 1.000 | 3416 | tags=30%, list=22%, signal=37% |
| 2021 | KEGG\_REGULATION\_OF\_ACTIN\_CYTOSKELETON |  | 199 | 0.45 | 0.99 | 0.559 | 0.845 | 1.000 | 3394 | tags=23%, list=22%, signal=29% |
| 2022 | FULCHER\_INFLAMMATORY\_RESPONSE\_LECTIN\_VS\_LPS\_DN |  | 359 | 0.44 | 0.99 | 0.533 | 0.846 | 1.000 | 3767 | tags=28%, list=24%, signal=36% |
| 2023 | REACTOME\_SIGNALING\_BY\_FGFR1\_FUSION\_MUTANTS |  | 16 | 0.52 | 0.99 | 0.516 | 0.845 | 1.000 | 5230 | tags=69%, list=33%, signal=103% |
| 2024 | FULCHER\_INFLAMMATORY\_RESPONSE\_LECTIN\_VS\_LPS\_UP |  | 485 | 0.44 | 0.99 | 0.557 | 0.847 | 1.000 | 3644 | tags=29%, list=23%, signal=36% |
| 2025 | KEGG\_ERBB\_SIGNALING\_PATHWAY |  | 84 | 0.46 | 0.99 | 0.535 | 0.847 | 1.000 | 5031 | tags=48%, list=32%, signal=70% |
| 2026 | RODWELL\_AGING\_KIDNEY\_DN |  | 118 | 0.45 | 0.99 | 0.556 | 0.848 | 1.000 | 3179 | tags=27%, list=20%, signal=34% |
| 2027 | REACTOME\_SIGNALLING\_TO\_ERKS |  | 34 | 0.48 | 0.99 | 0.541 | 0.848 | 1.000 | 5230 | tags=65%, list=33%, signal=97% |
| 2028 | DOUGLAS\_BMI1\_TARGETS\_UP |  | 487 | 0.44 | 0.99 | 0.568 | 0.849 | 1.000 | 3927 | tags=34%, list=25%, signal=44% |
| 2029 | ZHAN\_MULTIPLE\_MYELOMA\_UP |  | 60 | 0.46 | 0.99 | 0.536 | 0.850 | 1.000 | 3339 | tags=38%, list=21%, signal=48% |
| 2030 | GALLUZZI\_PREVENT\_MITOCHONDIAL\_PERMEABILIZATION |  | 22 | 0.50 | 0.99 | 0.547 | 0.850 | 1.000 | 5598 | tags=59%, list=36%, signal=92% |
| 2031 | CAMPS\_COLON\_CANCER\_COPY\_NUMBER\_UP |  | 76 | 0.46 | 0.99 | 0.556 | 0.850 | 1.000 | 4495 | tags=26%, list=29%, signal=37% |
| 2032 | NIKOLSKY\_BREAST\_CANCER\_20Q12\_Q13\_AMPLICON |  | 112 | 0.45 | 0.99 | 0.554 | 0.850 | 1.000 | 3270 | tags=24%, list=21%, signal=30% |
| 2033 | REACTOME\_SIGNALING\_BY\_FGFR\_MUTANTS |  | 41 | 0.47 | 0.99 | 0.536 | 0.852 | 1.000 | 3733 | tags=32%, list=24%, signal=41% |
| 2034 | BOCHKIS\_FOXA2\_TARGETS |  | 384 | 0.44 | 0.99 | 0.564 | 0.852 | 1.000 | 4098 | tags=32%, list=26%, signal=43% |
| 2035 | SANA\_TNF\_SIGNALING\_UP |  | 69 | 0.46 | 0.99 | 0.549 | 0.852 | 1.000 | 3005 | tags=23%, list=19%, signal=29% |
| 2036 | FONTAINE\_FOLLICULAR\_THYROID\_ADENOMA\_DN |  | 62 | 0.46 | 0.99 | 0.555 | 0.852 | 1.000 | 3346 | tags=27%, list=21%, signal=35% |
| 2037 | ST\_INTEGRIN\_SIGNALING\_PATHWAY |  | 80 | 0.46 | 0.99 | 0.552 | 0.852 | 1.000 | 4360 | tags=43%, list=28%, signal=58% |
| 2038 | BRUECKNER\_TARGETS\_OF\_MIRLET7A3\_UP |  | 99 | 0.46 | 0.99 | 0.558 | 0.853 | 1.000 | 3795 | tags=35%, list=24%, signal=46% |
| 2039 | KEGG\_VEGF\_SIGNALING\_PATHWAY |  | 71 | 0.46 | 0.99 | 0.558 | 0.853 | 1.000 | 2737 | tags=25%, list=17%, signal=31% |
| 2040 | HAN\_SATB1\_TARGETS\_DN |  | 377 | 0.44 | 0.99 | 0.568 | 0.853 | 1.000 | 3108 | tags=26%, list=20%, signal=32% |
| 2041 | BROWNE\_HCMV\_INFECTION\_14HR\_DN |  | 266 | 0.44 | 0.99 | 0.560 | 0.852 | 1.000 | 4438 | tags=38%, list=28%, signal=52% |
| 2042 | RUTELLA\_RESPONSE\_TO\_HGF\_VS\_CSF2RB\_AND\_IL4\_DN |  | 217 | 0.45 | 0.99 | 0.562 | 0.852 | 1.000 | 3701 | tags=30%, list=23%, signal=39% |
| 2043 | KLEIN\_PRIMARY\_EFFUSION\_LYMPHOMA\_UP |  | 49 | 0.47 | 0.99 | 0.557 | 0.852 | 1.000 | 2565 | tags=29%, list=16%, signal=34% |
| 2044 | BRUNO\_HEMATOPOIESIS |  | 61 | 0.46 | 0.99 | 0.526 | 0.851 | 1.000 | 4880 | tags=44%, list=31%, signal=64% |
| 2045 | IVANOVA\_HEMATOPOIESIS\_STEM\_CELL |  | 219 | 0.45 | 0.99 | 0.566 | 0.852 | 1.000 | 3408 | tags=26%, list=22%, signal=33% |
| 2046 | REACTOME\_SIGNALING\_BY\_FGFR1\_MUTANTS |  | 27 | 0.48 | 0.99 | 0.543 | 0.856 | 1.000 | 5230 | tags=41%, list=33%, signal=61% |
| 2047 | REACTOME\_SIGNALING\_BY\_SCF\_KIT |  | 71 | 0.46 | 0.99 | 0.555 | 0.857 | 1.000 | 3677 | tags=31%, list=23%, signal=40% |
| 2048 | DARWICHE\_SKIN\_TUMOR\_PROMOTER\_UP |  | 120 | 0.45 | 0.99 | 0.559 | 0.857 | 1.000 | 4757 | tags=46%, list=30%, signal=65% |
| 2049 | DOUGLAS\_BMI1\_TARGETS\_DN |  | 257 | 0.44 | 0.99 | 0.564 | 0.857 | 1.000 | 5340 | tags=47%, list=34%, signal=69% |
| 2050 | PARENT\_MTOR\_SIGNALING\_UP |  | 480 | 0.44 | 0.99 | 0.582 | 0.857 | 1.000 | 3674 | tags=33%, list=23%, signal=41% |
| 2051 | SHETH\_LIVER\_CANCER\_VS\_TXNIP\_LOSS\_PAM2 |  | 140 | 0.45 | 0.99 | 0.558 | 0.857 | 1.000 | 2962 | tags=27%, list=19%, signal=33% |
| 2052 | BIOCARTA\_AT1R\_PATHWAY |  | 31 | 0.48 | 0.99 | 0.546 | 0.859 | 1.000 | 5333 | tags=55%, list=34%, signal=83% |
| 2053 | DELACROIX\_RARG\_BOUND\_MEF |  | 331 | 0.44 | 0.99 | 0.573 | 0.859 | 1.000 | 3184 | tags=27%, list=20%, signal=33% |
| 2054 | KEGG\_B\_CELL\_RECEPTOR\_SIGNALING\_PATHWAY |  | 73 | 0.46 | 0.99 | 0.562 | 0.858 | 1.000 | 4113 | tags=30%, list=26%, signal=41% |
| 2055 | UZONYI\_RESPONSE\_TO\_LEUKOTRIENE\_AND\_THROMBIN |  | 33 | 0.48 | 0.99 | 0.549 | 0.859 | 1.000 | 3469 | tags=27%, list=22%, signal=35% |
| 2056 | PURBEY\_TARGETS\_OF\_CTBP1\_NOT\_SATB1\_UP |  | 287 | 0.44 | 0.99 | 0.591 | 0.858 | 1.000 | 3430 | tags=28%, list=22%, signal=35% |
| 2057 | WIERENGA\_STAT5A\_TARGETS\_GROUP2 |  | 47 | 0.46 | 0.99 | 0.550 | 0.859 | 1.000 | 3491 | tags=28%, list=22%, signal=35% |
| 2058 | SUZUKI\_RESPONSE\_TO\_TSA\_AND\_DECITABINE\_1B |  | 17 | 0.52 | 0.99 | 0.540 | 0.860 | 1.000 | 935 | tags=12%, list=6%, signal=12% |
| 2059 | CHANG\_IMMORTALIZED\_BY\_HPV31\_UP |  | 61 | 0.46 | 0.99 | 0.545 | 0.864 | 1.000 | 3586 | tags=33%, list=23%, signal=42% |
| 2060 | NIKOLSKY\_BREAST\_CANCER\_15Q26\_AMPLICON |  | 18 | 0.51 | 0.98 | 0.556 | 0.866 | 1.000 | 3776 | tags=39%, list=24%, signal=51% |
| 2061 | REACTOME\_AMINO\_ACID\_AND\_OLIGOPEPTIDE\_SLC\_TRANSPORTERS |  | 48 | 0.46 | 0.98 | 0.564 | 0.868 | 1.000 | 3312 | tags=19%, list=21%, signal=24% |
| 2062 | REACTOME\_SIGNALING\_BY\_NOTCH1 |  | 62 | 0.45 | 0.98 | 0.536 | 0.872 | 1.000 | 2172 | tags=21%, list=14%, signal=24% |
| 2063 | MIKKELSEN\_ES\_HCP\_WITH\_H3\_UNMETHYLATED |  | 54 | 0.46 | 0.98 | 0.575 | 0.872 | 1.000 | 336 | tags=4%, list=2%, signal=4% |
| 2064 | SESTO\_RESPONSE\_TO\_UV\_C4 |  | 16 | 0.51 | 0.98 | 0.542 | 0.872 | 1.000 | 3182 | tags=44%, list=20%, signal=55% |
| 2065 | ZHAN\_MULTIPLE\_MYELOMA\_MS\_UP |  | 41 | 0.47 | 0.98 | 0.566 | 0.874 | 1.000 | 300 | tags=7%, list=2%, signal=7% |
| 2066 | GAJATE\_RESPONSE\_TO\_TRABECTEDIN\_DN |  | 18 | 0.51 | 0.98 | 0.559 | 0.875 | 1.000 | 2931 | tags=44%, list=19%, signal=55% |
| 2067 | RIZKI\_TUMOR\_INVASIVENESS\_3D\_DN |  | 240 | 0.44 | 0.98 | 0.611 | 0.875 | 1.000 | 3643 | tags=33%, list=23%, signal=42% |
| 2068 | ALONSO\_METASTASIS\_EMT\_UP |  | 35 | 0.48 | 0.98 | 0.531 | 0.876 | 1.000 | 4229 | tags=43%, list=27%, signal=58% |
| 2069 | REACTOME\_SIGNALING\_BY\_PDGF |  | 112 | 0.44 | 0.98 | 0.564 | 0.879 | 1.000 | 4185 | tags=33%, list=27%, signal=45% |
| 2070 | QI\_HYPOXIA\_TARGETS\_OF\_HIF1A\_AND\_FOXA2 |  | 32 | 0.47 | 0.98 | 0.565 | 0.881 | 1.000 | 4681 | tags=50%, list=30%, signal=71% |
| 2071 | MEINHOLD\_OVARIAN\_CANCER\_LOW\_GRADE\_UP |  | 17 | 0.51 | 0.98 | 0.557 | 0.881 | 1.000 | 3152 | tags=35%, list=20%, signal=44% |
| 2072 | REACTOME\_FGFR\_LIGAND\_BINDING\_AND\_ACTIVATION |  | 22 | 0.50 | 0.98 | 0.560 | 0.881 | 1.000 | 129 | tags=5%, list=1%, signal=5% |
| 2073 | PID\_IL12\_2PATHWAY |  | 57 | 0.45 | 0.98 | 0.574 | 0.881 | 1.000 | 3286 | tags=21%, list=21%, signal=27% |
| 2074 | REACTOME\_ASPARAGINE\_N\_LINKED\_GLYCOSYLATION |  | 75 | 0.45 | 0.98 | 0.562 | 0.884 | 1.000 | 4615 | tags=44%, list=29%, signal=62% |
| 2075 | SHETH\_LIVER\_CANCER\_VS\_TXNIP\_LOSS\_PAM1 |  | 208 | 0.44 | 0.98 | 0.623 | 0.885 | 1.000 | 2245 | tags=20%, list=14%, signal=23% |
| 2076 | RODWELL\_AGING\_KIDNEY\_NO\_BLOOD\_UP |  | 185 | 0.44 | 0.98 | 0.605 | 0.885 | 1.000 | 3605 | tags=34%, list=23%, signal=44% |
| 2077 | PID\_P38ALPHABETAPATHWAY |  | 30 | 0.48 | 0.98 | 0.557 | 0.885 | 1.000 | 3295 | tags=33%, list=21%, signal=42% |
| 2078 | MARSON\_FOXP3\_CORE\_DIRECT\_TARGETS |  | 17 | 0.52 | 0.98 | 0.585 | 0.884 | 1.000 | 5061 | tags=41%, list=32%, signal=61% |
| 2079 | REACTOME\_TIE2\_SIGNALING |  | 17 | 0.51 | 0.98 | 0.562 | 0.885 | 1.000 | 5230 | tags=53%, list=33%, signal=79% |
| 2080 | CHANG\_IMMORTALIZED\_BY\_HPV31\_DN |  | 57 | 0.46 | 0.98 | 0.582 | 0.886 | 1.000 | 3036 | tags=23%, list=19%, signal=28% |
| 2081 | SCHEIDEREIT\_IKK\_INTERACTING\_PROTEINS |  | 55 | 0.46 | 0.98 | 0.582 | 0.886 | 1.000 | 3661 | tags=40%, list=23%, signal=52% |
| 2082 | REACTOME\_PI\_METABOLISM |  | 37 | 0.47 | 0.98 | 0.566 | 0.887 | 1.000 | 5906 | tags=59%, list=37%, signal=95% |
| 2083 | MULLIGHAN\_NPM1\_SIGNATURE\_3\_DN |  | 145 | 0.44 | 0.98 | 0.595 | 0.887 | 1.000 | 2357 | tags=21%, list=15%, signal=25% |
| 2084 | WIEDERSCHAIN\_TARGETS\_OF\_BMI1\_AND\_PCGF2 |  | 54 | 0.46 | 0.98 | 0.580 | 0.888 | 1.000 | 1110 | tags=13%, list=7%, signal=14% |
| 2085 | DEURIG\_T\_CELL\_PROLYMPHOCYTIC\_LEUKEMIA\_UP |  | 314 | 0.43 | 0.98 | 0.646 | 0.889 | 1.000 | 3606 | tags=29%, list=23%, signal=37% |
| 2086 | BRUINS\_UVC\_RESPONSE\_MIDDLE |  | 82 | 0.45 | 0.97 | 0.591 | 0.893 | 1.000 | 5197 | tags=45%, list=33%, signal=67% |
| 2087 | MORI\_SMALL\_PRE\_BII\_LYMPHOCYTE\_UP |  | 80 | 0.45 | 0.97 | 0.593 | 0.894 | 1.000 | 3455 | tags=29%, list=22%, signal=37% |
| 2088 | NUTT\_GBM\_VS\_AO\_GLIOMA\_UP |  | 43 | 0.46 | 0.97 | 0.574 | 0.896 | 1.000 | 2434 | tags=21%, list=15%, signal=25% |
| 2089 | KYNG\_DNA\_DAMAGE\_BY\_GAMMA\_RADIATION |  | 72 | 0.45 | 0.97 | 0.576 | 0.897 | 1.000 | 3001 | tags=26%, list=19%, signal=32% |
| 2090 | BOYLAN\_MULTIPLE\_MYELOMA\_D\_DN |  | 65 | 0.45 | 0.97 | 0.581 | 0.896 | 1.000 | 4041 | tags=34%, list=26%, signal=45% |
| 2091 | PID\_HIVNEFPATHWAY |  | 35 | 0.47 | 0.97 | 0.583 | 0.896 | 1.000 | 4508 | tags=37%, list=29%, signal=52% |
| 2092 | BONOME\_OVARIAN\_CANCER\_SURVIVAL\_SUBOPTIMAL\_DEBULKING |  | 441 | 0.43 | 0.97 | 0.675 | 0.896 | 1.000 | 3976 | tags=33%, list=25%, signal=43% |
| 2093 | MOOTHA\_GLUCONEOGENESIS |  | 31 | 0.47 | 0.97 | 0.565 | 0.902 | 1.000 | 4058 | tags=45%, list=26%, signal=61% |
| 2094 | SESTO\_RESPONSE\_TO\_UV\_C3 |  | 19 | 0.50 | 0.97 | 0.548 | 0.902 | 1.000 | 3561 | tags=37%, list=23%, signal=48% |
| 2095 | PID\_ARF\_3PATHWAY |  | 16 | 0.51 | 0.97 | 0.572 | 0.901 | 1.000 | 5362 | tags=56%, list=34%, signal=85% |
| 2096 | CHEBOTAEV\_GR\_TARGETS\_DN |  | 107 | 0.44 | 0.97 | 0.594 | 0.901 | 1.000 | 3476 | tags=21%, list=22%, signal=26% |
| 2097 | GAJATE\_RESPONSE\_TO\_TRABECTEDIN\_UP |  | 60 | 0.45 | 0.97 | 0.600 | 0.902 | 1.000 | 2452 | tags=30%, list=16%, signal=35% |
| 2098 | HOFMANN\_MYELODYSPLASTIC\_SYNDROM\_HIGH\_RISK\_DN |  | 15 | 0.51 | 0.97 | 0.560 | 0.901 | 1.000 | 467 | tags=7%, list=3%, signal=7% |
| 2099 | ALONSO\_METASTASIS\_NEURAL\_UP |  | 17 | 0.51 | 0.97 | 0.561 | 0.901 | 1.000 | 2216 | tags=29%, list=14%, signal=34% |
| 2100 | PID\_HES\_HEYPATHWAY |  | 45 | 0.46 | 0.97 | 0.590 | 0.901 | 1.000 | 3406 | tags=31%, list=22%, signal=40% |
| 2101 | SWEET\_LUNG\_CANCER\_KRAS\_UP |  | 439 | 0.43 | 0.97 | 0.682 | 0.901 | 1.000 | 3332 | tags=31%, list=21%, signal=38% |
| 2102 | KYNG\_NORMAL\_AGING\_DN |  | 23 | 0.49 | 0.97 | 0.563 | 0.901 | 1.000 | 3465 | tags=26%, list=22%, signal=33% |
| 2103 | REACTOME\_FATTY\_ACID\_TRIACYLGLYCEROL\_AND\_KETONE\_BODY\_METABOLISM |  | 141 | 0.44 | 0.97 | 0.622 | 0.901 | 1.000 | 3311 | tags=32%, list=21%, signal=40% |
| 2104 | ZHANG\_RESPONSE\_TO\_CANTHARIDIN\_UP |  | 17 | 0.51 | 0.97 | 0.573 | 0.902 | 1.000 | 3491 | tags=29%, list=22%, signal=38% |
| 2105 | TAVOR\_CEBPA\_TARGETS\_UP |  | 45 | 0.46 | 0.97 | 0.582 | 0.901 | 1.000 | 3025 | tags=27%, list=19%, signal=33% |
| 2106 | MAHADEVAN\_RESPONSE\_TO\_MP470\_DN |  | 17 | 0.51 | 0.97 | 0.569 | 0.903 | 1.000 | 1809 | tags=24%, list=11%, signal=27% |
| 2107 | BROWNE\_HCMV\_INFECTION\_24HR\_DN |  | 133 | 0.44 | 0.97 | 0.631 | 0.903 | 1.000 | 3159 | tags=29%, list=20%, signal=35% |
| 2108 | XU\_HGF\_SIGNALING\_NOT\_VIA\_AKT1\_6HR |  | 23 | 0.48 | 0.97 | 0.574 | 0.905 | 1.000 | 1801 | tags=26%, list=11%, signal=29% |
| 2109 | FARMER\_BREAST\_CANCER\_APOCRINE\_VS\_LUMINAL |  | 282 | 0.43 | 0.97 | 0.670 | 0.905 | 1.000 | 4070 | tags=32%, list=26%, signal=42% |
| 2110 | REACTOME\_MITOCHONDRIAL\_TRNA\_AMINOACYLATION |  | 21 | 0.49 | 0.97 | 0.587 | 0.906 | 1.000 | 4707 | tags=38%, list=30%, signal=54% |
| 2111 | WINZEN\_DEGRADED\_VIA\_KHSRP |  | 95 | 0.45 | 0.97 | 0.609 | 0.906 | 1.000 | 3304 | tags=21%, list=21%, signal=26% |
| 2112 | PID\_THROMBIN\_PAR1\_PATHWAY |  | 41 | 0.46 | 0.97 | 0.575 | 0.907 | 1.000 | 3484 | tags=32%, list=22%, signal=41% |
| 2113 | MAHADEVAN\_GIST\_MORPHOLOGICAL\_SWITCH |  | 15 | 0.51 | 0.97 | 0.579 | 0.908 | 1.000 | 1330 | tags=13%, list=8%, signal=15% |
| 2114 | PID\_PTP1BPATHWAY |  | 52 | 0.46 | 0.97 | 0.583 | 0.909 | 1.000 | 4197 | tags=27%, list=27%, signal=37% |
| 2115 | JOSEPH\_RESPONSE\_TO\_SODIUM\_BUTYRATE\_UP |  | 28 | 0.47 | 0.97 | 0.577 | 0.909 | 1.000 | 3266 | tags=39%, list=21%, signal=49% |
| 2116 | KAYO\_AGING\_MUSCLE\_UP |  | 213 | 0.43 | 0.97 | 0.640 | 0.909 | 1.000 | 3589 | tags=23%, list=23%, signal=29% |
| 2117 | BIOCARTA\_ETS\_PATHWAY |  | 18 | 0.50 | 0.97 | 0.589 | 0.911 | 1.000 | 3673 | tags=50%, list=23%, signal=65% |
| 2118 | HAHTOLA\_MYCOSIS\_FUNGOIDES\_CD4\_UP |  | 58 | 0.45 | 0.97 | 0.601 | 0.913 | 1.000 | 2165 | tags=21%, list=14%, signal=24% |
| 2119 | CHIANG\_LIVER\_CANCER\_SUBCLASS\_INTERFERON\_DN |  | 45 | 0.46 | 0.96 | 0.591 | 0.914 | 1.000 | 2885 | tags=18%, list=18%, signal=22% |
| 2120 | REACTOME\_SIGNALING\_BY\_ILS |  | 100 | 0.44 | 0.96 | 0.622 | 0.915 | 1.000 | 4031 | tags=31%, list=26%, signal=41% |
| 2121 | BIOCARTA\_PTEN\_PATHWAY |  | 18 | 0.51 | 0.96 | 0.583 | 0.916 | 1.000 | 4098 | tags=56%, list=26%, signal=75% |
| 2122 | BILANGES\_SERUM\_SENSITIVE\_VIA\_TSC1 |  | 22 | 0.49 | 0.96 | 0.592 | 0.917 | 1.000 | 3209 | tags=36%, list=20%, signal=46% |
| 2123 | WANG\_HCP\_PROSTATE\_CANCER |  | 99 | 0.44 | 0.96 | 0.613 | 0.917 | 1.000 | 3481 | tags=28%, list=22%, signal=36% |
| 2124 | RUTELLA\_RESPONSE\_TO\_HGF\_VS\_CSF2RB\_AND\_IL4\_UP |  | 343 | 0.43 | 0.96 | 0.702 | 0.918 | 1.000 | 4236 | tags=32%, list=27%, signal=43% |
| 2125 | FARMER\_BREAST\_CANCER\_CLUSTER\_5 |  | 19 | 0.50 | 0.96 | 0.576 | 0.918 | 1.000 | 2990 | tags=26%, list=19%, signal=32% |
| 2126 | DACOSTA\_UV\_RESPONSE\_VIA\_ERCC3\_XPCS\_UP |  | 22 | 0.49 | 0.96 | 0.583 | 0.918 | 1.000 | 3475 | tags=41%, list=22%, signal=52% |
| 2127 | PETRETTO\_HEART\_MASS\_QTL\_CIS\_DN |  | 21 | 0.49 | 0.96 | 0.614 | 0.917 | 1.000 | 3563 | tags=43%, list=23%, signal=55% |
| 2128 | LANDIS\_ERBB2\_BREAST\_TUMORS\_324\_UP |  | 134 | 0.44 | 0.96 | 0.646 | 0.918 | 1.000 | 2009 | tags=22%, list=13%, signal=25% |
| 2129 | VERHAAK\_GLIOBLASTOMA\_PRONEURAL |  | 180 | 0.43 | 0.96 | 0.668 | 0.924 | 1.000 | 4208 | tags=26%, list=27%, signal=35% |
| 2130 | PID\_ERBB\_NETWORK\_PATHWAY |  | 15 | 0.51 | 0.96 | 0.585 | 0.925 | 1.000 | 1699 | tags=20%, list=11%, signal=22% |
| 2131 | REACTOME\_NEF\_MEDIATES\_DOWN\_MODULATION\_OF\_CELL\_SURFACE\_RECEPTORS\_BY\_RECRUITING\_THEM\_TO\_CLATHRIN\_ADAPTERS |  | 19 | 0.50 | 0.96 | 0.593 | 0.926 | 1.000 | 1944 | tags=32%, list=12%, signal=36% |
| 2132 | ENK\_UV\_RESPONSE\_EPIDERMIS\_DN |  | 474 | 0.42 | 0.96 | 0.753 | 0.927 | 1.000 | 3628 | tags=32%, list=23%, signal=40% |
| 2133 | BILBAN\_B\_CLL\_LPL\_UP |  | 58 | 0.45 | 0.96 | 0.615 | 0.927 | 1.000 | 4632 | tags=33%, list=29%, signal=46% |
| 2134 | GERY\_CEBP\_TARGETS |  | 116 | 0.43 | 0.96 | 0.643 | 0.927 | 1.000 | 3147 | tags=29%, list=20%, signal=36% |
| 2135 | HOSHIDA\_LIVER\_CANCER\_LATE\_RECURRENCE\_UP |  | 53 | 0.45 | 0.96 | 0.617 | 0.927 | 1.000 | 2125 | tags=23%, list=13%, signal=26% |
| 2136 | NIKOLSKY\_BREAST\_CANCER\_5P15\_AMPLICON |  | 22 | 0.48 | 0.96 | 0.586 | 0.928 | 1.000 | 3854 | tags=36%, list=24%, signal=48% |
| 2137 | KYNG\_ENVIRONMENTAL\_STRESS\_RESPONSE\_NOT\_BY\_4NQO\_IN\_WS |  | 38 | 0.46 | 0.96 | 0.610 | 0.929 | 1.000 | 3218 | tags=34%, list=20%, signal=43% |
| 2138 | VERRECCHIA\_RESPONSE\_TO\_TGFB1\_C1 |  | 19 | 0.50 | 0.96 | 0.596 | 0.929 | 1.000 | 540 | tags=16%, list=3%, signal=16% |
| 2139 | LEIN\_PONS\_MARKERS |  | 82 | 0.44 | 0.96 | 0.625 | 0.930 | 1.000 | 1806 | tags=13%, list=11%, signal=15% |
| 2140 | MOOTHA\_TCA |  | 16 | 0.50 | 0.96 | 0.579 | 0.931 | 1.000 | 4799 | tags=56%, list=30%, signal=81% |
| 2141 | BROWN\_MYELOID\_CELL\_DEVELOPMENT\_UP |  | 148 | 0.43 | 0.96 | 0.664 | 0.933 | 1.000 | 3310 | tags=23%, list=21%, signal=29% |
| 2142 | DAVICIONI\_TARGETS\_OF\_PAX\_FOXO1\_FUSIONS\_DN |  | 61 | 0.44 | 0.96 | 0.628 | 0.934 | 1.000 | 2476 | tags=13%, list=16%, signal=16% |
| 2143 | BROWNE\_HCMV\_INFECTION\_20HR\_UP |  | 207 | 0.43 | 0.96 | 0.692 | 0.935 | 1.000 | 3960 | tags=32%, list=25%, signal=42% |
| 2144 | PID\_CD40\_PATHWAY |  | 29 | 0.47 | 0.96 | 0.603 | 0.935 | 1.000 | 4725 | tags=45%, list=30%, signal=64% |
| 2145 | KEGG\_FOCAL\_ADHESION |  | 188 | 0.43 | 0.96 | 0.678 | 0.935 | 1.000 | 4113 | tags=32%, list=26%, signal=43% |
| 2146 | NAKAMURA\_ADIPOGENESIS\_EARLY\_UP |  | 54 | 0.45 | 0.95 | 0.624 | 0.935 | 1.000 | 3971 | tags=35%, list=25%, signal=47% |
| 2147 | BIOCARTA\_PML\_PATHWAY |  | 17 | 0.50 | 0.95 | 0.616 | 0.935 | 1.000 | 3146 | tags=29%, list=20%, signal=37% |
| 2148 | KEGG\_BIOSYNTHESIS\_OF\_UNSATURATED\_FATTY\_ACIDS |  | 20 | 0.50 | 0.95 | 0.616 | 0.935 | 1.000 | 5009 | tags=55%, list=32%, signal=81% |
| 2149 | BOYAULT\_LIVER\_CANCER\_SUBCLASS\_G2 |  | 24 | 0.48 | 0.95 | 0.600 | 0.935 | 1.000 | 4850 | tags=46%, list=31%, signal=66% |
| 2150 | MORI\_PRE\_BI\_LYMPHOCYTE\_DN |  | 70 | 0.44 | 0.95 | 0.642 | 0.936 | 1.000 | 3307 | tags=24%, list=21%, signal=31% |
| 2151 | BIOCARTA\_PAR1\_PATHWAY |  | 36 | 0.46 | 0.95 | 0.612 | 0.936 | 1.000 | 4482 | tags=42%, list=28%, signal=58% |
| 2152 | PID\_INTEGRIN1\_PATHWAY |  | 66 | 0.44 | 0.95 | 0.632 | 0.936 | 1.000 | 2977 | tags=23%, list=19%, signal=28% |
| 2153 | ZHAN\_LATE\_DIFFERENTIATION\_GENES\_DN |  | 15 | 0.51 | 0.95 | 0.589 | 0.935 | 1.000 | 2663 | tags=33%, list=17%, signal=40% |
| 2154 | XU\_GH1\_EXOGENOUS\_TARGETS\_DN |  | 99 | 0.44 | 0.95 | 0.640 | 0.935 | 1.000 | 4328 | tags=26%, list=27%, signal=36% |
| 2155 | KEGG\_VIRAL\_MYOCARDITIS |  | 60 | 0.44 | 0.95 | 0.612 | 0.934 | 1.000 | 1728 | tags=15%, list=11%, signal=17% |
| 2156 | MIZUSHIMA\_AUTOPHAGOSOME\_FORMATION |  | 15 | 0.51 | 0.95 | 0.602 | 0.935 | 1.000 | 3816 | tags=40%, list=24%, signal=53% |
| 2157 | PID\_RET\_PATHWAY |  | 39 | 0.45 | 0.95 | 0.618 | 0.935 | 1.000 | 4466 | tags=49%, list=28%, signal=68% |
| 2158 | WANG\_LSD1\_TARGETS\_UP |  | 22 | 0.48 | 0.95 | 0.603 | 0.935 | 1.000 | 1031 | tags=23%, list=7%, signal=24% |
| 2159 | BOSCO\_ALLERGEN\_INDUCED\_TH2\_ASSOCIATED\_MODULE |  | 134 | 0.43 | 0.95 | 0.656 | 0.935 | 1.000 | 3574 | tags=24%, list=23%, signal=31% |
| 2160 | BIOCARTA\_CCR5\_PATHWAY |  | 15 | 0.50 | 0.95 | 0.606 | 0.937 | 1.000 | 4482 | tags=47%, list=28%, signal=65% |
| 2161 | KEGG\_ACUTE\_MYELOID\_LEUKEMIA |  | 56 | 0.45 | 0.95 | 0.623 | 0.937 | 1.000 | 3328 | tags=29%, list=21%, signal=36% |
| 2162 | MARCHINI\_TRABECTEDIN\_RESISTANCE\_UP |  | 19 | 0.49 | 0.95 | 0.590 | 0.940 | 1.000 | 2868 | tags=32%, list=18%, signal=39% |
| 2163 | JUBAN\_TARGETS\_OF\_SPI1\_AND\_FLI1\_UP |  | 103 | 0.43 | 0.95 | 0.661 | 0.940 | 1.000 | 3603 | tags=34%, list=23%, signal=44% |
| 2164 | GROSS\_HIF1A\_TARGETS\_DN |  | 24 | 0.47 | 0.95 | 0.617 | 0.941 | 1.000 | 4206 | tags=50%, list=27%, signal=68% |
| 2165 | STEIN\_ESRRA\_TARGETS |  | 454 | 0.42 | 0.95 | 0.787 | 0.941 | 1.000 | 4154 | tags=37%, list=26%, signal=49% |
| 2166 | GAZDA\_DIAMOND\_BLACKFAN\_ANEMIA\_MYELOID\_UP |  | 26 | 0.47 | 0.95 | 0.600 | 0.943 | 1.000 | 4282 | tags=50%, list=27%, signal=69% |
| 2167 | REACTOME\_TRANSMISSION\_ACROSS\_CHEMICAL\_SYNAPSES |  | 180 | 0.43 | 0.95 | 0.701 | 0.944 | 1.000 | 3484 | tags=15%, list=22%, signal=19% |
| 2168 | PID\_ALK1PATHWAY |  | 26 | 0.47 | 0.95 | 0.613 | 0.944 | 1.000 | 3642 | tags=38%, list=23%, signal=50% |
| 2169 | INGRAM\_SHH\_TARGETS\_DN |  | 56 | 0.44 | 0.95 | 0.632 | 0.944 | 1.000 | 1792 | tags=20%, list=11%, signal=22% |
| 2170 | UDAYAKUMAR\_MED1\_TARGETS\_DN |  | 208 | 0.43 | 0.95 | 0.712 | 0.946 | 1.000 | 4430 | tags=39%, list=28%, signal=53% |
| 2171 | NEWMAN\_ERCC6\_TARGETS\_UP |  | 18 | 0.49 | 0.95 | 0.620 | 0.946 | 1.000 | 3301 | tags=33%, list=21%, signal=42% |
| 2172 | KEGG\_MAPK\_SIGNALING\_PATHWAY |  | 253 | 0.42 | 0.95 | 0.731 | 0.947 | 1.000 | 4879 | tags=35%, list=31%, signal=50% |
| 2173 | KEGG\_ENDOCYTOSIS |  | 155 | 0.43 | 0.95 | 0.682 | 0.947 | 1.000 | 3776 | tags=32%, list=24%, signal=42% |
| 2174 | PID\_ERBB2ERBB3PATHWAY |  | 43 | 0.45 | 0.95 | 0.632 | 0.948 | 1.000 | 5333 | tags=58%, list=34%, signal=88% |
| 2175 | REACTOME\_PHOSPHOLIPASE\_C\_MEDIATED\_CASCADE |  | 51 | 0.45 | 0.95 | 0.635 | 0.948 | 1.000 | 566 | tags=8%, list=4%, signal=8% |
| 2176 | PID\_IL3\_PATHWAY |  | 25 | 0.48 | 0.95 | 0.600 | 0.948 | 1.000 | 4025 | tags=40%, list=26%, signal=54% |
| 2177 | ENK\_UV\_RESPONSE\_KERATINOCYTE\_UP |  | 487 | 0.42 | 0.95 | 0.814 | 0.948 | 1.000 | 3672 | tags=32%, list=23%, signal=41% |
| 2178 | MASSARWEH\_TAMOXIFEN\_RESISTANCE\_DN |  | 205 | 0.43 | 0.95 | 0.722 | 0.948 | 1.000 | 4197 | tags=28%, list=27%, signal=37% |
| 2179 | BURTON\_ADIPOGENESIS\_PEAK\_AT\_2HR |  | 50 | 0.44 | 0.95 | 0.645 | 0.949 | 1.000 | 3561 | tags=32%, list=23%, signal=41% |
| 2180 | BIOCARTA\_GLEEVEC\_PATHWAY |  | 23 | 0.48 | 0.95 | 0.615 | 0.951 | 1.000 | 5230 | tags=57%, list=33%, signal=84% |
| 2181 | THEODOROU\_MAMMARY\_TUMORIGENESIS |  | 30 | 0.46 | 0.95 | 0.604 | 0.951 | 1.000 | 590 | tags=7%, list=4%, signal=7% |
| 2182 | ZHAN\_MULTIPLE\_MYELOMA\_LB\_DN |  | 34 | 0.46 | 0.94 | 0.623 | 0.953 | 1.000 | 4540 | tags=35%, list=29%, signal=49% |
| 2183 | CEBALLOS\_TARGETS\_OF\_TP53\_AND\_MYC\_DN |  | 34 | 0.46 | 0.94 | 0.633 | 0.954 | 1.000 | 2450 | tags=26%, list=16%, signal=31% |
| 2184 | DAVICIONI\_RHABDOMYOSARCOMA\_PAX\_FOXO1\_FUSION\_UP |  | 56 | 0.43 | 0.94 | 0.634 | 0.954 | 1.000 | 3725 | tags=23%, list=24%, signal=30% |
| 2185 | DARWICHE\_PAPILLOMA\_RISK\_HIGH\_DN |  | 145 | 0.43 | 0.94 | 0.694 | 0.953 | 1.000 | 2400 | tags=21%, list=15%, signal=25% |
| 2186 | YAO\_TEMPORAL\_RESPONSE\_TO\_PROGESTERONE\_CLUSTER\_15 |  | 32 | 0.46 | 0.94 | 0.624 | 0.955 | 1.000 | 2822 | tags=28%, list=18%, signal=34% |
| 2187 | PAPASPYRIDONOS\_UNSTABLE\_ATEROSCLEROTIC\_PLAQUE\_DN |  | 40 | 0.44 | 0.94 | 0.636 | 0.956 | 1.000 | 2436 | tags=30%, list=15%, signal=35% |
| 2188 | CHARAFE\_BREAST\_CANCER\_BASAL\_VS\_MESENCHYMAL\_DN |  | 43 | 0.45 | 0.94 | 0.637 | 0.956 | 1.000 | 2436 | tags=19%, list=15%, signal=22% |
| 2189 | GAVIN\_IL2\_RESPONSIVE\_FOXP3\_TARGETS\_UP |  | 19 | 0.49 | 0.94 | 0.616 | 0.956 | 1.000 | 4836 | tags=47%, list=31%, signal=68% |
| 2190 | LIEN\_BREAST\_CARCINOMA\_METAPLASTIC |  | 33 | 0.46 | 0.94 | 0.640 | 0.957 | 1.000 | 2133 | tags=27%, list=14%, signal=31% |
| 2191 | ZHAN\_MULTIPLE\_MYELOMA\_CD1\_UP |  | 42 | 0.45 | 0.94 | 0.640 | 0.957 | 1.000 | 1665 | tags=19%, list=11%, signal=21% |
| 2192 | WANG\_NEOPLASTIC\_TRANSFORMATION\_BY\_CCND1\_MYC |  | 20 | 0.49 | 0.94 | 0.616 | 0.958 | 1.000 | 924 | tags=15%, list=6%, signal=16% |
| 2193 | MIKKELSEN\_NPC\_ICP\_WITH\_H3K4ME3 |  | 380 | 0.42 | 0.94 | 0.789 | 0.958 | 1.000 | 4956 | tags=32%, list=31%, signal=45% |
| 2194 | LIANG\_HEMATOPOIESIS\_STEM\_CELL\_NUMBER\_SMALL\_VS\_HUGE\_DN |  | 28 | 0.46 | 0.94 | 0.631 | 0.959 | 1.000 | 4360 | tags=46%, list=28%, signal=64% |
| 2195 | STEGER\_ADIPOGENESIS\_DN |  | 25 | 0.47 | 0.94 | 0.618 | 0.959 | 1.000 | 2054 | tags=28%, list=13%, signal=32% |
| 2196 | REACTOME\_OTHER\_SEMAPHORIN\_INTERACTIONS |  | 15 | 0.50 | 0.94 | 0.605 | 0.960 | 1.000 | 2059 | tags=13%, list=13%, signal=15% |
| 2197 | KEGG\_PORPHYRIN\_AND\_CHLOROPHYLL\_METABOLISM |  | 34 | 0.46 | 0.94 | 0.638 | 0.960 | 1.000 | 3637 | tags=24%, list=23%, signal=31% |
| 2198 | ST\_P38\_MAPK\_PATHWAY |  | 35 | 0.45 | 0.94 | 0.625 | 0.960 | 1.000 | 4953 | tags=60%, list=31%, signal=87% |
| 2199 | TURASHVILI\_BREAST\_LOBULAR\_CARCINOMA\_VS\_LOBULAR\_NORMAL\_DN |  | 67 | 0.44 | 0.94 | 0.657 | 0.960 | 1.000 | 2871 | tags=21%, list=18%, signal=25% |
| 2200 | TERAO\_AOX4\_TARGETS\_SKIN\_DN |  | 20 | 0.48 | 0.94 | 0.632 | 0.961 | 1.000 | 95 | tags=5%, list=1%, signal=5% |
| 2201 | KEGG\_BUTANOATE\_METABOLISM |  | 30 | 0.46 | 0.94 | 0.642 | 0.961 | 1.000 | 340 | tags=13%, list=2%, signal=14% |
| 2202 | KHETCHOUMIAN\_TRIM24\_TARGETS\_UP |  | 47 | 0.44 | 0.94 | 0.654 | 0.960 | 1.000 | 2165 | tags=26%, list=14%, signal=30% |
| 2203 | BIOCARTA\_STATHMIN\_PATHWAY |  | 17 | 0.49 | 0.94 | 0.590 | 0.960 | 1.000 | 3256 | tags=29%, list=21%, signal=37% |
| 2204 | DE\_YY1\_TARGETS\_UP |  | 17 | 0.49 | 0.94 | 0.620 | 0.961 | 1.000 | 987 | tags=12%, list=6%, signal=13% |
| 2205 | DANG\_MYC\_TARGETS\_DN |  | 30 | 0.46 | 0.94 | 0.633 | 0.962 | 1.000 | 4747 | tags=47%, list=30%, signal=67% |
| 2206 | ROPERO\_HDAC2\_TARGETS |  | 86 | 0.43 | 0.94 | 0.690 | 0.962 | 1.000 | 2570 | tags=17%, list=16%, signal=21% |
| 2207 | VERRECCHIA\_DELAYED\_RESPONSE\_TO\_TGFB1 |  | 39 | 0.45 | 0.94 | 0.638 | 0.961 | 1.000 | 3952 | tags=44%, list=25%, signal=58% |
| 2208 | SHETH\_LIVER\_CANCER\_VS\_TXNIP\_LOSS\_PAM5 |  | 81 | 0.44 | 0.94 | 0.663 | 0.961 | 1.000 | 4328 | tags=23%, list=27%, signal=32% |
| 2209 | BASSO\_HAIRY\_CELL\_LEUKEMIA\_DN |  | 74 | 0.43 | 0.94 | 0.685 | 0.962 | 1.000 | 3975 | tags=31%, list=25%, signal=41% |
| 2210 | LI\_INDUCED\_T\_TO\_NATURAL\_KILLER\_UP |  | 263 | 0.42 | 0.94 | 0.772 | 0.962 | 1.000 | 2906 | tags=23%, list=18%, signal=28% |
| 2211 | MOREIRA\_RESPONSE\_TO\_TSA\_DN |  | 18 | 0.49 | 0.94 | 0.615 | 0.962 | 1.000 | 448 | tags=11%, list=3%, signal=11% |
| 2212 | SASSON\_RESPONSE\_TO\_FORSKOLIN\_DN |  | 82 | 0.43 | 0.94 | 0.679 | 0.962 | 1.000 | 1792 | tags=26%, list=11%, signal=29% |
| 2213 | REACTOME\_THE\_ROLE\_OF\_NEF\_IN\_HIV1\_REPLICATION\_AND\_DISEASE\_PATHOGENESIS |  | 26 | 0.46 | 0.94 | 0.620 | 0.962 | 1.000 | 2297 | tags=27%, list=15%, signal=31% |
| 2214 | BURTON\_ADIPOGENESIS\_1 |  | 32 | 0.45 | 0.94 | 0.632 | 0.962 | 1.000 | 1201 | tags=19%, list=8%, signal=20% |
| 2215 | DAZARD\_UV\_RESPONSE\_CLUSTER\_G3 |  | 15 | 0.50 | 0.94 | 0.635 | 0.962 | 1.000 | 3475 | tags=27%, list=22%, signal=34% |
| 2216 | DACOSTA\_UV\_RESPONSE\_VIA\_ERCC3\_UP |  | 274 | 0.42 | 0.94 | 0.772 | 0.962 | 1.000 | 3320 | tags=31%, list=21%, signal=39% |
| 2217 | JAZAG\_TGFB1\_SIGNALING\_VIA\_SMAD4\_UP |  | 97 | 0.42 | 0.94 | 0.698 | 0.962 | 1.000 | 2721 | tags=19%, list=17%, signal=22% |
| 2218 | RICKMAN\_TUMOR\_DIFFERENTIATED\_MODERATELY\_VS\_POORLY\_UP |  | 103 | 0.43 | 0.94 | 0.691 | 0.962 | 1.000 | 2256 | tags=18%, list=14%, signal=21% |
| 2219 | REACTOME\_G\_ALPHA1213\_SIGNALLING\_EVENTS |  | 72 | 0.43 | 0.94 | 0.677 | 0.962 | 1.000 | 5348 | tags=38%, list=34%, signal=57% |
| 2220 | FIRESTEIN\_PROLIFERATION |  | 159 | 0.42 | 0.94 | 0.746 | 0.962 | 1.000 | 3197 | tags=22%, list=20%, signal=27% |
| 2221 | REACTOME\_COLLAGEN\_FORMATION |  | 57 | 0.44 | 0.94 | 0.665 | 0.962 | 1.000 | 2708 | tags=18%, list=17%, signal=21% |
| 2222 | NADLER\_HYPERGLYCEMIA\_AT\_OBESITY |  | 54 | 0.43 | 0.94 | 0.657 | 0.962 | 1.000 | 5597 | tags=57%, list=36%, signal=89% |
| 2223 | REACTOME\_REGULATION\_OF\_SIGNALING\_BY\_CBL |  | 18 | 0.49 | 0.94 | 0.625 | 0.962 | 1.000 | 5703 | tags=61%, list=36%, signal=96% |
| 2224 | HIRSCH\_CELLULAR\_TRANSFORMATION\_SIGNATURE\_UP |  | 214 | 0.42 | 0.94 | 0.753 | 0.962 | 1.000 | 4424 | tags=37%, list=28%, signal=51% |
| 2225 | LEE\_NEURAL\_CREST\_STEM\_CELL\_UP |  | 134 | 0.42 | 0.94 | 0.718 | 0.962 | 1.000 | 2313 | tags=15%, list=15%, signal=17% |
| 2226 | LINDGREN\_BLADDER\_CANCER\_CLUSTER\_3\_DN |  | 184 | 0.42 | 0.94 | 0.735 | 0.962 | 1.000 | 4107 | tags=32%, list=26%, signal=42% |
| 2227 | KEGG\_PENTOSE\_PHOSPHATE\_PATHWAY |  | 26 | 0.45 | 0.93 | 0.639 | 0.963 | 1.000 | 1572 | tags=19%, list=10%, signal=21% |
| 2228 | PID\_TCR\_PATHWAY |  | 63 | 0.43 | 0.93 | 0.663 | 0.964 | 1.000 | 4098 | tags=29%, list=26%, signal=38% |
| 2229 | MANTOVANI\_VIRAL\_GPCR\_SIGNALING\_UP |  | 82 | 0.43 | 0.93 | 0.679 | 0.965 | 1.000 | 3362 | tags=23%, list=21%, signal=29% |
| 2230 | BOQUEST\_STEM\_CELL\_CULTURED\_VS\_FRESH\_UP |  | 385 | 0.41 | 0.93 | 0.816 | 0.969 | 1.000 | 3497 | tags=25%, list=22%, signal=31% |
| 2231 | PID\_S1P\_S1P3\_PATHWAY |  | 26 | 0.46 | 0.93 | 0.649 | 0.969 | 1.000 | 4522 | tags=42%, list=29%, signal=59% |
| 2232 | WARTERS\_RESPONSE\_TO\_IR\_SKIN |  | 62 | 0.43 | 0.93 | 0.671 | 0.973 | 1.000 | 2961 | tags=23%, list=19%, signal=28% |
| 2233 | LEIN\_NEURON\_MARKERS |  | 62 | 0.43 | 0.93 | 0.669 | 0.975 | 1.000 | 4133 | tags=24%, list=26%, signal=33% |
| 2234 | GU\_PDEF\_TARGETS\_UP |  | 70 | 0.43 | 0.93 | 0.680 | 0.975 | 1.000 | 3666 | tags=34%, list=23%, signal=44% |
| 2235 | PID\_HIF2PATHWAY |  | 32 | 0.45 | 0.93 | 0.645 | 0.976 | 1.000 | 4930 | tags=53%, list=31%, signal=77% |
| 2236 | PHONG\_TNF\_TARGETS\_UP |  | 58 | 0.43 | 0.93 | 0.672 | 0.978 | 1.000 | 1985 | tags=16%, list=13%, signal=18% |
| 2237 | PLASARI\_NFIC\_TARGETS\_BASAL\_DN |  | 18 | 0.49 | 0.93 | 0.660 | 0.977 | 1.000 | 2712 | tags=28%, list=17%, signal=34% |
| 2238 | CHEN\_HOXA5\_TARGETS\_9HR\_DN |  | 36 | 0.44 | 0.93 | 0.660 | 0.978 | 1.000 | 3062 | tags=36%, list=19%, signal=45% |
| 2239 | HUMMERICH\_MALIGNANT\_SKIN\_TUMOR\_UP |  | 15 | 0.49 | 0.93 | 0.638 | 0.978 | 1.000 | 2779 | tags=27%, list=18%, signal=32% |
| 2240 | PHONG\_TNF\_RESPONSE\_VIA\_P38\_PARTIAL |  | 148 | 0.42 | 0.93 | 0.753 | 0.977 | 1.000 | 3908 | tags=30%, list=25%, signal=40% |
| 2241 | KEGG\_GLYCOSAMINOGLYCAN\_BIOSYNTHESIS\_HEPARAN\_SULFATE |  | 25 | 0.46 | 0.93 | 0.645 | 0.978 | 1.000 | 3512 | tags=24%, list=22%, signal=31% |
| 2242 | WAKABAYASHI\_ADIPOGENESIS\_PPARG\_RXRA\_BOUND\_WITH\_H4K20ME1\_MARK |  | 121 | 0.42 | 0.93 | 0.741 | 0.978 | 1.000 | 4324 | tags=40%, list=27%, signal=54% |
| 2243 | DAVIES\_MULTIPLE\_MYELOMA\_VS\_MGUS\_DN |  | 26 | 0.46 | 0.93 | 0.632 | 0.978 | 1.000 | 3206 | tags=31%, list=20%, signal=39% |
| 2244 | SUNG\_METASTASIS\_STROMA\_UP |  | 96 | 0.43 | 0.93 | 0.704 | 0.978 | 1.000 | 2587 | tags=23%, list=16%, signal=27% |
| 2245 | REACTOME\_GAP\_JUNCTION\_TRAFFICKING |  | 20 | 0.48 | 0.93 | 0.641 | 0.978 | 1.000 | 2527 | tags=25%, list=16%, signal=30% |
| 2246 | KUMAR\_TARGETS\_OF\_MLL\_AF9\_FUSION |  | 359 | 0.41 | 0.93 | 0.836 | 0.979 | 1.000 | 3146 | tags=23%, list=20%, signal=28% |
| 2247 | LANDEMAINE\_LUNG\_METASTASIS |  | 17 | 0.48 | 0.93 | 0.628 | 0.979 | 1.000 | 2795 | tags=18%, list=18%, signal=21% |
| 2248 | REACTOME\_TRANS\_GOLGI\_NETWORK\_VESICLE\_BUDDING |  | 56 | 0.43 | 0.93 | 0.684 | 0.980 | 1.000 | 4286 | tags=45%, list=27%, signal=61% |
| 2249 | YANG\_BREAST\_CANCER\_ESR1\_BULK\_DN |  | 21 | 0.47 | 0.93 | 0.644 | 0.980 | 1.000 | 1918 | tags=33%, list=12%, signal=38% |
| 2250 | REACTOME\_REGULATION\_OF\_INSULIN\_SECRETION\_BY\_GLUCAGON\_LIKE\_PEPTIDE1 |  | 41 | 0.44 | 0.92 | 0.671 | 0.983 | 1.000 | 4853 | tags=32%, list=31%, signal=46% |
| 2251 | KEGG\_ECM\_RECEPTOR\_INTERACTION |  | 82 | 0.43 | 0.92 | 0.710 | 0.983 | 1.000 | 2977 | tags=18%, list=19%, signal=22% |
| 2252 | BIOCARTA\_NTHI\_PATHWAY |  | 23 | 0.47 | 0.92 | 0.632 | 0.983 | 1.000 | 4435 | tags=48%, list=28%, signal=66% |
| 2253 | WANG\_ADIPOGENIC\_GENES\_REPRESSED\_BY\_SIRT1 |  | 26 | 0.46 | 0.92 | 0.660 | 0.983 | 1.000 | 1469 | tags=19%, list=9%, signal=21% |
| 2254 | KEGG\_CARDIAC\_MUSCLE\_CONTRACTION |  | 72 | 0.43 | 0.92 | 0.714 | 0.983 | 1.000 | 993 | tags=11%, list=6%, signal=12% |
| 2255 | SEITZ\_NEOPLASTIC\_TRANSFORMATION\_BY\_8P\_DELETION\_DN |  | 28 | 0.46 | 0.92 | 0.652 | 0.984 | 1.000 | 2592 | tags=25%, list=16%, signal=30% |
| 2256 | RICKMAN\_TUMOR\_DIFFERENTIATED\_WELL\_VS\_MODERATELY\_UP |  | 88 | 0.42 | 0.92 | 0.713 | 0.983 | 1.000 | 4669 | tags=39%, list=30%, signal=55% |
| 2257 | BARRIER\_CANCER\_RELAPSE\_NORMAL\_SAMPLE\_UP |  | 29 | 0.46 | 0.92 | 0.653 | 0.985 | 1.000 | 3932 | tags=34%, list=25%, signal=46% |
| 2258 | REACTOME\_HS\_GAG\_BIOSYNTHESIS |  | 28 | 0.45 | 0.92 | 0.645 | 0.986 | 1.000 | 1574 | tags=18%, list=10%, signal=20% |
| 2259 | BREDEMEYER\_RAG\_SIGNALING\_VIA\_ATM\_NOT\_VIA\_NFKB\_DN |  | 35 | 0.45 | 0.92 | 0.678 | 0.987 | 1.000 | 5694 | tags=46%, list=36%, signal=71% |
| 2260 | SENESE\_HDAC1\_AND\_HDAC2\_TARGETS\_DN |  | 198 | 0.41 | 0.92 | 0.792 | 0.986 | 1.000 | 1806 | tags=16%, list=11%, signal=18% |
| 2261 | BIOCARTA\_AGR\_PATHWAY |  | 36 | 0.44 | 0.92 | 0.664 | 0.987 | 1.000 | 4360 | tags=28%, list=28%, signal=38% |
| 2262 | SASSON\_RESPONSE\_TO\_GONADOTROPHINS\_UP |  | 83 | 0.42 | 0.92 | 0.716 | 0.987 | 1.000 | 3680 | tags=30%, list=23%, signal=39% |
| 2263 | LI\_WILMS\_TUMOR\_VS\_FETAL\_KIDNEY\_1\_UP |  | 174 | 0.41 | 0.92 | 0.773 | 0.987 | 1.000 | 1662 | tags=18%, list=11%, signal=20% |
| 2264 | CASORELLI\_ACUTE\_PROMYELOCYTIC\_LEUKEMIA\_UP |  | 144 | 0.41 | 0.92 | 0.767 | 0.987 | 1.000 | 3605 | tags=27%, list=23%, signal=35% |
| 2265 | WANG\_ESOPHAGUS\_CANCER\_VS\_NORMAL\_DN |  | 93 | 0.42 | 0.92 | 0.734 | 0.987 | 1.000 | 3603 | tags=31%, list=23%, signal=40% |
| 2266 | ODONNELL\_METASTASIS\_DN |  | 24 | 0.46 | 0.92 | 0.652 | 0.986 | 1.000 | 2724 | tags=33%, list=17%, signal=40% |
| 2267 | LIAN\_LIPA\_TARGETS\_3M |  | 56 | 0.43 | 0.92 | 0.695 | 0.987 | 1.000 | 979 | tags=9%, list=6%, signal=9% |
| 2268 | AMUNDSON\_DNA\_DAMAGE\_RESPONSE\_TP53 |  | 15 | 0.49 | 0.92 | 0.652 | 0.986 | 1.000 | 5028 | tags=67%, list=32%, signal=98% |
| 2269 | ZHU\_SKIL\_TARGETS\_UP |  | 20 | 0.47 | 0.92 | 0.659 | 0.986 | 1.000 | 2928 | tags=30%, list=19%, signal=37% |
| 2270 | KEGG\_NOD\_LIKE\_RECEPTOR\_SIGNALING\_PATHWAY |  | 52 | 0.43 | 0.92 | 0.709 | 0.986 | 1.000 | 4725 | tags=38%, list=30%, signal=55% |
| 2271 | ROYLANCE\_BREAST\_CANCER\_16Q\_COPY\_NUMBER\_UP |  | 45 | 0.44 | 0.92 | 0.686 | 0.988 | 1.000 | 3946 | tags=31%, list=25%, signal=41% |
| 2272 | HOEGERKORP\_CD44\_TARGETS\_TEMPORAL\_DN |  | 23 | 0.46 | 0.92 | 0.651 | 0.989 | 1.000 | 4772 | tags=39%, list=30%, signal=56% |
| 2273 | PAPASPYRIDONOS\_UNSTABLE\_ATEROSCLEROTIC\_PLAQUE\_UP |  | 45 | 0.43 | 0.92 | 0.688 | 0.989 | 1.000 | 2855 | tags=24%, list=18%, signal=30% |
| 2274 | HINATA\_NFKB\_TARGETS\_KERATINOCYTE\_UP |  | 87 | 0.42 | 0.92 | 0.737 | 0.988 | 1.000 | 2890 | tags=22%, list=18%, signal=27% |
| 2275 | PID\_BMPPATHWAY |  | 39 | 0.44 | 0.92 | 0.675 | 0.989 | 1.000 | 4113 | tags=38%, list=26%, signal=52% |
| 2276 | VISALA\_AGING\_LYMPHOCYTE\_DN |  | 15 | 0.48 | 0.92 | 0.644 | 0.989 | 1.000 | 2442 | tags=33%, list=16%, signal=39% |
| 2277 | STREICHER\_LSM1\_TARGETS\_DN |  | 17 | 0.48 | 0.92 | 0.655 | 0.988 | 1.000 | 2867 | tags=29%, list=18%, signal=36% |
| 2278 | BOSCO\_TH1\_CYTOTOXIC\_MODULE |  | 96 | 0.42 | 0.92 | 0.733 | 0.988 | 1.000 | 4657 | tags=20%, list=30%, signal=28% |
| 2279 | ROVERSI\_GLIOMA\_COPY\_NUMBER\_DN |  | 48 | 0.43 | 0.92 | 0.694 | 0.988 | 1.000 | 5520 | tags=35%, list=35%, signal=54% |
| 2280 | KEGG\_HYPERTROPHIC\_CARDIOMYOPATHY\_HCM |  | 83 | 0.42 | 0.92 | 0.725 | 0.990 | 1.000 | 1595 | tags=11%, list=10%, signal=12% |
| 2281 | ZHOU\_INFLAMMATORY\_RESPONSE\_LPS\_UP |  | 321 | 0.41 | 0.92 | 0.847 | 0.990 | 1.000 | 3691 | tags=20%, list=23%, signal=26% |
| 2282 | SWEET\_KRAS\_TARGETS\_UP |  | 77 | 0.42 | 0.92 | 0.699 | 0.990 | 1.000 | 3073 | tags=19%, list=20%, signal=24% |
| 2283 | AMIT\_EGF\_RESPONSE\_480\_MCF10A |  | 41 | 0.44 | 0.92 | 0.705 | 0.990 | 1.000 | 4148 | tags=32%, list=26%, signal=43% |
| 2284 | WEINMANN\_ADAPTATION\_TO\_HYPOXIA\_DN |  | 38 | 0.45 | 0.92 | 0.671 | 0.991 | 1.000 | 1261 | tags=16%, list=8%, signal=17% |
| 2285 | REACTOME\_TRAF6\_MEDIATED\_NFKB\_ACTIVATION |  | 19 | 0.47 | 0.92 | 0.651 | 0.992 | 1.000 | 3201 | tags=26%, list=20%, signal=33% |
| 2286 | BIOCARTA\_STRESS\_PATHWAY |  | 25 | 0.45 | 0.92 | 0.650 | 0.992 | 1.000 | 4508 | tags=44%, list=29%, signal=62% |
| 2287 | ST\_INTERLEUKIN\_4\_PATHWAY |  | 24 | 0.46 | 0.91 | 0.662 | 0.993 | 1.000 | 4687 | tags=50%, list=30%, signal=71% |
| 2288 | JI\_CARCINOGENESIS\_BY\_KRAS\_AND\_STK11\_DN |  | 17 | 0.47 | 0.91 | 0.654 | 0.993 | 1.000 | 2577 | tags=29%, list=16%, signal=35% |
| 2289 | LEE\_LIVER\_CANCER\_MYC\_E2F1\_DN |  | 61 | 0.42 | 0.91 | 0.717 | 0.993 | 1.000 | 1661 | tags=13%, list=11%, signal=15% |
| 2290 | BIOCARTA\_INSULIN\_PATHWAY |  | 22 | 0.46 | 0.91 | 0.656 | 0.994 | 1.000 | 5230 | tags=59%, list=33%, signal=88% |
| 2291 | DALESSIO\_TSA\_RESPONSE |  | 20 | 0.46 | 0.91 | 0.663 | 0.994 | 1.000 | 1809 | tags=15%, list=11%, signal=17% |
| 2292 | SABATES\_COLORECTAL\_ADENOMA\_SIZE\_UP |  | 17 | 0.48 | 0.91 | 0.650 | 0.993 | 1.000 | 4558 | tags=47%, list=29%, signal=66% |
| 2293 | SETLUR\_PROSTATE\_CANCER\_TMPRSS2\_ERG\_FUSION\_DN |  | 19 | 0.46 | 0.91 | 0.648 | 0.995 | 1.000 | 239 | tags=5%, list=2%, signal=5% |
| 2294 | ZUCCHI\_METASTASIS\_DN |  | 37 | 0.44 | 0.91 | 0.689 | 0.995 | 1.000 | 1705 | tags=27%, list=11%, signal=30% |
| 2295 | PID\_EPHA2\_FWDPATHWAY |  | 18 | 0.47 | 0.91 | 0.641 | 0.996 | 1.000 | 956 | tags=17%, list=6%, signal=18% |
| 2296 | BIOCARTA\_CXCR4\_PATHWAY |  | 23 | 0.45 | 0.91 | 0.676 | 0.997 | 1.000 | 5031 | tags=52%, list=32%, signal=77% |
| 2297 | WENG\_POR\_TARGETS\_LIVER\_DN |  | 19 | 0.46 | 0.91 | 0.654 | 0.998 | 1.000 | 1492 | tags=16%, list=9%, signal=17% |
| 2298 | KONDO\_PROSTATE\_CANCER\_HCP\_WITH\_H3K27ME3 |  | 76 | 0.42 | 0.91 | 0.736 | 0.997 | 1.000 | 2042 | tags=12%, list=13%, signal=14% |
| 2299 | CHEN\_LIVER\_METABOLISM\_QTL\_CIS |  | 82 | 0.42 | 0.91 | 0.739 | 0.997 | 1.000 | 3979 | tags=32%, list=25%, signal=42% |
| 2300 | YAMAZAKI\_TCEB3\_TARGETS\_UP |  | 163 | 0.41 | 0.91 | 0.796 | 0.997 | 1.000 | 3617 | tags=32%, list=23%, signal=41% |
| 2301 | MATZUK\_SPERMATID\_DIFFERENTIATION |  | 35 | 0.44 | 0.91 | 0.669 | 0.998 | 1.000 | 2515 | tags=20%, list=16%, signal=24% |
| 2302 | BIOCARTA\_TPO\_PATHWAY |  | 23 | 0.46 | 0.91 | 0.673 | 0.999 | 1.000 | 5230 | tags=57%, list=33%, signal=84% |
| 2303 | KEGG\_FATTY\_ACID\_METABOLISM |  | 37 | 0.44 | 0.91 | 0.697 | 0.999 | 1.000 | 3147 | tags=30%, list=20%, signal=37% |
| 2304 | KEGG\_TIGHT\_JUNCTION |  | 125 | 0.41 | 0.91 | 0.780 | 0.999 | 1.000 | 3642 | tags=30%, list=23%, signal=38% |
| 2305 | BOWIE\_RESPONSE\_TO\_EXTRACELLULAR\_MATRIX |  | 15 | 0.48 | 0.91 | 0.643 | 1.000 | 1.000 | 3036 | tags=27%, list=19%, signal=33% |
| 2306 | SASSON\_RESPONSE\_TO\_GONADOTROPHINS\_DN |  | 81 | 0.42 | 0.91 | 0.738 | 1.000 | 1.000 | 3555 | tags=37%, list=23%, signal=48% |
| 2307 | LEE\_CALORIE\_RESTRICTION\_MUSCLE\_UP |  | 38 | 0.43 | 0.91 | 0.696 | 1.000 | 1.000 | 1498 | tags=18%, list=10%, signal=20% |
| 2308 | KEGG\_PHENYLALANINE\_METABOLISM |  | 18 | 0.47 | 0.91 | 0.654 | 1.000 | 1.000 | 976 | tags=17%, list=6%, signal=18% |
| 2309 | MCBRYAN\_PUBERTAL\_TGFB1\_TARGETS\_UP |  | 164 | 0.41 | 0.91 | 0.811 | 1.000 | 1.000 | 2938 | tags=26%, list=19%, signal=31% |
| 2310 | KUMAR\_PATHOGEN\_LOAD\_BY\_MACROPHAGES |  | 199 | 0.41 | 0.91 | 0.837 | 1.000 | 1.000 | 4935 | tags=33%, list=31%, signal=48% |
| 2311 | PETROVA\_PROX1\_TARGETS\_DN |  | 62 | 0.42 | 0.91 | 0.733 | 1.000 | 1.000 | 4553 | tags=40%, list=29%, signal=56% |
| 2312 | DEBIASI\_APOPTOSIS\_BY\_REOVIRUS\_INFECTION\_DN |  | 252 | 0.41 | 0.91 | 0.858 | 1.000 | 1.000 | 3359 | tags=28%, list=21%, signal=35% |
| 2313 | PID\_AR\_NONGENOMIC\_PATHWAY |  | 31 | 0.44 | 0.91 | 0.672 | 1.000 | 1.000 | 5031 | tags=48%, list=32%, signal=71% |
| 2314 | ULE\_SPLICING\_VIA\_NOVA2 |  | 43 | 0.43 | 0.91 | 0.709 | 1.000 | 1.000 | 4208 | tags=47%, list=27%, signal=63% |
| 2315 | SAMOLS\_TARGETS\_OF\_KHSV\_MIRNAS\_DN |  | 50 | 0.42 | 0.91 | 0.724 | 1.000 | 1.000 | 3112 | tags=30%, list=20%, signal=37% |
| 2316 | GRADE\_COLON\_CANCER\_DN |  | 25 | 0.45 | 0.91 | 0.682 | 1.000 | 1.000 | 4963 | tags=48%, list=32%, signal=70% |
| 2317 | ELLWOOD\_MYC\_TARGETS\_DN |  | 32 | 0.44 | 0.91 | 0.681 | 1.000 | 1.000 | 3606 | tags=31%, list=23%, signal=40% |
| 2318 | KEGG\_TOLL\_LIKE\_RECEPTOR\_SIGNALING\_PATHWAY |  | 89 | 0.42 | 0.90 | 0.754 | 1.000 | 1.000 | 5041 | tags=36%, list=32%, signal=53% |
| 2319 | HOFMANN\_CELL\_LYMPHOMA\_DN |  | 37 | 0.44 | 0.90 | 0.684 | 1.000 | 1.000 | 4470 | tags=32%, list=28%, signal=45% |
| 2320 | MODY\_HIPPOCAMPUS\_POSTNATAL |  | 61 | 0.42 | 0.90 | 0.724 | 1.000 | 1.000 | 4466 | tags=33%, list=28%, signal=46% |
| 2321 | REACTOME\_PLATELET\_ACTIVATION\_SIGNALING\_AND\_AGGREGATION |  | 193 | 0.41 | 0.90 | 0.826 | 1.000 | 1.000 | 4098 | tags=25%, list=26%, signal=34% |
| 2322 | ACEVEDO\_FGFR1\_TARGETS\_IN\_PROSTATE\_CANCER\_MODEL\_UP |  | 257 | 0.40 | 0.90 | 0.844 | 1.000 | 1.000 | 2989 | tags=25%, list=19%, signal=31% |
| 2323 | REACTOME\_PRE\_NOTCH\_EXPRESSION\_AND\_PROCESSING |  | 38 | 0.43 | 0.90 | 0.705 | 1.000 | 1.000 | 3416 | tags=32%, list=22%, signal=40% |
| 2324 | SUH\_COEXPRESSED\_WITH\_ID1\_AND\_ID2\_UP |  | 17 | 0.47 | 0.90 | 0.667 | 1.000 | 1.000 | 2792 | tags=35%, list=18%, signal=43% |
| 2325 | LENAOUR\_DENDRITIC\_CELL\_MATURATION\_UP |  | 104 | 0.41 | 0.90 | 0.772 | 1.000 | 1.000 | 1550 | tags=18%, list=10%, signal=20% |
| 2326 | JOHNSTONE\_PARVB\_TARGETS\_3\_UP |  | 376 | 0.40 | 0.90 | 0.914 | 1.000 | 1.000 | 3062 | tags=25%, list=19%, signal=31% |
| 2327 | CHEOK\_RESPONSE\_TO\_MERCAPTOPURINE\_DN |  | 19 | 0.46 | 0.90 | 0.671 | 1.000 | 1.000 | 2762 | tags=32%, list=18%, signal=38% |
| 2328 | REACTOME\_ADP\_SIGNALLING\_THROUGH\_P2RY12 |  | 21 | 0.46 | 0.90 | 0.674 | 1.000 | 1.000 | 5784 | tags=38%, list=37%, signal=60% |
| 2329 | FOSTER\_TOLERANT\_MACROPHAGE\_UP |  | 139 | 0.41 | 0.90 | 0.797 | 1.000 | 1.000 | 4472 | tags=27%, list=28%, signal=38% |
| 2330 | ELVIDGE\_HYPOXIA\_BY\_DMOG\_DN |  | 53 | 0.42 | 0.90 | 0.738 | 1.000 | 1.000 | 2930 | tags=30%, list=19%, signal=37% |
| 2331 | BIOCARTA\_EGF\_PATHWAY |  | 30 | 0.44 | 0.90 | 0.699 | 1.000 | 1.000 | 5230 | tags=57%, list=33%, signal=85% |
| 2332 | CHIBA\_RESPONSE\_TO\_TSA |  | 44 | 0.43 | 0.90 | 0.723 | 1.000 | 1.000 | 2641 | tags=30%, list=17%, signal=35% |
| 2333 | VALK\_AML\_CLUSTER\_1 |  | 25 | 0.45 | 0.90 | 0.678 | 1.000 | 1.000 | 2667 | tags=20%, list=17%, signal=24% |
| 2334 | PID\_AR\_TF\_PATHWAY |  | 49 | 0.42 | 0.90 | 0.720 | 1.000 | 1.000 | 5174 | tags=49%, list=33%, signal=73% |
| 2335 | TURASHVILI\_BREAST\_CARCINOMA\_DUCTAL\_VS\_LOBULAR\_UP |  | 16 | 0.48 | 0.90 | 0.672 | 1.000 | 1.000 | 3470 | tags=44%, list=22%, signal=56% |
| 2336 | REACTOME\_OLFACTORY\_SIGNALING\_PATHWAY |  | 235 | 0.40 | 0.90 | 0.843 | 1.000 | 1.000 | 3457 | tags=0%, list=22%, signal=1% |
| 2337 | LEE\_NEURAL\_CREST\_STEM\_CELL\_DN |  | 105 | 0.41 | 0.90 | 0.784 | 1.000 | 1.000 | 2358 | tags=15%, list=15%, signal=18% |
| 2338 | REACTOME\_ADHERENS\_JUNCTIONS\_INTERACTIONS |  | 27 | 0.45 | 0.90 | 0.698 | 1.000 | 1.000 | 1037 | tags=15%, list=7%, signal=16% |
| 2339 | WAKABAYASHI\_ADIPOGENESIS\_PPARG\_RXRA\_BOUND\_36HR |  | 126 | 0.41 | 0.90 | 0.809 | 1.000 | 1.000 | 3746 | tags=31%, list=24%, signal=40% |
| 2340 | CERIBELLI\_GENES\_INACTIVE\_AND\_BOUND\_BY\_NFY |  | 24 | 0.45 | 0.90 | 0.686 | 1.000 | 1.000 | 1585 | tags=8%, list=10%, signal=9% |
| 2341 | LIAN\_LIPA\_TARGETS\_6M |  | 71 | 0.41 | 0.90 | 0.740 | 1.000 | 1.000 | 2134 | tags=11%, list=14%, signal=13% |
| 2342 | REACTOME\_GOLGI\_ASSOCIATED\_VESICLE\_BIOGENESIS |  | 49 | 0.42 | 0.90 | 0.720 | 1.000 | 1.000 | 4286 | tags=45%, list=27%, signal=61% |
| 2343 | BIOCARTA\_NGF\_PATHWAY |  | 17 | 0.46 | 0.90 | 0.686 | 1.000 | 1.000 | 5230 | tags=65%, list=33%, signal=97% |
| 2344 | KEGG\_GAP\_JUNCTION |  | 79 | 0.41 | 0.90 | 0.751 | 1.000 | 1.000 | 4729 | tags=33%, list=30%, signal=47% |
| 2345 | LI\_CISPLATIN\_RESISTANCE\_DN |  | 27 | 0.45 | 0.90 | 0.687 | 1.000 | 1.000 | 2269 | tags=11%, list=14%, signal=13% |
| 2346 | VALK\_AML\_WITH\_FLT3\_ITD |  | 35 | 0.43 | 0.90 | 0.695 | 1.000 | 1.000 | 1 | tags=3%, list=0%, signal=3% |
| 2347 | FOURNIER\_ACINAR\_DEVELOPMENT\_EARLY\_UP |  | 17 | 0.47 | 0.90 | 0.669 | 1.000 | 1.000 | 2482 | tags=29%, list=16%, signal=35% |
| 2348 | SHIN\_B\_CELL\_LYMPHOMA\_CLUSTER\_2 |  | 29 | 0.44 | 0.90 | 0.698 | 1.000 | 1.000 | 1100 | tags=10%, list=7%, signal=11% |
| 2349 | GAL\_LEUKEMIC\_STEM\_CELL\_UP |  | 111 | 0.41 | 0.90 | 0.800 | 1.000 | 1.000 | 5508 | tags=41%, list=35%, signal=63% |
| 2350 | WNT\_SIGNALING |  | 89 | 0.41 | 0.90 | 0.771 | 1.000 | 1.000 | 4295 | tags=28%, list=27%, signal=38% |
| 2351 | VANDESLUIS\_COMMD1\_TARGETS\_GROUP\_4\_DN |  | 16 | 0.47 | 0.90 | 0.685 | 1.000 | 1.000 | 825 | tags=13%, list=5%, signal=13% |
| 2352 | REACTOME\_REGULATION\_OF\_WATER\_BALANCE\_BY\_RENAL\_AQUAPORINS |  | 42 | 0.43 | 0.90 | 0.730 | 1.000 | 1.000 | 5784 | tags=40%, list=37%, signal=64% |
| 2353 | ZIRN\_TRETINOIN\_RESPONSE\_WT1\_UP |  | 20 | 0.46 | 0.90 | 0.676 | 1.000 | 1.000 | 4138 | tags=45%, list=26%, signal=61% |
| 2354 | REACTOME\_TRIGLYCERIDE\_BIOSYNTHESIS |  | 31 | 0.44 | 0.90 | 0.709 | 1.000 | 1.000 | 3303 | tags=29%, list=21%, signal=37% |
| 2355 | NEWMAN\_ERCC6\_TARGETS\_DN |  | 31 | 0.43 | 0.90 | 0.707 | 1.000 | 1.000 | 4553 | tags=29%, list=29%, signal=41% |
| 2356 | ASTIER\_INTEGRIN\_SIGNALING |  | 51 | 0.42 | 0.90 | 0.718 | 1.000 | 1.000 | 1683 | tags=20%, list=11%, signal=22% |
| 2357 | REACTOME\_SIGNALING\_BY\_NOTCH |  | 93 | 0.41 | 0.90 | 0.778 | 1.000 | 1.000 | 2225 | tags=20%, list=14%, signal=24% |
| 2358 | MATZUK\_SPERMATOZOA |  | 107 | 0.41 | 0.89 | 0.806 | 1.000 | 1.000 | 4030 | tags=22%, list=26%, signal=30% |
| 2359 | REACTOME\_PKA\_MEDIATED\_PHOSPHORYLATION\_OF\_CREB |  | 16 | 0.47 | 0.89 | 0.692 | 1.000 | 1.000 | 3830 | tags=31%, list=24%, signal=41% |
| 2360 | REACTOME\_INWARDLY\_RECTIFYING\_K\_CHANNELS |  | 31 | 0.44 | 0.89 | 0.713 | 1.000 | 1.000 | 5784 | tags=26%, list=37%, signal=41% |
| 2361 | BIOCARTA\_EDG1\_PATHWAY |  | 24 | 0.45 | 0.89 | 0.701 | 1.000 | 1.000 | 4098 | tags=42%, list=26%, signal=56% |
| 2362 | REACTOME\_INNATE\_IMMUNE\_SYSTEM |  | 212 | 0.40 | 0.89 | 0.882 | 1.000 | 1.000 | 3768 | tags=25%, list=24%, signal=33% |
| 2363 | GUENTHER\_GROWTH\_SPHERICAL\_VS\_ADHERENT\_UP |  | 21 | 0.45 | 0.89 | 0.689 | 1.000 | 1.000 | 1507 | tags=14%, list=10%, signal=16% |
| 2364 | PANGAS\_TUMOR\_SUPPRESSION\_BY\_SMAD1\_AND\_SMAD5\_DN |  | 131 | 0.41 | 0.89 | 0.824 | 1.000 | 1.000 | 4102 | tags=31%, list=26%, signal=42% |
| 2365 | KEGG\_TYROSINE\_METABOLISM |  | 38 | 0.43 | 0.89 | 0.719 | 1.000 | 1.000 | 976 | tags=13%, list=6%, signal=14% |
| 2366 | REACTOME\_LYSOSOME\_VESICLE\_BIOGENESIS |  | 21 | 0.45 | 0.89 | 0.687 | 1.000 | 1.000 | 1944 | tags=29%, list=12%, signal=33% |
| 2367 | ZHANG\_TARGETS\_OF\_EWSR1\_FLI1\_FUSION |  | 75 | 0.41 | 0.89 | 0.774 | 1.000 | 1.000 | 4993 | tags=49%, list=32%, signal=72% |
| 2368 | SANA\_TNF\_SIGNALING\_DN |  | 81 | 0.41 | 0.89 | 0.774 | 1.000 | 1.000 | 2978 | tags=30%, list=19%, signal=36% |
| 2369 | ALCALAY\_AML\_BY\_NPM1\_LOCALIZATION\_UP |  | 127 | 0.40 | 0.89 | 0.809 | 1.000 | 1.000 | 3767 | tags=25%, list=24%, signal=33% |
| 2370 | ROSS\_AML\_WITH\_CBFB\_MYH11\_FUSION |  | 45 | 0.42 | 0.89 | 0.728 | 1.000 | 1.000 | 1183 | tags=11%, list=8%, signal=12% |
| 2371 | BROWNE\_HCMV\_INFECTION\_1HR\_UP |  | 55 | 0.42 | 0.89 | 0.730 | 1.000 | 1.000 | 3776 | tags=20%, list=24%, signal=26% |
| 2372 | REACTOME\_GLYCOGEN\_BREAKDOWN\_GLYCOGENOLYSIS |  | 15 | 0.47 | 0.89 | 0.681 | 1.000 | 1.000 | 3533 | tags=33%, list=22%, signal=43% |
| 2373 | REACTOME\_REGULATION\_OF\_INSULIN\_SECRETION |  | 87 | 0.41 | 0.89 | 0.777 | 1.000 | 1.000 | 4871 | tags=31%, list=31%, signal=45% |
| 2374 | SEITZ\_NEOPLASTIC\_TRANSFORMATION\_BY\_8P\_DELETION\_UP |  | 61 | 0.41 | 0.89 | 0.741 | 1.000 | 1.000 | 3036 | tags=25%, list=19%, signal=30% |
| 2375 | ABE\_VEGFA\_TARGETS |  | 18 | 0.46 | 0.89 | 0.663 | 1.000 | 1.000 | 1699 | tags=22%, list=11%, signal=25% |
| 2376 | BILD\_HRAS\_ONCOGENIC\_SIGNATURE |  | 225 | 0.40 | 0.89 | 0.872 | 1.000 | 1.000 | 3644 | tags=26%, list=23%, signal=34% |
| 2377 | HOFFMANN\_SMALL\_PRE\_BII\_TO\_IMMATURE\_B\_LYMPHOCYTE\_UP |  | 66 | 0.42 | 0.89 | 0.777 | 1.000 | 1.000 | 1100 | tags=14%, list=7%, signal=15% |
| 2378 | DEMAGALHAES\_AGING\_UP |  | 50 | 0.42 | 0.89 | 0.737 | 1.000 | 1.000 | 1454 | tags=24%, list=9%, signal=26% |
| 2379 | OKAWA\_NEUROBLASTOMA\_1P36\_31\_DELETION |  | 22 | 0.45 | 0.89 | 0.690 | 1.000 | 1.000 | 4889 | tags=36%, list=31%, signal=53% |
| 2380 | YANG\_BREAST\_CANCER\_ESR1\_DN |  | 24 | 0.45 | 0.89 | 0.705 | 1.000 | 1.000 | 2246 | tags=21%, list=14%, signal=24% |
| 2381 | JAZAG\_TGFB1\_SIGNALING\_VIA\_SMAD4\_DN |  | 60 | 0.41 | 0.89 | 0.748 | 1.000 | 1.000 | 3573 | tags=33%, list=23%, signal=43% |
| 2382 | KEGG\_ADIPOCYTOKINE\_SIGNALING\_PATHWAY |  | 66 | 0.41 | 0.89 | 0.758 | 1.000 | 1.000 | 4725 | tags=30%, list=30%, signal=43% |
| 2383 | KEGG\_PENTOSE\_AND\_GLUCURONATE\_INTERCONVERSIONS |  | 20 | 0.46 | 0.89 | 0.681 | 1.000 | 1.000 | 2202 | tags=20%, list=14%, signal=23% |
| 2384 | BIOCARTA\_41BB\_PATHWAY |  | 17 | 0.46 | 0.89 | 0.696 | 1.000 | 1.000 | 5335 | tags=53%, list=34%, signal=80% |
| 2385 | STARK\_HYPPOCAMPUS\_22Q11\_DELETION\_UP |  | 45 | 0.42 | 0.89 | 0.737 | 1.000 | 1.000 | 2920 | tags=20%, list=19%, signal=24% |
| 2386 | DUTERTRE\_ESTRADIOL\_RESPONSE\_24HR\_DN |  | 443 | 0.39 | 0.89 | 0.955 | 1.000 | 1.000 | 3008 | tags=21%, list=19%, signal=25% |
| 2387 | TORCHIA\_TARGETS\_OF\_EWSR1\_FLI1\_FUSION\_DN |  | 285 | 0.39 | 0.89 | 0.911 | 1.000 | 1.000 | 4271 | tags=27%, list=27%, signal=36% |
| 2388 | SABATES\_COLORECTAL\_ADENOMA\_UP |  | 118 | 0.40 | 0.89 | 0.827 | 1.000 | 1.000 | 1865 | tags=13%, list=12%, signal=14% |
| 2389 | RICKMAN\_TUMOR\_DIFFERENTIATED\_WELL\_VS\_POORLY\_DN |  | 321 | 0.40 | 0.89 | 0.926 | 1.000 | 1.000 | 3908 | tags=29%, list=25%, signal=38% |
| 2390 | NIKOLSKY\_BREAST\_CANCER\_8Q23\_Q24\_AMPLICON |  | 121 | 0.40 | 0.89 | 0.824 | 1.000 | 1.000 | 2666 | tags=19%, list=17%, signal=23% |
| 2391 | BASSO\_CD40\_SIGNALING\_UP |  | 90 | 0.40 | 0.89 | 0.796 | 1.000 | 1.000 | 271 | tags=7%, list=2%, signal=7% |
| 2392 | WANG\_LSD1\_TARGETS\_DN |  | 38 | 0.42 | 0.88 | 0.706 | 1.000 | 1.000 | 3025 | tags=21%, list=19%, signal=26% |
| 2393 | BASAKI\_YBX1\_TARGETS\_DN |  | 314 | 0.40 | 0.88 | 0.916 | 1.000 | 1.000 | 4236 | tags=33%, list=27%, signal=44% |
| 2394 | WANG\_SMARCE1\_TARGETS\_UP |  | 249 | 0.39 | 0.88 | 0.896 | 1.000 | 1.000 | 3207 | tags=21%, list=20%, signal=26% |
| 2395 | VERHAAK\_AML\_WITH\_NPM1\_MUTATED\_UP |  | 163 | 0.40 | 0.88 | 0.858 | 1.000 | 1.000 | 2656 | tags=15%, list=17%, signal=18% |
| 2396 | HASLINGER\_B\_CLL\_WITH\_13Q14\_DELETION |  | 21 | 0.45 | 0.88 | 0.702 | 1.000 | 1.000 | 2925 | tags=19%, list=19%, signal=23% |
| 2397 | REACTOME\_PROSTACYCLIN\_SIGNALLING\_THROUGH\_PROSTACYCLIN\_RECEPTOR |  | 19 | 0.45 | 0.88 | 0.702 | 1.000 | 1.000 | 5784 | tags=42%, list=37%, signal=66% |
| 2398 | BIOCARTA\_TALL1\_PATHWAY |  | 15 | 0.47 | 0.88 | 0.697 | 1.000 | 1.000 | 5680 | tags=53%, list=36%, signal=83% |
| 2399 | MIKKELSEN\_ES\_HCP\_WITH\_H3K27ME3 |  | 40 | 0.42 | 0.88 | 0.739 | 1.000 | 1.000 | 2313 | tags=5%, list=15%, signal=6% |
| 2400 | KYNG\_ENVIRONMENTAL\_STRESS\_RESPONSE\_NOT\_BY\_UV\_IN\_OLD |  | 23 | 0.45 | 0.88 | 0.706 | 1.000 | 1.000 | 2482 | tags=26%, list=16%, signal=31% |
| 2401 | HOFFMANN\_IMMATURE\_TO\_MATURE\_B\_LYMPHOCYTE\_DN |  | 46 | 0.42 | 0.88 | 0.731 | 1.000 | 1.000 | 3780 | tags=30%, list=24%, signal=40% |
| 2402 | ST\_G\_ALPHA\_I\_PATHWAY |  | 34 | 0.43 | 0.88 | 0.724 | 1.000 | 1.000 | 5230 | tags=50%, list=33%, signal=75% |
| 2403 | JOHANSSON\_GLIOMAGENESIS\_BY\_PDGFB\_DN |  | 18 | 0.46 | 0.88 | 0.689 | 1.000 | 1.000 | 1891 | tags=17%, list=12%, signal=19% |
| 2404 | LIM\_MAMMARY\_LUMINAL\_MATURE\_DN |  | 92 | 0.40 | 0.88 | 0.787 | 1.000 | 1.000 | 3643 | tags=27%, list=23%, signal=35% |
| 2405 | DARWICHE\_PAPILLOMA\_PROGRESSION\_RISK |  | 63 | 0.41 | 0.88 | 0.779 | 1.000 | 1.000 | 4994 | tags=25%, list=32%, signal=37% |
| 2406 | CERIBELLI\_PROMOTERS\_INACTIVE\_AND\_BOUND\_BY\_NFY |  | 25 | 0.43 | 0.88 | 0.718 | 1.000 | 1.000 | 2878 | tags=16%, list=18%, signal=20% |
| 2407 | UROSEVIC\_RESPONSE\_TO\_IMIQUIMOD |  | 19 | 0.46 | 0.88 | 0.693 | 1.000 | 1.000 | 2844 | tags=21%, list=18%, signal=26% |
| 2408 | MASRI\_RESISTANCE\_TO\_TAMOXIFEN\_AND\_AROMATASE\_INHIBITORS\_DN |  | 19 | 0.46 | 0.88 | 0.703 | 1.000 | 1.000 | 0 | tags=5%, list=0%, signal=5% |
| 2409 | OZEN\_MIR125B1\_TARGETS |  | 23 | 0.44 | 0.88 | 0.699 | 1.000 | 1.000 | 3312 | tags=30%, list=21%, signal=38% |
| 2410 | REACTOME\_RIP\_MEDIATED\_NFKB\_ACTIVATION\_VIA\_DAI |  | 17 | 0.46 | 0.88 | 0.699 | 1.000 | 1.000 | 3295 | tags=29%, list=21%, signal=37% |
| 2411 | MULLIGHAN\_MLL\_SIGNATURE\_1\_DN |  | 207 | 0.39 | 0.88 | 0.891 | 1.000 | 1.000 | 3162 | tags=26%, list=20%, signal=32% |
| 2412 | ST\_B\_CELL\_ANTIGEN\_RECEPTOR |  | 37 | 0.42 | 0.88 | 0.732 | 1.000 | 1.000 | 5410 | tags=46%, list=34%, signal=70% |
| 2413 | CHIARADONNA\_NEOPLASTIC\_TRANSFORMATION\_CDC25\_DN |  | 146 | 0.40 | 0.88 | 0.857 | 1.000 | 1.000 | 2963 | tags=28%, list=19%, signal=34% |
| 2414 | BOYAULT\_LIVER\_CANCER\_SUBCLASS\_G1\_UP |  | 95 | 0.40 | 0.88 | 0.789 | 1.000 | 1.000 | 3229 | tags=32%, list=21%, signal=39% |
| 2415 | BAKKER\_FOXO3\_TARGETS\_UP |  | 52 | 0.41 | 0.88 | 0.754 | 1.000 | 1.000 | 3776 | tags=31%, list=24%, signal=40% |
| 2416 | REACTOME\_CELL\_SURFACE\_INTERACTIONS\_AT\_THE\_VASCULAR\_WALL |  | 80 | 0.40 | 0.88 | 0.799 | 1.000 | 1.000 | 3328 | tags=23%, list=21%, signal=28% |
| 2417 | BIOCARTA\_MAPK\_PATHWAY |  | 86 | 0.40 | 0.88 | 0.804 | 1.000 | 1.000 | 5391 | tags=56%, list=34%, signal=84% |
| 2418 | VERRECCHIA\_RESPONSE\_TO\_TGFB1\_C5 |  | 21 | 0.44 | 0.88 | 0.725 | 1.000 | 1.000 | 3952 | tags=38%, list=25%, signal=51% |
| 2419 | BOHN\_PRIMARY\_IMMUNODEFICIENCY\_SYNDROM\_DN |  | 37 | 0.42 | 0.88 | 0.743 | 1.000 | 1.000 | 1520 | tags=11%, list=10%, signal=12% |
| 2420 | HOOI\_ST7\_TARGETS\_DN |  | 94 | 0.40 | 0.88 | 0.811 | 1.000 | 1.000 | 3722 | tags=27%, list=24%, signal=35% |
| 2421 | HOFFMANN\_PRE\_BI\_TO\_LARGE\_PRE\_BII\_LYMPHOCYTE\_DN |  | 70 | 0.40 | 0.88 | 0.792 | 1.000 | 1.000 | 4429 | tags=37%, list=28%, signal=51% |
| 2422 | KONDO\_EZH2\_TARGETS |  | 195 | 0.39 | 0.88 | 0.878 | 1.000 | 1.000 | 3412 | tags=21%, list=22%, signal=27% |
| 2423 | YOSHIOKA\_LIVER\_CANCER\_EARLY\_RECURRENCE\_UP |  | 30 | 0.42 | 0.88 | 0.721 | 1.000 | 1.000 | 5182 | tags=37%, list=33%, signal=55% |
| 2424 | REACTOME\_COSTIMULATION\_BY\_THE\_CD28\_FAMILY |  | 55 | 0.41 | 0.88 | 0.771 | 1.000 | 1.000 | 4185 | tags=33%, list=27%, signal=44% |
| 2425 | STEARMAN\_TUMOR\_FIELD\_EFFECT\_UP |  | 34 | 0.42 | 0.88 | 0.735 | 1.000 | 1.000 | 1732 | tags=15%, list=11%, signal=16% |
| 2426 | SCHUETZ\_BREAST\_CANCER\_DUCTAL\_INVASIVE\_UP |  | 317 | 0.39 | 0.88 | 0.935 | 1.000 | 1.000 | 2778 | tags=19%, list=18%, signal=23% |
| 2427 | STEIN\_ESTROGEN\_RESPONSE\_NOT\_VIA\_ESRRA |  | 17 | 0.46 | 0.88 | 0.689 | 1.000 | 1.000 | 334 | tags=12%, list=2%, signal=12% |
| 2428 | PHONG\_TNF\_RESPONSE\_NOT\_VIA\_P38 |  | 309 | 0.39 | 0.88 | 0.928 | 1.000 | 1.000 | 4337 | tags=31%, list=28%, signal=42% |
| 2429 | BOYAULT\_LIVER\_CANCER\_SUBCLASS\_G1\_DN |  | 35 | 0.42 | 0.88 | 0.735 | 1.000 | 1.000 | 4340 | tags=40%, list=28%, signal=55% |
| 2430 | BIOCARTA\_BIOPEPTIDES\_PATHWAY |  | 41 | 0.42 | 0.87 | 0.742 | 1.000 | 1.000 | 5230 | tags=44%, list=33%, signal=66% |
| 2431 | VALK\_AML\_WITH\_CEBPA |  | 31 | 0.42 | 0.87 | 0.722 | 1.000 | 1.000 | 4268 | tags=32%, list=27%, signal=44% |
| 2432 | NIELSEN\_LEIOMYOSARCOMA\_UP |  | 15 | 0.46 | 0.87 | 0.717 | 1.000 | 1.000 | 113 | tags=7%, list=1%, signal=7% |
| 2433 | ST\_ADRENERGIC |  | 34 | 0.42 | 0.87 | 0.734 | 1.000 | 1.000 | 5167 | tags=44%, list=33%, signal=66% |
| 2434 | HEIDENBLAD\_AMPLICON\_8Q24\_DN |  | 38 | 0.42 | 0.87 | 0.762 | 1.000 | 1.000 | 3736 | tags=37%, list=24%, signal=48% |
| 2435 | SCHAEFFER\_PROSTATE\_DEVELOPMENT\_48HR\_DN |  | 385 | 0.39 | 0.87 | 0.952 | 1.000 | 1.000 | 3135 | tags=18%, list=20%, signal=22% |
| 2436 | SHIN\_B\_CELL\_LYMPHOMA\_CLUSTER\_3 |  | 28 | 0.43 | 0.87 | 0.719 | 1.000 | 1.000 | 3286 | tags=25%, list=21%, signal=32% |
| 2437 | TSAI\_RESPONSE\_TO\_RADIATION\_THERAPY |  | 30 | 0.43 | 0.87 | 0.741 | 1.000 | 1.000 | 3297 | tags=30%, list=21%, signal=38% |
| 2438 | CROONQUIST\_NRAS\_VS\_STROMAL\_STIMULATION\_UP |  | 37 | 0.42 | 0.87 | 0.762 | 1.000 | 1.000 | 3922 | tags=30%, list=25%, signal=39% |
| 2439 | ZHAN\_MULTIPLE\_MYELOMA\_PR\_DN |  | 38 | 0.41 | 0.87 | 0.743 | 1.000 | 1.000 | 5820 | tags=42%, list=37%, signal=67% |
| 2440 | REACTOME\_CIRCADIAN\_REPRESSION\_OF\_EXPRESSION\_BY\_REV\_ERBA |  | 22 | 0.44 | 0.87 | 0.717 | 1.000 | 1.000 | 5480 | tags=50%, list=35%, signal=77% |
| 2441 | DAVICIONI\_PAX\_FOXO1\_SIGNATURE\_IN\_ARMS\_UP |  | 54 | 0.40 | 0.87 | 0.757 | 1.000 | 1.000 | 3514 | tags=20%, list=22%, signal=26% |
| 2442 | MIYAGAWA\_TARGETS\_OF\_EWSR1\_ETS\_FUSIONS\_DN |  | 190 | 0.39 | 0.87 | 0.877 | 1.000 | 1.000 | 4553 | tags=30%, list=29%, signal=42% |
| 2443 | LIU\_CMYB\_TARGETS\_UP |  | 145 | 0.39 | 0.87 | 0.858 | 1.000 | 1.000 | 3707 | tags=32%, list=24%, signal=41% |
| 2444 | KEGG\_INSULIN\_SIGNALING\_PATHWAY |  | 132 | 0.39 | 0.87 | 0.862 | 1.000 | 1.000 | 4879 | tags=37%, list=31%, signal=53% |
| 2445 | WEINMANN\_ADAPTATION\_TO\_HYPOXIA\_UP |  | 27 | 0.43 | 0.87 | 0.735 | 1.000 | 1.000 | 3605 | tags=33%, list=23%, signal=43% |
| 2446 | PID\_A6B1\_A6B4\_INTEGRIN\_PATHWAY |  | 46 | 0.41 | 0.87 | 0.750 | 1.000 | 1.000 | 3066 | tags=30%, list=19%, signal=38% |
| 2447 | LEONARD\_HYPOXIA |  | 40 | 0.42 | 0.87 | 0.777 | 1.000 | 1.000 | 3491 | tags=28%, list=22%, signal=35% |
| 2448 | WU\_ALZHEIMER\_DISEASE\_DN |  | 16 | 0.46 | 0.87 | 0.721 | 1.000 | 1.000 | 2746 | tags=31%, list=17%, signal=38% |
| 2449 | PLASARI\_TGFB1\_SIGNALING\_VIA\_NFIC\_1HR\_DN |  | 93 | 0.40 | 0.87 | 0.811 | 1.000 | 1.000 | 3650 | tags=33%, list=23%, signal=43% |
| 2450 | HOELZEL\_NF1\_TARGETS\_UP |  | 122 | 0.39 | 0.87 | 0.852 | 1.000 | 1.000 | 3306 | tags=17%, list=21%, signal=22% |
| 2451 | WILCOX\_PRESPONSE\_TO\_ROGESTERONE\_DN |  | 57 | 0.40 | 0.87 | 0.770 | 1.000 | 1.000 | 2977 | tags=30%, list=19%, signal=37% |
| 2452 | CHEN\_LVAD\_SUPPORT\_OF\_FAILING\_HEART\_UP |  | 91 | 0.40 | 0.87 | 0.834 | 1.000 | 1.000 | 3491 | tags=24%, list=22%, signal=31% |
| 2453 | BURTON\_ADIPOGENESIS\_6 |  | 174 | 0.39 | 0.87 | 0.890 | 1.000 | 1.000 | 3258 | tags=28%, list=21%, signal=34% |
| 2454 | BIOCARTA\_TOLL\_PATHWAY |  | 34 | 0.42 | 0.87 | 0.747 | 1.000 | 1.000 | 5879 | tags=56%, list=37%, signal=89% |
| 2455 | KEGG\_NATURAL\_KILLER\_CELL\_MEDIATED\_CYTOTOXICITY |  | 103 | 0.39 | 0.87 | 0.855 | 1.000 | 1.000 | 3666 | tags=17%, list=23%, signal=21% |
| 2456 | WOO\_LIVER\_CANCER\_RECURRENCE\_UP |  | 97 | 0.40 | 0.87 | 0.833 | 1.000 | 1.000 | 3416 | tags=27%, list=22%, signal=34% |
| 2457 | PICCALUGA\_ANGIOIMMUNOBLASTIC\_LYMPHOMA\_UP |  | 187 | 0.39 | 0.87 | 0.907 | 1.000 | 1.000 | 3570 | tags=28%, list=23%, signal=36% |
| 2458 | REACTOME\_IL\_2\_SIGNALING |  | 40 | 0.42 | 0.87 | 0.762 | 1.000 | 1.000 | 3328 | tags=23%, list=21%, signal=28% |
| 2459 | KEGG\_VASCULAR\_SMOOTH\_MUSCLE\_CONTRACTION |  | 108 | 0.39 | 0.87 | 0.851 | 1.000 | 1.000 | 956 | tags=8%, list=6%, signal=9% |
| 2460 | GAVIN\_FOXP3\_TARGETS\_CLUSTER\_P4 |  | 88 | 0.40 | 0.87 | 0.821 | 1.000 | 1.000 | 2952 | tags=20%, list=19%, signal=25% |
| 2461 | ELVIDGE\_HIF1A\_AND\_HIF2A\_TARGETS\_DN |  | 93 | 0.39 | 0.87 | 0.828 | 1.000 | 1.000 | 1683 | tags=19%, list=11%, signal=22% |
| 2462 | PID\_IFNGPATHWAY |  | 38 | 0.41 | 0.87 | 0.764 | 1.000 | 1.000 | 4281 | tags=39%, list=27%, signal=54% |
| 2463 | NIELSEN\_GIST\_VS\_SYNOVIAL\_SARCOMA\_UP |  | 18 | 0.45 | 0.87 | 0.716 | 1.000 | 1.000 | 1357 | tags=17%, list=9%, signal=18% |
| 2464 | MATZUK\_IMPLANTATION\_AND\_UTERINE |  | 19 | 0.45 | 0.86 | 0.721 | 1.000 | 1.000 | 891 | tags=16%, list=6%, signal=17% |
| 2465 | HOUSTIS\_ROS |  | 33 | 0.42 | 0.86 | 0.755 | 1.000 | 1.000 | 3184 | tags=33%, list=20%, signal=42% |
| 2466 | NIKOLSKY\_BREAST\_CANCER\_20Q11\_AMPLICON |  | 24 | 0.43 | 0.86 | 0.737 | 1.000 | 1.000 | 3805 | tags=33%, list=24%, signal=44% |
| 2467 | LE\_EGR2\_TARGETS\_DN |  | 102 | 0.39 | 0.86 | 0.830 | 1.000 | 1.000 | 3024 | tags=26%, list=19%, signal=33% |
| 2468 | REACTOME\_SIGNALLING\_TO\_RAS |  | 25 | 0.43 | 0.86 | 0.737 | 1.000 | 1.000 | 5230 | tags=60%, list=33%, signal=90% |
| 2469 | HOFFMANN\_IMMATURE\_TO\_MATURE\_B\_LYMPHOCYTE\_UP |  | 40 | 0.41 | 0.86 | 0.746 | 1.000 | 1.000 | 3297 | tags=23%, list=21%, signal=28% |
| 2470 | TAKEDA\_TARGETS\_OF\_NUP98\_HOXA9\_FUSION\_6HR\_DN |  | 37 | 0.41 | 0.86 | 0.761 | 1.000 | 1.000 | 2667 | tags=24%, list=17%, signal=29% |
| 2471 | PID\_KITPATHWAY |  | 51 | 0.41 | 0.86 | 0.783 | 1.000 | 1.000 | 5230 | tags=49%, list=33%, signal=73% |
| 2472 | VERRECCHIA\_RESPONSE\_TO\_TGFB1\_C2 |  | 24 | 0.43 | 0.86 | 0.738 | 1.000 | 1.000 | 2577 | tags=29%, list=16%, signal=35% |
| 2473 | FIGUEROA\_AML\_METHYLATION\_CLUSTER\_6\_UP |  | 116 | 0.39 | 0.86 | 0.865 | 1.000 | 1.000 | 4639 | tags=32%, list=29%, signal=45% |
| 2474 | BOSCO\_INTERFERON\_INDUCED\_ANTIVIRAL\_MODULE |  | 61 | 0.40 | 0.86 | 0.802 | 1.000 | 1.000 | 4040 | tags=25%, list=26%, signal=33% |
| 2475 | BIOCARTA\_ALK\_PATHWAY |  | 36 | 0.42 | 0.86 | 0.767 | 1.000 | 1.000 | 4435 | tags=42%, list=28%, signal=58% |
| 2476 | SMIRNOV\_RESPONSE\_TO\_IR\_6HR\_UP |  | 133 | 0.39 | 0.86 | 0.866 | 1.000 | 1.000 | 5531 | tags=44%, list=35%, signal=68% |
| 2477 | REACTOME\_ACTIVATED\_AMPK\_STIMULATES\_FATTY\_ACID\_OXIDATION\_IN\_MUSCLE |  | 16 | 0.46 | 0.86 | 0.727 | 1.000 | 1.000 | 2802 | tags=25%, list=18%, signal=30% |
| 2478 | BROWNE\_HCMV\_INFECTION\_8HR\_DN |  | 41 | 0.41 | 0.86 | 0.772 | 1.000 | 1.000 | 4080 | tags=37%, list=26%, signal=49% |
| 2479 | REACTOME\_BMAL1\_CLOCK\_NPAS2\_ACTIVATES\_CIRCADIAN\_EXPRESSION |  | 32 | 0.42 | 0.86 | 0.746 | 1.000 | 1.000 | 1615 | tags=16%, list=10%, signal=17% |
| 2480 | PID\_TAP63PATHWAY |  | 50 | 0.40 | 0.86 | 0.778 | 1.000 | 1.000 | 4281 | tags=30%, list=27%, signal=41% |
| 2481 | BROWNE\_HCMV\_INFECTION\_1HR\_DN |  | 201 | 0.38 | 0.86 | 0.919 | 1.000 | 1.000 | 4793 | tags=33%, list=30%, signal=47% |
| 2482 | REACTOME\_SIGNALING\_BY\_BMP |  | 22 | 0.43 | 0.86 | 0.730 | 1.000 | 1.000 | 3556 | tags=36%, list=23%, signal=47% |
| 2483 | YAUCH\_HEDGEHOG\_SIGNALING\_PARACRINE\_DN |  | 224 | 0.39 | 0.86 | 0.937 | 1.000 | 1.000 | 4616 | tags=17%, list=29%, signal=24% |
| 2484 | CREIGHTON\_ENDOCRINE\_THERAPY\_RESISTANCE\_5 |  | 391 | 0.38 | 0.86 | 0.968 | 1.000 | 1.000 | 3040 | tags=24%, list=19%, signal=28% |
| 2485 | KRASNOSELSKAYA\_ILF3\_TARGETS\_UP |  | 33 | 0.42 | 0.86 | 0.774 | 1.000 | 1.000 | 2622 | tags=21%, list=17%, signal=25% |
| 2486 | LEE\_METASTASIS\_AND\_ALTERNATIVE\_SPLICING\_UP |  | 71 | 0.40 | 0.86 | 0.824 | 1.000 | 1.000 | 2546 | tags=21%, list=16%, signal=25% |
| 2487 | REACTOME\_BOTULINUM\_NEUROTOXICITY |  | 16 | 0.46 | 0.86 | 0.735 | 1.000 | 1.000 | 5995 | tags=50%, list=38%, signal=81% |
| 2488 | HU\_ANGIOGENESIS\_UP |  | 20 | 0.44 | 0.86 | 0.744 | 1.000 | 1.000 | 4443 | tags=45%, list=28%, signal=63% |
| 2489 | LI\_PROSTATE\_CANCER\_EPIGENETIC |  | 29 | 0.43 | 0.86 | 0.752 | 1.000 | 1.000 | 2086 | tags=24%, list=13%, signal=28% |
| 2490 | WESTON\_VEGFA\_TARGETS\_12HR |  | 35 | 0.41 | 0.86 | 0.758 | 1.000 | 1.000 | 1655 | tags=20%, list=11%, signal=22% |
| 2491 | BIOCARTA\_PTDINS\_PATHWAY |  | 23 | 0.43 | 0.86 | 0.780 | 1.000 | 1.000 | 4113 | tags=43%, list=26%, signal=59% |
| 2492 | TAVAZOIE\_METASTASIS |  | 83 | 0.39 | 0.86 | 0.827 | 1.000 | 1.000 | 604 | tags=6%, list=4%, signal=6% |
| 2493 | GYORFFY\_DOXORUBICIN\_RESISTANCE |  | 40 | 0.41 | 0.86 | 0.775 | 1.000 | 1.000 | 3973 | tags=30%, list=25%, signal=40% |
| 2494 | BIOCARTA\_CARDIACEGF\_PATHWAY |  | 17 | 0.45 | 0.86 | 0.723 | 1.000 | 1.000 | 4466 | tags=35%, list=28%, signal=49% |
| 2495 | BANDRES\_RESPONSE\_TO\_CARMUSTIN\_WITHOUT\_MGMT\_48HR\_UP |  | 18 | 0.44 | 0.86 | 0.743 | 1.000 | 1.000 | 3691 | tags=33%, list=23%, signal=43% |
| 2496 | YAO\_TEMPORAL\_RESPONSE\_TO\_PROGESTERONE\_CLUSTER\_16 |  | 74 | 0.40 | 0.86 | 0.831 | 1.000 | 1.000 | 3206 | tags=26%, list=20%, signal=32% |
| 2497 | ST\_MYOCYTE\_AD\_PATHWAY |  | 26 | 0.42 | 0.86 | 0.747 | 1.000 | 1.000 | 4729 | tags=31%, list=30%, signal=44% |
| 2498 | IVANOVA\_HEMATOPOIESIS\_STEM\_CELL\_LONG\_TERM |  | 266 | 0.38 | 0.86 | 0.944 | 1.000 | 1.000 | 3583 | tags=23%, list=23%, signal=30% |
| 2499 | NIELSEN\_LIPOSARCOMA\_DN |  | 19 | 0.44 | 0.86 | 0.720 | 1.000 | 1.000 | 2529 | tags=32%, list=16%, signal=38% |
| 2500 | NIELSEN\_MALIGNAT\_FIBROUS\_HISTIOCYTOMA\_DN |  | 18 | 0.44 | 0.86 | 0.731 | 1.000 | 1.000 | 5827 | tags=56%, list=37%, signal=88% |
| 2501 | DOANE\_BREAST\_CANCER\_ESR1\_DN |  | 45 | 0.40 | 0.86 | 0.772 | 1.000 | 1.000 | 3659 | tags=18%, list=23%, signal=23% |
| 2502 | BIOCARTA\_MAL\_PATHWAY |  | 18 | 0.44 | 0.86 | 0.737 | 1.000 | 1.000 | 5333 | tags=56%, list=34%, signal=84% |
| 2503 | ABE\_INNER\_EAR |  | 44 | 0.41 | 0.85 | 0.789 | 1.000 | 1.000 | 3104 | tags=25%, list=20%, signal=31% |
| 2504 | TUOMISTO\_TUMOR\_SUPPRESSION\_BY\_COL13A1\_UP |  | 16 | 0.45 | 0.85 | 0.736 | 1.000 | 1.000 | 1395 | tags=19%, list=9%, signal=21% |
| 2505 | DIRMEIER\_LMP1\_RESPONSE\_EARLY |  | 63 | 0.40 | 0.85 | 0.820 | 1.000 | 1.000 | 3046 | tags=22%, list=19%, signal=27% |
| 2506 | MCCLUNG\_CREB1\_TARGETS\_UP |  | 91 | 0.39 | 0.85 | 0.834 | 1.000 | 1.000 | 3758 | tags=22%, list=24%, signal=29% |
| 2507 | XU\_CREBBP\_TARGETS\_DN |  | 41 | 0.41 | 0.85 | 0.776 | 1.000 | 1.000 | 2238 | tags=17%, list=14%, signal=20% |
| 2508 | KEGG\_FC\_GAMMA\_R\_MEDIATED\_PHAGOCYTOSIS |  | 88 | 0.39 | 0.85 | 0.848 | 1.000 | 1.000 | 4098 | tags=26%, list=26%, signal=35% |
| 2509 | BIOCARTA\_NFKB\_PATHWAY |  | 22 | 0.43 | 0.85 | 0.749 | 1.000 | 1.000 | 5331 | tags=50%, list=34%, signal=75% |
| 2510 | REACTOME\_NEGATIVE\_REGULATION\_OF\_FGFR\_SIGNALING |  | 36 | 0.41 | 0.85 | 0.758 | 1.000 | 1.000 | 2587 | tags=14%, list=16%, signal=17% |
| 2511 | BIOCARTA\_IL7\_PATHWAY |  | 17 | 0.44 | 0.85 | 0.730 | 1.000 | 1.000 | 5018 | tags=41%, list=32%, signal=60% |
| 2512 | DARWICHE\_SQUAMOUS\_CELL\_CARCINOMA\_DN |  | 148 | 0.38 | 0.85 | 0.908 | 1.000 | 1.000 | 2400 | tags=19%, list=15%, signal=22% |
| 2513 | MAINA\_VHL\_TARGETS\_DN |  | 16 | 0.45 | 0.85 | 0.731 | 1.000 | 1.000 | 4963 | tags=50%, list=32%, signal=73% |
| 2514 | TONKS\_TARGETS\_OF\_RUNX1\_RUNX1T1\_FUSION\_GRANULOCYTE\_UP |  | 47 | 0.40 | 0.85 | 0.788 | 1.000 | 1.000 | 2472 | tags=26%, list=16%, signal=30% |
| 2515 | RICKMAN\_HEAD\_AND\_NECK\_CANCER\_E |  | 69 | 0.39 | 0.85 | 0.815 | 1.000 | 1.000 | 1222 | tags=9%, list=8%, signal=9% |
| 2516 | MISSIAGLIA\_REGULATED\_BY\_METHYLATION\_UP |  | 107 | 0.39 | 0.85 | 0.856 | 1.000 | 1.000 | 2563 | tags=19%, list=16%, signal=22% |
| 2517 | BIOCARTA\_MYOSIN\_PATHWAY |  | 30 | 0.41 | 0.85 | 0.755 | 1.000 | 1.000 | 4482 | tags=40%, list=28%, signal=56% |
| 2518 | KAYO\_CALORIE\_RESTRICTION\_MUSCLE\_DN |  | 77 | 0.39 | 0.85 | 0.844 | 1.000 | 1.000 | 3679 | tags=30%, list=23%, signal=39% |
| 2519 | MAGRANGEAS\_MULTIPLE\_MYELOMA\_IGLL\_VS\_IGLK\_DN |  | 22 | 0.42 | 0.85 | 0.752 | 1.000 | 1.000 | 1061 | tags=14%, list=7%, signal=15% |
| 2520 | URS\_ADIPOCYTE\_DIFFERENTIATION\_UP |  | 64 | 0.39 | 0.85 | 0.823 | 1.000 | 1.000 | 2924 | tags=14%, list=19%, signal=17% |
| 2521 | KEGG\_LONG\_TERM\_DEPRESSION |  | 65 | 0.40 | 0.85 | 0.824 | 1.000 | 1.000 | 5031 | tags=34%, list=32%, signal=50% |
| 2522 | TOMIDA\_METASTASIS\_DN |  | 16 | 0.45 | 0.85 | 0.741 | 1.000 | 1.000 | 2573 | tags=50%, list=16%, signal=60% |
| 2523 | SHETH\_LIVER\_CANCER\_VS\_TXNIP\_LOSS\_PAM3 |  | 66 | 0.40 | 0.85 | 0.850 | 1.000 | 1.000 | 3300 | tags=30%, list=21%, signal=38% |
| 2524 | XU\_HGF\_TARGETS\_REPRESSED\_BY\_AKT1\_DN |  | 73 | 0.40 | 0.85 | 0.830 | 1.000 | 1.000 | 4304 | tags=23%, list=27%, signal=32% |
| 2525 | RODRIGUES\_DCC\_TARGETS\_DN |  | 112 | 0.38 | 0.85 | 0.863 | 1.000 | 1.000 | 4669 | tags=40%, list=30%, signal=57% |
| 2526 | MCBRYAN\_PUBERTAL\_BREAST\_4\_5WK\_DN |  | 171 | 0.38 | 0.85 | 0.921 | 1.000 | 1.000 | 3349 | tags=26%, list=21%, signal=32% |
| 2527 | OUYANG\_PROSTATE\_CANCER\_PROGRESSION\_UP |  | 18 | 0.44 | 0.85 | 0.737 | 1.000 | 1.000 | 5230 | tags=50%, list=33%, signal=75% |
| 2528 | DARWICHE\_PAPILLOMA\_RISK\_LOW\_DN |  | 131 | 0.38 | 0.85 | 0.884 | 1.000 | 1.000 | 1578 | tags=17%, list=10%, signal=19% |
| 2529 | PID\_SYNDECAN\_1\_PATHWAY |  | 46 | 0.40 | 0.85 | 0.808 | 1.000 | 1.000 | 2954 | tags=22%, list=19%, signal=27% |
| 2530 | REACTOME\_TRANSPORT\_TO\_THE\_GOLGI\_AND\_SUBSEQUENT\_MODIFICATION |  | 33 | 0.42 | 0.85 | 0.789 | 1.000 | 1.000 | 4596 | tags=42%, list=29%, signal=60% |
| 2531 | MARTENS\_BOUND\_BY\_PML\_RARA\_FUSION |  | 392 | 0.38 | 0.85 | 0.984 | 1.000 | 1.000 | 4233 | tags=27%, list=27%, signal=36% |
| 2532 | ZHU\_CMV\_ALL\_DN |  | 124 | 0.38 | 0.85 | 0.884 | 1.000 | 1.000 | 3389 | tags=30%, list=22%, signal=38% |
| 2533 | LIU\_TARGETS\_OF\_VMYB\_VS\_CMYB\_DN |  | 39 | 0.41 | 0.85 | 0.805 | 1.000 | 1.000 | 4333 | tags=26%, list=28%, signal=35% |
| 2534 | STEIN\_ESRRA\_TARGETS\_UP |  | 334 | 0.38 | 0.85 | 0.970 | 1.000 | 1.000 | 4148 | tags=35%, list=26%, signal=47% |
| 2535 | IWANAGA\_CARCINOGENESIS\_BY\_KRAS\_PTEN\_DN |  | 305 | 0.38 | 0.85 | 0.972 | 1.000 | 1.000 | 4594 | tags=32%, list=29%, signal=44% |
| 2536 | REACTOME\_SLC\_MEDIATED\_TRANSMEMBRANE\_TRANSPORT |  | 236 | 0.38 | 0.84 | 0.951 | 1.000 | 1.000 | 3343 | tags=17%, list=21%, signal=21% |
| 2537 | GAVIN\_FOXP3\_TARGETS\_CLUSTER\_P2 |  | 67 | 0.39 | 0.84 | 0.826 | 1.000 | 1.000 | 3029 | tags=19%, list=19%, signal=24% |
| 2538 | KEGG\_GNRH\_SIGNALING\_PATHWAY |  | 95 | 0.38 | 0.84 | 0.862 | 1.000 | 1.000 | 5031 | tags=39%, list=32%, signal=57% |
| 2539 | NELSON\_RESPONSE\_TO\_ANDROGEN\_DN |  | 18 | 0.44 | 0.84 | 0.745 | 1.000 | 1.000 | 3927 | tags=33%, list=25%, signal=44% |
| 2540 | HAN\_JNK\_SINGALING\_UP |  | 32 | 0.41 | 0.84 | 0.776 | 1.000 | 1.000 | 4312 | tags=44%, list=27%, signal=60% |
| 2541 | PID\_WNT\_NONCANONICAL\_PATHWAY |  | 30 | 0.41 | 0.84 | 0.774 | 1.000 | 1.000 | 4803 | tags=43%, list=30%, signal=62% |
| 2542 | MCBRYAN\_PUBERTAL\_BREAST\_3\_4WK\_DN |  | 28 | 0.42 | 0.84 | 0.776 | 1.000 | 1.000 | 2165 | tags=25%, list=14%, signal=29% |
| 2543 | CROONQUIST\_IL6\_DEPRIVATION\_UP |  | 18 | 0.44 | 0.84 | 0.765 | 1.000 | 1.000 | 3036 | tags=22%, list=19%, signal=27% |
| 2544 | BOYAULT\_LIVER\_CANCER\_SUBCLASS\_G123\_DN |  | 48 | 0.39 | 0.84 | 0.796 | 1.000 | 1.000 | 1730 | tags=15%, list=11%, signal=16% |
| 2545 | KEGG\_ADHERENS\_JUNCTION |  | 73 | 0.39 | 0.84 | 0.840 | 1.000 | 1.000 | 4060 | tags=37%, list=26%, signal=50% |
| 2546 | BIOCARTA\_ERK5\_PATHWAY |  | 17 | 0.43 | 0.84 | 0.726 | 1.000 | 1.000 | 5230 | tags=59%, list=33%, signal=88% |
| 2547 | PID\_INTEGRIN2\_PATHWAY |  | 28 | 0.41 | 0.84 | 0.767 | 1.000 | 1.000 | 1110 | tags=11%, list=7%, signal=12% |
| 2548 | NELSON\_RESPONSE\_TO\_ANDROGEN\_UP |  | 81 | 0.39 | 0.84 | 0.851 | 1.000 | 1.000 | 3206 | tags=27%, list=20%, signal=34% |
| 2549 | KEGG\_PRION\_DISEASES |  | 33 | 0.41 | 0.84 | 0.784 | 1.000 | 1.000 | 2684 | tags=24%, list=17%, signal=29% |
| 2550 | RODRIGUES\_THYROID\_CARCINOMA\_ANAPLASTIC\_DN |  | 430 | 0.37 | 0.84 | 0.991 | 1.000 | 1.000 | 4738 | tags=34%, list=30%, signal=47% |
| 2551 | RODWELL\_AGING\_KIDNEY\_UP |  | 390 | 0.37 | 0.84 | 0.981 | 1.000 | 1.000 | 3643 | tags=24%, list=23%, signal=31% |
| 2552 | REACTOME\_G\_PROTEIN\_ACTIVATION |  | 27 | 0.42 | 0.84 | 0.777 | 1.000 | 1.000 | 5784 | tags=30%, list=37%, signal=47% |
| 2553 | KEGG\_LINOLEIC\_ACID\_METABOLISM |  | 27 | 0.41 | 0.84 | 0.770 | 1.000 | 1.000 | 703 | tags=7%, list=4%, signal=8% |
| 2554 | NIELSEN\_GIST\_AND\_SYNOVIAL\_SARCOMA\_DN |  | 19 | 0.43 | 0.84 | 0.759 | 1.000 | 1.000 | 3277 | tags=21%, list=21%, signal=27% |
| 2555 | GRAHAM\_CML\_QUIESCENT\_VS\_NORMAL\_QUIESCENT\_DN |  | 38 | 0.40 | 0.84 | 0.799 | 1.000 | 1.000 | 1487 | tags=16%, list=9%, signal=17% |
| 2556 | REACTOME\_EXTRACELLULAR\_MATRIX\_ORGANIZATION |  | 84 | 0.39 | 0.84 | 0.868 | 1.000 | 1.000 | 2708 | tags=14%, list=17%, signal=17% |
| 2557 | ACEVEDO\_LIVER\_TUMOR\_VS\_NORMAL\_ADJACENT\_TISSUE\_DN |  | 225 | 0.38 | 0.84 | 0.941 | 1.000 | 1.000 | 3471 | tags=24%, list=22%, signal=30% |
| 2558 | BIOCARTA\_IL6\_PATHWAY |  | 22 | 0.43 | 0.84 | 0.753 | 1.000 | 1.000 | 5230 | tags=55%, list=33%, signal=82% |
| 2559 | IIZUKA\_LIVER\_CANCER\_PROGRESSION\_G2\_G3\_UP |  | 25 | 0.41 | 0.84 | 0.761 | 1.000 | 1.000 | 3258 | tags=24%, list=21%, signal=30% |
| 2560 | PID\_HIF1\_TFPATHWAY |  | 61 | 0.39 | 0.84 | 0.847 | 1.000 | 1.000 | 4747 | tags=43%, list=30%, signal=61% |
| 2561 | MULLIGHAN\_MLL\_SIGNATURE\_2\_UP |  | 356 | 0.37 | 0.84 | 0.979 | 1.000 | 1.000 | 2781 | tags=20%, list=18%, signal=24% |
| 2562 | NIKOLSKY\_MUTATED\_AND\_AMPLIFIED\_IN\_BREAST\_CANCER |  | 74 | 0.39 | 0.84 | 0.854 | 1.000 | 1.000 | 4906 | tags=31%, list=31%, signal=45% |
| 2563 | HAHTOLA\_MYCOSIS\_FUNGOIDES\_UP |  | 15 | 0.44 | 0.84 | 0.757 | 1.000 | 1.000 | 3395 | tags=27%, list=22%, signal=34% |
| 2564 | HANSON\_HRAS\_SIGNALING\_VIA\_NFKB |  | 20 | 0.43 | 0.84 | 0.768 | 1.000 | 1.000 | 1706 | tags=15%, list=11%, signal=17% |
| 2565 | REACTOME\_GPVI\_MEDIATED\_ACTIVATION\_CASCADE |  | 30 | 0.41 | 0.84 | 0.787 | 1.000 | 1.000 | 3450 | tags=27%, list=22%, signal=34% |
| 2566 | DELACROIX\_RAR\_TARGETS\_DN |  | 23 | 0.42 | 0.83 | 0.767 | 1.000 | 1.000 | 4407 | tags=39%, list=28%, signal=54% |
| 2567 | HUANG\_GATA2\_TARGETS\_UP |  | 134 | 0.38 | 0.83 | 0.915 | 1.000 | 1.000 | 2792 | tags=19%, list=18%, signal=23% |
| 2568 | DELLA\_RESPONSE\_TO\_TSA\_AND\_BUTYRATE |  | 21 | 0.42 | 0.83 | 0.742 | 1.000 | 1.000 | 2165 | tags=29%, list=14%, signal=33% |
| 2569 | CHENG\_IMPRINTED\_BY\_ESTRADIOL |  | 93 | 0.38 | 0.83 | 0.877 | 1.000 | 1.000 | 4999 | tags=34%, list=32%, signal=50% |
| 2570 | RIZ\_ERYTHROID\_DIFFERENTIATION\_12HR |  | 43 | 0.40 | 0.83 | 0.827 | 1.000 | 1.000 | 1985 | tags=14%, list=13%, signal=16% |
| 2571 | ZHOU\_INFLAMMATORY\_RESPONSE\_FIMA\_DN |  | 215 | 0.37 | 0.83 | 0.960 | 1.000 | 1.000 | 5000 | tags=33%, list=32%, signal=48% |
| 2572 | VART\_KSHV\_INFECTION\_ANGIOGENIC\_MARKERS\_UP |  | 157 | 0.37 | 0.83 | 0.928 | 1.000 | 1.000 | 3025 | tags=17%, list=19%, signal=20% |
| 2573 | HUPER\_BREAST\_BASAL\_VS\_LUMINAL\_UP |  | 48 | 0.39 | 0.83 | 0.819 | 1.000 | 1.000 | 1611 | tags=13%, list=10%, signal=14% |
| 2574 | KEEN\_RESPONSE\_TO\_ROSIGLITAZONE\_UP |  | 32 | 0.41 | 0.83 | 0.793 | 1.000 | 1.000 | 2305 | tags=22%, list=15%, signal=26% |
| 2575 | KAMIKUBO\_MYELOID\_CEBPA\_NETWORK |  | 27 | 0.41 | 0.83 | 0.788 | 1.000 | 1.000 | 697 | tags=11%, list=4%, signal=12% |
| 2576 | ZEMBUTSU\_SENSITIVITY\_TO\_MITOMYCIN |  | 16 | 0.44 | 0.83 | 0.784 | 1.000 | 1.000 | 4508 | tags=38%, list=29%, signal=52% |
| 2577 | CAMPS\_COLON\_CANCER\_COPY\_NUMBER\_DN |  | 39 | 0.40 | 0.83 | 0.797 | 1.000 | 1.000 | 2213 | tags=13%, list=14%, signal=15% |
| 2578 | MOOTHA\_FFA\_OXYDATION |  | 20 | 0.42 | 0.83 | 0.759 | 1.000 | 1.000 | 4298 | tags=55%, list=27%, signal=76% |
| 2579 | MANTOVANI\_NFKB\_TARGETS\_UP |  | 42 | 0.40 | 0.83 | 0.818 | 1.000 | 1.000 | 946 | tags=10%, list=6%, signal=10% |
| 2580 | VERRECCHIA\_EARLY\_RESPONSE\_TO\_TGFB1 |  | 57 | 0.39 | 0.83 | 0.837 | 1.000 | 1.000 | 964 | tags=16%, list=6%, signal=17% |
| 2581 | MONNIER\_POSTRADIATION\_TUMOR\_ESCAPE\_DN |  | 310 | 0.37 | 0.83 | 0.976 | 1.000 | 1.000 | 5064 | tags=44%, list=32%, signal=63% |
| 2582 | WATANABE\_COLON\_CANCER\_MSI\_VS\_MSS\_DN |  | 66 | 0.38 | 0.83 | 0.856 | 1.000 | 1.000 | 4264 | tags=29%, list=27%, signal=39% |
| 2583 | BERTUCCI\_MEDULLARY\_VS\_DUCTAL\_BREAST\_CANCER\_DN |  | 138 | 0.37 | 0.83 | 0.907 | 1.000 | 1.000 | 2125 | tags=17%, list=13%, signal=20% |
| 2584 | LEE\_LIVER\_CANCER\_E2F1\_DN |  | 59 | 0.39 | 0.83 | 0.851 | 1.000 | 1.000 | 3345 | tags=24%, list=21%, signal=30% |
| 2585 | REACTOME\_IL\_3\_5\_AND\_GM\_CSF\_SIGNALING |  | 43 | 0.39 | 0.83 | 0.812 | 1.000 | 1.000 | 4031 | tags=30%, list=26%, signal=41% |
| 2586 | REACTOME\_INTEGRATION\_OF\_ENERGY\_METABOLISM |  | 114 | 0.38 | 0.83 | 0.911 | 1.000 | 1.000 | 4594 | tags=27%, list=29%, signal=38% |
| 2587 | NIELSEN\_GIST |  | 82 | 0.38 | 0.83 | 0.872 | 1.000 | 1.000 | 4208 | tags=24%, list=27%, signal=33% |
| 2588 | MUELLER\_COMMON\_TARGETS\_OF\_AML\_FUSIONS\_DN |  | 26 | 0.41 | 0.83 | 0.780 | 1.000 | 1.000 | 3950 | tags=31%, list=25%, signal=41% |
| 2589 | GOZGIT\_ESR1\_TARGETS\_UP |  | 119 | 0.38 | 0.83 | 0.925 | 1.000 | 1.000 | 2996 | tags=20%, list=19%, signal=25% |
| 2590 | ZHOU\_INFLAMMATORY\_RESPONSE\_LIVE\_DN |  | 292 | 0.37 | 0.83 | 0.974 | 1.000 | 1.000 | 5037 | tags=38%, list=32%, signal=54% |
| 2591 | BHAT\_ESR1\_TARGETS\_NOT\_VIA\_AKT1\_UP |  | 188 | 0.37 | 0.83 | 0.950 | 1.000 | 1.000 | 2910 | tags=20%, list=18%, signal=24% |
| 2592 | RASHI\_RESPONSE\_TO\_IONIZING\_RADIATION\_6 |  | 77 | 0.38 | 0.83 | 0.881 | 1.000 | 1.000 | 3600 | tags=19%, list=23%, signal=25% |
| 2593 | ST\_T\_CELL\_SIGNAL\_TRANSDUCTION |  | 43 | 0.40 | 0.83 | 0.812 | 1.000 | 1.000 | 6079 | tags=44%, list=39%, signal=72% |
| 2594 | PID\_IL12\_STAT4PATHWAY |  | 32 | 0.40 | 0.83 | 0.795 | 1.000 | 1.000 | 812 | tags=9%, list=5%, signal=10% |
| 2595 | GAUSSMANN\_MLL\_AF4\_FUSION\_TARGETS\_D\_UP |  | 34 | 0.40 | 0.83 | 0.784 | 1.000 | 1.000 | 2009 | tags=9%, list=13%, signal=10% |
| 2596 | ENGELMANN\_CANCER\_PROGENITORS\_UP |  | 44 | 0.39 | 0.83 | 0.820 | 1.000 | 1.000 | 2979 | tags=27%, list=19%, signal=34% |
| 2597 | SARRIO\_EPITHELIAL\_MESENCHYMAL\_TRANSITION\_DN |  | 139 | 0.37 | 0.82 | 0.926 | 1.000 | 1.000 | 3025 | tags=23%, list=19%, signal=28% |
| 2598 | HOQUE\_METHYLATED\_IN\_CANCER |  | 55 | 0.39 | 0.82 | 0.846 | 1.000 | 1.000 | 3284 | tags=22%, list=21%, signal=27% |
| 2599 | MCCABE\_HOXC6\_TARGETS\_CANCER\_UP |  | 25 | 0.41 | 0.82 | 0.791 | 1.000 | 1.000 | 4705 | tags=36%, list=30%, signal=51% |
| 2600 | REACTOME\_NCAM\_SIGNALING\_FOR\_NEURITE\_OUT\_GROWTH |  | 62 | 0.38 | 0.82 | 0.835 | 1.000 | 1.000 | 3328 | tags=18%, list=21%, signal=22% |
| 2601 | PID\_HDAC\_CLASSIII\_PATHWAY |  | 24 | 0.41 | 0.82 | 0.786 | 1.000 | 1.000 | 2811 | tags=25%, list=18%, signal=30% |
| 2602 | GERHOLD\_ADIPOGENESIS\_UP |  | 47 | 0.39 | 0.82 | 0.823 | 1.000 | 1.000 | 1223 | tags=17%, list=8%, signal=18% |
| 2603 | WHITE\_NEUROBLASTOMA\_WITH\_1P36.3\_DELETION |  | 20 | 0.42 | 0.82 | 0.788 | 1.000 | 1.000 | 4889 | tags=35%, list=31%, signal=51% |
| 2604 | COATES\_MACROPHAGE\_M1\_VS\_M2\_DN |  | 63 | 0.38 | 0.82 | 0.861 | 1.000 | 1.000 | 3258 | tags=22%, list=21%, signal=28% |
| 2605 | BOYLAN\_MULTIPLE\_MYELOMA\_PCA3\_UP |  | 65 | 0.38 | 0.82 | 0.882 | 1.000 | 1.000 | 3079 | tags=18%, list=20%, signal=23% |
| 2606 | REACTOME\_NOD1\_2\_SIGNALING\_PATHWAY |  | 26 | 0.41 | 0.82 | 0.809 | 1.000 | 1.000 | 4953 | tags=50%, list=31%, signal=73% |
| 2607 | VALK\_AML\_WITH\_EVI1 |  | 20 | 0.42 | 0.82 | 0.804 | 1.000 | 1.000 | 1342 | tags=15%, list=9%, signal=16% |
| 2608 | BRACHAT\_RESPONSE\_TO\_CISPLATIN |  | 20 | 0.42 | 0.82 | 0.785 | 1.000 | 1.000 | 562 | tags=15%, list=4%, signal=16% |
| 2609 | BROWNE\_HCMV\_INFECTION\_48HR\_DN |  | 449 | 0.36 | 0.82 | 0.994 | 1.000 | 1.000 | 3956 | tags=24%, list=25%, signal=32% |
| 2610 | FONTAINE\_FOLLICULAR\_THYROID\_ADENOMA\_UP |  | 61 | 0.38 | 0.82 | 0.851 | 1.000 | 1.000 | 3619 | tags=31%, list=23%, signal=40% |
| 2611 | ZWANG\_CLASS\_3\_TRANSIENTLY\_INDUCED\_BY\_EGF |  | 189 | 0.37 | 0.82 | 0.958 | 1.000 | 1.000 | 4223 | tags=26%, list=27%, signal=35% |
| 2612 | SHEDDEN\_LUNG\_CANCER\_GOOD\_SURVIVAL\_A12 |  | 242 | 0.37 | 0.82 | 0.967 | 1.000 | 1.000 | 3417 | tags=17%, list=22%, signal=21% |
| 2613 | LINDSTEDT\_DENDRITIC\_CELL\_MATURATION\_D |  | 64 | 0.38 | 0.82 | 0.880 | 1.000 | 1.000 | 3247 | tags=28%, list=21%, signal=35% |
| 2614 | FIGUEROA\_AML\_METHYLATION\_CLUSTER\_5\_DN |  | 42 | 0.39 | 0.82 | 0.810 | 1.000 | 1.000 | 4378 | tags=21%, list=28%, signal=30% |
| 2615 | YAO\_TEMPORAL\_RESPONSE\_TO\_PROGESTERONE\_CLUSTER\_9 |  | 64 | 0.38 | 0.82 | 0.857 | 1.000 | 1.000 | 3956 | tags=42%, list=25%, signal=56% |
| 2616 | LIM\_MAMMARY\_STEM\_CELL\_UP |  | 446 | 0.36 | 0.82 | 0.995 | 1.000 | 1.000 | 3216 | tags=20%, list=20%, signal=24% |
| 2617 | BECKER\_TAMOXIFEN\_RESISTANCE\_UP |  | 46 | 0.39 | 0.82 | 0.837 | 1.000 | 1.000 | 3005 | tags=30%, list=19%, signal=38% |
| 2618 | BIOCARTA\_EPO\_PATHWAY |  | 19 | 0.42 | 0.82 | 0.788 | 1.000 | 1.000 | 5230 | tags=53%, list=33%, signal=79% |
| 2619 | CHIARETTI\_T\_ALL\_REFRACTORY\_TO\_THERAPY |  | 27 | 0.41 | 0.82 | 0.805 | 1.000 | 1.000 | 1201 | tags=11%, list=8%, signal=12% |
| 2620 | REACTOME\_ACYL\_CHAIN\_REMODELLING\_OF\_PC |  | 16 | 0.43 | 0.81 | 0.779 | 1.000 | 1.000 | 703 | tags=13%, list=4%, signal=13% |
| 2621 | LEE\_LIVER\_CANCER\_MYC\_TGFA\_UP |  | 59 | 0.38 | 0.81 | 0.851 | 1.000 | 1.000 | 2975 | tags=31%, list=19%, signal=37% |
| 2622 | PID\_CXCR4\_PATHWAY |  | 98 | 0.37 | 0.81 | 0.903 | 1.000 | 1.000 | 4185 | tags=29%, list=27%, signal=39% |
| 2623 | BIOCARTA\_PDGF\_PATHWAY |  | 31 | 0.40 | 0.81 | 0.823 | 1.000 | 1.000 | 5230 | tags=55%, list=33%, signal=82% |
| 2624 | LABBE\_TARGETS\_OF\_TGFB1\_AND\_WNT3A\_DN |  | 103 | 0.37 | 0.81 | 0.917 | 1.000 | 1.000 | 1722 | tags=17%, list=11%, signal=19% |
| 2625 | ZEMBUTSU\_SENSITIVITY\_TO\_VINBLASTINE |  | 17 | 0.42 | 0.81 | 0.772 | 1.000 | 1.000 | 1959 | tags=29%, list=12%, signal=34% |
| 2626 | BIOCARTA\_ACH\_PATHWAY |  | 16 | 0.43 | 0.81 | 0.757 | 1.000 | 1.000 | 5440 | tags=38%, list=35%, signal=57% |
| 2627 | COATES\_MACROPHAGE\_M1\_VS\_M2\_UP |  | 73 | 0.38 | 0.81 | 0.896 | 1.000 | 1.000 | 2031 | tags=16%, list=13%, signal=19% |
| 2628 | BREDEMEYER\_RAG\_SIGNALING\_NOT\_VIA\_ATM\_DN |  | 50 | 0.38 | 0.81 | 0.838 | 1.000 | 1.000 | 3556 | tags=24%, list=23%, signal=31% |
| 2629 | KIM\_LRRC3B\_TARGETS |  | 23 | 0.41 | 0.81 | 0.778 | 1.000 | 1.000 | 2871 | tags=35%, list=18%, signal=42% |
| 2630 | TOMLINS\_METASTASIS\_DN |  | 18 | 0.42 | 0.81 | 0.798 | 1.000 | 1.000 | 2277 | tags=22%, list=14%, signal=26% |
| 2631 | DELACROIX\_RAR\_BOUND\_ES |  | 391 | 0.36 | 0.81 | 0.991 | 1.000 | 1.000 | 3914 | tags=27%, list=25%, signal=35% |
| 2632 | RASHI\_NFKB1\_TARGETS |  | 17 | 0.42 | 0.81 | 0.779 | 1.000 | 1.000 | 2051 | tags=24%, list=13%, signal=27% |
| 2633 | LEE\_LIVER\_CANCER\_ACOX1\_UP |  | 57 | 0.38 | 0.81 | 0.857 | 1.000 | 1.000 | 2953 | tags=25%, list=19%, signal=30% |
| 2634 | KIM\_WT1\_TARGETS\_12HR\_UP |  | 139 | 0.37 | 0.81 | 0.938 | 1.000 | 1.000 | 2869 | tags=22%, list=18%, signal=27% |
| 2635 | REACTOME\_TAK1\_ACTIVATES\_NFKB\_BY\_PHOSPHORYLATION\_AND\_ACTIVATION\_OF\_IKKS\_COMPLEX |  | 19 | 0.42 | 0.81 | 0.812 | 1.000 | 1.000 | 2068 | tags=21%, list=13%, signal=24% |
| 2636 | MCBRYAN\_PUBERTAL\_BREAST\_6\_7WK\_DN |  | 72 | 0.37 | 0.81 | 0.867 | 1.000 | 1.000 | 2953 | tags=29%, list=19%, signal=36% |
| 2637 | LEIN\_CEREBELLUM\_MARKERS |  | 76 | 0.37 | 0.81 | 0.893 | 1.000 | 1.000 | 2233 | tags=11%, list=14%, signal=12% |
| 2638 | JEON\_SMAD6\_TARGETS\_UP |  | 23 | 0.41 | 0.81 | 0.811 | 1.000 | 1.000 | 3185 | tags=26%, list=20%, signal=33% |
| 2639 | PID\_EPHA\_FWDPATHWAY |  | 33 | 0.39 | 0.81 | 0.832 | 1.000 | 1.000 | 4031 | tags=24%, list=26%, signal=33% |
| 2640 | REACTOME\_ABCA\_TRANSPORTERS\_IN\_LIPID\_HOMEOSTASIS |  | 16 | 0.43 | 0.81 | 0.781 | 1.000 | 1.000 | 1767 | tags=13%, list=11%, signal=14% |
| 2641 | LEIN\_MIDBRAIN\_MARKERS |  | 71 | 0.37 | 0.81 | 0.878 | 1.000 | 1.000 | 4183 | tags=23%, list=27%, signal=31% |
| 2642 | BURTON\_ADIPOGENESIS\_PEAK\_AT\_0HR |  | 58 | 0.37 | 0.81 | 0.860 | 1.000 | 1.000 | 2909 | tags=29%, list=18%, signal=36% |
| 2643 | PID\_IL8CXCR2\_PATHWAY |  | 29 | 0.40 | 0.81 | 0.813 | 1.000 | 1.000 | 4124 | tags=34%, list=26%, signal=47% |
| 2644 | ABRAHAM\_ALPC\_VS\_MULTIPLE\_MYELOMA\_UP |  | 26 | 0.40 | 0.81 | 0.822 | 1.000 | 1.000 | 3406 | tags=27%, list=22%, signal=34% |
| 2645 | KEGG\_ALPHA\_LINOLENIC\_ACID\_METABOLISM |  | 17 | 0.42 | 0.81 | 0.785 | 1.000 | 1.000 | 703 | tags=12%, list=4%, signal=12% |
| 2646 | SEMENZA\_HIF1\_TARGETS |  | 35 | 0.39 | 0.81 | 0.838 | 1.000 | 1.000 | 4656 | tags=43%, list=30%, signal=61% |
| 2647 | REACTOME\_INTERFERON\_ALPHA\_BETA\_SIGNALING |  | 47 | 0.38 | 0.81 | 0.847 | 1.000 | 1.000 | 5394 | tags=34%, list=34%, signal=52% |
| 2648 | KEGG\_SELENOAMINO\_ACID\_METABOLISM |  | 22 | 0.41 | 0.81 | 0.807 | 1.000 | 1.000 | 3799 | tags=45%, list=24%, signal=60% |
| 2649 | BIOCARTA\_HER2\_PATHWAY |  | 22 | 0.41 | 0.81 | 0.806 | 1.000 | 1.000 | 5230 | tags=59%, list=33%, signal=88% |
| 2650 | KEGG\_ARRHYTHMOGENIC\_RIGHT\_VENTRICULAR\_CARDIOMYOPATHY\_ARVC |  | 74 | 0.37 | 0.80 | 0.881 | 1.000 | 1.000 | 3206 | tags=16%, list=20%, signal=20% |
| 2651 | ELVIDGE\_HIF1A\_TARGETS\_DN |  | 83 | 0.37 | 0.80 | 0.890 | 1.000 | 1.000 | 2922 | tags=23%, list=19%, signal=28% |
| 2652 | REACTOME\_SYNTHESIS\_OF\_PIPS\_AT\_THE\_PLASMA\_MEMBRANE |  | 24 | 0.40 | 0.80 | 0.822 | 1.000 | 1.000 | 6055 | tags=58%, list=38%, signal=95% |
| 2653 | YEGNASUBRAMANIAN\_PROSTATE\_CANCER |  | 104 | 0.37 | 0.80 | 0.926 | 1.000 | 1.000 | 3752 | tags=23%, list=24%, signal=30% |
| 2654 | PAL\_PRMT5\_TARGETS\_DN |  | 28 | 0.40 | 0.80 | 0.824 | 1.000 | 1.000 | 3654 | tags=25%, list=23%, signal=32% |
| 2655 | BIOCARTA\_RELA\_PATHWAY |  | 16 | 0.42 | 0.80 | 0.790 | 1.000 | 1.000 | 5066 | tags=50%, list=32%, signal=74% |
| 2656 | FIGUEROA\_AML\_METHYLATION\_CLUSTER\_3\_DN |  | 30 | 0.39 | 0.80 | 0.824 | 1.000 | 1.000 | 1825 | tags=17%, list=12%, signal=19% |
| 2657 | QI\_PLASMACYTOMA\_UP |  | 247 | 0.36 | 0.80 | 0.983 | 1.000 | 1.000 | 1897 | tags=12%, list=12%, signal=13% |
| 2658 | LA\_MEN1\_TARGETS |  | 24 | 0.40 | 0.80 | 0.804 | 1.000 | 1.000 | 3507 | tags=29%, list=22%, signal=37% |
| 2659 | CASTELLANO\_NRAS\_TARGETS\_UP |  | 62 | 0.37 | 0.80 | 0.866 | 1.000 | 1.000 | 3649 | tags=29%, list=23%, signal=38% |
| 2660 | DUAN\_PRDM5\_TARGETS |  | 67 | 0.37 | 0.80 | 0.871 | 1.000 | 1.000 | 4223 | tags=25%, list=27%, signal=35% |
| 2661 | CROMER\_TUMORIGENESIS\_UP |  | 54 | 0.38 | 0.80 | 0.874 | 1.000 | 1.000 | 3504 | tags=19%, list=22%, signal=24% |
| 2662 | DAZARD\_UV\_RESPONSE\_CLUSTER\_G24 |  | 26 | 0.40 | 0.80 | 0.835 | 1.000 | 1.000 | 1353 | tags=12%, list=9%, signal=13% |
| 2663 | RUIZ\_TNC\_TARGETS\_UP |  | 136 | 0.36 | 0.80 | 0.941 | 1.000 | 1.000 | 3241 | tags=24%, list=21%, signal=30% |
| 2664 | REACTOME\_ACYL\_CHAIN\_REMODELLING\_OF\_PE |  | 17 | 0.42 | 0.80 | 0.794 | 1.000 | 1.000 | 703 | tags=12%, list=4%, signal=12% |
| 2665 | BANDRES\_RESPONSE\_TO\_CARMUSTIN\_MGMT\_24HR\_DN |  | 31 | 0.39 | 0.80 | 0.822 | 1.000 | 1.000 | 3206 | tags=19%, list=20%, signal=24% |
| 2666 | ZHOU\_INFLAMMATORY\_RESPONSE\_LIVE\_UP |  | 382 | 0.36 | 0.80 | 0.994 | 1.000 | 1.000 | 3322 | tags=18%, list=21%, signal=22% |
| 2667 | GAVIN\_FOXP3\_TARGETS\_CLUSTER\_P7 |  | 79 | 0.37 | 0.80 | 0.894 | 1.000 | 1.000 | 4110 | tags=25%, list=26%, signal=34% |
| 2668 | REACTOME\_RORA\_ACTIVATES\_CIRCADIAN\_EXPRESSION |  | 23 | 0.40 | 0.80 | 0.815 | 1.000 | 1.000 | 5480 | tags=48%, list=35%, signal=73% |
| 2669 | KARLSSON\_TGFB1\_TARGETS\_DN |  | 187 | 0.36 | 0.80 | 0.964 | 1.000 | 1.000 | 4424 | tags=35%, list=28%, signal=48% |
| 2670 | SENESE\_HDAC2\_TARGETS\_DN |  | 109 | 0.36 | 0.80 | 0.933 | 1.000 | 1.000 | 1544 | tags=15%, list=10%, signal=16% |
| 2671 | BIOCARTA\_TNFR2\_PATHWAY |  | 18 | 0.42 | 0.80 | 0.794 | 1.000 | 1.000 | 3295 | tags=33%, list=21%, signal=42% |
| 2672 | PID\_FGF\_PATHWAY |  | 55 | 0.37 | 0.80 | 0.872 | 1.000 | 1.000 | 4098 | tags=27%, list=26%, signal=37% |
| 2673 | PID\_RAS\_PATHWAY |  | 28 | 0.39 | 0.80 | 0.829 | 1.000 | 1.000 | 1169 | tags=14%, list=7%, signal=15% |
| 2674 | KEGG\_AMINO\_SUGAR\_AND\_NUCLEOTIDE\_SUGAR\_METABOLISM |  | 44 | 0.38 | 0.80 | 0.852 | 1.000 | 1.000 | 3148 | tags=27%, list=20%, signal=34% |
| 2675 | PID\_IL4\_2PATHWAY |  | 62 | 0.37 | 0.80 | 0.890 | 1.000 | 1.000 | 4417 | tags=29%, list=28%, signal=40% |
| 2676 | BRUINS\_UVC\_RESPONSE\_VIA\_TP53\_GROUP\_C |  | 72 | 0.37 | 0.80 | 0.895 | 1.000 | 1.000 | 3527 | tags=19%, list=22%, signal=25% |
| 2677 | GILDEA\_METASTASIS |  | 27 | 0.39 | 0.80 | 0.812 | 1.000 | 1.000 | 3005 | tags=37%, list=19%, signal=46% |
| 2678 | KEGG\_N\_GLYCAN\_BIOSYNTHESIS |  | 44 | 0.38 | 0.80 | 0.858 | 1.000 | 1.000 | 4615 | tags=41%, list=29%, signal=58% |
| 2679 | REACTOME\_SEMA4D\_IN\_SEMAPHORIN\_SIGNALING |  | 27 | 0.39 | 0.80 | 0.820 | 1.000 | 1.000 | 1682 | tags=22%, list=11%, signal=25% |
| 2680 | PEDERSEN\_METASTASIS\_BY\_ERBB2\_ISOFORM\_4 |  | 94 | 0.36 | 0.80 | 0.922 | 1.000 | 1.000 | 2953 | tags=23%, list=19%, signal=29% |
| 2681 | KOYAMA\_SEMA3B\_TARGETS\_UP |  | 231 | 0.36 | 0.80 | 0.975 | 1.000 | 1.000 | 3123 | tags=19%, list=20%, signal=24% |
| 2682 | MOROSETTI\_FACIOSCAPULOHUMERAL\_MUSCULAR\_DISTROPHY\_UP |  | 20 | 0.41 | 0.80 | 0.803 | 1.000 | 1.000 | 2622 | tags=25%, list=17%, signal=30% |
| 2683 | REACTOME\_METABOLISM\_OF\_VITAMINS\_AND\_COFACTORS |  | 49 | 0.37 | 0.80 | 0.873 | 1.000 | 1.000 | 3481 | tags=29%, list=22%, signal=37% |
| 2684 | ONDER\_CDH1\_TARGETS\_2\_UP |  | 227 | 0.36 | 0.79 | 0.979 | 1.000 | 1.000 | 3484 | tags=23%, list=22%, signal=29% |
| 2685 | AMIT\_SERUM\_RESPONSE\_120\_MCF10A |  | 56 | 0.37 | 0.79 | 0.873 | 1.000 | 1.000 | 5475 | tags=39%, list=35%, signal=60% |
| 2686 | REACTOME\_TCR\_SIGNALING |  | 45 | 0.38 | 0.79 | 0.857 | 1.000 | 1.000 | 3666 | tags=29%, list=23%, signal=38% |
| 2687 | OSADA\_ASCL1\_TARGETS\_DN |  | 24 | 0.40 | 0.79 | 0.815 | 1.000 | 1.000 | 1342 | tags=17%, list=9%, signal=18% |
| 2688 | GRAESSMANN\_RESPONSE\_TO\_MC\_AND\_SERUM\_DEPRIVATION\_DN |  | 77 | 0.36 | 0.79 | 0.902 | 1.000 | 1.000 | 4553 | tags=35%, list=29%, signal=49% |
| 2689 | FIGUEROA\_AML\_METHYLATION\_CLUSTER\_7\_UP |  | 94 | 0.36 | 0.79 | 0.922 | 1.000 | 1.000 | 4692 | tags=33%, list=30%, signal=47% |
| 2690 | BERENJENO\_TRANSFORMED\_BY\_RHOA\_REVERSIBLY\_DN |  | 28 | 0.39 | 0.79 | 0.815 | 1.000 | 1.000 | 3362 | tags=43%, list=21%, signal=54% |
| 2691 | YANG\_BREAST\_CANCER\_ESR1\_BULK\_UP |  | 21 | 0.40 | 0.79 | 0.812 | 1.000 | 1.000 | 1254 | tags=19%, list=8%, signal=21% |
| 2692 | ST\_WNT\_CA2\_CYCLIC\_GMP\_PATHWAY |  | 20 | 0.40 | 0.79 | 0.802 | 1.000 | 1.000 | 4729 | tags=30%, list=30%, signal=43% |
| 2693 | TAKEDA\_TARGETS\_OF\_NUP98\_HOXA9\_FUSION\_16D\_DN |  | 105 | 0.36 | 0.79 | 0.930 | 1.000 | 1.000 | 3006 | tags=19%, list=19%, signal=23% |
| 2694 | ELVIDGE\_HYPOXIA\_BY\_DMOG\_UP |  | 118 | 0.36 | 0.79 | 0.938 | 1.000 | 1.000 | 3491 | tags=28%, list=22%, signal=36% |
| 2695 | SARTIPY\_BLUNTED\_BY\_INSULIN\_RESISTANCE\_DN |  | 16 | 0.42 | 0.79 | 0.814 | 1.000 | 1.000 | 3245 | tags=25%, list=21%, signal=31% |
| 2696 | LANDIS\_ERBB2\_BREAST\_PRENEOPLASTIC\_DN |  | 51 | 0.37 | 0.79 | 0.874 | 1.000 | 1.000 | 4431 | tags=39%, list=28%, signal=54% |
| 2697 | CHANG\_CORE\_SERUM\_RESPONSE\_DN |  | 168 | 0.36 | 0.79 | 0.964 | 1.000 | 1.000 | 4700 | tags=36%, list=30%, signal=50% |
| 2698 | BIOCARTA\_TOB1\_PATHWAY |  | 19 | 0.41 | 0.79 | 0.802 | 1.000 | 1.000 | 6532 | tags=42%, list=41%, signal=72% |
| 2699 | PID\_GMCSF\_PATHWAY |  | 36 | 0.38 | 0.79 | 0.838 | 1.000 | 1.000 | 5230 | tags=44%, list=33%, signal=66% |
| 2700 | VANHARANTA\_UTERINE\_FIBROID\_UP |  | 42 | 0.38 | 0.79 | 0.834 | 1.000 | 1.000 | 4902 | tags=29%, list=31%, signal=41% |
| 2701 | NIKOLSKY\_BREAST\_CANCER\_17Q21\_Q25\_AMPLICON |  | 282 | 0.35 | 0.79 | 0.994 | 1.000 | 1.000 | 4091 | tags=24%, list=26%, signal=32% |
| 2702 | REACTOME\_GLUCAGON\_SIGNALING\_IN\_METABOLIC\_REGULATION |  | 32 | 0.38 | 0.79 | 0.832 | 1.000 | 1.000 | 5784 | tags=44%, list=37%, signal=69% |
| 2703 | MORI\_LARGE\_PRE\_BII\_LYMPHOCYTE\_DN |  | 52 | 0.37 | 0.79 | 0.865 | 1.000 | 1.000 | 3363 | tags=23%, list=21%, signal=29% |
| 2704 | BASSO\_CD40\_SIGNALING\_DN |  | 61 | 0.37 | 0.79 | 0.896 | 1.000 | 1.000 | 2919 | tags=18%, list=19%, signal=22% |
| 2705 | SHEDDEN\_LUNG\_CANCER\_GOOD\_SURVIVAL\_A4 |  | 165 | 0.36 | 0.79 | 0.968 | 1.000 | 1.000 | 4138 | tags=23%, list=26%, signal=31% |
| 2706 | TAKEDA\_TARGETS\_OF\_NUP98\_HOXA9\_FUSION\_10D\_UP |  | 157 | 0.35 | 0.79 | 0.951 | 1.000 | 1.000 | 3636 | tags=19%, list=23%, signal=25% |
| 2707 | KAAB\_FAILED\_HEART\_VENTRICLE\_DN |  | 38 | 0.37 | 0.79 | 0.855 | 1.000 | 1.000 | 2452 | tags=26%, list=16%, signal=31% |
| 2708 | MIKKELSEN\_IPS\_WITH\_HCP\_H3K27ME3 |  | 97 | 0.36 | 0.79 | 0.922 | 1.000 | 1.000 | 2491 | tags=6%, list=16%, signal=7% |
| 2709 | ELVIDGE\_HYPOXIA\_UP |  | 153 | 0.36 | 0.79 | 0.964 | 1.000 | 1.000 | 3519 | tags=27%, list=22%, signal=34% |
| 2710 | VALK\_AML\_CLUSTER\_9 |  | 33 | 0.38 | 0.79 | 0.852 | 1.000 | 1.000 | 1188 | tags=12%, list=8%, signal=13% |
| 2711 | PENG\_RAPAMYCIN\_RESPONSE\_UP |  | 177 | 0.35 | 0.79 | 0.979 | 1.000 | 1.000 | 2482 | tags=19%, list=16%, signal=22% |
| 2712 | BIOCARTA\_IL2\_PATHWAY |  | 22 | 0.40 | 0.79 | 0.819 | 1.000 | 1.000 | 5230 | tags=41%, list=33%, signal=61% |
| 2713 | KAAB\_HEART\_ATRIUM\_VS\_VENTRICLE\_DN |  | 243 | 0.35 | 0.79 | 0.987 | 1.000 | 1.000 | 4690 | tags=35%, list=30%, signal=48% |
| 2714 | GARGALOVIC\_RESPONSE\_TO\_OXIDIZED\_PHOSPHOLIPIDS\_RED\_DN |  | 22 | 0.39 | 0.79 | 0.814 | 1.000 | 1.000 | 1158 | tags=14%, list=7%, signal=15% |
| 2715 | MEISSNER\_NPC\_ICP\_WITH\_H3K4ME3 |  | 16 | 0.42 | 0.79 | 0.808 | 1.000 | 1.000 | 3590 | tags=31%, list=23%, signal=40% |
| 2716 | SENESE\_HDAC1\_TARGETS\_DN |  | 204 | 0.35 | 0.79 | 0.980 | 1.000 | 1.000 | 3635 | tags=25%, list=23%, signal=31% |
| 2717 | KEGG\_ASCORBATE\_AND\_ALDARATE\_METABOLISM |  | 18 | 0.41 | 0.79 | 0.830 | 1.000 | 1.000 | 76 | tags=6%, list=0%, signal=6% |
| 2718 | ZHAN\_MULTIPLE\_MYELOMA\_CD1\_VS\_CD2\_DN |  | 44 | 0.38 | 0.79 | 0.865 | 1.000 | 1.000 | 4477 | tags=41%, list=28%, signal=57% |
| 2719 | MCBRYAN\_PUBERTAL\_BREAST\_5\_6WK\_UP |  | 105 | 0.36 | 0.79 | 0.937 | 1.000 | 1.000 | 3061 | tags=19%, list=19%, signal=23% |
| 2720 | ACEVEDO\_LIVER\_CANCER\_DN |  | 454 | 0.35 | 0.79 | 0.997 | 1.000 | 1.000 | 3216 | tags=22%, list=20%, signal=26% |
| 2721 | BHAT\_ESR1\_TARGETS\_VIA\_AKT1\_UP |  | 242 | 0.35 | 0.78 | 0.991 | 1.000 | 1.000 | 3705 | tags=25%, list=24%, signal=32% |
| 2722 | LIU\_PROSTATE\_CANCER\_DN |  | 413 | 0.35 | 0.78 | 0.998 | 1.000 | 1.000 | 2436 | tags=17%, list=15%, signal=19% |
| 2723 | LABBE\_WNT3A\_TARGETS\_DN |  | 90 | 0.36 | 0.78 | 0.919 | 1.000 | 1.000 | 1044 | tags=9%, list=7%, signal=9% |
| 2724 | REACTOME\_SYNTHESIS\_OF\_PA |  | 22 | 0.40 | 0.78 | 0.801 | 1.000 | 1.000 | 1120 | tags=14%, list=7%, signal=15% |
| 2725 | ODONNELL\_TFRC\_TARGETS\_UP |  | 329 | 0.35 | 0.78 | 0.996 | 1.000 | 1.000 | 3497 | tags=18%, list=22%, signal=22% |
| 2726 | OUILLETTE\_CLL\_13Q14\_DELETION\_DN |  | 52 | 0.37 | 0.78 | 0.883 | 1.000 | 1.000 | 5143 | tags=40%, list=33%, signal=60% |
| 2727 | ZHOU\_INFLAMMATORY\_RESPONSE\_FIMA\_UP |  | 403 | 0.35 | 0.78 | 0.998 | 1.000 | 1.000 | 3738 | tags=15%, list=24%, signal=20% |
| 2728 | KEGG\_PROPANOATE\_METABOLISM |  | 30 | 0.38 | 0.78 | 0.840 | 1.000 | 1.000 | 4171 | tags=47%, list=26%, signal=63% |
| 2729 | DACOSTA\_UV\_RESPONSE\_VIA\_ERCC3\_TTD\_UP |  | 58 | 0.36 | 0.78 | 0.897 | 1.000 | 1.000 | 3780 | tags=28%, list=24%, signal=36% |
| 2730 | KEGG\_DILATED\_CARDIOMYOPATHY |  | 89 | 0.36 | 0.78 | 0.916 | 1.000 | 1.000 | 2877 | tags=12%, list=18%, signal=15% |
| 2731 | GENTILE\_UV\_HIGH\_DOSE\_UP |  | 23 | 0.39 | 0.78 | 0.828 | 1.000 | 1.000 | 6082 | tags=52%, list=39%, signal=85% |
| 2732 | SAKAI\_CHRONIC\_HEPATITIS\_VS\_LIVER\_CANCER\_DN |  | 31 | 0.38 | 0.78 | 0.839 | 1.000 | 1.000 | 4342 | tags=26%, list=28%, signal=36% |
| 2733 | MIKKELSEN\_NPC\_LCP\_WITH\_H3K4ME3 |  | 48 | 0.37 | 0.78 | 0.880 | 1.000 | 1.000 | 3444 | tags=19%, list=22%, signal=24% |
| 2734 | TIAN\_BHLHA15\_TARGETS |  | 15 | 0.42 | 0.78 | 0.819 | 1.000 | 1.000 | 3357 | tags=27%, list=21%, signal=34% |
| 2735 | YAMASHITA\_METHYLATED\_IN\_PROSTATE\_CANCER |  | 54 | 0.37 | 0.78 | 0.893 | 1.000 | 1.000 | 2206 | tags=15%, list=14%, signal=17% |
| 2736 | HARRIS\_BRAIN\_CANCER\_PROGENITORS |  | 38 | 0.38 | 0.78 | 0.875 | 1.000 | 1.000 | 1454 | tags=13%, list=9%, signal=14% |
| 2737 | CORRE\_MULTIPLE\_MYELOMA\_UP |  | 59 | 0.36 | 0.78 | 0.905 | 1.000 | 1.000 | 2979 | tags=12%, list=19%, signal=15% |
| 2738 | CHUNG\_BLISTER\_CYTOTOXICITY\_DN |  | 34 | 0.38 | 0.78 | 0.860 | 1.000 | 1.000 | 3763 | tags=26%, list=24%, signal=35% |
| 2739 | KAAB\_HEART\_ATRIUM\_VS\_VENTRICLE\_UP |  | 231 | 0.35 | 0.78 | 0.992 | 1.000 | 1.000 | 3323 | tags=22%, list=21%, signal=27% |
| 2740 | KEGG\_FC\_EPSILON\_RI\_SIGNALING\_PATHWAY |  | 76 | 0.36 | 0.78 | 0.925 | 1.000 | 1.000 | 5031 | tags=36%, list=32%, signal=52% |
| 2741 | REACTOME\_ADP\_SIGNALLING\_THROUGH\_P2RY1 |  | 25 | 0.39 | 0.78 | 0.832 | 1.000 | 1.000 | 5348 | tags=36%, list=34%, signal=54% |
| 2742 | BRUINS\_UVC\_RESPONSE\_VIA\_TP53\_GROUP\_B |  | 479 | 0.35 | 0.78 | 1.000 | 1.000 | 1.000 | 3020 | tags=19%, list=19%, signal=23% |
| 2743 | KLEIN\_PRIMARY\_EFFUSION\_LYMPHOMA\_DN |  | 51 | 0.37 | 0.78 | 0.881 | 1.000 | 1.000 | 2238 | tags=14%, list=14%, signal=16% |
| 2744 | VALK\_AML\_CLUSTER\_16 |  | 23 | 0.40 | 0.78 | 0.825 | 1.000 | 1.000 | 616 | tags=9%, list=4%, signal=9% |
| 2745 | WOTTON\_RUNX\_TARGETS\_UP |  | 16 | 0.41 | 0.78 | 0.800 | 1.000 | 1.000 | 2924 | tags=19%, list=19%, signal=23% |
| 2746 | REACTOME\_NUCLEOTIDE\_BINDING\_DOMAIN\_LEUCINE\_RICH\_REPEAT\_CONTAINING\_RECEPTOR\_NLR\_SIGNALING\_PATHWAYS |  | 41 | 0.37 | 0.78 | 0.860 | 1.000 | 1.000 | 5081 | tags=44%, list=32%, signal=65% |
| 2747 | TAKEDA\_TARGETS\_OF\_NUP98\_HOXA9\_FUSION\_3D\_UP |  | 143 | 0.35 | 0.78 | 0.967 | 1.000 | 1.000 | 3036 | tags=16%, list=19%, signal=20% |
| 2748 | ZHANG\_GATA6\_TARGETS\_DN |  | 60 | 0.36 | 0.78 | 0.902 | 1.000 | 1.000 | 1715 | tags=8%, list=11%, signal=9% |
| 2749 | MASSARWEH\_TAMOXIFEN\_RESISTANCE\_UP |  | 467 | 0.34 | 0.78 | 0.999 | 1.000 | 1.000 | 4622 | tags=31%, list=29%, signal=42% |
| 2750 | REACTOME\_NEURONAL\_SYSTEM |  | 267 | 0.35 | 0.78 | 0.991 | 1.000 | 1.000 | 3484 | tags=12%, list=22%, signal=15% |
| 2751 | DIAZ\_CHRONIC\_MEYLOGENOUS\_LEUKEMIA\_DN |  | 104 | 0.35 | 0.78 | 0.943 | 1.000 | 1.000 | 2354 | tags=13%, list=15%, signal=16% |
| 2752 | KEGG\_VALINE\_LEUCINE\_AND\_ISOLEUCINE\_DEGRADATION |  | 43 | 0.37 | 0.78 | 0.875 | 1.000 | 1.000 | 3147 | tags=33%, list=20%, signal=41% |
| 2753 | SERVITJA\_ISLET\_HNF1A\_TARGETS\_UP |  | 159 | 0.35 | 0.78 | 0.975 | 1.000 | 1.000 | 2313 | tags=16%, list=15%, signal=18% |
| 2754 | KORKOLA\_YOLK\_SAC\_TUMOR |  | 52 | 0.36 | 0.77 | 0.892 | 1.000 | 1.000 | 3185 | tags=17%, list=20%, signal=22% |
| 2755 | MCLACHLAN\_DENTAL\_CARIES\_DN |  | 202 | 0.35 | 0.77 | 0.980 | 1.000 | 1.000 | 1626 | tags=10%, list=10%, signal=11% |
| 2756 | WANG\_ESOPHAGUS\_CANCER\_VS\_NORMAL\_UP |  | 111 | 0.35 | 0.77 | 0.948 | 1.000 | 1.000 | 2606 | tags=17%, list=17%, signal=20% |
| 2757 | FRIDMAN\_IMMORTALIZATION\_DN |  | 32 | 0.38 | 0.77 | 0.847 | 1.000 | 1.000 | 2925 | tags=28%, list=19%, signal=34% |
| 2758 | CROONQUIST\_NRAS\_SIGNALING\_UP |  | 35 | 0.37 | 0.77 | 0.852 | 1.000 | 1.000 | 1562 | tags=9%, list=10%, signal=9% |
| 2759 | GRADE\_COLON\_VS\_RECTAL\_CANCER\_DN |  | 48 | 0.36 | 0.77 | 0.892 | 1.000 | 1.000 | 3817 | tags=27%, list=24%, signal=36% |
| 2760 | MULLIGHAN\_MLL\_SIGNATURE\_1\_UP |  | 326 | 0.34 | 0.77 | 1.000 | 1.000 | 1.000 | 3988 | tags=25%, list=25%, signal=33% |
| 2761 | KEGG\_LEUKOCYTE\_TRANSENDOTHELIAL\_MIGRATION |  | 107 | 0.35 | 0.77 | 0.947 | 1.000 | 1.000 | 2393 | tags=17%, list=15%, signal=20% |
| 2762 | STARK\_HYPPOCAMPUS\_22Q11\_DELETION\_DN |  | 17 | 0.40 | 0.77 | 0.823 | 1.000 | 1.000 | 2950 | tags=24%, list=19%, signal=29% |
| 2763 | BIOCARTA\_MTA3\_PATHWAY |  | 17 | 0.41 | 0.77 | 0.838 | 1.000 | 1.000 | 4078 | tags=47%, list=26%, signal=63% |
| 2764 | JIANG\_TIP30\_TARGETS\_DN |  | 22 | 0.39 | 0.77 | 0.836 | 1.000 | 1.000 | 2288 | tags=27%, list=15%, signal=32% |
| 2765 | HUPER\_BREAST\_BASAL\_VS\_LUMINAL\_DN |  | 54 | 0.37 | 0.77 | 0.903 | 1.000 | 1.000 | 2031 | tags=20%, list=13%, signal=23% |
| 2766 | REACTOME\_METABOLISM\_OF\_LIPIDS\_AND\_LIPOPROTEINS |  | 403 | 0.34 | 0.77 | 1.000 | 1.000 | 1.000 | 4431 | tags=30%, list=28%, signal=40% |
| 2767 | GOBERT\_CORE\_OLIGODENDROCYTE\_DIFFERENTIATION |  | 39 | 0.36 | 0.77 | 0.862 | 1.000 | 1.000 | 6132 | tags=51%, list=39%, signal=84% |
| 2768 | KAYO\_CALORIE\_RESTRICTION\_MUSCLE\_UP |  | 87 | 0.35 | 0.77 | 0.941 | 1.000 | 1.000 | 3596 | tags=24%, list=23%, signal=31% |
| 2769 | REACTOME\_POST\_TRANSLATIONAL\_MODIFICATION\_SYNTHESIS\_OF\_GPI\_ANCHORED\_PROTEINS |  | 26 | 0.38 | 0.77 | 0.856 | 1.000 | 1.000 | 2854 | tags=19%, list=18%, signal=23% |
| 2770 | JOHNSTONE\_PARVB\_TARGETS\_2\_UP |  | 125 | 0.35 | 0.77 | 0.960 | 1.000 | 1.000 | 2884 | tags=24%, list=18%, signal=29% |
| 2771 | REACTOME\_POST\_TRANSLATIONAL\_PROTEIN\_MODIFICATION |  | 166 | 0.35 | 0.77 | 0.975 | 1.000 | 1.000 | 4615 | tags=31%, list=29%, signal=44% |
| 2772 | REACTOME\_SIGNAL\_AMPLIFICATION |  | 31 | 0.38 | 0.77 | 0.851 | 1.000 | 1.000 | 5348 | tags=35%, list=34%, signal=54% |
| 2773 | KEGG\_MELANOGENESIS |  | 97 | 0.35 | 0.77 | 0.942 | 1.000 | 1.000 | 4622 | tags=26%, list=29%, signal=36% |
| 2774 | TAKEDA\_TARGETS\_OF\_NUP98\_HOXA9\_FUSION\_16D\_UP |  | 141 | 0.35 | 0.77 | 0.972 | 1.000 | 1.000 | 3636 | tags=21%, list=23%, signal=27% |
| 2775 | KUROZUMI\_RESPONSE\_TO\_ONCOCYTIC\_VIRUS |  | 39 | 0.37 | 0.77 | 0.870 | 1.000 | 1.000 | 33 | tags=3%, list=0%, signal=3% |
| 2776 | ROY\_WOUND\_BLOOD\_VESSEL\_UP |  | 45 | 0.36 | 0.77 | 0.887 | 1.000 | 1.000 | 4472 | tags=27%, list=28%, signal=37% |
| 2777 | LEE\_NAIVE\_T\_LYMPHOCYTE |  | 15 | 0.40 | 0.77 | 0.831 | 1.000 | 1.000 | 5176 | tags=53%, list=33%, signal=79% |
| 2778 | WEST\_ADRENOCORTICAL\_TUMOR\_DN |  | 480 | 0.34 | 0.77 | 1.000 | 1.000 | 1.000 | 2681 | tags=17%, list=17%, signal=20% |
| 2779 | FARDIN\_HYPOXIA\_11 |  | 26 | 0.38 | 0.77 | 0.841 | 1.000 | 1.000 | 5421 | tags=54%, list=34%, signal=82% |
| 2780 | BIOCARTA\_SPRY\_PATHWAY |  | 18 | 0.40 | 0.76 | 0.843 | 1.000 | 1.000 | 5230 | tags=44%, list=33%, signal=66% |
| 2781 | BIOCARTA\_IL1R\_PATHWAY |  | 31 | 0.37 | 0.76 | 0.862 | 1.000 | 1.000 | 6532 | tags=55%, list=41%, signal=94% |
| 2782 | LINDSTEDT\_DENDRITIC\_CELL\_MATURATION\_B |  | 49 | 0.36 | 0.76 | 0.894 | 1.000 | 1.000 | 3144 | tags=24%, list=20%, signal=31% |
| 2783 | PID\_TXA2PATHWAY |  | 54 | 0.36 | 0.76 | 0.915 | 1.000 | 1.000 | 4482 | tags=30%, list=28%, signal=41% |
| 2784 | KEGG\_CHEMOKINE\_SIGNALING\_PATHWAY |  | 167 | 0.34 | 0.76 | 0.982 | 1.000 | 1.000 | 4271 | tags=22%, list=27%, signal=30% |
| 2785 | SCHRAETS\_MLL\_TARGETS\_UP |  | 34 | 0.37 | 0.76 | 0.877 | 1.000 | 1.000 | 1507 | tags=18%, list=10%, signal=19% |
| 2786 | REACTOME\_DOWNSTREAM\_TCR\_SIGNALING |  | 28 | 0.38 | 0.76 | 0.850 | 1.000 | 1.000 | 3450 | tags=32%, list=22%, signal=41% |
| 2787 | GARGALOVIC\_RESPONSE\_TO\_OXIDIZED\_PHOSPHOLIPIDS\_GREY\_DN |  | 49 | 0.36 | 0.76 | 0.903 | 1.000 | 1.000 | 3957 | tags=22%, list=25%, signal=30% |
| 2788 | MATZUK\_SPERMATOGONIA |  | 23 | 0.38 | 0.76 | 0.847 | 1.000 | 1.000 | 5096 | tags=35%, list=32%, signal=51% |
| 2789 | SIG\_PIP3\_SIGNALING\_IN\_B\_LYMPHOCYTES |  | 35 | 0.37 | 0.76 | 0.862 | 1.000 | 1.000 | 5133 | tags=37%, list=33%, signal=55% |
| 2790 | HAN\_SATB1\_TARGETS\_UP |  | 330 | 0.34 | 0.76 | 0.997 | 1.000 | 1.000 | 3643 | tags=24%, list=23%, signal=30% |
| 2791 | MAGRANGEAS\_MULTIPLE\_MYELOMA\_IGLL\_VS\_IGLK\_UP |  | 39 | 0.36 | 0.76 | 0.879 | 1.000 | 1.000 | 3415 | tags=26%, list=22%, signal=33% |
| 2792 | PEDRIOLI\_MIR31\_TARGETS\_UP |  | 162 | 0.34 | 0.76 | 0.976 | 1.000 | 1.000 | 2673 | tags=14%, list=17%, signal=17% |
| 2793 | BILBAN\_B\_CLL\_LPL\_DN |  | 32 | 0.37 | 0.76 | 0.877 | 1.000 | 1.000 | 1523 | tags=16%, list=10%, signal=17% |
| 2794 | VALK\_AML\_CLUSTER\_2 |  | 24 | 0.38 | 0.76 | 0.837 | 1.000 | 1.000 | 2021 | tags=17%, list=13%, signal=19% |
| 2795 | HERNANDEZ\_ABERRANT\_MITOSIS\_BY\_DOCETACEL\_2NM\_UP |  | 72 | 0.35 | 0.76 | 0.929 | 1.000 | 1.000 | 3363 | tags=24%, list=21%, signal=30% |
| 2796 | WILENSKY\_RESPONSE\_TO\_DARAPLADIB |  | 28 | 0.38 | 0.76 | 0.859 | 1.000 | 1.000 | 2134 | tags=11%, list=14%, signal=12% |
| 2797 | BIOCARTA\_GH\_PATHWAY |  | 25 | 0.38 | 0.76 | 0.866 | 1.000 | 1.000 | 5230 | tags=52%, list=33%, signal=78% |
| 2798 | REACTOME\_SULFUR\_AMINO\_ACID\_METABOLISM |  | 23 | 0.38 | 0.76 | 0.858 | 1.000 | 1.000 | 3857 | tags=43%, list=24%, signal=57% |
| 2799 | EBAUER\_TARGETS\_OF\_PAX3\_FOXO1\_FUSION\_UP |  | 184 | 0.34 | 0.76 | 0.987 | 1.000 | 1.000 | 3010 | tags=16%, list=19%, signal=20% |
| 2800 | LINDGREN\_BLADDER\_CANCER\_CLUSTER\_2B |  | 343 | 0.34 | 0.76 | 0.999 | 1.000 | 1.000 | 2112 | tags=15%, list=13%, signal=17% |
| 2801 | BIOCARTA\_INTRINSIC\_PATHWAY |  | 23 | 0.38 | 0.76 | 0.848 | 1.000 | 1.000 | 2645 | tags=9%, list=17%, signal=10% |
| 2802 | GARGALOVIC\_RESPONSE\_TO\_OXIDIZED\_PHOSPHOLIPIDS\_GREY\_UP |  | 16 | 0.40 | 0.76 | 0.844 | 1.000 | 1.000 | 5293 | tags=44%, list=34%, signal=66% |
| 2803 | GARGALOVIC\_RESPONSE\_TO\_OXIDIZED\_PHOSPHOLIPIDS\_BLUE\_DN |  | 47 | 0.36 | 0.76 | 0.899 | 1.000 | 1.000 | 5066 | tags=36%, list=32%, signal=53% |
| 2804 | WAMUNYOKOLI\_OVARIAN\_CANCER\_GRADES\_1\_2\_UP |  | 121 | 0.34 | 0.76 | 0.970 | 1.000 | 1.000 | 2948 | tags=23%, list=19%, signal=28% |
| 2805 | LEE\_LIVER\_CANCER\_E2F1\_UP |  | 58 | 0.35 | 0.76 | 0.910 | 1.000 | 1.000 | 3300 | tags=26%, list=21%, signal=33% |
| 2806 | AMIT\_SERUM\_RESPONSE\_240\_MCF10A |  | 52 | 0.35 | 0.76 | 0.898 | 1.000 | 1.000 | 2977 | tags=23%, list=19%, signal=28% |
| 2807 | VALK\_AML\_CLUSTER\_4 |  | 24 | 0.38 | 0.76 | 0.848 | 1.000 | 1.000 | 4142 | tags=29%, list=26%, signal=40% |
| 2808 | HELLER\_HDAC\_TARGETS\_SILENCED\_BY\_METHYLATION\_UP |  | 382 | 0.34 | 0.76 | 0.999 | 1.000 | 1.000 | 3025 | tags=19%, list=19%, signal=22% |
| 2809 | PETROVA\_ENDOTHELIUM\_LYMPHATIC\_VS\_BLOOD\_DN |  | 150 | 0.34 | 0.75 | 0.974 | 1.000 | 1.000 | 3900 | tags=32%, list=25%, signal=42% |
| 2810 | KEGG\_JAK\_STAT\_SIGNALING\_PATHWAY |  | 137 | 0.34 | 0.75 | 0.980 | 1.000 | 1.000 | 3573 | tags=16%, list=23%, signal=21% |
| 2811 | SCHAEFFER\_PROSTATE\_DEVELOPMENT\_12HR\_UP |  | 108 | 0.34 | 0.75 | 0.966 | 1.000 | 1.000 | 1595 | tags=11%, list=10%, signal=12% |
| 2812 | BIOCARTA\_RAC1\_PATHWAY |  | 23 | 0.38 | 0.75 | 0.868 | 1.000 | 1.000 | 6662 | tags=57%, list=42%, signal=98% |
| 2813 | LEE\_EARLY\_T\_LYMPHOCYTE\_DN |  | 46 | 0.35 | 0.75 | 0.884 | 1.000 | 1.000 | 1314 | tags=9%, list=8%, signal=9% |
| 2814 | CHIANG\_LIVER\_CANCER\_SUBCLASS\_UNANNOTATED\_UP |  | 65 | 0.34 | 0.75 | 0.916 | 1.000 | 1.000 | 5898 | tags=40%, list=37%, signal=64% |
| 2815 | TENEDINI\_MEGAKARYOCYTE\_MARKERS |  | 62 | 0.35 | 0.75 | 0.928 | 1.000 | 1.000 | 4429 | tags=26%, list=28%, signal=36% |
| 2816 | BERENJENO\_TRANSFORMED\_BY\_RHOA\_DN |  | 360 | 0.33 | 0.75 | 1.000 | 1.000 | 1.000 | 3664 | tags=27%, list=23%, signal=34% |
| 2817 | LI\_WILMS\_TUMOR\_VS\_FETAL\_KIDNEY\_2\_DN |  | 51 | 0.35 | 0.75 | 0.908 | 1.000 | 1.000 | 1087 | tags=14%, list=7%, signal=15% |
| 2818 | LIU\_PROSTATE\_CANCER\_UP |  | 76 | 0.34 | 0.75 | 0.933 | 1.000 | 1.000 | 2163 | tags=18%, list=14%, signal=21% |
| 2819 | STREICHER\_LSM1\_TARGETS\_UP |  | 42 | 0.36 | 0.75 | 0.896 | 1.000 | 1.000 | 4404 | tags=33%, list=28%, signal=46% |
| 2820 | REACTOME\_REGULATION\_OF\_IFNA\_SIGNALING |  | 16 | 0.39 | 0.75 | 0.835 | 1.000 | 1.000 | 5041 | tags=25%, list=32%, signal=37% |
| 2821 | LENAOUR\_DENDRITIC\_CELL\_MATURATION\_DN |  | 117 | 0.34 | 0.75 | 0.972 | 1.000 | 1.000 | 3922 | tags=26%, list=25%, signal=35% |
| 2822 | KANG\_GIST\_WITH\_PDGFRA\_UP |  | 46 | 0.35 | 0.75 | 0.900 | 1.000 | 1.000 | 2452 | tags=17%, list=16%, signal=21% |
| 2823 | BONOME\_OVARIAN\_CANCER\_POOR\_SURVIVAL\_UP |  | 28 | 0.37 | 0.75 | 0.891 | 1.000 | 1.000 | 3445 | tags=18%, list=22%, signal=23% |
| 2824 | REACTOME\_TRANSMEMBRANE\_TRANSPORT\_OF\_SMALL\_MOLECULES |  | 399 | 0.33 | 0.75 | 0.997 | 1.000 | 1.000 | 4139 | tags=20%, list=26%, signal=26% |
| 2825 | ZHONG\_RESPONSE\_TO\_AZACITIDINE\_AND\_TSA\_UP |  | 155 | 0.34 | 0.75 | 0.986 | 1.000 | 1.000 | 3426 | tags=23%, list=22%, signal=29% |
| 2826 | KANG\_IMMORTALIZED\_BY\_TERT\_UP |  | 81 | 0.34 | 0.74 | 0.944 | 1.000 | 1.000 | 2039 | tags=16%, list=13%, signal=18% |
| 2827 | KEGG\_ABC\_TRANSPORTERS |  | 42 | 0.35 | 0.74 | 0.906 | 1.000 | 1.000 | 3983 | tags=21%, list=25%, signal=29% |
| 2828 | REACTOME\_TRAF6\_MEDIATED\_IRF7\_ACTIVATION |  | 20 | 0.38 | 0.74 | 0.868 | 1.000 | 1.000 | 5068 | tags=35%, list=32%, signal=52% |
| 2829 | SASAI\_RESISTANCE\_TO\_NEOPLASTIC\_TRANSFROMATION |  | 50 | 0.35 | 0.74 | 0.902 | 1.000 | 1.000 | 1988 | tags=18%, list=13%, signal=21% |
| 2830 | DASU\_IL6\_SIGNALING\_UP |  | 55 | 0.35 | 0.74 | 0.915 | 1.000 | 1.000 | 1959 | tags=15%, list=12%, signal=17% |
| 2831 | HANN\_RESISTANCE\_TO\_BCL2\_INHIBITOR\_DN |  | 46 | 0.35 | 0.74 | 0.903 | 1.000 | 1.000 | 2157 | tags=15%, list=14%, signal=18% |
| 2832 | RASHI\_RESPONSE\_TO\_IONIZING\_RADIATION\_2 |  | 115 | 0.34 | 0.74 | 0.962 | 1.000 | 1.000 | 3025 | tags=25%, list=19%, signal=31% |
| 2833 | FUKUSHIMA\_TNFSF11\_TARGETS |  | 16 | 0.39 | 0.74 | 0.858 | 1.000 | 1.000 | 230 | tags=6%, list=1%, signal=6% |
| 2834 | WELCH\_GATA1\_TARGETS |  | 21 | 0.38 | 0.74 | 0.877 | 1.000 | 1.000 | 5842 | tags=38%, list=37%, signal=60% |
| 2835 | VARELA\_ZMPSTE24\_TARGETS\_UP |  | 36 | 0.35 | 0.74 | 0.890 | 1.000 | 1.000 | 3945 | tags=28%, list=25%, signal=37% |
| 2836 | BIOCARTA\_CDMAC\_PATHWAY |  | 15 | 0.39 | 0.74 | 0.844 | 1.000 | 1.000 | 5031 | tags=47%, list=32%, signal=69% |
| 2837 | KEGG\_GLUTATHIONE\_METABOLISM |  | 44 | 0.35 | 0.74 | 0.895 | 1.000 | 1.000 | 4398 | tags=45%, list=28%, signal=63% |
| 2838 | BIOCARTA\_CD40\_PATHWAY |  | 15 | 0.39 | 0.74 | 0.845 | 1.000 | 1.000 | 3201 | tags=27%, list=20%, signal=33% |
| 2839 | YANG\_BREAST\_CANCER\_ESR1\_LASER\_UP |  | 27 | 0.36 | 0.74 | 0.890 | 1.000 | 1.000 | 4970 | tags=44%, list=32%, signal=65% |
| 2840 | ICHIBA\_GRAFT\_VERSUS\_HOST\_DISEASE\_35D\_DN |  | 45 | 0.35 | 0.74 | 0.913 | 1.000 | 1.000 | 3553 | tags=16%, list=23%, signal=20% |
| 2841 | REACTOME\_INTERFERON\_GAMMA\_SIGNALING |  | 48 | 0.35 | 0.74 | 0.913 | 1.000 | 1.000 | 3286 | tags=25%, list=21%, signal=31% |
| 2842 | LEE\_LIVER\_CANCER\_MYC\_DN |  | 58 | 0.34 | 0.74 | 0.915 | 1.000 | 1.000 | 1637 | tags=14%, list=10%, signal=15% |
| 2843 | HERNANDEZ\_MITOTIC\_ARREST\_BY\_DOCETAXEL\_2\_DN |  | 16 | 0.39 | 0.74 | 0.846 | 1.000 | 1.000 | 1050 | tags=19%, list=7%, signal=20% |
| 2844 | HUANG\_FOXA2\_TARGETS\_UP |  | 42 | 0.36 | 0.74 | 0.905 | 1.000 | 1.000 | 3583 | tags=26%, list=23%, signal=34% |
| 2845 | MATZUK\_EMBRYONIC\_GERM\_CELL |  | 18 | 0.37 | 0.74 | 0.852 | 1.000 | 1.000 | 3522 | tags=33%, list=22%, signal=43% |
| 2846 | REACTOME\_CELL\_CELL\_COMMUNICATION |  | 113 | 0.34 | 0.74 | 0.971 | 1.000 | 1.000 | 3206 | tags=22%, list=20%, signal=28% |
| 2847 | KUUSELO\_PANCREATIC\_CANCER\_19Q13\_AMPLIFICATION |  | 22 | 0.37 | 0.74 | 0.873 | 1.000 | 1.000 | 3577 | tags=23%, list=23%, signal=29% |
| 2848 | NIELSEN\_SYNOVIAL\_SARCOMA\_DN |  | 17 | 0.38 | 0.74 | 0.859 | 1.000 | 1.000 | 151 | tags=6%, list=1%, signal=6% |
| 2849 | SWEET\_LUNG\_CANCER\_KRAS\_DN |  | 406 | 0.33 | 0.74 | 1.000 | 1.000 | 1.000 | 2712 | tags=17%, list=17%, signal=21% |
| 2850 | WORSCHECH\_TUMOR\_EVASION\_AND\_TOLEROGENICITY\_UP |  | 30 | 0.36 | 0.74 | 0.888 | 1.000 | 1.000 | 89 | tags=3%, list=1%, signal=3% |
| 2851 | REACTOME\_REGULATION\_OF\_KIT\_SIGNALING |  | 16 | 0.38 | 0.74 | 0.853 | 1.000 | 1.000 | 5703 | tags=56%, list=36%, signal=88% |
| 2852 | TARTE\_PLASMA\_CELL\_VS\_PLASMABLAST\_UP |  | 339 | 0.33 | 0.74 | 1.000 | 1.000 | 1.000 | 3043 | tags=16%, list=19%, signal=19% |
| 2853 | HENDRICKS\_SMARCA4\_TARGETS\_UP |  | 48 | 0.35 | 0.73 | 0.925 | 1.000 | 1.000 | 3630 | tags=29%, list=23%, signal=38% |
| 2854 | CHANGOLKAR\_H2AFY\_TARGETS\_UP |  | 41 | 0.35 | 0.73 | 0.913 | 1.000 | 1.000 | 4789 | tags=39%, list=30%, signal=56% |
| 2855 | MCLACHLAN\_DENTAL\_CARIES\_UP |  | 204 | 0.33 | 0.73 | 0.989 | 1.000 | 1.000 | 1626 | tags=9%, list=10%, signal=10% |
| 2856 | KYNG\_ENVIRONMENTAL\_STRESS\_RESPONSE\_NOT\_BY\_GAMMA\_IN\_OLD |  | 30 | 0.36 | 0.73 | 0.901 | 1.000 | 1.000 | 4747 | tags=40%, list=30%, signal=57% |
| 2857 | KIM\_MYC\_AMPLIFICATION\_TARGETS\_DN |  | 78 | 0.34 | 0.73 | 0.941 | 1.000 | 1.000 | 2853 | tags=18%, list=18%, signal=22% |
| 2858 | IIZUKA\_LIVER\_CANCER\_PROGRESSION\_L1\_G1\_UP |  | 19 | 0.38 | 0.73 | 0.869 | 1.000 | 1.000 | 3533 | tags=21%, list=22%, signal=27% |
| 2859 | DURCHDEWALD\_SKIN\_CARCINOGENESIS\_UP |  | 76 | 0.34 | 0.73 | 0.944 | 1.000 | 1.000 | 3491 | tags=25%, list=22%, signal=32% |
| 2860 | PID\_NFKAPPABATYPICALPATHWAY |  | 17 | 0.38 | 0.73 | 0.873 | 1.000 | 1.000 | 5315 | tags=53%, list=34%, signal=80% |
| 2861 | PASINI\_SUZ12\_TARGETS\_UP |  | 99 | 0.33 | 0.73 | 0.961 | 1.000 | 1.000 | 2038 | tags=11%, list=13%, signal=13% |
| 2862 | SCHAEFFER\_SOX9\_TARGETS\_IN\_PROSTATE\_DEVELOPMENT\_UP |  | 21 | 0.37 | 0.73 | 0.874 | 1.000 | 1.000 | 2073 | tags=19%, list=13%, signal=22% |
| 2863 | RODRIGUES\_NTN1\_AND\_DCC\_TARGETS |  | 32 | 0.36 | 0.73 | 0.897 | 1.000 | 1.000 | 3377 | tags=28%, list=21%, signal=36% |
| 2864 | TANG\_SENESCENCE\_TP53\_TARGETS\_UP |  | 31 | 0.36 | 0.73 | 0.895 | 1.000 | 1.000 | 4063 | tags=26%, list=26%, signal=35% |
| 2865 | REACTOME\_GABA\_B\_RECEPTOR\_ACTIVATION |  | 38 | 0.35 | 0.73 | 0.910 | 1.000 | 1.000 | 5784 | tags=26%, list=37%, signal=41% |
| 2866 | NIKOLSKY\_BREAST\_CANCER\_17Q11\_Q21\_AMPLICON |  | 97 | 0.33 | 0.73 | 0.955 | 1.000 | 1.000 | 3315 | tags=18%, list=21%, signal=22% |
| 2867 | KEGG\_SYSTEMIC\_LUPUS\_ERYTHEMATOSUS |  | 86 | 0.33 | 0.73 | 0.961 | 1.000 | 1.000 | 2316 | tags=12%, list=15%, signal=14% |
| 2868 | NADLER\_OBESITY\_DN |  | 47 | 0.34 | 0.73 | 0.908 | 1.000 | 1.000 | 3147 | tags=26%, list=20%, signal=32% |
| 2869 | VANHARANTA\_UTERINE\_FIBROID\_DN |  | 57 | 0.34 | 0.73 | 0.940 | 1.000 | 1.000 | 3811 | tags=25%, list=24%, signal=32% |
| 2870 | SHIN\_B\_CELL\_LYMPHOMA\_CLUSTER\_7 |  | 27 | 0.36 | 0.73 | 0.892 | 1.000 | 1.000 | 4417 | tags=41%, list=28%, signal=57% |
| 2871 | BROWNE\_HCMV\_INFECTION\_18HR\_DN |  | 158 | 0.33 | 0.73 | 0.988 | 1.000 | 1.000 | 2977 | tags=20%, list=19%, signal=24% |
| 2872 | ONDER\_CDH1\_TARGETS\_1\_UP |  | 117 | 0.33 | 0.73 | 0.980 | 1.000 | 1.000 | 2843 | tags=21%, list=18%, signal=25% |
| 2873 | REACTOME\_GABA\_RECEPTOR\_ACTIVATION |  | 52 | 0.34 | 0.73 | 0.925 | 1.000 | 1.000 | 5784 | tags=19%, list=37%, signal=30% |
| 2874 | CROMER\_METASTASIS\_DN |  | 71 | 0.34 | 0.73 | 0.941 | 1.000 | 1.000 | 2800 | tags=23%, list=18%, signal=27% |
| 2875 | ROSS\_AML\_WITH\_MLL\_FUSIONS |  | 68 | 0.34 | 0.73 | 0.957 | 1.000 | 1.000 | 845 | tags=10%, list=5%, signal=11% |
| 2876 | TURASHVILI\_BREAST\_LOBULAR\_CARCINOMA\_VS\_LOBULAR\_NORMAL\_UP |  | 83 | 0.33 | 0.73 | 0.963 | 1.000 | 1.000 | 2065 | tags=14%, list=13%, signal=17% |
| 2877 | ROVERSI\_GLIOMA\_COPY\_NUMBER\_UP |  | 90 | 0.33 | 0.73 | 0.956 | 1.000 | 1.000 | 3301 | tags=19%, list=21%, signal=24% |
| 2878 | HALMOS\_CEBPA\_TARGETS\_UP |  | 47 | 0.34 | 0.73 | 0.923 | 1.000 | 1.000 | 3666 | tags=23%, list=23%, signal=30% |
| 2879 | VERHAAK\_AML\_WITH\_NPM1\_MUTATED\_DN |  | 217 | 0.33 | 0.73 | 0.996 | 1.000 | 1.000 | 3073 | tags=17%, list=20%, signal=20% |
| 2880 | LABBE\_TGFB1\_TARGETS\_DN |  | 103 | 0.33 | 0.73 | 0.978 | 1.000 | 1.000 | 2953 | tags=17%, list=19%, signal=21% |
| 2881 | MANALO\_HYPOXIA\_UP |  | 185 | 0.33 | 0.72 | 0.994 | 1.000 | 1.000 | 3258 | tags=19%, list=21%, signal=24% |
| 2882 | REACTOME\_LIPID\_DIGESTION\_MOBILIZATION\_AND\_TRANSPORT |  | 41 | 0.34 | 0.72 | 0.908 | 1.000 | 1.000 | 4336 | tags=27%, list=28%, signal=37% |
| 2883 | MORI\_MATURE\_B\_LYMPHOCYTE\_UP |  | 82 | 0.33 | 0.72 | 0.953 | 1.000 | 1.000 | 3434 | tags=23%, list=22%, signal=29% |
| 2884 | WORSCHECH\_TUMOR\_REJECTION\_UP |  | 51 | 0.34 | 0.72 | 0.927 | 1.000 | 1.000 | 4417 | tags=18%, list=28%, signal=24% |
| 2885 | MCCLUNG\_CREB1\_TARGETS\_DN |  | 53 | 0.34 | 0.72 | 0.923 | 1.000 | 1.000 | 3322 | tags=23%, list=21%, signal=29% |
| 2886 | REACTOME\_SEMA4D\_INDUCED\_CELL\_MIGRATION\_AND\_GROWTH\_CONE\_COLLAPSE |  | 23 | 0.36 | 0.72 | 0.897 | 1.000 | 1.000 | 1682 | tags=22%, list=11%, signal=24% |
| 2887 | FIGUEROA\_AML\_METHYLATION\_CLUSTER\_4\_UP |  | 95 | 0.33 | 0.72 | 0.975 | 1.000 | 1.000 | 4737 | tags=28%, list=30%, signal=40% |
| 2888 | KASLER\_HDAC7\_TARGETS\_2\_DN |  | 30 | 0.36 | 0.72 | 0.883 | 1.000 | 1.000 | 4041 | tags=27%, list=26%, signal=36% |
| 2889 | FOSTER\_KDM1A\_TARGETS\_DN |  | 179 | 0.32 | 0.72 | 0.992 | 1.000 | 1.000 | 4087 | tags=31%, list=26%, signal=41% |
| 2890 | TONKS\_TARGETS\_OF\_RUNX1\_RUNX1T1\_FUSION\_SUSTAINDED\_IN\_ERYTHROCYTE\_UP |  | 43 | 0.34 | 0.72 | 0.917 | 1.000 | 1.000 | 1985 | tags=14%, list=13%, signal=16% |
| 2891 | KEGG\_PEROXISOME |  | 73 | 0.33 | 0.72 | 0.953 | 1.000 | 1.000 | 1661 | tags=12%, list=11%, signal=14% |
| 2892 | REACTOME\_BIOSYNTHESIS\_OF\_THE\_N\_GLYCAN\_PRECURSOR\_DOLICHOL\_LIPID\_LINKED\_OLIGOSACCHARIDE\_LLO\_AND\_TRANSFER\_TO\_A\_NASCENT\_PROTEIN |  | 27 | 0.36 | 0.72 | 0.896 | 1.000 | 1.000 | 5051 | tags=44%, list=32%, signal=65% |
| 2893 | SPIELMAN\_LYMPHOBLAST\_EUROPEAN\_VS\_ASIAN\_2FC\_DN |  | 16 | 0.37 | 0.72 | 0.867 | 1.000 | 1.000 | 1986 | tags=25%, list=13%, signal=29% |
| 2894 | TONKS\_TARGETS\_OF\_RUNX1\_RUNX1T1\_FUSION\_HSC\_DN |  | 168 | 0.32 | 0.72 | 0.991 | 1.000 | 1.000 | 2483 | tags=13%, list=16%, signal=15% |
| 2895 | ZHAN\_MULTIPLE\_MYELOMA\_MF\_UP |  | 40 | 0.34 | 0.72 | 0.905 | 1.000 | 1.000 | 3767 | tags=25%, list=24%, signal=33% |
| 2896 | CERVERA\_SDHB\_TARGETS\_1\_DN |  | 33 | 0.35 | 0.72 | 0.922 | 1.000 | 1.000 | 1004 | tags=6%, list=6%, signal=6% |
| 2897 | LEE\_DIFFERENTIATING\_T\_LYMPHOCYTE |  | 158 | 0.32 | 0.72 | 0.984 | 1.000 | 1.000 | 3720 | tags=20%, list=24%, signal=25% |
| 2898 | ALTEMEIER\_RESPONSE\_TO\_LPS\_WITH\_MECHANICAL\_VENTILATION |  | 116 | 0.33 | 0.72 | 0.984 | 1.000 | 1.000 | 3491 | tags=13%, list=22%, signal=16% |
| 2899 | REACTOME\_SYNTHESIS\_OF\_GLYCOSYLPHOSPHATIDYLINOSITOL\_GPI |  | 17 | 0.38 | 0.72 | 0.888 | 1.000 | 1.000 | 5873 | tags=41%, list=37%, signal=66% |
| 2900 | PETRETTO\_HEART\_MASS\_QTL\_CIS\_UP |  | 23 | 0.36 | 0.72 | 0.902 | 1.000 | 1.000 | 4787 | tags=43%, list=30%, signal=62% |
| 2901 | KOBAYASHI\_RESPONSE\_TO\_ROMIDEPSIN |  | 16 | 0.37 | 0.72 | 0.880 | 1.000 | 1.000 | 5270 | tags=38%, list=33%, signal=56% |
| 2902 | PEDRIOLI\_MIR31\_TARGETS\_DN |  | 329 | 0.32 | 0.72 | 0.999 | 1.000 | 1.000 | 4954 | tags=23%, list=31%, signal=33% |
| 2903 | BIOCARTA\_ERK\_PATHWAY |  | 27 | 0.35 | 0.72 | 0.897 | 1.000 | 1.000 | 5595 | tags=48%, list=36%, signal=75% |
| 2904 | HATADA\_METHYLATED\_IN\_LUNG\_CANCER\_UP |  | 326 | 0.32 | 0.71 | 1.000 | 1.000 | 1.000 | 2744 | tags=12%, list=17%, signal=14% |
| 2905 | KEGG\_RIBOFLAVIN\_METABOLISM |  | 16 | 0.37 | 0.71 | 0.884 | 1.000 | 1.000 | 5368 | tags=44%, list=34%, signal=66% |
| 2906 | XU\_GH1\_EXOGENOUS\_TARGETS\_UP |  | 66 | 0.33 | 0.71 | 0.953 | 1.000 | 1.000 | 5111 | tags=24%, list=32%, signal=36% |
| 2907 | VERHAAK\_GLIOBLASTOMA\_CLASSICAL |  | 186 | 0.32 | 0.71 | 0.994 | 1.000 | 1.000 | 3767 | tags=19%, list=24%, signal=25% |
| 2908 | AMIT\_SERUM\_RESPONSE\_40\_MCF10A |  | 28 | 0.35 | 0.71 | 0.897 | 1.000 | 1.000 | 3469 | tags=29%, list=22%, signal=37% |
| 2909 | WENG\_POR\_TARGETS\_GLOBAL\_UP |  | 18 | 0.38 | 0.71 | 0.871 | 1.000 | 1.000 | 1761 | tags=28%, list=11%, signal=31% |
| 2910 | GOLDRATH\_IMMUNE\_MEMORY |  | 63 | 0.33 | 0.71 | 0.947 | 1.000 | 1.000 | 4983 | tags=32%, list=32%, signal=46% |
| 2911 | ANASTASSIOU\_CANCER\_MESENCHYMAL\_TRANSITION\_SIGNATURE |  | 61 | 0.33 | 0.71 | 0.950 | 1.000 | 1.000 | 2125 | tags=15%, list=13%, signal=17% |
| 2912 | DAVICIONI\_MOLECULAR\_ARMS\_VS\_ERMS\_DN |  | 158 | 0.32 | 0.71 | 0.993 | 1.000 | 1.000 | 3017 | tags=20%, list=19%, signal=25% |
| 2913 | HINATA\_NFKB\_TARGETS\_KERATINOCYTE\_DN |  | 18 | 0.37 | 0.71 | 0.885 | 1.000 | 1.000 | 4906 | tags=22%, list=31%, signal=32% |
| 2914 | MIKKELSEN\_MEF\_LCP\_WITH\_H3K4ME3 |  | 110 | 0.32 | 0.71 | 0.982 | 1.000 | 1.000 | 3596 | tags=23%, list=23%, signal=29% |
| 2915 | TARTE\_PLASMA\_CELL\_VS\_B\_LYMPHOCYTE\_DN |  | 34 | 0.34 | 0.71 | 0.903 | 1.000 | 1.000 | 3674 | tags=26%, list=23%, signal=34% |
| 2916 | WALLACE\_PROSTATE\_CANCER\_UP |  | 18 | 0.37 | 0.71 | 0.866 | 1.000 | 1.000 | 1499 | tags=22%, list=10%, signal=25% |
| 2917 | SA\_B\_CELL\_RECEPTOR\_COMPLEXES |  | 24 | 0.35 | 0.71 | 0.905 | 1.000 | 1.000 | 5333 | tags=46%, list=34%, signal=69% |
| 2918 | PANGAS\_TUMOR\_SUPPRESSION\_BY\_SMAD1\_AND\_SMAD5\_UP |  | 114 | 0.32 | 0.71 | 0.985 | 1.000 | 1.000 | 2283 | tags=17%, list=14%, signal=19% |
| 2919 | HELLER\_HDAC\_TARGETS\_UP |  | 258 | 0.32 | 0.71 | 0.999 | 1.000 | 1.000 | 3006 | tags=19%, list=19%, signal=24% |
| 2920 | LIANG\_SILENCED\_BY\_METHYLATION\_2 |  | 46 | 0.34 | 0.71 | 0.923 | 1.000 | 1.000 | 3057 | tags=20%, list=19%, signal=24% |
| 2921 | PID\_ECADHERIN\_KERATINOCYTE\_PATHWAY |  | 21 | 0.36 | 0.71 | 0.900 | 1.000 | 1.000 | 2223 | tags=29%, list=14%, signal=33% |
| 2922 | HEIDENBLAD\_AMPLICON\_12P11\_12\_DN |  | 17 | 0.38 | 0.71 | 0.898 | 1.000 | 1.000 | 1058 | tags=12%, list=7%, signal=13% |
| 2923 | KEGG\_HEMATOPOIETIC\_CELL\_LINEAGE |  | 78 | 0.33 | 0.71 | 0.970 | 1.000 | 1.000 | 1485 | tags=8%, list=9%, signal=8% |
| 2924 | ROSS\_AML\_WITH\_PML\_RARA\_FUSION |  | 68 | 0.33 | 0.71 | 0.961 | 1.000 | 1.000 | 2920 | tags=18%, list=19%, signal=22% |
| 2925 | PID\_EPHRINBREVPATHWAY |  | 30 | 0.35 | 0.71 | 0.915 | 1.000 | 1.000 | 3066 | tags=20%, list=19%, signal=25% |
| 2926 | FIGUEROA\_AML\_METHYLATION\_CLUSTER\_1\_UP |  | 97 | 0.32 | 0.71 | 0.970 | 1.000 | 1.000 | 4899 | tags=25%, list=31%, signal=36% |
| 2927 | WAGNER\_APO2\_SENSITIVITY |  | 21 | 0.36 | 0.71 | 0.896 | 1.000 | 1.000 | 1139 | tags=10%, list=7%, signal=10% |
| 2928 | PARK\_TRETINOIN\_RESPONSE\_AND\_PML\_RARA\_FUSION |  | 30 | 0.35 | 0.71 | 0.901 | 1.000 | 1.000 | 1129 | tags=10%, list=7%, signal=11% |
| 2929 | DARWICHE\_PAPILLOMA\_RISK\_HIGH\_VS\_LOW\_DN |  | 27 | 0.35 | 0.70 | 0.910 | 1.000 | 1.000 | 2881 | tags=15%, list=18%, signal=18% |
| 2930 | NIELSEN\_LEIOMYOSARCOMA\_CNN1\_DN |  | 18 | 0.36 | 0.70 | 0.894 | 1.000 | 1.000 | 1555 | tags=17%, list=10%, signal=18% |
| 2931 | BOQUEST\_STEM\_CELL\_UP |  | 244 | 0.31 | 0.70 | 0.997 | 1.000 | 1.000 | 3658 | tags=20%, list=23%, signal=26% |
| 2932 | BEIER\_GLIOMA\_STEM\_CELL\_DN |  | 54 | 0.33 | 0.70 | 0.941 | 1.000 | 1.000 | 3064 | tags=24%, list=19%, signal=30% |
| 2933 | HELLER\_SILENCED\_BY\_METHYLATION\_UP |  | 237 | 0.32 | 0.70 | 0.998 | 1.000 | 1.000 | 3040 | tags=16%, list=19%, signal=20% |
| 2934 | ZHU\_CMV\_24\_HR\_DN |  | 88 | 0.32 | 0.70 | 0.967 | 1.000 | 1.000 | 3389 | tags=25%, list=22%, signal=32% |
| 2935 | YAO\_TEMPORAL\_RESPONSE\_TO\_PROGESTERONE\_CLUSTER\_6 |  | 64 | 0.33 | 0.70 | 0.952 | 1.000 | 1.000 | 2001 | tags=16%, list=13%, signal=18% |
| 2936 | SHIPP\_DLBCL\_VS\_FOLLICULAR\_LYMPHOMA\_DN |  | 42 | 0.33 | 0.70 | 0.934 | 1.000 | 1.000 | 4183 | tags=21%, list=27%, signal=29% |
| 2937 | BIOCARTA\_IL10\_PATHWAY |  | 17 | 0.36 | 0.70 | 0.888 | 1.000 | 1.000 | 5041 | tags=29%, list=32%, signal=43% |
| 2938 | GRAESSMANN\_RESPONSE\_TO\_MC\_AND\_SERUM\_DEPRIVATION\_UP |  | 181 | 0.31 | 0.70 | 0.996 | 1.000 | 1.000 | 3216 | tags=18%, list=20%, signal=22% |
| 2939 | ST\_WNT\_BETA\_CATENIN\_PATHWAY |  | 34 | 0.34 | 0.70 | 0.921 | 1.000 | 1.000 | 4808 | tags=44%, list=31%, signal=63% |
| 2940 | PHESSE\_TARGETS\_OF\_APC\_AND\_MBD2\_UP |  | 15 | 0.36 | 0.70 | 0.892 | 1.000 | 1.000 | 963 | tags=7%, list=6%, signal=7% |
| 2941 | SCHLOSSER\_SERUM\_RESPONSE\_UP |  | 112 | 0.32 | 0.70 | 0.986 | 1.000 | 1.000 | 3580 | tags=21%, list=23%, signal=28% |
| 2942 | PID\_S1P\_S1P2\_PATHWAY |  | 23 | 0.35 | 0.70 | 0.892 | 1.000 | 1.000 | 5333 | tags=48%, list=34%, signal=72% |
| 2943 | JI\_METASTASIS\_REPRESSED\_BY\_STK11 |  | 23 | 0.35 | 0.70 | 0.904 | 1.000 | 1.000 | 518 | tags=9%, list=3%, signal=9% |
| 2944 | KEGG\_TRYPTOPHAN\_METABOLISM |  | 37 | 0.34 | 0.70 | 0.925 | 1.000 | 1.000 | 4971 | tags=27%, list=32%, signal=39% |
| 2945 | RUTELLA\_RESPONSE\_TO\_CSF2RB\_AND\_IL4\_DN |  | 256 | 0.31 | 0.70 | 1.000 | 1.000 | 1.000 | 4191 | tags=25%, list=27%, signal=34% |
| 2946 | REACTOME\_NA\_CL\_DEPENDENT\_NEUROTRANSMITTER\_TRANSPORTERS |  | 16 | 0.37 | 0.70 | 0.907 | 1.000 | 1.000 | 3310 | tags=13%, list=21%, signal=16% |
| 2947 | WIKMAN\_ASBESTOS\_LUNG\_CANCER\_UP |  | 16 | 0.36 | 0.70 | 0.901 | 1.000 | 1.000 | 2675 | tags=25%, list=17%, signal=30% |
| 2948 | REACTOME\_PLATELET\_HOMEOSTASIS |  | 74 | 0.32 | 0.69 | 0.965 | 1.000 | 1.000 | 4594 | tags=22%, list=29%, signal=30% |
| 2949 | REACTOME\_N\_GLYCAN\_ANTENNAE\_ELONGATION\_IN\_THE\_MEDIAL\_TRANS\_GOLGI |  | 18 | 0.36 | 0.69 | 0.895 | 1.000 | 1.000 | 1899 | tags=17%, list=12%, signal=19% |
| 2950 | PID\_S1P\_META\_PATHWAY |  | 16 | 0.36 | 0.69 | 0.889 | 1.000 | 1.000 | 4583 | tags=38%, list=29%, signal=53% |
| 2951 | REACTOME\_CELL\_JUNCTION\_ORGANIZATION |  | 74 | 0.32 | 0.69 | 0.956 | 1.000 | 1.000 | 3555 | tags=23%, list=23%, signal=30% |
| 2952 | LANDIS\_ERBB2\_BREAST\_TUMORS\_324\_DN |  | 143 | 0.31 | 0.69 | 0.995 | 1.000 | 1.000 | 1871 | tags=17%, list=12%, signal=20% |
| 2953 | SANA\_RESPONSE\_TO\_IFNG\_UP |  | 55 | 0.32 | 0.69 | 0.958 | 1.000 | 1.000 | 2354 | tags=15%, list=15%, signal=17% |
| 2954 | GRATIAS\_RETINOBLASTOMA\_16Q24 |  | 17 | 0.36 | 0.69 | 0.917 | 1.000 | 1.000 | 3007 | tags=29%, list=19%, signal=36% |
| 2955 | ST\_GA12\_PATHWAY |  | 23 | 0.35 | 0.69 | 0.923 | 1.000 | 1.000 | 466 | tags=9%, list=3%, signal=9% |
| 2956 | RICKMAN\_HEAD\_AND\_NECK\_CANCER\_B |  | 38 | 0.33 | 0.69 | 0.940 | 1.000 | 1.000 | 3272 | tags=8%, list=21%, signal=10% |
| 2957 | TAKEDA\_TARGETS\_OF\_NUP98\_HOXA9\_FUSION\_10D\_DN |  | 116 | 0.31 | 0.69 | 0.984 | 1.000 | 1.000 | 1297 | tags=9%, list=8%, signal=9% |
| 2958 | FRASOR\_RESPONSE\_TO\_ESTRADIOL\_DN |  | 76 | 0.32 | 0.69 | 0.973 | 1.000 | 1.000 | 1353 | tags=14%, list=9%, signal=16% |
| 2959 | BRACHAT\_RESPONSE\_TO\_METHOTREXATE\_UP |  | 22 | 0.35 | 0.69 | 0.911 | 1.000 | 1.000 | 562 | tags=9%, list=4%, signal=9% |
| 2960 | YANG\_MUC2\_TARGETS\_DUODENUM\_6MO\_DN |  | 20 | 0.35 | 0.69 | 0.910 | 1.000 | 1.000 | 2779 | tags=20%, list=18%, signal=24% |
| 2961 | WANG\_RESPONSE\_TO\_GSK3\_INHIBITOR\_SB216763\_UP |  | 278 | 0.31 | 0.69 | 1.000 | 1.000 | 1.000 | 4983 | tags=30%, list=32%, signal=43% |
| 2962 | GNATENKO\_PLATELET\_SIGNATURE |  | 40 | 0.33 | 0.69 | 0.937 | 1.000 | 1.000 | 4366 | tags=35%, list=28%, signal=48% |
| 2963 | NIELSEN\_SYNOVIAL\_SARCOMA\_UP |  | 18 | 0.35 | 0.69 | 0.913 | 1.000 | 1.000 | 4056 | tags=17%, list=26%, signal=22% |
| 2964 | REACTOME\_THROMBIN\_SIGNALLING\_THROUGH\_PROTEINASE\_ACTIVATED\_RECEPTORS\_PARS |  | 32 | 0.33 | 0.69 | 0.927 | 1.000 | 1.000 | 5348 | tags=38%, list=34%, signal=57% |
| 2965 | KEGG\_NICOTINATE\_AND\_NICOTINAMIDE\_METABOLISM |  | 21 | 0.34 | 0.68 | 0.907 | 1.000 | 1.000 | 3619 | tags=24%, list=23%, signal=31% |
| 2966 | YAMASHITA\_LIVER\_CANCER\_STEM\_CELL\_DN |  | 71 | 0.32 | 0.68 | 0.974 | 1.000 | 1.000 | 2977 | tags=15%, list=19%, signal=19% |
| 2967 | DACOSTA\_ERCC3\_ALLELE\_XPCS\_VS\_TTD\_DN |  | 33 | 0.33 | 0.68 | 0.933 | 1.000 | 1.000 | 2236 | tags=15%, list=14%, signal=18% |
| 2968 | KIM\_MYCN\_AMPLIFICATION\_TARGETS\_DN |  | 89 | 0.31 | 0.68 | 0.985 | 1.000 | 1.000 | 4747 | tags=36%, list=30%, signal=51% |
| 2969 | HOEGERKORP\_CD44\_TARGETS\_DIRECT\_UP |  | 25 | 0.34 | 0.68 | 0.918 | 1.000 | 1.000 | 5512 | tags=36%, list=35%, signal=55% |
| 2970 | SMID\_BREAST\_CANCER\_LUMINAL\_B\_DN |  | 478 | 0.30 | 0.68 | 1.000 | 1.000 | 1.000 | 2890 | tags=13%, list=18%, signal=15% |
| 2971 | CERVERA\_SDHB\_TARGETS\_2 |  | 100 | 0.31 | 0.68 | 0.985 | 1.000 | 1.000 | 2187 | tags=11%, list=14%, signal=13% |
| 2972 | REACTOME\_IL\_RECEPTOR\_SHC\_SIGNALING |  | 27 | 0.33 | 0.68 | 0.937 | 1.000 | 1.000 | 6055 | tags=44%, list=38%, signal=72% |
| 2973 | FUJII\_YBX1\_TARGETS\_UP |  | 36 | 0.33 | 0.68 | 0.934 | 1.000 | 1.000 | 2384 | tags=17%, list=15%, signal=20% |
| 2974 | BIDUS\_METASTASIS\_DN |  | 124 | 0.31 | 0.68 | 0.996 | 1.000 | 1.000 | 4550 | tags=35%, list=29%, signal=50% |
| 2975 | TAKEDA\_TARGETS\_OF\_NUP98\_HOXA9\_FUSION\_8D\_UP |  | 134 | 0.31 | 0.68 | 0.991 | 1.000 | 1.000 | 3036 | tags=16%, list=19%, signal=20% |
| 2976 | CERVERA\_SDHB\_TARGETS\_1\_UP |  | 91 | 0.31 | 0.68 | 0.981 | 1.000 | 1.000 | 3036 | tags=16%, list=19%, signal=20% |
| 2977 | ST\_GAQ\_PATHWAY |  | 26 | 0.34 | 0.68 | 0.922 | 1.000 | 1.000 | 5362 | tags=50%, list=34%, signal=76% |
| 2978 | PID\_ENDOTHELINPATHWAY |  | 61 | 0.31 | 0.68 | 0.964 | 1.000 | 1.000 | 4482 | tags=26%, list=28%, signal=37% |
| 2979 | ONGUSAHA\_TP53\_TARGETS |  | 36 | 0.33 | 0.68 | 0.936 | 1.000 | 1.000 | 4063 | tags=36%, list=26%, signal=49% |
| 2980 | PID\_IL8CXCR1\_PATHWAY |  | 23 | 0.34 | 0.68 | 0.917 | 1.000 | 1.000 | 4098 | tags=30%, list=26%, signal=41% |
| 2981 | SIG\_CD40PATHWAYMAP |  | 33 | 0.33 | 0.68 | 0.929 | 1.000 | 1.000 | 5018 | tags=55%, list=32%, signal=80% |
| 2982 | MARIADASON\_REGULATED\_BY\_HISTONE\_ACETYLATION\_UP |  | 68 | 0.31 | 0.68 | 0.967 | 1.000 | 1.000 | 3763 | tags=24%, list=24%, signal=31% |
| 2983 | BIOCARTA\_BARRESTIN\_SRC\_PATHWAY |  | 15 | 0.36 | 0.67 | 0.912 | 1.000 | 1.000 | 5595 | tags=47%, list=36%, signal=72% |
| 2984 | FARMER\_BREAST\_CANCER\_CLUSTER\_7 |  | 18 | 0.35 | 0.67 | 0.913 | 1.000 | 1.000 | 3335 | tags=28%, list=21%, signal=35% |
| 2985 | RAY\_TUMORIGENESIS\_BY\_ERBB2\_CDC25A\_DN |  | 141 | 0.30 | 0.67 | 0.991 | 1.000 | 1.000 | 3216 | tags=21%, list=20%, signal=26% |
| 2986 | VARELA\_ZMPSTE24\_TARGETS\_DN |  | 35 | 0.33 | 0.67 | 0.933 | 1.000 | 1.000 | 4040 | tags=23%, list=26%, signal=31% |
| 2987 | KASLER\_HDAC7\_TARGETS\_1\_DN |  | 17 | 0.35 | 0.67 | 0.922 | 1.000 | 1.000 | 3058 | tags=24%, list=19%, signal=29% |
| 2988 | GESERICK\_TERT\_TARGETS\_DN |  | 20 | 0.34 | 0.67 | 0.932 | 1.000 | 1.000 | 5013 | tags=35%, list=32%, signal=51% |
| 2989 | PID\_LYSOPHOSPHOLIPID\_PATHWAY |  | 61 | 0.31 | 0.67 | 0.963 | 1.000 | 1.000 | 4482 | tags=28%, list=28%, signal=39% |
| 2990 | ONO\_AML1\_TARGETS\_DN |  | 37 | 0.32 | 0.67 | 0.942 | 1.000 | 1.000 | 3406 | tags=14%, list=22%, signal=17% |
| 2991 | KEGG\_STARCH\_AND\_SUCROSE\_METABOLISM |  | 40 | 0.32 | 0.67 | 0.947 | 1.000 | 1.000 | 4721 | tags=25%, list=30%, signal=36% |
| 2992 | WESTON\_VEGFA\_TARGETS\_3HR |  | 69 | 0.31 | 0.67 | 0.972 | 1.000 | 1.000 | 1753 | tags=17%, list=11%, signal=19% |
| 2993 | PIONTEK\_PKD1\_TARGETS\_UP |  | 33 | 0.33 | 0.67 | 0.946 | 1.000 | 1.000 | 3507 | tags=18%, list=22%, signal=23% |
[truncated: 25,900 more chars]
