# Supplementary material for: Immunomic, genomic and transcriptomic characterization of CT26 colorectal carcinoma
Source: BMC Genomics. 2014 Mar 13;15(1):190. doi: 10.1186/1471-2164-15-190 (PMC4007559; doi:10.1186/1471-2164-15-190)
Supplement: Supplementary file 8 — Additional file 8: Contains the Gene Pattern gene set membership and enrichment values in an html format. The file index.html is the entry point. (ZIP 13 MB) [file 12864_2013_7028_MOESM8_ESM.zip › index.html]

Index for xtools.gsea.GseaPreranked .

### GSEA Report for Dataset CT26\_gene\_expression

#### Enrichment in phenotype: **na**

- 3186 / 3660 gene sets are upregulated in phenotype **na\_pos**- 974 gene sets are significant at FDR < 25%- 634 gene sets are significantly enriched at nominal pvalue < 1%- 896 gene sets are significantly enriched at nominal pvalue < 5%- Snapshot of enrichment results- Detailed enrichment results in html format- Detailed enrichment results in excel format (tab delimited text)- Guide to interpret results

#### Enrichment in phenotype: **na**

- 474 / 3660 gene sets are upregulated in phenotype **na\_neg**- 241 gene sets are significantly enriched at FDR < 25%- 144 gene sets are significantly enriched at nominal pvalue < 1%- 172 gene sets are significantly enriched at nominal pvalue < 5%- Snapshot of enrichment results- Detailed enrichment results in html format- Detailed enrichment results in excel format (tab delimited text)- Guide to interpret results

#### Dataset details

- The dataset has 15750 features (genes)- No probe set => gene symbol collapsing was requested, so all 15750 features were used

#### Gene set details

- Gene set size filters (min=15, max=500) resulted in filtering out 1062 / 4722 gene sets- The remaining 3660 gene sets were used in the analysis- List of gene sets used and their sizes (restricted to features in the specified dataset)

#### Gene markers for the **na\_pos** *versus* **na\_neg** comparison

- The dataset has 15750 features (genes)- Detailed rank ordered gene list for all features in the dataset

#### Global statistics and plots

- Plot of p-values *vs.* NES- Global ES histogram

#### Other

- Parameters used for this analysis

---

Report: my\_analysis.GseaPreranked.1374178656685.rpt   by user: gpprod

xtools.gsea.GseaPreranked [Thu, Jul 18, '13 4 PM 17]

Website: www.broadinstitute.org/GSEA
Questions & Suggestions: Email
