# Supplementary figures and images for: Immunomic, genomic and transcriptomic characterization of CT26 colorectal carcinoma
Source: BMC Genomics. 2014 Mar 13;15(1):190. doi: 10.1186/1471-2164-15-190 (PMC4007559; doi:10.1186/1471-2164-15-190)

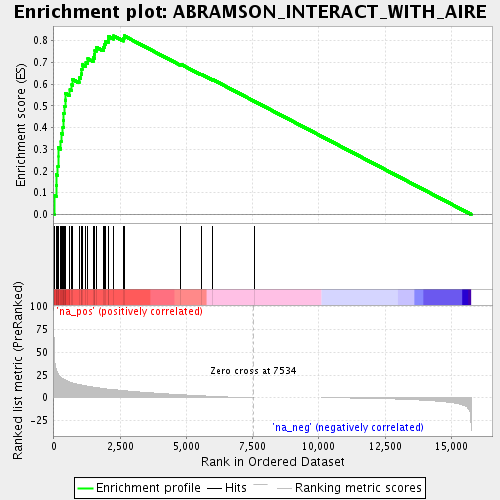

Supplement: Supplementary file 8 — Additional file 8: Contains the Gene Pattern gene set membership and enrichment values in an html format. The file index.html is the entry point. (ZIP 13 MB) [file 12864_2013_7028_MOESM8_ESM.zip › enplot_ABRAMSON_INTERACT_WITH_AIRE_9.png]

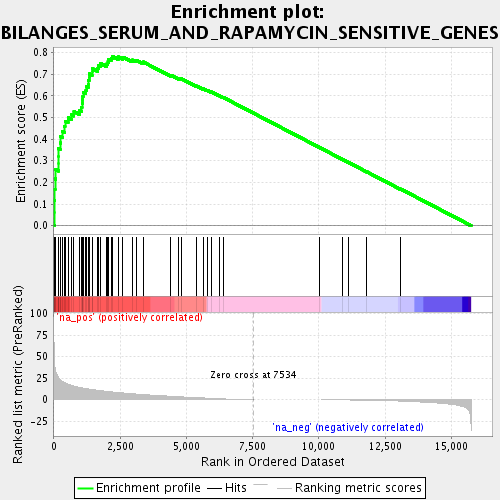

Supplement: Supplementary file 8 — Additional file 8: Contains the Gene Pattern gene set membership and enrichment values in an html format. The file index.html is the entry point. (ZIP 13 MB) [file 12864_2013_7028_MOESM8_ESM.zip › enplot_BILANGES_SERUM_AND_RAPAMYCIN_SENSITIVE_GENES_37.png]

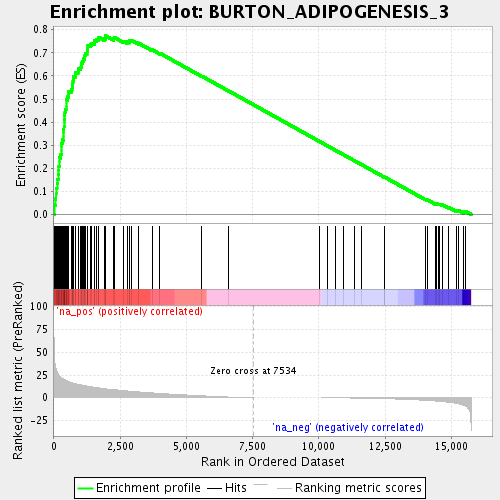

Supplement: Supplementary file 8 — Additional file 8: Contains the Gene Pattern gene set membership and enrichment values in an html format. The file index.html is the entry point. (ZIP 13 MB) [file 12864_2013_7028_MOESM8_ESM.zip › enplot_BURTON_ADIPOGENESIS_3_39.png]

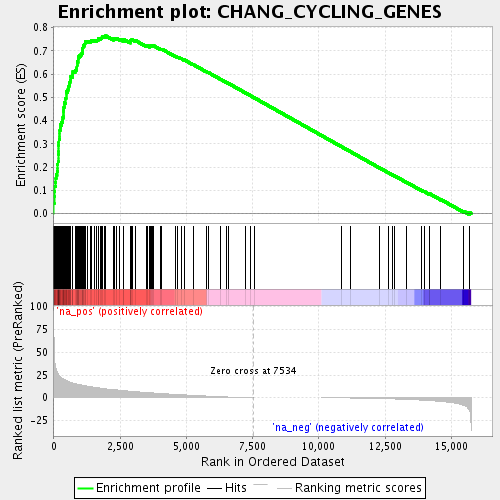

Supplement: Supplementary file 8 — Additional file 8: Contains the Gene Pattern gene set membership and enrichment values in an html format. The file index.html is the entry point. (ZIP 13 MB) [file 12864_2013_7028_MOESM8_ESM.zip › enplot_CHANG_CYCLING_GENES_27.png]

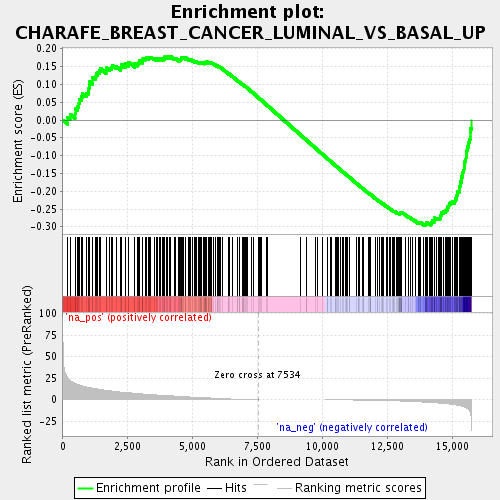

Supplement: Supplementary file 8 — Additional file 8: Contains the Gene Pattern gene set membership and enrichment values in an html format. The file index.html is the entry point. (ZIP 13 MB) [file 12864_2013_7028_MOESM8_ESM.zip › enplot_CHARAFE_BREAST_CANCER_LUMINAL_VS_BASAL_UP_71.png]

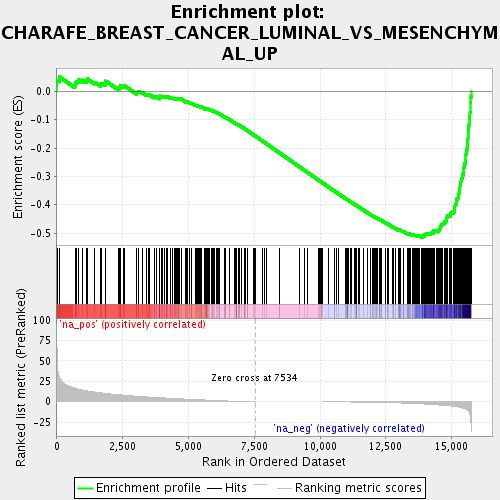

Supplement: Supplementary file 8 — Additional file 8: Contains the Gene Pattern gene set membership and enrichment values in an html format. The file index.html is the entry point. (ZIP 13 MB) [file 12864_2013_7028_MOESM8_ESM.zip › enplot_CHARAFE_BREAST_CANCER_LUMINAL_VS_MESENCHYMAL_UP_73.png]

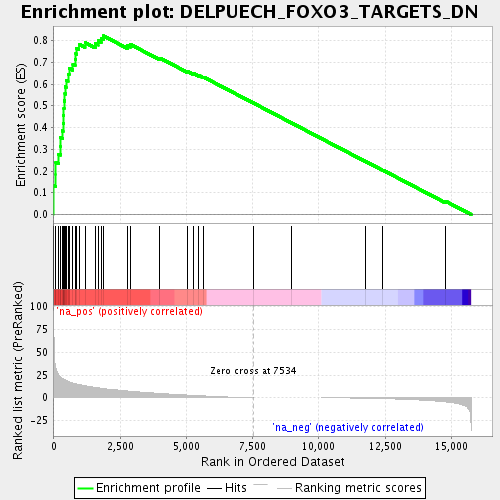

Supplement: Supplementary file 8 — Additional file 8: Contains the Gene Pattern gene set membership and enrichment values in an html format. The file index.html is the entry point. (ZIP 13 MB) [file 12864_2013_7028_MOESM8_ESM.zip › enplot_DELPUECH_FOXO3_TARGETS_DN_13.png]

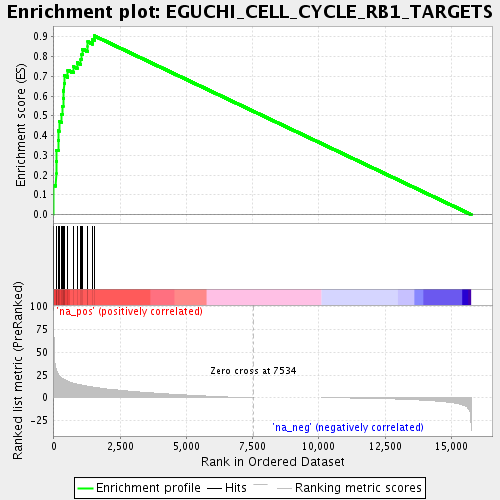

Supplement: Supplementary file 8 — Additional file 8: Contains the Gene Pattern gene set membership and enrichment values in an html format. The file index.html is the entry point. (ZIP 13 MB) [file 12864_2013_7028_MOESM8_ESM.zip › enplot_EGUCHI_CELL_CYCLE_RB1_TARGETS_1.png]

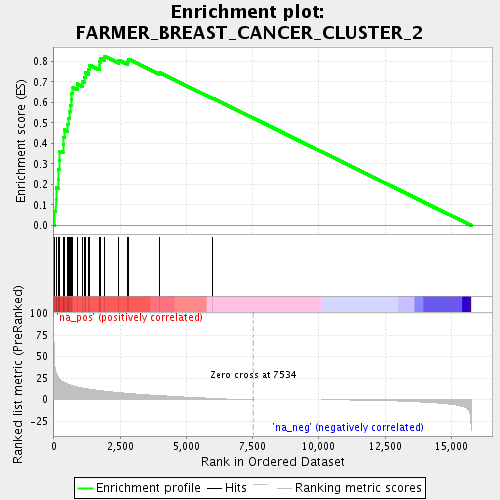

Supplement: Supplementary file 8 — Additional file 8: Contains the Gene Pattern gene set membership and enrichment values in an html format. The file index.html is the entry point. (ZIP 13 MB) [file 12864_2013_7028_MOESM8_ESM.zip › enplot_FARMER_BREAST_CANCER_CLUSTER_2_25.png]

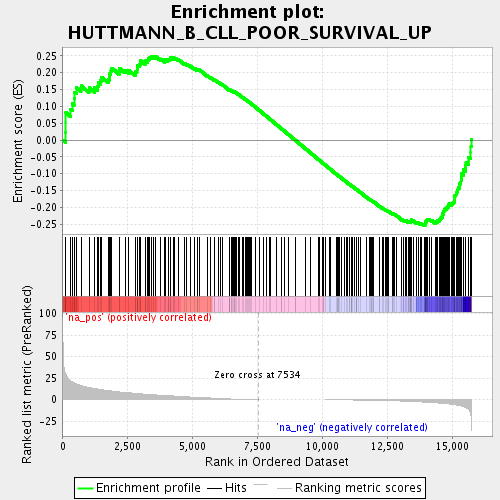

Supplement: Supplementary file 8 — Additional file 8: Contains the Gene Pattern gene set membership and enrichment values in an html format. The file index.html is the entry point. (ZIP 13 MB) [file 12864_2013_7028_MOESM8_ESM.zip › enplot_HUTTMANN_B_CLL_POOR_SURVIVAL_UP_69.png]

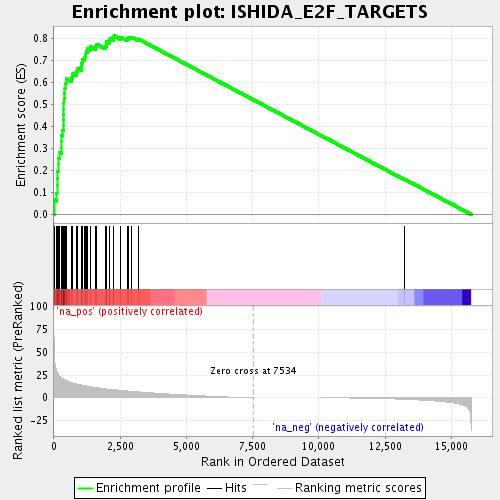

Supplement: Supplementary file 8 — Additional file 8: Contains the Gene Pattern gene set membership and enrichment values in an html format. The file index.html is the entry point. (ZIP 13 MB) [file 12864_2013_7028_MOESM8_ESM.zip › enplot_ISHIDA_E2F_TARGETS_15.png]

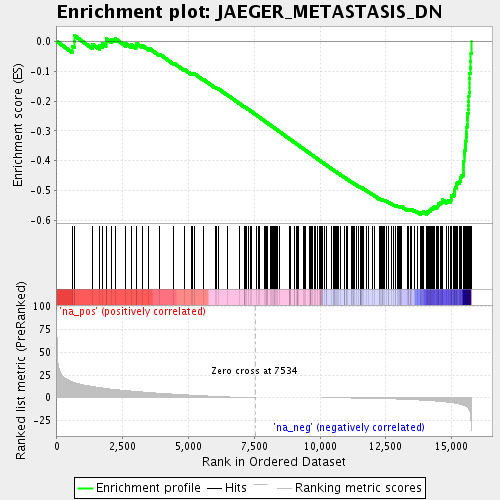

Supplement: Supplementary file 8 — Additional file 8: Contains the Gene Pattern gene set membership and enrichment values in an html format. The file index.html is the entry point. (ZIP 13 MB) [file 12864_2013_7028_MOESM8_ESM.zip › enplot_JAEGER_METASTASIS_DN_79.png]

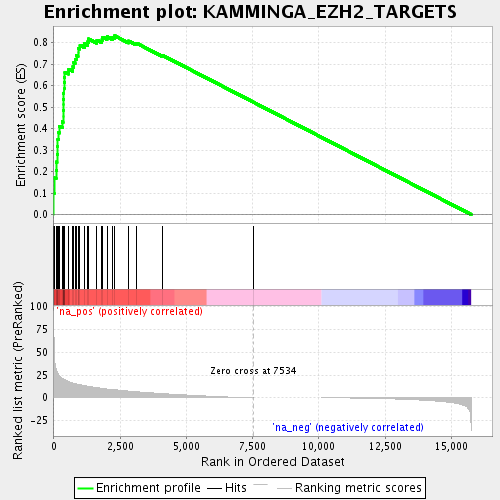

Supplement: Supplementary file 8 — Additional file 8: Contains the Gene Pattern gene set membership and enrichment values in an html format. The file index.html is the entry point. (ZIP 13 MB) [file 12864_2013_7028_MOESM8_ESM.zip › enplot_KAMMINGA_EZH2_TARGETS_5.png]

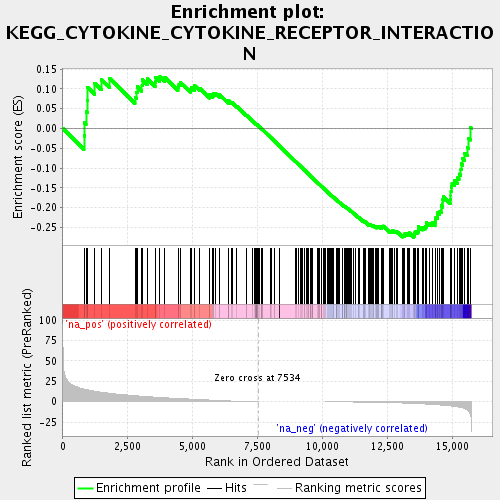

Supplement: Supplementary file 8 — Additional file 8: Contains the Gene Pattern gene set membership and enrichment values in an html format. The file index.html is the entry point. (ZIP 13 MB) [file 12864_2013_7028_MOESM8_ESM.zip › enplot_KEGG_CYTOKINE_CYTOKINE_RECEPTOR_INTERACTION_41.png]

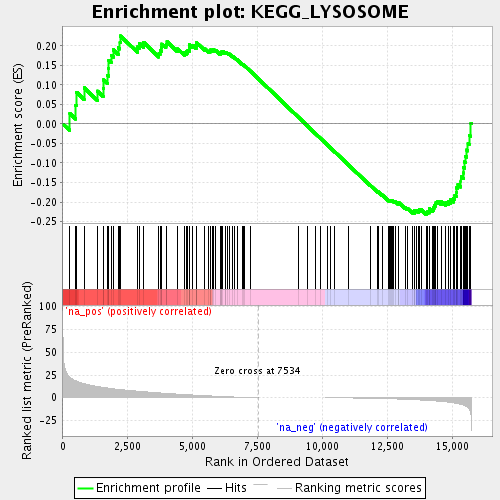

Supplement: Supplementary file 8 — Additional file 8: Contains the Gene Pattern gene set membership and enrichment values in an html format. The file index.html is the entry point. (ZIP 13 MB) [file 12864_2013_7028_MOESM8_ESM.zip › enplot_KEGG_LYSOSOME_45.png]

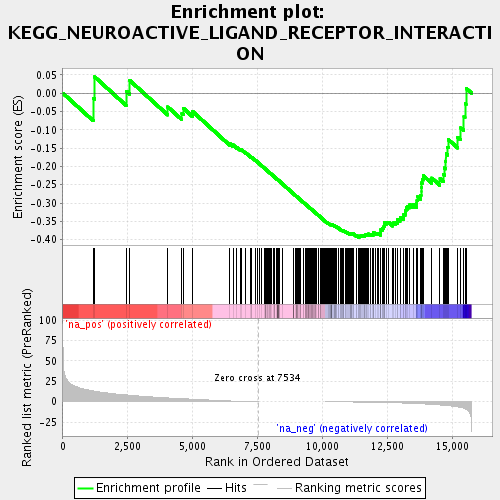

Supplement: Supplementary file 8 — Additional file 8: Contains the Gene Pattern gene set membership and enrichment values in an html format. The file index.html is the entry point. (ZIP 13 MB) [file 12864_2013_7028_MOESM8_ESM.zip › enplot_KEGG_NEUROACTIVE_LIGAND_RECEPTOR_INTERACTION_43.png]

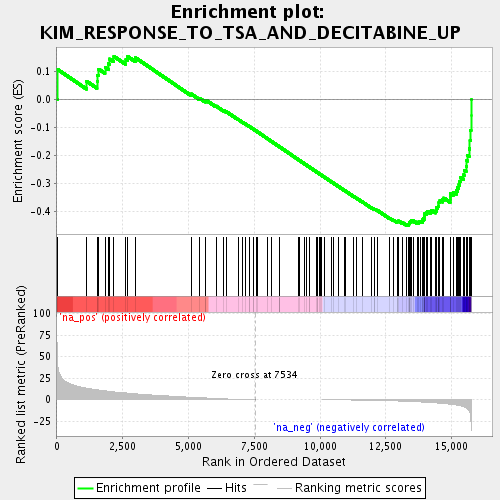

Supplement: Supplementary file 8 — Additional file 8: Contains the Gene Pattern gene set membership and enrichment values in an html format. The file index.html is the entry point. (ZIP 13 MB) [file 12864_2013_7028_MOESM8_ESM.zip › enplot_KIM_RESPONSE_TO_TSA_AND_DECITABINE_UP_63.png]

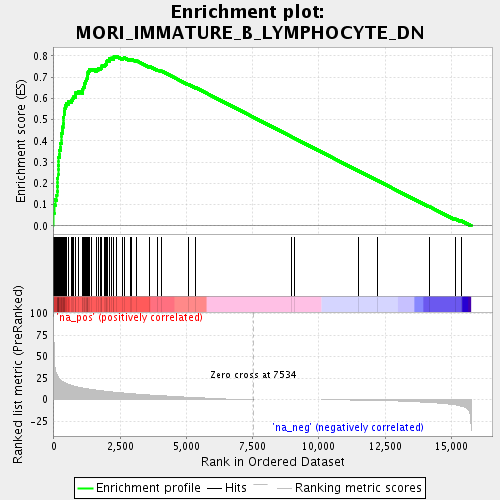

Supplement: Supplementary file 8 — Additional file 8: Contains the Gene Pattern gene set membership and enrichment values in an html format. The file index.html is the entry point. (ZIP 13 MB) [file 12864_2013_7028_MOESM8_ESM.zip › enplot_MORI_IMMATURE_B_LYMPHOCYTE_DN_7.png]

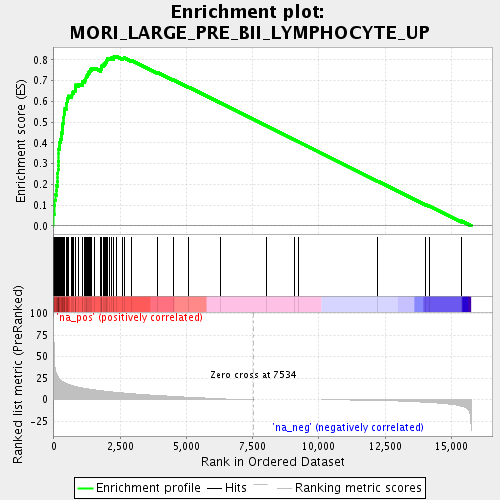

Supplement: Supplementary file 8 — Additional file 8: Contains the Gene Pattern gene set membership and enrichment values in an html format. The file index.html is the entry point. (ZIP 13 MB) [file 12864_2013_7028_MOESM8_ESM.zip › enplot_MORI_LARGE_PRE_BII_LYMPHOCYTE_UP_3.png]

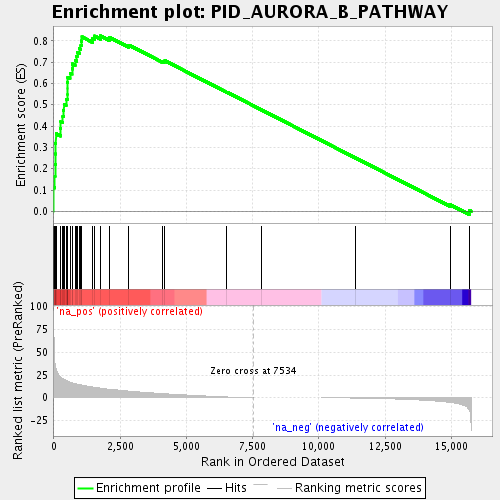

Supplement: Supplementary file 8 — Additional file 8: Contains the Gene Pattern gene set membership and enrichment values in an html format. The file index.html is the entry point. (ZIP 13 MB) [file 12864_2013_7028_MOESM8_ESM.zip › enplot_PID_AURORA_B_PATHWAY_19.png]

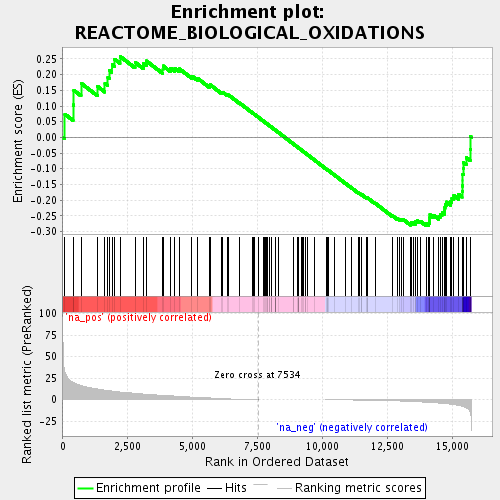

Supplement: Supplementary file 8 — Additional file 8: Contains the Gene Pattern gene set membership and enrichment values in an html format. The file index.html is the entry point. (ZIP 13 MB) [file 12864_2013_7028_MOESM8_ESM.zip › enplot_REACTOME_BIOLOGICAL_OXIDATIONS_49.png]

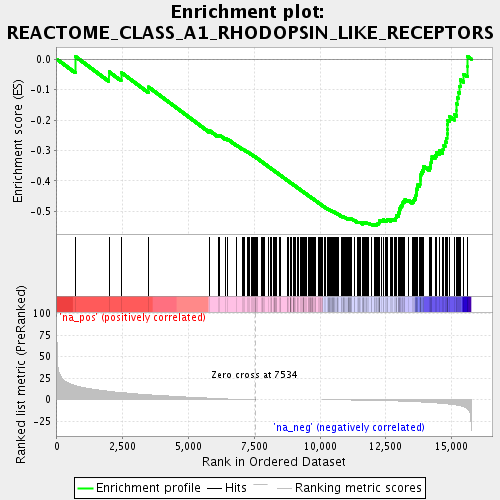

Supplement: Supplementary file 8 — Additional file 8: Contains the Gene Pattern gene set membership and enrichment values in an html format. The file index.html is the entry point. (ZIP 13 MB) [file 12864_2013_7028_MOESM8_ESM.zip › enplot_REACTOME_CLASS_A1_RHODOPSIN_LIKE_RECEPTORS_53.png]

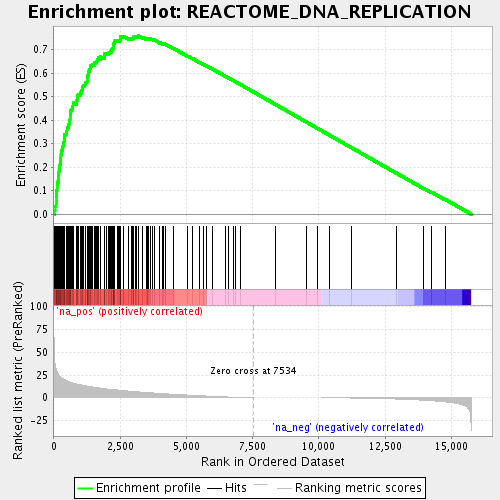

Supplement: Supplementary file 8 — Additional file 8: Contains the Gene Pattern gene set membership and enrichment values in an html format. The file index.html is the entry point. (ZIP 13 MB) [file 12864_2013_7028_MOESM8_ESM.zip › enplot_REACTOME_DNA_REPLICATION_31.png]

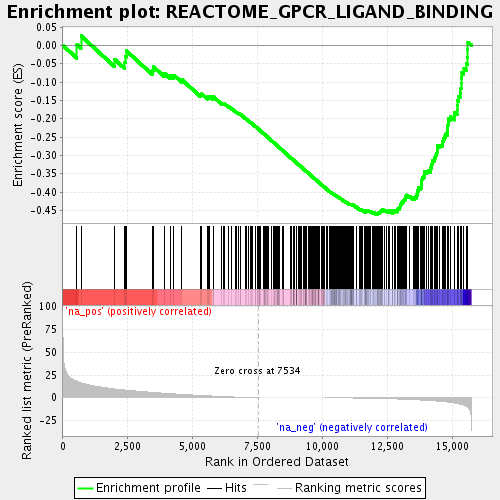

Supplement: Supplementary file 8 — Additional file 8: Contains the Gene Pattern gene set membership and enrichment values in an html format. The file index.html is the entry point. (ZIP 13 MB) [file 12864_2013_7028_MOESM8_ESM.zip › enplot_REACTOME_GPCR_LIGAND_BINDING_61.png]

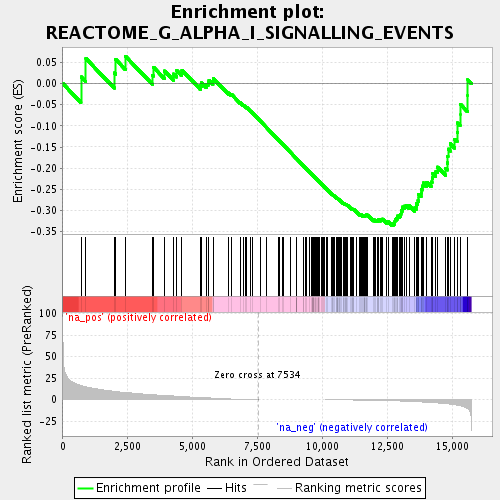

Supplement: Supplementary file 8 — Additional file 8: Contains the Gene Pattern gene set membership and enrichment values in an html format. The file index.html is the entry point. (ZIP 13 MB) [file 12864_2013_7028_MOESM8_ESM.zip › enplot_REACTOME_G_ALPHA_I_SIGNALLING_EVENTS_57.png]

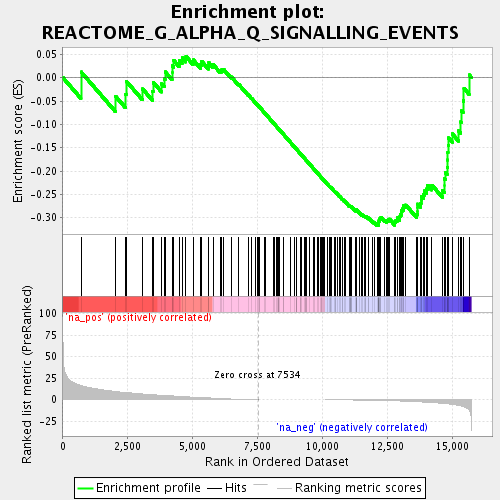

Supplement: Supplementary file 8 — Additional file 8: Contains the Gene Pattern gene set membership and enrichment values in an html format. The file index.html is the entry point. (ZIP 13 MB) [file 12864_2013_7028_MOESM8_ESM.zip › enplot_REACTOME_G_ALPHA_Q_SIGNALLING_EVENTS_55.png]

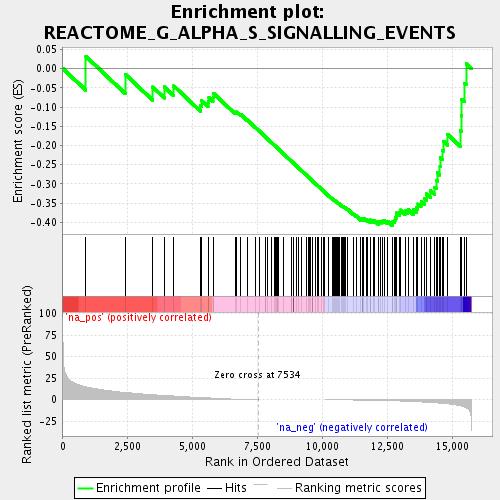

Supplement: Supplementary file 8 — Additional file 8: Contains the Gene Pattern gene set membership and enrichment values in an html format. The file index.html is the entry point. (ZIP 13 MB) [file 12864_2013_7028_MOESM8_ESM.zip › enplot_REACTOME_G_ALPHA_S_SIGNALLING_EVENTS_59.png]

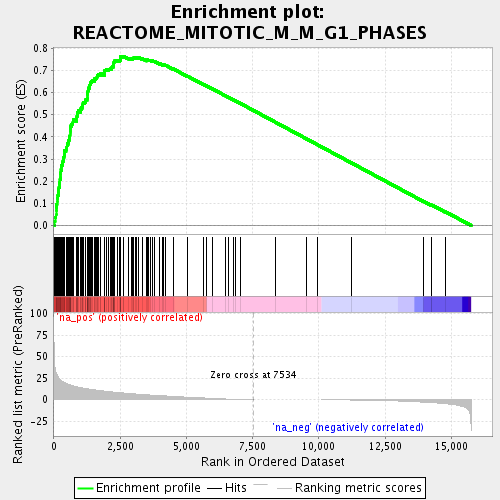

Supplement: Supplementary file 8 — Additional file 8: Contains the Gene Pattern gene set membership and enrichment values in an html format. The file index.html is the entry point. (ZIP 13 MB) [file 12864_2013_7028_MOESM8_ESM.zip › enplot_REACTOME_MITOTIC_M_M_G1_PHASES_29.png]

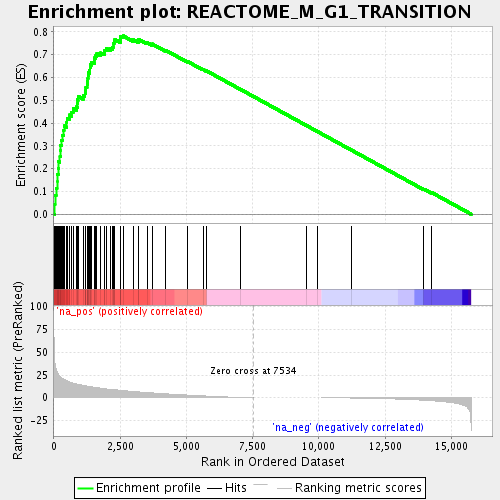

Supplement: Supplementary file 8 — Additional file 8: Contains the Gene Pattern gene set membership and enrichment values in an html format. The file index.html is the entry point. (ZIP 13 MB) [file 12864_2013_7028_MOESM8_ESM.zip › enplot_REACTOME_M_G1_TRANSITION_17.png]

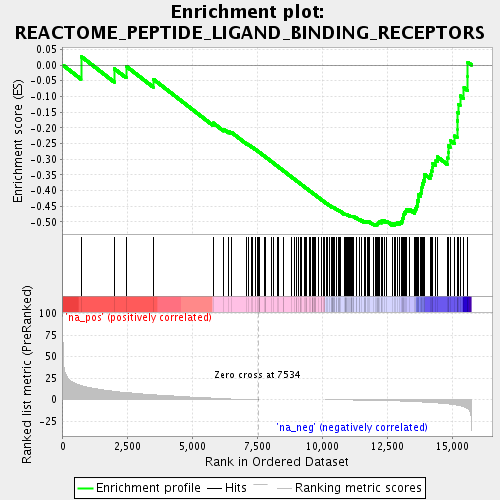

Supplement: Supplementary file 8 — Additional file 8: Contains the Gene Pattern gene set membership and enrichment values in an html format. The file index.html is the entry point. (ZIP 13 MB) [file 12864_2013_7028_MOESM8_ESM.zip › enplot_REACTOME_PEPTIDE_LIGAND_BINDING_RECEPTORS_51.png]

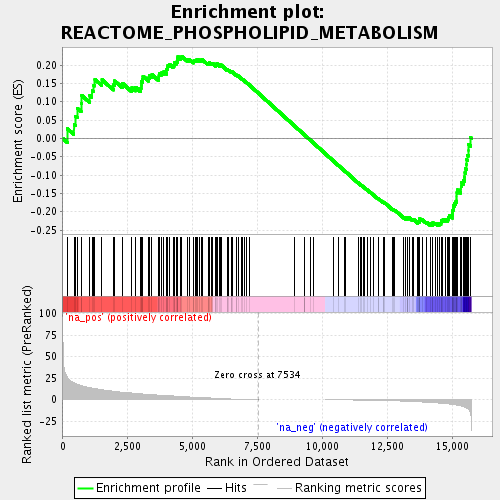

Supplement: Supplementary file 8 — Additional file 8: Contains the Gene Pattern gene set membership and enrichment values in an html format. The file index.html is the entry point. (ZIP 13 MB) [file 12864_2013_7028_MOESM8_ESM.zip › enplot_REACTOME_PHOSPHOLIPID_METABOLISM_47.png]

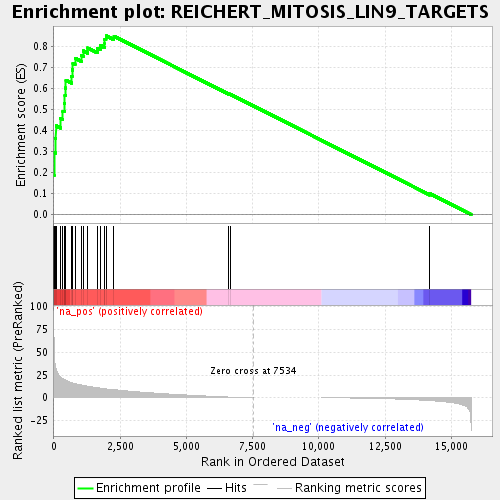

Supplement: Supplementary file 8 — Additional file 8: Contains the Gene Pattern gene set membership and enrichment values in an html format. The file index.html is the entry point. (ZIP 13 MB) [file 12864_2013_7028_MOESM8_ESM.zip › enplot_REICHERT_MITOSIS_LIN9_TARGETS_11.png]

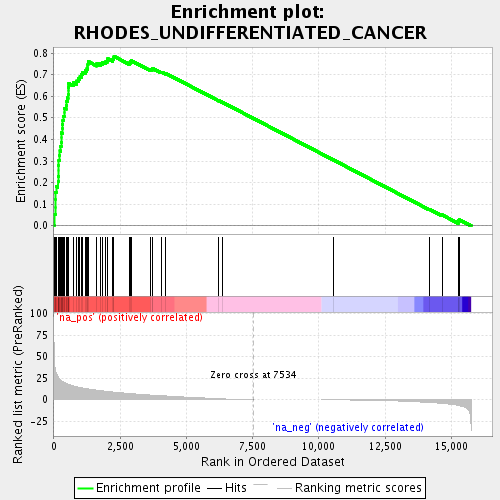

Supplement: Supplementary file 8 — Additional file 8: Contains the Gene Pattern gene set membership and enrichment values in an html format. The file index.html is the entry point. (ZIP 13 MB) [file 12864_2013_7028_MOESM8_ESM.zip › enplot_RHODES_UNDIFFERENTIATED_CANCER_23.png]

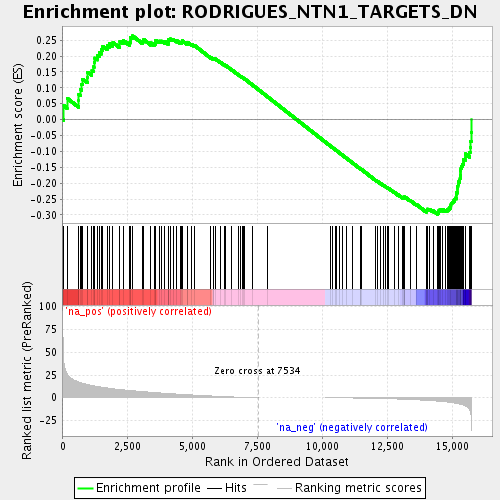

Supplement: Supplementary file 8 — Additional file 8: Contains the Gene Pattern gene set membership and enrichment values in an html format. The file index.html is the entry point. (ZIP 13 MB) [file 12864_2013_7028_MOESM8_ESM.zip › enplot_RODRIGUES_NTN1_TARGETS_DN_75.png]

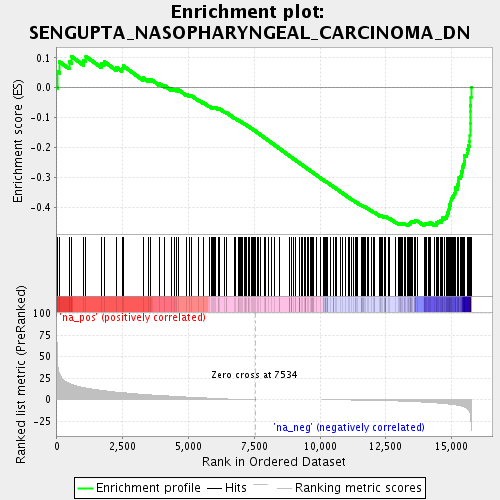

Supplement: Supplementary file 8 — Additional file 8: Contains the Gene Pattern gene set membership and enrichment values in an html format. The file index.html is the entry point. (ZIP 13 MB) [file 12864_2013_7028_MOESM8_ESM.zip › enplot_SENGUPTA_NASOPHARYNGEAL_CARCINOMA_DN_65.png]

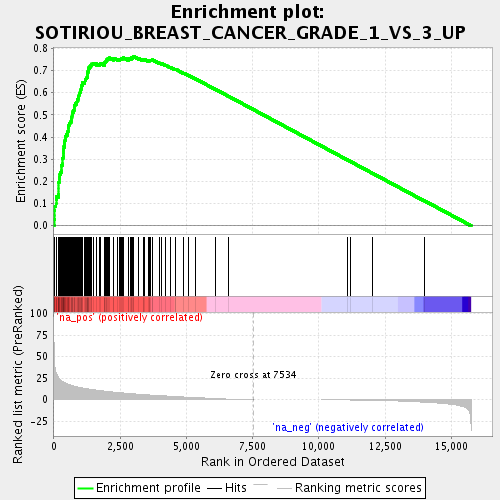

Supplement: Supplementary file 8 — Additional file 8: Contains the Gene Pattern gene set membership and enrichment values in an html format. The file index.html is the entry point. (ZIP 13 MB) [file 12864_2013_7028_MOESM8_ESM.zip › enplot_SOTIRIOU_BREAST_CANCER_GRADE_1_VS_3_UP_33.png]

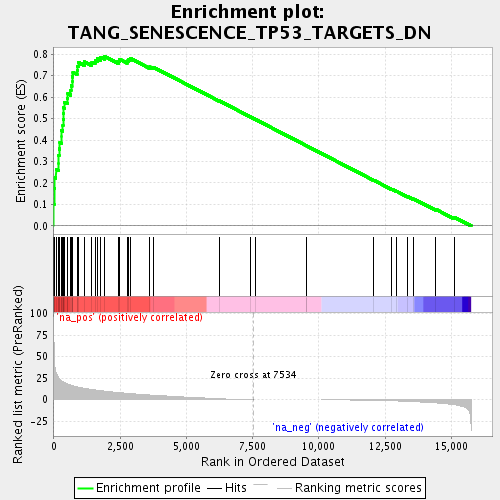

Supplement: Supplementary file 8 — Additional file 8: Contains the Gene Pattern gene set membership and enrichment values in an html format. The file index.html is the entry point. (ZIP 13 MB) [file 12864_2013_7028_MOESM8_ESM.zip › enplot_TANG_SENESCENCE_TP53_TARGETS_DN_35.png]

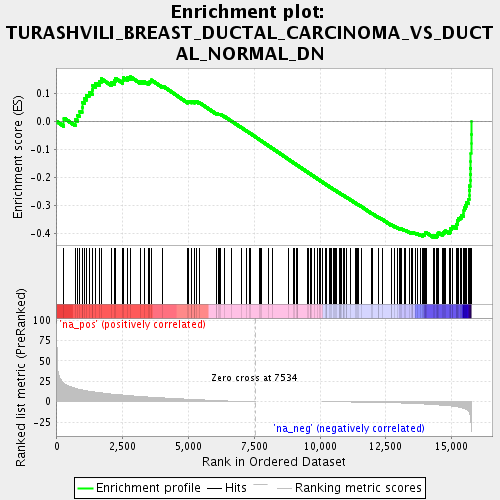

Supplement: Supplementary file 8 — Additional file 8: Contains the Gene Pattern gene set membership and enrichment values in an html format. The file index.html is the entry point. (ZIP 13 MB) [file 12864_2013_7028_MOESM8_ESM.zip › enplot_TURASHVILI_BREAST_DUCTAL_CARCINOMA_VS_DUCTAL_NORMAL_DN_67.png]

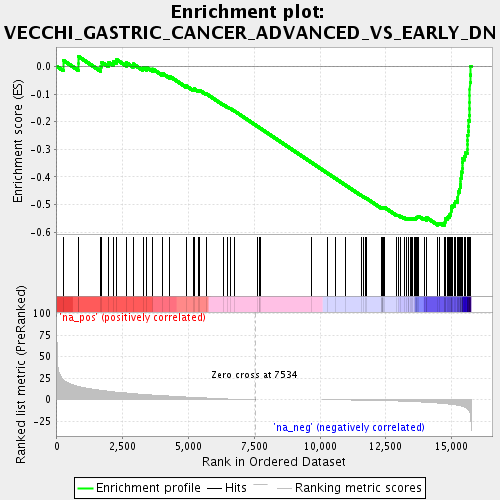

Supplement: Supplementary file 8 — Additional file 8: Contains the Gene Pattern gene set membership and enrichment values in an html format. The file index.html is the entry point. (ZIP 13 MB) [file 12864_2013_7028_MOESM8_ESM.zip › enplot_VECCHI_GASTRIC_CANCER_ADVANCED_VS_EARLY_DN_77.png]

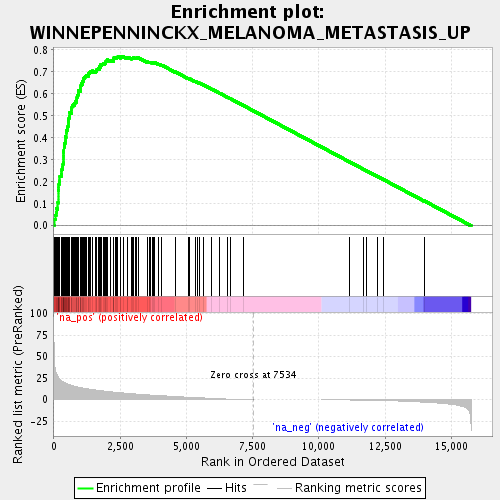

Supplement: Supplementary file 8 — Additional file 8: Contains the Gene Pattern gene set membership and enrichment values in an html format. The file index.html is the entry point. (ZIP 13 MB) [file 12864_2013_7028_MOESM8_ESM.zip › enplot_WINNEPENNINCKX_MELANOMA_METASTASIS_UP_21.png]

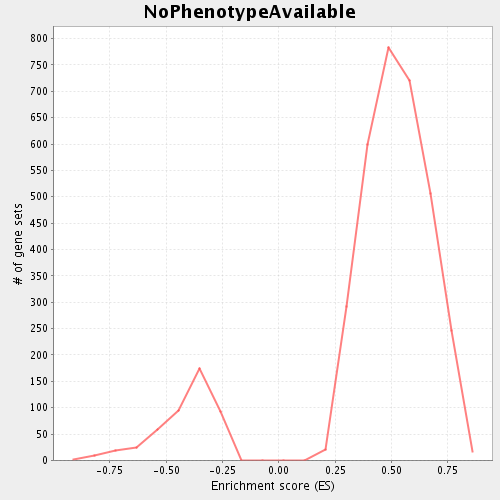

Supplement: Supplementary file 8 — Additional file 8: Contains the Gene Pattern gene set membership and enrichment values in an html format. The file index.html is the entry point. (ZIP 13 MB) [file 12864_2013_7028_MOESM8_ESM.zip › global_es_histogram.png]

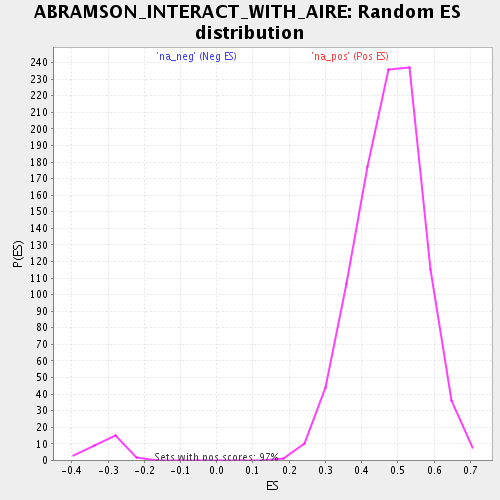

Supplement: Supplementary file 8 — Additional file 8: Contains the Gene Pattern gene set membership and enrichment values in an html format. The file index.html is the entry point. (ZIP 13 MB) [file 12864_2013_7028_MOESM8_ESM.zip › gset_rnd_es_dist_10.png]

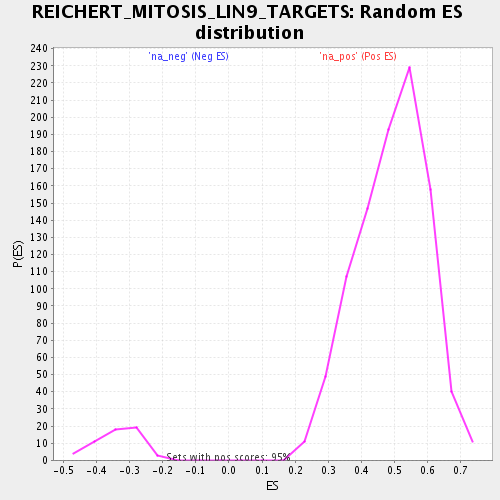

Supplement: Supplementary file 8 — Additional file 8: Contains the Gene Pattern gene set membership and enrichment values in an html format. The file index.html is the entry point. (ZIP 13 MB) [file 12864_2013_7028_MOESM8_ESM.zip › gset_rnd_es_dist_12.png]

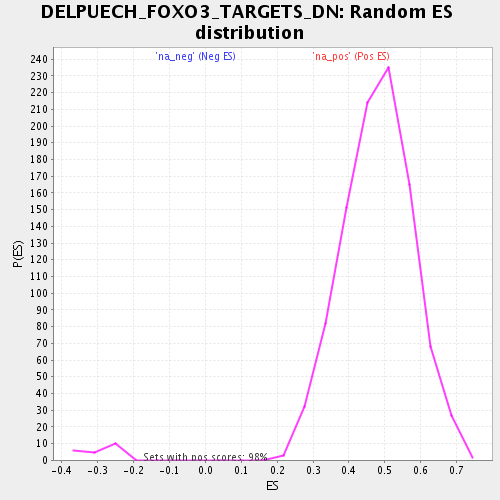

Supplement: Supplementary file 8 — Additional file 8: Contains the Gene Pattern gene set membership and enrichment values in an html format. The file index.html is the entry point. (ZIP 13 MB) [file 12864_2013_7028_MOESM8_ESM.zip › gset_rnd_es_dist_14.png]

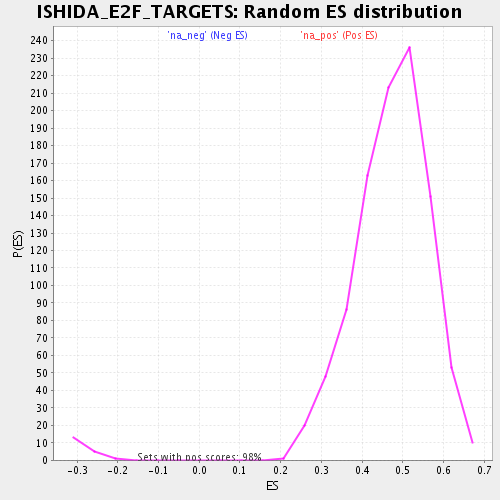

Supplement: Supplementary file 8 — Additional file 8: Contains the Gene Pattern gene set membership and enrichment values in an html format. The file index.html is the entry point. (ZIP 13 MB) [file 12864_2013_7028_MOESM8_ESM.zip › gset_rnd_es_dist_16.png]

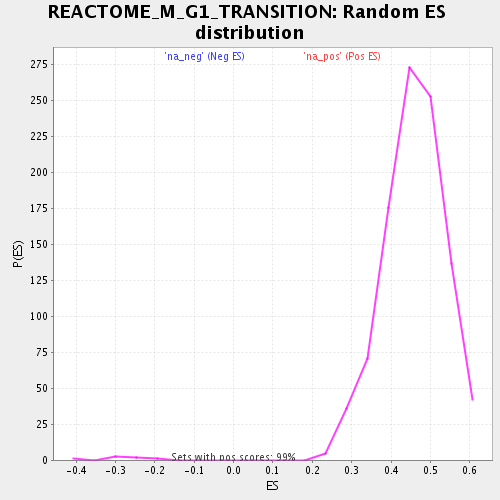

Supplement: Supplementary file 8 — Additional file 8: Contains the Gene Pattern gene set membership and enrichment values in an html format. The file index.html is the entry point. (ZIP 13 MB) [file 12864_2013_7028_MOESM8_ESM.zip › gset_rnd_es_dist_18.png]

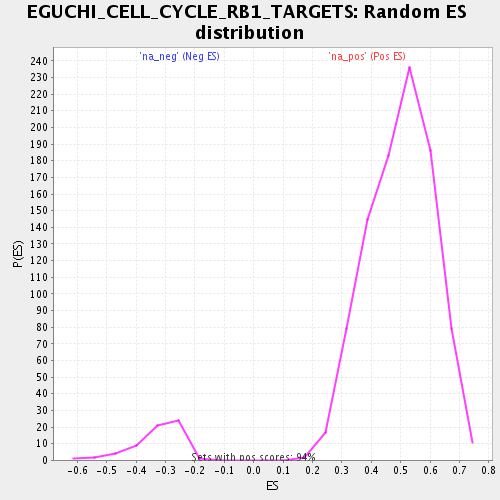

Supplement: Supplementary file 8 — Additional file 8: Contains the Gene Pattern gene set membership and enrichment values in an html format. The file index.html is the entry point. (ZIP 13 MB) [file 12864_2013_7028_MOESM8_ESM.zip › gset_rnd_es_dist_2.png]

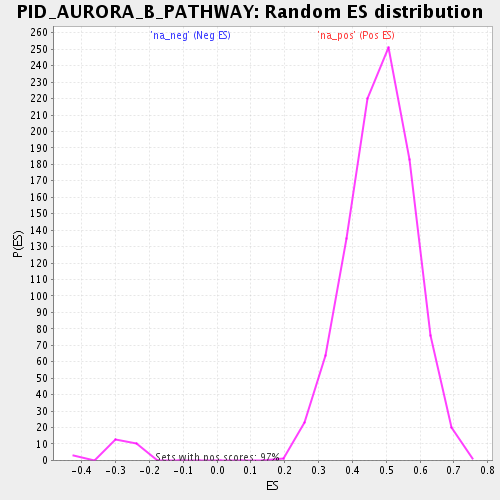

Supplement: Supplementary file 8 — Additional file 8: Contains the Gene Pattern gene set membership and enrichment values in an html format. The file index.html is the entry point. (ZIP 13 MB) [file 12864_2013_7028_MOESM8_ESM.zip › gset_rnd_es_dist_20.png]

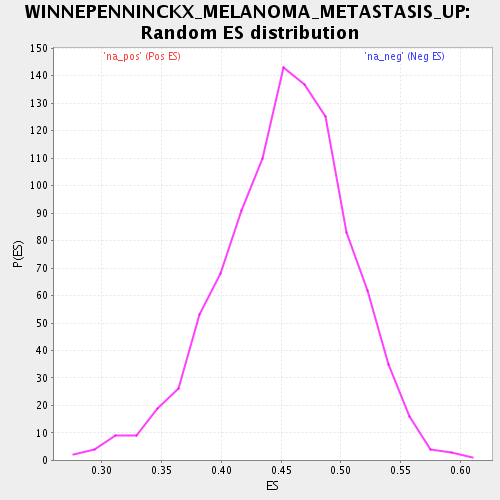

Supplement: Supplementary file 8 — Additional file 8: Contains the Gene Pattern gene set membership and enrichment values in an html format. The file index.html is the entry point. (ZIP 13 MB) [file 12864_2013_7028_MOESM8_ESM.zip › gset_rnd_es_dist_22.png]

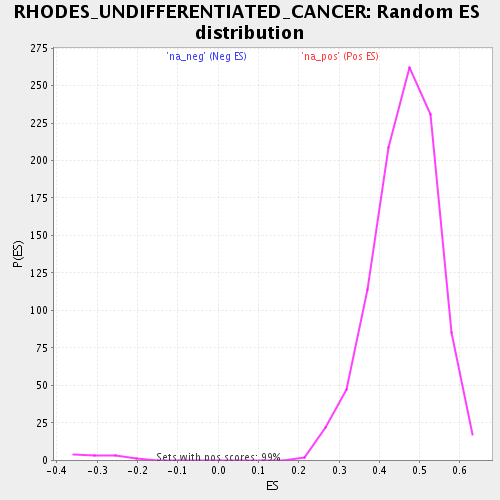

Supplement: Supplementary file 8 — Additional file 8: Contains the Gene Pattern gene set membership and enrichment values in an html format. The file index.html is the entry point. (ZIP 13 MB) [file 12864_2013_7028_MOESM8_ESM.zip › gset_rnd_es_dist_24.png]

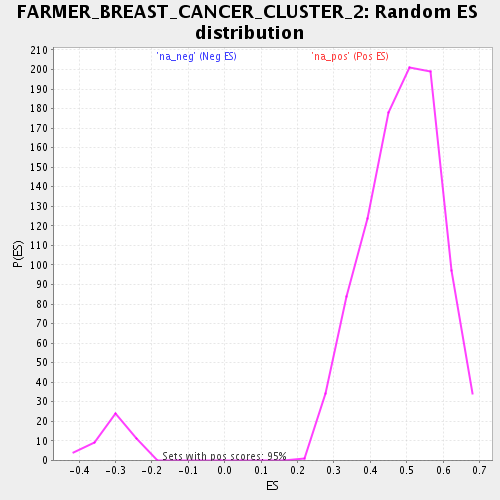

Supplement: Supplementary file 8 — Additional file 8: Contains the Gene Pattern gene set membership and enrichment values in an html format. The file index.html is the entry point. (ZIP 13 MB) [file 12864_2013_7028_MOESM8_ESM.zip › gset_rnd_es_dist_26.png]

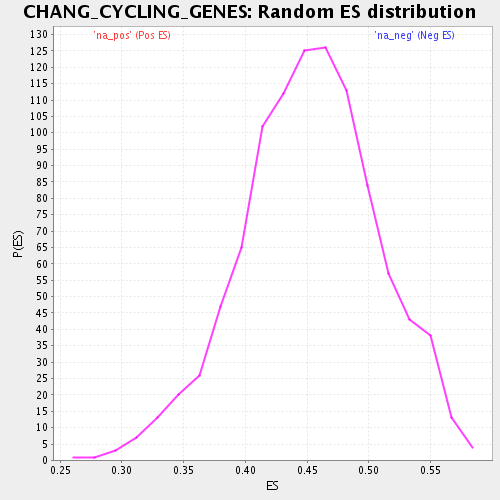

Supplement: Supplementary file 8 — Additional file 8: Contains the Gene Pattern gene set membership and enrichment values in an html format. The file index.html is the entry point. (ZIP 13 MB) [file 12864_2013_7028_MOESM8_ESM.zip › gset_rnd_es_dist_28.png]

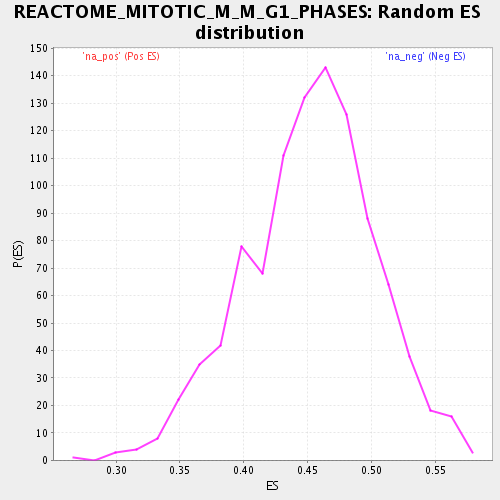

Supplement: Supplementary file 8 — Additional file 8: Contains the Gene Pattern gene set membership and enrichment values in an html format. The file index.html is the entry point. (ZIP 13 MB) [file 12864_2013_7028_MOESM8_ESM.zip › gset_rnd_es_dist_30.png]

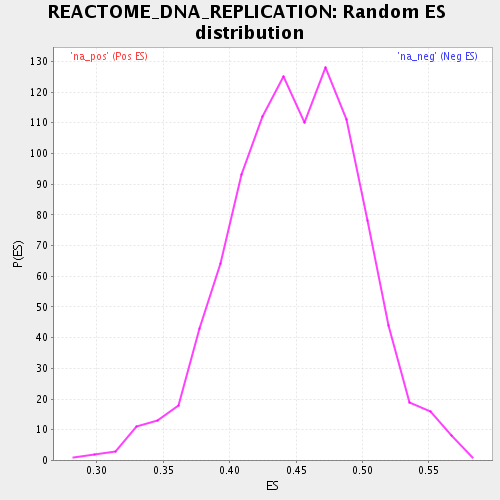

Supplement: Supplementary file 8 — Additional file 8: Contains the Gene Pattern gene set membership and enrichment values in an html format. The file index.html is the entry point. (ZIP 13 MB) [file 12864_2013_7028_MOESM8_ESM.zip › gset_rnd_es_dist_32.png]

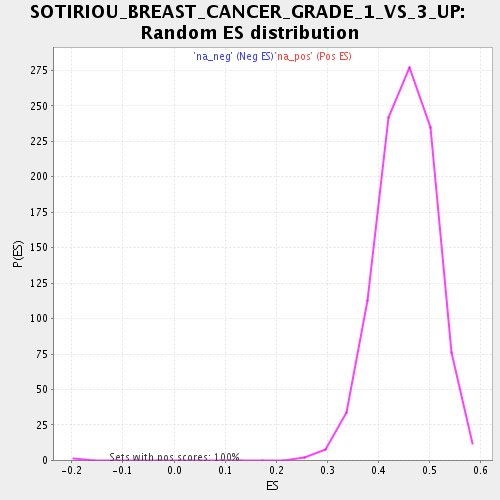

Supplement: Supplementary file 8 — Additional file 8: Contains the Gene Pattern gene set membership and enrichment values in an html format. The file index.html is the entry point. (ZIP 13 MB) [file 12864_2013_7028_MOESM8_ESM.zip › gset_rnd_es_dist_34.png]

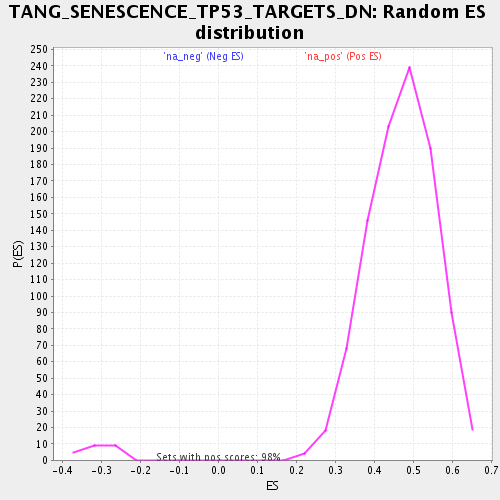

Supplement: Supplementary file 8 — Additional file 8: Contains the Gene Pattern gene set membership and enrichment values in an html format. The file index.html is the entry point. (ZIP 13 MB) [file 12864_2013_7028_MOESM8_ESM.zip › gset_rnd_es_dist_36.png]

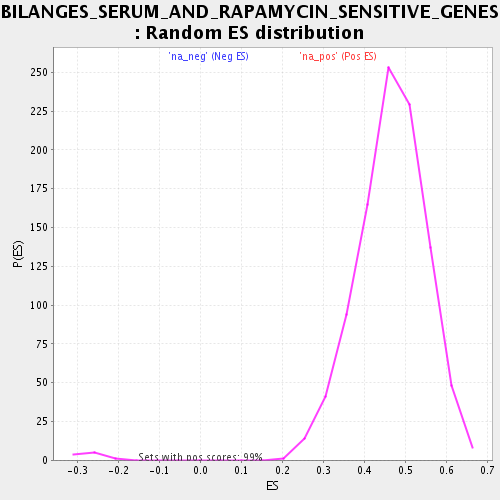

Supplement: Supplementary file 8 — Additional file 8: Contains the Gene Pattern gene set membership and enrichment values in an html format. The file index.html is the entry point. (ZIP 13 MB) [file 12864_2013_7028_MOESM8_ESM.zip › gset_rnd_es_dist_38.png]

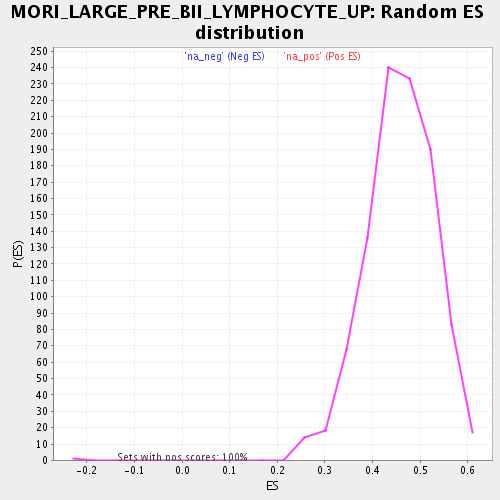

Supplement: Supplementary file 8 — Additional file 8: Contains the Gene Pattern gene set membership and enrichment values in an html format. The file index.html is the entry point. (ZIP 13 MB) [file 12864_2013_7028_MOESM8_ESM.zip › gset_rnd_es_dist_4.png]

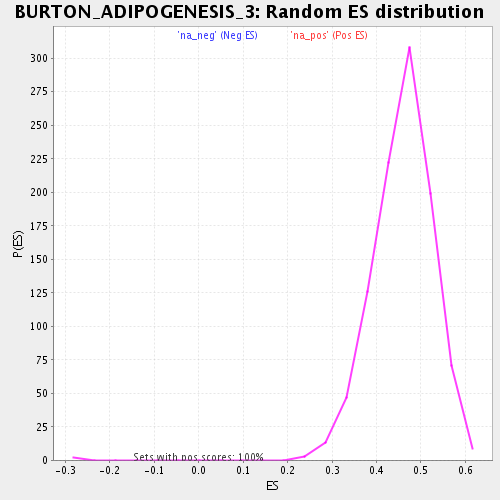

Supplement: Supplementary file 8 — Additional file 8: Contains the Gene Pattern gene set membership and enrichment values in an html format. The file index.html is the entry point. (ZIP 13 MB) [file 12864_2013_7028_MOESM8_ESM.zip › gset_rnd_es_dist_40.png]

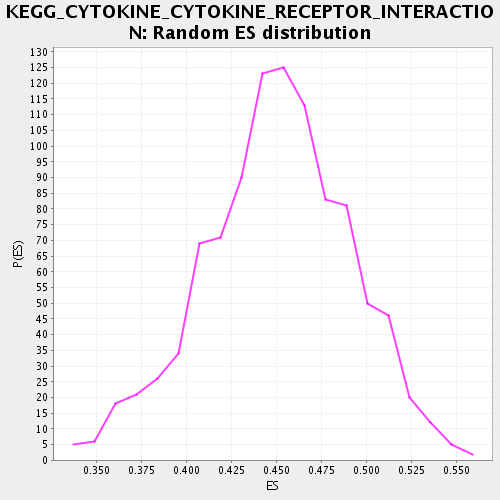

Supplement: Supplementary file 8 — Additional file 8: Contains the Gene Pattern gene set membership and enrichment values in an html format. The file index.html is the entry point. (ZIP 13 MB) [file 12864_2013_7028_MOESM8_ESM.zip › gset_rnd_es_dist_42.png]

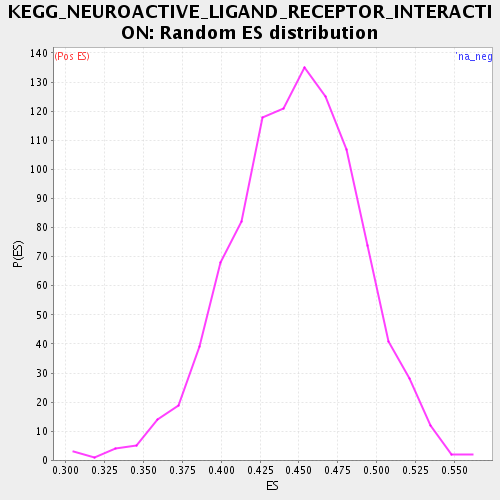

Supplement: Supplementary file 8 — Additional file 8: Contains the Gene Pattern gene set membership and enrichment values in an html format. The file index.html is the entry point. (ZIP 13 MB) [file 12864_2013_7028_MOESM8_ESM.zip › gset_rnd_es_dist_44.png]

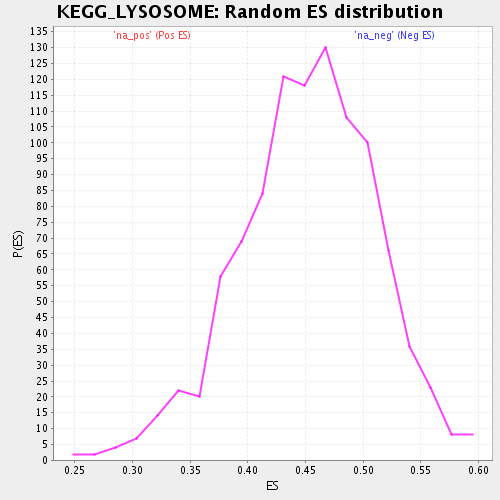

Supplement: Supplementary file 8 — Additional file 8: Contains the Gene Pattern gene set membership and enrichment values in an html format. The file index.html is the entry point. (ZIP 13 MB) [file 12864_2013_7028_MOESM8_ESM.zip › gset_rnd_es_dist_46.png]

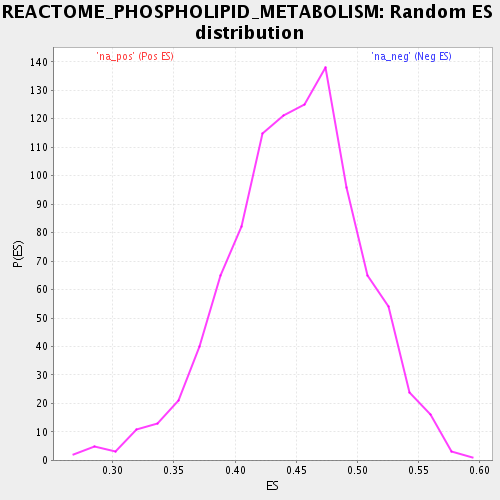

Supplement: Supplementary file 8 — Additional file 8: Contains the Gene Pattern gene set membership and enrichment values in an html format. The file index.html is the entry point. (ZIP 13 MB) [file 12864_2013_7028_MOESM8_ESM.zip › gset_rnd_es_dist_48.png]

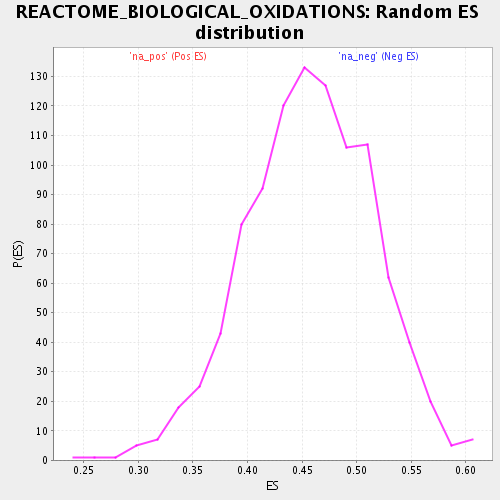

Supplement: Supplementary file 8 — Additional file 8: Contains the Gene Pattern gene set membership and enrichment values in an html format. The file index.html is the entry point. (ZIP 13 MB) [file 12864_2013_7028_MOESM8_ESM.zip › gset_rnd_es_dist_50.png]

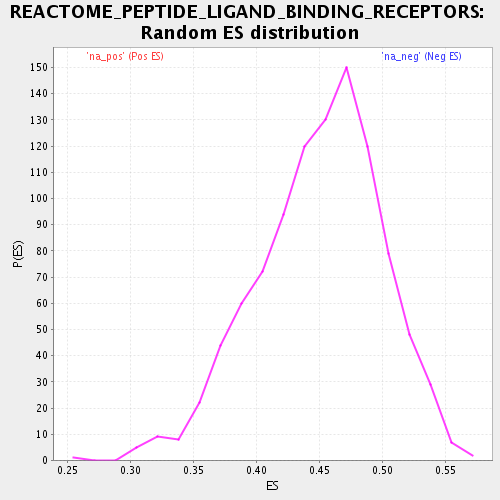

Supplement: Supplementary file 8 — Additional file 8: Contains the Gene Pattern gene set membership and enrichment values in an html format. The file index.html is the entry point. (ZIP 13 MB) [file 12864_2013_7028_MOESM8_ESM.zip › gset_rnd_es_dist_52.png]

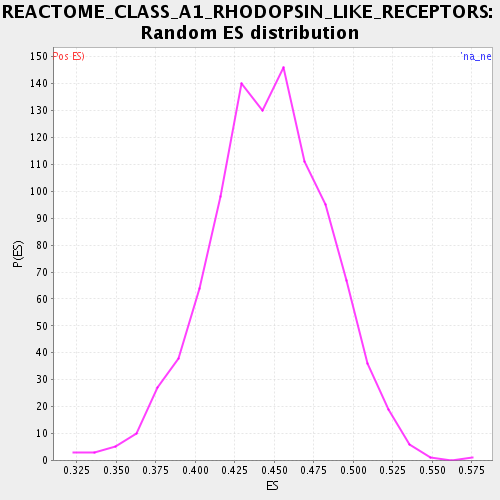

Supplement: Supplementary file 8 — Additional file 8: Contains the Gene Pattern gene set membership and enrichment values in an html format. The file index.html is the entry point. (ZIP 13 MB) [file 12864_2013_7028_MOESM8_ESM.zip › gset_rnd_es_dist_54.png]

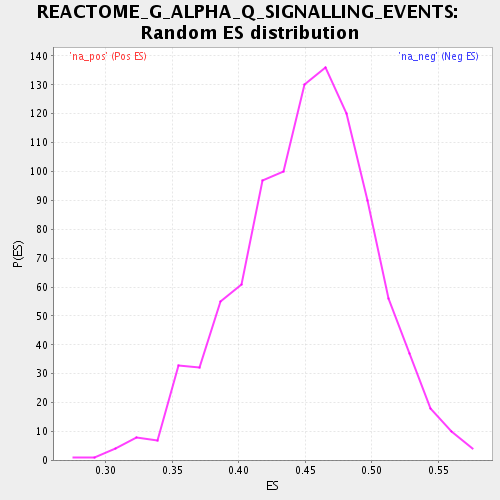

Supplement: Supplementary file 8 — Additional file 8: Contains the Gene Pattern gene set membership and enrichment values in an html format. The file index.html is the entry point. (ZIP 13 MB) [file 12864_2013_7028_MOESM8_ESM.zip › gset_rnd_es_dist_56.png]

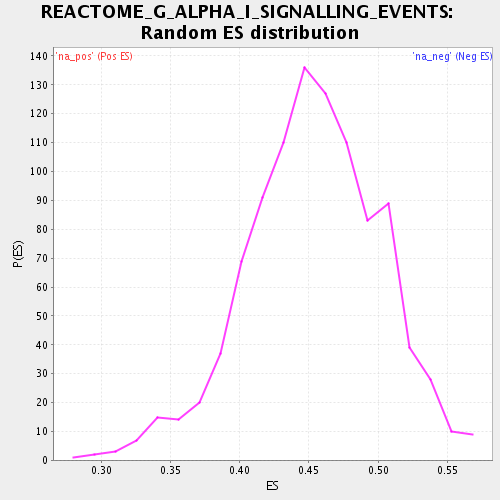

Supplement: Supplementary file 8 — Additional file 8: Contains the Gene Pattern gene set membership and enrichment values in an html format. The file index.html is the entry point. (ZIP 13 MB) [file 12864_2013_7028_MOESM8_ESM.zip › gset_rnd_es_dist_58.png]

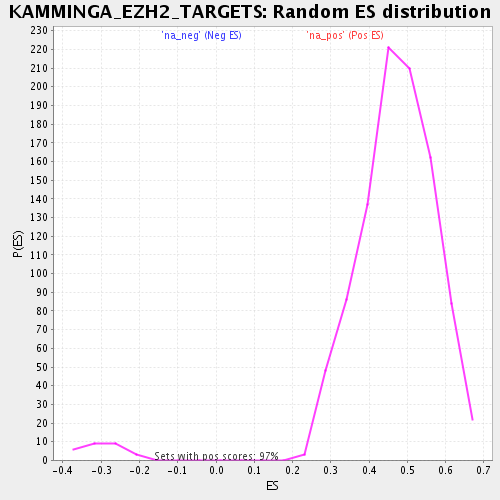

Supplement: Supplementary file 8 — Additional file 8: Contains the Gene Pattern gene set membership and enrichment values in an html format. The file index.html is the entry point. (ZIP 13 MB) [file 12864_2013_7028_MOESM8_ESM.zip › gset_rnd_es_dist_6.png]

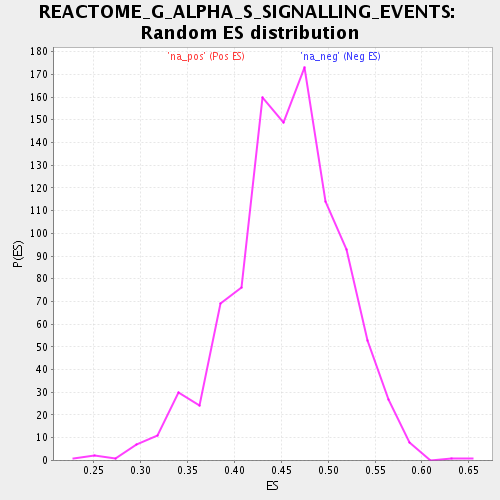

Supplement: Supplementary file 8 — Additional file 8: Contains the Gene Pattern gene set membership and enrichment values in an html format. The file index.html is the entry point. (ZIP 13 MB) [file 12864_2013_7028_MOESM8_ESM.zip › gset_rnd_es_dist_60.png]

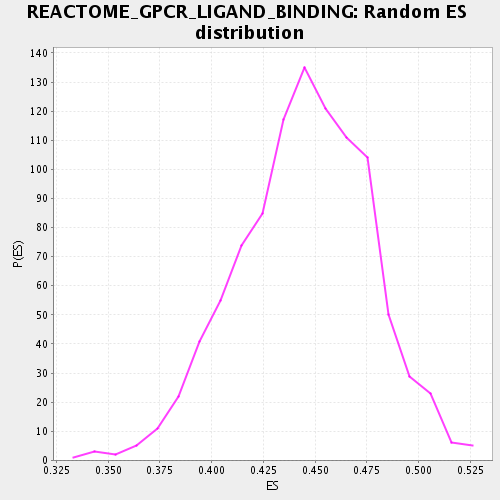

Supplement: Supplementary file 8 — Additional file 8: Contains the Gene Pattern gene set membership and enrichment values in an html format. The file index.html is the entry point. (ZIP 13 MB) [file 12864_2013_7028_MOESM8_ESM.zip › gset_rnd_es_dist_62.png]

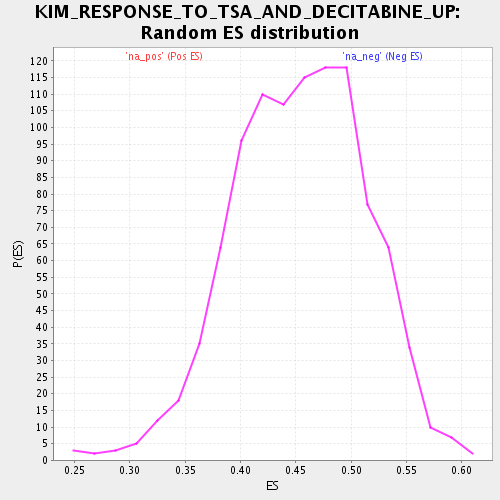

Supplement: Supplementary file 8 — Additional file 8: Contains the Gene Pattern gene set membership and enrichment values in an html format. The file index.html is the entry point. (ZIP 13 MB) [file 12864_2013_7028_MOESM8_ESM.zip › gset_rnd_es_dist_64.png]

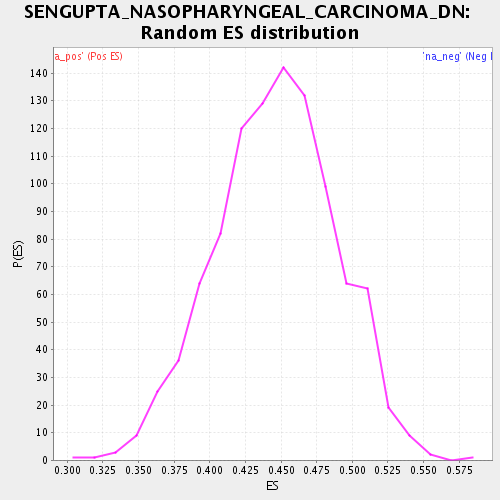

Supplement: Supplementary file 8 — Additional file 8: Contains the Gene Pattern gene set membership and enrichment values in an html format. The file index.html is the entry point. (ZIP 13 MB) [file 12864_2013_7028_MOESM8_ESM.zip › gset_rnd_es_dist_66.png]

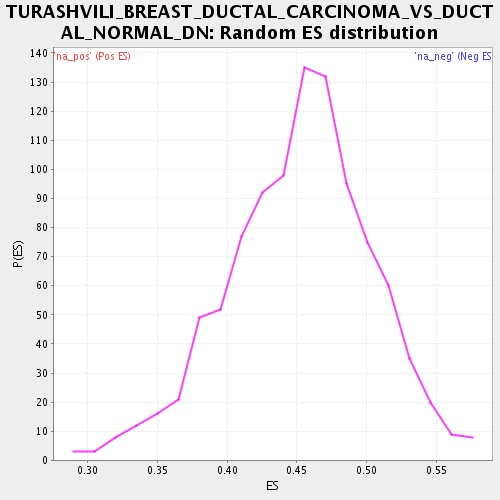

Supplement: Supplementary file 8 — Additional file 8: Contains the Gene Pattern gene set membership and enrichment values in an html format. The file index.html is the entry point. (ZIP 13 MB) [file 12864_2013_7028_MOESM8_ESM.zip › gset_rnd_es_dist_68.png]

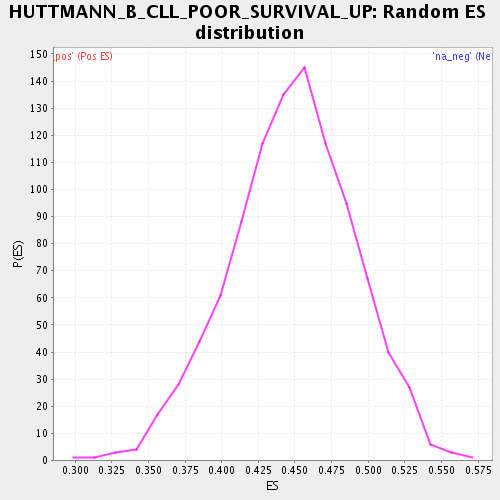

Supplement: Supplementary file 8 — Additional file 8: Contains the Gene Pattern gene set membership and enrichment values in an html format. The file index.html is the entry point. (ZIP 13 MB) [file 12864_2013_7028_MOESM8_ESM.zip › gset_rnd_es_dist_70.png]

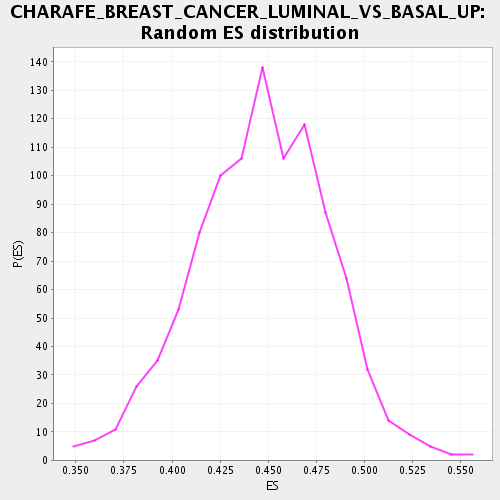

Supplement: Supplementary file 8 — Additional file 8: Contains the Gene Pattern gene set membership and enrichment values in an html format. The file index.html is the entry point. (ZIP 13 MB) [file 12864_2013_7028_MOESM8_ESM.zip › gset_rnd_es_dist_72.png]

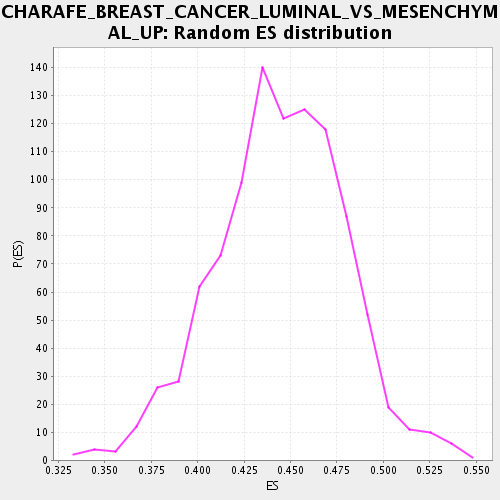

Supplement: Supplementary file 8 — Additional file 8: Contains the Gene Pattern gene set membership and enrichment values in an html format. The file index.html is the entry point. (ZIP 13 MB) [file 12864_2013_7028_MOESM8_ESM.zip › gset_rnd_es_dist_74.png]

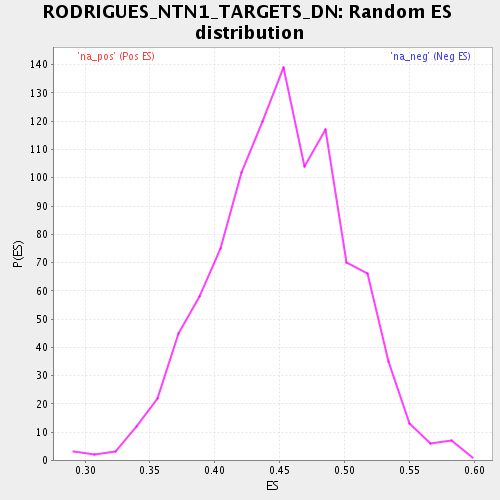

Supplement: Supplementary file 8 — Additional file 8: Contains the Gene Pattern gene set membership and enrichment values in an html format. The file index.html is the entry point. (ZIP 13 MB) [file 12864_2013_7028_MOESM8_ESM.zip › gset_rnd_es_dist_76.png]

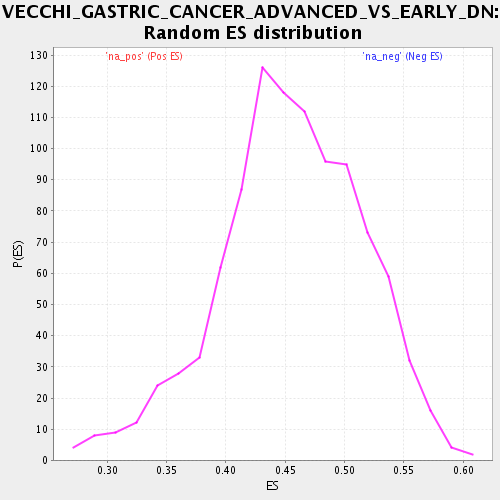

Supplement: Supplementary file 8 — Additional file 8: Contains the Gene Pattern gene set membership and enrichment values in an html format. The file index.html is the entry point. (ZIP 13 MB) [file 12864_2013_7028_MOESM8_ESM.zip › gset_rnd_es_dist_78.png]

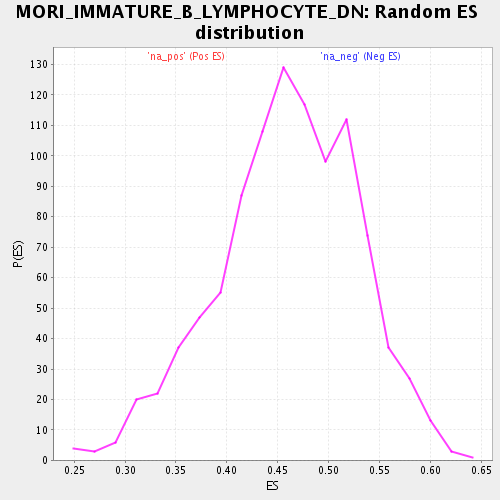

Supplement: Supplementary file 8 — Additional file 8: Contains the Gene Pattern gene set membership and enrichment values in an html format. The file index.html is the entry point. (ZIP 13 MB) [file 12864_2013_7028_MOESM8_ESM.zip › gset_rnd_es_dist_8.png]

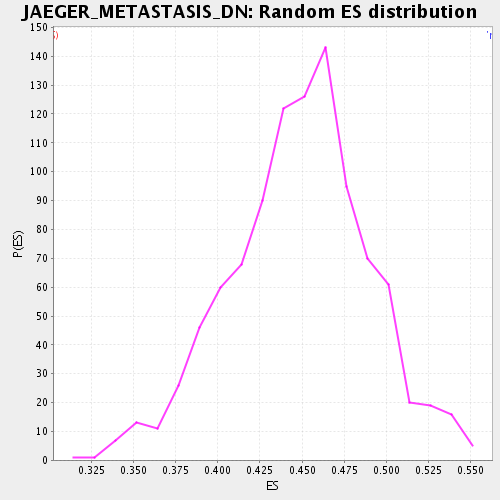

Supplement: Supplementary file 8 — Additional file 8: Contains the Gene Pattern gene set membership and enrichment values in an html format. The file index.html is the entry point. (ZIP 13 MB) [file 12864_2013_7028_MOESM8_ESM.zip › gset_rnd_es_dist_80.png]

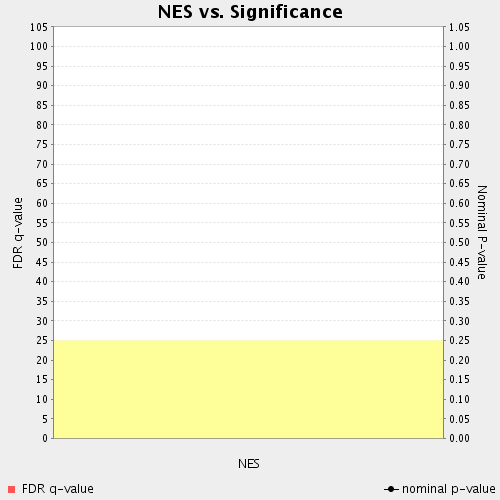

Supplement: Supplementary file 8 — Additional file 8: Contains the Gene Pattern gene set membership and enrichment values in an html format. The file index.html is the entry point. (ZIP 13 MB) [file 12864_2013_7028_MOESM8_ESM.zip › pvalues_vs_nes_plot.png]
